# Single Cell Sequencing Identifies Distinct Cellular Alterations in Impaired Aged and Diabetic Wounds

**DOI:** 10.1111/acel.70217

**Published:** 2025-11-04

**Authors:** Leticia Rojas Cortez, Hamideh Afzali, Zhe Lyu, Min Liu, Adolfo Rojas, Jane Yang, Michael V. Gonzalez, Erick Armingol, Michael Troka, Vinicius Maracaja‐Coutinho, Patricio Smith, Kang I. Ko, Dana T. Graves

**Affiliations:** ^1^ Department of Periodontics, School of Dental Medicine University of Pennsylvania Philadelphia Pennsylvania USA; ^2^ The First Affiliated Hospital, College of Medicine Zhejiang University Hangzhou China; ^3^ Advanced Center for Chronic Diseases‐ACCDiS, Facultad de Ciencias Químicas y Farmacéuticas Universidad de Chile Santiago Chile; ^4^ Center for Cytokine Storm Treatment & Laboratory, Department of Medicine University of Pennsylvania Philadelphia Pennsylvania USA; ^5^ Bioinformatics and Systems Biology Graduate Program University of California, San Diego La Jolla California USA; ^6^ School of Dentistry, Faculty of Medicine Pontificia Universidad Católica de Chile Santiago Chile

**Keywords:** aging, gingiva, hyperglycemia, oral mucosa, repair, stromal

## Abstract

Impaired wound healing in aged and diabetic wounds involves complex cellular dysregulation that hinders tissue repair. Using single‐cell RNA sequencing (scRNA‐seq) and validation techniques, we investigated impaired wound healing to identify whether there were significant changes linked to each condition. Comparative mucosal wound analysis revealed distinct differences between diabetic and normoglycemic (NG)‐aged mice, which had an impact on connective tissue formation and epithelial closure. Wounds in NG‐aged mice exhibited prolonged granulation tissue and upregulation of genes linked to chemotaxis, cell migration, neutrophil degranulation, and antimicrobial defense pathways compared to the diabetic wounds. In comparison to healing in young animals, wounds in NG‐aged mice had a shift in fibroblast subtypes with fewer matrix‐producing myofibroblasts and increased inflammatory fibroblasts. Furthermore, wounds in NG‐aged mice versus wounds in diabetic mice had an upregulation of lytic enzymes, with striking differences in cathepsin‐expressing fibroblasts. Since diabetic wounds healed more slowly than wounds in NG‐aged mice, the results suggest that the upregulation of lytic enzymes that characterized diabetic wounds is particularly damaging to healing. In addition to the transcriptional differences, pseudotime analysis revealed that fibroblasts in wounds from diabetic mice progressed towards a protease‐enriched state, while those in aged mice shifted towards an inflammatory phenotype. This is the first study to directly compare aged and diabetic healing at the single‐cell level and provides distinct molecular mechanisms that may allow more precise therapeutic targets to improve healing in aged and diabetic wounds.

## Graphical abstract

Single‐cell RNA sequencing revealed distinct fibroblast dynamics in aged and diabetic wounds. Aged mice showed reduced matrix‐producing myofibroblasts and increased inflammatory fibroblasts, while diabetic mice exhibited elevated lytic enzyme expression. These differences reflect the impact of diabetes and aging on fibroblast subtypes that may negatively impair healing in each condition.

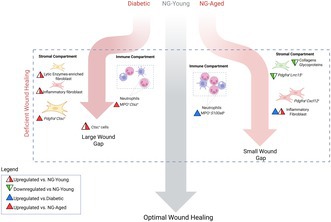

## 1 Introduction

Wound healing is a physiological response activated by injury and is comprised of four main phases—hemostasis, inflammation, proliferation, and remodeling (Wilkinson and Hardman 2020). These dynamic stages require tight coordination and are dysregulated in abnormal healing responses by conditions such as diabetes, smoking, infection, and aging (Beyene et al. 2020; Ko et al. 2021; Thanapaul et al. 2021). Impaired healing is frequently associated with persistent inflammation and reduced new tissue formation (Ko et al. 2021; Zhu et al. 2022). Delayed healing in diabetic and normoglycemic (NG)‐aged individuals can lead to chronic, non‐healing cutaneous and mucosal wounds and is therefore a significant health risk (Kim et al. 2019; Keyes et al. 2016). The distinguishing factors that lead to delayed healing in diabetic and NG‐wounds in the elderly remain incompletely understood, which is important in identifying more specific pathologic mechanisms and therapeutic targets.

There is a substantial body of evidence that an inflammatory phenotype drives deficient healing in aged and diabetic wounds (Al‐Rikabi et al. 2021; Vu et al. 2022; Wong et al. 2015). A significant part of this may be due to neutrophils. An increase in neutrophils has been shown to impair healing through multiple mechanisms, including increased NETosis in both skin and mucosal wounds (Wong et al. 2015; Sabbatini et al. 2021; Fadini et al. 2016; Kleinstein et al. 2021; Wang et al. 2022). Diabetes primes neutrophils to undergo NETosis, which impairs wound healing (Wong et al. 2015). Under normal conditions, neutrophils are needed for repair, particularly in the early phases. In contrast, prolonged or excessive neutrophil activity can impair healing in both skin and mucosal wounds as shown in multiple studies. This is largely due to the release of destructive enzymes, reactive oxygen species, and NETosis (Wong et al. 2015; Fadini et al. 2016). Thus, there is substantial literature supporting that in the early phases of healing neutrophils play an important and positive role, while their prolonged presence is problematic in both aged and diabetic wounds (Thanapaul et al. 2021; Vu et al. 2022; Wong et al. 2015; Fadini et al. 2016). In support of the former, a delay in the acute phase response of wound healing negatively impacts the healing process (Thanapaul et al. 2021; Vu et al. 2022). It is noteworthy that the features that distinguish aged and diabetic healing have not been substantially investigated although both impair healing and involve dysregulated inflammatory pathways (Kita et al. 2024; Parella et al. 2023). The purpose of our study was to build upon these previous studies to investigate by single cell RNAseq (scRNA‐seq) specific aspects of the healing process that distinguish aged from diabetic wounds, particularly in mucosal wounds, which have distinct healing characteristics (Ko et al. 2023; Almet et al. 2024). Furthermore, we focused on fibroblast heterogeneity, given the recent interest in fibroblast subtypes and how they can promote or inhibit healing responses. Here, we found that the healing of diabetic mucosal wounds was more impaired than similar wounds in aged animals, although both had reduced healing compared to matched wounds from young normoglycemic animals. scRNA‐seq uncovered striking differences in neutrophils and fibroblasts. Diabetic wounds were linked to greater matrix degradation pathways with heightened expression of matrix‐degrading cysteine proteases than wounds in aged mice. Wounds in aged animals were linked to increased pro‐inflammatory signatures marked by elevated chemokine expression, neutrophil recruitment, and more persistent granulation tissue than wounds in diabetic mice. These results pinpoint distinct molecular mechanisms driving impaired healing in both conditions, revealing potential cell‐ and pathway‐based targets to enhance tissue repair in diabetes and aging.

## 2 Results

### 2.1 Diabetic and NG‐Aged Oral Wounds Exhibit Different Healing Characteristics and Have Altered Fibroblast and Neutrophil Composition

**FIGURE 1:**
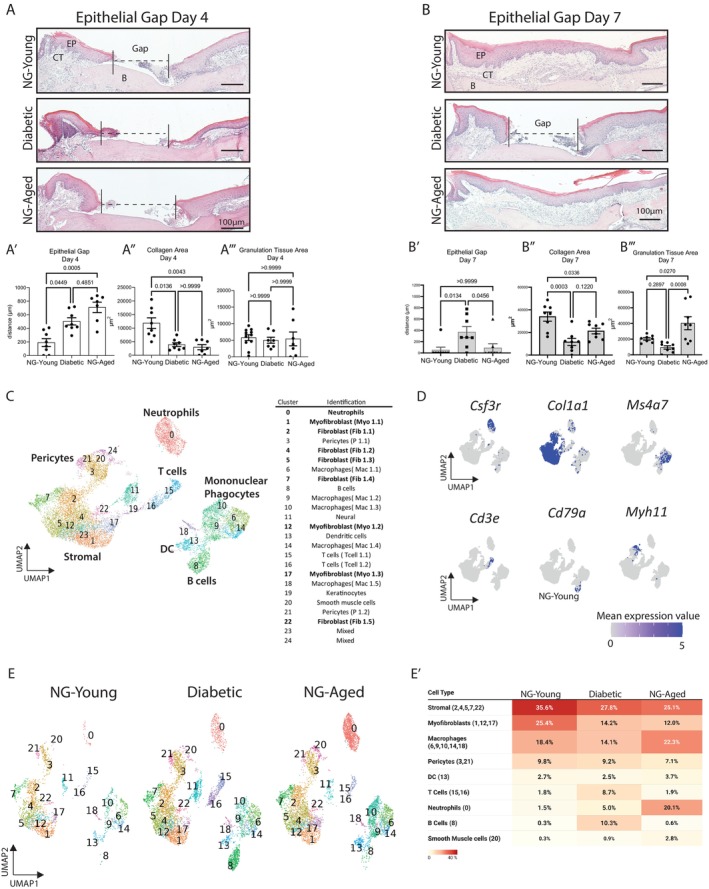
Histologic and scRNA‐seq analysis of wounded gingiva from NG‐Young, diabetic, and NG‐Aged mice. (A) Representative hematoxylin and eosin (H&E) staining of wounded palatal mucosa in day 4 wounds. (A′) The wound gap (dashed line) was measured as the distance between the wound edges (μm). Masson‐Trichrome stained sections were used to measure: (A″) Collagen area (μm^2^). (A‴) Granulation tissue area (μm^2^). (B) Representative H&E staining of wounded palatal mucosa in 7 day wounds. (B′) Wound gap measurement. (B″) Collagen area. (B‴) Granulation tissue area. Data represents mean ± SEM (*n* = 7–8 mice per group). One‐way ANOVA post hoc Tukey. *p* values are shown numerically; statistical significance was defined as *p* < 0.05. EP, epithelium; CT, connective tissue; B, palatal bone. Scale = 100 μm. (C) scRNA‐seq data of major cell types obtained from wounded NG‐Young, diabetic, and NG‐Aged palatal mucosa at 7 day wounds represented as uniform manifold approximation and projection (UMAP). (D) UMAP of mean expression values of genes representing specific cell types: neutrophils (*Csf3r*), stromal cells (*Col1a1*), macrophages (*Ms4a7*), T lymphocytes (*Cd3e*), B lymphocytes (*Cd79a*), smooth muscle cells (*Myh11*), keratinocytes (*Krt14*) and neural cells (*Plp1*). (E) UMAP plots split by conditions, and (E′) proportion heatmap table showing the percentage of major cell types in wounded NG‐Young, diabetic, and NG‐Aged palatal mucosa in 7 day wounds. Values represent percentages.

We created full‐thickness excisional 1‐mm wounds in the palatal mucosa to examine the effects of diabetes and aging on wound closure (Figure 1A,B). At day 4 post‐wounding (PW), diabetic and NG‐Aged mice showed significantly greater wound gaps between the epithelial edges compared to their NG‐Young counterparts (Figure 1A′). On day 7, the wound gap was still present in the diabetic group while the wounds had closed in NG‐Aged and NG‐Young groups (*p* < 0.05) (Figure 1B′). Masson's trichrome stained sections (Figure 1″,B‴, Figure S1A) revealed that the area of new connective tissue formation was reduced in diabetic and NG‐Aged wounds compared to control NG‐Young mice on day 4 and 7 post‐wounding (*p* < 0.05) (Figure 1A″,B″). The area of granulation tissue was similar for all groups in day 4 wounds but significantly greater in the wounds from NG‐Aged mice compared to NG‐Young and diabetic groups in day 7 wounds, demonstrating greater persistence (*p* < 0.05) (Figure 1A‴,B‴).

To gain further insight into the healing mucosal wounds, scRNA‐seq analysis was carried out focusing on fibroblasts and leukocytes. Cells were isolated from day 7 wounds and sorted by flow cytometry to obtain live cells by excluding dead DAPI^+^ cells. Lineage‐negative cells were removed as Ter119^+^ erythrocytes, CD31^+^ endothelial cells, and Epcam^+^ epithelial cells. After stringent quality control (Figure S1B), normalization, and integration as described in the Methods, the resulting dataset consisted of 15,449 cells and 41,764 genes. The integrated dataset was clustered, and the major cell groups were identified as mononuclear phagocytes, neutrophils, T cells, and stromal cells, with multiple distinct cell clusters in the groups. Genes enriched in each cluster are shown in Table S1, and the top 10 genes per cluster are illustrated as a dot plot (Figure S1C). Cell type assignation by EnrichR is presented in Table S2. Cell clusters were further annotated by cellular markers: *Col1a1* ^+^ for mesenchymal cells, *Csfr3* ^+^ for neutrophils, *Ms4a7* ^+^ for mononuclear phagocytes, *Cd3e* ^+^ for T cells, and *Cd79a* ^+^ for B cells (Figure 1D). Analysis of cell clusters in wounds from NG‐Young, NG‐Aged, and diabetic mice revealed a striking difference in cellular composition, particularly for neutrophil populations. NG‐Aged mice exhibited a 13‐fold increase in the proportion of neutrophils (cluster 0) compared to young mice and a 4‐fold increase compared to diabetic mice (Figure 1E,E′).

In the stromal compartment, an overall decrease in fibroblasts (Clusters 2, 4, 5, 6, and 22) and myofibroblasts (Clusters 1,12, and 17) was observed in wounds from diabetic and NG‐Aged mice compared to the NG‐Young group (Figure 1E′). Macrophage, pericyte, and dendritic cells were less affected. Both T and B cells were increased in diabetic wounds compared to wounds in NG‐Aged and NG‐Young mice (Figure 1E′). Taken together, scRNA‐seq results point to differences in neutrophil and stromal cell populations between the mucosal wounds of diabetic and aged groups.

### 2.2 Changes in Neutrophil Gene Expression Profile in Diabetic and Mucosal Wounds of Aged Mice

**FIGURE 2:**
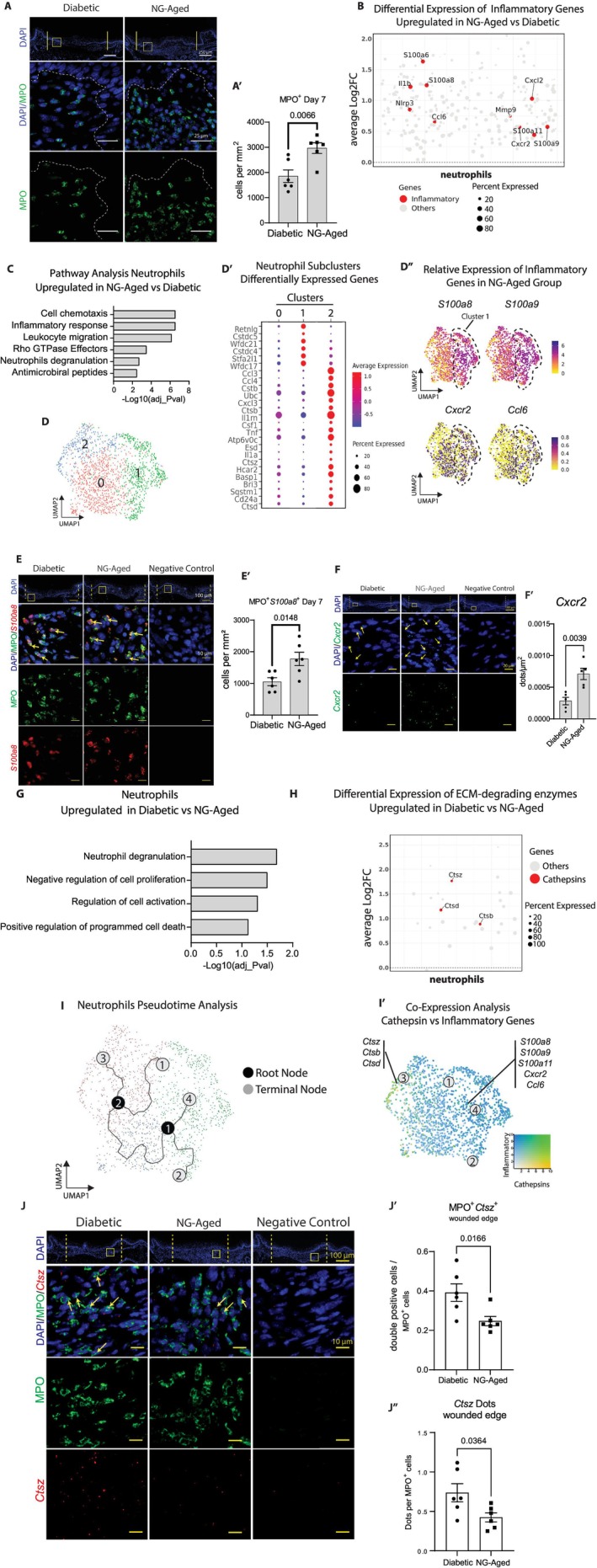
Neutrophils in diabetic and NG‐Aged oral mucosal wounds exhibit functional differences. (A) Representative immunofluorescent images showing myeloperoxidase (MPO) expression in diabetic and NG‐Aged conditions in 7 day wounds. Vertical yellow lines indicate wound edges. Yellow box highlights the region of interest (ROI). (A′) Bar graph depicts MPO‐positive cells per mm^2^ (*n* = 6). (B) Jitterplot of inflammation‐associated genes that are differentially expressed in neutrophils in the NG‐Aged group versus diabetic group. Genes that are significantly upregulated (*p* adj value < 0.05) are highlighted in red. The size of the dot depicts the percent of cells expressing the gene. (C) Bar graph of pathways significantly enriched in neutrophils in the NG‐Aged group compared to the diabetic counterpart. (D) UMAP plot of neutrophil clusters. (D′) Dotplot of differentially expressed genes in neutrophils. Size of the dot depicts the percent of cells expressing the gene. (D′) UMAP of single cell expression of *S100a8*, *S100a9*, *Cxcr2*, and *Ccl6* in the neutrophil from NG‐Aged group. Black dotted line circumscribes cluster 1. (E) Immunofluorescent images showing MPO (green) and *S100a8* (red) positive cells across conditions. Dotted vertical lines represent the wound edge; yellow box highlights the ROI; yellow arrows point to MPO^+^ *S100a8* ^+^ double‐positive cells. (E′) Quantification of MPO^+^ *S100a8* ^+^ double‐positive cells per mm^2^ (*n* = 6). (F) RNAScope of *Cxcr2* gene expression in diabetic and NG‐Aged conditions in 7 day wounds. Dotted vertical lines indicate wound edges; yellow box highlights the ROI; yellow arrows point to *Ccr2* ^+^ cells. (F′) Quantification of MPO^+^ *Cxcr2* ^+^ double positive cells per μm^2^ (*n* = 6). (G) Bar graph of pathways significantly enriched in neutrophils in the diabetic group compared to the NG‐Aged counterpart. (H) Jitterplot of cathepsin genes differentially expressed in neutrophils from diabetic versus NG‐Aged wounds. Genes that are significantly upregulated are highlighted in red. (I) Monocle3‐pseudotime trajectories of neutrophils projected in UMAP. (I′) Co‐expression of cathepsin and inflammatory genes visualized in neutrophil UMAP. (J) Multiplexed RNAScope in situ hybridization of *Ctsz* transcripts localized to MPO^+^ neutrophils. (J′) Percent MPO^+^ *Ctsz* ^+^ double‐positive cells per total number of cells. (J″) Quantification of *Ctsz* ^+^ dots per MPO^+^ cells. Low magnification scale bar: 250 μm and 25 μm in high magnification images (A) 100 μm and 10 μm in (D). Bar graphs (A′ and D′) represent mean ± SEM, *p*‐values were calculated using unpaired two‐tailed Student's *t*‐test for pairwise comparison between diabetic and NG‐Aged groups. *p* values are shown numerically; statistical significance was defined as *p* < 0.05.

The changes in neutrophil numbers from the scRNA‐seq results were confirmed by immunofluorescence with an antibody specific to myeloperoxidase (MPO) (Figure 2A). Wounds from the NG‐Aged group had a 60% increase in neutrophils per tissue area compared to the diabetic group (*p* < 0.05) (Figure 2A′). Further scRNA‐seq analysis of neutrophils showed that the NG‐Aged group had upregulated expression of several inflammatory mediators including S100a family members, interleukins, and chemokines and chemokine receptors when compared to diabetic mice (adj_*p*val < 0.001) (Figure 2B). The pathway analysis revealed that these upregulated genes from neutrophils in the NG‐Aged group were implicated in enhanced chemotaxis and migration, inflammatory, neutrophil degranulation, and antimicrobial defense pathways compared to the diabetic group (adj_*p*val < 0.001) (Figure 2C, Table S3). Neutrophil clusters 1 and 2 exhibited unique gene expression patterns (Figure 2D′). Neutrophil cluster 1 had upregulated expression of genes from the cystatin (*Cstdc5* & *Cstdc4*) and whey acidic protein (*Wfdc21* & *Wfdc17*) families (adj_*p*val < 0.001), which are implicated in modulating the function of cysteine proteases. Neutrophil cluster 2 exhibited an enrichment in chemokines and cathepsins compared to all other clusters (adj_*p*val < 0.001) (Figure 2D′). Neutrophil cluster 1 compared to other neutrophil clusters had upregulation of *S100a8* in wounds from NG‐Aged mice (Figure 2D″). The upregulation of neutrophils with an inflammatory signature was confirmed by double immunofluorescence with antibodies specific for MPO and *S100a8*. Wounds from the NG‐Aged group had a 68% increase in MPO^+^ *S100a8* ^+^ double‐positive cells per area compared to wounds from diabetic mice at day 7 (*p* < 0.05) (Figure 2E–E″). RNA in situ hybridization (RNAscope) was performed to detect *Cxcr2* mRNA transcripts (Figure 2F). We found that *Cxcr2* mRNA expression was upregulated approximately 3‐fold at the wound edge of the NG‐Aged versus diabetic group (*p* < 0.05) (Figure 2F′).

We performed pathway analysis using the upregulated genes in neutrophils from diabetic wounds compared to the NG‐Aged group. This indicates an upregulation of genes linked to neutrophil degranulation, negative regulation of cell proliferation, and positive regulation of programmed cell death pathways in the diabetic wounds compared to wounds from the NG‐Aged group (Figure 2G, Table S3). Interestingly, several enriched pathways involved genes from the cathepsin family (adj_*p*val < 0.001), particularly *Ctsz*, *Ctsd*, and *Ctsb* proteases that were upregulated in wounds from diabetic group compared to wounds from NG‐Aged mice (adj_*p*val < 0.001) (Figure 2H).

To better understand how the neutrophil clusters were related to each other, we interrogated the dataset using Monocle 3 trajectory analysis. We identified four terminal branch points (light circles, Figure 2I) emerging from two root nodes that shared an origin in Cluster 0 (dark circles, Figure 2I). We examined whether inflammatory‐ and cathepsin‐enriched neutrophil clusters were localized to the terminal branch points. Cathepsin genes such as *Ctsz*, *Ctsb*, and *Ctsd* mapped to branch point #3 while S100a‐associated transcripts mapped to branch point #4 (Figure 2I′). We next carried out a combined immunofluorescence and RNAscope analysis to quantify MPO^+^ *Ctsz* ^+^ cells (Figure 2J). The number of MPO^+^ *Ctsz* ^+^ cells were significantly increased by 62% in diabetic wound compared to NG‐Aged groups (*p* < 0.05) (Figure 2J′). Diabetic wounds also had a 73% increase in *Ctsz* transcript numbers per MPO^+^ cell in comparison to the NG‐Aged group (*p* < 0.05) (Figure 2J”). These data show distinct differences in how aging and diabetes impact neutrophil phenotype in wound healing, with increased expression of proteolytic enzymes in neutrophils from diabetic wounds and enhanced inflammatory mediator expression by neutrophils in wounds in the elderly.

### 2.3 Lytic Enzyme Expression Distinguishes Fibroblast Role in Diabetic Wounded Mice

**FIGURE 3:**
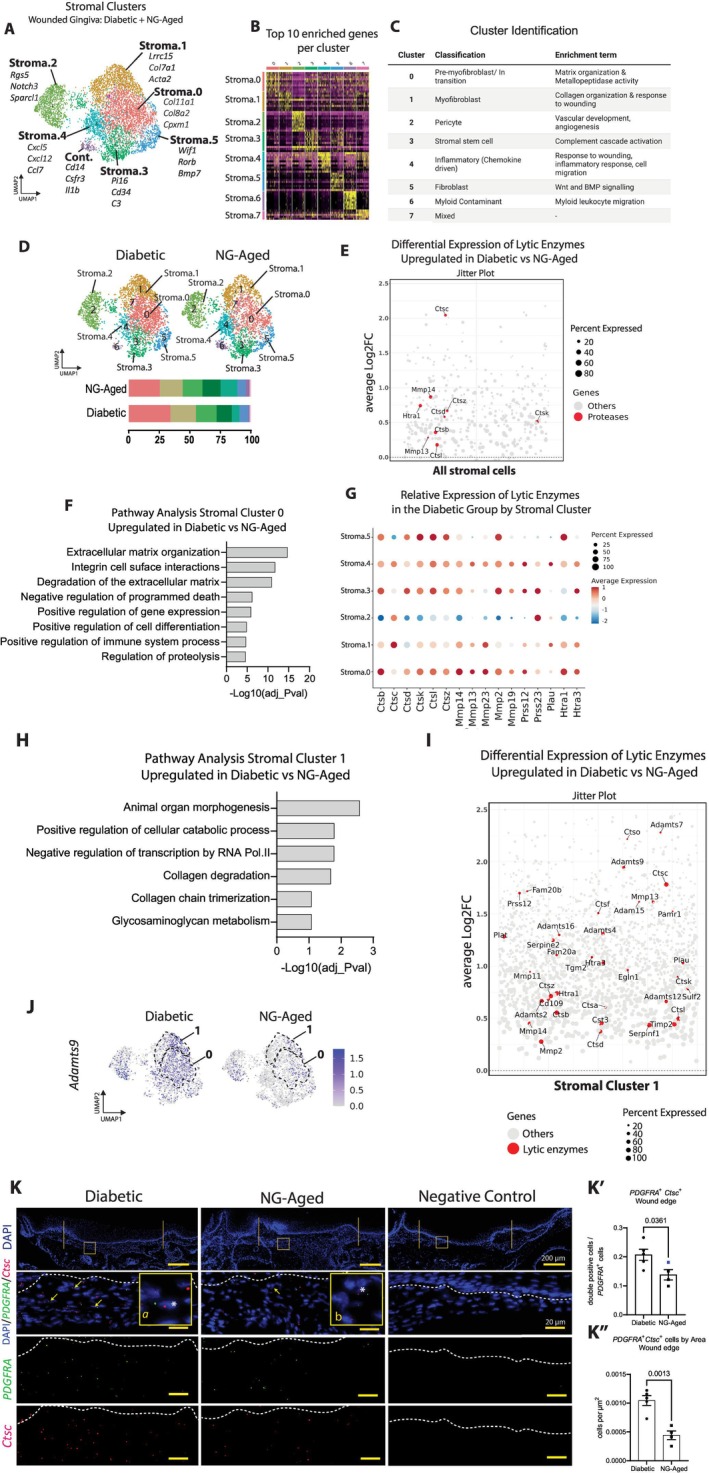
Lytic enzyme expression is characteristic of stromal cells in diabetic mucosal wounds in comparison to NG‐Aged wounds in 7 day wounds. (A) UMAP plot of stromal subclusters. Gene labels designate top 3 differentially expressed genes for each cluster. (B) Heatmap of differentially upregulated genes for each stromal subcluster. (C) List of stromal cell type classification and significantly enriched pathways for each cluster by EnrichR. (D) UMAP of stromal subclusters split up by condition and the proportion of each subcluster normalized by total stromal cell numbers (bottom). (E) Jitterplot of lytic‐enzyme genes differentially expressed in stromal cells of the diabetic group versus NG‐Aged group. Significantly expressed genes (*p*adj‐value < 0.05) are highlighted in red. Size of the dot depicts the percent of cells expressing the gene. (F) Bar graph of pathways significantly enriched in stromal cluster 0 in the diabetic group compared to the NG‐Aged counterpart. (G) Dotplot depicting relative expression of lytic enzyme genes across stromal clusters in the diabetic group. (H) Bar graph of pathways significantly enriched in stromal cluster 1 in the diabetic group compared to the NG‐Aged group. (I) Jitterplot of lytic enzymes genes differentially expressed in stromal cluster 1 in the diabetic group versus NG‐Aged group. Significantly expressed genes (*p*adj value < 0.05) are highlighted in red. (J) Feature plot showing *Adamst9* expression across conditions. Dotted lines circumscribe Clusters 0 and 1. (K) RNAScope in situ hybridization of *Pdgfra* and *Ctsc* transcripts (*n* = 4–5). Solid vertical lines indicate the wound edge; white dotted line demarcates epithelial‐connective tissue junction. (a, b) ×200 zoomed area. (*) indicates *Pdgfra* ^+^ *Ctsc* ^+^ double‐positive cells. Low magnification scale bar: 200 μm; high magnification: 20 μm. (K′) Quantification of *Pdgfra* ^+^ *Ctsc* ^+^ cells per *Pdgfra* ^+^ cells, and (K″) per μm^2^ (*n* = 4–5). Data represent mean ± SEM, *p* values were calculated using unpaired two‐tailed Student's t‐test for pairwise comparison between diabetic and NG‐Aged groups. *p* values are shown numerically; statistical significance was defined as *p* < 0.05.

Stromal cells were subclustered and examined in from wounds of diabetic, aged and young mice to achieve higher resolution (Figure 3A). Eight clusters were identified and had distinctive gene signatures as shown in the heat map (Figure 3B, Table S3). The identity of each cluster (Figure 3C) was established by the characteristic transcriptomic profile identified by EnrichR pathway analysis (Table S5). The largest cluster (0) displayed features of pre‐myofibroblasts based on the upregulation of matrix organization functions in the absence of prototypic myofibroblast marker *Acta2* (adj_*p*val < 0.001) (Figure 3C, Table S5). Cluster 1 exhibited upregulation of myofibroblast markers *Acta2* and *Lrrc15* linked to wound healing responses (adj_*p*val < 0.001) (Figure 3C, Table S5). Pericytes (cluster 2) were identified by a transcript signature of *Rgs5*, *Notch3*, and *Sparcl1* (Figure 3A) and transcripts related to pro‐angiogenesis (Figure 3C, Table S5). Cluster 3 gene signature exhibited high expression of *Pi16 and Cd34*, markers of stromal progenitors (Gao et al. 2024). Inflammatory fibroblasts were identified by marked expression of *Cxcl5*, *Cxcl12*, and *Ccl7* in cluster 4 (Figure 3A–C, Table S5) (adj_*p*val < 0.001). The *Wif1* transcript, which encodes the WNT inhibitor, was upregulated in cluster 5, an important regulator of fibroblast differentiation in mucosal wounds (Ko et al. 2023). Clusters 6 and 7 were unidentified and excluded for further analysis (Table S4). There was generally little difference in the number of cells in each cluster based on condition (NG‐Aged vs. diabetic) except for cluster 4, which had a 2.2‐fold increase in wounds from NG‐Aged vs. diabetic mice (Figure 3D).

Differential gene expression analysis of all stromal cells (Table S6) identified cathepsins such as cathepsin C (*Ctsc*) and *Ctsz*, metalloproteases, and other serine proteases as upregulated in wounded diabetic mice compared to wounds in NG‐Aged mice (adj_*p*val < 0.001) (Figure 3E). Pathway analysis of stromal cluster 0 indicated that extracellular matrix organization and degradation were significantly upregulated in wounds from diabetic mice in comparison to the NG‐Aged group (adj_*p*val < 0.001) (Figure 3F). Moreover, protease transcripts were upregulated in wounds from diabetic versus NG‐Aged animals throughout the stromal clusters except for pericytes, stromal cluster 2 (Figure 3G). Stromal cluster 1 had pathway enrichment related to catabolic and collagen degradation pathways, among others, in wounds from diabetic versus NG‐Aged mice (adj_*p*val < 0.001) (Figure 3H). Consistent with this, a broad range of proteolytic enzymes from the Adamts, cathepsins, and MMPs families were upregulated in stromal cluster 1 in wounds from the diabetic versus NG‐Aged groups (adj_*p*val < 0.001) (Figure 6I). *Adamts9* transcripts were largely associated with stroma 0 and stroma 1 clusters in the diabetic as shown by their UMAP distribution (Figure 3J). RNAscope experiments examined *Ctsc* expression in fibroblasts identified by the pan‐marker *Pdgfra*. *Ctsc* mRNA fluorescent signal was detectable in *Pdgfra* ^+^ fibroblasts in the wound edge (Figure 3K). The percent of fibroblasts that expressed *Ctsc* was 53% higher in the diabetic compared to NG‐Aged mice (*p* < 0.05) (Figure 3K′). Moreover, the density of double‐positive cells per mm^2^ was 2.5‐fold higher in wounds from diabetic mice than NG‐Aged mice (*p* < 0.05) (Figure 3K″). Taken together, the scRNAseq and RNAscope data support that the enhanced proteolytic gene signature in fibroblasts is a distinguishing feature of diabetic wounds compared to wounds in aged mice.

### 2.4 Inflammatory Fibroblast Transcriptome in Wounds From NG‐Aged and Diabetic Mice

**FIGURE 4:**
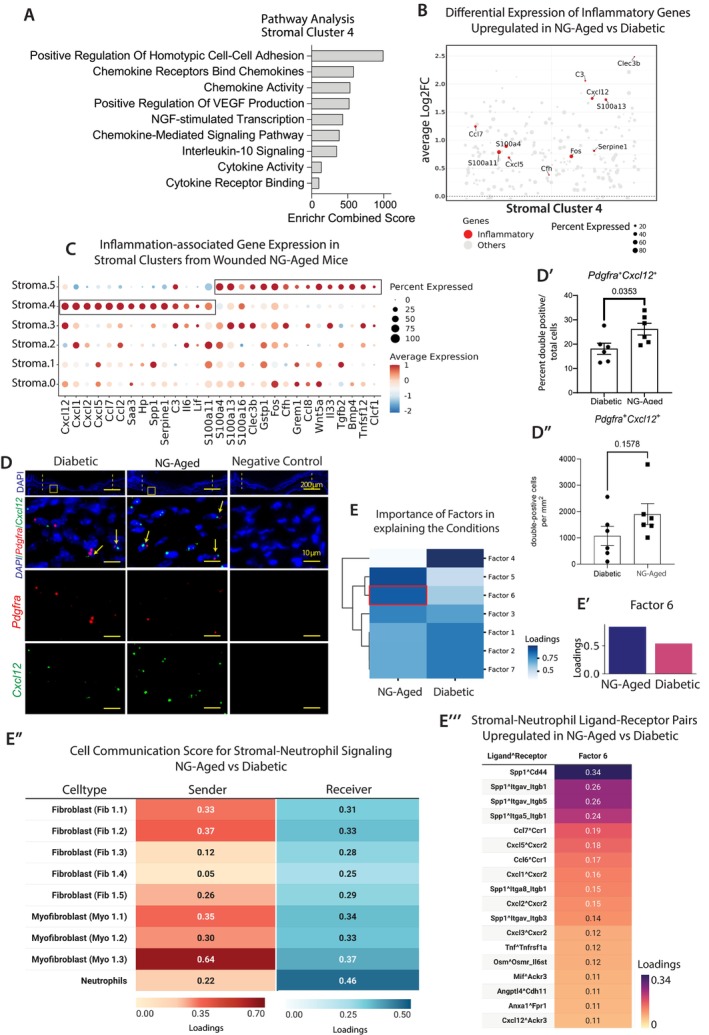
Proinflammatory fibroblasts are enriched in NG‐Aged oral mucosal wounds. Stromal cells from diabetic and NG‐Aged wounds in 7 day wounds were subclustered, differentially expressed genes calculated, pathway analysis performed and validated using in situ hybridization. (A) Bar graph of GO and Reactome pathway analyses for genes significantly enriched in stromal cluster 4 compared to other clusters. (B) Jitterplot of inflammatory genes differentially expressed in stromal cluster 4 in NG‐Aged group versus diabetic group. Significantly expressed genes (*p*adj value < 0.05) are highlighted in red. (C) Dotplot depicting relative expression of inflammation‐associated genes across stromal clusters in the NG‐Aged group. The size of the dot depicts the percent of cells expressing the gene. (D) RNAScope in situ hybridization of *Pdgfra* (red) and *Cxcl12* (green) (*n* = 6). Yellow arrows point to *Pdgfra* ^+^ *Cxcl12* ^+^ double‐positive cells. (D′) Percent of *Pdgfra* ^+^ *Cxcl12* ^+^ cells per total cells. Data represent mean ± SEM, *p* values were calculated using unpaired two‐tailed Student's *t*‐test for pairwise comparison between diabetic and NG‐Aged groups. *p* values are shown numerically; statistical significance was defined as *p* < 0.05. (E) Heatmap indicating the cell‐cell communication factors associated with each condition and expressed as score values. Legend indicates the loading values (light blue: low; dark blue: high). (E′) Factor 6 reflects the communication changes in NG‐Aged group versus diabetic group. (E″) Heatmap depicting communication scores where fibroblast subtypes are the source of ligand (sender) and neutrophils are the receiver cells. (E‴) Ligand‐receptor pairs associated with the communication program (Factor 6). Data represent mean communication score.

Inflammatory fibroblasts have a significant impact on wound healing outcomes (Sinha et al. 2022), therefore, we next focused on stromal cluster 4, which had an inflammatory profile (Figure 3C). Pathway analysis indicated that compared to other stromal clusters, stromal cluster 4 has upregulated pathways related to cytokine and chemokine signaling (adj_*p*val < 0.001) (Figure 4A). The differential gene expression pattern in cluster 4 showed upregulation of several chemokines and other inflammatory mediators in wounds from NG‐Aged mice compared to the diabetic (adj_*p*val < 0.001) (Figure 4B). Although stromal cluster 5 did not exhibit an overall pro‐inflammatory profile compared to other clusters (Figure 4C), its expression of pro‐inflammatory genes was significantly upregulated in the NG‐Aged group compared to the young diabetic, suggesting the acquisition of a more inflammatory phenotype induced by aging (Figure 4C). To confirm chemokine upregulation in fibroblasts in NG‐Aged wounds, RNAscope was carried out with probes specific to *Pdgfra* and *Cxcl12* (Figure 4D). The percentage of fibroblasts that expressed *Cxcl12* was increased by 45% in wounds of NG‐Aged versus diabetic mice (*p* < 0.05) (Figure 4D′), consistent with scRNA‐seq result.

Enhanced cytokine expression from the fibroblasts may be linked to increased numbers and hyperinflammatory state of neutrophils in NG‐Aged mice. To gain insight into potential signaling between fibroblasts and neutrophils, we examined inferred cell–cell communication networks using Tensor‐cell2cell (Figure 4E, Figure S2A–D) focusing on the clusters representing fibroblasts and neutrophils in Figure 1C. First, we summarized the gene signature similarity between stromal clusters (Figure 1C) and the stromal subclusters (Figure 4A) in Table S7. We identified a similar proinflammatory profile between cluster 4 (Figure 1C) and stromal cluster 4 (Figure 4A) by expressing *Cxcl5*, *Cxcl12*, and *Ccl7* (Table S7, Sheet “1.Similarity among Seurat objs”). Myofibroblast gene profiles observed in cluster 1 and 12 (Figure 1C) were similar to those in stromal cluster 1, including *Lrrc15*, *Lrrc17*, *Loxl2*, *and Tnc* (Table S7, Sheet “1. Similarity among Seurat objs”). Of the seven factors identified by Tensor‐cell2cell, Factors 5 and 6 best represented the upregulated ligand‐receptor pair interactomes in the wounds of NG‐Aged mice compared to the diabetic group (Figure 4E–E′). We focused on Factor 6, as Factor 5 shows lower ligand‐receptor inference values for similar ligand‐receptor pairs than Factor 6 (Table S8). In NG‐Aged mice, the main ligand sender cells were inflammatory fibroblasts (cluster 2 and 4) and myofibroblasts (cluster 1 and 17) (Figure 4E″, Figure S2C). The ligand‐receptor pairs in Factor 6 mostly consisted of secreted signaling and ECM‐Receptor interactions (Figure S2B). The secreted ligand and cognate receptor pairs with the highest scores in Factor 6 included interactions between *Ccl7*‐*Ccr1*, *Cxcl5*‐*Cxcr2*, and *Ccl6*‐*Ccr1* (Figure 4E‴, Table S8), implicating hyperactive signaling between fibroblasts and neutrophils in wounds in the elderly. Collectively, these data suggest that subpopulations of fibroblasts in aged mice communicate with neutrophils through specific ligand‐receptor pairs, which is reduced in diabetic mice.

### 2.5 Reduced Collagen Formation Is Associated With Decreased ECM Gene Expression and Loss of *Lrrc15*+ Myofibroblasts in Wounded Aged Mice

**FIGURE 5:**
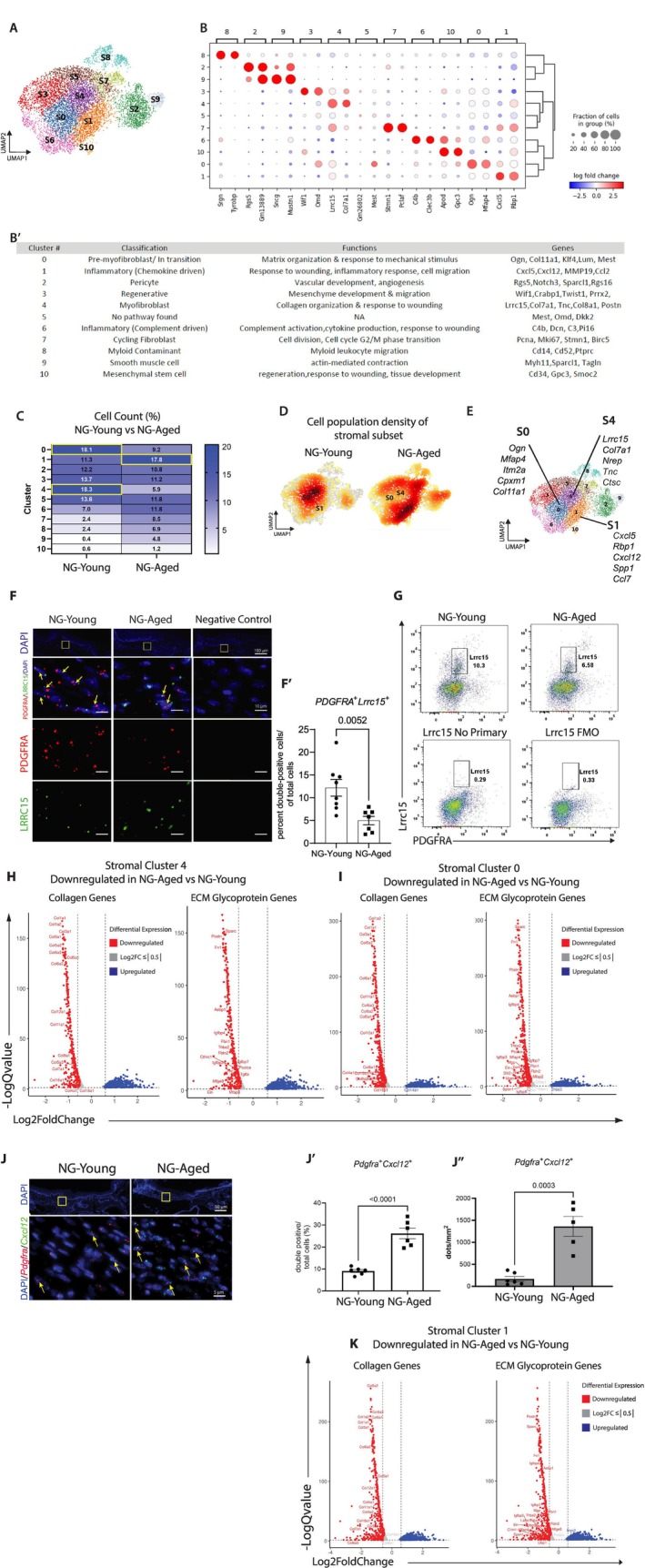
Reduction of extracellular matrix gene expression characterizes stromal component of the NG‐Aged wounds compared to NG‐Young group. Stromal cells from NG‐Young and NG‐Aged mucosal wounds in 7 day wounds were subclustered, differential gene expression analyzed, and *Pdgfra* ^+^ *Lrrc15* ^+^ transcripts quantified using in situ hybridization. (A) UMAP plots of stromal subset determined by unsupervised clustering in Scanpy (resolution 0.9). (B) Dotplot showing the top 2 marker genes expressed in each stromal subset using Scanpy clustering method. Data are expressed as log fold change. Size of the dot depicts the percent of cells expressing the gene. (B′) Table showing stromal cell type classification, pathways significantly enriched and the top 5 genes upregulated. (C) Heatmap showing percent of all stromal cluster cells across NG‐Young and NG‐Aged mice. (D) Density plot of stromal cells split up by NG‐Young and NG‐Aged groups. Density values are scaled. (E) UMAP plot depicting the top 5 marker genes for clusters S0, S1, and S4. (F) Representative RNAScope images of *Pdgfra* and *Lrrc15* transcripts in oral mucosal wounds (*n* = 7–8). (F′) Percent of *Pdgfra* ^+^ *Lrrc15* ^+^ cells per total cells. Data represent mean ± SEM, *p* values were calculated using unpaired two‐tailed Student's t‐test for pairwise comparison between NG‐Young group and NG‐Aged group. *p* values are shown numerically; statistical significance was defined as *p* < 0.05. (G) Flow cytometry plots for *Pdgfra* and *Lrrc15* expression in cells isolated from oral wounds. Representative of two independent experiments. Fluorescent minus one (FMO) and no primary antibody for Lrrc15 antibody was used as controls. (H) Volcano plot depicting differential expression of collagen genes and extracellular matrix glycoproteins genes in cluster S4, S0 (I), and S1 (K). All genes plotted were prefiltered for significant –LogQvalue. *p*‐values were calculated using a regression model and significant differences were obtained using the Wald‐test and adjusted by Benjamini‐Hochberg method (Qvalue) for pairwise comparison between NG‐Aged and NG‐Young groups. Significant Qvalue < 0.05. (J) RNAScope images of *Pdgfra* and *Cxcl12* transcripts in the mucosal wounds of NG‐Young and NG‐Aged mice (*n* = 6). (J′) Percent of *Pdgfra* ^+^ *Cxcl12* ^+^ double‐positive cells per total cells and by area (J″). Data represent mean ± SEM, *p* values were calculated using unpaired two‐tailed Student's *t*‐test for pairwise comparison between NG‐Young and NG‐Aged. *p* values are shown numerically; statistical significance was defined as *p* < 0.05.

To further explore the impact of aging, we analyzed the stromal compartment using Scanpy to compare stromal subpopulations in wounds from young and aged mice. This analysis identified 10 distinct stromal clusters (Figure 5A) based on their transcript signatures (Figure 5B, Table S7 Sheet “5. Scanpy‐stromal subset”). Figure 5B′ shows the functional classification of each stromal cluster. Worth noting is the clear identification of inflammatory and Lrrc‐positive stromal clusters. Stromal cluster‐0 (S‐0) is enriched in pro‐myofibroblast markers (*Ogn* and *Col11a1*), stromal cluster‐4 (S‐4) is enriched in myofibroblast markers (*Lrrc15*, *Col7a1*, and *Tnc*) and stromal cluster‐1 (S‐1) is enriched in inflammatory markers (*Cxcl5*, *Cxcl12* and *Ccl7*) (adj_*p*val < 0.001) (Figure 5E, Table S7 Sheet “5. Scanpy‐stromal subset”). Similarity of transcript signatures among Scanpy and Seurat analyses for stromal cells is summarized in Table S7 (Sheet “2. Seurat‐Scanpy‐Jaccard Index”). The cell proportions changed with aging, with a 50% decline in stromal cluster‐0 and a 67% decline in stromal cluster‐4, while stromal cluster‐1 exhibited a 57.5% increase compared to wounds from young mice (Figure 5C). Cell density analysis was undertaken to compare stromal cell populations in wounds from young and aged mice (Figure 5D). Clusters S‐0 and S‐4 were under‐represented in the aged wound fibroblasts, while cluster S‐1 was over‐represented in wounds from aged versus young mice (Figure 5D,E).

We next asked if the persistent granulation tissue observed in wounds from aged mice could be linked to differential expression of ECM‐related genes in the above stromal cell clusters. Our analysis revealed that collagen genes such as *Col1a1*, *Col1a2*, *Col3a1*, and *Col5a2* were all significantly downregulated in stromal clusters 0, 1, and 4 in wounds from the aged mice compared to young mice (adj_*p*val < 0.001) (Figure 5H,I,K‐left side‐). Moreover, ECM glycoprotein genes such as *Postn* and *Sparc*, *Fn1*, and *Aebp1*, important for collagen type I production and preserving matrix integrity (Ham et al. 2023), were all significantly downregulated in wounds from aged mice across stromal clusters 0, 1, and 4 compared to young counterparts (adj_*p*val < 0.001) (Figure 5H,I,K‐right side‐). To confirm the changes in myofibroblast and proinflammatory fibroblast numbers from the scRNA‐seq stromal clusters 1 and 4, validation experiments were carried out on wounds from aged and young mice sections, stromal clusters 1 and 4. RNAscope analysis confirmed a 2.4‐fold increase (*p* < 0.05) in *Pdgfra* ^+^ *Lrrc15* ^+^ myofibroblasts in the young group relative to the aged group (Figure 5F,F′). Flow cytometry further corroborated this finding (Figure 5G). Inflammatory fibroblasts showed both the percentage and normalized numbers of *Cxcl12* ^+^ *Pdgfra* ^+^ fibroblasts were increased in wounds from aged mice compared to young controls, agreeing well with the scRNA‐seq result. Collectively, our data demonstrate that wounds in the elderly have fewer matrix‐producing myofibroblasts compared to young groups and more inflammatory niches arising from fibroblast‐neutrophil communication, which may contribute to an impaired connective tissue turnover.

### 2.6 Diabetic Wounds Exhibit Gene Profiles Diverging From Optimal Healing Observed in NG‐Young Mice

**FIGURE 6:**
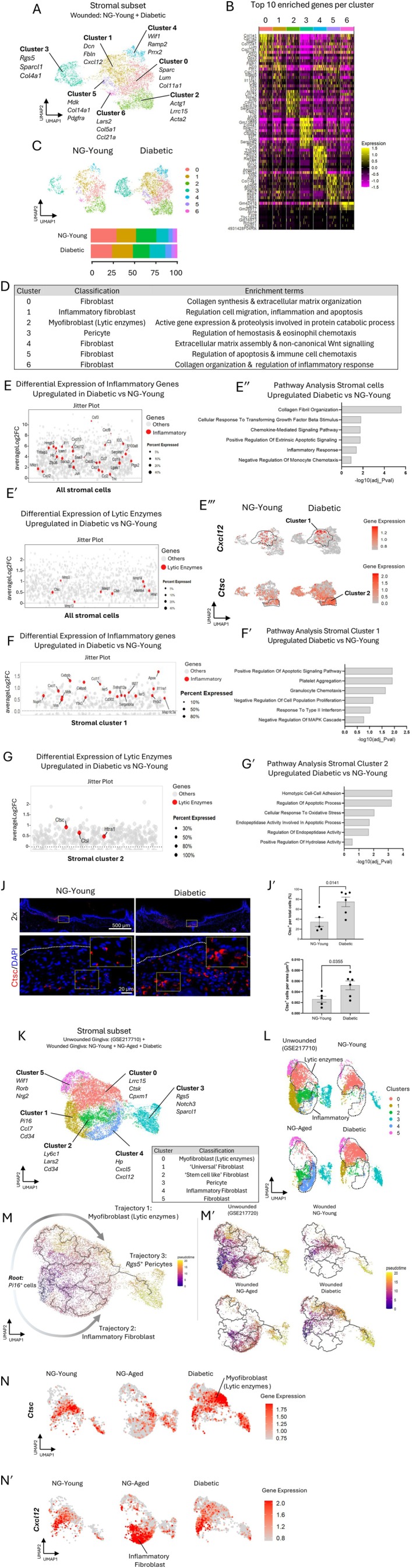
Comparative framework of gingival wounds from NG‐Young and diabetic mice in 7 day wounds. Stromal cells were subclustered, clusters classified, differentially expressed genes calculated, lytic enzymes‐related genes explored, and trajectory analysis performed. (A) UMAP plots of stromal subclusters from NG‐Young and diabetic mice showing the top 3 upregulated gene markers for each cluster. (B) Heatmap depicting the top 10 cluster‐specific gene sets. (C) UMAP plot of stromal subclusters split by condition (upper panel) and cell proportion bar graph of each cluster split by condition (lower panel). (D) Table showing stromal cluster classification and functional inference based on GO‐terms enrichment. (E) Jitterplot of inflammation‐associated genes and (E′) lytic enzymes genes differentially expressed in stromal diabetic group versus NG‐Young group. Significantly expressed genes (*p*adj value < 0.05) are highlighted in red. The size of the dot depicts the percent of cells expressing the gene. (E″) Bar graph of pathways significantly enriched in stromal cell in the diabetic group compared to the NG‐Young group. Data ranked by lowest −log10 (adj_*p*val). (E‴) UMAP depicting *Ctsc* and *Cxcl12* gene expression level in diabetic and NG‐Young wounded mice. Dotted lines circumscribe cluster 1 and 2 location, respectively. (F) Jitterplot of differentially expressed inflammation‐associated genes in stromal cluster 1 in diabetic group versus NG‐Young group. (F′) Bar graph of pathways significantly enriched in stromal cluster 1 in the diabetic group compared to the NG‐Young group. (G) Jitterplot of differentially expressed lytic enzyme genes in stromal cluster 2 in diabetic group versus NG‐Young group. (G′) Bar graph of pathways significantly enriched in stromal cluster 2 in the diabetic group compared to the NG‐Young group. (J) Immunofluorecent images of Ctsc protein in the gingival tissue of diabetic and NG‐Young mice (*n* = 5–6). White dotted line represents epithelial‐connective tissue junction. Yellow dotted box indicates a region of interest (ROI) in 2×, 40× and zoom ROI. (J′) Percent of Ctsc^+^ cells per total cell number in ROI and (J″) by area. Data represent mean ± SEM, *p* values were calculated using unpaired two‐tailed Student's *t*‐test for pairwise comparison between diabetic group and NG‐Young group. Statistical significance set at p < 0.05. (K) UMAP of stromal subclusters from unwounded gingiva (GSE217710), NG‐Young, diabetic and NG‐Aged mice showing the top 3 upregulated gene markers for each cluster. (L) UMAP split by condition highlighting inflammatory and lytic enzymes clusters (dotted areas). (M) UMAP showing the pseudotime trajectories (1, 2, 3) of the aggregated datasets from (K). Gray arrows represent main trajectories progress over pseudotime. (M') UMAP showing the pseudotime trajectories split by condition. (N) UMAP depicting *Ctsc* and (N′) *Cxcl12* gene expression level in the mucosal wounds of NG‐Young, diabetic and NG‐Aged mice.

Diabetic and NG‐Young stromal cells (fibroblast clusters 1, 2, 4, 5, 7, 12, 17, 22, and pericyte clusters 3, 21) in Figure 1E′ were subclustered and separated into 7 clusters (Figure 6A). The top 10 ranked genes for each cluster are shown in the heatmap in Figure 6B. Cluster 0 was the most abundant cluster with prototypic fibroblast characteristics exemplified by expressing collagens, *Sparc* and *Lum* genes. Inflammatory fibroblasts were identified in cluster 1 with elevated expression of chemokines *Cxcl12* and *Cxcl2*; proinflammatory cytokine *Il33* and complement genes from *C3* and *C4* families. Cluster 2 exhibited a myofibroblast phenotype with elevated expression of Lrrc family members, including *Lrrc15* and several matrix‐degrading enzymes such as *Ctsc* and *Ctsl*. Pericytes were mapped to cluster 3, expressing canonical gene markers *Rgs5* and *Sparcl1*. Fibroblasts in cluster 4 express high levels of *Wif1*, *Ramp2*, and *Prrx2* and are associated with high proliferative potential. Fibroblasts in cluster 5 had unique expression of *Col14a1* in combination with chemoattraction gene signatures such as *Cxcl13* and *Ccl8*, and matrix remodeling enzymes *Mmp13*, *Ctsb* and *Mmp3*. This fibroblast subset in gingiva has been designated as “fibroblasts activated to guide leukocyte migration” (Kondo et al. 2023). Cluster 6 showed high expression of collagen 5 and 6 family members linked with fibrillogenesis and *Cd74* genes related to inflammation regulatory functions. No major cell proportion differences were found in diabetic mice compared to NG‐Young counterparts, except for the increase in pericytes in the diabetic group (Figure 6C). A summary of each stromal cell function is provided in Figure 6D.

Next, we explored the expression of a set of inflammatory and matrix‐degrading genes across stromal cells in wounds from diabetic and matched NG‐Young mice. We observed an inflammatory signature enriched in *Cxcl12*, *Cxcl5*, *S100a8*, and *Il33* in wounds from diabetic mice in comparison to NG‐Young mice (Figure 6E). Lytic enzymes characterized wounds in diabetic mice with significant upregulation of *Ctsc*, *Ctsb*, *Mmp14*, and *Mmp13* compared to their NG‐Young counterparts (Figure 6E′). A full list of differentially expressed genes for all stromal cells and by stromal cell clusters is provided in Table S9. Gene ontology analysis identified collagen organization, chemotaxis, and inflammatory response as the main upregulated pathways in diabetic versus NG‐Young mice (Figure 6E″). We confirmed the upregulation of *Cxcl12* in cluster 2, whereas *Ctsc* was increased in cluster 1 in diabetic versus NG‐Young mice (Figure 6E‴). Thus, stromal cells from diabetic mice had increased expression of proteolytic enzymes and inflammatory mediators compared to similarly aged normoglycemic mice.

Cluster‐specific differential expression analysis of stromal cells confirmed the upregulation of pro‐inflammatory and proteolytic genes in wounds in diabetic versus matched normoglycemic mice. We identified significant upregulation of *Cxcl1*, *Ccl11* and *Tnfrsf12a* inflammatory genes in diabetic stromal cluster 1 compared to NG‐Young mice (Figure 6F). The upregulated genes in this cluster from diabetic mice were linked to apoptosis, granulocyte chemotaxis and cell proliferation pathways when compared to NG‐Young mice (Figure 6F′). Interestingly, we observed that diabetes induced upregulation of cathepsin genes and other lytic enzymes in stromal cluster 2 in comparison to NG‐Young mice (Figure 6G). These genes are involved in cysteine endopeptidase activity and apoptotic pathways in diabetic mice compared to its NG‐Young counterparts (Figure 6G′). We next detected Ctsc protein levels in gingival tissue by immunofluorescence (Figure 6J) and confirmed a 46% increase of Ctsc‐positive cells and a 38% increase in Ctsc^+^ cells per area in diabetic mice compared to NG‐Young mice (Figure 6J′). Overall, these results demonstrate that diabetes exerted transcriptional changes towards a more lytic‐enzymes signature than NG‐Young mice.

To determine whether fibroblasts enriched with lytic‐enzyme genes in diabetic mice and inflammatory fibroblasts observed in aged mice represent different cell states than those in NG‐Young mice, we integrated fibroblast scRNA‐seq dataset from unwounded mouse gingiva (GSE217710) (Ko et al. 2023) to set a common baseline for all wounded samples (Figure 6K). The baseline (root node) was set at Pi16^+^ “universal” fibroblasts based on Buechler et al. (Buechler et al. 2021). We identified cathepsin‐expressing cells in stromal cluster 0, *Pi16*‐expressing universal fibroblasts in cluster 1, and inflammatory fibroblasts in cluster 4 marked by *Cxcl12* and *Cxcl5* gene expression (Figure 6K,L). The trajectory analysis identified three main paths rooted in *Pi16* ^+^ universal fibroblast populations, including a progression towards a lytic‐enzymes cluster (Trajectory 1), an inflammatory fibroblast cluster (Trajectory 2) and a pericyte cluster (Trajectory 3) at later pseudotime (Figure 6M). Splitting different groups revealed that fibroblasts in diabetic mice acquire a trajectory towards those enriched in the expression of lytic enzymes, whereas aged mice had a different trajectory towards inflammatory an fibroblast cluster (Figure 6M′). We confirmed the upregulation of *Ctsc* gene expression in the diabetic group compared to NG‐Young or NG‐Aged mice (Figure 6N), while *Cxcl12* was robustly expressed by NG‐Aged mice compared to all other conditions (Figure 6N′). Together, these results suggest that lytic enzyme‐enriched fibroblasts from diabetic mice display a unique trajectory compared to the NG‐Young or NG‐Aged group, revealing distinct states of these cells between wounds from diabetic and aged mice. Moreover, inflammatory fibroblasts were expanded in NG‐Aged mice and exhibited a transcriptional state distinct from those in NG‐Young mice.

## 3 Discussion

**DIAGRAM 1:**
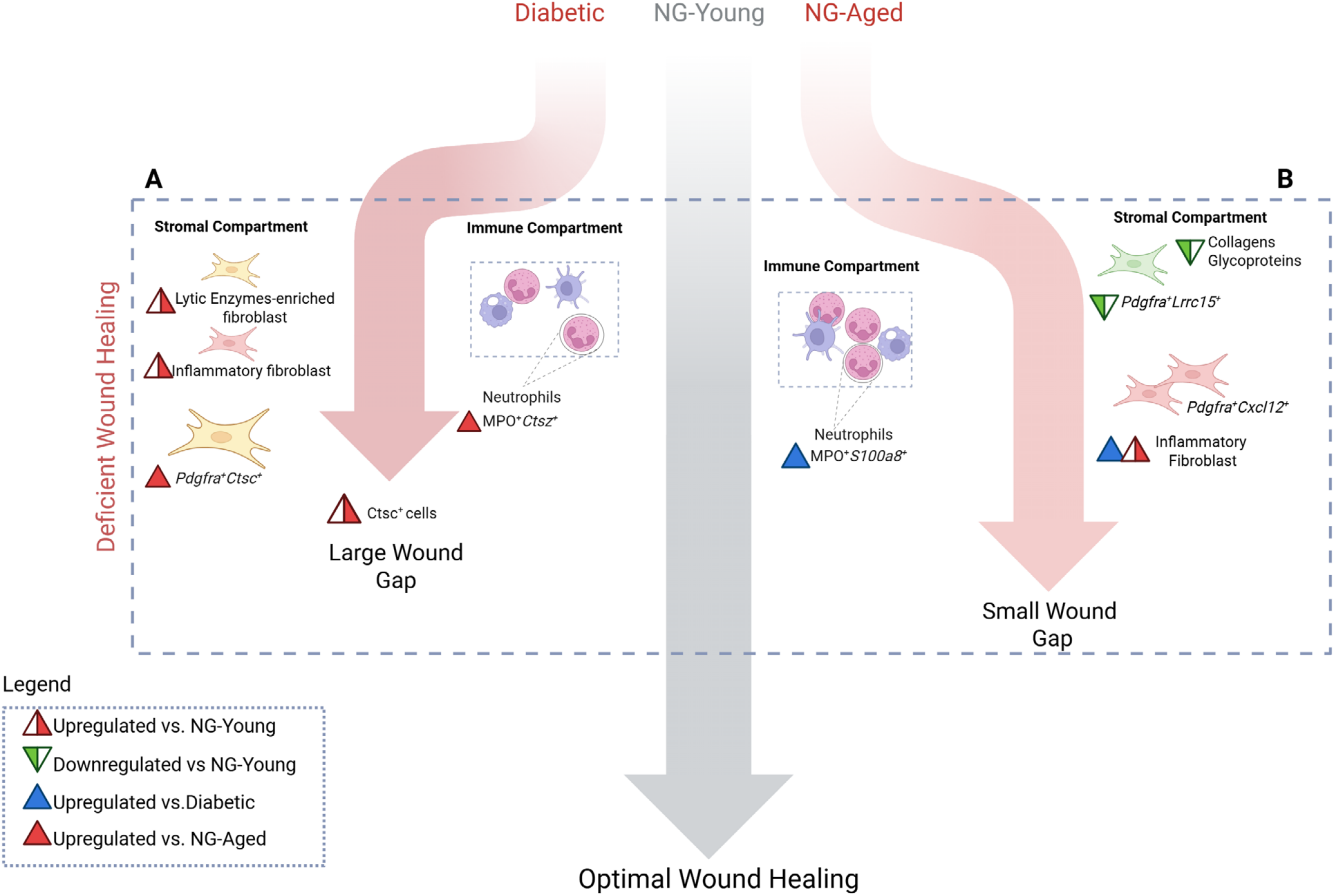
Schematic diagram depicting two distinct skews from optimal mucosal healing in diabetes and aging in 7 day wounds. At day 7, NG‐Young mice exhibit optimal wound healing characterized by wound gap closure, balanced expression of lytic enzymes, and low levels of inflammation. In contrast, diabetic mice show an increase in mononuclear phagocytes and express lytic enzymes, but in a distinct pattern, accompanied by a larger wound gap compared to NG‐Young mice. Notably, cathepsin expression is markedly upregulated in neutrophils and fibroblasts from diabetic wounds when compared to normoglycemic aged mice (A). In wounds from aged mice, mononuclear phagocytes are increased, inflammatory fibroblasts are decreased and *Lrrc15* ^+^ myofibroblasts are decreased when compared to wounds from young mice. Interestingly, lytic enzymes are downregulated in aged mice in comparison to their young counterpart, whereas neutrophils and fibroblasts from aged mice exhibit an increased inflammatory signature when compared to diabetic or NG‐Young mice. (B). Legend: Up‐pointing triangle with right half red: upregulated versus NG‐Young; down‐pointing triangle with left half green: downregulation versus NG‐Young; Blue up‐pointing triangle: upregulated versus diabetic; red up‐pointing triangle: upregulated versus NG‐Aged.

Defective wound healing is a hallmark of several conditions, including diabetes and aging (Ko et al. 2021; Thanapaul et al. 2021). In this study, we demonstrate that diabetes and aging result in distinct patterns of impaired mucosal wound healing, which we analyzed at histological, cellular, and molecular levels. We demonstrated that stromal cells in diabetic wounds diverge transcriptionally from the optimal healing profiles seen in normoglycemic young mice by upregulation of apoptotic processes, inflammation and lytic‐enzyme regulation. In addition, normoglycemic aged mice had an inflammatory fibroblast and neutrophil program characterized by upregulation of chemokines, cytokines, and S100a family genes, leading to increased neutrophil accumulation and prolonged inflammation (see Diagram 1). Diabetic wounds were impacted to a greater extent than matched wounds in normoglycemic aged mice as evidenced by substantially reduced epithelial closure. Mechanistically, this was linked to reduced granulation tissue formation, impaired collagen deposition, and increased expression of proteolytic enzymes. By Day 7, diabetic wounds remained open, while aged and young wounds had closed, although aged wounds retained persistent granulation tissue and reduced collagen content.

Diabetic mucosal wounds healed more slowly than wounds from normoglycemic aged or young mice. They exhibited reduced numbers of fibroblasts compared to aged mice as shown by scRNAseq and by histologic analysis, which is likely to contribute to the reduced production of granulation tissue and matrix. We previously reported that diabetes decreases fibroblast numbers through increased apoptosis and reduced proliferation, which may be mediated through increased activation of the transcription factor FOXO1 (Desta et al. 2010). The diabetic group was also characterized by an increase in matrix‐degrading enzymes in fibroblasts and neutrophils. Enhanced levels of cathepsins C, Z, D, K, B, and L were observed in fibroblasts and neutrophils of the diabetic group compared to aged mice, which has not been previously linked to impaired diabetic healing. Cathepsin C mRNA transcripts were upregulated in *Pdgfra* ^+^ fibroblasts at the wound edge of diabetic mice compared to normoglycemic aged mice. Cathepsin C is part of the cysteine family and degrades collagen, elastin, fibronectin, laminin and proteoglycans (Vidak et al. 2019). scRNAseq analysis found that Ctsc was significantly upregulated in myofibroblasts that expressed other lytic enzymes. This population is in a distinct state with upregulated pathways associated with apoptotic and proteolytic processes. Trajectory analysis confirmed that diabetic fibroblasts followed a distinct differentiation path toward lyctic enzyme‐rich states, whereas aged fibroblasts progressed toward inflammatory states.

Neutrophils in diabetic mice also had a more pronounced expression of proteolytic enzymes, with increases in cathepsins Z, D, and B compared to aged mice. A significant accumulation of *Ctsz* ^+^ neutrophils was identified at the wound edges of diabetic mice, a pattern not observed in aged mice. This overexpression of cathepsins in both fibroblasts and neutrophils likely contributes to excessive matrix degradation and delayed closure (Wilgus et al. 2013). While cathepsin Z lacks direct lytic activity, it plays a pivotal role in activating other ECM‐degrading cathepsins, modulating cell adhesion and migration, and degrading Cxcl12, a key chemokine for neutrophil trafficking (Xu et al. 2024; Mitrovic et al. 2022; Staudt et al. 2010). In contrast to neutrophils, we saw little differences in macrophage phenotypes, which is surprising given that they promote the healing process by undergoing a transition from M1 to M2 phenotypes (DiPietro et al. 2021).

Compared to young mice, healing was impaired in aged mice, which was reflected by delayed epithelial gap closure and persistent granulation tissue formation. scRNAseq analysis revealed that fibroblasts in aged mice had a shift towards a more inflammatory phenotype with higher transcript levels of *Cxcl5*, *Cxcl12*, *S100a13*, *Il6*, and *Serpine1,* amongst other inflammatory mediators. This was supported by a higher proportion of inflammatory *Pdgfra* ^+^ *Cxcl12* ^+^ fibroblasts in aged mice compared to diabetic. In addition, pseudotime analysis showed that inflammatory fibroblasts in wounds from aged mice are in a different cell state than diabetic or young mice. Myofibroblasts from wounds in aged mice exhibited a downregulation of *Col1a1*, *Col1a2*, and *Fn1* transcripts, that encode matrix proteins that are essential for connective tissue healing (Gimeno et al. 2022). Myofibroblasts are critical cells for producing connective tissue matrix (Hinz et al. 2001; Hinz et al. 2002; Zhang et al. 1994) and were reduced in wounds from aged compared to young mice shown by multiple approaches. The loss of myofibroblasts appears closely related to aging, as they were not diminished in diabetic wounds.

Neutrophils were increased in NG‐Aged mice compared to diabetic and NG‐Young mice at Day 7 post‐wounding, which reflects prolonged inflammation associated with impaired wound healing (Oliveira‐Costa et al. 2022). The expression of chemokines by fibroblasts may play a role in this recruitment, as has been shown for periodontitis (Kondo et al. 2023; Williams et al. 2021). In addition, prolonged inflammation may be due to neutrophils that exhibit upregulation of members of the S100a family, *S100a8*, and *S100a9*, which enhance neutrophil recruitment and are pro‐inflammatory (Hsu et al. 2009; Lackmann et al. 1992). Ligand‐receptor analysis revealed that fibroblast‐neutrophil signaling through chemokine pairs such as *Cxcl5‐Cxcr2* and *Ccl7‐Ccr1* was elevated in aged wounds, supporting an enhanced inflammatory communication network.

In conclusion, our study highlights distinct transcriptional profiles and cell states, suggesting mechanisms that drive impaired wound healing in diabetic mice and normoglycemic aged mice. Distinguishing features in wounds of diabetic mice included reduced matrix formation and greater expression of matrix degrading enzymes. Noteworthy was elevated levels of cathepsin family proteases, particularly cathepsin Z and C, which exacerbate tissue damage and delay healing. Aged Mice were characterized by an increase in neutrophils and higher expression of S100a family members. They also exhibited a marked reduction in collagen‐encoding gene expression and a loss of *Lrrc15*+ myofibroblasts, alongside increased inflammatory fibroblast populations that engage in heightened fibroblast‐neutrophil communication. Our study offers valuable insights into the unique differences in mucosal healing of diabetic and normoglycemic aged wounds. These findings may serve as a foundation for developing targeted interventions to improve wound healing outcomes.

## 4 Methods

### 4.1 Experimental Model and Mice Details

#### 4.1.1 Animals, Induction of Diabetes and Aging

All animal experiments were approved by the University of Pennsylvania Institutional Animal Care and Use Committee (Protocol #804855). Wild‐type mice were Col1a2Cre^−/−^; Foxo^L/L^ mice, which are on a mixed C57B6‐FVB background. Mice were divided into three groups: normoglycemic 3–6 months (NG‐Young), diabetic young and normoglycemic 16–22 months (NG‐Aged). There were a balanced number of female‐male ratios per experiment. Diabetes type 1 was induced using 50 mg/Kg streptozotocin (Sigma Aldrich, #S0130) in 10 mM citrate buffer (pH 7). Daily intraperitoneal injections were performed for 5 days on young mice. Control mice were treated identically with 10 mM citrate buffer. Blood glucose was monitored and all mice were hyperglycemic for at least 3 weeks (> 220 mg/dL) before performing oral surgery.

#### 4.1.2 Mucosal Wound Creation

Mice were anesthetized with 2%–3% isoflurane (Covetrus, #029404) using a nasal mask, and 3.25 mg/kg Buprenorphine extended‐release (Ethiqa XR, Fidelis, #267.10000.3) was used to control post‐procedural pain in mice following the manufacturer's recommendations. A mucoperiosteal wound was created in the palate between the 1st and 2nd molar using a 1 mm biopsy punch (Integra Miltex, #95039‐090). After wounding, mice were fed with DietGel 76A (ClearH2O, #72‐07‐5022) and housed in alpha‐dri bedding until euthanasia on Day 4 or 7 post‐wounding.

#### 4.1.3 Histology and Morphometric Analysis (Day‐4 and ‐7 Post Wounding)

Samples were fixed in 10% formalin at 4°C overnight, washed, and decalcified in 14% EDTA (pH 7.4) for 2–3 weeks. Next, decalcified tissues were embedded in paraffin, and 5 μm thickness paraffin sections were prepared for H&E or Masson's Trichrome staining (Newcomer Supply company, #9176B) following the manufacturer's recommendations. The epithelial gap, granulation tissue formation, and collagen area were measured using Nikon NIS‐elements image analysis software (Nikon) as previously described (Zhang et al. 2017).

#### 4.1.4 Immunofluorescence (Day‐7 Post Wounding)

Paraffin sections were deparaffinized, rehydrated, and epitopes unmasked using 1× saline sodium citrate buffer (pH 6) or Tris‐EDTA (pH 9) at 120°C under pressure for 20 min. Samples were permeabilized with 1× PBS + 0.1% Triton X‐100 for 10 min and blocked in a solution containing 0.01% Tween‐20,0.1% BSA, and 1% serum from host species matching that of secondary antibody serum in 1× PBS. Slides were incubated with the MPO (1 μg/mL dilution, Bio‐Techne, #AF3667) and S100a8 (6.25 ng/mL, Cell Signaling, #47310) or Ctsc (2 μg/mL, R&D Systems, #AF1034‐SP) antibodies at 4°C overnight. Next, slides were washed, endogenous peroxidase activity blocked with 3% H_2_O_2_ and incubated with Alexa Fluor 488 and Alexa Fluor 647 at room temperature for 45 min. Slides were mounted with mounting medium with 4′,6‐diamidino‐2‐phenylindole (DAPI). Images were captured at 40× magnification with a fluorescence microscope (Eclipse 90i; Nikon) and analyzed using Artificial Intelligence‐trained cell detection in Nikon GA3 software tool or Qupath (Bankhead et al. 2017).

#### 4.1.5 RNA In Situ Hybridization and Dual RNA‐Protein Detection (Day‐7 Post Wounding)

RNAscope Multiplex Fluorescent Detection Kit v2 was purchased from ACD, and reagents were used as recommended by the manufacturer. Deparaffinized samples were treated with hydrogen peroxide at RT for 10 min, and antigen retrieved with Protease Plus at 40°C for 15 min. The study used the RNA probes purchased from ACD as follows: *Cxcr2* (#461531‐C2), *Pdgfra* (#480661‐C3), *Cxcl12* (#422711), *Lrrc15* (#467831‐C2), *Ctsc* (#1195511‐C1), and *Ctsz* (#556911). Hybridization solution or negative control probe was incubated at 40°C for 2 h. TSA Vivid fluorophore 570 (1:1000‐1:1500 dilution, #323272) or TSA Vivid fluorophore 650 (1:1000–1:1500 dilution, #323273) was used. DAPI was added to each sample and mounted (abcam, #ab104135). We performed RNAscope Multiplex Fluorescent v2 for *Ctsz* with sequential protein detection of MPO following ACD protocol #323100‐TN. Imaging acquisition was made using 40× magnification Keyence BZ‐X800 Microscope or fluorescence microscope (Eclipse 90i; Nikon). RNA signal analysis was performed with Nikon GA3 software tool.

#### 4.1.6 Flow Cytometry (Day‐7 Post Wounding)

Palatal gingiva was obtained using a 2 mm biopsy punch (Integra Miltex, #12‐460‐399) over the wounded area. For flow cytometry and single cell sequencing experiments, six to eight wounds were combined for enzymatic digestion. Tissues were minced and digested at 37°C with constant agitation in DMEM media containing DNAse I (0.15 mg/mL; Roche), collagenase type IV (3.2 mg/mL; Gibco), and dispase (2.6 mg/mL; Sigma‐Aldrich) for 1 h. Fc receptors were blocked (Biolegend, #101319), and viability dye (Zombie Yellow; Biolegend, #423103). We followed the extracellular staining protocol as previously described (Ko et al. 2023). For lineage‐negative selection, cells were stained with CD31 (Biolegend, #102509), Ter119 (Biolegend, #116211), and Epcam (Biolegend, #141720) antibodies, and positive events were excluded. CD45 (Biolegend, #147709) CD140A (Biolegend, #135923) were used to stain and identify fibroblasts and the expression of Lrrc15 (Donation from Dr. Shannon Turley, Genentech). Data were acquired using a BD LSR II Flow Cytometer and analyzed using FlowJo software. Isotype IgG or fluorescence‐minus‐one controls were used to determine the gating strategy.

#### 4.1.7 scRNA‐Seq and Computational Analysis (Day‐7 Post Wounding)

Stromal and immune cells were sorted on the FACSAria II instrument. An enrichment of the stromal compartment was made by mixing 60% of Live CD31^−^, Ter119^−^, Epcam^−^ CD45^−^, and 40% of Live CD31^−^, Ter119^−^, Epcam^−^ CD45^+^ cells for each condition. 10,000 cells per condition were sequenced on an Illumina NovaSeq 6000 instrument at the Penn Genomic and Sequencing Core. CellRanger (v6.0.2) generates FASTQ files and aligns sequencing reads to the mouse reference genome (mm10). Seurat and Scanpy tools were used for quality control downstream analysis. Data was filtered using the following parameters: ambient mRNA contamination was removed using Soupx (v1.3.0), nCount_RNA ≥ 500 & nFeature_RNA > 200 & nFeature_RNA < 6500 & percent.mt < 15. Briefly, gene expression from filtered cells of all three conditions was normalized, scaled, integrated, and visualized via low‐dimensional uniform manifold approximation and projection (UMAP) to identify cell clusters. Differentially expressed genes (DEGs) were identified using Seurat (Butler et al. 2018). DEGs in addition to EnrichR (Panglao database) (Franzen et al. 2019) were used to annotate cell populations. DEGs were generated using “FindMarkers” function and a MAST test with a *p*‐adjusted value cut‐off < 0.05 and log2FC > or < 0.5. Cluster‐based functional changes were tested using clusterProfiler or EnrichR (Gene Ontology (GO) and Reactome databases) (Wu et al. 2021) and with a Benjamini–Hochberg (BH) multiple testing adjustment and a false‐discovery rate (FDR) cutoff of 0.1. For comparative analysis with publicly available datasets, unwounded gingiva was used (GSE217710) (Ko et al. 2023). We run “ScTransform” separately for each sample followed by sample integration and “PrepSCTFindMarkers” to harmonize the sequencing depth and control batch effect before running downstream analysis. Pseudotime analysis was performed using the Monocle3 pipeline (Trapnell et al. 2014). It should be noted that we were limited in the analysis of neutrophils due to the small cell number obtained in the NG‐Young group. Similar limitations have been noted by others (Williams et al. 2021; Hoogendijk et al. 2019). For visualization, UMAP plot, heatmaps, volcano, density, and jitter plots were used. Cell proportions, cell–cell interactions, and clusters gene overlap from the scRNA‐seq analysis were graphed using Datawrapper and the eVenn web tool (Yang et al. 2024).

#### 4.1.8 Differential Expression of Cell–Cell Communication Ligand‐Receptor Pairs Between Fibroblasts and Neutrophils in NG‐Aged Versus Diabetic Wounded Mice at Day‐7 Post Wounding

Gene expression was used to compute the potential of cell–cell communication for a list of ligand‐receptor pairs across all combinations of sender‐receiver pairs of cell types. We used a pipeline combining LIANA and Tensor‐cell2cell to study cell–cell communication across conditions (Baghdassarian et al. 2024). The input communication scores were computed as the geometric mean of the expression of the ligand in a sender cell type and the receptor in a receiver cell type, using the ligand‐receptor interactions of mice available on CellChat (Jin et al. 2021).

### 4.2 Statistical Methods

General: Statistical analysis was performed in GraphPad Prism software, and results are shown as mean ± SEM. Normality of the data was checked utilizing the Shapiro–Wilk test. *p* values were calculated using unpaired two‐tailed Student's *t*‐test for pairwise comparison between Diabetic and NG‐Aged or ANOVA for all condition comparisons. At least six biological replicates were performed for validating the scRNAseq results for each condition. Significant mean difference values were set at *p* < 0.05. Statistical significance for transcriptome differential expression (DE) analysis was set at < 0.05 for adjusted *p*‐values or stated otherwise in the figure's legend.

## Author Contributions

Dana T. Graves conceptualized the study. Dana T. Graves, Leticia Rojas Cortez, and Kang I. Ko designed the experiments. Leticia Rojas Cortez, Zhe Lyu, Hamideh Afzali, Min Liu, and Jane Yang performed experiments. Computational analysis and resources were performed by Leticia Rojas Cortez, Adolfo Rojas, Erick Armingol, Michael Troka, Michael V. Gonzalez, and Vinicius Maracaja‐Coutinho. The original draft was written by Dana T. Graves and Leticia Rojas Cortez and edited by Dana T. Graves, Kang I. Ko, Leticia Rojas Cortez, and Patricio Smith. All authors reviewed and approved the final version of the manuscript for submission.

## Conflicts of Interest

The authors declare no conflicts of interest.

## Supporting information


**Figure S1:** Masson's trichrome staining of the granulation tissue and quality control of single cell sequencing of gingival wounds from NG‐Young, diabetic, and NG‐Aged mice. (A) Representative images of Masson Trichrome staining in 4 and 7 day wounds from diabetic, NG‐Aged and NG‐Young mice. Scale bar = 100 μm. (B) Bar graph depicting scRNA‐seq data quality parameters for each condition, including number of genes (nFeature_RNA), number of UMIs (nCount_RNA) and percentage of mitochondrial (percent. mt) genes. Red dashed lines represent the threshold applied to nCount_RNA ≥ 500, nFeature_RNA > 200 and < 6500, and percent. mt < 15. (C) Dot plot depicting the top 10 cluster gene markers in the integrated data. Legend indicates average expression (purple:low; red:high) and dot size represents percent expression.

**Figure S2:** Cell–cell communication pre‐processing steps for stromal cell communication with all other cell types in wounds from diabetic and NG‐Aged mice at 7 days. (A) Elbow plot indicating normalized error curve, which reflects the supervised machine learning efficiency, that guides the selection of six relevant factors. (B) Distribution of ligand receptor pairs for Factor 6 among the three major cell2cell ligand‐receptor pair components. Color code: gray: cell–cell contact; yellow: ECM‐Receptor and purple: secreted signaling. (C) Barplot of sender cells in Factor 6. (D) Barplot of receiver cells in Factor 6. Data expressed as the mean of the communication score. The arrow points to the highest value in both bar plots.

**Table S1:** Cluster classification of the commonly identified immune and stromal cell clusters in NG‐Young, diabetic, and NG‐Aged mice in 7 day wound. Differentially expressed genes by clusters and EnrichR cell annotation of wounded oral mucosa. Differentially expressed genes defining fibroblasts, pericytes, neutrophils, macrophages, dendritic cells, T‐ and B cells present in diabetic, NG‐Aged and NG‐Young mice. Values are expressed as the average of logarithmic fold change (avg_log2FC). The genes which have *p*‐adjusted value (*p*_val_adj) < 0.05 are considered as differentially expressed. Percent of cells expressing the gene in the cluster is represented by ‘pct.1’ and in all other cluster by “pct.2.”

**Table S2:** Stromal and immune cells from NG‐Young, diabetic and NG‐Aged wounded gingiva at 7 days were sorted, sequenced, cell type inferred and clusters labeled. Table presents cell type inference output from EnrichR gene set enrichment method for each cluster. It provides the overlap between our gene list and their curated database and cell type is ranked by odds ratio or combined score with a respective adjusted *p*‐value. The cell assignation was made based on higher combined score with *p*_val_adj < 0.05 and manually curated.

**Table S3:** Neutrophils from Diabetic and NG‐Aged wounded gingiva at 7 days were subclustered, differential gene expression calculated, and pathway analysis performed. Table presents pathway analysis of neutrophils based on the up‐ and down‐regulated genes found in wounds from diabetic mice compared to the NG‐Aged group. The results of GO and Reactome database are displayed. The “ID” column indicates the database used, the “description” column shows the pathway, the count indicates the common genes between our data set and the pathway name, while GeneRatio column indicates the total genes of our data set out of all the genes involved in the pathway. The result of the statistical test for over‐representation are in the adjusted *p* value column, significance was set at with *p* adjust < 0.05 and the data was sorted by the −log10 (*p*adj) column.

**Table S4:** Differential gene expression of stromal cell subclusters in wounded gingiva from diabetic and NG‐Aged mice in 7 day wounds. Table presents the differential expression genes for each stromal subclusters. Values are expressed as average of logarithmic fold‐change (avg_log2FC). The genes which have *p*‐adjusted value (*p*_val_adj) < 0.05 are considered as differentially expressed. Percent of cells expressing the gene in the cluster is represented by “pct.1” and in all other cluster by “pct.2.” The “cluster” column indicates the stromal subcluster ID.

**Table S5:** Pathway analysis was inferred based on differential gene expression analysis on stromal cell subclusters from diabetic wounded gingiva and NG‐Aged mice in 7 day wounds. Table summarizing the pathway analysis for each stromal cluster to classify their main functions. The results are expressed as combined score for GO and Reactome databases are displayed for each cluster. This selection of pathways considered the top 3 results ranked by combined score in each database that had a *p*_val_adj < 0.1, with three or more genes involved in it and with minimal repeated terms.

**Table S6:** Differential gene expression of all stromal cell in wounded gingiva from diabetic wounded gingiva and NG‐Aged mice in 7 day wounds. Differential expression gene in stromal cells from diabetic versus NG‐Aged mice. Each comparison run is noted in column “comparison.” DEG was analyzed using MAST method. Values are expressed as average of logarithmic fold‐change (avg_log2FC). The genes which have *p*‐adjusted value (*p*_val_adj) < 0.05 are considered as differentially expressed. Percent of cells expressing the gene in the cluster is represented by “pct.1” and in all other cluster by “pct.2.”

**Table S7:** Comparison of Seurat and Scanpy clustering similarity for stromal cells in Diabetic and NG‐Aged groups at 7 days. Cluster similarity analysis. (Sheet 1) Cluster signature genes compared among Cluster 4 (Figure 1C) and Cluster 4 (Figure 3A) or Cluster 1 (Figure 1C) and Cluster 1 (Figure 3A) or Cluster 12 (Figure 1C) and Cluster 1 (Figure 3A), indicating the total number of common genes and the corresponding Jaccard index. (Sheet 2) Signature genes similarity for stromal cells compared among Seurat and Scanpy clustering method. Metrics show the total number of common genes and the Jaccard index. (Sheet 3) Top 50 signature genes of stromal cells present in Figure 1C. (Sheet 4) Top 50 signature genes of stromal subcluster using Seurat method. (Sheet 5) Top 50 signature genes of stromal subcluster using Scanpy method.

**Table S8:** Communication scores for NG‐Aged gingival wounds versus diabetic wounds in 7 day wounds. Ligand receptor pairs for Factor 5 and 6. Data indicate the category of the ligand‐receptor pair (cell–cell contact, secreted signaling or ECM‐receptor) and the communication score values are represented as loadings values (color coded: green‐low values; red‐high values).

**Table S9:** Differential gene expression of all stromal cell and by subclusters diabetic and NG‐Young mice in 7 day wounds. Differential expression genes in all stromal cells (“DEG_All_StromalCells_DBvsNG”) and by stromal cell clusters (“DEG_by_Cluster_DBvsNG”) from diabetic versus NG‐Young groups. Differential gene expression was analyzed using MAST method. Values are expressed as average of logarithmic fold‐change (avg_log2FC). The genes which have *p*‐adjusted value (*p*_val_adj) < 0.05 are considered as differentially expressed. Percentage of cells expressing the gene in the cluster is represented by “pct.1” and in all other cluster by “pct.2.”

## Data Availability

The data that support the findings of this study are openly available in Gene Expression Omnibus at https://www.ncbi.nlm.nih.gov/gds, reference number GSE271589.
