# Supplementary figures and images for: Single Cell Sequencing Identifies Distinct Cellular Alterations in Impaired Aged and Diabetic Wounds
Source: Aging Cell. 2025 Nov 4;24(12):e70217. doi: 10.1111/acel.70217 (PMC12686554; doi:10.1111/acel.70217)

Supplementary Figure 2

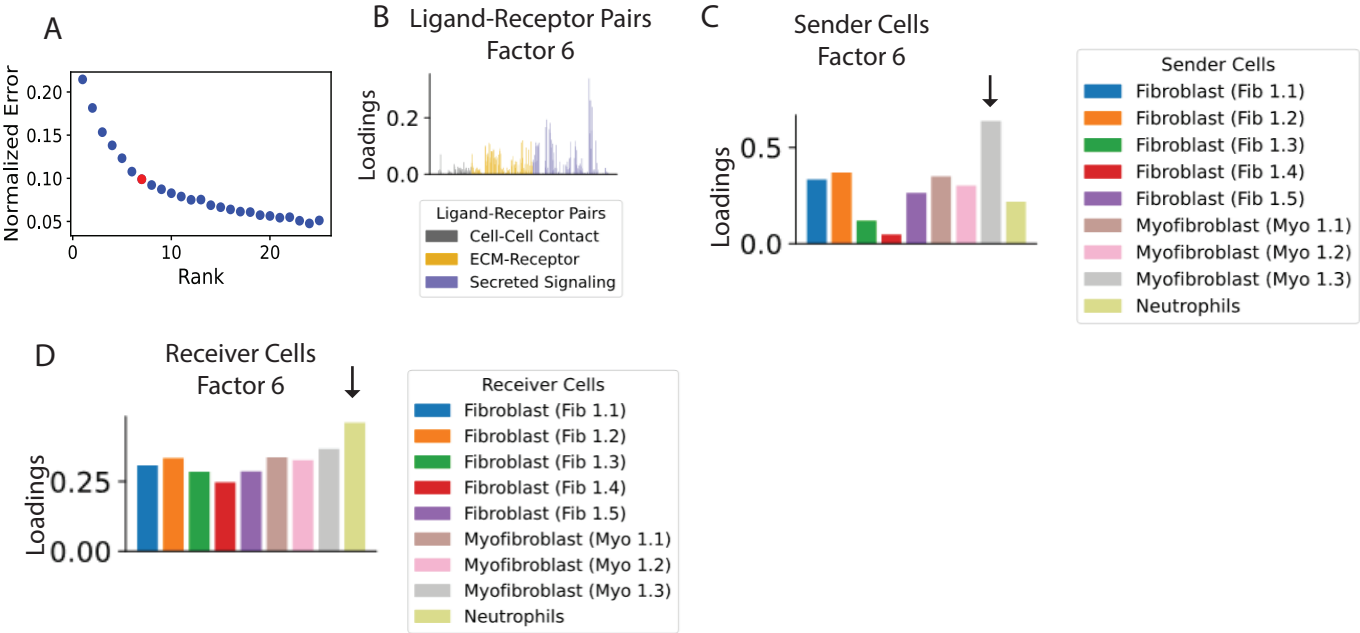

Supplement: Supplementary file 2 — Figure S2: Cell–cell communication pre‐processing steps for stromal cell communication with all other cell types in wounds from diabetic and NG‐Aged mice at 7 days. (A) Elbow plot indicating normalized error curve, which reflects the supervised machine learning efficiency, that guides the selection of six relevant factors. (B) Distribution of ligand receptor pairs for Factor 6 among the three major cell2cell ligand‐receptor pair components. Color code: gray: cell–cell contact; yellow: ECM‐Receptor and purple: secreted signaling. (C) Barplot of sender cells in Factor 6. (D) Barplot of receiver cells in Factor 6. Data expressed as the mean of the communication score. The arrow points to the highest value in both bar plots. [file ACEL-24-e70217-s009.pdf]
