# Supplementary material for: Single Cell Sequencing Identifies Distinct Cellular Alterations in Impaired Aged and Diabetic Wounds
Source: Aging Cell. 2025 Nov 4;24(12):e70217. doi: 10.1111/acel.70217 (PMC12686554; doi:10.1111/acel.70217)
Supplement: Supplementary file 3 — Table S1: Cluster classification of the commonly identified immune and stromal cell clusters in NG‐Young, diabetic, and NG‐Aged mice in 7 day wound. Differentially expressed genes by clusters and EnrichR cell annotation of wounded oral mucosa. Differentially expressed genes defining fibroblasts, pericytes, neutrophils, macrophages, dendritic cells, T‐ and B cells present in diabetic, NG‐Aged and NG‐Young mice. Values are expressed as the average of logarithmic fold change (avg_log2FC). The genes which have p‐adjusted value (p_val_adj) < 0.05 are considered as differentially expressed. Percent of cells expressing the gene in the cluster is represented by ‘pct.1’ and in all other cluster by “pct.2.” [file ACEL-24-e70217-s010.docx]

Table S1. Cluster classification of the commonly identified immune and stromal cell clusters in NG-Young, diabetic, and NG-Aged mice at day 7 post gingival wounding. Differentially expressed genes by clusters and EnrichR cell annotation of wounded oral mucosa. Differentially expressed genes defining fibroblasts, pericytes, neutrophils, macrophages, dendritic cells, T- and B cells present in diabetic, NG-Aged and NG-Young mice. Values are expressed as average of logarithmic fold change (avg_log2FC). The genes which have p-adjusted value (*p*_val_adj) < 0.05 are considered as differentially expressed. Percentage of cells expressing the gene in the cluster is represented by ‘pct.1’ and in all other cluster by ‘pct.2’.

| avg_log2FC | pct.1 | pct.2 | p_val_adj | cluster number | gene | Annotation_EnrichR |
| --- | --- | --- | --- | --- | --- | --- |
| 6.002353 | 0.946 | 0.028 | 0 | 0 | S100a9 | Neutrophils |
| 3.677229 | 0.819 | 0.025 | 0 | 0 | G0s2 | Neutrophils |
| 3.563748 | 0.994 | 0.316 | 0 | 0 | Srgn | Neutrophils |
| 3.309569 | 0.979 | 0.167 | 0 | 0 | Il1b | Neutrophils |
| 3.158773 | 0.978 | 0.211 | 0 | 0 | Cd14 | Neutrophils |
| 2.809817 | 0.895 | 0.116 | 0 | 0 | Ccrl2 | Neutrophils |
| 2.740107 | 0.954 | 0.344 | 0 | 0 | Cxcl2 | Neutrophils |
| 2.412493 | 0.98 | 0.251 | 0 | 0 | Tyrobp | Neutrophils |
| 2.398523 | 0.928 | 0.131 | 0 | 0 | Clec4e | Neutrophils |
| 2.366128 | 0.905 | 0.022 | 0 | 0 | Hdc | Neutrophils |
| 2.363405 | 0.998 | 0.944 | 0 | 0 | Fth1 | Neutrophils |
| 2.310569 | 0.973 | 0.646 | 0 | 0 | Nfkbia | Neutrophils |
| 2.230957 | 0.92 | 0.094 | 0 | 0 | Mxd1 | Neutrophils |
| 2.208687 | 0.916 | 0.194 | 0 | 0 | Marcksl1 | Neutrophils |
| 2.157002 | 0.93 | 0.131 | 0 | 0 | Clec4d | Neutrophils |
| 2.150299 | 0.745 | 0.147 | 0 | 0 | Slpi | Neutrophils |
| 2.108985 | 0.896 | 0.087 | 0 | 0 | Slc7a11 | Neutrophils |
| 2.104751 | 0.977 | 0.532 | 0 | 0 | Cebpb | Neutrophils |
| 2.040712 | 0.961 | 0.701 | 0 | 0 | S100a11 | Neutrophils |
| 1.972948 | 0.859 | 0.366 | 0 | 0 | Gadd45b | Neutrophils |
| 1.939373 | 0.737 | 0.025 | 0 | 0 | Acod1 | Neutrophils |
| 1.933318 | 0.886 | 0.159 | 0 | 0 | Plek | Neutrophils |
| 1.926605 | 0.856 | 0.218 | 0 | 0 | Tnfaip2 | Neutrophils |
| 1.868613 | 0.321 | 0.005 | 0 | 0 | Cstdc4 | Neutrophils |
| 1.848831 | 0.853 | 0.071 | 0 | 0 | Samsn1 | Neutrophils |
| 1.817627 | 0.367 | 0.003 | 0 | 0 | Retnlg | Neutrophils |
| 1.789496 | 0.786 | 0.034 | 0 | 0 | Il1r2 | Neutrophils |
| 1.767757 | 0.337 | 0.055 | 0 | 0 | Ccl3 | Neutrophils |
| 1.752684 | 0.775 | 0.083 | 0 | 0 | Nlrp3 | Neutrophils |
| 1.74533 | 0.952 | 0.242 | 0 | 0 | Fcer1g | Neutrophils |
| 1.716923 | 0.827 | 0.107 | 0 | 0 | Msrb1 | Neutrophils |
| 1.714189 | 0.95 | 0.414 | 0 | 0 | Mcl1 | Neutrophils |
| 1.692655 | 0.958 | 0.707 | 0 | 0 | Ifitm2 | Neutrophils |
| 1.663262 | 0.399 | 0.005 | 0 | 0 | Stfa2l1 | Neutrophils |
| 1.654518 | 0.859 | 0.305 | 0 | 0 | Btg2 | Neutrophils |
| 1.646118 | 0.5 | 0.106 | 0 | 0 | Wfdc17 | Neutrophils |
| 1.646096 | 0.925 | 0.325 | 0 | 0 | Pim1 | Neutrophils |
| 1.643862 | 0.9 | 0.236 | 0 | 0 | Cd52 | Neutrophils |
| 1.612351 | 0.842 | 0.313 | 0 | 0 | Nfkbiz | Neutrophils |
| 1.611048 | 0.781 | 0.024 | 0 | 0 | Trem1 | Neutrophils |
| 1.598107 | 0.785 | 0.126 | 0 | 0 | Il1rn | Neutrophils |
| 1.585047 | 0.628 | 0.124 | 0 | 0 | Ifitm1 | Neutrophils |
| 1.529437 | 0.422 | 0.007 | 0 | 0 | Lcn2 | Neutrophils |
| 1.515411 | 0.814 | 0.066 | 0 | 0 | Lmnb1 | Neutrophils |
| 1.486619 | 0.624 | 0.01 | 0 | 0 | Lrg1 | Neutrophils |
| 1.486613 | 0.825 | 0.102 | 0 | 0 | Tpd52 | Neutrophils |
| 1.479538 | 0.805 | 0.069 | 0 | 0 | Grina | Neutrophils |
| 1.4739 | 0.854 | 0.412 | 0 | 0 | Dusp1 | Neutrophils |
| 1.438927 | 0.771 | 0.011 | 0 | 0 | Csf3r | Neutrophils |
| 1.437003 | 0.766 | 0.121 | 0 | 0 | Lst1 | Neutrophils |
| 1.436823 | 0.887 | 0.401 | 0 | 0 | Marcks | Neutrophils |
| 1.431539 | 0.856 | 0.211 | 0 | 0 | Lcp1 | Neutrophils |
| 1.396773 | 0.885 | 0.336 | 0 | 0 | Fxyd5 | Neutrophils |
| 1.376885 | 0.707 | 0.049 | 0 | 0 | Slc16a3 | Neutrophils |
| 1.360586 | 0.761 | 0.18 | 0 | 0 | Cox17 | Neutrophils |
| 1.349145 | 0.666 | 0.061 | 0 | 0 | Hp | Neutrophils |
| 1.326188 | 0.623 | 0.107 | 0 | 0 | Bcl2a1b | Neutrophils |
| 1.321557 | 0.783 | 0.097 | 0 | 0 | Ets2 | Neutrophils |
| 1.317524 | 0.713 | 0.036 | 0 | 0 | Entpd1 | Neutrophils |
| 1.306277 | 0.755 | 0.102 | 0 | 0 | Ptafr | Neutrophils |
| 1.296437 | 0.667 | 0.005 | 0 | 0 | Cxcr2 | Neutrophils |
| 1.287378 | 0.777 | 0.156 | 0 | 0 | Ptprc | Neutrophils |
| 1.283222 | 0.952 | 0.558 | 0 | 0 | Btg1 | Neutrophils |
| 1.246563 | 0.744 | 0.086 | 0 | 0 | Ccr1 | Neutrophils |
| 1.223958 | 0.777 | 0.148 | 0 | 0 | Gmfg | Neutrophils |
| 1.215845 | 0.99 | 0.921 | 0 | 0 | Eif1 | Neutrophils |
| 1.151642 | 0.716 | 0.177 | 0 | 0 | Taldo1 | Neutrophils |
| 1.140484 | 0.793 | 0.187 | 0 | 0 | Litaf | Neutrophils |
| 1.129215 | 0.746 | 0.2 | 0 | 0 | Slfn2 | Neutrophils |
| 1.125382 | 0.751 | 0.319 | 0 | 0 | Prdx5 | Neutrophils |
| 1.099143 | 0.509 | 0.005 | 0 | 0 | Hcar2 | Neutrophils |
| 1.092943 | 0.525 | 0.104 | 0 | 0 | Cdk2ap2 | Neutrophils |
| 1.084484 | 0.937 | 0.687 | 0 | 0 | Junb | Neutrophils |
| 1.075855 | 0.706 | 0.215 | 0 | 0 | Tnfaip3 | Neutrophils |
| 1.075123 | 0.758 | 0.157 | 0 | 0 | Cd53 | Neutrophils |
| 1.064086 | 0.737 | 0.101 | 0 | 0 | C5ar1 | Neutrophils |
| 1.061269 | 0.617 | 0.121 | 0 | 0 | Kctd12 | Neutrophils |
| 1.052927 | 0.818 | 0.322 | 0 | 0 | Cd44 | Neutrophils |
| 1.047633 | 0.775 | 0.321 | 0 | 0 | Pnrc1 | Neutrophils |
| 1.044738 | 0.6 | 0.024 | 0 | 0 | Cyp4f18 | Neutrophils |
| 1.041633 | 0.952 | 0.856 | 0 | 0 | Actg1 | Neutrophils |
| 1.040189 | 0.667 | 0.049 | 0 | 0 | Snx20 | Neutrophils |
| 1.039954 | 0.527 | 0.072 | 0 | 0 | Ccl6 | Neutrophils |
| 1.030079 | 0.732 | 0.173 | 0 | 0 | Alox5ap | Neutrophils |
| 1.022913 | 0.392 | 0.056 | 0 | 0 | Isg15 | Neutrophils |
| 1.015074 | 0.732 | 0.238 | 0 | 0 | Nfe2l2 | Neutrophils |
| 1.004312 | 0.661 | 0.086 | 0 | 0 | Lilr4b | Neutrophils |
| 0.991675 | 0.891 | 0.485 | 0 | 0 | Cd9 | Neutrophils |
| 0.98349 | 0.578 | 0.028 | 0 | 0 | Mcemp1 | Neutrophils |
| 0.980616 | 0.532 | 0.061 | 0 | 0 | Resf1 | Neutrophils |
| 0.978416 | 0.676 | 0.165 | 0 | 0 | Ehd1 | Neutrophils |
| 0.975386 | 0.626 | 0.233 | 0 | 0 | Mrpl33 | Neutrophils |
| 0.974636 | 0.83 | 0.43 | 0 | 0 | Arpc3 | Neutrophils |
| 0.958685 | 0.747 | 0.275 | 0 | 0 | Kdm6b | Neutrophils |
| 0.956895 | 0.399 | 0.003 | 0 | 0 | Wfdc21 | Neutrophils |
| 0.954102 | 0.673 | 0.116 | 0 | 0 | Lilrb4a | Neutrophils |
| 0.9531 | 0.592 | 0.046 | 0 | 0 | Gsr | Neutrophils |
| 0.95135 | 0.578 | 0.02 | 0 | 0 | Cd300ld | Neutrophils |
| 0.949156 | 0.56 | 0.163 | 0 | 0 | Plaur | Neutrophils |
| 0.94307 | 0.606 | 0.031 | 0 | 0 | Sorl1 | Neutrophils |
| 0.93628 | 0.617 | 0.083 | 0 | 0 | Ncf2 | Neutrophils |
| 0.935397 | 0.558 | 0.023 | 0 | 0 | Dusp16 | Neutrophils |
| 0.933168 | 0.994 | 0.891 | 0 | 0 | Tmsb4x | Neutrophils |
| 0.931896 | 0.401 | 0.079 | 0 | 0 | Tnf | Neutrophils |
| 0.928863 | 0.623 | 0.065 | 0 | 0 | Rnf149 | Neutrophils |
| 0.928696 | 0.504 | 0.005 | 0 | 0 | Pglyrp1 | Neutrophils |
| 0.923481 | 0.167 | 0.002 | 0 | 0 | Cstdc5 | Neutrophils |
| 0.915763 | 0.528 | 0.013 | 0 | 0 | Sell | Neutrophils |
| 0.894276 | 0.543 | 0.018 | 0 | 0 | Arg2 | Neutrophils |
| 0.887393 | 0.56 | 0.058 | 0 | 0 | Nfkbid | Neutrophils |
| 0.879966 | 0.969 | 0.766 | 0 | 0 | Gm42418 | Neutrophils |
| 0.874 | 0.553 | 0.059 | 0 | 0 | Smox | Neutrophils |
| 0.869627 | 0.301 | 0.003 | 0 | 0 | Asprv1 | Neutrophils |
| 0.866266 | 0.602 | 0.091 | 0 | 0 | Pla2g7 | Neutrophils |
| 0.8642 | 0.595 | 0.072 | 0 | 0 | Slc15a3 | Neutrophils |
| 0.854598 | 0.418 | 0.003 | 0 | 0 | Il1f9 | Neutrophils |
| 0.852367 | 0.429 | 0.004 | 0 | 0 | Fpr1 | Neutrophils |
| 0.840949 | 0.522 | 0.045 | 0 | 0 | Ncf1 | Neutrophils |
| 0.83683 | 0.629 | 0.121 | 0 | 0 | Ncf4 | Neutrophils |
| 0.836428 | 0.612 | 0.106 | 0 | 0 | Rac2 | Neutrophils |
| 0.823946 | 0.509 | 0.073 | 0 | 0 | Txnip | Neutrophils |
| 0.81896 | 0.492 | 0.103 | 0 | 0 | Icam1 | Neutrophils |
| 0.815907 | 0.564 | 0.134 | 0 | 0 | Stk17b | Neutrophils |
| 0.810569 | 0.563 | 0.085 | 0 | 0 | Ndel1 | Neutrophils |
| 0.805523 | 0.557 | 0.045 | 0 | 0 | Snap23 | Neutrophils |
| 0.804185 | 0.607 | 0.151 | 0 | 0 | Spi1 | Neutrophils |
| 0.801062 | 0.44 | 0.095 | 0 | 0 | Clec4n | Neutrophils |
| 0.796775 | 0.616 | 0.108 | 0 | 0 | Rhog | Neutrophils |
| 0.794126 | 0.687 | 0.211 | 0 | 0 | Coro1a | Neutrophils |
| 0.780398 | 0.581 | 0.18 | 0 | 0 | Fosl2 | Neutrophils |
| 0.77858 | 0.59 | 0.169 | 0 | 0 | Ppp1r15a | Neutrophils |
| 0.774513 | 0.335 | 0.004 | 0 | 0 | F630028O10Rik | Neutrophils |
| 0.767839 | 0.447 | 0.028 | 0 | 0 | Cd24a | Neutrophils |
| 0.752662 | 0.428 | 0.086 | 0 | 0 | Dusp2 | Neutrophils |
| 0.750803 | 0.454 | 0.011 | 0 | 0 | Fpr2 | Neutrophils |
| 0.722102 | 0.537 | 0.106 | 0 | 0 | Pde4b | Neutrophils |
| 0.718555 | 0.535 | 0.134 | 0 | 0 | Rab7 | Neutrophils |
| 0.706469 | 0.462 | 0.066 | 0 | 0 | Glrx | Neutrophils |
| 0.695199 | 0.435 | 0.039 | 0 | 0 | Rab11fip1 | Neutrophils |
| 0.691108 | 0.502 | 0.08 | 0 | 0 | Csf2rb | Neutrophils |
| 0.687874 | 0.449 | 0.011 | 0 | 0 | Cd33 | Neutrophils |
| 0.681843 | 0.489 | 0.114 | 0 | 0 | Tlr2 | Neutrophils |
| 0.67955 | 0.506 | 0.102 | 0 | 0 | Vasp | Neutrophils |
| 0.676524 | 0.442 | 0.037 | 0 | 0 | Gcnt2 | Neutrophils |
| 0.673146 | 0.423 | 0.023 | 0 | 0 | Igsf6 | Neutrophils |
| 0.659515 | 0.377 | 0.067 | 0 | 0 | Gdpd3 | Neutrophils |
| 0.659055 | 0.498 | 0.084 | 0 | 0 | Emilin2 | Neutrophils |
| 0.658813 | 0.401 | 0.014 | 0 | 0 | Il1rap | Neutrophils |
| 0.651199 | 0.426 | 0.018 | 0 | 0 | Mmp9 | Neutrophils |
| 0.645765 | 0.467 | 0.062 | 0 | 0 | Snx18 | Neutrophils |
| 0.644148 | 0.417 | 0.08 | 0 | 0 | Tgm2 | Neutrophils |
| 0.641083 | 0.446 | 0.022 | 0 | 0 | Fgr | Neutrophils |
| 0.639654 | 0.463 | 0.088 | 0 | 0 | Trib1 | Neutrophils |
| 0.639319 | 0.427 | 0.009 | 0 | 0 | Pilra | Neutrophils |
| 0.634149 | 0.588 | 0.193 | 0 | 0 | Myl12b | Neutrophils |
| 0.62991 | 0.45 | 0.064 | 0 | 0 | Selplg | Neutrophils |
| 0.622327 | 0.404 | 0.06 | 0 | 0 | Fgl2 | Neutrophils |
| 0.622238 | 0.423 | 0.056 | 0 | 0 | Birc3 | Neutrophils |
| 0.617396 | 0.489 | 0.095 | 0 | 0 | Tnfrsf1b | Neutrophils |
| 0.614882 | 0.359 | 0.043 | 0 | 0 | Ltb | Neutrophils |
| 0.613426 | 0.422 | 0.058 | 0 | 0 | Rab20 | Neutrophils |
| 0.612198 | 0.394 | 0.032 | 0 | 0 | Stx11 | Neutrophils |
| 0.610757 | 0.471 | 0.09 | 0 | 0 | Rab8b | Neutrophils |
| 0.609986 | 0.386 | 0.006 | 0 | 0 | 2310001H17Rik | Neutrophils |
| 0.609445 | 0.424 | 0.053 | 0 | 0 | Tspan13 | Neutrophils |
| 0.602111 | 0.391 | 0.02 | 0 | 0 | Itgal | Neutrophils |
| 0.599776 | 0.461 | 0.118 | 0 | 0 | Txnrd1 | Neutrophils |
| 0.593013 | 0.369 | 0.026 | 0 | 0 | Mpp7 | Neutrophils |
| 0.592582 | 0.484 | 0.111 | 0 | 0 | Cap1 | Neutrophils |
| 0.586845 | 0.327 | 0.021 | 0 | 0 | Fbxl5 | Neutrophils |
| 0.577483 | 0.423 | 0.063 | 0 | 0 | 07-Mar | Neutrophils |
| 0.576632 | 0.435 | 0.08 | 0 | 0 | Adam8 | Neutrophils |
| 0.575716 | 0.381 | 0.043 | 0 | 0 | Gda | Neutrophils |
| 0.572275 | 0.43 | 0.086 | 0 | 0 | Zyx | Neutrophils |
| 0.565098 | 0.422 | 0.056 | 0 | 0 | Adipor1 | Neutrophils |
| 0.560088 | 0.426 | 0.075 | 0 | 0 | Tgoln1 | Neutrophils |
| 0.550352 | 0.372 | 0.031 | 0 | 0 | Antxr2 | Neutrophils |
| 0.548076 | 0.32 | 0.029 | 0 | 0 | Gadd45a | Neutrophils |
| 0.541866 | 0.39 | 0.07 | 0 | 0 | Dennd4a | Neutrophils |
| 0.538851 | 0.337 | 0.011 | 0 | 0 | Ankrd33b | Neutrophils |
| 0.532801 | 0.367 | 0.062 | 0 | 0 | N4bp1 | Neutrophils |
| 0.531269 | 0.362 | 0.067 | 0 | 0 | Adgre5 | Neutrophils |
| 0.530023 | 0.391 | 0.057 | 0 | 0 | Neurl3 | Neutrophils |
| 0.522706 | 0.275 | 0.003 | 0 | 0 | Slfn4 | Neutrophils |
| 0.520088 | 0.41 | 0.091 | 0 | 0 | Ddx6 | Neutrophils |
| 0.519171 | 0.314 | 0.017 | 0 | 0 | Chil1 | Neutrophils |
| 0.513941 | 0.394 | 0.078 | 0 | 0 | Ell2 | Neutrophils |
| 0.513476 | 0.386 | 0.053 | 0 | 0 | Fam32a | Neutrophils |
| 0.50715 | 0.293 | 0.007 | 0 | 0 | Slfn1 | Neutrophils |
| 0.501812 | 0.317 | 0.001 | 0 | 0 | Mirt2 | Neutrophils |
| 0.500791 | 0.31 | 0.023 | 0 | 0 | Cd274 | Neutrophils |
| 0.495634 | 0.386 | 0.072 | 0 | 0 | Samhd1 | Neutrophils |
| 0.493589 | 0.358 | 0.049 | 0 | 0 | Mir22hg | Neutrophils |
| 0.488456 | 0.242 | 0.005 | 0 | 0 | Upp1 | Neutrophils |
| 0.48734 | 0.318 | 0.013 | 0 | 0 | Lyst | Neutrophils |
| 0.48695 | 0.313 | 0.002 | 0 | 0 | Trem3 | Neutrophils |
| 0.486179 | 0.288 | 0.003 | 0 | 0 | Dgat2 | Neutrophils |
| 0.481535 | 0.324 | 0.003 | 0 | 0 | Themis2 | Neutrophils |
| 0.48048 | 0.373 | 0.064 | 0 | 0 | Tut7 | Neutrophils |
| 0.478053 | 0.31 | 0.011 | 0 | 0 | Cd300lf | Neutrophils |
| 0.476371 | 0.244 | 0.017 | 0 | 0 | AA467197 | Neutrophils |
| 0.476336 | 0.301 | 0.022 | 0 | 0 | Mefv | Neutrophils |
| 0.468793 | 0.301 | 0.021 | 0 | 0 | Fas | Neutrophils |
| 0.468179 | 0.336 | 0.031 | 0 | 0 | Hcst | Neutrophils |
| 0.467371 | 0.387 | 0.081 | 0 | 0 | Coq10b | Neutrophils |
| 0.464759 | 0.329 | 0.014 | 0 | 0 | Lpcat2 | Neutrophils |
| 0.464287 | 0.353 | 0.05 | 0 | 0 | Skap2 | Neutrophils |
| 0.463207 | 0.362 | 0.061 | 0 | 0 | Sde2 | Neutrophils |
| 0.458159 | 0.276 | 0.02 | 0 | 0 | Sgms2 | Neutrophils |
| 0.457411 | 0.315 | 0.018 | 0 | 0 | Selenon | Neutrophils |
| 0.449429 | 0.366 | 0.065 | 0 | 0 | Hcls1 | Neutrophils |
| 0.448702 | 0.331 | 0.037 | 0 | 0 | Rabgef1 | Neutrophils |
| 0.44511 | 0.324 | 0.05 | 0 | 0 | Nfkbie | Neutrophils |
| 0.443706 | 0.348 | 0.057 | 0 | 0 | Bcl3 | Neutrophils |
| 0.438349 | 0.299 | 0.028 | 0 | 0 | Lcp2 | Neutrophils |
| 0.4357 | 0.299 | 0.031 | 0 | 0 | Pik3ap1 | Neutrophils |
| 0.43387 | 0.282 | 0.027 | 0 | 0 | Cd300a | Neutrophils |
| 0.432359 | 0.289 | 0.019 | 0 | 0 | Spata13 | Neutrophils |
| 0.431306 | 0.329 | 0.029 | 0 | 0 | Fmnl1 | Neutrophils |
| 0.42791 | 0.311 | 0.048 | 0 | 0 | Klf3 | Neutrophils |
| 0.427203 | 0.286 | 0.025 | 0 | 0 | Lrrc25 | Neutrophils |
| 0.422554 | 0.256 | 0.014 | 0 | 0 | Cpeb2 | Neutrophils |
| 0.421608 | 0.273 | 0.02 | 0 | 0 | Alas1 | Neutrophils |
| 0.416733 | 0.278 | 0.004 | 0 | 0 | Ppp1r3b | Neutrophils |
| 0.415707 | 0.312 | 0.037 | 0 | 0 | Atg3 | Neutrophils |
| 0.414307 | 0.307 | 0.038 | 0 | 0 | Retreg1 | Neutrophils |
| 0.408805 | 0.282 | 0.025 | 0 | 0 | Snx10 | Neutrophils |
| 0.398451 | 0.258 | 0.007 | 0 | 0 | Gk | Neutrophils |
| 0.39674 | 0.252 | 0.001 | 0 | 0 | Ptgs2os2 | Neutrophils |
| 0.395553 | 0.307 | 0.049 | 0 | 0 | Syk | Neutrophils |
| 0.390838 | 0.257 | 0.017 | 0 | 0 | Gm26740 | Neutrophils |
| 0.388372 | 0.249 | 0.013 | 0 | 0 | Ogfrl1 | Neutrophils |
| 0.38825 | 0.246 | 0 | 0 | 0 | Mrgpra2a | Neutrophils |
| 0.384659 | 0.273 | 0.027 | 0 | 0 | Nadk | Neutrophils |
| 0.381924 | 0.261 | 0.014 | 0 | 0 | Sirpb1c | Neutrophils |
| 0.380873 | 0.254 | 0.014 | 0 | 0 | Cd80 | Neutrophils |
| 0.379025 | 0.225 | 0.01 | 0 | 0 | Isg20 | Neutrophils |
| 0.376908 | 0.233 | 0.004 | 0 | 0 | 4833407H14Rik | Neutrophils |
| 0.369717 | 0.227 | 0.01 | 0 | 0 | Jaml | Neutrophils |
| 0.369043 | 0.251 | 0.008 | 0 | 0 | Bst1 | Neutrophils |
| 0.36644 | 0.239 | 0.002 | 0 | 0 | Siglece | Neutrophils |
| 0.364168 | 0.255 | 0.023 | 0 | 0 | Plk3 | Neutrophils |
| 0.357023 | 0.244 | 0.009 | 0 | 0 | Slc2a6 | Neutrophils |
| 0.356349 | 0.228 | 0.005 | 0 | 0 | Mirt1 | Neutrophils |
| 0.352518 | 0.241 | 0.008 | 0 | 0 | Pygl | Neutrophils |
| 0.350387 | 0.242 | 0.022 | 0 | 0 | Gpcpd1 | Neutrophils |
| 0.348334 | 0.275 | 0.038 | 0 | 0 | Gpsm3 | Neutrophils |
| 0.347692 | 0.237 | 0.017 | 0 | 0 | Arrdc4 | Neutrophils |
| 0.339481 | 0.208 | 0.012 | 0 | 0 | Pts | Neutrophils |
| 0.338703 | 0.234 | 0.02 | 0 | 0 | AB124611 | Neutrophils |
| 0.334614 | 0.21 | 0.001 | 0 | 0 | Mrgpra2b | Neutrophils |
| 0.331639 | 0.165 | 0.005 | 0 | 0 | Slc2a3 | Neutrophils |
| 0.331134 | 0.218 | 0.01 | 0 | 0 | Nfam1 | Neutrophils |
| 0.322574 | 0.23 | 0.019 | 0 | 0 | Il10ra | Neutrophils |
| 0.312896 | 0.211 | 0.014 | 0 | 0 | Borcs6 | Neutrophils |
| 0.311492 | 0.207 | 0.003 | 0 | 0 | Il18rap | Neutrophils |
| 0.309665 | 0.171 | 0.004 | 0 | 0 | Gpr84 | Neutrophils |
| 0.306288 | 0.225 | 0.018 | 0 | 0 | Hck | Neutrophils |
| 0.305859 | 0.201 | 0.004 | 0 | 0 | Gm5150 | Neutrophils |
| 0.290592 | 0.176 | 0.011 | 0 | 0 | Cdc42ep2 | Neutrophils |
| 0.290545 | 0.188 | 0.001 | 0 | 0 | Dhrs9 | Neutrophils |
| 0.288165 | 0.183 | 0.005 | 0 | 0 | Tarm1 | Neutrophils |
| 0.282202 | 0.174 | 0.003 | 0 | 0 | Ebi3 | Neutrophils |
| 0.272307 | 0.182 | 0.007 | 0 | 0 | Ripor2 | Neutrophils |
| 0.270044 | 0.195 | 0.01 | 0 | 0 | Mboat7 | Neutrophils |
| 0.264698 | 0.186 | 0.005 | 0 | 0 | Klra2 | Neutrophils |
| 0.258056 | 0.178 | 0.012 | 0 | 0 | Itprip | Neutrophils |
| 0.257842 | 0.164 | 0.001 | 0 | 0 | Rab44 | Neutrophils |
| 0.253179 | 0.159 | 0.005 | 0 | 0 | St3gal6 | Neutrophils |
| 0.267726 | 0.185 | 0.014 | 2.55E-302 | 0 | Rab32 | Neutrophils |
| 0.329253 | 0.12 | 0.002 | 6.34E-302 | 0 | Il23a | Neutrophils |
| 0.492545 | 0.428 | 0.102 | 1.65E-301 | 0 | Lyn | Neutrophils |
| 0.674286 | 0.537 | 0.165 | 7.21E-301 | 0 | Ninj1 | Neutrophils |
| 0.687818 | 0.568 | 0.187 | 1.17E-300 | 0 | Hmgb2 | Neutrophils |
| 0.343782 | 0.249 | 0.032 | 8.14E-299 | 0 | Ccpg1 | Neutrophils |
| 0.535357 | 0.414 | 0.1 | 9.17E-293 | 0 | Mapkapk2 | Neutrophils |
| 0.293865 | 0.22 | 0.024 | 2.09E-292 | 0 | Myd88 | Neutrophils |
| 0.272708 | 0.185 | 0.015 | 2.35E-292 | 0 | Nabp1 | Neutrophils |
| 0.620633 | 0.506 | 0.156 | 3.11E-292 | 0 | Spag9 | Neutrophils |
| 0.700836 | 0.617 | 0.237 | 1.93E-291 | 0 | Picalm | Neutrophils |
| 0.406597 | 0.284 | 0.044 | 1.16E-290 | 0 | Slc11a1 | Neutrophils |
| 0.350219 | 0.271 | 0.04 | 6.03E-290 | 0 | Notch2 | Neutrophils |
| 0.375974 | 0.3 | 0.051 | 5.36E-284 | 0 | Nup98 | Neutrophils |
| 0.287959 | 0.202 | 0.02 | 2.20E-283 | 0 | Gpr35 | Neutrophils |
| 0.258976 | 0.189 | 0.017 | 2.88E-281 | 0 | Zswim4 | Neutrophils |
| 0.538143 | 0.464 | 0.133 | 6.04E-273 | 0 | Csrnp1 | Neutrophils |
| 0.591032 | 0.484 | 0.146 | 9.28E-272 | 0 | Card19 | Neutrophils |
| 0.429864 | 0.307 | 0.058 | 1.91E-267 | 0 | Zc3h12a | Neutrophils |
| 0.476 | 0.353 | 0.078 | 2.41E-267 | 0 | Fcgr3 | Neutrophils |
| 0.336966 | 0.202 | 0.022 | 1.31E-266 | 0 | Prkd3 | Neutrophils |
| 0.406416 | 0.342 | 0.072 | 1.55E-266 | 0 | Ssu72 | Neutrophils |
| 0.616314 | 0.618 | 0.252 | 3.36E-262 | 0 | Rap1b | Neutrophils |
| 0.366385 | 0.289 | 0.052 | 2.93E-260 | 0 | Il10rb | Neutrophils |
| 0.361886 | 0.258 | 0.04 | 1.53E-259 | 0 | Sema4d | Neutrophils |
| 0.656242 | 0.659 | 0.291 | 3.81E-259 | 0 | Ier5 | Neutrophils |
| 0.274078 | 0.19 | 0.02 | 1.60E-258 | 0 | Irak2 | Neutrophils |
| 0.287227 | 0.202 | 0.023 | 1.85E-258 | 0 | Carhsp1 | Neutrophils |
| 0.410344 | 0.364 | 0.084 | 5.02E-257 | 0 | Itgb2 | Neutrophils |
| 1.177384 | 0.298 | 0.056 | 2.28E-256 | 0 | Cxcl3 | Neutrophils |
| 0.262116 | 0.199 | 0.023 | 1.94E-250 | 0 | Anxa11 | Neutrophils |
| 0.295541 | 0.216 | 0.028 | 5.08E-250 | 0 | Dhrs7 | Neutrophils |
| 0.547163 | 0.209 | 0.026 | 6.75E-249 | 0 | Il1a | Neutrophils |
| 0.606997 | 0.374 | 0.095 | 1.30E-248 | 0 | Bcl2l11 | Neutrophils |
| 0.254532 | 0.2 | 0.024 | 1.79E-247 | 0 | Cmip | Neutrophils |
| 1.021199 | 0.658 | 0.283 | 1.14E-245 | 0 | Ptgs2 | Neutrophils |
| 0.282973 | 0.194 | 0.022 | 2.92E-245 | 0 | Ankrd44 | Neutrophils |
| 0.337641 | 0.264 | 0.046 | 2.40E-241 | 0 | Baz2b | Neutrophils |
| 0.251665 | 0.176 | 0.018 | 3.79E-241 | 0 | Trim30a | Neutrophils |
| 0.26316 | 0.182 | 0.019 | 1.03E-240 | 0 | Sp140 | Neutrophils |
| 0.401731 | 0.312 | 0.066 | 6.31E-240 | 0 | Emd | Neutrophils |
| 0.255546 | 0.17 | 0.017 | 3.78E-239 | 0 | Dmxl2 | Neutrophils |
| 0.37002 | 0.286 | 0.056 | 9.66E-238 | 0 | Kpna4 | Neutrophils |
| 0.339801 | 0.244 | 0.04 | 2.02E-236 | 0 | Apbb1ip | Neutrophils |
| 0.401207 | 0.352 | 0.085 | 2.25E-236 | 0 | Diaph1 | Neutrophils |
| 0.274215 | 0.214 | 0.03 | 1.85E-234 | 0 | Arih2 | Neutrophils |
| 0.749734 | 0.6 | 0.242 | 1.89E-234 | 0 | Klf2 | Neutrophils |
| 0.668212 | 0.946 | 0.854 | 5.93E-233 | 0 | H3f3a | Neutrophils |
| 0.526888 | 0.512 | 0.183 | 8.46E-233 | 0 | Sf3b1 | Neutrophils |
| 0.378054 | 0.279 | 0.055 | 8.64E-230 | 0 | Klf7 | Neutrophils |
| 0.396689 | 0.351 | 0.086 | 2.39E-228 | 0 | Itgam | Neutrophils |
| 0.265632 | 0.175 | 0.019 | 2.01E-226 | 0 | Tmcc1 | Neutrophils |
| 1.012527 | 0.76 | 0.436 | 2.75E-225 | 0 | Zfp36 | Neutrophils |
| 0.279042 | 0.21 | 0.03 | 3.00E-224 | 0 | Slc2a1 | Neutrophils |
| 0.493048 | 0.52 | 0.189 | 3.71E-224 | 0 | Ube2b | Neutrophils |
| 0.563868 | 0.569 | 0.233 | 5.72E-222 | 0 | Ostf1 | Neutrophils |
| 0.276443 | 0.155 | 0.015 | 2.15E-219 | 0 | Ifitm6 | Neutrophils |
| 0.513792 | 0.471 | 0.163 | 2.99E-218 | 0 | Tax1bp1 | Neutrophils |
| 0.590853 | 0.572 | 0.234 | 4.28E-218 | 0 | Lsp1 | Neutrophils |
| 0.291549 | 0.224 | 0.036 | 3.73E-217 | 0 | Osbpl9 | Neutrophils |
| 0.277296 | 0.197 | 0.027 | 1.47E-214 | 0 | Cd84 | Neutrophils |
| 0.257224 | 0.189 | 0.025 | 5.39E-214 | 0 | Fam111a | Neutrophils |
| 0.391364 | 0.296 | 0.066 | 2.23E-212 | 0 | Jdp2 | Neutrophils |
| 0.434244 | 0.407 | 0.125 | 8.87E-207 | 0 | Srsf5 | Neutrophils |
| 0.417218 | 0.398 | 0.121 | 4.78E-206 | 0 | Dazap2 | Neutrophils |
| 0.415243 | 0.342 | 0.091 | 1.54E-205 | 0 | Prr13 | Neutrophils |
| 0.536269 | 0.555 | 0.235 | 1.12E-204 | 0 | Actr3 | Neutrophils |
| 0.677509 | 0.685 | 0.392 | 1.32E-204 | 0 | Map1lc3b | Neutrophils |
| 0.38896 | 0.301 | 0.071 | 2.21E-203 | 0 | Fam107b | Neutrophils |
| 0.687322 | 0.992 | 0.956 | 5.97E-203 | 0 | Actb | Neutrophils |
| 0.255431 | 0.189 | 0.027 | 1.29E-201 | 0 | Tet2 | Neutrophils |
| 0.298349 | 0.236 | 0.044 | 7.97E-200 | 0 | Cyth4 | Neutrophils |
| 0.310356 | 0.216 | 0.037 | 2.22E-199 | 0 | Casp4 | Neutrophils |
| 0.280246 | 0.21 | 0.035 | 9.69E-199 | 0 | Ptpn6 | Neutrophils |
| 0.312519 | 0.249 | 0.05 | 1.19E-198 | 0 | Hnrnph2 | Neutrophils |
| 0.727124 | 0.943 | 0.891 | 2.72E-196 | 0 | H3f3b | Neutrophils |
| 0.304815 | 0.262 | 0.055 | 4.43E-196 | 0 | Rnf11 | Neutrophils |
| 0.278293 | 0.22 | 0.039 | 5.13E-196 | 0 | Vps4b | Neutrophils |
| 0.471675 | 0.242 | 0.049 | 1.83E-195 | 0 | Med21 | Neutrophils |
| 0.822403 | 0.794 | 0.58 | 4.43E-193 | 0 | Txn1 | Neutrophils |
| 0.840858 | 0.504 | 0.214 | 2.40E-191 | 0 | Basp1 | Neutrophils |
| 0.318067 | 0.178 | 0.025 | 2.50E-191 | 0 | Steap4 | Neutrophils |
| 0.263938 | 0.206 | 0.035 | 5.30E-189 | 0 | Fem1c | Neutrophils |
| 0.645872 | 0.931 | 0.621 | 8.52E-187 | 0 | H2-D1 | Neutrophils |
| 0.256933 | 0.192 | 0.031 | 5.41E-186 | 0 | Rab8a | Neutrophils |
| 0.372105 | 0.185 | 0.029 | 4.08E-184 | 0 | Csf1 | Neutrophils |
| 0.479505 | 0.439 | 0.16 | 2.76E-182 | 0 | Skil | Neutrophils |
| 0.327485 | 0.261 | 0.059 | 1.79E-181 | 0 | Nab1 | Neutrophils |
| 0.617509 | 0.818 | 0.628 | 2.76E-181 | 0 | Ddx5 | Neutrophils |
| 0.270174 | 0.206 | 0.037 | 9.58E-180 | 0 | Glipr2 | Neutrophils |
| 0.252389 | 0.193 | 0.032 | 3.57E-179 | 0 | Il13ra1 | Neutrophils |
| 0.283595 | 0.242 | 0.052 | 1.10E-177 | 0 | Zfp106 | Neutrophils |
| 0.304772 | 0.225 | 0.045 | 2.68E-177 | 0 | Tes | Neutrophils |
| 0.618376 | 0.883 | 0.635 | 2.77E-177 | 0 | B2m | Neutrophils |
| 0.515446 | 0.564 | 0.264 | 1.73E-175 | 0 | Arpc5 | Neutrophils |
| 0.279986 | 0.2 | 0.036 | 2.57E-175 | 0 | Slc9a3r1 | Neutrophils |
| 0.28418 | 0.227 | 0.046 | 7.11E-175 | 0 | Chd2 | Neutrophils |
| 0.283316 | 0.241 | 0.052 | 2.14E-174 | 0 | Grk2 | Neutrophils |
| 0.381397 | 0.366 | 0.117 | 6.97E-170 | 0 | Ppp1r2 | Neutrophils |
| 0.265253 | 0.23 | 0.048 | 1.89E-169 | 0 | Tcn2 | Neutrophils |
| 0.25999 | 0.209 | 0.04 | 8.22E-169 | 0 | Chd7 | Neutrophils |
| 0.302682 | 0.24 | 0.054 | 4.76E-168 | 0 | Igf1r | Neutrophils |
| 0.264304 | 0.218 | 0.044 | 8.72E-168 | 0 | Oser1 | Neutrophils |
| 0.269105 | 0.23 | 0.049 | 2.78E-167 | 0 | Glipr1 | Neutrophils |
| 0.535564 | 0.509 | 0.23 | 1.61E-165 | 0 | Vmp1 | Neutrophils |
| 0.480435 | 0.272 | 0.071 | 5.44E-165 | 0 | Cks2 | Neutrophils |
| 0.274745 | 0.22 | 0.047 | 5.87E-162 | 0 | Csf2ra | Neutrophils |
| 0.378574 | 0.345 | 0.111 | 4.24E-159 | 0 | Supt4a | Neutrophils |
| 0.37536 | 0.397 | 0.135 | 5.94E-159 | 0 | Cytip | Neutrophils |
| 0.300847 | 0.263 | 0.067 | 4.33E-158 | 0 | Maff | Neutrophils |
| 0.536633 | 0.466 | 0.198 | 1.53E-157 | 0 | Nudt4 | Neutrophils |
| 0.345251 | 0.342 | 0.109 | 9.04E-157 | 0 | Grb2 | Neutrophils |
| 0.384474 | 0.31 | 0.094 | 1.68E-152 | 0 | Clec2d | Neutrophils |
| 0.354058 | 0.336 | 0.109 | 2.65E-151 | 0 | Pak2 | Neutrophils |
| 0.278191 | 0.249 | 0.063 | 3.24E-151 | 0 | Cript | Neutrophils |
| 0.503627 | 0.619 | 0.337 | 7.63E-150 | 0 | Iqgap1 | Neutrophils |
| 0.535462 | 0.536 | 0.256 | 6.75E-149 | 0 | Sat1 | Neutrophils |
| 0.360153 | 0.328 | 0.105 | 1.04E-147 | 0 | Fyb | Neutrophils |
| 0.53217 | 0.318 | 0.103 | 2.32E-146 | 0 | Traf1 | Neutrophils |
| 0.263976 | 0.209 | 0.046 | 3.79E-146 | 0 | Ppt1 | Neutrophils |
| 0.316921 | 0.304 | 0.093 | 5.04E-144 | 0 | Kras | Neutrophils |
| 0.415299 | 0.35 | 0.125 | 3.40E-141 | 0 | Tgif1 | Neutrophils |
| 0.291332 | 0.241 | 0.063 | 1.75E-139 | 0 | Sbno1 | Neutrophils |
| 0.327057 | 0.261 | 0.074 | 1.89E-137 | 0 | Nfil3 | Neutrophils |
| 0.44075 | 0.502 | 0.231 | 3.77E-137 | 0 | Sdcbp | Neutrophils |
| 0.48536 | 0.369 | 0.14 | 4.80E-132 | 0 | Cxcr4 | Neutrophils |
| 0.572262 | 0.873 | 0.785 | 6.91E-131 | 0 | Pfn1 | Neutrophils |
| 0.287324 | 0.242 | 0.067 | 3.33E-129 | 0 | Kdm7a | Neutrophils |
| 0.536636 | 0.741 | 0.582 | 1.45E-128 | 0 | Gng5 | Neutrophils |
| 0.275952 | 0.242 | 0.067 | 3.61E-128 | 0 | Cdkn1b | Neutrophils |
| 0.422203 | 0.46 | 0.212 | 2.07E-125 | 0 | Ccnl1 | Neutrophils |
| 0.374605 | 0.419 | 0.18 | 1.13E-124 | 0 | Chmp4b | Neutrophils |
| 0.261673 | 0.236 | 0.065 | 2.38E-124 | 0 | Por | Neutrophils |
| 0.290771 | 0.269 | 0.084 | 1.55E-122 | 0 | Pet100 | Neutrophils |
| 0.402589 | 0.692 | 0.37 | 5.40E-122 | 0 | Cyba | Neutrophils |
| 0.338294 | 0.362 | 0.14 | 2.92E-121 | 0 | Ptbp3 | Neutrophils |
| 0.275052 | 0.238 | 0.068 | 1.00E-119 | 0 | Gm26532 | Neutrophils |
| 0.499261 | 0.492 | 0.258 | 9.06E-119 | 0 | D8Ertd738e | Neutrophils |
| 0.381811 | 0.423 | 0.189 | 1.14E-118 | 0 | Rbms1 | Neutrophils |
| 0.568133 | 0.967 | 0.906 | 5.31E-118 | 0 | Ftl1 | Neutrophils |
| 0.371537 | 0.397 | 0.166 | 8.23E-118 | 0 | Cotl1 | Neutrophils |
| 0.257564 | 0.249 | 0.075 | 9.15E-118 | 0 | Ppp1r18 | Neutrophils |
| 0.392606 | 0.509 | 0.261 | 1.08E-117 | 0 | Tmbim6 | Neutrophils |
| 0.322519 | 0.344 | 0.133 | 2.48E-115 | 0 | Ypel3 | Neutrophils |
| 0.294209 | 0.279 | 0.095 | 4.48E-112 | 0 | Sra1 | Neutrophils |
| 0.48868 | 0.506 | 0.28 | 7.57E-112 | 0 | Lamp2 | Neutrophils |
| 0.261499 | 0.272 | 0.09 | 2.72E-111 | 0 | Tnfrsf1a | Neutrophils |
| 0.281976 | 0.312 | 0.114 | 9.27E-111 | 0 | Fam49b | Neutrophils |
| 1.5635 | 0.225 | 0.066 | 3.65E-110 | 0 | Ccl4 | Neutrophils |
| 0.408798 | 0.449 | 0.218 | 5.68E-110 | 0 | H2afj | Neutrophils |
| 0.329916 | 0.328 | 0.127 | 8.12E-108 | 0 | Gm2a | Neutrophils |
| 0.378618 | 0.405 | 0.18 | 7.38E-107 | 0 | Efhd2 | Neutrophils |
| 0.415581 | 0.464 | 0.234 | 1.76E-106 | 0 | Ddx3x | Neutrophils |
| 0.275249 | 0.211 | 0.061 | 1.02E-104 | 0 | Smchd1 | Neutrophils |
| 0.307482 | 0.213 | 0.061 | 5.77E-104 | 0 | Osm | Neutrophils |
| 0.280061 | 0.287 | 0.105 | 1.35E-101 | 0 | Map2k3 | Neutrophils |
| 0.417436 | 0.538 | 0.31 | 1.22E-100 | 0 | Msn | Neutrophils |
| 0.290878 | 0.269 | 0.097 | 7.95E-98 | 0 | Tmed5 | Neutrophils |
| 0.317859 | 0.504 | 0.246 | 2.21E-97 | 0 | Laptm5 | Neutrophils |
| 0.258772 | 0.244 | 0.082 | 2.72E-97 | 0 | Riok3 | Neutrophils |
| 0.29121 | 0.277 | 0.104 | 2.45E-92 | 0 | Cflar | Neutrophils |
| 0.440343 | 0.817 | 0.722 | 2.74E-92 | 0 | Gabarap | Neutrophils |
| 0.313728 | 0.3 | 0.121 | 3.36E-92 | 0 | Ptpn1 | Neutrophils |
| 0.359728 | 0.293 | 0.115 | 3.98E-92 | 0 | Nr4a3 | Neutrophils |
| 0.307148 | 0.359 | 0.16 | 6.86E-91 | 0 | Clk1 | Neutrophils |
| 0.293333 | 0.306 | 0.125 | 7.47E-90 | 0 | Tra2a | Neutrophils |
| 0.586726 | 0.415 | 0.223 | 3.91E-87 | 0 | Sod2 | Neutrophils |
| 0.273065 | 0.322 | 0.134 | 5.34E-87 | 0 | Rel | Neutrophils |
| 0.334527 | 0.444 | 0.225 | 5.21E-86 | 0 | Arhgdib | Neutrophils |
| 0.388315 | 0.719 | 0.527 | 7.55E-86 | 0 | Arpc2 | Neutrophils |
| 0.358491 | 0.477 | 0.268 | 6.28E-85 | 0 | Capzb | Neutrophils |
| 0.398984 | 0.698 | 0.504 | 9.06E-84 | 0 | Arpc1b | Neutrophils |
| 0.261657 | 0.249 | 0.095 | 5.24E-79 | 0 | Ezr | Neutrophils |
| 0.396084 | 0.527 | 0.336 | 9.17E-78 | 0 | Atp6v1g1 | Neutrophils |
| 0.250929 | 0.243 | 0.09 | 1.00E-77 | 0 | Malt1 | Neutrophils |
| 0.368712 | 0.369 | 0.183 | 4.14E-77 | 0 | Atp6v1e1 | Neutrophils |
| 0.273399 | 0.268 | 0.109 | 3.55E-76 | 0 | Dusp5 | Neutrophils |
| 0.265567 | 0.342 | 0.161 | 8.57E-75 | 0 | Actr2 | Neutrophils |
| 0.258463 | 0.32 | 0.149 | 1.35E-70 | 0 | Jak1 | Neutrophils |
| 0.376332 | 0.565 | 0.368 | 1.80E-70 | 0 | Pkm | Neutrophils |
| 0.334005 | 0.417 | 0.235 | 2.44E-64 | 0 | Atp2b1 | Neutrophils |
| 0.339118 | 0.343 | 0.179 | 8.32E-63 | 0 | Wnk1 | Neutrophils |
| 0.382677 | 0.613 | 0.422 | 2.10E-60 | 0 | Neat1 | Neutrophils |
| 0.252367 | 0.321 | 0.161 | 4.74E-60 | 0 | Vamp8 | Neutrophils |
| 0.336847 | 0.646 | 0.521 | 4.48E-52 | 0 | Cdc42 | Neutrophils |
| 0.29988 | 0.505 | 0.349 | 1.12E-49 | 0 | Ubl5 | Neutrophils |
| 0.294417 | 0.393 | 0.234 | 1.73E-48 | 0 | Ier2 | Neutrophils |
| 0.291328 | 0.509 | 0.354 | 4.30E-47 | 0 | Ube2d3 | Neutrophils |
| 0.279618 | 0.423 | 0.269 | 1.02E-46 | 0 | Rac1 | Neutrophils |
| 0.392216 | 0.643 | 0.481 | 5.81E-42 | 0 | Atp6v0c | Neutrophils |
| 0.541212 | 0.381 | 0.258 | 5.00E-34 | 0 | Thbs1 | Neutrophils |
| 0.273946 | 0.479 | 0.352 | 5.00E-33 | 0 | Atp6v0e | Neutrophils |
| 0.272667 | 0.515 | 0.405 | 1.65E-28 | 0 | Tpm4 | Neutrophils |
| 0.261404 | 0.491 | 0.368 | 4.20E-27 | 0 | Klf6 | Neutrophils |
| 0.268948 | 0.469 | 0.338 | 6.02E-26 | 0 | Nr4a1 | Neutrophils |
| 0.358016 | 0.381 | 0.292 | 2.16E-18 | 0 | Cdkn1a | Neutrophils |
| 0.259083 | 0.415 | 0.321 | 3.22E-18 | 0 | Dnaja1 | Neutrophils |
| 0.274364 | 0.702 | 0.674 | 4.23E-09 | 0 | Jund | Neutrophils |
| 1.494971 | 0.999 | 0.515 | 0 | 1 | Col1a1 | Myofibroblast (Myo 1.1) |
| 1.433352 | 0.999 | 0.526 | 0 | 1 | Col1a2 | Myofibroblast (Myo 1.1) |
| 1.358829 | 0.986 | 0.41 | 0 | 1 | Postn | Myofibroblast (Myo 1.1) |
| 1.314798 | 0.985 | 0.458 | 0 | 1 | Serpinh1 | Myofibroblast (Myo 1.1) |
| 1.297275 | 0.978 | 0.393 | 0 | 1 | Col6a3 | Myofibroblast (Myo 1.1) |
| 1.225936 | 0.983 | 0.367 | 0 | 1 | Col5a1 | Myofibroblast (Myo 1.1) |
| 1.225396 | 1 | 0.552 | 0 | 1 | Sparc | Myofibroblast (Myo 1.1) |
| 1.222268 | 0.987 | 0.425 | 0 | 1 | Col6a1 | Myofibroblast (Myo 1.1) |
| 1.19619 | 0.885 | 0.254 | 0 | 1 | Acta2 | Myofibroblast (Myo 1.1) |
| 1.122834 | 0.606 | 0.126 | 0 | 1 | Tnc | Myofibroblast (Myo 1.1) |
| 1.094326 | 0.99 | 0.452 | 0 | 1 | Col5a2 | Myofibroblast (Myo 1.1) |
| 1.087665 | 0.998 | 0.517 | 0 | 1 | Col3a1 | Myofibroblast (Myo 1.1) |
| 1.081036 | 0.777 | 0.171 | 0 | 1 | Cthrc1 | Myofibroblast (Myo 1.1) |
| 1.025886 | 0.927 | 0.287 | 0 | 1 | Col12a1 | Myofibroblast (Myo 1.1) |
| 1.025695 | 0.976 | 0.385 | 0 | 1 | Col6a2 | Myofibroblast (Myo 1.1) |
| 0.943022 | 0.821 | 0.29 | 0 | 1 | Csrp2 | Myofibroblast (Myo 1.1) |
| 0.940205 | 0.886 | 0.405 | 0 | 1 | Pmepa1 | Myofibroblast (Myo 1.1) |
| 0.923987 | 0.747 | 0.105 | 0 | 1 | Lrrc15 | Myofibroblast (Myo 1.1) |
| 0.885337 | 0.669 | 0.14 | 0 | 1 | Col8a1 | Myofibroblast (Myo 1.1) |
| 0.870898 | 0.87 | 0.335 | 0 | 1 | Tpm1 | Myofibroblast (Myo 1.1) |
| 0.864073 | 0.974 | 0.622 | 0 | 1 | Cd63 | Myofibroblast (Myo 1.1) |
| 0.851584 | 0.948 | 0.389 | 0 | 1 | Fstl1 | Myofibroblast (Myo 1.1) |
| 0.805082 | 0.957 | 0.69 | 0 | 1 | Nme2 | Myofibroblast (Myo 1.1) |
| 0.787894 | 0.776 | 0.225 | 0 | 1 | Col11a1 | Myofibroblast (Myo 1.1) |
| 0.782718 | 0.944 | 0.374 | 0 | 1 | Serpinf1 | Myofibroblast (Myo 1.1) |
| 0.762143 | 0.896 | 0.429 | 0 | 1 | Rrbp1 | Myofibroblast (Myo 1.1) |
| 0.747873 | 0.824 | 0.273 | 0 | 1 | Kdelr2 | Myofibroblast (Myo 1.1) |
| 0.704115 | 0.858 | 0.296 | 0 | 1 | Rcn3 | Myofibroblast (Myo 1.1) |
| 0.700522 | 0.579 | 0.124 | 0 | 1 | Tnn | Myofibroblast (Myo 1.1) |
| 0.699479 | 0.635 | 0.187 | 0 | 1 | Col5a3 | Myofibroblast (Myo 1.1) |
| 0.695271 | 0.934 | 0.425 | 0 | 1 | Cald1 | Myofibroblast (Myo 1.1) |
| 0.69474 | 0.399 | 0.038 | 0 | 1 | Col7a1 | Myofibroblast (Myo 1.1) |
| 0.669054 | 0.781 | 0.236 | 0 | 1 | Tpm2 | Myofibroblast (Myo 1.1) |
| 0.654288 | 0.616 | 0.137 | 0 | 1 | Loxl2 | Myofibroblast (Myo 1.1) |
| 0.642431 | 0.63 | 0.179 | 0 | 1 | Tagln | Myofibroblast (Myo 1.1) |
| 0.633762 | 0.842 | 0.34 | 0 | 1 | Selenom | Myofibroblast (Myo 1.1) |
| 0.627034 | 0.819 | 0.302 | 0 | 1 | Fbn1 | Myofibroblast (Myo 1.1) |
| 0.602571 | 0.722 | 0.237 | 0 | 1 | Lox | Myofibroblast (Myo 1.1) |
| 0.594507 | 0.714 | 0.214 | 0 | 1 | Ckap4 | Myofibroblast (Myo 1.1) |
| 0.581115 | 0.733 | 0.252 | 0 | 1 | Ostc | Myofibroblast (Myo 1.1) |
| 0.572776 | 0.566 | 0.13 | 0 | 1 | Nrep | Myofibroblast (Myo 1.1) |
| 0.537602 | 0.68 | 0.223 | 0 | 1 | Ssr2 | Myofibroblast (Myo 1.1) |
| 0.502861 | 0.614 | 0.18 | 0 | 1 | Loxl1 | Myofibroblast (Myo 1.1) |
| 0.478621 | 0.397 | 0.046 | 0 | 1 | Lrrc17 | Myofibroblast (Myo 1.1) |
| 0.476104 | 0.558 | 0.146 | 0 | 1 | Lman1 | Myofibroblast (Myo 1.1) |
| 0.471701 | 0.586 | 0.156 | 0 | 1 | Myl9 | Myofibroblast (Myo 1.1) |
| 0.438899 | 0.466 | 0.096 | 0 | 1 | Kdelr3 | Myofibroblast (Myo 1.1) |
| 0.410351 | 0.435 | 0.091 | 0 | 1 | Fkbp10 | Myofibroblast (Myo 1.1) |
| 0.392845 | 0.388 | 0.071 | 0 | 1 | Fkbp11 | Myofibroblast (Myo 1.1) |
| 0.647522 | 0.862 | 0.37 | 1.82E-303 | 1 | Ppic | Myofibroblast (Myo 1.1) |
| 0.677106 | 0.882 | 0.468 | 1.96E-298 | 1 | Sec61b | Myofibroblast (Myo 1.1) |
| 0.414681 | 0.412 | 0.085 | 3.75E-297 | 1 | Ccn4 | Myofibroblast (Myo 1.1) |
| 0.664505 | 0.683 | 0.233 | 1.17E-291 | 1 | Thbs2 | Myofibroblast (Myo 1.1) |
| 0.588494 | 0.674 | 0.228 | 6.17E-291 | 1 | Fbln2 | Myofibroblast (Myo 1.1) |
| 0.423089 | 0.313 | 0.049 | 8.92E-291 | 1 | C1qtnf3 | Myofibroblast (Myo 1.1) |
| 0.865835 | 0.974 | 0.512 | 6.50E-289 | 1 | Fn1 | Myofibroblast (Myo 1.1) |
| 0.851146 | 0.991 | 0.488 | 4.34E-286 | 1 | Bgn | Myofibroblast (Myo 1.1) |
| 0.685865 | 0.806 | 0.315 | 6.67E-282 | 1 | Timp1 | Myofibroblast (Myo 1.1) |
| 0.473146 | 0.606 | 0.185 | 2.40E-281 | 1 | Olfml3 | Myofibroblast (Myo 1.1) |
| 0.747559 | 0.462 | 0.114 | 3.24E-278 | 1 | Igfbp3 | Myofibroblast (Myo 1.1) |
| 0.556646 | 0.812 | 0.356 | 4.76E-275 | 1 | P4hb | Myofibroblast (Myo 1.1) |
| 0.486799 | 0.654 | 0.222 | 1.10E-273 | 1 | Calu | Myofibroblast (Myo 1.1) |
| 0.43211 | 0.493 | 0.131 | 2.36E-270 | 1 | Txndc5 | Myofibroblast (Myo 1.1) |
| 0.627838 | 0.923 | 0.584 | 3.63E-257 | 1 | Ppib | Myofibroblast (Myo 1.1) |
| 0.824997 | 0.985 | 0.716 | 7.73E-257 | 1 | Lgals1 | Myofibroblast (Myo 1.1) |
| 0.376866 | 0.424 | 0.102 | 8.92E-255 | 1 | Rcn1 | Myofibroblast (Myo 1.1) |
| 0.869622 | 0.978 | 0.711 | 2.93E-249 | 1 | Tmsb10 | Myofibroblast (Myo 1.1) |
| 0.67505 | 0.917 | 0.588 | 3.45E-241 | 1 | Sec61g | Myofibroblast (Myo 1.1) |
| 0.449719 | 0.536 | 0.168 | 5.56E-240 | 1 | Olfml2b | Myofibroblast (Myo 1.1) |
| 0.409497 | 0.515 | 0.154 | 3.00E-238 | 1 | Maged1 | Myofibroblast (Myo 1.1) |
| 0.55437 | 0.765 | 0.306 | 4.55E-237 | 1 | Aspn | Myofibroblast (Myo 1.1) |
| 0.748687 | 0.481 | 0.141 | 1.62E-233 | 1 | Crabp1 | Myofibroblast (Myo 1.1) |
| 0.608917 | 0.871 | 0.492 | 1.90E-232 | 1 | Hsp90b1 | Myofibroblast (Myo 1.1) |
| 0.419716 | 0.47 | 0.133 | 2.86E-230 | 1 | Palld | Myofibroblast (Myo 1.1) |
| 0.45885 | 0.704 | 0.285 | 3.38E-225 | 1 | Tceal9 | Myofibroblast (Myo 1.1) |
| 0.527853 | 0.91 | 0.36 | 7.87E-225 | 1 | Lum | Myofibroblast (Myo 1.1) |
| 0.506824 | 0.856 | 0.358 | 8.38E-225 | 1 | Aebp1 | Myofibroblast (Myo 1.1) |
| 0.501853 | 0.791 | 0.382 | 1.51E-223 | 1 | Pdia3 | Myofibroblast (Myo 1.1) |
| 0.434778 | 0.65 | 0.249 | 3.50E-221 | 1 | Hdlbp | Myofibroblast (Myo 1.1) |
| 0.416511 | 0.57 | 0.196 | 2.55E-219 | 1 | Ssr3 | Myofibroblast (Myo 1.1) |
| 0.30982 | 0.271 | 0.048 | 3.23E-219 | 1 | Angptl2 | Myofibroblast (Myo 1.1) |
| 0.427437 | 0.642 | 0.248 | 4.57E-213 | 1 | Tmed9 | Myofibroblast (Myo 1.1) |
| 0.399369 | 0.478 | 0.148 | 1.90E-210 | 1 | Wls | Myofibroblast (Myo 1.1) |
| 0.500516 | 0.703 | 0.3 | 1.16E-205 | 1 | Tuba1a | Myofibroblast (Myo 1.1) |
| 0.436801 | 0.61 | 0.225 | 1.13E-204 | 1 | Cdh11 | Myofibroblast (Myo 1.1) |
| 0.369124 | 0.424 | 0.121 | 8.69E-204 | 1 | Oaf | Myofibroblast (Myo 1.1) |
| 0.392406 | 0.374 | 0.095 | 4.57E-202 | 1 | Wnt5a | Myofibroblast (Myo 1.1) |
| 0.492509 | 0.751 | 0.333 | 6.26E-202 | 1 | Mmp14 | Myofibroblast (Myo 1.1) |
| 0.327708 | 0.327 | 0.076 | 1.53E-200 | 1 | Creb3l1 | Myofibroblast (Myo 1.1) |
| 0.323205 | 0.271 | 0.052 | 1.08E-199 | 1 | Bpgm | Myofibroblast (Myo 1.1) |
| 0.495614 | 0.971 | 0.808 | 3.72E-199 | 1 | Myl6 | Myofibroblast (Myo 1.1) |
| 0.37915 | 0.527 | 0.18 | 5.01E-199 | 1 | Tmed3 | Myofibroblast (Myo 1.1) |
| 0.492447 | 0.866 | 0.518 | 3.88E-197 | 1 | Myl12a | Myofibroblast (Myo 1.1) |
| 0.329192 | 0.285 | 0.059 | 3.54E-196 | 1 | Rflnb | Myofibroblast (Myo 1.1) |
| 0.469973 | 0.776 | 0.398 | 1.51E-195 | 1 | Ssr4 | Myofibroblast (Myo 1.1) |
| 0.481894 | 0.633 | 0.245 | 2.25E-195 | 1 | Htra1 | Myofibroblast (Myo 1.1) |
| 0.399617 | 0.535 | 0.189 | 6.14E-195 | 1 | Cnn3 | Myofibroblast (Myo 1.1) |
| 0.37878 | 0.525 | 0.186 | 9.59E-189 | 1 | Selenos | Myofibroblast (Myo 1.1) |
| 0.349807 | 0.316 | 0.074 | 1.34E-188 | 1 | Tgfb2 | Myofibroblast (Myo 1.1) |
| 0.49511 | 0.921 | 0.608 | 2.69E-188 | 1 | Gnas | Myofibroblast (Myo 1.1) |
| 0.409505 | 0.622 | 0.233 | 4.35E-185 | 1 | Gpx3 | Myofibroblast (Myo 1.1) |
| 0.362581 | 0.501 | 0.175 | 4.92E-182 | 1 | Arl1 | Myofibroblast (Myo 1.1) |
| 0.401111 | 0.628 | 0.264 | 9.34E-181 | 1 | Krtcap2 | Myofibroblast (Myo 1.1) |
| 0.407069 | 0.746 | 0.322 | 5.26E-180 | 1 | Pcolce | Myofibroblast (Myo 1.1) |
| 0.484349 | 0.82 | 0.458 | 1.35E-177 | 1 | Calr | Myofibroblast (Myo 1.1) |
| 0.43801 | 0.767 | 0.381 | 2.97E-175 | 1 | Tpm4 | Myofibroblast (Myo 1.1) |
| 0.296652 | 0.298 | 0.07 | 1.30E-174 | 1 | Plod2 | Myofibroblast (Myo 1.1) |
| 0.344801 | 0.43 | 0.138 | 2.86E-174 | 1 | Ift20 | Myofibroblast (Myo 1.1) |
| 0.518684 | 0.999 | 0.939 | 4.38E-174 | 1 | Rpl41 | Myofibroblast (Myo 1.1) |
| 0.391728 | 0.781 | 0.369 | 5.28E-174 | 1 | Nedd4 | Myofibroblast (Myo 1.1) |
| 0.331468 | 0.417 | 0.13 | 5.84E-174 | 1 | Copz2 | Myofibroblast (Myo 1.1) |
| 0.410386 | 0.608 | 0.259 | 5.04E-171 | 1 | Pdia6 | Myofibroblast (Myo 1.1) |
| 0.419753 | 0.78 | 0.413 | 6.11E-171 | 1 | Dad1 | Myofibroblast (Myo 1.1) |
| 0.370626 | 0.487 | 0.175 | 1.25E-168 | 1 | Cnn2 | Myofibroblast (Myo 1.1) |
| 0.334528 | 0.421 | 0.133 | 2.24E-167 | 1 | Csrp1 | Myofibroblast (Myo 1.1) |
| 0.437067 | 0.606 | 0.265 | 7.01E-167 | 1 | Manf | Myofibroblast (Myo 1.1) |
| 0.362255 | 0.546 | 0.213 | 5.30E-165 | 1 | Mtch1 | Myofibroblast (Myo 1.1) |
| 0.419007 | 0.397 | 0.123 | 1.00E-160 | 1 | Inhba | Myofibroblast (Myo 1.1) |
| 0.376986 | 0.756 | 0.385 | 1.99E-156 | 1 | Arf4 | Myofibroblast (Myo 1.1) |
| 0.271917 | 0.287 | 0.072 | 2.31E-154 | 1 | Srpx2 | Myofibroblast (Myo 1.1) |
| 0.771382 | 0.59 | 0.271 | 2.50E-153 | 1 | Fabp5 | Myofibroblast (Myo 1.1) |
| 0.369637 | 0.476 | 0.177 | 4.48E-153 | 1 | Fgfr1 | Myofibroblast (Myo 1.1) |
| 0.298633 | 0.33 | 0.094 | 2.59E-152 | 1 | Sec61a1 | Myofibroblast (Myo 1.1) |
| 0.308463 | 0.465 | 0.166 | 6.23E-151 | 1 | Vcan | Myofibroblast (Myo 1.1) |
| 0.384085 | 0.773 | 0.406 | 8.52E-151 | 1 | Slc25a4 | Myofibroblast (Myo 1.1) |
| 0.329687 | 0.418 | 0.144 | 7.26E-150 | 1 | Surf4 | Myofibroblast (Myo 1.1) |
| 0.34115 | 0.425 | 0.148 | 4.15E-149 | 1 | Bmp1 | Myofibroblast (Myo 1.1) |
| 0.391542 | 0.754 | 0.41 | 1.44E-147 | 1 | Reep5 | Myofibroblast (Myo 1.1) |
| 0.408369 | 0.74 | 0.383 | 1.08E-146 | 1 | Itgb1 | Myofibroblast (Myo 1.1) |
| 0.368869 | 0.769 | 0.409 | 1.06E-145 | 1 | Bsg | Myofibroblast (Myo 1.1) |
| 0.313897 | 0.43 | 0.153 | 8.98E-145 | 1 | Kdelr1 | Myofibroblast (Myo 1.1) |
| 0.327593 | 0.48 | 0.18 | 5.37E-144 | 1 | Mfge8 | Myofibroblast (Myo 1.1) |
| 0.330151 | 0.495 | 0.196 | 3.58E-142 | 1 | Swi5 | Myofibroblast (Myo 1.1) |
| 0.322879 | 0.407 | 0.143 | 2.16E-140 | 1 | C1qtnf6 | Myofibroblast (Myo 1.1) |
| 0.326015 | 0.443 | 0.164 | 3.85E-140 | 1 | Prdx4 | Myofibroblast (Myo 1.1) |
| 0.333175 | 0.54 | 0.227 | 2.10E-137 | 1 | Ppp1r14b | Myofibroblast (Myo 1.1) |
| 0.435941 | 0.966 | 0.827 | 1.63E-136 | 1 | Serf2 | Myofibroblast (Myo 1.1) |
| 0.493934 | 0.542 | 0.232 | 7.04E-136 | 1 | Meg3 | Myofibroblast (Myo 1.1) |
| 0.443693 | 0.866 | 0.612 | 2.17E-134 | 1 | Ybx1 | Myofibroblast (Myo 1.1) |
| 0.281947 | 0.251 | 0.063 | 3.06E-133 | 1 | Fibin | Myofibroblast (Myo 1.1) |
| 0.334908 | 0.574 | 0.257 | 1.11E-132 | 1 | Dstn | Myofibroblast (Myo 1.1) |
| 0.278368 | 0.331 | 0.103 | 1.51E-132 | 1 | Ssr1 | Myofibroblast (Myo 1.1) |
| 0.324512 | 0.491 | 0.197 | 4.93E-132 | 1 | Ccdc80 | Myofibroblast (Myo 1.1) |
| 0.365206 | 0.782 | 0.463 | 7.05E-129 | 1 | Arf5 | Myofibroblast (Myo 1.1) |
| 0.424172 | 0.787 | 0.469 | 2.97E-125 | 1 | Hmgb1 | Myofibroblast (Myo 1.1) |
| 0.351248 | 0.785 | 0.45 | 9.42E-125 | 1 | Selenof | Myofibroblast (Myo 1.1) |
| 0.400908 | 0.862 | 0.542 | 9.44E-125 | 1 | Rpl22l1 | Myofibroblast (Myo 1.1) |
| 0.380217 | 0.707 | 0.377 | 1.87E-124 | 1 | Tubb5 | Myofibroblast (Myo 1.1) |
| 0.358538 | 0.552 | 0.258 | 8.07E-124 | 1 | Tmem258 | Myofibroblast (Myo 1.1) |
| 0.349499 | 0.835 | 0.474 | 1.77E-123 | 1 | Laptm4a | Myofibroblast (Myo 1.1) |
| 0.263874 | 0.29 | 0.087 | 2.28E-121 | 1 | Plat | Myofibroblast (Myo 1.1) |
| 0.370105 | 0.691 | 0.342 | 1.59E-120 | 1 | Nupr1 | Myofibroblast (Myo 1.1) |
| 0.280965 | 0.345 | 0.122 | 1.76E-113 | 1 | Fkbp9 | Myofibroblast (Myo 1.1) |
| 0.25805 | 0.221 | 0.056 | 5.64E-113 | 1 | Grem1 | Myofibroblast (Myo 1.1) |
| 0.286653 | 0.394 | 0.151 | 5.75E-113 | 1 | Vkorc1 | Myofibroblast (Myo 1.1) |
| 0.408404 | 0.96 | 0.77 | 5.03E-110 | 1 | Rps2 | Myofibroblast (Myo 1.1) |
| 0.254907 | 0.313 | 0.106 | 2.22E-108 | 1 | Pofut2 | Myofibroblast (Myo 1.1) |
| 0.340877 | 0.855 | 0.484 | 9.61E-108 | 1 | Timp2 | Myofibroblast (Myo 1.1) |
| 0.281701 | 0.461 | 0.199 | 1.16E-107 | 1 | Tmem167 | Myofibroblast (Myo 1.1) |
| 0.308493 | 0.652 | 0.345 | 2.62E-105 | 1 | Eef1g | Myofibroblast (Myo 1.1) |
| 0.279252 | 0.39 | 0.155 | 7.02E-105 | 1 | Sar1a | Myofibroblast (Myo 1.1) |
| 0.296989 | 0.571 | 0.286 | 7.98E-103 | 1 | Tmed2 | Myofibroblast (Myo 1.1) |
| 0.268503 | 0.376 | 0.148 | 2.21E-101 | 1 | Dap | Myofibroblast (Myo 1.1) |
| 0.314619 | 0.489 | 0.222 | 6.13E-100 | 1 | Tsc22d1 | Myofibroblast (Myo 1.1) |
| 0.256597 | 0.347 | 0.132 | 1.89E-99 | 1 | Ddost | Myofibroblast (Myo 1.1) |
| 0.285864 | 0.523 | 0.251 | 1.07E-98 | 1 | Pebp1 | Myofibroblast (Myo 1.1) |
| 0.313366 | 0.503 | 0.24 | 1.53E-97 | 1 | Tcf4 | Myofibroblast (Myo 1.1) |
| 0.265618 | 0.363 | 0.144 | 9.22E-95 | 1 | Sdc1 | Myofibroblast (Myo 1.1) |
| 0.270861 | 0.603 | 0.308 | 6.77E-91 | 1 | Prrx1 | Myofibroblast (Myo 1.1) |
| 0.288457 | 0.639 | 0.357 | 2.00E-90 | 1 | Prdx2 | Myofibroblast (Myo 1.1) |
| 0.254275 | 0.358 | 0.145 | 4.39E-89 | 1 | Col16a1 | Myofibroblast (Myo 1.1) |
| 0.260073 | 0.356 | 0.147 | 7.55E-88 | 1 | Snhg18 | Myofibroblast (Myo 1.1) |
| 0.276698 | 0.525 | 0.268 | 1.77E-85 | 1 | Hmgn1 | Myofibroblast (Myo 1.1) |
| 0.294287 | 0.801 | 0.5 | 4.11E-85 | 1 | Anxa5 | Myofibroblast (Myo 1.1) |
| 0.268132 | 0.467 | 0.223 | 4.51E-85 | 1 | Flna | Myofibroblast (Myo 1.1) |
| 0.316706 | 0.97 | 0.856 | 3.61E-82 | 1 | Actg1 | Myofibroblast (Myo 1.1) |
| 0.255086 | 0.336 | 0.139 | 1.07E-80 | 1 | Actn1 | Myofibroblast (Myo 1.1) |
| 0.295514 | 0.843 | 0.569 | 3.75E-80 | 1 | Npm1 | Myofibroblast (Myo 1.1) |
| 0.278532 | 0.417 | 0.19 | 6.99E-78 | 1 | Rbp1 | Myofibroblast (Myo 1.1) |
| 0.304939 | 0.785 | 0.551 | 1.01E-75 | 1 | Elob | Myofibroblast (Myo 1.1) |
| 0.262006 | 0.648 | 0.383 | 1.68E-75 | 1 | Ndufa4 | Myofibroblast (Myo 1.1) |
| 0.274466 | 0.689 | 0.423 | 1.36E-74 | 1 | Rps27l | Myofibroblast (Myo 1.1) |
| 0.259023 | 0.575 | 0.321 | 1.42E-74 | 1 | Atp5g1 | Myofibroblast (Myo 1.1) |
| 0.286421 | 0.985 | 0.848 | 2.65E-71 | 1 | Rpsa | Myofibroblast (Myo 1.1) |
| 0.272442 | 0.126 | 0.029 | 5.88E-71 | 1 | Igfbp2 | Myofibroblast (Myo 1.1) |
| 0.265384 | 0.826 | 0.604 | 9.77E-66 | 1 | Rbm3 | Myofibroblast (Myo 1.1) |
| 0.302321 | 0.338 | 0.156 | 3.02E-62 | 1 | Itm2a | Myofibroblast (Myo 1.1) |
| 0.253714 | 0.42 | 0.22 | 2.73E-60 | 1 | Myh9 | Myofibroblast (Myo 1.1) |
| 0.250398 | 0.895 | 0.672 | 1.61E-59 | 1 | Eef1b2 | Myofibroblast (Myo 1.1) |
| 0.250501 | 0.642 | 0.389 | 3.89E-57 | 1 | Jun | Myofibroblast (Myo 1.1) |
| 0.259837 | 0.986 | 0.859 | 8.75E-52 | 1 | Rplp0 | Myofibroblast (Myo 1.1) |
| 0.332938 | 0.946 | 0.783 | 6.12E-41 | 1 | Gm10076 | Myofibroblast (Myo 1.1) |
| 2.097999 | 0.853 | 0.24 | 0 | 2 | Mgp | Fibroblast (Fib 1.1) |
| 1.734296 | 0.978 | 0.373 | 0 | 2 | Dcn | Fibroblast (Fib 1.1) |
| 1.273151 | 0.911 | 0.384 | 0 | 2 | Igfbp4 | Fibroblast (Fib 1.1) |
| 1.243669 | 0.926 | 0.361 | 0 | 2 | Lum | Fibroblast (Fib 1.1) |
| 1.016247 | 0.853 | 0.335 | 0 | 2 | Mmp2 | Fibroblast (Fib 1.1) |
| 0.991439 | 0.584 | 0.143 | 0 | 2 | Mfap5 | Fibroblast (Fib 1.1) |
| 0.935715 | 0.906 | 0.396 | 0 | 2 | Fstl1 | Fibroblast (Fib 1.1) |
| 0.897111 | 0.713 | 0.241 | 0 | 2 | Serping1 | Fibroblast (Fib 1.1) |
| 0.791135 | 0.605 | 0.173 | 0 | 2 | Rbp1 | Fibroblast (Fib 1.1) |
| 0.756907 | 0.656 | 0.195 | 0 | 2 | Nbl1 | Fibroblast (Fib 1.1) |
| 0.664488 | 0.497 | 0.092 | 0 | 2 | Fbln1 | Fibroblast (Fib 1.1) |
| 0.732382 | 0.5 | 0.122 | 1.39E-300 | 2 | Cxcl12 | Fibroblast (Fib 1.1) |
| 0.504697 | 0.442 | 0.095 | 2.32E-299 | 2 | C1s1 | Fibroblast (Fib 1.1) |
| 1.19172 | 0.988 | 0.52 | 2.14E-292 | 2 | Col3a1 | Fibroblast (Fib 1.1) |
| 0.939422 | 0.986 | 0.491 | 1.26E-287 | 2 | Bgn | Fibroblast (Fib 1.1) |
| 0.405873 | 0.265 | 0.033 | 2.00E-285 | 2 | C4b | Fibroblast (Fib 1.1) |
| 0.393211 | 0.261 | 0.034 | 3.57E-269 | 2 | Il33 | Fibroblast (Fib 1.1) |
| 0.44323 | 0.335 | 0.06 | 2.52E-267 | 2 | Cygb | Fibroblast (Fib 1.1) |
| 0.461133 | 0.442 | 0.106 | 7.43E-262 | 2 | Bicc1 | Fibroblast (Fib 1.1) |
| 0.713614 | 0.506 | 0.141 | 9.71E-259 | 2 | Igf1 | Fibroblast (Fib 1.1) |
| 0.612825 | 0.53 | 0.16 | 1.08E-249 | 2 | Cpxm1 | Fibroblast (Fib 1.1) |
| 0.419894 | 0.376 | 0.08 | 2.36E-247 | 2 | Il11ra1 | Fibroblast (Fib 1.1) |
| 0.638891 | 0.476 | 0.131 | 5.08E-243 | 2 | Mfap4 | Fibroblast (Fib 1.1) |
| 0.689744 | 0.463 | 0.128 | 1.08E-233 | 2 | Igfbp6 | Fibroblast (Fib 1.1) |
| 0.700855 | 0.897 | 0.482 | 1.29E-224 | 2 | Timp2 | Fibroblast (Fib 1.1) |
| 0.681998 | 0.942 | 0.459 | 2.01E-211 | 2 | Col5a2 | Fibroblast (Fib 1.1) |
| 0.795092 | 0.362 | 0.087 | 3.57E-208 | 2 | Eln | Fibroblast (Fib 1.1) |
| 0.911951 | 0.408 | 0.114 | 5.00E-205 | 2 | Sfrp2 | Fibroblast (Fib 1.1) |
| 0.562284 | 0.869 | 0.384 | 1.24E-203 | 2 | Serpinf1 | Fibroblast (Fib 1.1) |
| 0.422864 | 0.751 | 0.324 | 2.54E-200 | 2 | Ctsk | Fibroblast (Fib 1.1) |
| 0.818175 | 0.794 | 0.425 | 4.85E-199 | 2 | Fosb | Fibroblast (Fib 1.1) |
| 0.511861 | 0.599 | 0.228 | 1.50E-196 | 2 | Cdh11 | Fibroblast (Fib 1.1) |
| 0.772832 | 0.994 | 0.529 | 1.05E-195 | 2 | Col1a2 | Fibroblast (Fib 1.1) |
| 0.473674 | 0.534 | 0.188 | 1.20E-192 | 2 | Mfap2 | Fibroblast (Fib 1.1) |
| 0.452315 | 0.504 | 0.169 | 1.31E-192 | 2 | Nfix | Fibroblast (Fib 1.1) |
| 0.713177 | 0.621 | 0.27 | 1.96E-186 | 2 | Klf4 | Fibroblast (Fib 1.1) |
| 0.816906 | 0.544 | 0.211 | 6.43E-185 | 2 | Mt2 | Fibroblast (Fib 1.1) |
| 0.543106 | 0.805 | 0.366 | 4.38E-182 | 2 | Aebp1 | Fibroblast (Fib 1.1) |
| 0.286718 | 0.171 | 0.021 | 8.37E-182 | 2 | Gdf10 | Fibroblast (Fib 1.1) |
| 0.33808 | 0.209 | 0.034 | 1.65E-176 | 2 | Has1 | Fibroblast (Fib 1.1) |
| 0.627442 | 0.896 | 0.469 | 6.66E-175 | 2 | Igfbp7 | Fibroblast (Fib 1.1) |
| 0.677174 | 0.99 | 0.518 | 2.92E-172 | 2 | Col1a1 | Fibroblast (Fib 1.1) |
| 0.485379 | 0.543 | 0.211 | 2.69E-168 | 2 | Klf9 | Fibroblast (Fib 1.1) |
| 0.731086 | 0.994 | 0.555 | 6.61E-168 | 2 | Sparc | Fibroblast (Fib 1.1) |
| 0.256961 | 0.223 | 0.041 | 1.33E-166 | 2 | Plxdc2 | Fibroblast (Fib 1.1) |
| 0.446053 | 0.508 | 0.192 | 1.27E-165 | 2 | Loxl1 | Fibroblast (Fib 1.1) |
| 0.553912 | 0.677 | 0.319 | 1.18E-164 | 2 | Fbn1 | Fibroblast (Fib 1.1) |
| 0.635923 | 0.167 | 0.023 | 9.05E-162 | 2 | Mmp3 | Fibroblast (Fib 1.1) |
| 0.960241 | 0.763 | 0.446 | 3.35E-158 | 2 | Mt1 | Fibroblast (Fib 1.1) |
| 0.446226 | 0.737 | 0.376 | 1.61E-157 | 2 | Nedd4 | Fibroblast (Fib 1.1) |
| 0.411414 | 0.504 | 0.187 | 1.99E-157 | 2 | Zbtb20 | Fibroblast (Fib 1.1) |
| 0.47771 | 0.648 | 0.305 | 2.06E-154 | 2 | Prrx1 | Fibroblast (Fib 1.1) |
| 0.401002 | 0.371 | 0.113 | 3.82E-153 | 2 | Mdk | Fibroblast (Fib 1.1) |
| 0.333412 | 0.327 | 0.091 | 2.45E-152 | 2 | Nfib | Fibroblast (Fib 1.1) |
| 0.480131 | 0.419 | 0.141 | 3.63E-150 | 2 | Ogn | Fibroblast (Fib 1.1) |
| 0.34145 | 0.182 | 0.03 | 7.83E-147 | 2 | Clec3b | Fibroblast (Fib 1.1) |
| 0.39004 | 0.301 | 0.082 | 1.52E-143 | 2 | Gas6 | Fibroblast (Fib 1.1) |
| 0.311802 | 0.338 | 0.097 | 3.96E-142 | 2 | Plpp3 | Fibroblast (Fib 1.1) |
| 1.202227 | 0.262 | 0.063 | 2.38E-141 | 2 | Apod | Fibroblast (Fib 1.1) |
| 1.017828 | 0.403 | 0.141 | 3.26E-141 | 2 | Igfbp5 | Fibroblast (Fib 1.1) |
| 0.529184 | 0.389 | 0.135 | 8.24E-138 | 2 | Sfrp1 | Fibroblast (Fib 1.1) |
| 0.337011 | 0.222 | 0.049 | 3.65E-137 | 2 | Ugdh | Fibroblast (Fib 1.1) |
| 0.486304 | 0.87 | 0.439 | 2.73E-135 | 2 | Col6a1 | Fibroblast (Fib 1.1) |
| 0.628424 | 0.678 | 0.345 | 6.35E-135 | 2 | Nupr1 | Fibroblast (Fib 1.1) |
| 0.514496 | 0.346 | 0.111 | 1.29E-132 | 2 | Ly6a | Fibroblast (Fib 1.1) |
| 0.475209 | 0.408 | 0.152 | 3.62E-132 | 2 | Hk2 | Fibroblast (Fib 1.1) |
| 0.430597 | 0.417 | 0.149 | 6.47E-132 | 2 | Itm2a | Fibroblast (Fib 1.1) |
| 0.403286 | 0.728 | 0.382 | 1.26E-131 | 2 | Cd81 | Fibroblast (Fib 1.1) |
| 0.422983 | 0.675 | 0.339 | 8.86E-131 | 2 | Lrp1 | Fibroblast (Fib 1.1) |
| 0.32517 | 0.225 | 0.052 | 3.15E-129 | 2 | Col14a1 | Fibroblast (Fib 1.1) |
| 0.418501 | 0.522 | 0.227 | 1.43E-127 | 2 | Mxra8 | Fibroblast (Fib 1.1) |
| 0.28397 | 0.3 | 0.087 | 1.68E-127 | 2 | Colec12 | Fibroblast (Fib 1.1) |
| 0.347169 | 0.416 | 0.154 | 4.15E-127 | 2 | Pdgfra | Fibroblast (Fib 1.1) |
| 0.452385 | 0.559 | 0.255 | 1.05E-126 | 2 | Lox | Fibroblast (Fib 1.1) |
| 0.548355 | 0.887 | 0.523 | 1.44E-125 | 2 | Fn1 | Fibroblast (Fib 1.1) |
| 0.364348 | 0.214 | 0.049 | 9.94E-124 | 2 | Ccl8 | Fibroblast (Fib 1.1) |
| 0.392192 | 0.737 | 0.385 | 7.44E-121 | 2 | Ppic | Fibroblast (Fib 1.1) |
| 0.342727 | 0.45 | 0.181 | 1.34E-118 | 2 | Fgfr1 | Fibroblast (Fib 1.1) |
| 0.453013 | 0.786 | 0.406 | 1.68E-118 | 2 | Col6a2 | Fibroblast (Fib 1.1) |
| 0.332617 | 0.239 | 0.062 | 2.73E-118 | 2 | Cfh | Fibroblast (Fib 1.1) |
| 0.276477 | 0.171 | 0.034 | 5.17E-114 | 2 | Ccl11 | Fibroblast (Fib 1.1) |
| 0.3961 | 0.754 | 0.462 | 1.73E-110 | 2 | Gas5 | Fibroblast (Fib 1.1) |
| 0.369147 | 0.808 | 0.479 | 1.29E-108 | 2 | Laptm4a | Fibroblast (Fib 1.1) |
| 0.478202 | 0.637 | 0.347 | 2.08E-108 | 2 | Mmp14 | Fibroblast (Fib 1.1) |
| 0.263766 | 0.246 | 0.069 | 2.06E-107 | 2 | Vasn | Fibroblast (Fib 1.1) |
| 0.411545 | 0.195 | 0.048 | 7.95E-104 | 2 | Ptx3 | Fibroblast (Fib 1.1) |
| 0.793106 | 0.546 | 0.297 | 7.10E-102 | 2 | Gsn | Fibroblast (Fib 1.1) |
| 0.284331 | 0.349 | 0.13 | 2.77E-101 | 2 | Rnase4 | Fibroblast (Fib 1.1) |
| 0.442786 | 0.729 | 0.422 | 1.65E-100 | 2 | Pmepa1 | Fibroblast (Fib 1.1) |
| 0.342286 | 0.455 | 0.202 | 2.24E-100 | 2 | Ccdc80 | Fibroblast (Fib 1.1) |
| 0.273148 | 0.699 | 0.398 | 2.13E-98 | 2 | Ctsl | Fibroblast (Fib 1.1) |
| 0.443415 | 0.611 | 0.341 | 4.43E-97 | 2 | Emp1 | Fibroblast (Fib 1.1) |
| 0.385971 | 0.288 | 0.099 | 1.12E-95 | 2 | Vcam1 | Fibroblast (Fib 1.1) |
| 0.373491 | 0.396 | 0.168 | 6.15E-94 | 2 | Gas1 | Fibroblast (Fib 1.1) |
| 0.345361 | 0.775 | 0.416 | 1.06E-88 | 2 | Col6a3 | Fibroblast (Fib 1.1) |
| 0.263351 | 0.288 | 0.102 | 8.16E-88 | 2 | Rarres2 | Fibroblast (Fib 1.1) |
| 0.306928 | 0.246 | 0.08 | 1.40E-86 | 2 | Uap1 | Fibroblast (Fib 1.1) |
| 0.528982 | 0.613 | 0.337 | 6.74E-86 | 2 | Timp1 | Fibroblast (Fib 1.1) |
| 0.263442 | 0.298 | 0.111 | 5.39E-84 | 2 | Nid1 | Fibroblast (Fib 1.1) |
| 0.281341 | 0.48 | 0.234 | 6.53E-84 | 2 | Nenf | Fibroblast (Fib 1.1) |
| 0.251476 | 0.262 | 0.088 | 7.97E-83 | 2 | Angptl1 | Fibroblast (Fib 1.1) |
| 0.28483 | 0.446 | 0.212 | 2.73E-81 | 2 | Serpinb6a | Fibroblast (Fib 1.1) |
| 0.365968 | 0.233 | 0.076 | 3.27E-81 | 2 | C3 | Fibroblast (Fib 1.1) |
| 0.30742 | 0.528 | 0.261 | 6.08E-79 | 2 | Col4a1 | Fibroblast (Fib 1.1) |
| 0.254203 | 0.891 | 0.47 | 2.32E-78 | 2 | Serpinh1 | Fibroblast (Fib 1.1) |
| 0.265236 | 0.187 | 0.053 | 6.71E-78 | 2 | Dpt | Fibroblast (Fib 1.1) |
| 0.277707 | 0.786 | 0.432 | 2.93E-77 | 2 | Postn | Fibroblast (Fib 1.1) |
| 0.338479 | 0.25 | 0.088 | 5.83E-76 | 2 | Tnfaip6 | Fibroblast (Fib 1.1) |
| 0.258057 | 0.325 | 0.134 | 8.78E-75 | 2 | Sptbn1 | Fibroblast (Fib 1.1) |
| 0.448447 | 0.448 | 0.231 | 2.98E-74 | 2 | Errfi1 | Fibroblast (Fib 1.1) |
| 0.357168 | 0.4 | 0.19 | 7.39E-74 | 2 | Nr4a2 | Fibroblast (Fib 1.1) |
| 0.297073 | 0.442 | 0.225 | 1.80E-72 | 2 | Mtch1 | Fibroblast (Fib 1.1) |
| 0.394729 | 0.476 | 0.24 | 1.06E-71 | 2 | Meg3 | Fibroblast (Fib 1.1) |
| 0.365903 | 0.71 | 0.467 | 1.59E-67 | 2 | Ahnak | Fibroblast (Fib 1.1) |
| 0.275503 | 0.605 | 0.338 | 1.66E-67 | 2 | Pcolce | Fibroblast (Fib 1.1) |
| 0.408897 | 0.309 | 0.128 | 5.77E-67 | 2 | Cxcl5 | Fibroblast (Fib 1.1) |
| 0.285218 | 0.489 | 0.266 | 1.06E-65 | 2 | 2410006H16Rik | Fibroblast (Fib 1.1) |
| 0.263364 | 0.429 | 0.207 | 1.26E-65 | 2 | Col4a2 | Fibroblast (Fib 1.1) |
| 0.329771 | 0.5 | 0.271 | 3.71E-65 | 2 | Cebpd | Fibroblast (Fib 1.1) |
| 0.420363 | 0.637 | 0.391 | 1.09E-63 | 2 | Jun | Fibroblast (Fib 1.1) |
| 0.270435 | 0.393 | 0.191 | 4.41E-63 | 2 | Gem | Fibroblast (Fib 1.1) |
| 0.387512 | 0.823 | 0.621 | 4.38E-62 | 2 | Lmna | Fibroblast (Fib 1.1) |
| 0.360783 | 0.201 | 0.071 | 2.29E-59 | 2 | Ccn2 | Fibroblast (Fib 1.1) |
| 0.499549 | 0.294 | 0.132 | 1.69E-57 | 2 | Igfbp3 | Fibroblast (Fib 1.1) |
| 0.366581 | 0.559 | 0.342 | 4.78E-57 | 2 | Zfp36l1 | Fibroblast (Fib 1.1) |
| 0.252776 | 0.613 | 0.365 | 2.12E-56 | 2 | Selenom | Fibroblast (Fib 1.1) |
| 0.330044 | 0.426 | 0.242 | 4.64E-49 | 2 | Ecm1 | Fibroblast (Fib 1.1) |
| 0.255222 | 0.251 | 0.111 | 4.85E-48 | 2 | Ccn5 | Fibroblast (Fib 1.1) |
| 0.306964 | 0.98 | 0.863 | 8.34E-48 | 2 | Rps20 | Fibroblast (Fib 1.1) |
| 0.297154 | 0.989 | 0.909 | 1.23E-46 | 2 | Rps8 | Fibroblast (Fib 1.1) |
| 0.398022 | 0.489 | 0.303 | 1.29E-46 | 2 | Egr1 | Fibroblast (Fib 1.1) |
| 0.329517 | 0.26 | 0.119 | 2.63E-46 | 2 | Timp3 | Fibroblast (Fib 1.1) |
| 0.270358 | 0.867 | 0.615 | 4.77E-46 | 2 | Gnas | Fibroblast (Fib 1.1) |
| 0.262399 | 0.989 | 0.903 | 1.85E-45 | 2 | Rps28 | Fibroblast (Fib 1.1) |
| 0.268869 | 0.408 | 0.231 | 4.86E-45 | 2 | Tsc22d1 | Fibroblast (Fib 1.1) |
| 0.262166 | 0.965 | 0.879 | 1.22E-41 | 2 | Rps3a1 | Fibroblast (Fib 1.1) |
| 0.264037 | 0.255 | 0.12 | 2.84E-41 | 2 | Aqp1 | Fibroblast (Fib 1.1) |
| 0.38983 | 0.589 | 0.429 | 9.32E-41 | 2 | Neat1 | Fibroblast (Fib 1.1) |
| 0.279439 | 0.528 | 0.332 | 9.25E-40 | 2 | Aspn | Fibroblast (Fib 1.1) |
| 0.257615 | 0.952 | 0.855 | 1.50E-38 | 2 | Rpl28 | Fibroblast (Fib 1.1) |
| 0.31902 | 0.841 | 0.738 | 1.01E-37 | 2 | Ubc | Fibroblast (Fib 1.1) |
| 0.327925 | 0.358 | 0.211 | 7.48E-37 | 2 | Sgk1 | Fibroblast (Fib 1.1) |
| 0.277486 | 0.182 | 0.076 | 2.89E-36 | 2 | Il6 | Fibroblast (Fib 1.1) |
| 0.260678 | 0.996 | 0.922 | 7.12E-34 | 2 | Rpl37a | Fibroblast (Fib 1.1) |
| 0.258919 | 0.98 | 0.9 | 3.54E-31 | 2 | Rpl39 | Fibroblast (Fib 1.1) |
| 0.273235 | 0.772 | 0.668 | 7.74E-31 | 2 | Jund | Fibroblast (Fib 1.1) |
| 0.251186 | 0.199 | 0.098 | 2.32E-26 | 2 | Mmp13 | Fibroblast (Fib 1.1) |
| 0.255711 | 0.416 | 0.269 | 2.51E-26 | 2 | Klf2 | Fibroblast (Fib 1.1) |
| 0.266451 | 0.872 | 0.791 | 4.11E-13 | 2 | Gm10076 | Fibroblast (Fib 1.1) |
| 3.401984 | 0.899 | 0.03 | 0 | 3 | Rgs5 | Pericytes (P 1.1) |
| 2.568103 | 0.962 | 0.243 | 0 | 3 | Mgp | Pericytes (P 1.1) |
| 2.068941 | 0.974 | 0.472 | 0 | 3 | Igfbp7 | Pericytes (P 1.1) |
| 1.936555 | 0.83 | 0.273 | 0 | 3 | Acta2 | Pericytes (P 1.1) |
| 1.913797 | 0.929 | 0.236 | 0 | 3 | Col4a1 | Pericytes (P 1.1) |
| 1.576816 | 0.726 | 0.044 | 0 | 3 | Gm13889 | Pericytes (P 1.1) |
| 1.462938 | 0.775 | 0.097 | 0 | 3 | Sparcl1 | Pericytes (P 1.1) |
| 1.421049 | 0.747 | 0.181 | 0 | 3 | Tagln | Pericytes (P 1.1) |
| 1.307336 | 0.873 | 0.178 | 0 | 3 | Col4a2 | Pericytes (P 1.1) |
| 1.297535 | 0.839 | 0.199 | 0 | 3 | Serpine2 | Pericytes (P 1.1) |
| 1.204068 | 0.961 | 0.436 | 0 | 3 | Cald1 | Pericytes (P 1.1) |
| 1.051664 | 0.64 | 0.03 | 0 | 3 | Ndufa4l2 | Pericytes (P 1.1) |
| 1.011016 | 0.672 | 0.088 | 0 | 3 | Gng11 | Pericytes (P 1.1) |
| 1.004472 | 0.775 | 0.161 | 0 | 3 | Ebf1 | Pericytes (P 1.1) |
| 0.976979 | 0.795 | 0.187 | 0 | 3 | Col18a1 | Pericytes (P 1.1) |
| 0.941906 | 0.54 | 0.05 | 0 | 3 | Mustn1 | Pericytes (P 1.1) |
| 0.917996 | 0.632 | 0.164 | 0 | 3 | Myl9 | Pericytes (P 1.1) |
| 0.821804 | 0.508 | 0.087 | 0 | 3 | Hspb1 | Pericytes (P 1.1) |
| 0.812566 | 0.679 | 0.194 | 0 | 3 | Tm4sf1 | Pericytes (P 1.1) |
| 0.808015 | 0.58 | 0.038 | 0 | 3 | Itga1 | Pericytes (P 1.1) |
| 0.781827 | 0.566 | 0.025 | 0 | 3 | Notch3 | Pericytes (P 1.1) |
| 0.774148 | 0.577 | 0.058 | 0 | 3 | Mylk | Pericytes (P 1.1) |
| 0.752672 | 0.534 | 0.061 | 0 | 3 | Col15a1 | Pericytes (P 1.1) |
| 0.742697 | 0.37 | 0.024 | 0 | 3 | Rgs16 | Pericytes (P 1.1) |
| 0.717192 | 0.62 | 0.132 | 0 | 3 | Prss23 | Pericytes (P 1.1) |
| 0.703307 | 0.621 | 0.114 | 0 | 3 | Pdgfrb | Pericytes (P 1.1) |
| 0.667369 | 0.618 | 0.12 | 0 | 3 | Crip2 | Pericytes (P 1.1) |
| 0.663108 | 0.549 | 0.096 | 0 | 3 | Pdgfa | Pericytes (P 1.1) |
| 0.646133 | 0.46 | 0.021 | 0 | 3 | Gjc1 | Pericytes (P 1.1) |
| 0.643295 | 0.641 | 0.175 | 0 | 3 | Mfge8 | Pericytes (P 1.1) |
| 0.627985 | 0.372 | 0.009 | 0 | 3 | Higd1b | Pericytes (P 1.1) |
| 0.611338 | 0.341 | 0.012 | 0 | 3 | Cox4i2 | Pericytes (P 1.1) |
| 0.546996 | 0.382 | 0.059 | 0 | 3 | Filip1l | Pericytes (P 1.1) |
| 0.489787 | 0.425 | 0.056 | 0 | 3 | Mprip | Pericytes (P 1.1) |
| 0.485682 | 0.379 | 0.041 | 0 | 3 | Mgst3 | Pericytes (P 1.1) |
| 0.481093 | 0.264 | 0.019 | 0 | 3 | Actg2 | Pericytes (P 1.1) |
| 0.476999 | 0.342 | 0.017 | 0 | 3 | Des | Pericytes (P 1.1) |
| 0.456757 | 0.337 | 0.024 | 0 | 3 | Mcam | Pericytes (P 1.1) |
| 0.451925 | 0.328 | 0.034 | 0 | 3 | Nr2f2 | Pericytes (P 1.1) |
| 0.398648 | 0.273 | 0.023 | 0 | 3 | Epas1 | Pericytes (P 1.1) |
| 0.390483 | 0.32 | 0.036 | 0 | 3 | Pdlim1 | Pericytes (P 1.1) |
| 0.390382 | 0.288 | 0.029 | 0 | 3 | Ptp4a3 | Pericytes (P 1.1) |
| 0.369539 | 0.264 | 0.018 | 0 | 3 | Tinagl1 | Pericytes (P 1.1) |
| 0.349456 | 0.233 | 0.016 | 0 | 3 | Abcc9 | Pericytes (P 1.1) |
| 0.342208 | 0.24 | 0.008 | 0 | 3 | 04-Sep | Pericytes (P 1.1) |
| 0.330666 | 0.21 | 0.011 | 0 | 3 | Adra2a | Pericytes (P 1.1) |
| 0.326407 | 0.227 | 0.014 | 0 | 3 | Gucy1a1 | Pericytes (P 1.1) |
| 0.279081 | 0.203 | 0.007 | 0 | 3 | Esam | Pericytes (P 1.1) |
| 0.277715 | 0.186 | 0.01 | 0 | 3 | Apold1 | Pericytes (P 1.1) |
| 0.272113 | 0.198 | 0.013 | 2.30E-300 | 3 | Parm1 | Pericytes (P 1.1) |
| 0.50238 | 0.522 | 0.116 | 6.10E-295 | 3 | Lhfp | Pericytes (P 1.1) |
| 1.372991 | 0.985 | 0.758 | 4.62E-293 | 3 | Crip1 | Pericytes (P 1.1) |
| 0.302436 | 0.204 | 0.015 | 9.88E-280 | 3 | Bcr | Pericytes (P 1.1) |
| 0.275854 | 0.19 | 0.013 | 6.45E-277 | 3 | Ppp1r14a | Pericytes (P 1.1) |
| 0.401603 | 0.341 | 0.051 | 1.10E-275 | 3 | Cd248 | Pericytes (P 1.1) |
| 0.36169 | 0.277 | 0.035 | 4.01E-261 | 3 | Rbpms | Pericytes (P 1.1) |
| 0.356888 | 0.316 | 0.047 | 1.43E-259 | 3 | Ehd2 | Pericytes (P 1.1) |
| 0.840754 | 0.716 | 0.254 | 4.78E-259 | 3 | Tpm2 | Pericytes (P 1.1) |
| 0.377475 | 0.327 | 0.053 | 3.52E-247 | 3 | Rhoj | Pericytes (P 1.1) |
| 0.616407 | 0.636 | 0.198 | 2.20E-239 | 3 | Col5a3 | Pericytes (P 1.1) |
| 0.885148 | 0.957 | 0.614 | 5.35E-239 | 3 | Ifitm3 | Pericytes (P 1.1) |
| 0.261948 | 0.19 | 0.016 | 1.49E-236 | 3 | Pde3a | Pericytes (P 1.1) |
| 0.329489 | 0.211 | 0.021 | 5.54E-231 | 3 | Myh11 | Pericytes (P 1.1) |
| 0.376508 | 0.346 | 0.064 | 4.86E-221 | 3 | Cygb | Pericytes (P 1.1) |
| 0.866768 | 0.654 | 0.253 | 2.24E-212 | 3 | Phlda1 | Pericytes (P 1.1) |
| 0.395061 | 0.208 | 0.024 | 1.37E-206 | 3 | Sncg | Pericytes (P 1.1) |
| 0.260455 | 0.219 | 0.028 | 1.06E-201 | 3 | Dbndd2 | Pericytes (P 1.1) |
| 0.278353 | 0.216 | 0.027 | 9.35E-201 | 3 | Ednra | Pericytes (P 1.1) |
| 0.267875 | 0.203 | 0.024 | 1.49E-196 | 3 | Myo1b | Pericytes (P 1.1) |
| 0.844058 | 0.766 | 0.356 | 1.52E-191 | 3 | Tpm1 | Pericytes (P 1.1) |
| 1.016936 | 0.709 | 0.318 | 7.72E-191 | 3 | Id3 | Pericytes (P 1.1) |
| 0.467006 | 0.46 | 0.131 | 5.07E-187 | 3 | Adamts2 | Pericytes (P 1.1) |
| 0.326853 | 0.302 | 0.059 | 2.11E-185 | 3 | Rras | Pericytes (P 1.1) |
| 0.29312 | 0.328 | 0.066 | 6.51E-182 | 3 | Cryab | Pericytes (P 1.1) |
| 0.45713 | 0.272 | 0.051 | 2.06E-173 | 3 | Adamts4 | Pericytes (P 1.1) |
| 0.668775 | 0.307 | 0.068 | 3.10E-160 | 3 | Il6 | Pericytes (P 1.1) |
| 0.535249 | 0.368 | 0.097 | 3.83E-160 | 3 | Adamts1 | Pericytes (P 1.1) |
| 0.384009 | 0.421 | 0.123 | 1.30E-159 | 3 | Fermt2 | Pericytes (P 1.1) |
| 0.407711 | 0.52 | 0.18 | 1.21E-155 | 3 | Rhoc | Pericytes (P 1.1) |
| 0.424165 | 0.464 | 0.152 | 2.47E-152 | 3 | Pten | Pericytes (P 1.1) |
| 0.558679 | 0.637 | 0.256 | 1.47E-150 | 3 | Serping1 | Pericytes (P 1.1) |
| 0.726842 | 0.826 | 0.528 | 2.16E-150 | 3 | Anxa1 | Pericytes (P 1.1) |
| 0.451497 | 0.551 | 0.219 | 1.86E-142 | 3 | 07-Sep | Pericytes (P 1.1) |
| 0.537309 | 0.743 | 0.391 | 4.30E-140 | 3 | Itgb1 | Pericytes (P 1.1) |
| 0.356477 | 0.386 | 0.121 | 1.26E-130 | 3 | Eva1b | Pericytes (P 1.1) |
| 0.409087 | 0.506 | 0.194 | 1.38E-129 | 3 | Cyb5r3 | Pericytes (P 1.1) |
| 0.326926 | 0.327 | 0.088 | 1.76E-129 | 3 | Mef2c | Pericytes (P 1.1) |
| 0.732465 | 0.98 | 0.823 | 6.40E-128 | 3 | Vim | Pericytes (P 1.1) |
| 0.375062 | 0.307 | 0.086 | 2.11E-120 | 3 | Tcim | Pericytes (P 1.1) |
| 0.418654 | 0.326 | 0.096 | 5.01E-120 | 3 | Ddit4 | Pericytes (P 1.1) |
| 0.44551 | 0.737 | 0.388 | 1.48E-119 | 3 | Cd81 | Pericytes (P 1.1) |
| 0.282993 | 0.259 | 0.064 | 2.93E-115 | 3 | Vcl | Pericytes (P 1.1) |
| 0.438182 | 0.533 | 0.231 | 1.16E-114 | 3 | Cavin3 | Pericytes (P 1.1) |
| 0.299475 | 0.297 | 0.085 | 1.75E-109 | 3 | Lamc1 | Pericytes (P 1.1) |
| 0.38155 | 0.397 | 0.142 | 8.98E-109 | 3 | Csrp1 | Pericytes (P 1.1) |
| 0.452926 | 0.799 | 0.486 | 3.01E-108 | 3 | Laptm4a | Pericytes (P 1.1) |
| 0.471519 | 0.795 | 0.421 | 6.74E-101 | 3 | Col6a3 | Pericytes (P 1.1) |
| 0.53147 | 0.913 | 0.753 | 4.46E-99 | 3 | Itm2b | Pericytes (P 1.1) |
| 0.281401 | 0.217 | 0.053 | 2.04E-97 | 3 | Tppp3 | Pericytes (P 1.1) |
| 0.519581 | 0.967 | 0.864 | 4.55E-97 | 3 | mt-Cytb | Pericytes (P 1.1) |
| 0.318412 | 0.379 | 0.137 | 1.70E-96 | 3 | Cav1 | Pericytes (P 1.1) |
| 0.356259 | 0.489 | 0.208 | 1.69E-95 | 3 | Nudt4 | Pericytes (P 1.1) |
| 0.410452 | 0.688 | 0.388 | 3.65E-94 | 3 | Ptms | Pericytes (P 1.1) |
| 0.548241 | 0.908 | 0.817 | 1.53E-92 | 3 | Myl6 | Pericytes (P 1.1) |
| 0.458776 | 0.97 | 0.834 | 2.47E-91 | 3 | Ptma | Pericytes (P 1.1) |
| 0.380213 | 0.744 | 0.461 | 2.37E-86 | 3 | Selenof | Pericytes (P 1.1) |
| 0.26213 | 0.304 | 0.101 | 4.20E-86 | 3 | Sdc2 | Pericytes (P 1.1) |
| 0.483712 | 0.943 | 0.833 | 2.38E-85 | 3 | Ppia | Pericytes (P 1.1) |
| 0.372494 | 0.427 | 0.182 | 2.47E-84 | 3 | Arid5b | Pericytes (P 1.1) |
| 0.320841 | 0.416 | 0.176 | 8.33E-82 | 3 | Map1lc3a | Pericytes (P 1.1) |
| 0.431987 | 0.827 | 0.662 | 1.07E-81 | 3 | Cox8a | Pericytes (P 1.1) |
| 0.304802 | 0.319 | 0.118 | 5.95E-78 | 3 | Ccnd2 | Pericytes (P 1.1) |
| 0.517593 | 0.876 | 0.703 | 3.73E-77 | 3 | Nme2 | Pericytes (P 1.1) |
| 0.410016 | 0.588 | 0.319 | 5.11E-76 | 3 | Tuba1a | Pericytes (P 1.1) |
| 0.360779 | 0.868 | 0.695 | 6.44E-71 | 3 | Naca | Pericytes (P 1.1) |
| 0.335387 | 0.698 | 0.421 | 9.28E-71 | 3 | Slc25a4 | Pericytes (P 1.1) |
| 0.256562 | 0.305 | 0.114 | 1.32E-70 | 3 | Nid1 | Pericytes (P 1.1) |
| 0.511542 | 0.485 | 0.244 | 1.34E-70 | 3 | Meg3 | Pericytes (P 1.1) |
| 0.347491 | 0.467 | 0.229 | 2.11E-69 | 3 | Tmem176b | Pericytes (P 1.1) |
| 0.388609 | 0.643 | 0.4 | 3.23E-67 | 3 | Tpm4 | Pericytes (P 1.1) |
| 0.349092 | 0.873 | 0.62 | 9.43E-67 | 3 | Gnas | Pericytes (P 1.1) |
| 0.400928 | 0.981 | 0.922 | 1.99E-66 | 3 | mt-Atp6 | Pericytes (P 1.1) |
| 0.596821 | 0.764 | 0.524 | 1.35E-65 | 3 | S100a4 | Pericytes (P 1.1) |
| 0.330839 | 0.695 | 0.449 | 6.80E-62 | 3 | Selenow | Pericytes (P 1.1) |
| 0.322017 | 0.57 | 0.317 | 4.93E-61 | 3 | Prrx1 | Pericytes (P 1.1) |
| 0.263817 | 0.801 | 0.509 | 4.01E-59 | 3 | Cd9 | Pericytes (P 1.1) |
| 0.438963 | 0.94 | 0.862 | 6.21E-59 | 3 | Ubb | Pericytes (P 1.1) |
| 0.303703 | 0.62 | 0.376 | 6.46E-59 | 3 | Atp5d | Pericytes (P 1.1) |
| 0.342752 | 0.498 | 0.27 | 5.17E-58 | 3 | Dstn | Pericytes (P 1.1) |
| 0.339086 | 0.951 | 0.818 | 6.06E-56 | 3 | Rpl15 | Pericytes (P 1.1) |
| 0.455239 | 0.886 | 0.769 | 9.78E-55 | 3 | Hspa8 | Pericytes (P 1.1) |
| 0.313189 | 0.872 | 0.722 | 1.33E-54 | 3 | Calm1 | Pericytes (P 1.1) |
| 0.262597 | 0.26 | 0.105 | 2.48E-50 | 3 | Cpe | Pericytes (P 1.1) |
| 0.302668 | 0.623 | 0.391 | 6.74E-49 | 3 | Nedd4 | Pericytes (P 1.1) |
| 0.267504 | 0.542 | 0.319 | 1.50E-48 | 3 | Slc25a5 | Pericytes (P 1.1) |
| 0.254777 | 0.668 | 0.397 | 2.58E-48 | 3 | Ppic | Pericytes (P 1.1) |
| 0.295699 | 0.726 | 0.519 | 3.07E-48 | 3 | Uqcrh | Pericytes (P 1.1) |
| 0.500901 | 0.955 | 0.845 | 7.64E-43 | 3 | S100a6 | Pericytes (P 1.1) |
| 0.313496 | 0.681 | 0.485 | 4.59E-42 | 3 | Hmgb1 | Pericytes (P 1.1) |
| 0.33101 | 0.597 | 0.394 | 5.13E-42 | 3 | Tubb5 | Pericytes (P 1.1) |
| 0.276545 | 0.788 | 0.624 | 5.52E-42 | 3 | Chchd2 | Pericytes (P 1.1) |
| 0.270097 | 0.357 | 0.187 | 8.80E-41 | 3 | Tmem176a | Pericytes (P 1.1) |
| 0.260494 | 0.584 | 0.387 | 8.54E-40 | 3 | Atp5b | Pericytes (P 1.1) |
| 0.28612 | 0.957 | 0.842 | 1.17E-39 | 3 | Rpl24 | Pericytes (P 1.1) |
| 0.275871 | 0.753 | 0.579 | 1.74E-39 | 3 | Cox6c | Pericytes (P 1.1) |
| 0.301039 | 0.721 | 0.551 | 1.94E-39 | 3 | Dynll1 | Pericytes (P 1.1) |
| 0.314971 | 0.919 | 0.778 | 7.32E-39 | 3 | Rps2 | Pericytes (P 1.1) |
| 0.289805 | 0.707 | 0.517 | 7.82E-39 | 3 | Tagln2 | Pericytes (P 1.1) |
| 0.388879 | 0.6 | 0.392 | 5.24E-37 | 3 | Cxcl1 | Pericytes (P 1.1) |
| 0.269802 | 0.265 | 0.121 | 1.58E-36 | 3 | Timp3 | Pericytes (P 1.1) |
| 0.281407 | 0.713 | 0.535 | 4.75E-35 | 3 | Rpl29 | Pericytes (P 1.1) |
| 0.26976 | 0.859 | 0.682 | 6.06E-35 | 3 | Rpl36a | Pericytes (P 1.1) |
| 0.255557 | 0.534 | 0.353 | 2.30E-34 | 3 | Cox7a2 | Pericytes (P 1.1) |
| 0.251062 | 0.869 | 0.745 | 2.24E-33 | 3 | Rpl7a | Pericytes (P 1.1) |
| 0.257962 | 0.976 | 0.924 | 5.58E-33 | 3 | mt-Co2 | Pericytes (P 1.1) |
| 0.274108 | 0.979 | 0.925 | 9.33E-33 | 3 | mt-Co3 | Pericytes (P 1.1) |
| 0.276476 | 0.983 | 0.901 | 1.79E-32 | 3 | Rpl39 | Pericytes (P 1.1) |
| 0.256938 | 0.698 | 0.539 | 5.02E-32 | 3 | Myl12a | Pericytes (P 1.1) |
| 0.255748 | 0.903 | 0.798 | 5.32E-32 | 3 | mt-Nd4 | Pericytes (P 1.1) |
| 0.278036 | 0.92 | 0.792 | 1.00E-27 | 3 | Rpl35 | Pericytes (P 1.1) |
| 0.282341 | 0.295 | 0.167 | 1.50E-24 | 3 | Atf3 | Pericytes (P 1.1) |
| 0.386687 | 0.403 | 0.283 | 5.34E-17 | 3 | Cebpd | Pericytes (P 1.1) |
| 0.284255 | 0.325 | 0.207 | 1.42E-16 | 3 | Ccl2 | Pericytes (P 1.1) |
| 0.287789 | 0.467 | 0.353 | 6.94E-14 | 3 | Zfp36l1 | Pericytes (P 1.1) |
| 0.389694 | 0.963 | 0.974 | 3.72E-10 | 3 | Malat1 | Pericytes (P 1.1) |
| 0.257763 | 0.544 | 0.469 | 0.010223 | 3 | Mt1 | Pericytes (P 1.1) |
| 1.658257 | 0.997 | 0.53 | 0 | 4 | Col3a1 | Fibroblast (Fib 1.2) |
| 1.625624 | 0.998 | 0.539 | 0 | 4 | Col1a2 | Fibroblast (Fib 1.2) |
| 1.604671 | 0.989 | 0.426 | 0 | 4 | Postn | Fibroblast (Fib 1.2) |
| 1.579948 | 0.996 | 0.529 | 0 | 4 | Col1a1 | Fibroblast (Fib 1.2) |
| 1.383134 | 0.998 | 0.565 | 0 | 4 | Sparc | Fibroblast (Fib 1.2) |
| 1.341582 | 0.589 | 0.112 | 0 | 4 | Cxcl5 | Fibroblast (Fib 1.2) |
| 1.043476 | 0.989 | 0.467 | 0 | 4 | Col5a2 | Fibroblast (Fib 1.2) |
| 1.033725 | 0.97 | 0.403 | 0 | 4 | Fstl1 | Fibroblast (Fib 1.2) |
| 0.909062 | 0.966 | 0.386 | 0 | 4 | Col5a1 | Fibroblast (Fib 1.2) |
| 1.077736 | 0.992 | 0.502 | 1.01E-302 | 4 | Bgn | Fibroblast (Fib 1.2) |
| 0.852609 | 0.938 | 0.366 | 3.36E-302 | 4 | Aebp1 | Fibroblast (Fib 1.2) |
| 0.750244 | 0.547 | 0.127 | 9.92E-293 | 4 | Cxcl12 | Fibroblast (Fib 1.2) |
| 1.215433 | 0.573 | 0.146 | 2.15E-285 | 4 | Ccl7 | Fibroblast (Fib 1.2) |
| 0.792596 | 0.909 | 0.343 | 2.87E-282 | 4 | Mmp2 | Fibroblast (Fib 1.2) |
| 0.754656 | 0.587 | 0.153 | 4.35E-272 | 4 | Mfap5 | Fibroblast (Fib 1.2) |
| 0.655092 | 0.75 | 0.248 | 6.04E-263 | 4 | Lox | Fibroblast (Fib 1.2) |
| 0.705933 | 0.973 | 0.387 | 3.72E-260 | 4 | Dcn | Fibroblast (Fib 1.2) |
| 0.890973 | 0.977 | 0.441 | 1.03E-259 | 4 | Col6a1 | Fibroblast (Fib 1.2) |
| 0.773643 | 0.944 | 0.404 | 2.32E-248 | 4 | Col6a2 | Fibroblast (Fib 1.2) |
| 0.566657 | 0.438 | 0.092 | 1.25E-246 | 4 | Vcam1 | Fibroblast (Fib 1.2) |
| 1.176545 | 0.613 | 0.187 | 1.54E-245 | 4 | Ccl2 | Fibroblast (Fib 1.2) |
| 0.691703 | 0.657 | 0.197 | 8.91E-243 | 4 | Cthrc1 | Fibroblast (Fib 1.2) |
| 0.557817 | 0.838 | 0.313 | 2.77E-242 | 4 | Rcn3 | Fibroblast (Fib 1.2) |
| 0.809102 | 0.817 | 0.315 | 1.11E-237 | 4 | Aspn | Fibroblast (Fib 1.2) |
| 0.659981 | 0.951 | 0.389 | 8.69E-234 | 4 | Serpinf1 | Fibroblast (Fib 1.2) |
| 0.605142 | 0.821 | 0.317 | 1.38E-233 | 4 | Fbn1 | Fibroblast (Fib 1.2) |
| 0.590071 | 0.839 | 0.328 | 5.58E-231 | 4 | Pcolce | Fibroblast (Fib 1.2) |
| 0.646986 | 0.849 | 0.311 | 1.45E-229 | 4 | Col12a1 | Fibroblast (Fib 1.2) |
| 0.582684 | 0.698 | 0.239 | 5.34E-224 | 4 | Fbln2 | Fibroblast (Fib 1.2) |
| 0.746796 | 0.967 | 0.474 | 1.60E-211 | 4 | Serpinh1 | Fibroblast (Fib 1.2) |
| 0.908891 | 0.776 | 0.331 | 9.03E-207 | 4 | Timp1 | Fibroblast (Fib 1.2) |
| 0.561017 | 0.701 | 0.244 | 1.03E-201 | 4 | Thbs2 | Fibroblast (Fib 1.2) |
| 0.739478 | 0.927 | 0.413 | 8.48E-199 | 4 | Col6a3 | Fibroblast (Fib 1.2) |
| 0.554327 | 0.484 | 0.134 | 2.54E-195 | 4 | Sfrp1 | Fibroblast (Fib 1.2) |
| 0.534936 | 0.868 | 0.383 | 1.53E-187 | 4 | Ppic | Fibroblast (Fib 1.2) |
| 0.890829 | 0.877 | 0.377 | 5.75E-187 | 4 | Lum | Fibroblast (Fib 1.2) |
| 0.979701 | 0.22 | 0.03 | 9.65E-187 | 4 | Saa3 | Fibroblast (Fib 1.2) |
| 0.722339 | 0.97 | 0.525 | 1.56E-186 | 4 | Fn1 | Fibroblast (Fib 1.2) |
| 0.584097 | 0.682 | 0.247 | 5.88E-181 | 4 | Col11a1 | Fibroblast (Fib 1.2) |
| 0.628834 | 0.562 | 0.185 | 2.76E-180 | 4 | Rbp1 | Fibroblast (Fib 1.2) |
| 0.871866 | 0.79 | 0.379 | 9.39E-175 | 4 | Cxcl1 | Fibroblast (Fib 1.2) |
| 0.489215 | 0.353 | 0.085 | 2.14E-165 | 4 | Tnfaip6 | Fibroblast (Fib 1.2) |
| 0.63781 | 0.857 | 0.4 | 5.95E-161 | 4 | Igfbp4 | Fibroblast (Fib 1.2) |
| 0.471508 | 0.798 | 0.358 | 3.60E-160 | 4 | Selenom | Fibroblast (Fib 1.2) |
| 0.576209 | 0.912 | 0.49 | 2.45E-157 | 4 | Timp2 | Fibroblast (Fib 1.2) |
| 0.440522 | 0.607 | 0.227 | 2.99E-154 | 4 | Mxra8 | Fibroblast (Fib 1.2) |
| 0.650073 | 0.609 | 0.235 | 3.56E-154 | 4 | Meg3 | Fibroblast (Fib 1.2) |
| 0.432601 | 0.811 | 0.378 | 1.02E-150 | 4 | Nedd4 | Fibroblast (Fib 1.2) |
| 0.569102 | 0.716 | 0.312 | 2.79E-148 | 4 | Csrp2 | Fibroblast (Fib 1.2) |
| 0.403534 | 0.607 | 0.236 | 1.34E-144 | 4 | Ckap4 | Fibroblast (Fib 1.2) |
| 0.375573 | 0.559 | 0.2 | 1.70E-144 | 4 | Olfml3 | Fibroblast (Fib 1.2) |
| 0.356246 | 0.456 | 0.146 | 1.63E-140 | 4 | C1qtnf6 | Fibroblast (Fib 1.2) |
| 0.324935 | 0.758 | 0.334 | 4.23E-135 | 4 | Ctsk | Fibroblast (Fib 1.2) |
| 0.550569 | 0.613 | 0.244 | 6.34E-135 | 4 | Gpx3 | Fibroblast (Fib 1.2) |
| 0.543611 | 0.822 | 0.431 | 9.90E-132 | 4 | Fosb | Fibroblast (Fib 1.2) |
| 0.272568 | 0.246 | 0.052 | 1.09E-126 | 4 | Fndc1 | Fibroblast (Fib 1.2) |
| 0.48824 | 0.561 | 0.217 | 1.44E-126 | 4 | Mt2 | Fibroblast (Fib 1.2) |
| 0.351383 | 0.511 | 0.191 | 1.09E-124 | 4 | Tmed3 | Fibroblast (Fib 1.2) |
| 0.354545 | 0.495 | 0.178 | 1.97E-124 | 4 | Nfix | Fibroblast (Fib 1.2) |
| 0.366355 | 0.525 | 0.197 | 8.19E-123 | 4 | Mfap2 | Fibroblast (Fib 1.2) |
| 0.391651 | 0.564 | 0.224 | 5.95E-122 | 4 | Tsc22d1 | Fibroblast (Fib 1.2) |
| 0.350069 | 0.454 | 0.157 | 1.56E-121 | 4 | Pdgfra | Fibroblast (Fib 1.2) |
| 0.4559 | 0.816 | 0.447 | 4.03E-121 | 4 | Rrbp1 | Fibroblast (Fib 1.2) |
| 0.344009 | 0.524 | 0.198 | 2.55E-120 | 4 | Loxl1 | Fibroblast (Fib 1.2) |
| 0.358534 | 0.553 | 0.222 | 9.59E-120 | 4 | Mtch1 | Fibroblast (Fib 1.2) |
| 0.347505 | 0.443 | 0.155 | 8.72E-119 | 4 | Bmp1 | Fibroblast (Fib 1.2) |
| 0.314254 | 0.23 | 0.049 | 2.16E-118 | 4 | Ptx3 | Fibroblast (Fib 1.2) |
| 0.390957 | 0.67 | 0.3 | 3.33E-118 | 4 | Kdelr2 | Fibroblast (Fib 1.2) |
| 0.529241 | 0.724 | 0.349 | 7.25E-117 | 4 | Nupr1 | Fibroblast (Fib 1.2) |
| 0.485904 | 0.607 | 0.259 | 9.78E-116 | 4 | Serping1 | Fibroblast (Fib 1.2) |
| 0.511033 | 0.999 | 0.941 | 4.17E-115 | 4 | Rpl41 | Fibroblast (Fib 1.2) |
| 0.481427 | 0.711 | 0.348 | 2.50E-114 | 4 | Mmp14 | Fibroblast (Fib 1.2) |
| 0.403335 | 0.47 | 0.172 | 4.76E-114 | 4 | Cpxm1 | Fibroblast (Fib 1.2) |
| 0.43304 | 0.913 | 0.594 | 1.75E-113 | 4 | Ppib | Fibroblast (Fib 1.2) |
| 0.529611 | 0.994 | 0.865 | 4.88E-113 | 4 | Rps20 | Fibroblast (Fib 1.2) |
| 0.516006 | 0.429 | 0.155 | 5.30E-112 | 4 | Itm2a | Fibroblast (Fib 1.2) |
| 0.498852 | 0.961 | 0.632 | 1.43E-111 | 4 | Cd63 | Fibroblast (Fib 1.2) |
| 0.43715 | 0.815 | 0.423 | 2.32E-109 | 4 | Pmepa1 | Fibroblast (Fib 1.2) |
| 0.289576 | 0.331 | 0.1 | 3.71E-108 | 4 | Ccn4 | Fibroblast (Fib 1.2) |
| 0.361075 | 0.648 | 0.301 | 4.90E-108 | 4 | Tceal9 | Fibroblast (Fib 1.2) |
| 0.376096 | 0.565 | 0.238 | 9.86E-107 | 4 | Cdh11 | Fibroblast (Fib 1.2) |
| 0.408588 | 0.414 | 0.148 | 4.08E-103 | 4 | Ogn | Fibroblast (Fib 1.2) |
| 0.516359 | 0.785 | 0.452 | 1.45E-102 | 4 | Mt1 | Fibroblast (Fib 1.2) |
| 0.291375 | 0.359 | 0.118 | 1.90E-101 | 4 | Antxr1 | Fibroblast (Fib 1.2) |
| 0.320317 | 0.405 | 0.148 | 3.08E-98 | 4 | Col16a1 | Fibroblast (Fib 1.2) |
| 0.444234 | 0.947 | 0.698 | 1.78E-96 | 4 | Nme2 | Fibroblast (Fib 1.2) |
| 0.250674 | 0.185 | 0.039 | 1.15E-95 | 4 | Ereg | Fibroblast (Fib 1.2) |
| 0.298418 | 0.437 | 0.17 | 5.59E-94 | 4 | Maged1 | Fibroblast (Fib 1.2) |
| 0.332682 | 0.259 | 0.072 | 7.72E-94 | 4 | Il1rl1 | Fibroblast (Fib 1.2) |
| 0.348745 | 0.867 | 0.482 | 3.82E-93 | 4 | Laptm4a | Fibroblast (Fib 1.2) |
| 0.277938 | 0.451 | 0.175 | 5.89E-93 | 4 | Vcan | Fibroblast (Fib 1.2) |
| 0.315352 | 0.576 | 0.265 | 4.66E-92 | 4 | Hdlbp | Fibroblast (Fib 1.2) |
| 0.309873 | 0.398 | 0.145 | 1.46E-89 | 4 | Mfap4 | Fibroblast (Fib 1.2) |
| 0.328548 | 0.485 | 0.205 | 3.22E-89 | 4 | Ccdc80 | Fibroblast (Fib 1.2) |
| 0.320066 | 0.713 | 0.376 | 2.45E-86 | 4 | P4hb | Fibroblast (Fib 1.2) |
| 0.478997 | 0.704 | 0.392 | 7.13E-86 | 4 | Jun | Fibroblast (Fib 1.2) |
| 0.365483 | 0.848 | 0.552 | 1.00E-85 | 4 | Rpl22l1 | Fibroblast (Fib 1.2) |
| 0.319826 | 0.677 | 0.347 | 3.39E-81 | 4 | Lrp1 | Fibroblast (Fib 1.2) |
| 0.257357 | 0.342 | 0.123 | 4.06E-80 | 4 | Sod3 | Fibroblast (Fib 1.2) |
| 0.306964 | 0.418 | 0.172 | 6.74E-80 | 4 | Gas1 | Fibroblast (Fib 1.2) |
| 0.492441 | 0.952 | 0.72 | 9.52E-80 | 4 | Tmsb10 | Fibroblast (Fib 1.2) |
| 0.402954 | 0.463 | 0.207 | 8.60E-77 | 4 | Sgk1 | Fibroblast (Fib 1.2) |
| 0.340573 | 0.931 | 0.617 | 8.56E-76 | 4 | Gnas | Fibroblast (Fib 1.2) |
| 0.323002 | 0.783 | 0.467 | 1.24E-75 | 4 | Gas5 | Fibroblast (Fib 1.2) |
| 0.291049 | 0.321 | 0.117 | 3.46E-75 | 4 | Serpine1 | Fibroblast (Fib 1.2) |
| 0.458918 | 0.973 | 0.724 | 4.46E-75 | 4 | Lgals1 | Fibroblast (Fib 1.2) |
| 0.279055 | 0.523 | 0.247 | 6.78E-75 | 4 | Ssr2 | Fibroblast (Fib 1.2) |
| 0.36265 | 0.983 | 0.884 | 5.01E-73 | 4 | Rps12 | Fibroblast (Fib 1.2) |
| 0.287497 | 0.429 | 0.189 | 3.85E-70 | 4 | Fgfr1 | Fibroblast (Fib 1.2) |
| 0.356054 | 0.993 | 0.862 | 1.63E-69 | 4 | Rplp0 | Fibroblast (Fib 1.2) |
| 0.583962 | 0.943 | 0.787 | 3.29E-69 | 4 | Gm10076 | Fibroblast (Fib 1.2) |
| 0.27924 | 0.613 | 0.315 | 2.17E-68 | 4 | Prrx1 | Fibroblast (Fib 1.2) |
| 0.426385 | 0.477 | 0.234 | 9.90E-68 | 4 | Errfi1 | Fibroblast (Fib 1.2) |
| 0.277009 | 0.417 | 0.187 | 1.37E-65 | 4 | Olfml2b | Fibroblast (Fib 1.2) |
| 0.347976 | 0.985 | 0.855 | 2.17E-65 | 4 | Rpl28 | Fibroblast (Fib 1.2) |
| 0.267461 | 0.737 | 0.419 | 2.62E-65 | 4 | Slc25a4 | Fibroblast (Fib 1.2) |
| 0.287185 | 0.587 | 0.3 | 8.32E-65 | 4 | Egr1 | Fibroblast (Fib 1.2) |
| 0.310692 | 0.919 | 0.676 | 3.54E-64 | 4 | Eef1b2 | Fibroblast (Fib 1.2) |
| 0.269938 | 0.723 | 0.422 | 2.07E-63 | 4 | Bsg | Fibroblast (Fib 1.2) |
| 0.284714 | 0.368 | 0.152 | 3.33E-63 | 4 | Tnn | Fibroblast (Fib 1.2) |
| 0.342581 | 0.85 | 0.602 | 5.92E-63 | 4 | Sec61g | Fibroblast (Fib 1.2) |
| 0.326045 | 0.905 | 0.62 | 6.00E-63 | 4 | Lmna | Fibroblast (Fib 1.2) |
| 0.253686 | 0.488 | 0.239 | 1.91E-62 | 4 | Nenf | Fibroblast (Fib 1.2) |
| 0.25202 | 0.45 | 0.217 | 1.50E-58 | 4 | Serpinb6a | Fibroblast (Fib 1.2) |
| 0.295582 | 0.849 | 0.576 | 3.66E-57 | 4 | Npm1 | Fibroblast (Fib 1.2) |
| 0.352064 | 0.989 | 0.901 | 3.71E-56 | 4 | Rpl39 | Fibroblast (Fib 1.2) |
| 0.267014 | 0.684 | 0.415 | 4.51E-55 | 4 | Ssr4 | Fibroblast (Fib 1.2) |
| 0.326291 | 0.994 | 0.91 | 1.49E-54 | 4 | Rps8 | Fibroblast (Fib 1.2) |
| 0.314981 | 0.36 | 0.16 | 1.54E-54 | 4 | Igf1 | Fibroblast (Fib 1.2) |
| 0.252353 | 0.989 | 0.87 | 1.92E-54 | 4 | Rplp1 | Fibroblast (Fib 1.2) |
| 0.254823 | 0.475 | 0.247 | 4.66E-52 | 4 | Calu | Fibroblast (Fib 1.2) |
| 0.287233 | 0.944 | 0.832 | 8.91E-52 | 4 | Serf2 | Fibroblast (Fib 1.2) |
| 0.28388 | 0.625 | 0.386 | 2.70E-50 | 4 | Gm10260 | Fibroblast (Fib 1.2) |
| 0.280933 | 0.736 | 0.49 | 4.30E-50 | 4 | Sec61b | Fibroblast (Fib 1.2) |
| 0.264577 | 0.345 | 0.158 | 4.40E-49 | 4 | Nrep | Fibroblast (Fib 1.2) |
| 0.270016 | 0.957 | 0.78 | 7.00E-49 | 4 | Rpl10a | Fibroblast (Fib 1.2) |
| 0.251453 | 0.433 | 0.214 | 7.88E-49 | 4 | Col5a3 | Fibroblast (Fib 1.2) |
| 0.286711 | 0.964 | 0.817 | 5.44E-48 | 4 | Rpl15 | Fibroblast (Fib 1.2) |
| 0.265115 | 0.996 | 0.905 | 1.19E-47 | 4 | Rps28 | Fibroblast (Fib 1.2) |
| 0.289244 | 0.979 | 0.85 | 3.26E-47 | 4 | Rps15a | Fibroblast (Fib 1.2) |
| 0.265492 | 0.895 | 0.68 | 2.80E-45 | 4 | Rpl36a | Fibroblast (Fib 1.2) |
| 0.252885 | 0.787 | 0.567 | 6.07E-44 | 4 | Rpl31 | Fibroblast (Fib 1.2) |
| 0.350938 | 0.299 | 0.135 | 5.00E-43 | 4 | Igfbp3 | Fibroblast (Fib 1.2) |
| 0.250698 | 0.386 | 0.196 | 2.42E-42 | 4 | Nr4a2 | Fibroblast (Fib 1.2) |
| 0.277818 | 0.5 | 0.287 | 6.11E-40 | 4 | Klf4 | Fibroblast (Fib 1.2) |
| 0.29188 | 0.636 | 0.429 | 1.88E-35 | 4 | Neat1 | Fibroblast (Fib 1.2) |
| 0.259559 | 0.317 | 0.164 | 8.98E-34 | 4 | Hk2 | Fibroblast (Fib 1.2) |
| 0.260787 | 0.277 | 0.139 | 4.72E-30 | 4 | Inhba | Fibroblast (Fib 1.2) |
| 0.404271 | 0.191 | 0.124 | 1.01E-06 | 4 | Hp | Fibroblast (Fib 1.2) |
| 1.820917 | 1 | 0.531 | 0 | 5 | Col1a1 | Fibroblast (Fib 1.3) |
| 1.740014 | 1 | 0.542 | 0 | 5 | Col1a2 | Fibroblast (Fib 1.3) |
| 1.710149 | 0.972 | 0.374 | 0 | 5 | Lum | Fibroblast (Fib 1.3) |
| 1.659564 | 1 | 0.567 | 0 | 5 | Sparc | Fibroblast (Fib 1.3) |
| 1.525398 | 0.774 | 0.236 | 0 | 5 | Gpx3 | Fibroblast (Fib 1.3) |
| 1.361819 | 0.917 | 0.309 | 0 | 5 | Col12a1 | Fibroblast (Fib 1.3) |
| 1.352076 | 0.948 | 0.396 | 0 | 5 | Igfbp4 | Fibroblast (Fib 1.3) |
| 1.289276 | 0.892 | 0.236 | 0 | 5 | Col11a1 | Fibroblast (Fib 1.3) |
| 1.165562 | 0.877 | 0.314 | 0 | 5 | Aspn | Fibroblast (Fib 1.3) |
| 1.14226 | 0.976 | 0.39 | 0 | 5 | Dcn | Fibroblast (Fib 1.3) |
| 1.051539 | 0.948 | 0.369 | 0 | 5 | Aebp1 | Fibroblast (Fib 1.3) |
| 1.0296 | 0.958 | 0.392 | 0 | 5 | Serpinf1 | Fibroblast (Fib 1.3) |
| 0.995985 | 0.605 | 0.113 | 0 | 5 | Fmod | Fibroblast (Fib 1.3) |
| 0.908899 | 0.652 | 0.134 | 0 | 5 | Ogn | Fibroblast (Fib 1.3) |
| 0.908038 | 0.901 | 0.326 | 0 | 5 | Pcolce | Fibroblast (Fib 1.3) |
| 0.884929 | 0.783 | 0.241 | 0 | 5 | Thbs2 | Fibroblast (Fib 1.3) |
| 0.883353 | 0.57 | 0.066 | 0 | 5 | Omd | Fibroblast (Fib 1.3) |
| 0.881378 | 0.519 | 0.086 | 0 | 5 | Ecrg4 | Fibroblast (Fib 1.3) |
| 0.747749 | 0.909 | 0.326 | 0 | 5 | Ctsk | Fibroblast (Fib 1.3) |
| 0.744806 | 0.355 | 0.033 | 0 | 5 | Matn4 | Fibroblast (Fib 1.3) |
| 0.708393 | 0.708 | 0.186 | 0 | 5 | Mfap2 | Fibroblast (Fib 1.3) |
| 0.690672 | 0.667 | 0.161 | 0 | 5 | Cpxm1 | Fibroblast (Fib 1.3) |
| 0.600404 | 0.521 | 0.088 | 0 | 5 | Itgbl1 | Fibroblast (Fib 1.3) |
| 0.949151 | 0.966 | 0.489 | 2.40E-303 | 5 | Timp2 | Fibroblast (Fib 1.3) |
| 0.434396 | 0.259 | 0.024 | 3.26E-290 | 5 | Tnmd | Fibroblast (Fib 1.3) |
| 1.096493 | 0.996 | 0.504 | 9.33E-287 | 5 | Bgn | Fibroblast (Fib 1.3) |
| 0.817441 | 0.94 | 0.344 | 1.33E-286 | 5 | Mmp2 | Fibroblast (Fib 1.3) |
| 0.921275 | 0.756 | 0.25 | 1.29E-278 | 5 | Htra1 | Fibroblast (Fib 1.3) |
| 1.234426 | 0.971 | 0.43 | 2.18E-266 | 5 | Postn | Fibroblast (Fib 1.3) |
| 0.617415 | 0.721 | 0.222 | 1.25E-264 | 5 | Mxra8 | Fibroblast (Fib 1.3) |
| 1.292122 | 0.996 | 0.533 | 3.09E-262 | 5 | Col3a1 | Fibroblast (Fib 1.3) |
| 0.782467 | 0.558 | 0.136 | 5.71E-261 | 5 | Mfap4 | Fibroblast (Fib 1.3) |
| 0.614672 | 0.309 | 0.041 | 8.45E-257 | 5 | Wif1 | Fibroblast (Fib 1.3) |
| 0.639062 | 0.725 | 0.23 | 3.83E-255 | 5 | Cdh11 | Fibroblast (Fib 1.3) |
| 0.643167 | 0.464 | 0.097 | 5.71E-255 | 5 | Ccn1 | Fibroblast (Fib 1.3) |
| 0.958936 | 0.981 | 0.47 | 2.09E-254 | 5 | Col5a2 | Fibroblast (Fib 1.3) |
| 1.496876 | 0.828 | 0.455 | 1.34E-233 | 5 | Fos | Fibroblast (Fib 1.3) |
| 0.509127 | 0.539 | 0.14 | 5.49E-227 | 5 | Col16a1 | Fibroblast (Fib 1.3) |
| 0.401551 | 0.394 | 0.076 | 1.38E-225 | 5 | Fbln5 | Fibroblast (Fib 1.3) |
| 0.69014 | 0.547 | 0.148 | 1.59E-220 | 5 | Itm2a | Fibroblast (Fib 1.3) |
| 1.104788 | 0.809 | 0.387 | 3.74E-219 | 5 | Jun | Fibroblast (Fib 1.3) |
| 0.580964 | 0.551 | 0.149 | 9.88E-219 | 5 | Igf1 | Fibroblast (Fib 1.3) |
| 0.540039 | 0.63 | 0.198 | 9.42E-217 | 5 | Olfml3 | Fibroblast (Fib 1.3) |
| 0.598203 | 0.808 | 0.318 | 2.26E-216 | 5 | Rcn3 | Fibroblast (Fib 1.3) |
| 0.849753 | 0.739 | 0.292 | 8.78E-215 | 5 | Egr1 | Fibroblast (Fib 1.3) |
| 0.581038 | 0.438 | 0.101 | 9.27E-213 | 5 | Wnt5a | Fibroblast (Fib 1.3) |
| 0.854834 | 0.964 | 0.477 | 4.76E-212 | 5 | Serpinh1 | Fibroblast (Fib 1.3) |
| 0.338355 | 0.308 | 0.05 | 9.62E-207 | 5 | Clec11a | Fibroblast (Fib 1.3) |
| 0.653345 | 0.516 | 0.144 | 3.23E-198 | 5 | Tnn | Fibroblast (Fib 1.3) |
| 0.718883 | 0.43 | 0.101 | 5.73E-198 | 5 | Thbs4 | Fibroblast (Fib 1.3) |
| 0.747795 | 0.947 | 0.446 | 2.25E-189 | 5 | Col6a1 | Fibroblast (Fib 1.3) |
| 0.389639 | 0.423 | 0.101 | 1.63E-185 | 5 | Prrx2 | Fibroblast (Fib 1.3) |
| 0.39418 | 0.412 | 0.099 | 2.67E-181 | 5 | Scarf2 | Fibroblast (Fib 1.3) |
| 0.485134 | 0.299 | 0.054 | 7.47E-181 | 5 | Chrdl1 | Fibroblast (Fib 1.3) |
| 0.651681 | 0.904 | 0.409 | 2.98E-180 | 5 | Col6a2 | Fibroblast (Fib 1.3) |
| 0.47139 | 0.381 | 0.085 | 5.46E-177 | 5 | Angptl1 | Fibroblast (Fib 1.3) |
| 0.563039 | 0.844 | 0.387 | 4.89E-176 | 5 | Ppic | Fibroblast (Fib 1.3) |
| 0.376991 | 0.327 | 0.066 | 2.61E-175 | 5 | Prelp | Fibroblast (Fib 1.3) |
| 0.420606 | 0.435 | 0.113 | 1.43E-173 | 5 | Twist1 | Fibroblast (Fib 1.3) |
| 0.900756 | 0.811 | 0.434 | 8.70E-172 | 5 | Fosb | Fibroblast (Fib 1.3) |
| 0.349818 | 0.311 | 0.061 | 3.56E-171 | 5 | Epha3 | Fibroblast (Fib 1.3) |
| 0.751894 | 0.44 | 0.12 | 6.16E-169 | 5 | Sfrp2 | Fibroblast (Fib 1.3) |
| 0.544886 | 0.797 | 0.36 | 1.15E-166 | 5 | Selenom | Fibroblast (Fib 1.3) |
| 0.601586 | 0.891 | 0.394 | 1.70E-166 | 5 | Col5a1 | Fibroblast (Fib 1.3) |
| 0.37584 | 0.331 | 0.074 | 5.79E-156 | 5 | Spon1 | Fibroblast (Fib 1.3) |
| 0.422274 | 0.425 | 0.117 | 7.44E-155 | 5 | Mdk | Fibroblast (Fib 1.3) |
| 0.268499 | 0.226 | 0.038 | 1.21E-146 | 5 | Srpx | Fibroblast (Fib 1.3) |
| 0.363998 | 0.225 | 0.038 | 9.42E-146 | 5 | Mest | Fibroblast (Fib 1.3) |
| 0.772768 | 0.366 | 0.095 | 3.53E-145 | 5 | Eln | Fibroblast (Fib 1.3) |
| 0.584304 | 0.916 | 0.596 | 4.26E-142 | 5 | Ppib | Fibroblast (Fib 1.3) |
| 0.639701 | 0.477 | 0.153 | 8.18E-140 | 5 | Crabp1 | Fibroblast (Fib 1.3) |
| 0.38865 | 0.473 | 0.154 | 1.40E-137 | 5 | Vkorc1 | Fibroblast (Fib 1.3) |
| 0.634507 | 0.706 | 0.322 | 4.30E-137 | 5 | Id3 | Fibroblast (Fib 1.3) |
| 0.492047 | 0.376 | 0.106 | 1.38E-133 | 5 | Ccn5 | Fibroblast (Fib 1.3) |
| 0.73063 | 0.726 | 0.351 | 2.10E-132 | 5 | Nupr1 | Fibroblast (Fib 1.3) |
| 0.452844 | 0.689 | 0.301 | 3.19E-132 | 5 | Kdelr2 | Fibroblast (Fib 1.3) |
| 0.537855 | 0.854 | 0.414 | 4.01E-126 | 5 | Fstl1 | Fibroblast (Fib 1.3) |
| 0.456879 | 0.872 | 0.484 | 5.29E-125 | 5 | Laptm4a | Fibroblast (Fib 1.3) |
| 0.264824 | 0.252 | 0.053 | 8.31E-124 | 5 | Fndc1 | Fibroblast (Fib 1.3) |
| 0.377755 | 0.504 | 0.179 | 2.45E-122 | 5 | Nfix | Fibroblast (Fib 1.3) |
| 0.266562 | 0.227 | 0.045 | 2.34E-121 | 5 | Cpxm2 | Fibroblast (Fib 1.3) |
| 0.305754 | 0.3 | 0.075 | 3.08E-121 | 5 | Pdgfrl | Fibroblast (Fib 1.3) |
| 0.371739 | 0.516 | 0.193 | 4.06E-121 | 5 | Tmed3 | Fibroblast (Fib 1.3) |
| 0.402943 | 0.178 | 0.029 | 5.87E-117 | 5 | Igfbp2 | Fibroblast (Fib 1.3) |
| 0.573798 | 0.953 | 0.635 | 2.23E-116 | 5 | Cd63 | Fibroblast (Fib 1.3) |
| 0.260438 | 0.225 | 0.046 | 1.14E-115 | 5 | Fzd1 | Fibroblast (Fib 1.3) |
| 0.39984 | 0.523 | 0.204 | 6.23E-114 | 5 | Ccdc80 | Fibroblast (Fib 1.3) |
| 0.522624 | 0.545 | 0.216 | 2.44E-113 | 5 | Nbl1 | Fibroblast (Fib 1.3) |
| 0.451237 | 0.42 | 0.14 | 3.53E-113 | 5 | Sfrp1 | Fibroblast (Fib 1.3) |
| 0.297774 | 0.327 | 0.091 | 2.20E-112 | 5 | Colec12 | Fibroblast (Fib 1.3) |
| 0.360636 | 0.567 | 0.235 | 1.21E-109 | 5 | Nenf | Fibroblast (Fib 1.3) |
| 0.458851 | 0.713 | 0.35 | 2.06E-107 | 5 | Mmp14 | Fibroblast (Fib 1.3) |
| 0.392051 | 0.611 | 0.26 | 1.65E-106 | 5 | Lox | Fibroblast (Fib 1.3) |
| 0.305275 | 0.358 | 0.111 | 7.68E-106 | 5 | Fkbp7 | Fibroblast (Fib 1.3) |
| 0.416854 | 0.667 | 0.313 | 1.98E-105 | 5 | Prrx1 | Fibroblast (Fib 1.3) |
| 0.367186 | 0.652 | 0.303 | 2.84E-103 | 5 | Tceal9 | Fibroblast (Fib 1.3) |
| 0.25299 | 0.26 | 0.067 | 3.56E-98 | 5 | Mmp23 | Fibroblast (Fib 1.3) |
| 0.587025 | 0.382 | 0.131 | 2.07E-97 | 5 | Igfbp3 | Fibroblast (Fib 1.3) |
| 0.305599 | 0.377 | 0.127 | 3.42E-97 | 5 | Fkbp9 | Fibroblast (Fib 1.3) |
| 0.326299 | 0.421 | 0.153 | 1.21E-96 | 5 | Cd302 | Fibroblast (Fib 1.3) |
| 0.390488 | 0.751 | 0.422 | 2.15E-96 | 5 | Bsg | Fibroblast (Fib 1.3) |
| 0.297963 | 0.391 | 0.137 | 2.05E-95 | 5 | Cnpy2 | Fibroblast (Fib 1.3) |
| 0.330105 | 0.351 | 0.113 | 3.92E-95 | 5 | Fbln1 | Fibroblast (Fib 1.3) |
| 0.420721 | 0.839 | 0.422 | 8.34E-94 | 5 | Col6a3 | Fibroblast (Fib 1.3) |
| 0.383108 | 0.756 | 0.419 | 2.39E-92 | 5 | Slc25a4 | Fibroblast (Fib 1.3) |
| 0.359484 | 0.737 | 0.386 | 3.12E-90 | 5 | Nedd4 | Fibroblast (Fib 1.3) |
| 0.378894 | 0.221 | 0.054 | 2.43E-89 | 5 | Cxcl14 | Fibroblast (Fib 1.3) |
| 0.432971 | 0.31 | 0.098 | 5.38E-89 | 5 | Gadd45g | Fibroblast (Fib 1.3) |
| 0.413745 | 0.924 | 0.619 | 1.01E-87 | 5 | Gnas | Fibroblast (Fib 1.3) |
| 0.44017 | 0.435 | 0.173 | 2.45E-86 | 5 | Col8a1 | Fibroblast (Fib 1.3) |
| 0.421734 | 0.925 | 0.53 | 1.03E-84 | 5 | Fn1 | Fibroblast (Fib 1.3) |
| 0.327163 | 0.267 | 0.078 | 6.24E-84 | 5 | Hes1 | Fibroblast (Fib 1.3) |
| 0.288232 | 0.376 | 0.142 | 6.81E-80 | 5 | Copz2 | Fibroblast (Fib 1.3) |
| 0.337929 | 0.421 | 0.173 | 7.78E-76 | 5 | Gas1 | Fibroblast (Fib 1.3) |
| 0.470968 | 0.523 | 0.243 | 8.41E-76 | 5 | Meg3 | Fibroblast (Fib 1.3) |
| 0.348714 | 0.711 | 0.393 | 2.10E-75 | 5 | Cd81 | Fibroblast (Fib 1.3) |
| 0.359756 | 0.504 | 0.23 | 3.68E-74 | 5 | Tsc22d1 | Fibroblast (Fib 1.3) |
| 0.279031 | 0.277 | 0.089 | 3.50E-73 | 5 | Gas6 | Fibroblast (Fib 1.3) |
| 0.257387 | 0.314 | 0.11 | 3.80E-73 | 5 | Efemp2 | Fibroblast (Fib 1.3) |
| 0.369546 | 0.762 | 0.429 | 4.35E-71 | 5 | Pmepa1 | Fibroblast (Fib 1.3) |
| 0.429901 | 0.977 | 0.725 | 2.29E-70 | 5 | Lgals1 | Fibroblast (Fib 1.3) |
| 0.473074 | 0.545 | 0.275 | 6.63E-70 | 5 | Cebpd | Fibroblast (Fib 1.3) |
| 0.272285 | 0.373 | 0.151 | 3.84E-68 | 5 | Txndc5 | Fibroblast (Fib 1.3) |
| 0.275704 | 0.375 | 0.152 | 6.32E-68 | 5 | Snhg18 | Fibroblast (Fib 1.3) |
| 0.329694 | 0.693 | 0.416 | 1.26E-65 | 5 | Ssr4 | Fibroblast (Fib 1.3) |
| 0.313439 | 0.753 | 0.463 | 1.32E-64 | 5 | Selenof | Fibroblast (Fib 1.3) |
| 0.318615 | 0.682 | 0.402 | 3.66E-63 | 5 | Arf4 | Fibroblast (Fib 1.3) |
| 0.402353 | 0.726 | 0.443 | 9.71E-63 | 5 | Dusp1 | Fibroblast (Fib 1.3) |
| 0.263444 | 0.368 | 0.153 | 9.77E-63 | 5 | Itm2c | Fibroblast (Fib 1.3) |
| 0.273717 | 0.444 | 0.2 | 8.95E-62 | 5 | Zbtb20 | Fibroblast (Fib 1.3) |
| 0.2552 | 0.318 | 0.124 | 2.70E-60 | 5 | Bicc1 | Fibroblast (Fib 1.3) |
| 0.367919 | 0.422 | 0.195 | 5.42E-60 | 5 | Gem | Fibroblast (Fib 1.3) |
| 0.42319 | 0.592 | 0.336 | 7.88E-59 | 5 | Nr4a1 | Fibroblast (Fib 1.3) |
| 0.31428 | 0.217 | 0.069 | 2.48E-58 | 5 | Ptn | Fibroblast (Fib 1.3) |
| 0.262696 | 0.417 | 0.191 | 3.35E-57 | 5 | Fgfr1 | Fibroblast (Fib 1.3) |
| 0.276204 | 0.541 | 0.265 | 4.69E-57 | 5 | Serping1 | Fibroblast (Fib 1.3) |
| 0.299069 | 0.755 | 0.453 | 8.89E-57 | 5 | Rrbp1 | Fibroblast (Fib 1.3) |
| 0.369761 | 0.534 | 0.286 | 1.29E-55 | 5 | Klf4 | Fibroblast (Fib 1.3) |
| 0.26618 | 0.441 | 0.214 | 6.32E-55 | 5 | Eid1 | Fibroblast (Fib 1.3) |
| 0.262613 | 0.433 | 0.207 | 1.58E-54 | 5 | Cnn3 | Fibroblast (Fib 1.3) |
| 0.355777 | 0.463 | 0.237 | 1.01E-53 | 5 | Ier2 | Fibroblast (Fib 1.3) |
| 0.262415 | 0.384 | 0.177 | 3.83E-53 | 5 | Prdx4 | Fibroblast (Fib 1.3) |
| 0.491713 | 0.497 | 0.268 | 1.05E-52 | 5 | Klf2 | Fibroblast (Fib 1.3) |
| 0.253698 | 0.388 | 0.179 | 1.25E-52 | 5 | Itgb5 | Fibroblast (Fib 1.3) |
| 0.261857 | 0.66 | 0.382 | 7.21E-52 | 5 | P4hb | Fibroblast (Fib 1.3) |
| 0.276078 | 0.492 | 0.262 | 6.75E-51 | 5 | Pebp1 | Fibroblast (Fib 1.3) |
| 0.283352 | 0.611 | 0.353 | 6.84E-51 | 5 | Lrp1 | Fibroblast (Fib 1.3) |
| 0.285236 | 0.74 | 0.472 | 1.52E-50 | 5 | Gas5 | Fibroblast (Fib 1.3) |
| 0.250258 | 0.377 | 0.175 | 3.20E-50 | 5 | Maged1 | Fibroblast (Fib 1.3) |
| 0.31974 | 0.946 | 0.833 | 5.75E-49 | 5 | Serf2 | Fibroblast (Fib 1.3) |
| 0.40331 | 0.555 | 0.327 | 7.57E-49 | 5 | Socs3 | Fibroblast (Fib 1.3) |
| 0.266501 | 0.47 | 0.247 | 2.11E-48 | 5 | Ckap4 | Fibroblast (Fib 1.3) |
| 0.256497 | 0.591 | 0.335 | 9.51E-46 | 5 | Fbn1 | Fibroblast (Fib 1.3) |
| 0.302876 | 0.575 | 0.324 | 2.47E-45 | 5 | Csrp2 | Fibroblast (Fib 1.3) |
| 0.351842 | 0.425 | 0.215 | 2.47E-45 | 5 | Cthrc1 | Fibroblast (Fib 1.3) |
| 0.252322 | 0.463 | 0.251 | 2.15E-41 | 5 | Tcf4 | Fibroblast (Fib 1.3) |
| 0.382154 | 0.327 | 0.153 | 3.01E-41 | 5 | Igfbp5 | Fibroblast (Fib 1.3) |
| 0.283593 | 0.445 | 0.246 | 1.83E-37 | 5 | Ecm1 | Fibroblast (Fib 1.3) |
| 0.267348 | 0.982 | 0.863 | 2.11E-34 | 5 | Rplp0 | Fibroblast (Fib 1.3) |
| 0.430749 | 0.372 | 0.235 | 4.85E-16 | 5 | Serpine2 | Fibroblast (Fib 1.3) |
| 3.078454 | 0.994 | 0.201 | 0 | 6 | Lyz2 | Macrophages (Mac 1.1) |
| 3.072091 | 0.908 | 0.218 | 0 | 6 | Apoe | Macrophages (Mac 1.1) |
| 2.788912 | 0.707 | 0.047 | 0 | 6 | Pf4 | Macrophages (Mac 1.1) |
| 2.343072 | 0.998 | 0.908 | 0 | 6 | Ftl1 | Macrophages (Mac 1.1) |
| 2.133294 | 0.986 | 0.509 | 0 | 6 | Ctsb | Macrophages (Mac 1.1) |
| 2.083332 | 0.962 | 0.364 | 0 | 6 | Lgals3 | Macrophages (Mac 1.1) |
| 2.079215 | 0.985 | 0.444 | 0 | 6 | Psap | Macrophages (Mac 1.1) |
| 2.027573 | 0.593 | 0.128 | 0 | 6 | Hmox1 | Macrophages (Mac 1.1) |
| 1.896141 | 0.928 | 0.287 | 0 | 6 | Ctsd | Macrophages (Mac 1.1) |
| 1.816921 | 0.963 | 0.186 | 0 | 6 | Ctss | Macrophages (Mac 1.1) |
| 1.498463 | 0.841 | 0.137 | 0 | 6 | Lgmn | Macrophages (Mac 1.1) |
| 1.476607 | 0.737 | 0.077 | 0 | 6 | Ms4a7 | Macrophages (Mac 1.1) |
| 1.318133 | 0.944 | 0.341 | 0 | 6 | Cstb | Macrophages (Mac 1.1) |
| 1.305102 | 0.525 | 0.063 | 0 | 6 | C1qb | Macrophages (Mac 1.1) |
| 1.238165 | 0.908 | 0.217 | 0 | 6 | Grn | Macrophages (Mac 1.1) |
| 1.228676 | 0.929 | 0.376 | 0 | 6 | Cyba | Macrophages (Mac 1.1) |
| 1.224042 | 0.483 | 0.058 | 0 | 6 | C1qa | Macrophages (Mac 1.1) |
| 1.091721 | 0.795 | 0.113 | 0 | 6 | Mpeg1 | Macrophages (Mac 1.1) |
| 1.036249 | 0.297 | 0.023 | 0 | 6 | Arg1 | Macrophages (Mac 1.1) |
| 0.990593 | 0.767 | 0.107 | 0 | 6 | Cd68 | Macrophages (Mac 1.1) |
| 0.93414 | 0.88 | 0.309 | 0 | 6 | Ctsz | Macrophages (Mac 1.1) |
| 0.92691 | 0.583 | 0.091 | 0 | 6 | Ccl9 | Macrophages (Mac 1.1) |
| 0.925354 | 0.671 | 0.079 | 0 | 6 | Plin2 | Macrophages (Mac 1.1) |
| 0.901127 | 0.651 | 0.054 | 0 | 6 | Trem2 | Macrophages (Mac 1.1) |
| 0.888156 | 0.468 | 0.051 | 0 | 6 | C1qc | Macrophages (Mac 1.1) |
| 0.875716 | 0.602 | 0.102 | 0 | 6 | Mafb | Macrophages (Mac 1.1) |
| 0.781883 | 0.643 | 0.082 | 0 | 6 | Ms4a6d | Macrophages (Mac 1.1) |
| 0.733794 | 0.555 | 0.075 | 0 | 6 | Abca1 | Macrophages (Mac 1.1) |
| 0.683287 | 0.593 | 0.114 | 0 | 6 | Ms4a6c | Macrophages (Mac 1.1) |
| 0.682345 | 0.505 | 0.08 | 0 | 6 | Creg1 | Macrophages (Mac 1.1) |
| 0.663153 | 0.622 | 0.132 | 0 | 6 | Ctsa | Macrophages (Mac 1.1) |
| 0.649243 | 0.574 | 0.093 | 0 | 6 | Sirpa | Macrophages (Mac 1.1) |
| 0.626889 | 0.504 | 0.04 | 0 | 6 | C3ar1 | Macrophages (Mac 1.1) |
| 0.585376 | 0.438 | 0.054 | 0 | 6 | Msr1 | Macrophages (Mac 1.1) |
| 0.524202 | 0.299 | 0.009 | 0 | 6 | Cd36 | Macrophages (Mac 1.1) |
| 0.51419 | 0.417 | 0.047 | 0 | 6 | Cd93 | Macrophages (Mac 1.1) |
| 0.491784 | 0.344 | 0.025 | 0 | 6 | Stab1 | Macrophages (Mac 1.1) |
| 0.487566 | 0.309 | 0.029 | 0 | 6 | Mrc1 | Macrophages (Mac 1.1) |
| 0.428257 | 0.318 | 0.028 | 0 | 6 | Adgre1 | Macrophages (Mac 1.1) |
| 0.331105 | 0.237 | 0.008 | 0 | 6 | Syngr1 | Macrophages (Mac 1.1) |
| 0.584336 | 0.538 | 0.095 | 1.89E-302 | 6 | Cxcl16 | Macrophages (Mac 1.1) |
| 1.034496 | 0.947 | 0.286 | 1.61E-301 | 6 | Fcer1g | Macrophages (Mac 1.1) |
| 0.499296 | 0.459 | 0.071 | 6.09E-298 | 6 | Cd300c2 | Macrophages (Mac 1.1) |
| 1.024792 | 0.955 | 0.474 | 1.62E-297 | 6 | Atp6v0c | Macrophages (Mac 1.1) |
| 0.924721 | 0.857 | 0.357 | 2.24E-287 | 6 | Lamp1 | Macrophages (Mac 1.1) |
| 0.727256 | 0.775 | 0.233 | 9.26E-270 | 6 | Sdcbp | Macrophages (Mac 1.1) |
| 0.632401 | 0.494 | 0.093 | 1.98E-266 | 6 | Dab2 | Macrophages (Mac 1.1) |
| 0.655124 | 0.695 | 0.179 | 1.92E-265 | 6 | Ninj1 | Macrophages (Mac 1.1) |
| 0.717984 | 0.309 | 0.036 | 4.95E-265 | 6 | Gpnmb | Macrophages (Mac 1.1) |
| 0.515253 | 0.469 | 0.085 | 1.34E-259 | 6 | Atp6v1a | Macrophages (Mac 1.1) |
| 0.426398 | 0.329 | 0.04 | 9.98E-258 | 6 | F13a1 | Macrophages (Mac 1.1) |
| 0.443688 | 0.33 | 0.042 | 1.13E-254 | 6 | Lipa | Macrophages (Mac 1.1) |
| 0.849838 | 0.266 | 0.027 | 1.71E-246 | 6 | Il10 | Macrophages (Mac 1.1) |
| 0.385143 | 0.315 | 0.04 | 2.38E-240 | 6 | Anxa4 | Macrophages (Mac 1.1) |
| 1.411415 | 1 | 0.947 | 3.93E-240 | 6 | Fth1 | Macrophages (Mac 1.1) |
| 0.913677 | 0.656 | 0.181 | 8.20E-239 | 6 | Id2 | Macrophages (Mac 1.1) |
| 1.235223 | 0.912 | 0.384 | 4.61E-237 | 6 | Cxcl2 | Macrophages (Mac 1.1) |
| 0.697545 | 0.785 | 0.247 | 5.58E-237 | 6 | Laptm5 | Macrophages (Mac 1.1) |
| 0.612501 | 0.744 | 0.207 | 4.06E-235 | 6 | Alox5ap | Macrophages (Mac 1.1) |
| 2.096858 | 0.614 | 0.17 | 4.48E-234 | 6 | Spp1 | Macrophages (Mac 1.1) |
| 0.606774 | 0.664 | 0.18 | 1.53E-233 | 6 | Efhd2 | Macrophages (Mac 1.1) |
| 0.904448 | 0.483 | 0.109 | 2.16E-224 | 6 | Selenop | Macrophages (Mac 1.1) |
| 0.279687 | 0.191 | 0.014 | 1.04E-222 | 6 | Slc7a8 | Macrophages (Mac 1.1) |
| 0.523379 | 0.613 | 0.16 | 2.24E-218 | 6 | Card19 | Macrophages (Mac 1.1) |
| 0.692168 | 0.682 | 0.214 | 1.45E-217 | 6 | Capg | Macrophages (Mac 1.1) |
| 1.560799 | 0.723 | 0.277 | 2.88E-211 | 6 | Fabp5 | Macrophages (Mac 1.1) |
| 0.39007 | 0.72 | 0.192 | 1.04E-206 | 6 | Clec4d | Macrophages (Mac 1.1) |
| 0.663969 | 0.749 | 0.234 | 7.01E-204 | 6 | Slfn2 | Macrophages (Mac 1.1) |
| 0.448072 | 0.409 | 0.079 | 1.13E-203 | 6 | Aif1 | Macrophages (Mac 1.1) |
| 0.445187 | 0.359 | 0.063 | 3.24E-201 | 6 | Blvrb | Macrophages (Mac 1.1) |
| 0.504818 | 0.463 | 0.1 | 1.31E-200 | 6 | Adam8 | Macrophages (Mac 1.1) |
| 0.293781 | 0.2 | 0.019 | 4.02E-198 | 6 | Apoc2 | Macrophages (Mac 1.1) |
| 0.669965 | 0.462 | 0.109 | 5.05E-198 | 6 | Pdpn | Macrophages (Mac 1.1) |
| 0.495338 | 0.462 | 0.103 | 1.97E-197 | 6 | Csf1r | Macrophages (Mac 1.1) |
| 0.593012 | 0.923 | 0.299 | 5.96E-196 | 6 | Tyrobp | Macrophages (Mac 1.1) |
| 0.283763 | 0.228 | 0.025 | 2.22E-195 | 6 | Slc48a1 | Macrophages (Mac 1.1) |
| 0.936705 | 0.854 | 0.434 | 2.36E-193 | 6 | Gpx1 | Macrophages (Mac 1.1) |
| 0.634292 | 0.484 | 0.114 | 6.19E-193 | 6 | Clec4n | Macrophages (Mac 1.1) |
| 0.4254 | 0.423 | 0.091 | 1.28E-183 | 6 | Fcgr3 | Macrophages (Mac 1.1) |
| 1.468513 | 0.872 | 0.571 | 7.76E-183 | 6 | Prdx1 | Macrophages (Mac 1.1) |
| 0.568621 | 0.409 | 0.088 | 7.90E-181 | 6 | Cybb | Macrophages (Mac 1.1) |
| 0.693314 | 0.871 | 0.457 | 3.55E-176 | 6 | Npc2 | Macrophages (Mac 1.1) |
| 0.583441 | 0.692 | 0.253 | 1.54E-175 | 6 | Bri3 | Macrophages (Mac 1.1) |
| 1.606387 | 0.773 | 0.406 | 6.82E-175 | 6 | Ctsl | Macrophages (Mac 1.1) |
| 0.414577 | 0.397 | 0.085 | 7.68E-175 | 6 | Unc93b1 | Macrophages (Mac 1.1) |
| 0.640108 | 0.742 | 0.32 | 1.08E-173 | 6 | Akr1a1 | Macrophages (Mac 1.1) |
| 0.573355 | 0.666 | 0.235 | 1.70E-172 | 6 | Aprt | Macrophages (Mac 1.1) |
| 0.953986 | 0.354 | 0.068 | 6.43E-172 | 6 | Cxcl3 | Macrophages (Mac 1.1) |
| 0.522629 | 0.424 | 0.099 | 7.31E-172 | 6 | Pltp | Macrophages (Mac 1.1) |
| 0.603761 | 0.721 | 0.261 | 1.78E-171 | 6 | Ucp2 | Macrophages (Mac 1.1) |
| 0.51679 | 0.716 | 0.266 | 1.70E-165 | 6 | Atp6v0b | Macrophages (Mac 1.1) |
| 0.479491 | 0.417 | 0.098 | 4.72E-165 | 6 | Itgb2 | Macrophages (Mac 1.1) |
| 0.333786 | 0.268 | 0.042 | 2.09E-164 | 6 | Tcirg1 | Macrophages (Mac 1.1) |
| 0.500434 | 0.536 | 0.16 | 5.25E-163 | 6 | Hexa | Macrophages (Mac 1.1) |
| 0.746817 | 0.591 | 0.208 | 9.01E-163 | 6 | Esd | Macrophages (Mac 1.1) |
| 0.465017 | 0.604 | 0.18 | 9.02E-163 | 6 | Ifi30 | Macrophages (Mac 1.1) |
| 0.547104 | 0.665 | 0.233 | 1.28E-159 | 6 | Atp2b1 | Macrophages (Mac 1.1) |
| 0.491953 | 0.414 | 0.099 | 3.51E-157 | 6 | Tnf | Macrophages (Mac 1.1) |
| 0.470398 | 0.556 | 0.172 | 7.17E-154 | 6 | Cotl1 | Macrophages (Mac 1.1) |
| 0.576939 | 0.386 | 0.088 | 3.96E-153 | 6 | Rgs1 | Macrophages (Mac 1.1) |
| 0.301336 | 0.337 | 0.069 | 9.93E-152 | 6 | Acp5 | Macrophages (Mac 1.1) |
| 0.710829 | 0.836 | 0.458 | 6.71E-151 | 6 | Sh3bgrl3 | Macrophages (Mac 1.1) |
| 0.453102 | 0.54 | 0.158 | 8.51E-151 | 6 | Lilrb4a | Macrophages (Mac 1.1) |
| 0.404056 | 0.366 | 0.082 | 1.81E-150 | 6 | Fcgr2b | Macrophages (Mac 1.1) |
| 0.462079 | 0.731 | 0.258 | 1.61E-149 | 6 | Lcp1 | Macrophages (Mac 1.1) |
| 0.260153 | 0.205 | 0.027 | 2.31E-147 | 6 | Clec4a1 | Macrophages (Mac 1.1) |
| 0.272566 | 0.199 | 0.026 | 3.02E-147 | 6 | Gclm | Macrophages (Mac 1.1) |
| 0.405617 | 0.402 | 0.099 | 5.79E-145 | 6 | Itgam | Macrophages (Mac 1.1) |
| 0.304894 | 0.253 | 0.043 | 1.42E-140 | 6 | Pld3 | Macrophages (Mac 1.1) |
| 0.467174 | 0.464 | 0.13 | 3.34E-140 | 6 | Pla2g7 | Macrophages (Mac 1.1) |
| 0.700712 | 0.84 | 0.469 | 2.30E-135 | 6 | Ahnak | Macrophages (Mac 1.1) |
| 0.507449 | 0.545 | 0.184 | 3.15E-135 | 6 | Atp6v1e1 | Macrophages (Mac 1.1) |
| 0.556886 | 0.391 | 0.102 | 1.39E-134 | 6 | Hilpda | Macrophages (Mac 1.1) |
| 0.499233 | 0.632 | 0.242 | 2.02E-129 | 6 | Zeb2 | Macrophages (Mac 1.1) |
| 0.402453 | 0.161 | 0.019 | 3.80E-129 | 6 | Gdf15 | Macrophages (Mac 1.1) |
| 0.383966 | 0.506 | 0.153 | 5.04E-129 | 6 | C5ar1 | Macrophages (Mac 1.1) |
| 0.275925 | 0.226 | 0.037 | 2.78E-128 | 6 | Ifi207 | Macrophages (Mac 1.1) |
| 0.390717 | 0.387 | 0.104 | 3.47E-125 | 6 | Rilpl2 | Macrophages (Mac 1.1) |
| 0.357968 | 0.319 | 0.075 | 1.80E-122 | 6 | Hexb | Macrophages (Mac 1.1) |
| 0.293844 | 0.205 | 0.033 | 1.44E-121 | 6 | Lhfpl2 | Macrophages (Mac 1.1) |
| 0.330217 | 0.31 | 0.071 | 1.76E-121 | 6 | Clec4a2 | Macrophages (Mac 1.1) |
| 0.511585 | 0.866 | 0.452 | 4.86E-121 | 6 | Mcl1 | Macrophages (Mac 1.1) |
| 0.495733 | 0.746 | 0.347 | 7.74E-120 | 6 | Iqgap1 | Macrophages (Mac 1.1) |
| 0.562601 | 0.54 | 0.18 | 1.13E-119 | 6 | Il1rn | Macrophages (Mac 1.1) |
| 0.270745 | 0.236 | 0.044 | 4.30E-117 | 6 | Tpp1 | Macrophages (Mac 1.1) |
| 0.29925 | 0.289 | 0.066 | 2.56E-113 | 6 | Pid1 | Macrophages (Mac 1.1) |
| 0.392564 | 0.489 | 0.165 | 6.91E-113 | 6 | Slc6a6 | Macrophages (Mac 1.1) |
| 0.391558 | 0.452 | 0.139 | 9.40E-113 | 6 | Tlr2 | Macrophages (Mac 1.1) |
| 0.391958 | 0.453 | 0.145 | 1.46E-112 | 6 | Arl4c | Macrophages (Mac 1.1) |
| 0.276488 | 0.275 | 0.06 | 1.82E-112 | 6 | Slc11a1 | Macrophages (Mac 1.1) |
| 0.598916 | 0.721 | 0.372 | 1.69E-111 | 6 | Pkm | Macrophages (Mac 1.1) |
| 0.307773 | 0.266 | 0.058 | 4.14E-110 | 6 | Ptpre | Macrophages (Mac 1.1) |
| 0.592815 | 0.962 | 0.646 | 3.12E-109 | 6 | B2m | Macrophages (Mac 1.1) |
| 0.271121 | 0.225 | 0.043 | 9.40E-109 | 6 | Pgd | Macrophages (Mac 1.1) |
| 0.352987 | 0.531 | 0.184 | 1.26E-108 | 6 | Spi1 | Macrophages (Mac 1.1) |
| 0.641726 | 0.998 | 0.897 | 1.20E-106 | 6 | Tmsb4x | Macrophages (Mac 1.1) |
| 0.390333 | 0.444 | 0.147 | 2.30E-106 | 6 | Cltc | Macrophages (Mac 1.1) |
| 0.352824 | 0.367 | 0.106 | 2.69E-105 | 6 | Ptpn18 | Macrophages (Mac 1.1) |
| 0.337643 | 0.355 | 0.1 | 4.50E-105 | 6 | Ctsh | Macrophages (Mac 1.1) |
| 0.451413 | 0.381 | 0.112 | 2.65E-104 | 6 | Cd83 | Macrophages (Mac 1.1) |
| 0.2634 | 0.246 | 0.053 | 4.46E-103 | 6 | Sgpl1 | Macrophages (Mac 1.1) |
| 0.615535 | 0.962 | 0.639 | 1.08E-102 | 6 | H2-D1 | Macrophages (Mac 1.1) |
| 0.329112 | 0.382 | 0.114 | 1.26E-101 | 6 | Metrnl | Macrophages (Mac 1.1) |
| 1.079021 | 0.71 | 0.39 | 5.43E-100 | 6 | Cxcl1 | Macrophages (Mac 1.1) |
| 0.28505 | 0.251 | 0.056 | 6.43E-100 | 6 | Ms4a6b | Macrophages (Mac 1.1) |
| 0.535231 | 0.944 | 0.787 | 1.32E-99 | 6 | Pfn1 | Macrophages (Mac 1.1) |
| 0.529731 | 0.933 | 0.721 | 1.54E-99 | 6 | Calm1 | Macrophages (Mac 1.1) |
| 0.250775 | 0.233 | 0.049 | 4.88E-99 | 6 | Pirb | Macrophages (Mac 1.1) |
| 0.352938 | 0.262 | 0.061 | 5.27E-99 | 6 | Mdm2 | Macrophages (Mac 1.1) |
| 0.294475 | 0.268 | 0.064 | 1.39E-98 | 6 | Soat1 | Macrophages (Mac 1.1) |
| 0.307052 | 0.345 | 0.098 | 2.31E-98 | 6 | Rgs10 | Macrophages (Mac 1.1) |
| 0.511763 | 0.732 | 0.357 | 7.33E-98 | 6 | Cd44 | Macrophages (Mac 1.1) |
| 0.265302 | 0.217 | 0.044 | 6.00E-97 | 6 | Fam129b | Macrophages (Mac 1.1) |
| 0.38625 | 0.442 | 0.15 | 3.19E-96 | 6 | Cxcr4 | Macrophages (Mac 1.1) |
| 0.418636 | 0.48 | 0.184 | 1.89E-95 | 6 | Pgam1 | Macrophages (Mac 1.1) |
| 0.430498 | 0.417 | 0.135 | 3.98E-95 | 6 | Wfdc17 | Macrophages (Mac 1.1) |
| 0.550054 | 0.596 | 0.27 | 8.86E-95 | 6 | Ctsc | Macrophages (Mac 1.1) |
| 0.529195 | 0.706 | 0.386 | 2.55E-94 | 6 | Aldoa | Macrophages (Mac 1.1) |
| 0.370741 | 0.489 | 0.186 | 3.51E-91 | 6 | Slc3a2 | Macrophages (Mac 1.1) |
| 0.320319 | 0.357 | 0.111 | 2.80E-89 | 6 | Cflar | Macrophages (Mac 1.1) |
| 0.574888 | 0.592 | 0.285 | 1.17E-87 | 6 | Sqstm1 | Macrophages (Mac 1.1) |
| 0.329542 | 0.408 | 0.135 | 1.56E-87 | 6 | Lilr4b | Macrophages (Mac 1.1) |
| 0.455116 | 0.757 | 0.416 | 3.01E-86 | 6 | Atox1 | Macrophages (Mac 1.1) |
| 0.250482 | 0.577 | 0.221 | 2.47E-85 | 6 | Plek | Macrophages (Mac 1.1) |
| 0.439115 | 0.85 | 0.532 | 3.00E-85 | 6 | Arpc2 | Macrophages (Mac 1.1) |
| 0.486084 | 0.681 | 0.348 | 8.78E-85 | 6 | Emp1 | Macrophages (Mac 1.1) |
| 0.573262 | 0.349 | 0.11 | 1.70E-84 | 6 | Ccl6 | Macrophages (Mac 1.1) |
| 0.41886 | 0.895 | 0.564 | 1.16E-83 | 6 | Cebpb | Macrophages (Mac 1.1) |
| 0.379439 | 0.375 | 0.126 | 2.87E-83 | 6 | Tnfrsf1b | Macrophages (Mac 1.1) |
| 0.366511 | 0.416 | 0.153 | 5.72E-83 | 6 | Rnh1 | Macrophages (Mac 1.1) |
| 0.318057 | 0.375 | 0.127 | 1.53E-82 | 6 | Atp1a1 | Macrophages (Mac 1.1) |
| 0.448406 | 0.532 | 0.233 | 2.07E-82 | 6 | Sdc4 | Macrophages (Mac 1.1) |
| 0.337018 | 0.434 | 0.165 | 8.52E-80 | 6 | Vamp8 | Macrophages (Mac 1.1) |
| 0.606114 | 0.619 | 0.31 | 1.48E-79 | 6 | Tgfbi | Macrophages (Mac 1.1) |
| 0.487865 | 0.803 | 0.523 | 3.68E-79 | 6 | Emp3 | Macrophages (Mac 1.1) |
| 0.255415 | 0.251 | 0.066 | 5.46E-79 | 6 | Gns | Macrophages (Mac 1.1) |
| 0.286995 | 0.311 | 0.095 | 1.66E-78 | 6 | Rbpj | Macrophages (Mac 1.1) |
| 0.396186 | 0.887 | 0.638 | 2.72E-78 | 6 | Cst3 | Macrophages (Mac 1.1) |
| 0.338861 | 0.382 | 0.136 | 3.56E-78 | 6 | BC005537 | Macrophages (Mac 1.1) |
| 0.441277 | 0.815 | 0.512 | 3.03E-76 | 6 | Anxa5 | Macrophages (Mac 1.1) |
| 0.308249 | 0.454 | 0.178 | 1.37E-75 | 6 | Pitpna | Macrophages (Mac 1.1) |
| 0.499927 | 0.258 | 0.073 | 2.36E-75 | 6 | Mmp19 | Macrophages (Mac 1.1) |
| 0.275453 | 0.352 | 0.118 | 3.20E-75 | 6 | Fyb | Macrophages (Mac 1.1) |
| 0.461646 | 0.84 | 0.513 | 9.45E-75 | 6 | H2-K1 | Macrophages (Mac 1.1) |
| 0.39041 | 0.816 | 0.519 | 2.23E-73 | 6 | Cdc42 | Macrophages (Mac 1.1) |
| 0.579508 | 0.881 | 0.587 | 8.60E-73 | 6 | Btg1 | Macrophages (Mac 1.1) |
| 0.414795 | 0.263 | 0.074 | 2.16E-72 | 6 | Ccl4 | Macrophages (Mac 1.1) |
| 0.262333 | 0.773 | 0.376 | 7.43E-71 | 6 | Fxyd5 | Macrophages (Mac 1.1) |
| 0.530743 | 0.849 | 0.524 | 1.35E-68 | 6 | S100a4 | Macrophages (Mac 1.1) |
| 0.34934 | 0.648 | 0.341 | 1.62E-68 | 6 | Atp6v1g1 | Macrophages (Mac 1.1) |
| 0.276659 | 0.309 | 0.102 | 5.32E-68 | 6 | Furin | Macrophages (Mac 1.1) |
| 0.849073 | 0.737 | 0.516 | 1.05E-67 | 6 | Ier3 | Macrophages (Mac 1.1) |
| 0.259391 | 0.261 | 0.078 | 3.11E-67 | 6 | Gngt2 | Macrophages (Mac 1.1) |
| 0.600109 | 0.885 | 0.757 | 1.23E-65 | 6 | Itm2b | Macrophages (Mac 1.1) |
| 0.312148 | 0.414 | 0.168 | 2.62E-65 | 6 | Actr2 | Macrophages (Mac 1.1) |
| 0.497988 | 0.998 | 0.958 | 4.63E-64 | 6 | Actb | Macrophages (Mac 1.1) |
| 0.283503 | 0.527 | 0.234 | 1.21E-63 | 6 | Arhgdib | Macrophages (Mac 1.1) |
| 0.392255 | 0.558 | 0.272 | 2.49E-63 | 6 | Sat1 | Macrophages (Mac 1.1) |
| 0.333315 | 0.377 | 0.15 | 5.30E-63 | 6 | Pgk1 | Macrophages (Mac 1.1) |
| 0.319976 | 0.569 | 0.277 | 9.50E-63 | 6 | Rap1b | Macrophages (Mac 1.1) |
| 0.30381 | 0.458 | 0.201 | 3.12E-62 | 6 | Capza2 | Macrophages (Mac 1.1) |
| 0.355858 | 0.651 | 0.354 | 2.01E-60 | 6 | Lrp1 | Macrophages (Mac 1.1) |
| 0.25372 | 0.262 | 0.084 | 2.39E-60 | 6 | M6pr | Macrophages (Mac 1.1) |
| 0.36105 | 0.701 | 0.425 | 2.73E-60 | 6 | Clta | Macrophages (Mac 1.1) |
| 0.326098 | 0.814 | 0.51 | 1.26E-59 | 6 | Arpc1b | Macrophages (Mac 1.1) |
| 0.427502 | 0.232 | 0.072 | 5.52E-59 | 6 | Lpl | Macrophages (Mac 1.1) |
| 0.312623 | 0.565 | 0.282 | 2.80E-58 | 6 | Arpc5 | Macrophages (Mac 1.1) |
| 0.329687 | 0.632 | 0.352 | 1.10E-57 | 6 | Atp6v0e | Macrophages (Mac 1.1) |
| 0.284081 | 0.491 | 0.233 | 2.28E-54 | 6 | Basp1 | Macrophages (Mac 1.1) |
| 0.259642 | 0.706 | 0.374 | 6.54E-54 | 6 | Pim1 | Macrophages (Mac 1.1) |
| 0.33698 | 0.881 | 0.683 | 2.49E-53 | 6 | Cfl1 | Macrophages (Mac 1.1) |
| 0.297516 | 0.355 | 0.145 | 4.12E-52 | 6 | Txnrd1 | Macrophages (Mac 1.1) |
| 0.292686 | 0.469 | 0.227 | 7.18E-52 | 6 | Arhgdia | Macrophages (Mac 1.1) |
| 0.262059 | 0.274 | 0.098 | 7.97E-51 | 6 | Malt1 | Macrophages (Mac 1.1) |
| 0.266097 | 0.769 | 0.458 | 2.12E-50 | 6 | Arpc3 | Macrophages (Mac 1.1) |
| 0.473083 | 0.794 | 0.593 | 7.70E-49 | 6 | Txn1 | Macrophages (Mac 1.1) |
| 0.319261 | 0.511 | 0.256 | 9.95E-49 | 6 | Tnfaip3 | Macrophages (Mac 1.1) |
| 0.436629 | 0.615 | 0.369 | 8.87E-48 | 6 | Klf6 | Macrophages (Mac 1.1) |
| 0.323219 | 0.663 | 0.394 | 9.63E-48 | 6 | Tspo | Macrophages (Mac 1.1) |
| 0.296225 | 0.268 | 0.102 | 1.62E-46 | 6 | Pmp22 | Macrophages (Mac 1.1) |
| 0.292638 | 0.398 | 0.186 | 9.05E-46 | 6 | Plec | Macrophages (Mac 1.1) |
| 0.259343 | 0.449 | 0.222 | 8.04E-44 | 6 | Atp6v1f | Macrophages (Mac 1.1) |
| 0.335523 | 0.538 | 0.289 | 1.21E-43 | 6 | Cdkn1a | Macrophages (Mac 1.1) |
| 0.413893 | 0.813 | 0.632 | 6.77E-39 | 6 | S100a10 | Macrophages (Mac 1.1) |
| 0.27623 | 0.339 | 0.158 | 1.16E-38 | 6 | Eno1 | Macrophages (Mac 1.1) |
| 0.426034 | 0.757 | 0.605 | 4.90E-38 | 6 | Gapdh | Macrophages (Mac 1.1) |
| 0.29986 | 0.725 | 0.507 | 1.63E-36 | 6 | Eif4a1 | Macrophages (Mac 1.1) |
| 0.367985 | 0.685 | 0.46 | 2.58E-36 | 6 | Zfp36 | Macrophages (Mac 1.1) |
| 0.392062 | 0.397 | 0.215 | 2.34E-32 | 6 | Sgk1 | Macrophages (Mac 1.1) |
| 0.318355 | 0.444 | 0.26 | 6.69E-31 | 6 | Hif1a | Macrophages (Mac 1.1) |
| 0.326495 | 0.546 | 0.344 | 1.22E-30 | 6 | Ifrd1 | Macrophages (Mac 1.1) |
| 0.257805 | 0.428 | 0.244 | 2.16E-28 | 6 | Zfand5 | Macrophages (Mac 1.1) |
| 0.36804 | 0.81 | 0.63 | 4.75E-28 | 6 | Lmna | Macrophages (Mac 1.1) |
| 0.27426 | 0.656 | 0.464 | 1.29E-27 | 6 | Clic1 | Macrophages (Mac 1.1) |
| 0.258044 | 0.411 | 0.232 | 3.85E-27 | 6 | Zfp36l2 | Macrophages (Mac 1.1) |
| 0.306933 | 0.427 | 0.263 | 2.92E-22 | 6 | Thbs1 | Macrophages (Mac 1.1) |
| 0.293147 | 0.991 | 0.826 | 2.94E-21 | 6 | Vim | Macrophages (Mac 1.1) |
| 0.261488 | 0.913 | 0.782 | 2.54E-19 | 6 | Gm42418 | Macrophages (Mac 1.1) |
| 0.485858 | 0.886 | 0.768 | 4.80E-19 | 6 | Crip1 | Macrophages (Mac 1.1) |
| 0.306908 | 0.298 | 0.172 | 2.18E-17 | 6 | Rgcc | Macrophages (Mac 1.1) |
| 0.293397 | 0.598 | 0.453 | 1.48E-13 | 6 | Dusp1 | Macrophages (Mac 1.1) |
| 0.259504 | 0.864 | 0.773 | 2.24E-13 | 6 | Hspa8 | Macrophages (Mac 1.1) |
| 0.349791 | 0.246 | 0.171 | 0.000113 | 6 | Ccl7 | Macrophages (Mac 1.1) |
| 2.635328 | 0.884 | 0.292 | 0 | 7 | Gsn | Fibroblast (Fib 1.4) |
| 1.933869 | 0.603 | 0.056 | 0 | 7 | Apod | Fibroblast (Fib 1.4) |
| 1.919645 | 0.96 | 0.405 | 0 | 7 | Igfbp4 | Fibroblast (Fib 1.4) |
| 1.907611 | 0.797 | 0.086 | 0 | 7 | Plpp3 | Fibroblast (Fib 1.4) |
| 1.587232 | 0.564 | 0.1 | 0 | 7 | Thbs4 | Fibroblast (Fib 1.4) |
| 1.429009 | 0.472 | 0.037 | 0 | 7 | Wif1 | Fibroblast (Fib 1.4) |
| 1.391177 | 0.864 | 0.184 | 0 | 7 | Zbtb20 | Fibroblast (Fib 1.4) |
| 1.337071 | 0.775 | 0.21 | 0 | 7 | Nbl1 | Fibroblast (Fib 1.4) |
| 1.324789 | 0.597 | 0.08 | 0 | 7 | Angptl1 | Fibroblast (Fib 1.4) |
| 0.90752 | 0.326 | 0.027 | 0 | 7 | Pi16 | Fibroblast (Fib 1.4) |
| 0.90068 | 0.36 | 0.029 | 0 | 7 | Clec3b | Fibroblast (Fib 1.4) |
| 0.857195 | 0.424 | 0.052 | 0 | 7 | Chrdl1 | Fibroblast (Fib 1.4) |
| 0.841869 | 0.511 | 0.044 | 0 | 7 | Dpt | Fibroblast (Fib 1.4) |
| 0.729315 | 0.455 | 0.016 | 0 | 7 | Ramp2 | Fibroblast (Fib 1.4) |
| 0.69234 | 0.541 | 0.086 | 0 | 7 | Il11ra1 | Fibroblast (Fib 1.4) |
| 0.614179 | 0.403 | 0.031 | 0 | 7 | Masp1 | Fibroblast (Fib 1.4) |
| 0.601806 | 0.331 | 0.007 | 0 | 7 | Rorb | Fibroblast (Fib 1.4) |
| 0.584508 | 0.325 | 0.019 | 0 | 7 | Cyp26b1 | Fibroblast (Fib 1.4) |
| 0.577107 | 0.28 | 0.013 | 0 | 7 | Slc5a3 | Fibroblast (Fib 1.4) |
| 0.512161 | 0.294 | 0.006 | 0 | 7 | Aldh3a1 | Fibroblast (Fib 1.4) |
| 0.486543 | 0.288 | 0.02 | 0 | 7 | Cd34 | Fibroblast (Fib 1.4) |
| 0.456708 | 0.26 | 0.014 | 0 | 7 | Scara5 | Fibroblast (Fib 1.4) |
| 0.415243 | 0.301 | 0.022 | 0 | 7 | Ptgis | Fibroblast (Fib 1.4) |
| 0.295384 | 0.212 | 0.009 | 0 | 7 | Ntrk2 | Fibroblast (Fib 1.4) |
| 0.340077 | 0.136 | 0.003 | 1.59E-300 | 7 | Pcp4 | Fibroblast (Fib 1.4) |
| 0.528812 | 0.27 | 0.02 | 1.71E-299 | 7 | Apcdd1 | Fibroblast (Fib 1.4) |
| 0.615763 | 0.401 | 0.051 | 2.42E-290 | 7 | Abi3bp | Fibroblast (Fib 1.4) |
| 2.386225 | 0.901 | 0.404 | 2.66E-288 | 7 | Dcn | Fibroblast (Fib 1.4) |
| 0.316214 | 0.22 | 0.013 | 3.74E-284 | 7 | Lama2 | Fibroblast (Fib 1.4) |
| 0.766506 | 0.346 | 0.039 | 8.14E-281 | 7 | C4b | Fibroblast (Fib 1.4) |
| 0.703367 | 0.524 | 0.102 | 1.61E-262 | 7 | Prrx2 | Fibroblast (Fib 1.4) |
| 0.465972 | 0.253 | 0.021 | 2.34E-262 | 7 | Enpp2 | Fibroblast (Fib 1.4) |
| 0.358919 | 0.227 | 0.016 | 1.10E-260 | 7 | Tmem100 | Fibroblast (Fib 1.4) |
| 1.124653 | 0.799 | 0.258 | 1.66E-256 | 7 | Serping1 | Fibroblast (Fib 1.4) |
| 0.83693 | 0.554 | 0.112 | 1.14E-255 | 7 | Ly6a | Fibroblast (Fib 1.4) |
| 1.13397 | 0.919 | 0.355 | 8.52E-254 | 7 | Mmp2 | Fibroblast (Fib 1.4) |
| 0.34399 | 0.212 | 0.015 | 4.32E-240 | 7 | Sema3c | Fibroblast (Fib 1.4) |
| 0.641374 | 0.501 | 0.1 | 1.92E-235 | 7 | Rarres2 | Fibroblast (Fib 1.4) |
| 0.563947 | 0.34 | 0.045 | 2.81E-233 | 7 | Ltbp4 | Fibroblast (Fib 1.4) |
| 0.638897 | 0.527 | 0.114 | 2.31E-228 | 7 | Twist1 | Fibroblast (Fib 1.4) |
| 0.695351 | 0.641 | 0.178 | 3.57E-217 | 7 | Nfix | Fibroblast (Fib 1.4) |
| 0.541258 | 0.316 | 0.042 | 8.08E-216 | 7 | Il33 | Fibroblast (Fib 1.4) |
| 0.467011 | 0.319 | 0.043 | 6.77E-212 | 7 | Mme | Fibroblast (Fib 1.4) |
| 0.508703 | 0.391 | 0.067 | 4.76E-206 | 7 | Prelp | Fibroblast (Fib 1.4) |
| 1.070051 | 0.901 | 0.404 | 4.76E-204 | 7 | Serpinf1 | Fibroblast (Fib 1.4) |
| 0.597993 | 0.578 | 0.151 | 5.89E-201 | 7 | Cd302 | Fibroblast (Fib 1.4) |
| 0.74924 | 0.494 | 0.115 | 4.32E-196 | 7 | Aqp1 | Fibroblast (Fib 1.4) |
| 0.437516 | 0.418 | 0.078 | 6.55E-196 | 7 | Fxyd1 | Fibroblast (Fib 1.4) |
| 1.413547 | 0.446 | 0.097 | 1.10E-195 | 7 | Ecrg4 | Fibroblast (Fib 1.4) |
| 0.332394 | 0.227 | 0.023 | 3.23E-195 | 7 | Dpep1 | Fibroblast (Fib 1.4) |
| 0.936229 | 0.688 | 0.238 | 8.13E-192 | 7 | Ecm1 | Fibroblast (Fib 1.4) |
| 0.490551 | 0.266 | 0.033 | 1.30E-190 | 7 | Cp | Fibroblast (Fib 1.4) |
| 1.210713 | 0.445 | 0.095 | 3.17E-188 | 7 | Eln | Fibroblast (Fib 1.4) |
| 0.532134 | 0.499 | 0.119 | 1.33E-184 | 7 | Bicc1 | Fibroblast (Fib 1.4) |
| 0.327791 | 0.184 | 0.016 | 1.95E-179 | 7 | Serpina3n | Fibroblast (Fib 1.4) |
| 0.795388 | 0.732 | 0.26 | 5.07E-179 | 7 | Htra1 | Fibroblast (Fib 1.4) |
| 0.74518 | 0.564 | 0.155 | 2.11E-178 | 7 | Igf1 | Fibroblast (Fib 1.4) |
| 0.847974 | 0.476 | 0.115 | 5.64E-176 | 7 | Timp3 | Fibroblast (Fib 1.4) |
| 0.638814 | 0.41 | 0.086 | 2.78E-174 | 7 | Gas6 | Fibroblast (Fib 1.4) |
| 0.601275 | 0.619 | 0.193 | 3.05E-172 | 7 | Cyb5a | Fibroblast (Fib 1.4) |
| 0.887806 | 0.917 | 0.5 | 1.32E-171 | 7 | Timp2 | Fibroblast (Fib 1.4) |
| 0.551076 | 0.486 | 0.121 | 1.31E-169 | 7 | Sod3 | Fibroblast (Fib 1.4) |
| 0.633739 | 0.465 | 0.112 | 1.39E-169 | 7 | Fbln1 | Fibroblast (Fib 1.4) |
| 0.397443 | 0.322 | 0.055 | 3.89E-168 | 7 | C1ra | Fibroblast (Fib 1.4) |
| 0.357948 | 0.28 | 0.041 | 4.79E-168 | 7 | Tpbg | Fibroblast (Fib 1.4) |
| 0.390832 | 0.332 | 0.058 | 8.43E-168 | 7 | Nfia | Fibroblast (Fib 1.4) |
| 0.275184 | 0.206 | 0.022 | 2.68E-167 | 7 | Nr1d1 | Fibroblast (Fib 1.4) |
| 1.145254 | 0.551 | 0.176 | 1.20E-165 | 7 | Vcan | Fibroblast (Fib 1.4) |
| 0.715887 | 0.648 | 0.221 | 6.65E-165 | 7 | Klf9 | Fibroblast (Fib 1.4) |
| 0.283532 | 0.12 | 0.006 | 5.80E-164 | 7 | Stmn2 | Fibroblast (Fib 1.4) |
| 0.773747 | 0.787 | 0.342 | 1.87E-162 | 7 | Ctsk | Fibroblast (Fib 1.4) |
| 0.427739 | 0.393 | 0.082 | 3.06E-160 | 7 | Prnp | Fibroblast (Fib 1.4) |
| 0.432588 | 0.386 | 0.082 | 1.12E-157 | 7 | Pam | Fibroblast (Fib 1.4) |
| 0.556228 | 0.544 | 0.16 | 8.62E-157 | 7 | Pdgfra | Fibroblast (Fib 1.4) |
| 1.041498 | 0.891 | 0.388 | 1.40E-155 | 7 | Lum | Fibroblast (Fib 1.4) |
| 0.568426 | 0.458 | 0.121 | 2.18E-147 | 7 | Mdk | Fibroblast (Fib 1.4) |
| 0.386081 | 0.363 | 0.076 | 9.68E-145 | 7 | Crispld2 | Fibroblast (Fib 1.4) |
| 0.372126 | 0.316 | 0.063 | 1.46E-137 | 7 | Shox2 | Fibroblast (Fib 1.4) |
| 0.870769 | 0.867 | 0.513 | 8.42E-137 | 7 | Cd9 | Fibroblast (Fib 1.4) |
| 0.627692 | 0.802 | 0.394 | 1.31E-136 | 7 | Cd81 | Fibroblast (Fib 1.4) |
| 0.525951 | 0.425 | 0.111 | 1.57E-135 | 7 | C1s1 | Fibroblast (Fib 1.4) |
| 0.261275 | 0.177 | 0.021 | 7.90E-129 | 7 | Cfb | Fibroblast (Fib 1.4) |
| 0.393296 | 0.21 | 0.03 | 1.75E-128 | 7 | Tnmd | Fibroblast (Fib 1.4) |
| 0.839181 | 0.499 | 0.157 | 4.88E-128 | 7 | Crabp1 | Fibroblast (Fib 1.4) |
| 0.832166 | 0.904 | 0.488 | 3.55E-126 | 7 | Igfbp7 | Fibroblast (Fib 1.4) |
| 0.756741 | 0.544 | 0.193 | 8.91E-124 | 7 | Gem | Fibroblast (Fib 1.4) |
| 0.803936 | 0.486 | 0.167 | 1.17E-120 | 7 | Slc6a6 | Fibroblast (Fib 1.4) |
| 0.583145 | 0.517 | 0.173 | 1.53E-120 | 7 | Gas1 | Fibroblast (Fib 1.4) |
| 0.286683 | 0.249 | 0.044 | 4.36E-120 | 7 | Zfhx4 | Fibroblast (Fib 1.4) |
| 0.341064 | 0.198 | 0.028 | 2.71E-119 | 7 | Gpc3 | Fibroblast (Fib 1.4) |
| 0.450501 | 0.451 | 0.135 | 5.04E-118 | 7 | Rnase4 | Fibroblast (Fib 1.4) |
| 0.72204 | 0.451 | 0.144 | 5.04E-115 | 7 | Igfbp6 | Fibroblast (Fib 1.4) |
| 0.612615 | 0.736 | 0.352 | 7.03E-115 | 7 | Lrp1 | Fibroblast (Fib 1.4) |
| 0.266968 | 0.23 | 0.039 | 7.39E-115 | 7 | Clmp | Fibroblast (Fib 1.4) |
| 0.66631 | 0.41 | 0.121 | 2.20E-114 | 7 | Tsc22d3 | Fibroblast (Fib 1.4) |
| 0.40914 | 0.374 | 0.099 | 1.56E-112 | 7 | Nfib | Fibroblast (Fib 1.4) |
| 0.755275 | 0.802 | 0.386 | 1.86E-112 | 7 | Aebp1 | Fibroblast (Fib 1.4) |
| 0.371571 | 0.331 | 0.08 | 2.20E-110 | 7 | Pbx1 | Fibroblast (Fib 1.4) |
| 0.89377 | 0.88 | 0.525 | 6.03E-110 | 7 | S100a4 | Fibroblast (Fib 1.4) |
| 0.410809 | 0.377 | 0.102 | 1.66E-109 | 7 | Itgbl1 | Fibroblast (Fib 1.4) |
| 0.947795 | 0.566 | 0.225 | 9.97E-109 | 7 | Mt2 | Fibroblast (Fib 1.4) |
| 0.761794 | 0.976 | 0.513 | 1.09E-105 | 7 | Bgn | Fibroblast (Fib 1.4) |
| 0.895797 | 0.623 | 0.276 | 1.74E-105 | 7 | Cebpd | Fibroblast (Fib 1.4) |
| 0.580338 | 0.681 | 0.319 | 2.16E-105 | 7 | Prrx1 | Fibroblast (Fib 1.4) |
| 0.267815 | 0.21 | 0.036 | 4.85E-104 | 7 | Gstm1 | Fibroblast (Fib 1.4) |
| 0.337171 | 0.282 | 0.063 | 2.37E-102 | 7 | Gpm6b | Fibroblast (Fib 1.4) |
| 0.656743 | 0.314 | 0.08 | 7.12E-102 | 7 | C3 | Fibroblast (Fib 1.4) |
| 0.901416 | 0.429 | 0.149 | 1.00E-97 | 7 | Mfap4 | Fibroblast (Fib 1.4) |
| 0.462493 | 0.284 | 0.068 | 4.10E-97 | 7 | Cfh | Fibroblast (Fib 1.4) |
| 0.387968 | 0.242 | 0.051 | 3.16E-95 | 7 | Ltbp2 | Fibroblast (Fib 1.4) |
| 0.694953 | 0.976 | 0.866 | 3.13E-94 | 7 | mt-Cytb | Fibroblast (Fib 1.4) |
| 0.26008 | 0.223 | 0.045 | 1.50E-90 | 7 | Socs2 | Fibroblast (Fib 1.4) |
| 0.513226 | 0.516 | 0.204 | 4.83E-90 | 7 | Mfap2 | Fibroblast (Fib 1.4) |
| 0.722076 | 0.678 | 0.33 | 6.55E-89 | 7 | Id3 | Fibroblast (Fib 1.4) |
| 0.301425 | 0.243 | 0.054 | 3.58E-88 | 7 | Ltbp1 | Fibroblast (Fib 1.4) |
| 0.540387 | 0.605 | 0.278 | 5.66E-88 | 7 | mt-Nd4l | Fibroblast (Fib 1.4) |
| 0.4231 | 0.359 | 0.11 | 1.08E-84 | 7 | Wnt5a | Fibroblast (Fib 1.4) |
| 0.424968 | 0.559 | 0.242 | 2.51E-84 | 7 | Nenf | Fibroblast (Fib 1.4) |
| 0.592568 | 0.886 | 0.663 | 2.41E-82 | 7 | mt-Nd2 | Fibroblast (Fib 1.4) |
| 0.654025 | 0.701 | 0.359 | 8.91E-82 | 7 | Nupr1 | Fibroblast (Fib 1.4) |
| 0.322478 | 0.305 | 0.086 | 2.87E-81 | 7 | Fbln5 | Fibroblast (Fib 1.4) |
| 0.362181 | 0.292 | 0.08 | 4.06E-81 | 7 | Spon1 | Fibroblast (Fib 1.4) |
| 1.477868 | 0.407 | 0.152 | 1.03E-77 | 7 | Igfbp5 | Fibroblast (Fib 1.4) |
| 0.255953 | 0.333 | 0.1 | 8.84E-77 | 7 | Pmp22 | Fibroblast (Fib 1.4) |
| 0.337308 | 0.208 | 0.045 | 1.17E-75 | 7 | Matn4 | Fibroblast (Fib 1.4) |
| 0.255245 | 0.243 | 0.06 | 1.57E-74 | 7 | Mxra7 | Fibroblast (Fib 1.4) |
| 0.452545 | 0.792 | 0.494 | 1.49E-72 | 7 | Laptm4a | Fibroblast (Fib 1.4) |
| 0.266128 | 0.157 | 0.028 | 1.30E-71 | 7 | Gdf10 | Fibroblast (Fib 1.4) |
| 0.354346 | 0.412 | 0.154 | 1.59E-71 | 7 | Itm2c | Fibroblast (Fib 1.4) |
| 0.541287 | 0.657 | 0.348 | 3.56E-71 | 7 | Pcolce | Fibroblast (Fib 1.4) |
| 0.452323 | 0.565 | 0.256 | 2.56E-70 | 7 | Fbln2 | Fibroblast (Fib 1.4) |
| 0.541949 | 0.999 | 0.923 | 1.12E-69 | 7 | mt-Atp6 | Fibroblast (Fib 1.4) |
| 0.457782 | 0.54 | 0.251 | 1.61E-69 | 7 | Tcf4 | Fibroblast (Fib 1.4) |
| 0.475368 | 0.99 | 0.925 | 4.45E-69 | 7 | mt-Co2 | Fibroblast (Fib 1.4) |
| 0.6265 | 0.638 | 0.348 | 1.55E-68 | 7 | Zfp36l1 | Fibroblast (Fib 1.4) |
| 0.729784 | 0.86 | 0.641 | 3.80E-68 | 7 | Cst3 | Fibroblast (Fib 1.4) |
| 0.359076 | 0.727 | 0.411 | 1.12E-67 | 7 | Ctsl | Fibroblast (Fib 1.4) |
| 0.264919 | 0.25 | 0.068 | 3.66E-67 | 7 | Nrp1 | Fibroblast (Fib 1.4) |
| 0.454282 | 0.449 | 0.18 | 4.90E-67 | 7 | Cpxm1 | Fibroblast (Fib 1.4) |
| 0.289255 | 0.288 | 0.085 | 2.08E-66 | 7 | Col15a1 | Fibroblast (Fib 1.4) |
| 0.289178 | 0.345 | 0.117 | 2.75E-66 | 7 | Fcgrt | Fibroblast (Fib 1.4) |
| 1.104711 | 0.725 | 0.462 | 3.41E-66 | 7 | Mt1 | Fibroblast (Fib 1.4) |
| 0.505336 | 0.911 | 0.801 | 3.97E-66 | 7 | mt-Nd4 | Fibroblast (Fib 1.4) |
| 0.516901 | 0.993 | 0.925 | 2.28E-65 | 7 | mt-Co3 | Fibroblast (Fib 1.4) |
| 0.491055 | 0.514 | 0.236 | 3.98E-64 | 7 | Sdc4 | Fibroblast (Fib 1.4) |
| 0.393845 | 0.186 | 0.043 | 4.36E-64 | 7 | Has1 | Fibroblast (Fib 1.4) |
| 0.270543 | 0.278 | 0.085 | 3.20E-63 | 7 | Fuca1 | Fibroblast (Fib 1.4) |
| 0.345042 | 0.371 | 0.14 | 1.42E-61 | 7 | Sptbn1 | Fibroblast (Fib 1.4) |
| 0.771005 | 0.349 | 0.134 | 3.24E-61 | 7 | Fmod | Fibroblast (Fib 1.4) |
| 0.344555 | 0.353 | 0.13 | 3.36E-60 | 7 | Cavin1 | Fibroblast (Fib 1.4) |
| 0.378214 | 0.219 | 0.06 | 1.99E-59 | 7 | Col14a1 | Fibroblast (Fib 1.4) |
| 0.452671 | 0.852 | 0.627 | 7.37E-59 | 7 | Gnas | Fibroblast (Fib 1.4) |
| 0.39826 | 0.469 | 0.213 | 2.47E-58 | 7 | Ccdc80 | Fibroblast (Fib 1.4) |
| 0.705537 | 0.977 | 0.973 | 4.12E-58 | 7 | Malat1 | Fibroblast (Fib 1.4) |
| 0.268688 | 0.282 | 0.091 | 4.15E-58 | 7 | Atraid | Fibroblast (Fib 1.4) |
| 0.395827 | 0.681 | 0.373 | 2.64E-57 | 7 | Selenom | Fibroblast (Fib 1.4) |
| 0.330157 | 0.448 | 0.193 | 4.73E-57 | 7 | Fgfr1 | Fibroblast (Fib 1.4) |
| 0.395499 | 0.501 | 0.241 | 3.84E-55 | 7 | Mxra8 | Fibroblast (Fib 1.4) |
| 0.324701 | 0.356 | 0.14 | 1.16E-53 | 7 | Pnp | Fibroblast (Fib 1.4) |
| 0.748466 | 0.549 | 0.309 | 4.08E-53 | 7 | Egr1 | Fibroblast (Fib 1.4) |
| 0.559784 | 0.78 | 0.448 | 5.36E-53 | 7 | Postn | Fibroblast (Fib 1.4) |
| 0.343832 | 0.499 | 0.241 | 1.32E-52 | 7 | Cavin3 | Fibroblast (Fib 1.4) |
| 0.41305 | 0.744 | 0.495 | 1.35E-52 | 7 | mt-Nd3 | Fibroblast (Fib 1.4) |
| 0.492757 | 0.275 | 0.094 | 2.44E-52 | 7 | Tnfaip6 | Fibroblast (Fib 1.4) |
| 0.255599 | 0.281 | 0.096 | 5.56E-52 | 7 | Sdf4 | Fibroblast (Fib 1.4) |
| 0.322681 | 0.463 | 0.217 | 3.20E-51 | 7 | Eid1 | Fibroblast (Fib 1.4) |
| 0.367924 | 0.304 | 0.111 | 2.93E-50 | 7 | Ccn1 | Fibroblast (Fib 1.4) |
| 0.403171 | 0.893 | 0.758 | 1.51E-49 | 7 | Itm2b | Fibroblast (Fib 1.4) |
| 0.386408 | 0.994 | 0.941 | 2.55E-48 | 7 | mt-Co1 | Fibroblast (Fib 1.4) |
| 0.269216 | 0.309 | 0.117 | 3.20E-48 | 7 | Fkbp7 | Fibroblast (Fib 1.4) |
| 0.305193 | 0.47 | 0.219 | 3.03E-46 | 7 | Col18a1 | Fibroblast (Fib 1.4) |
| 0.419217 | 0.869 | 0.627 | 3.38E-46 | 7 | Ifitm3 | Fibroblast (Fib 1.4) |
| 0.275051 | 0.23 | 0.077 | 4.23E-43 | 7 | Cygb | Fibroblast (Fib 1.4) |
| 0.411409 | 0.288 | 0.115 | 8.64E-42 | 7 | Ccn5 | Fibroblast (Fib 1.4) |
| 0.505813 | 0.35 | 0.162 | 8.83E-40 | 7 | Tnc | Fibroblast (Fib 1.4) |
| 0.465273 | 0.131 | 0.031 | 8.93E-40 | 7 | Mmp3 | Fibroblast (Fib 1.4) |
| 0.653125 | 0.648 | 0.448 | 1.59E-39 | 7 | Fosb | Fibroblast (Fib 1.4) |
| 0.324835 | 0.418 | 0.215 | 5.51E-37 | 7 | Olfml3 | Fibroblast (Fib 1.4) |
| 0.610084 | 0.448 | 0.234 | 9.13E-37 | 7 | Serpine2 | Fibroblast (Fib 1.4) |
| 0.515068 | 0.497 | 0.292 | 1.27E-35 | 7 | Klf4 | Fibroblast (Fib 1.4) |
| 0.361888 | 0.908 | 0.782 | 1.37E-34 | 7 | mt-Nd1 | Fibroblast (Fib 1.4) |
| 0.253594 | 0.376 | 0.183 | 3.25E-34 | 7 | Oat | Fibroblast (Fib 1.4) |
| 0.535431 | 0.531 | 0.344 | 1.04E-32 | 7 | Nr4a1 | Fibroblast (Fib 1.4) |
| 0.422769 | 0.973 | 0.847 | 5.66E-32 | 7 | S100a6 | Fibroblast (Fib 1.4) |
| 0.307881 | 0.465 | 0.268 | 7.01E-32 | 7 | Pebp1 | Fibroblast (Fib 1.4) |
| 0.256266 | 0.357 | 0.175 | 8.83E-32 | 7 | S100a13 | Fibroblast (Fib 1.4) |
| 0.267377 | 0.479 | 0.27 | 1.19E-31 | 7 | Tmem59 | Fibroblast (Fib 1.4) |
| 0.299755 | 0.14 | 0.041 | 4.57E-31 | 7 | Ccl11 | Fibroblast (Fib 1.4) |
| 0.349149 | 0.739 | 0.54 | 1.36E-30 | 7 | Anxa1 | Fibroblast (Fib 1.4) |
| 0.435894 | 0.319 | 0.16 | 3.87E-29 | 7 | Tnn | Fibroblast (Fib 1.4) |
| 0.462143 | 0.305 | 0.148 | 3.20E-28 | 7 | Cxcl12 | Fibroblast (Fib 1.4) |
| 0.299181 | 0.644 | 0.458 | 6.29E-28 | 7 | Selenow | Fibroblast (Fib 1.4) |
| 0.359419 | 0.684 | 0.479 | 3.00E-27 | 7 | Ahnak | Fibroblast (Fib 1.4) |
| 0.255567 | 0.503 | 0.316 | 2.73E-24 | 7 | Rabac1 | Fibroblast (Fib 1.4) |
| 0.260683 | 0.599 | 0.398 | 3.56E-24 | 7 | Nedd4 | Fibroblast (Fib 1.4) |
| 0.370539 | 0.297 | 0.158 | 2.04E-20 | 7 | Lars2 | Fibroblast (Fib 1.4) |
| 0.418926 | 0.582 | 0.405 | 4.50E-20 | 7 | Jun | Fibroblast (Fib 1.4) |
| 0.260475 | 0.328 | 0.175 | 2.58E-19 | 7 | Mfap5 | Fibroblast (Fib 1.4) |
| 0.535543 | 0.884 | 0.784 | 2.85E-19 | 7 | Gm42418 | Fibroblast (Fib 1.4) |
| 0.712464 | 0.603 | 0.472 | 8.40E-19 | 7 | Fos | Fibroblast (Fib 1.4) |
| 0.260356 | 0.692 | 0.53 | 9.30E-19 | 7 | Emp3 | Fibroblast (Fib 1.4) |
| 0.273268 | 0.475 | 0.313 | 1.97E-18 | 7 | Slc38a2 | Fibroblast (Fib 1.4) |
| 0.256039 | 0.589 | 0.435 | 3.99E-18 | 7 | Reep5 | Fibroblast (Fib 1.4) |
| 0.343975 | 0.88 | 0.867 | 2.88E-17 | 7 | Ubb | Fibroblast (Fib 1.4) |
| 0.276923 | 0.794 | 0.632 | 1.53E-12 | 7 | Lmna | Fibroblast (Fib 1.4) |
| 0.305435 | 0.804 | 0.745 | 1.46E-10 | 7 | Ubc | Fibroblast (Fib 1.4) |
| 0.253147 | 0.316 | 0.203 | 1.99E-10 | 7 | Nr4a2 | Fibroblast (Fib 1.4) |
| 0.332767 | 0.493 | 0.34 | 3.23E-10 | 7 | Col12a1 | Fibroblast (Fib 1.4) |
| 0.283866 | 0.124 | 0.061 | 1.45E-07 | 7 | Cxcl14 | Fibroblast (Fib 1.4) |
| 0.505272 | 0.203 | 0.137 | 0.001366 | 7 | Sfrp2 | Fibroblast (Fib 1.4) |
| 2.74833 | 1 | 0.146 | 0 | 8 | Cd74 | B_Cells |
| 2.496346 | 0.886 | 0.009 | 0 | 8 | Igkc | B_Cells |
| 2.186193 | 1 | 0.108 | 0 | 8 | H2-Ab1 | B_Cells |
| 2.12139 | 0.996 | 0.109 | 0 | 8 | H2-Aa | B_Cells |
| 2.009369 | 0.89 | 0.007 | 0 | 8 | Cd79a | B_Cells |
| 1.548012 | 0.999 | 0.942 | 0 | 8 | Rps27 | B_Cells |
| 1.492951 | 0.986 | 0.757 | 0 | 8 | Rpl12 | B_Cells |
| 1.406617 | 0.987 | 0.8 | 0 | 8 | Rpl13a | B_Cells |
| 1.363238 | 0.999 | 0.899 | 0 | 8 | Rps24 | B_Cells |
| 1.300336 | 0.996 | 0.895 | 0 | 8 | Rpl13 | B_Cells |
| 1.300021 | 0.991 | 0.842 | 0 | 8 | Rps5 | B_Cells |
| 1.295768 | 0.986 | 0.835 | 0 | 8 | Rps7 | B_Cells |
| 1.286839 | 0.991 | 0.869 | 0 | 8 | Rpl18a | B_Cells |
| 1.286279 | 0.993 | 0.849 | 0 | 8 | Rpl8 | B_Cells |
| 1.280754 | 0.991 | 0.846 | 0 | 8 | Rps13 | B_Cells |
| 1.270823 | 0.991 | 0.835 | 0 | 8 | Rpl17 | B_Cells |
| 1.257304 | 1 | 0.968 | 0 | 8 | Fau | B_Cells |
| 1.248921 | 1 | 0.972 | 0 | 8 | Rps29 | B_Cells |
| 1.091392 | 0.792 | 0.177 | 0 | 8 | Ebf1 | B_Cells |
| 0.988163 | 0.63 | 0.08 | 0 | 8 | Mef2c | B_Cells |
| 0.917886 | 0.422 | 0.05 | 0 | 8 | H2-Eb1 | B_Cells |
| 0.803933 | 0.568 | 0.05 | 0 | 8 | Cd37 | B_Cells |
| 0.792905 | 0.515 | 0.03 | 0 | 8 | Satb1 | B_Cells |
| 0.785231 | 0.566 | 0.059 | 0 | 8 | Napsa | B_Cells |
| 0.753756 | 0.428 | 0.003 | 0 | 8 | Cd79b | B_Cells |
| 0.729523 | 0.469 | 0.012 | 0 | 8 | Ighm | B_Cells |
| 0.686985 | 0.518 | 0.049 | 0 | 8 | H2-DMa | B_Cells |
| 0.663144 | 0.472 | 0.03 | 0 | 8 | Ccr7 | B_Cells |
| 0.618326 | 0.41 | 0.001 | 0 | 8 | Ighd | B_Cells |
| 0.556271 | 0.394 | 0.015 | 0 | 8 | H2-DMb2 | B_Cells |
| 0.550785 | 0.358 | 0.001 | 0 | 8 | Fcrla | B_Cells |
| 0.527168 | 0.349 | 0.012 | 0 | 8 | Scd1 | B_Cells |
| 0.496386 | 0.333 | 0.003 | 0 | 8 | H2-Oa | B_Cells |
| 0.493454 | 0.307 | 0.002 | 0 | 8 | Mzb1 | B_Cells |
| 0.458818 | 0.309 | 0.001 | 0 | 8 | Fcmr | B_Cells |
| 0.457891 | 0.334 | 0.021 | 0 | 8 | Ptprcap | B_Cells |
| 0.457813 | 0.256 | 0.004 | 0 | 8 | Ly6d | B_Cells |
| 0.452642 | 0.293 | 0.001 | 0 | 8 | Tnfrsf13c | B_Cells |
| 0.420833 | 0.266 | 0.001 | 0 | 8 | Ms4a1 | B_Cells |
| 0.380211 | 0.265 | 0.013 | 0 | 8 | Arap2 | B_Cells |
| 0.364347 | 0.249 | 0.012 | 0 | 8 | Ppp1r16b | B_Cells |
| 0.362731 | 0.243 | 0.001 | 0 | 8 | H2-Ob | B_Cells |
| 0.358429 | 0.243 | 0.001 | 0 | 8 | Bank1 | B_Cells |
| 0.302409 | 0.213 | 0 | 0 | 8 | Cd19 | B_Cells |
| 0.29855 | 0.219 | 0.011 | 0 | 8 | Plekha2 | B_Cells |
| 1.157662 | 0.997 | 0.879 | 2.55E-300 | 8 | Rpl19 | B_Cells |
| 1.131918 | 0.997 | 0.948 | 3.02E-300 | 8 | Rps9 | B_Cells |
| 1.231147 | 0.99 | 0.866 | 1.60E-299 | 8 | Rpl9 | B_Cells |
| 1.193174 | 0.991 | 0.833 | 1.18E-294 | 8 | Rps3 | B_Cells |
| 1.170549 | 0.996 | 0.841 | 2.64E-283 | 8 | Rps19 | B_Cells |
| 0.333701 | 0.248 | 0.017 | 1.38E-279 | 8 | Gimap6 | B_Cells |
| 1.207091 | 0.993 | 0.841 | 8.34E-276 | 8 | Rps4x | B_Cells |
| 0.343349 | 0.276 | 0.024 | 6.56E-268 | 8 | Ly86 | B_Cells |
| 0.460708 | 0.367 | 0.045 | 1.25E-266 | 8 | Cd86 | B_Cells |
| 0.335038 | 0.255 | 0.02 | 1.08E-264 | 8 | Il21r | B_Cells |
| 1.166361 | 0.997 | 0.854 | 3.60E-264 | 8 | Rpsa | B_Cells |
| 0.595929 | 0.469 | 0.077 | 5.36E-263 | 8 | Crem | B_Cells |
| 1.112412 | 0.855 | 0.342 | 4.65E-257 | 8 | Sub1 | B_Cells |
| 1.009917 | 0.996 | 0.881 | 5.46E-251 | 8 | Rps10 | B_Cells |
| 0.996553 | 0.999 | 0.979 | 1.61E-250 | 8 | Tpt1 | B_Cells |
| 1.074737 | 0.959 | 0.658 | 1.37E-244 | 8 | Rpl5 | B_Cells |
| 1.098968 | 0.959 | 0.703 | 5.83E-244 | 8 | Rps6 | B_Cells |
| 1.041183 | 0.983 | 0.828 | 1.18E-243 | 8 | Rpl21 | B_Cells |
| 0.314929 | 0.232 | 0.018 | 2.00E-243 | 8 | P2ry10 | B_Cells |
| 1.075415 | 0.991 | 0.81 | 8.53E-243 | 8 | Rpl7 | B_Cells |
| 1.054431 | 0.993 | 0.868 | 3.31E-238 | 8 | Rps20 | B_Cells |
| 0.662437 | 0.494 | 0.092 | 7.57E-238 | 8 | Vps37b | B_Cells |
| 0.916262 | 0.993 | 0.908 | 2.15E-230 | 8 | Rps16 | B_Cells |
| 0.322218 | 0.253 | 0.024 | 1.45E-224 | 8 | Cd72 | B_Cells |
| 1.058664 | 0.984 | 0.757 | 2.91E-224 | 8 | Rpl3 | B_Cells |
| 0.418065 | 0.323 | 0.042 | 1.63E-223 | 8 | Cirbp | B_Cells |
| 0.311311 | 0.226 | 0.019 | 2.00E-221 | 8 | Stat4 | B_Cells |
| 0.931494 | 0.805 | 0.287 | 5.58E-221 | 8 | Ly6e | B_Cells |
| 0.768328 | 0.56 | 0.136 | 2.92E-216 | 8 | Rel | B_Cells |
| 0.964732 | 0.99 | 0.846 | 6.41E-214 | 8 | Rpl32 | B_Cells |
| 0.979323 | 0.973 | 0.752 | 3.07E-209 | 8 | Rack1 | B_Cells |
| 0.920138 | 0.99 | 0.881 | 4.98E-209 | 8 | Rpl27a | B_Cells |
| 0.915419 | 0.993 | 0.857 | 2.86E-205 | 8 | Rps11 | B_Cells |
| 0.642158 | 0.505 | 0.108 | 7.99E-204 | 8 | Cd83 | B_Cells |
| 0.566093 | 0.448 | 0.091 | 1.36E-202 | 8 | Pold4 | B_Cells |
| 0.885779 | 0.994 | 0.856 | 1.92E-201 | 8 | Rpl6 | B_Cells |
| 0.329846 | 0.216 | 0.02 | 3.88E-199 | 8 | Dmxl1 | B_Cells |
| 0.88188 | 0.99 | 0.907 | 5.40E-198 | 8 | Rps21 | B_Cells |
| 0.870276 | 0.987 | 0.834 | 2.14E-197 | 8 | Rpl26 | B_Cells |
| 0.894753 | 0.986 | 0.851 | 2.84E-196 | 8 | Rpl18 | B_Cells |
| 0.833985 | 0.969 | 0.791 | 5.08E-191 | 8 | Rpl27 | B_Cells |
| 0.830415 | 0.982 | 0.848 | 4.14E-186 | 8 | Rps14 | B_Cells |
| 0.816275 | 0.996 | 0.915 | 1.44E-185 | 8 | Rpl23 | B_Cells |
| 0.903554 | 0.972 | 0.823 | 2.76E-184 | 8 | Rpl10 | B_Cells |
| 0.865134 | 0.987 | 0.853 | 4.68E-182 | 8 | Rps15a | B_Cells |
| 0.624297 | 0.542 | 0.147 | 8.15E-180 | 8 | Srsf7 | B_Cells |
| 0.913524 | 0.974 | 0.859 | 1.63E-179 | 8 | H3f3a | B_Cells |
| 0.823209 | 0.993 | 0.882 | 7.84E-173 | 8 | Rps3a1 | B_Cells |
| 0.759163 | 0.997 | 0.907 | 3.17E-170 | 8 | Rps28 | B_Cells |
| 0.838081 | 0.977 | 0.828 | 3.25E-170 | 8 | Rpl30 | B_Cells |
| 0.80774 | 0.982 | 0.839 | 3.20E-168 | 8 | Rpl11 | B_Cells |
| 0.381574 | 0.314 | 0.052 | 3.68E-167 | 8 | Inpp5d | B_Cells |
| 0.557644 | 0.546 | 0.146 | 8.04E-167 | 8 | Cytip | B_Cells |
| 0.34161 | 0.269 | 0.039 | 2.27E-162 | 8 | Cnot6l | B_Cells |
| 0.252215 | 0.193 | 0.02 | 3.06E-162 | 8 | Gramd3 | B_Cells |
| 0.503727 | 0.435 | 0.102 | 3.71E-161 | 8 | Kras | B_Cells |
| 0.722344 | 0.994 | 0.926 | 3.79E-160 | 8 | Eef1a1 | B_Cells |
| 0.743271 | 0.993 | 0.899 | 4.08E-160 | 8 | Rpl38 | B_Cells |
| 0.781523 | 1 | 0.923 | 2.36E-158 | 8 | mt-Atp6 | B_Cells |
| 0.317469 | 0.257 | 0.037 | 1.59E-156 | 8 | Arhgap45 | B_Cells |
| 0.765634 | 0.98 | 0.838 | 3.03E-156 | 8 | Rplp2 | B_Cells |
| 0.259656 | 0.211 | 0.025 | 6.51E-154 | 8 | Rps27rt | B_Cells |
| 0.261324 | 0.206 | 0.024 | 2.23E-151 | 8 | Ppm1g | B_Cells |
| 0.891968 | 0.915 | 0.662 | 2.08E-149 | 8 | mt-Nd2 | B_Cells |
| 0.828247 | 0.964 | 0.784 | 5.39E-148 | 8 | Rpl10a | B_Cells |
| 0.765965 | 0.986 | 0.873 | 1.65E-147 | 8 | Rplp1 | B_Cells |
| 0.796599 | 0.908 | 0.512 | 1.06E-146 | 8 | H2-K1 | B_Cells |
| 0.781249 | 0.986 | 0.893 | 7.86E-142 | 8 | H3f3b | B_Cells |
| 0.558219 | 0.521 | 0.159 | 1.57E-141 | 8 | Shisa5 | B_Cells |
| 0.361586 | 0.286 | 0.05 | 2.00E-141 | 8 | Tut4 | B_Cells |
| 0.706327 | 0.987 | 0.859 | 1.27E-138 | 8 | Rps23 | B_Cells |
| 0.267975 | 0.225 | 0.032 | 2.15E-138 | 8 | Ttc14 | B_Cells |
| 0.741884 | 0.947 | 0.746 | 3.41E-135 | 8 | Rpl14 | B_Cells |
| 0.679829 | 0.994 | 0.91 | 1.33E-134 | 8 | Rps27a | B_Cells |
| 0.254471 | 0.168 | 0.018 | 2.41E-134 | 8 | Cd69 | B_Cells |
| 0.410069 | 0.349 | 0.076 | 1.76E-132 | 8 | Ets1 | B_Cells |
| 0.254266 | 0.212 | 0.029 | 2.13E-132 | 8 | Sp140 | B_Cells |
| 0.767116 | 0.976 | 0.64 | 2.58E-131 | 8 | H2-D1 | B_Cells |
| 0.575307 | 0.516 | 0.17 | 1.40E-128 | 8 | Foxp1 | B_Cells |
| 0.638273 | 0.999 | 0.912 | 2.69E-128 | 8 | Rps8 | B_Cells |
| 0.626826 | 0.622 | 0.251 | 5.30E-127 | 8 | Gdi2 | B_Cells |
| 0.76362 | 0.526 | 0.173 | 4.00E-126 | 8 | Plac8 | B_Cells |
| 0.385241 | 0.36 | 0.084 | 7.14E-125 | 8 | Fam107b | B_Cells |
| 0.511023 | 0.568 | 0.184 | 6.22E-124 | 8 | Ifi30 | B_Cells |
| 0.669025 | 0.997 | 0.925 | 1.26E-122 | 8 | mt-Co3 | B_Cells |
| 0.326704 | 0.22 | 0.034 | 3.61E-122 | 8 | Stap1 | B_Cells |
| 0.67153 | 0.974 | 0.834 | 4.27E-122 | 8 | Rps26 | B_Cells |
| 0.396857 | 0.361 | 0.087 | 1.23E-121 | 8 | Arl6ip1 | B_Cells |
| 0.631592 | 0.997 | 0.925 | 3.79E-121 | 8 | Rpl37a | B_Cells |
| 0.362738 | 0.333 | 0.075 | 1.14E-120 | 8 | Got1 | B_Cells |
| 0.308373 | 0.266 | 0.05 | 2.81E-120 | 8 | Il2rg | B_Cells |
| 0.379019 | 0.354 | 0.084 | 2.93E-120 | 8 | Syngr2 | B_Cells |
| 0.289117 | 0.245 | 0.043 | 4.16E-119 | 8 | Cyth1 | B_Cells |
| 0.522885 | 0.514 | 0.166 | 2.89E-118 | 8 | Stk17b | B_Cells |
| 0.668071 | 0.932 | 0.748 | 1.10E-117 | 8 | Rps25 | B_Cells |
| 0.428086 | 0.413 | 0.11 | 7.59E-117 | 8 | Dusp2 | B_Cells |
| 0.609577 | 0.999 | 0.925 | 1.33E-116 | 8 | mt-Co2 | B_Cells |
| 0.312879 | 0.263 | 0.05 | 6.98E-116 | 8 | Limd2 | B_Cells |
| 0.659799 | 0.974 | 0.852 | 9.91E-116 | 8 | Rpl36 | B_Cells |
| 0.691024 | 0.923 | 0.731 | 1.32E-115 | 8 | Rps15 | B_Cells |
| 0.252546 | 0.205 | 0.031 | 1.15E-114 | 8 | Nop56 | B_Cells |
| 0.348569 | 0.313 | 0.07 | 6.01E-114 | 8 | Prpf4b | B_Cells |
| 0.689279 | 0.844 | 0.59 | 9.67E-114 | 8 | Rpl4 | B_Cells |
| 0.701649 | 0.875 | 0.632 | 8.62E-113 | 8 | Rps18 | B_Cells |
| 0.692655 | 0.991 | 0.886 | 1.07E-112 | 8 | Rps12 | B_Cells |
| 0.313092 | 0.275 | 0.056 | 2.94E-112 | 8 | Ssh2 | B_Cells |
| 0.52969 | 0.548 | 0.205 | 5.62E-112 | 8 | Cnbp | B_Cells |
| 0.255132 | 0.208 | 0.033 | 1.70E-111 | 8 | Krit1 | B_Cells |
| 0.329155 | 0.279 | 0.058 | 4.75E-111 | 8 | Nrd1 | B_Cells |
| 0.473864 | 0.371 | 0.1 | 2.72E-110 | 8 | Ezr | B_Cells |
| 0.631009 | 0.997 | 0.972 | 1.81E-108 | 8 | Malat1 | B_Cells |
| 0.343438 | 0.339 | 0.083 | 9.37E-108 | 8 | Tspan13 | B_Cells |
| 0.322883 | 0.304 | 0.07 | 1.25E-107 | 8 | Nop53 | B_Cells |
| 0.263059 | 0.222 | 0.039 | 3.50E-107 | 8 | Pnn | B_Cells |
| 0.461732 | 0.471 | 0.151 | 6.83E-106 | 8 | Cxcr4 | B_Cells |
| 0.329537 | 0.337 | 0.084 | 2.97E-102 | 8 | Sorl1 | B_Cells |
| 0.610355 | 0.991 | 0.903 | 6.68E-101 | 8 | Rpl39 | B_Cells |
| 0.396093 | 0.327 | 0.083 | 6.69E-101 | 8 | Neurl3 | B_Cells |
| 0.6985 | 0.667 | 0.335 | 1.09E-100 | 8 | Ncl | B_Cells |
| 0.322688 | 0.293 | 0.068 | 2.38E-100 | 8 | Smchd1 | B_Cells |
| 0.277249 | 0.243 | 0.048 | 2.43E-100 | 8 | Tcp11l2 | B_Cells |
| 0.266394 | 0.233 | 0.045 | 1.73E-99 | 8 | Rbm5 | B_Cells |
| 0.526088 | 0.518 | 0.2 | 2.01E-99 | 8 | Serp1 | B_Cells |
| 0.277742 | 0.24 | 0.05 | 1.82E-95 | 8 | Srsf10 | B_Cells |
| 0.37516 | 0.42 | 0.131 | 2.50E-95 | 8 | Psmb8 | B_Cells |
| 0.290877 | 0.191 | 0.032 | 6.29E-95 | 8 | AC149090.1 | B_Cells |
| 0.497396 | 0.472 | 0.172 | 1.47E-94 | 8 | Tgfb1 | B_Cells |
| 0.576617 | 0.977 | 0.869 | 3.13E-92 | 8 | Rpl34 | B_Cells |
| 0.277705 | 0.24 | 0.051 | 8.28E-92 | 8 | Ddx21 | B_Cells |
| 0.329589 | 0.73 | 0.29 | 6.14E-90 | 8 | Cd52 | B_Cells |
| 0.569614 | 0.929 | 0.738 | 1.15E-89 | 8 | Rpl22 | B_Cells |
| 0.558369 | 0.984 | 0.89 | 6.27E-88 | 8 | Rpl35a | B_Cells |
| 0.278911 | 0.252 | 0.057 | 2.33E-87 | 8 | Snx5 | B_Cells |
| 0.462572 | 0.596 | 0.248 | 6.74E-85 | 8 | Coro1a | B_Cells |
| 0.524437 | 0.602 | 0.285 | 8.81E-85 | 8 | mt-Nd5 | B_Cells |
| 0.445592 | 0.523 | 0.218 | 1.31E-83 | 8 | Cox7a2l | B_Cells |
| 0.298742 | 0.276 | 0.071 | 6.06E-82 | 8 | Ddx24 | B_Cells |
| 0.386416 | 0.403 | 0.138 | 7.20E-82 | 8 | Kmt2e | B_Cells |
| 0.594684 | 0.89 | 0.685 | 2.00E-81 | 8 | Rpl36a | B_Cells |
| 0.561259 | 0.967 | 0.82 | 3.13E-80 | 8 | Rpl15 | B_Cells |
| 0.660888 | 0.787 | 0.536 | 6.73E-80 | 8 | Rpl29 | B_Cells |
| 0.590723 | 0.886 | 0.683 | 1.58E-79 | 8 | Eef1b2 | B_Cells |
| 0.304887 | 0.303 | 0.085 | 5.82E-79 | 8 | Syf2 | B_Cells |
| 0.251684 | 0.223 | 0.05 | 3.57E-78 | 8 | Rrp1 | B_Cells |
| 0.570917 | 0.986 | 0.865 | 1.06E-77 | 8 | Rplp0 | B_Cells |
| 0.291547 | 0.276 | 0.074 | 8.07E-77 | 8 | Psma1 | B_Cells |
| 0.523251 | 0.987 | 0.906 | 3.31E-76 | 8 | Rpl37 | B_Cells |
| 0.586676 | 0.973 | 0.838 | 9.59E-76 | 8 | Ptma | B_Cells |
| 0.497016 | 1 | 0.897 | 9.42E-74 | 8 | Tmsb4x | B_Cells |
| 0.287475 | 0.272 | 0.076 | 1.46E-71 | 8 | Llph | B_Cells |
| 0.337012 | 0.415 | 0.15 | 3.50E-70 | 8 | Rac2 | B_Cells |
| 0.251024 | 0.24 | 0.062 | 4.11E-69 | 8 | Cisd2 | B_Cells |
| 0.430241 | 0.528 | 0.246 | 2.98E-68 | 8 | Hnrnpf | B_Cells |
| 0.341862 | 0.364 | 0.127 | 1.39E-67 | 8 | Lyn | B_Cells |
| 0.376734 | 0.39 | 0.146 | 2.22E-66 | 8 | Tubb4b | B_Cells |
| 0.266833 | 0.266 | 0.076 | 5.23E-66 | 8 | Nol7 | B_Cells |
| 0.288707 | 0.32 | 0.104 | 8.63E-66 | 8 | Zfp706 | B_Cells |
| 0.492572 | 0.65 | 0.39 | 1.04E-63 | 8 | Eif3f | B_Cells |
| 0.402848 | 0.498 | 0.229 | 2.88E-63 | 8 | Hnrnpa1 | B_Cells |
| 0.535777 | 0.939 | 0.78 | 3.84E-63 | 8 | mt-Nd1 | B_Cells |
| 0.576544 | 0.933 | 0.781 | 4.55E-63 | 8 | Rps2 | B_Cells |
| 0.391144 | 0.556 | 0.261 | 1.63E-59 | 8 | Laptm5 | B_Cells |
| 0.315184 | 0.353 | 0.13 | 2.44E-59 | 8 | Rbm25 | B_Cells |
| 0.389908 | 0.508 | 0.237 | 1.16E-58 | 8 | Arhgdib | B_Cells |
| 0.376436 | 0.427 | 0.183 | 2.29E-58 | 8 | Paip2 | B_Cells |
| 0.275148 | 0.289 | 0.096 | 7.92E-57 | 8 | Smim14 | B_Cells |
| 0.460735 | 0.872 | 0.699 | 1.97E-55 | 8 | Naca | B_Cells |
| 0.336388 | 0.38 | 0.155 | 9.32E-55 | 8 | Srsf2 | B_Cells |
| 0.481908 | 0.846 | 0.68 | 1.06E-54 | 8 | Rpl23a | B_Cells |
| 0.447084 | 0.639 | 0.393 | 1.34E-52 | 8 | Snrpg | B_Cells |
| 0.399796 | 0.495 | 0.24 | 4.18E-52 | 8 | Ier2 | B_Cells |
| 0.417024 | 0.657 | 0.39 | 7.46E-52 | 8 | Gm10260 | B_Cells |
| 0.439613 | 0.982 | 0.858 | 1.23E-50 | 8 | Rpl28 | B_Cells |
| 0.297089 | 0.343 | 0.136 | 3.26E-49 | 8 | Tra2a | B_Cells |
| 0.325563 | 0.405 | 0.181 | 1.07E-48 | 8 | Srrm2 | B_Cells |
| 0.292404 | 0.326 | 0.125 | 2.27E-48 | 8 | Tsc22d3 | B_Cells |
| 0.325318 | 0.393 | 0.172 | 4.93E-48 | 8 | Clk1 | B_Cells |
| 0.307484 | 0.344 | 0.14 | 9.62E-48 | 8 | Psme1 | B_Cells |
| 0.294074 | 0.351 | 0.145 | 4.72E-47 | 8 | Tra2b | B_Cells |
| 0.264389 | 0.266 | 0.093 | 7.84E-47 | 8 | Nop10 | B_Cells |
| 0.30086 | 0.353 | 0.146 | 2.48E-46 | 8 | Srsf5 | B_Cells |
| 0.252369 | 0.265 | 0.094 | 7.92E-46 | 8 | Eif3m | B_Cells |
| 0.402883 | 0.879 | 0.748 | 3.61E-44 | 8 | Rpl7a | B_Cells |
| 0.357511 | 0.488 | 0.259 | 3.22E-42 | 8 | Nsa2 | B_Cells |
| 0.363622 | 0.532 | 0.309 | 4.32E-41 | 8 | Eif3h | B_Cells |
| 0.274923 | 0.32 | 0.135 | 3.85E-40 | 8 | Eif5b | B_Cells |
| 0.36962 | 0.711 | 0.463 | 5.76E-40 | 8 | Arpc3 | B_Cells |
| 0.278267 | 0.455 | 0.226 | 6.78E-39 | 8 | Myl12b | B_Cells |
| 0.367923 | 0.787 | 0.653 | 2.10E-38 | 8 | Btf3 | B_Cells |
| 0.301867 | 0.432 | 0.216 | 2.25E-38 | 8 | Ube2b | B_Cells |
| 0.369308 | 0.576 | 0.364 | 3.05E-38 | 8 | Eef1g | B_Cells |
| 0.304904 | 0.997 | 0.941 | 4.56E-37 | 8 | mt-Co1 | B_Cells |
| 0.362807 | 0.61 | 0.399 | 5.50E-37 | 8 | Rbm39 | B_Cells |
| 0.381044 | 0.947 | 0.846 | 1.39E-36 | 8 | Rpl24 | B_Cells |
| 0.371147 | 0.945 | 0.799 | 1.42E-36 | 8 | mt-Nd4 | B_Cells |
| 0.36286 | 0.679 | 0.513 | 3.48E-35 | 8 | Pfdn5 | B_Cells |
| 0.255231 | 0.39 | 0.194 | 9.98E-34 | 8 | Eif3e | B_Cells |
| 0.295639 | 0.428 | 0.231 | 3.04E-32 | 8 | Srsf3 | B_Cells |
| 0.293076 | 0.825 | 0.591 | 4.94E-32 | 8 | Btg1 | B_Cells |
| 0.260748 | 0.336 | 0.163 | 3.05E-30 | 8 | Mpc1 | B_Cells |
| 0.337329 | 0.768 | 0.643 | 2.65E-29 | 8 | Ddx5 | B_Cells |
| 0.2501 | 0.349 | 0.174 | 1.28E-28 | 8 | Mbnl1 | B_Cells |
| 0.29329 | 0.464 | 0.277 | 1.13E-26 | 8 | 2410006H16Rik | B_Cells |
| 0.268879 | 0.387 | 0.215 | 3.63E-26 | 8 | Hnrnpa0 | B_Cells |
| 0.298744 | 0.462 | 0.281 | 6.92E-26 | 8 | Eif5 | B_Cells |
| 0.312487 | 0.552 | 0.379 | 8.22E-25 | 8 | Serbp1 | B_Cells |
| 0.293971 | 0.42 | 0.247 | 1.91E-24 | 8 | Atf4 | B_Cells |
| 0.264699 | 0.494 | 0.318 | 7.90E-22 | 8 | Son | B_Cells |
| 0.301925 | 0.653 | 0.5 | 8.68E-22 | 8 | mt-Nd3 | B_Cells |
| 0.282886 | 0.615 | 0.462 | 1.16E-20 | 8 | Pabpc1 | B_Cells |
| 0.386024 | 0.496 | 0.349 | 1.58E-20 | 8 | Xist | B_Cells |
| 0.253222 | 0.477 | 0.328 | 5.13E-16 | 8 | Slc25a5 | B_Cells |
| 0.266205 | 0.694 | 0.566 | 6.59E-14 | 8 | Rpl22l1 | B_Cells |
| 0.257298 | 0.465 | 0.334 | 1.11E-13 | 8 | Eif2s2 | B_Cells |
| 0.260121 | 0.679 | 0.59 | 9.22E-09 | 8 | Npm1 | B_Cells |
| 2.778452 | 1 | 0.147 | 0 | 9 | Cd74 | Macrophages (Mac 1.2) |
| 2.513941 | 0.99 | 0.111 | 0 | 9 | H2-Aa | Macrophages (Mac 1.2) |
| 2.434311 | 0.981 | 0.11 | 0 | 9 | H2-Ab1 | Macrophages (Mac 1.2) |
| 2.069009 | 0.866 | 0.225 | 0 | 9 | Apoe | Macrophages (Mac 1.2) |
| 1.816797 | 0.966 | 0.209 | 0 | 9 | Lyz2 | Macrophages (Mac 1.2) |
| 1.682232 | 0.575 | 0.065 | 0 | 9 | C1qb | Macrophages (Mac 1.2) |
| 1.601221 | 0.525 | 0.06 | 0 | 9 | C1qa | Macrophages (Mac 1.2) |
| 1.42285 | 0.941 | 0.193 | 0 | 9 | Ctss | Macrophages (Mac 1.2) |
| 1.255731 | 0.459 | 0.049 | 0 | 9 | H2-Eb1 | Macrophages (Mac 1.2) |
| 1.07768 | 0.488 | 0.053 | 0 | 9 | C1qc | Macrophages (Mac 1.2) |
| 1.03402 | 0.606 | 0.088 | 0 | 9 | Ms4a7 | Macrophages (Mac 1.2) |
| 1.028037 | 0.753 | 0.11 | 0 | 9 | Ms4a6c | Macrophages (Mac 1.2) |
| 0.935355 | 0.762 | 0.176 | 0 | 9 | Ifi30 | Macrophages (Mac 1.2) |
| 0.926892 | 0.712 | 0.149 | 0 | 9 | Lgmn | Macrophages (Mac 1.2) |
| 0.853238 | 0.668 | 0.07 | 0 | 9 | Aif1 | Macrophages (Mac 1.2) |
| 0.846238 | 0.6 | 0.105 | 0 | 9 | Cd83 | Macrophages (Mac 1.2) |
| 0.840808 | 0.646 | 0.094 | 0 | 9 | Cxcl16 | Macrophages (Mac 1.2) |
| 0.739583 | 0.649 | 0.097 | 0 | 9 | Csf1r | Macrophages (Mac 1.2) |
| 0.727712 | 0.597 | 0.089 | 0 | 9 | Ms4a6d | Macrophages (Mac 1.2) |
| 0.712154 | 0.553 | 0.085 | 0 | 9 | Cybb | Macrophages (Mac 1.2) |
| 0.509502 | 0.39 | 0.035 | 0 | 9 | H2-DMb1 | Macrophages (Mac 1.2) |
| 0.615269 | 0.5 | 0.078 | 1.41E-288 | 9 | Fcgr2b | Macrophages (Mac 1.2) |
| 0.536934 | 0.397 | 0.048 | 1.16E-285 | 9 | Ccr2 | Macrophages (Mac 1.2) |
| 1.14547 | 0.957 | 0.291 | 5.92E-281 | 9 | Fcer1g | Macrophages (Mac 1.2) |
| 0.498459 | 0.415 | 0.055 | 2.99E-275 | 9 | H2-DMa | Macrophages (Mac 1.2) |
| 0.687589 | 0.621 | 0.126 | 4.07E-266 | 9 | Mpeg1 | Macrophages (Mac 1.2) |
| 0.812132 | 0.841 | 0.249 | 4.15E-261 | 9 | Laptm5 | Macrophages (Mac 1.2) |
| 1.215382 | 0.834 | 0.303 | 4.07E-250 | 9 | Tgfbi | Macrophages (Mac 1.2) |
| 0.998949 | 0.919 | 0.267 | 2.44E-245 | 9 | Cd14 | Macrophages (Mac 1.2) |
| 0.810482 | 0.822 | 0.261 | 4.12E-243 | 9 | Ucp2 | Macrophages (Mac 1.2) |
| 0.857791 | 0.813 | 0.235 | 7.59E-241 | 9 | Slfn2 | Macrophages (Mac 1.2) |
| 0.769495 | 0.54 | 0.11 | 2.91E-238 | 9 | Mafb | Macrophages (Mac 1.2) |
| 0.921466 | 0.966 | 0.303 | 4.14E-237 | 9 | Tyrobp | Macrophages (Mac 1.2) |
| 0.993596 | 0.919 | 0.382 | 1.25E-234 | 9 | Cyba | Macrophages (Mac 1.2) |
| 0.501078 | 0.472 | 0.084 | 6.32E-231 | 9 | Unc93b1 | Macrophages (Mac 1.2) |
| 0.646096 | 0.696 | 0.182 | 3.87E-225 | 9 | Efhd2 | Macrophages (Mac 1.2) |
| 0.475202 | 0.3 | 0.036 | 2.30E-216 | 9 | Ms4a4c | Macrophages (Mac 1.2) |
| 0.871143 | 0.775 | 0.264 | 5.43E-213 | 9 | Ctsc | Macrophages (Mac 1.2) |
| 0.430195 | 0.359 | 0.053 | 2.04E-212 | 9 | Ms4a6b | Macrophages (Mac 1.2) |
| 0.795662 | 0.46 | 0.087 | 4.36E-212 | 9 | Rgs1 | Macrophages (Mac 1.2) |
| 1.233522 | 0.803 | 0.232 | 1.06E-211 | 9 | Il1b | Macrophages (Mac 1.2) |
| 0.364904 | 0.278 | 0.031 | 1.70E-211 | 9 | Clec4a3 | Macrophages (Mac 1.2) |
| 0.615325 | 0.582 | 0.136 | 1.12E-208 | 9 | Tlr2 | Macrophages (Mac 1.2) |
| 0.577368 | 0.585 | 0.142 | 1.16E-206 | 9 | Arl4c | Macrophages (Mac 1.2) |
| 1.16132 | 0.691 | 0.211 | 3.23E-201 | 9 | Ifi27l2a | Macrophages (Mac 1.2) |
| 0.660225 | 0.654 | 0.171 | 1.09E-199 | 9 | Lst1 | Macrophages (Mac 1.2) |
| 0.894029 | 0.925 | 0.452 | 6.17E-199 | 9 | Psap | Macrophages (Mac 1.2) |
| 0.613728 | 0.484 | 0.1 | 7.44E-196 | 9 | Ccl9 | Macrophages (Mac 1.2) |
| 0.906404 | 0.844 | 0.285 | 8.46E-195 | 9 | Cd52 | Macrophages (Mac 1.2) |
| 0.570111 | 0.524 | 0.115 | 4.11E-193 | 9 | Clec4n | Macrophages (Mac 1.2) |
| 0.477965 | 0.456 | 0.095 | 4.31E-186 | 9 | Rgs10 | Macrophages (Mac 1.2) |
| 0.982231 | 0.634 | 0.179 | 1.18E-181 | 9 | Il1rn | Macrophages (Mac 1.2) |
| 0.429655 | 0.406 | 0.077 | 1.32E-181 | 9 | Cd300c2 | Macrophages (Mac 1.2) |
| 1.097172 | 0.999 | 0.909 | 6.26E-181 | 9 | Ftl1 | Macrophages (Mac 1.2) |
| 1.00638 | 0.876 | 0.373 | 9.82E-179 | 9 | Lgals3 | Macrophages (Mac 1.2) |
| 0.75927 | 0.934 | 0.479 | 9.27E-178 | 9 | Atp6v0c | Macrophages (Mac 1.2) |
| 0.298665 | 0.221 | 0.023 | 1.79E-174 | 9 | Fcgr1 | Macrophages (Mac 1.2) |
| 0.490136 | 0.451 | 0.099 | 8.91E-171 | 9 | Dab2 | Macrophages (Mac 1.2) |
| 0.831915 | 0.621 | 0.187 | 6.01E-170 | 9 | Id2 | Macrophages (Mac 1.2) |
| 0.871685 | 0.851 | 0.414 | 2.06E-169 | 9 | Atox1 | Macrophages (Mac 1.2) |
| 0.77597 | 0.756 | 0.281 | 1.74E-166 | 9 | Cdkn1a | Macrophages (Mac 1.2) |
| 0.499438 | 0.51 | 0.125 | 4.56E-165 | 9 | Cd68 | Macrophages (Mac 1.2) |
| 0.473862 | 0.522 | 0.132 | 1.08E-163 | 9 | Gm2a | Macrophages (Mac 1.2) |
| 0.973023 | 1 | 0.897 | 2.46E-161 | 9 | Tmsb4x | Macrophages (Mac 1.2) |
| 0.682412 | 0.785 | 0.318 | 1.38E-160 | 9 | Ctsz | Macrophages (Mac 1.2) |
| 0.374147 | 0.35 | 0.065 | 6.38E-158 | 9 | Pid1 | Macrophages (Mac 1.2) |
| 0.840657 | 0.966 | 0.649 | 1.22E-156 | 9 | B2m | Macrophages (Mac 1.2) |
| 0.273712 | 0.225 | 0.027 | 1.56E-156 | 9 | Pld4 | Macrophages (Mac 1.2) |
| 0.421713 | 0.369 | 0.072 | 1.07E-155 | 9 | Trem2 | Macrophages (Mac 1.2) |
| 0.818594 | 0.819 | 0.352 | 6.15E-154 | 9 | Cstb | Macrophages (Mac 1.2) |
| 0.486297 | 0.346 | 0.066 | 1.28E-152 | 9 | Osm | Macrophages (Mac 1.2) |
| 0.479624 | 0.772 | 0.26 | 2.72E-149 | 9 | Lcp1 | Macrophages (Mac 1.2) |
| 0.585301 | 0.678 | 0.236 | 3.44E-148 | 9 | Atp2b1 | Macrophages (Mac 1.2) |
| 0.285288 | 0.219 | 0.028 | 2.17E-147 | 9 | Ccr5 | Macrophages (Mac 1.2) |
| 0.585055 | 0.422 | 0.102 | 2.79E-144 | 9 | Pltp | Macrophages (Mac 1.2) |
| 0.43637 | 0.434 | 0.105 | 4.43E-143 | 9 | Ptpn18 | Macrophages (Mac 1.2) |
| 0.697746 | 0.856 | 0.462 | 7.76E-141 | 9 | Npc2 | Macrophages (Mac 1.2) |
| 0.897104 | 0.999 | 0.948 | 1.38E-139 | 9 | Fth1 | Macrophages (Mac 1.2) |
| 0.529254 | 0.666 | 0.216 | 4.36E-139 | 9 | Alox5ap | Macrophages (Mac 1.2) |
| 0.421817 | 0.425 | 0.104 | 1.63E-135 | 9 | Sirpa | Macrophages (Mac 1.2) |
| 0.521916 | 0.699 | 0.244 | 1.34E-133 | 9 | Coro1a | Macrophages (Mac 1.2) |
| 0.325355 | 0.272 | 0.047 | 4.09E-133 | 9 | Runx3 | Macrophages (Mac 1.2) |
| 0.824873 | 0.976 | 0.641 | 4.43E-133 | 9 | H2-D1 | Macrophages (Mac 1.2) |
| 0.452538 | 0.587 | 0.184 | 7.19E-132 | 9 | Spi1 | Macrophages (Mac 1.2) |
| 0.386641 | 0.406 | 0.1 | 6.79E-129 | 9 | Ctsh | Macrophages (Mac 1.2) |
| 0.356487 | 0.338 | 0.072 | 8.94E-129 | 9 | Cfp | Macrophages (Mac 1.2) |
| 0.360272 | 0.338 | 0.072 | 2.45E-128 | 9 | Clec4a2 | Macrophages (Mac 1.2) |
| 0.425829 | 0.449 | 0.121 | 1.44E-127 | 9 | Fam49b | Macrophages (Mac 1.2) |
| 0.624081 | 0.84 | 0.377 | 1.82E-127 | 9 | Fxyd5 | Macrophages (Mac 1.2) |
| 0.541635 | 0.288 | 0.055 | 2.89E-127 | 9 | Tnfsf9 | Macrophages (Mac 1.2) |
| 0.491553 | 0.543 | 0.176 | 2.05E-125 | 9 | Pitpna | Macrophages (Mac 1.2) |
| 0.617526 | 0.834 | 0.371 | 1.47E-124 | 9 | Pim1 | Macrophages (Mac 1.2) |
| 0.679082 | 0.916 | 0.512 | 2.40E-124 | 9 | H2-K1 | Macrophages (Mac 1.2) |
| 0.294321 | 0.184 | 0.023 | 3.61E-123 | 9 | Tnip3 | Macrophages (Mac 1.2) |
| 0.404999 | 0.66 | 0.22 | 4.20E-121 | 9 | Plek | Macrophages (Mac 1.2) |
| 0.64769 | 0.697 | 0.293 | 6.11E-121 | 9 | Ly6e | Macrophages (Mac 1.2) |
| 0.444793 | 0.626 | 0.2 | 1.51E-120 | 9 | Clec4e | Macrophages (Mac 1.2) |
| 0.305427 | 0.235 | 0.039 | 9.65E-120 | 9 | Ifi207 | Macrophages (Mac 1.2) |
| 0.354511 | 0.306 | 0.063 | 3.10E-118 | 9 | Msr1 | Macrophages (Mac 1.2) |
| 0.453098 | 0.374 | 0.095 | 1.03E-112 | 9 | Malt1 | Macrophages (Mac 1.2) |
| 0.543724 | 0.625 | 0.236 | 1.11E-112 | 9 | Grn | Macrophages (Mac 1.2) |
| 0.680346 | 0.206 | 0.032 | 1.74E-110 | 9 | Il10 | Macrophages (Mac 1.2) |
| 0.40695 | 0.51 | 0.162 | 2.06E-110 | 9 | Ncf4 | Macrophages (Mac 1.2) |
| 0.421883 | 0.518 | 0.162 | 8.43E-110 | 9 | Lilrb4a | Macrophages (Mac 1.2) |
| 0.439405 | 0.39 | 0.102 | 1.58E-109 | 9 | Tnf | Macrophages (Mac 1.2) |
| 0.305786 | 0.272 | 0.054 | 3.33E-109 | 9 | C3ar1 | Macrophages (Mac 1.2) |
| 0.478822 | 0.55 | 0.19 | 6.10E-108 | 9 | Ninj1 | Macrophages (Mac 1.2) |
| 0.380693 | 0.415 | 0.117 | 2.76E-107 | 9 | Fyb | Macrophages (Mac 1.2) |
| 0.676089 | 0.907 | 0.639 | 5.25E-107 | 9 | Cst3 | Macrophages (Mac 1.2) |
| 0.410002 | 0.465 | 0.141 | 6.39E-107 | 9 | Rel | Macrophages (Mac 1.2) |
| 0.355003 | 0.515 | 0.159 | 1.30E-106 | 9 | Ptafr | Macrophages (Mac 1.2) |
| 0.92201 | 0.793 | 0.394 | 4.11E-106 | 9 | Cxcl2 | Macrophages (Mac 1.2) |
| 0.309549 | 0.276 | 0.058 | 9.63E-106 | 9 | Jarid2 | Macrophages (Mac 1.2) |
| 0.379003 | 0.594 | 0.208 | 8.54E-104 | 9 | Ptprc | Macrophages (Mac 1.2) |
| 0.42418 | 0.478 | 0.151 | 1.01E-103 | 9 | Cxcr4 | Macrophages (Mac 1.2) |
| 0.912425 | 0.315 | 0.073 | 2.18E-103 | 9 | Ccl4 | Macrophages (Mac 1.2) |
| 0.729297 | 0.834 | 0.455 | 9.93E-103 | 9 | Zfp36 | Macrophages (Mac 1.2) |
| 0.524468 | 0.863 | 0.456 | 1.38E-102 | 9 | Mcl1 | Macrophages (Mac 1.2) |
| 0.290895 | 0.266 | 0.055 | 2.38E-102 | 9 | Plbd1 | Macrophages (Mac 1.2) |
| 0.394844 | 0.404 | 0.114 | 2.59E-102 | 9 | Traf1 | Macrophages (Mac 1.2) |
| 0.557882 | 0.399 | 0.116 | 1.10E-101 | 9 | Selenop | Macrophages (Mac 1.2) |
| 0.463061 | 0.624 | 0.244 | 5.36E-101 | 9 | Sdcbp | Macrophages (Mac 1.2) |
| 0.503073 | 0.612 | 0.246 | 4.95E-100 | 9 | Zeb2 | Macrophages (Mac 1.2) |
| 0.418414 | 0.612 | 0.233 | 6.76E-100 | 9 | Arhgdib | Macrophages (Mac 1.2) |
| 0.33352 | 0.268 | 0.057 | 2.36E-99 | 9 | Trf | Macrophages (Mac 1.2) |
| 0.28957 | 0.21 | 0.036 | 2.97E-99 | 9 | Mrc1 | Macrophages (Mac 1.2) |
| 0.468284 | 0.997 | 0.973 | 8.74E-99 | 9 | Rps29 | Macrophages (Mac 1.2) |
| 0.624143 | 0.912 | 0.566 | 7.19E-96 | 9 | Cebpb | Macrophages (Mac 1.2) |
| 0.304904 | 0.332 | 0.085 | 1.13E-94 | 9 | Syngr2 | Macrophages (Mac 1.2) |
| 0.378191 | 0.437 | 0.139 | 7.46E-94 | 9 | Ifngr1 | Macrophages (Mac 1.2) |
| 0.452421 | 0.526 | 0.192 | 9.06E-94 | 9 | Plaur | Macrophages (Mac 1.2) |
| 0.55866 | 0.801 | 0.44 | 6.75E-92 | 9 | Gpx1 | Macrophages (Mac 1.2) |
| 0.332412 | 0.365 | 0.104 | 8.83E-90 | 9 | Itgam | Macrophages (Mac 1.2) |
| 0.305004 | 0.304 | 0.077 | 1.21E-89 | 9 | Got1 | Macrophages (Mac 1.2) |
| 0.47532 | 0.86 | 0.51 | 1.22E-89 | 9 | Arpc1b | Macrophages (Mac 1.2) |
| 0.253001 | 0.241 | 0.052 | 3.83E-87 | 9 | Cd86 | Macrophages (Mac 1.2) |
| 0.3578 | 0.375 | 0.112 | 6.74E-87 | 9 | Cflar | Macrophages (Mac 1.2) |
| 0.636458 | 0.834 | 0.52 | 3.49E-82 | 9 | Ctsb | Macrophages (Mac 1.2) |
| 0.359636 | 0.475 | 0.17 | 4.68E-82 | 9 | Card19 | Macrophages (Mac 1.2) |
| 0.532003 | 0.925 | 0.723 | 5.34E-82 | 9 | Calm1 | Macrophages (Mac 1.2) |
| 0.337974 | 0.485 | 0.178 | 1.00E-78 | 9 | Cotl1 | Macrophages (Mac 1.2) |
| 0.263421 | 0.631 | 0.258 | 1.01E-78 | 9 | Marcksl1 | Macrophages (Mac 1.2) |
| 0.342628 | 0.457 | 0.158 | 2.19E-78 | 9 | C5ar1 | Macrophages (Mac 1.2) |
| 0.493698 | 0.988 | 0.969 | 6.46E-78 | 9 | Fau | Macrophages (Mac 1.2) |
| 0.477441 | 0.844 | 0.535 | 7.58E-78 | 9 | Arpc2 | Macrophages (Mac 1.2) |
| 0.29675 | 0.332 | 0.098 | 5.94E-76 | 9 | Fcgr3 | Macrophages (Mac 1.2) |
| 0.34491 | 0.363 | 0.116 | 8.55E-76 | 9 | Dusp5 | Macrophages (Mac 1.2) |
| 0.370441 | 0.501 | 0.202 | 9.72E-75 | 9 | Capza2 | Macrophages (Mac 1.2) |
| 0.319226 | 0.363 | 0.117 | 3.59E-74 | 9 | Gna13 | Macrophages (Mac 1.2) |
| 0.369718 | 0.357 | 0.113 | 1.81E-73 | 9 | Dusp2 | Macrophages (Mac 1.2) |
| 0.273618 | 0.216 | 0.048 | 1.82E-73 | 9 | F13a1 | Macrophages (Mac 1.2) |
| 0.487821 | 0.593 | 0.273 | 4.47E-72 | 9 | Sat1 | Macrophages (Mac 1.2) |
| 0.255193 | 0.225 | 0.053 | 7.70E-72 | 9 | Tmem189 | Macrophages (Mac 1.2) |
| 0.917129 | 0.766 | 0.517 | 8.69E-72 | 9 | Ier3 | Macrophages (Mac 1.2) |
| 0.453211 | 0.962 | 0.787 | 4.79E-71 | 9 | Pfn1 | Macrophages (Mac 1.2) |
| 0.375368 | 0.6 | 0.275 | 1.16E-70 | 9 | Atp6v0b | Macrophages (Mac 1.2) |
| 0.27266 | 0.281 | 0.078 | 1.62E-69 | 9 | Gngt2 | Macrophages (Mac 1.2) |
| 0.350721 | 0.422 | 0.152 | 1.87E-69 | 9 | Cytip | Macrophages (Mac 1.2) |
| 0.289961 | 0.366 | 0.119 | 3.70E-69 | 9 | Slc15a3 | Macrophages (Mac 1.2) |
| 0.329866 | 0.34 | 0.109 | 4.80E-69 | 9 | Rilpl2 | Macrophages (Mac 1.2) |
| 0.306322 | 0.278 | 0.079 | 4.99E-69 | 9 | Hexb | Macrophages (Mac 1.2) |
| 0.278837 | 0.24 | 0.06 | 5.27E-68 | 9 | Gpr183 | Macrophages (Mac 1.2) |
| 0.368319 | 0.824 | 0.458 | 7.92E-68 | 9 | Arpc3 | Macrophages (Mac 1.2) |
| 0.306888 | 0.374 | 0.127 | 1.59E-67 | 9 | Lyn | Macrophages (Mac 1.2) |
| 0.297824 | 0.356 | 0.116 | 2.06E-67 | 9 | Csf2rb | Macrophages (Mac 1.2) |
| 0.355536 | 0.296 | 0.089 | 2.47E-67 | 9 | Vegfa | Macrophages (Mac 1.2) |
| 0.30448 | 0.322 | 0.1 | 9.81E-67 | 9 | Plin2 | Macrophages (Mac 1.2) |
| 0.29132 | 0.418 | 0.147 | 1.25E-66 | 9 | Ccr1 | Macrophages (Mac 1.2) |
| 0.452635 | 0.884 | 0.673 | 4.82E-65 | 9 | Nfkbia | Macrophages (Mac 1.2) |
| 0.379425 | 0.572 | 0.266 | 2.31E-63 | 9 | Picalm | Macrophages (Mac 1.2) |
| 0.348952 | 0.6 | 0.283 | 2.80E-63 | 9 | Arpc5 | Macrophages (Mac 1.2) |
| 0.501532 | 0.997 | 0.958 | 9.90E-62 | 9 | Actb | Macrophages (Mac 1.2) |
| 0.296224 | 0.294 | 0.091 | 1.50E-61 | 9 | Abca1 | Macrophages (Mac 1.2) |
| 0.302908 | 0.262 | 0.073 | 1.76E-61 | 9 | Pf4 | Macrophages (Mac 1.2) |
| 0.280601 | 0.509 | 0.21 | 8.27E-60 | 9 | Cd53 | Macrophages (Mac 1.2) |
| 0.312569 | 0.384 | 0.147 | 1.27E-57 | 9 | Ctsa | Macrophages (Mac 1.2) |
| 0.322074 | 0.428 | 0.175 | 2.60E-57 | 9 | Tgfb1 | Macrophages (Mac 1.2) |
| 0.460133 | 0.499 | 0.235 | 6.41E-56 | 9 | Basp1 | Macrophages (Mac 1.2) |
| 0.404192 | 0.759 | 0.465 | 6.71E-55 | 9 | Sh3bgrl3 | Macrophages (Mac 1.2) |
| 0.405341 | 0.672 | 0.363 | 1.93E-54 | 9 | Cd44 | Macrophages (Mac 1.2) |
| 0.323931 | 0.347 | 0.129 | 1.38E-52 | 9 | Tnfrsf1b | Macrophages (Mac 1.2) |
| 1.126775 | 0.503 | 0.261 | 2.62E-52 | 9 | Thbs1 | Macrophages (Mac 1.2) |
| 0.286455 | 0.363 | 0.14 | 3.44E-52 | 9 | Tgif1 | Macrophages (Mac 1.2) |
| 0.338016 | 0.666 | 0.37 | 8.82E-52 | 9 | Lamp1 | Macrophages (Mac 1.2) |
| 0.279966 | 0.406 | 0.168 | 3.20E-51 | 9 | Vamp8 | Macrophages (Mac 1.2) |
| 0.811468 | 0.569 | 0.313 | 6.09E-51 | 9 | Ptgs2 | Macrophages (Mac 1.2) |
| 0.341051 | 0.8 | 0.523 | 7.48E-51 | 9 | Cdc42 | Macrophages (Mac 1.2) |
| 0.407188 | 0.672 | 0.396 | 8.53E-51 | 9 | Tspo | Macrophages (Mac 1.2) |
| 0.274395 | 0.366 | 0.14 | 1.21E-50 | 9 | Lilr4b | Macrophages (Mac 1.2) |
| 0.456904 | 0.866 | 0.59 | 8.34E-50 | 9 | Btg1 | Macrophages (Mac 1.2) |
| 0.362402 | 0.603 | 0.315 | 9.68E-50 | 9 | Kdm6b | Macrophages (Mac 1.2) |
| 0.400942 | 0.606 | 0.329 | 9.83E-50 | 9 | Socs3 | Macrophages (Mac 1.2) |
| 0.387327 | 0.493 | 0.238 | 2.35E-49 | 9 | Sdc4 | Macrophages (Mac 1.2) |
| 0.305254 | 0.566 | 0.28 | 2.82E-49 | 9 | Nfe2l2 | Macrophages (Mac 1.2) |
| 0.363297 | 0.529 | 0.258 | 2.37E-48 | 9 | Tnfaip3 | Macrophages (Mac 1.2) |
| 0.445138 | 0.915 | 0.705 | 8.27E-48 | 9 | Junb | Macrophages (Mac 1.2) |
| 0.285844 | 0.468 | 0.211 | 8.60E-48 | 9 | Ehd1 | Macrophages (Mac 1.2) |
| 0.2809 | 0.4 | 0.171 | 6.06E-47 | 9 | Actr2 | Macrophages (Mac 1.2) |
| 0.250944 | 0.346 | 0.135 | 1.13E-46 | 9 | Psmb8 | Macrophages (Mac 1.2) |
| 0.263467 | 0.356 | 0.138 | 1.24E-46 | 9 | Pla2g7 | Macrophages (Mac 1.2) |
| 0.469033 | 0.765 | 0.511 | 1.50E-46 | 9 | H2afz | Macrophages (Mac 1.2) |
| 0.487753 | 0.597 | 0.343 | 5.69E-46 | 9 | Ifrd1 | Macrophages (Mac 1.2) |
| 0.323436 | 0.496 | 0.247 | 3.11E-45 | 9 | Aprt | Macrophages (Mac 1.2) |
| 0.317053 | 0.944 | 0.86 | 9.74E-45 | 9 | H3f3a | Macrophages (Mac 1.2) |
| 0.37468 | 0.856 | 0.527 | 1.15E-44 | 9 | S100a4 | Macrophages (Mac 1.2) |
| 0.275465 | 0.362 | 0.151 | 2.16E-44 | 9 | Ywhah | Macrophages (Mac 1.2) |
| 0.330943 | 0.565 | 0.31 | 1.93E-43 | 9 | Cd47 | Macrophages (Mac 1.2) |
| 0.294417 | 0.415 | 0.192 | 6.52E-42 | 9 | Slc3a2 | Macrophages (Mac 1.2) |
| 0.301061 | 0.629 | 0.356 | 1.15E-40 | 9 | Iqgap1 | Macrophages (Mac 1.2) |
| 0.33252 | 0.715 | 0.463 | 5.11E-40 | 9 | Clic1 | Macrophages (Mac 1.2) |
| 0.277039 | 0.507 | 0.26 | 6.13E-40 | 9 | Actr3 | Macrophages (Mac 1.2) |
| 0.256743 | 0.385 | 0.166 | 7.88E-40 | 9 | Kctd12 | Macrophages (Mac 1.2) |
| 0.428133 | 0.449 | 0.232 | 9.42E-40 | 9 | Zfp36l2 | Macrophages (Mac 1.2) |
| 0.308499 | 0.793 | 0.571 | 2.97E-39 | 9 | Gnai2 | Macrophages (Mac 1.2) |
| 0.269168 | 0.537 | 0.281 | 8.01E-39 | 9 | Rap1b | Macrophages (Mac 1.2) |
| 0.255272 | 0.106 | 0.022 | 2.95E-38 | 9 | Ly6c2 | Macrophages (Mac 1.2) |
| 0.287752 | 0.501 | 0.265 | 7.92E-38 | 9 | Bri3 | Macrophages (Mac 1.2) |
| 0.427495 | 0.688 | 0.45 | 6.62E-36 | 9 | Dusp1 | Macrophages (Mac 1.2) |
| 0.285182 | 0.866 | 0.685 | 2.28E-34 | 9 | Cfl1 | Macrophages (Mac 1.2) |
| 0.313263 | 0.282 | 0.115 | 7.85E-33 | 9 | Ccl6 | Macrophages (Mac 1.2) |
| 0.295607 | 0.651 | 0.43 | 2.65E-31 | 9 | Clta | Macrophages (Mac 1.2) |
| 0.319268 | 0.728 | 0.529 | 1.50E-30 | 9 | Emp3 | Macrophages (Mac 1.2) |
| 0.252302 | 0.44 | 0.229 | 1.62E-30 | 9 | Capg | Macrophages (Mac 1.2) |
| 0.25055 | 0.562 | 0.325 | 3.21E-30 | 9 | Msn | Macrophages (Mac 1.2) |
| 0.40451 | 0.95 | 0.894 | 7.55E-30 | 9 | H3f3b | Macrophages (Mac 1.2) |
| 0.260975 | 0.465 | 0.259 | 2.14E-29 | 9 | Gdi2 | Macrophages (Mac 1.2) |
| 0.293655 | 0.972 | 0.95 | 5.80E-28 | 9 | Rps9 | Macrophages (Mac 1.2) |
| 0.357887 | 0.688 | 0.479 | 8.63E-28 | 9 | Ahnak | Macrophages (Mac 1.2) |
| 0.523435 | 0.353 | 0.182 | 1.42E-27 | 9 | Plac8 | Macrophages (Mac 1.2) |
| 0.296184 | 0.753 | 0.579 | 2.68E-27 | 9 | Sem1 | Macrophages (Mac 1.2) |
| 0.272464 | 0.868 | 0.751 | 5.13E-27 | 9 | Rps25 | Macrophages (Mac 1.2) |
| 0.254818 | 0.54 | 0.333 | 2.20E-26 | 9 | Cox5a | Macrophages (Mac 1.2) |
| 0.27623 | 0.254 | 0.111 | 2.97E-26 | 9 | Hilpda | Macrophages (Mac 1.2) |
| 0.285576 | 0.954 | 0.891 | 1.55E-24 | 9 | Rpl35a | Macrophages (Mac 1.2) |
| 0.276567 | 0.196 | 0.078 | 2.35E-24 | 9 | Mmp19 | Macrophages (Mac 1.2) |
| 0.254726 | 0.604 | 0.411 | 2.63E-23 | 9 | Ndufb1-ps | Macrophages (Mac 1.2) |
| 0.270312 | 0.703 | 0.509 | 2.91E-22 | 9 | Eif4a1 | Macrophages (Mac 1.2) |
| 0.291715 | 0.429 | 0.262 | 2.75E-21 | 9 | Hif1a | Macrophages (Mac 1.2) |
| 0.356209 | 0.866 | 0.773 | 2.44E-19 | 9 | Hspa8 | Macrophages (Mac 1.2) |
| 0.296071 | 0.454 | 0.294 | 2.71E-18 | 9 | Sqstm1 | Macrophages (Mac 1.2) |
| 0.311391 | 0.778 | 0.635 | 3.31E-18 | 9 | S100a10 | Macrophages (Mac 1.2) |
| 0.307681 | 0.69 | 0.469 | 1.74E-17 | 9 | Fos | Macrophages (Mac 1.2) |
| 0.26961 | 0.387 | 0.238 | 3.29E-16 | 9 | Sod2 | Macrophages (Mac 1.2) |
| 0.261472 | 0.529 | 0.375 | 3.37E-13 | 9 | Klf6 | Macrophages (Mac 1.2) |
| 0.317003 | 0.829 | 0.761 | 8.46E-10 | 9 | Itm2b | Macrophages (Mac 1.2) |
| 2.537299 | 0.868 | 0.244 | 0 | 10 | Thbs1 | Macrophages (Mac 1.3) |
| 1.958728 | 0.936 | 0.227 | 0 | 10 | Il1b | Macrophages (Mac 1.3) |
| 1.913368 | 0.901 | 0.212 | 0 | 10 | Lyz2 | Macrophages (Mac 1.3) |
| 1.539762 | 0.952 | 0.266 | 0 | 10 | Cd14 | Macrophages (Mac 1.3) |
| 1.210241 | 0.897 | 0.232 | 0 | 10 | Slfn2 | Macrophages (Mac 1.3) |
| 1.124809 | 0.665 | 0.092 | 0 | 10 | Ccl9 | Macrophages (Mac 1.3) |
| 0.997354 | 0.883 | 0.189 | 0 | 10 | Clec4e | Macrophages (Mac 1.3) |
| 0.98194 | 0.844 | 0.198 | 0 | 10 | Ctss | Macrophages (Mac 1.3) |
| 0.91934 | 0.681 | 0.114 | 0 | 10 | Ms4a6c | Macrophages (Mac 1.3) |
| 0.893837 | 0.496 | 0.025 | 0 | 10 | F10 | Macrophages (Mac 1.3) |
| 0.785982 | 0.666 | 0.132 | 0 | 10 | Tlr2 | Macrophages (Mac 1.3) |
| 0.714523 | 0.497 | 0.059 | 0 | 10 | Osm | Macrophages (Mac 1.3) |
| 0.710863 | 0.472 | 0.037 | 0 | 10 | F13a1 | Macrophages (Mac 1.3) |
| 0.686998 | 0.569 | 0.091 | 0 | 10 | Ms4a6d | Macrophages (Mac 1.3) |
| 0.627541 | 0.497 | 0.055 | 0 | 10 | Msr1 | Macrophages (Mac 1.3) |
| 0.571448 | 0.26 | 0.007 | 0 | 10 | Chil3 | Macrophages (Mac 1.3) |
| 0.537271 | 0.355 | 0.034 | 0 | 10 | Ms4a4c | Macrophages (Mac 1.3) |
| 0.522042 | 0.31 | 0.025 | 0 | 10 | Flrt3 | Macrophages (Mac 1.3) |
| 0.556106 | 0.449 | 0.06 | 1.25E-296 | 10 | Pid1 | Macrophages (Mac 1.3) |
| 0.616505 | 0.584 | 0.109 | 8.98E-276 | 10 | Emilin2 | Macrophages (Mac 1.3) |
| 0.741469 | 0.513 | 0.087 | 1.81E-273 | 10 | Cybb | Macrophages (Mac 1.3) |
| 1.140909 | 0.946 | 0.292 | 5.53E-270 | 10 | Fcer1g | Macrophages (Mac 1.3) |
| 0.862843 | 0.79 | 0.21 | 1.56E-268 | 10 | Alox5ap | Macrophages (Mac 1.3) |
| 0.640783 | 0.481 | 0.079 | 2.40E-261 | 10 | Fcgr2b | Macrophages (Mac 1.3) |
| 1.390799 | 0.828 | 0.303 | 6.19E-260 | 10 | Tgfbi | Macrophages (Mac 1.3) |
| 0.621793 | 0.53 | 0.096 | 1.69E-258 | 10 | Itgam | Macrophages (Mac 1.3) |
| 0.703319 | 0.789 | 0.194 | 2.05E-249 | 10 | Clec4d | Macrophages (Mac 1.3) |
| 1.177667 | 0.933 | 0.371 | 6.21E-248 | 10 | Lgals3 | Macrophages (Mac 1.3) |
| 0.451665 | 0.228 | 0.016 | 6.93E-248 | 10 | Ly6c2 | Macrophages (Mac 1.3) |
| 2.078582 | 0.43 | 0.067 | 3.58E-247 | 10 | Cxcl3 | Macrophages (Mac 1.3) |
| 1.296321 | 0.701 | 0.176 | 7.13E-247 | 10 | Il1rn | Macrophages (Mac 1.3) |
| 0.80642 | 0.804 | 0.214 | 2.10E-246 | 10 | Plek | Macrophages (Mac 1.3) |
| 2.18148 | 0.795 | 0.303 | 3.68E-238 | 10 | Ptgs2 | Macrophages (Mac 1.3) |
| 2.286622 | 0.883 | 0.391 | 5.03E-237 | 10 | Cxcl2 | Macrophages (Mac 1.3) |
| 1.534992 | 0.425 | 0.068 | 1.38E-230 | 10 | Ccl4 | Macrophages (Mac 1.3) |
| 0.441494 | 0.376 | 0.053 | 2.54E-229 | 10 | Jarid2 | Macrophages (Mac 1.3) |
| 0.554814 | 0.522 | 0.103 | 6.59E-226 | 10 | Csf1r | Macrophages (Mac 1.3) |
| 0.766226 | 0.301 | 0.035 | 4.49E-223 | 10 | Il1a | Macrophages (Mac 1.3) |
| 0.7505 | 0.689 | 0.185 | 1.50E-222 | 10 | Plaur | Macrophages (Mac 1.3) |
| 0.623532 | 0.596 | 0.142 | 4.26E-217 | 10 | Arl4c | Macrophages (Mac 1.3) |
| 0.822245 | 0.954 | 0.366 | 1.02E-216 | 10 | Srgn | Macrophages (Mac 1.3) |
| 0.934387 | 0.933 | 0.305 | 3.18E-213 | 10 | Tyrobp | Macrophages (Mac 1.3) |
| 0.925947 | 0.79 | 0.28 | 7.51E-212 | 10 | Cdkn1a | Macrophages (Mac 1.3) |
| 0.817455 | 0.578 | 0.131 | 5.47E-211 | 10 | Wfdc17 | Macrophages (Mac 1.3) |
| 0.948744 | 0.507 | 0.105 | 1.61E-210 | 10 | Ccl6 | Macrophages (Mac 1.3) |
| 1.547397 | 0.895 | 0.511 | 9.16E-208 | 10 | Ier3 | Macrophages (Mac 1.3) |
| 0.603106 | 0.633 | 0.157 | 9.47E-205 | 10 | Lilrb4a | Macrophages (Mac 1.3) |
| 0.442256 | 0.347 | 0.051 | 8.47E-202 | 10 | Ccr2 | Macrophages (Mac 1.3) |
| 0.626775 | 0.63 | 0.172 | 2.44E-201 | 10 | Pitpna | Macrophages (Mac 1.3) |
| 0.574598 | 0.564 | 0.129 | 5.86E-199 | 10 | Mpeg1 | Macrophages (Mac 1.3) |
| 0.780882 | 0.63 | 0.156 | 3.98E-197 | 10 | Slc7a11 | Macrophages (Mac 1.3) |
| 0.866595 | 0.868 | 0.354 | 2.26E-196 | 10 | Cd44 | Macrophages (Mac 1.3) |
| 0.295069 | 0.234 | 0.023 | 6.23E-195 | 10 | Fcgr1 | Macrophages (Mac 1.3) |
| 0.533233 | 0.496 | 0.107 | 1.81E-194 | 10 | Cflar | Macrophages (Mac 1.3) |
| 1.201472 | 0.678 | 0.212 | 8.02E-193 | 10 | Ifi27l2a | Macrophages (Mac 1.3) |
| 0.319259 | 0.244 | 0.027 | 2.42E-187 | 10 | Ccr5 | Macrophages (Mac 1.3) |
| 0.867873 | 0.246 | 0.028 | 1.17E-183 | 10 | Arg1 | Macrophages (Mac 1.3) |
| 1.012318 | 0.798 | 0.287 | 1.74E-181 | 10 | Cd52 | Macrophages (Mac 1.3) |
| 0.85977 | 0.879 | 0.369 | 2.81E-181 | 10 | Pim1 | Macrophages (Mac 1.3) |
| 0.875441 | 0.735 | 0.232 | 9.69E-181 | 10 | Apoe | Macrophages (Mac 1.3) |
| 0.255861 | 0.207 | 0.02 | 2.78E-176 | 10 | Adora2b | Macrophages (Mac 1.3) |
| 1.004829 | 0.822 | 0.352 | 3.54E-176 | 10 | Cstb | Macrophages (Mac 1.3) |
| 0.842084 | 0.906 | 0.453 | 1.02E-175 | 10 | Psap | Macrophages (Mac 1.3) |
| 0.507488 | 0.572 | 0.14 | 1.43E-175 | 10 | Ccr1 | Macrophages (Mac 1.3) |
| 0.605156 | 0.636 | 0.185 | 3.49E-175 | 10 | Efhd2 | Macrophages (Mac 1.3) |
| 0.5417 | 0.419 | 0.085 | 7.07E-174 | 10 | Abca1 | Macrophages (Mac 1.3) |
| 0.777544 | 0.696 | 0.242 | 1.81E-173 | 10 | Zeb2 | Macrophages (Mac 1.3) |
| 0.486953 | 0.494 | 0.113 | 2.89E-169 | 10 | Slc15a3 | Macrophages (Mac 1.3) |
| 0.490472 | 0.496 | 0.119 | 6.98E-168 | 10 | Fam49b | Macrophages (Mac 1.3) |
| 0.532159 | 0.581 | 0.153 | 2.66E-167 | 10 | C5ar1 | Macrophages (Mac 1.3) |
| 0.279807 | 0.216 | 0.023 | 3.89E-166 | 10 | Ccdc71l | Macrophages (Mac 1.3) |
| 0.799029 | 0.876 | 0.384 | 1.87E-165 | 10 | Cyba | Macrophages (Mac 1.3) |
| 1.033415 | 0.946 | 0.565 | 2.15E-164 | 10 | Cebpb | Macrophages (Mac 1.3) |
| 0.9895 | 0.379 | 0.073 | 1.50E-163 | 10 | Ccl3 | Macrophages (Mac 1.3) |
| 1.351446 | 0.996 | 0.948 | 1.82E-163 | 10 | Fth1 | Macrophages (Mac 1.3) |
| 0.325324 | 0.269 | 0.037 | 2.38E-163 | 10 | Ifi207 | Macrophages (Mac 1.3) |
| 0.893864 | 0.606 | 0.188 | 6.86E-161 | 10 | Id2 | Macrophages (Mac 1.3) |
| 0.575735 | 0.659 | 0.204 | 4.58E-160 | 10 | Cd53 | Macrophages (Mac 1.3) |
| 0.631974 | 0.741 | 0.254 | 7.09E-160 | 10 | Laptm5 | Macrophages (Mac 1.3) |
| 0.747553 | 0.816 | 0.371 | 1.72E-159 | 10 | Pkm | Macrophages (Mac 1.3) |
| 0.763099 | 0.44 | 0.1 | 1.15E-156 | 10 | Tnf | Macrophages (Mac 1.3) |
| 0.498803 | 0.578 | 0.156 | 1.24E-155 | 10 | Ptafr | Macrophages (Mac 1.3) |
| 0.300189 | 0.22 | 0.027 | 3.22E-152 | 10 | Ifi202b | Macrophages (Mac 1.3) |
| 0.509844 | 0.392 | 0.085 | 5.48E-151 | 10 | Vegfa | Macrophages (Mac 1.3) |
| 0.723981 | 0.9 | 0.454 | 1.09E-150 | 10 | Mcl1 | Macrophages (Mac 1.3) |
| 0.659579 | 0.681 | 0.251 | 3.05E-150 | 10 | Hif1a | Macrophages (Mac 1.3) |
| 0.283319 | 0.24 | 0.032 | 1.38E-148 | 10 | Uck2 | Macrophages (Mac 1.3) |
| 0.544643 | 0.481 | 0.123 | 3.48E-147 | 10 | Tnfrsf1b | Macrophages (Mac 1.3) |
| 0.495051 | 0.44 | 0.105 | 3.87E-147 | 10 | Adam8 | Macrophages (Mac 1.3) |
| 0.536101 | 0.476 | 0.118 | 6.63E-147 | 10 | Clec4n | Macrophages (Mac 1.3) |
| 0.864555 | 0.762 | 0.349 | 9.53E-146 | 10 | Prdx5 | Macrophages (Mac 1.3) |
| 0.714636 | 0.904 | 0.481 | 2.46E-145 | 10 | Atp6v0c | Macrophages (Mac 1.3) |
| 0.627772 | 0.404 | 0.094 | 2.98E-143 | 10 | Malt1 | Macrophages (Mac 1.3) |
| 0.439809 | 0.43 | 0.099 | 3.17E-143 | 10 | Smox | Macrophages (Mac 1.3) |
| 0.427758 | 0.584 | 0.164 | 1.52E-142 | 10 | Tpd52 | Macrophages (Mac 1.3) |
| 0.549831 | 0.452 | 0.112 | 2.87E-142 | 10 | Cd83 | Macrophages (Mac 1.3) |
| 0.278805 | 0.189 | 0.021 | 5.00E-142 | 10 | Apoc2 | Macrophages (Mac 1.3) |
| 0.887841 | 0.939 | 0.671 | 7.48E-141 | 10 | Nfkbia | Macrophages (Mac 1.3) |
| 0.644845 | 0.662 | 0.237 | 7.76E-141 | 10 | Atp2b1 | Macrophages (Mac 1.3) |
| 0.394909 | 0.413 | 0.095 | 3.89E-140 | 10 | Fcgr3 | Macrophages (Mac 1.3) |
| 0.40628 | 0.424 | 0.101 | 1.41E-138 | 10 | Itgb2 | Macrophages (Mac 1.3) |
| 0.45137 | 0.506 | 0.133 | 6.17E-138 | 10 | Lilr4b | Macrophages (Mac 1.3) |
| 0.523407 | 0.738 | 0.262 | 2.16E-136 | 10 | Lcp1 | Macrophages (Mac 1.3) |
| 0.266763 | 0.199 | 0.024 | 6.66E-136 | 10 | Ifi204 | Macrophages (Mac 1.3) |
| 0.664881 | 0.442 | 0.114 | 1.41E-134 | 10 | Mafb | Macrophages (Mac 1.3) |
| 0.824844 | 0.988 | 0.909 | 4.19E-134 | 10 | Ftl1 | Macrophages (Mac 1.3) |
| 0.533269 | 0.588 | 0.189 | 5.22E-134 | 10 | Ninj1 | Macrophages (Mac 1.3) |
| 0.356813 | 0.53 | 0.143 | 8.35E-133 | 10 | Nlrp3 | Macrophages (Mac 1.3) |
| 0.537369 | 0.614 | 0.204 | 1.73E-132 | 10 | Ehd1 | Macrophages (Mac 1.3) |
| 0.49449 | 0.579 | 0.175 | 3.40E-129 | 10 | Lst1 | Macrophages (Mac 1.3) |
| 0.588283 | 0.692 | 0.267 | 3.68E-126 | 10 | Ucp2 | Macrophages (Mac 1.3) |
| 0.682204 | 0.826 | 0.416 | 1.65E-125 | 10 | Atox1 | Macrophages (Mac 1.3) |
| 0.561796 | 0.677 | 0.261 | 3.71E-124 | 10 | Picalm | Macrophages (Mac 1.3) |
| 0.357082 | 0.293 | 0.056 | 1.43E-123 | 10 | Trf | Macrophages (Mac 1.3) |
| 0.470928 | 0.43 | 0.113 | 1.04E-122 | 10 | Bcl2l11 | Macrophages (Mac 1.3) |
| 0.492992 | 0.53 | 0.168 | 2.39E-122 | 10 | Card19 | Macrophages (Mac 1.3) |
| 0.424617 | 0.433 | 0.113 | 4.59E-122 | 10 | Csf2rb | Macrophages (Mac 1.3) |
| 0.34125 | 0.296 | 0.058 | 9.68E-122 | 10 | Ptpre | Macrophages (Mac 1.3) |
| 0.425652 | 0.409 | 0.104 | 1.63E-120 | 10 | Tgm2 | Macrophages (Mac 1.3) |
| 0.753616 | 0.596 | 0.228 | 3.61E-119 | 10 | Sod2 | Macrophages (Mac 1.3) |
| 0.415991 | 0.448 | 0.124 | 9.13E-119 | 10 | Lyn | Macrophages (Mac 1.3) |
| 0.381866 | 0.361 | 0.084 | 1.15E-118 | 10 | Aif1 | Macrophages (Mac 1.3) |
| 0.462941 | 0.695 | 0.255 | 1.08E-117 | 10 | Marcksl1 | Macrophages (Mac 1.3) |
| 0.848732 | 0.93 | 0.587 | 5.01E-116 | 10 | Btg1 | Macrophages (Mac 1.3) |
| 0.597637 | 0.822 | 0.378 | 1.73E-112 | 10 | Fxyd5 | Macrophages (Mac 1.3) |
| 0.296902 | 0.256 | 0.047 | 2.77E-111 | 10 | Runx3 | Macrophages (Mac 1.3) |
| 0.253826 | 0.21 | 0.032 | 3.99E-111 | 10 | Gpr35 | Macrophages (Mac 1.3) |
| 0.392464 | 0.419 | 0.114 | 5.33E-111 | 10 | Traf1 | Macrophages (Mac 1.3) |
| 0.729167 | 0.843 | 0.455 | 3.66E-110 | 10 | Zfp36 | Macrophages (Mac 1.3) |
| 0.525914 | 0.65 | 0.247 | 5.12E-110 | 10 | Coro1a | Macrophages (Mac 1.3) |
| 0.301882 | 0.26 | 0.05 | 8.15E-109 | 10 | Mxi1 | Macrophages (Mac 1.3) |
| 0.483074 | 0.507 | 0.161 | 1.71E-108 | 10 | Kctd12 | Macrophages (Mac 1.3) |
| 0.676914 | 0.169 | 0.022 | 2.03E-108 | 10 | Areg | Macrophages (Mac 1.3) |
| 0.388826 | 0.385 | 0.102 | 3.10E-106 | 10 | Dab2 | Macrophages (Mac 1.3) |
| 0.328402 | 0.323 | 0.075 | 1.63E-105 | 10 | Kdm7a | Macrophages (Mac 1.3) |
| 0.357057 | 0.418 | 0.117 | 5.25E-105 | 10 | Fyb | Macrophages (Mac 1.3) |
| 0.298101 | 0.275 | 0.057 | 1.04E-104 | 10 | Cd93 | Macrophages (Mac 1.3) |
| 0.367158 | 0.382 | 0.102 | 2.27E-104 | 10 | Diaph1 | Macrophages (Mac 1.3) |
| 0.388657 | 0.5 | 0.162 | 7.95E-101 | 10 | Ncf4 | Macrophages (Mac 1.3) |
| 0.355088 | 0.283 | 0.064 | 1.47E-98 | 10 | Ass1 | Macrophages (Mac 1.3) |
| 0.468898 | 0.304 | 0.073 | 2.22E-98 | 10 | Mmp19 | Macrophages (Mac 1.3) |
| 0.303734 | 0.31 | 0.073 | 2.40E-97 | 10 | Rab11fip1 | Macrophages (Mac 1.3) |
| 0.744531 | 0.883 | 0.631 | 3.49E-97 | 10 | S100a10 | Macrophages (Mac 1.3) |
| 0.597845 | 0.746 | 0.393 | 3.89E-96 | 10 | Tspo | Macrophages (Mac 1.3) |
| 0.400447 | 0.533 | 0.187 | 1.72E-95 | 10 | Spi1 | Macrophages (Mac 1.3) |
| 0.422943 | 0.461 | 0.154 | 1.83E-95 | 10 | Eno1 | Macrophages (Mac 1.3) |
| 0.425746 | 0.494 | 0.172 | 2.16E-95 | 10 | Tgfb1 | Macrophages (Mac 1.3) |
| 0.359402 | 0.364 | 0.102 | 1.13E-93 | 10 | Lrrfip1 | Macrophages (Mac 1.3) |
| 0.459368 | 0.587 | 0.234 | 1.54E-92 | 10 | Arhgdib | Macrophages (Mac 1.3) |
| 0.350227 | 0.359 | 0.099 | 3.08E-91 | 10 | Plin2 | Macrophages (Mac 1.3) |
| 0.322347 | 0.425 | 0.129 | 5.23E-90 | 10 | Cd68 | Macrophages (Mac 1.3) |
| 0.940535 | 0.466 | 0.177 | 1.10E-89 | 10 | Plac8 | Macrophages (Mac 1.3) |
| 0.346765 | 0.575 | 0.209 | 1.25E-89 | 10 | Ptprc | Macrophages (Mac 1.3) |
| 0.63153 | 0.814 | 0.474 | 1.32E-89 | 10 | Ahnak | Macrophages (Mac 1.3) |
| 0.294271 | 0.268 | 0.061 | 2.80E-89 | 10 | Gch1 | Macrophages (Mac 1.3) |
| 0.687833 | 0.993 | 0.898 | 3.48E-89 | 10 | Tmsb4x | Macrophages (Mac 1.3) |
| 0.269601 | 0.246 | 0.052 | 5.51E-89 | 10 | Tmem189 | Macrophages (Mac 1.3) |
| 0.303826 | 0.298 | 0.074 | 8.63E-89 | 10 | Cfp | Macrophages (Mac 1.3) |
| 0.318783 | 0.484 | 0.159 | 1.11E-87 | 10 | Ets2 | Macrophages (Mac 1.3) |
| 0.337151 | 0.343 | 0.096 | 7.28E-87 | 10 | Rbpj | Macrophages (Mac 1.3) |
| 0.527283 | 0.826 | 0.512 | 9.26E-87 | 10 | Arpc1b | Macrophages (Mac 1.3) |
| 0.516976 | 0.835 | 0.535 | 1.15E-86 | 10 | Arpc2 | Macrophages (Mac 1.3) |
| 0.331944 | 0.352 | 0.099 | 2.98E-86 | 10 | Glrx | Macrophages (Mac 1.3) |
| 0.38617 | 0.454 | 0.151 | 5.07E-86 | 10 | Cytip | Macrophages (Mac 1.3) |
| 0.313767 | 0.418 | 0.13 | 3.76E-85 | 10 | Ncf2 | Macrophages (Mac 1.3) |
| 0.39797 | 0.251 | 0.057 | 5.98E-85 | 10 | Tnfsf9 | Macrophages (Mac 1.3) |
| 0.279364 | 0.254 | 0.058 | 7.13E-85 | 10 | Ms4a6b | Macrophages (Mac 1.3) |
| 0.58859 | 0.784 | 0.465 | 1.56E-84 | 10 | Npc2 | Macrophages (Mac 1.3) |
| 0.387352 | 0.524 | 0.201 | 2.10E-84 | 10 | Capza2 | Macrophages (Mac 1.3) |
| 0.535829 | 0.81 | 0.576 | 2.68E-84 | 10 | Sem1 | Macrophages (Mac 1.3) |
| 0.272724 | 0.383 | 0.11 | 2.70E-84 | 10 | Slc16a3 | Macrophages (Mac 1.3) |
| 0.413137 | 0.641 | 0.282 | 1.09E-83 | 10 | Arpc5 | Macrophages (Mac 1.3) |
| 0.549087 | 0.612 | 0.272 | 2.07E-83 | 10 | Ctsc | Macrophages (Mac 1.3) |
| 0.525229 | 0.674 | 0.323 | 9.90E-83 | 10 | Ctsz | Macrophages (Mac 1.3) |
| 0.256714 | 0.207 | 0.041 | 9.12E-81 | 10 | St3gal1 | Macrophages (Mac 1.3) |
| 0.688699 | 0.993 | 0.959 | 1.28E-80 | 10 | Actb | Macrophages (Mac 1.3) |
| 0.516991 | 0.587 | 0.255 | 1.13E-78 | 10 | Tnfaip3 | Macrophages (Mac 1.3) |
| 0.42824 | 0.579 | 0.247 | 1.64E-78 | 10 | Sdcbp | Macrophages (Mac 1.3) |
| 0.319132 | 0.543 | 0.203 | 2.70E-78 | 10 | Gmfg | Macrophages (Mac 1.3) |
| 0.321595 | 0.256 | 0.064 | 6.85E-76 | 10 | Mdm2 | Macrophages (Mac 1.3) |
| 0.699645 | 0.452 | 0.168 | 2.38E-75 | 10 | Ifitm1 | Macrophages (Mac 1.3) |
| 0.487309 | 0.684 | 0.354 | 1.35E-74 | 10 | Iqgap1 | Macrophages (Mac 1.3) |
| 0.292089 | 0.325 | 0.097 | 1.11E-71 | 10 | Snx18 | Macrophages (Mac 1.3) |
| 0.308767 | 0.374 | 0.124 | 1.90E-70 | 10 | Mapkapk2 | Macrophages (Mac 1.3) |
| 0.338549 | 0.464 | 0.18 | 7.04E-68 | 10 | Cotl1 | Macrophages (Mac 1.3) |
| 0.43518 | 0.4 | 0.151 | 2.45E-67 | 10 | Pgk1 | Macrophages (Mac 1.3) |
| 0.282148 | 0.319 | 0.099 | 5.30E-67 | 10 | Atp1b3 | Macrophages (Mac 1.3) |
| 0.373033 | 0.475 | 0.191 | 7.82E-67 | 10 | Atp6v1e1 | Macrophages (Mac 1.3) |
| 0.277636 | 0.277 | 0.078 | 9.71E-67 | 10 | Got1 | Macrophages (Mac 1.3) |
| 0.30872 | 0.338 | 0.109 | 1.09E-66 | 10 | Rilpl2 | Macrophages (Mac 1.3) |
| 0.311668 | 0.404 | 0.144 | 8.14E-66 | 10 | Rel | Macrophages (Mac 1.3) |
| 0.390855 | 0.436 | 0.162 | 1.57E-64 | 10 | Lgmn | Macrophages (Mac 1.3) |
| 0.306113 | 0.349 | 0.118 | 3.66E-64 | 10 | Gna13 | Macrophages (Mac 1.3) |
| 0.305829 | 0.305 | 0.096 | 8.94E-64 | 10 | Trps1 | Macrophages (Mac 1.3) |
| 0.482046 | 0.596 | 0.298 | 2.48E-63 | 10 | Ly6e | Macrophages (Mac 1.3) |
| 0.373559 | 0.59 | 0.279 | 3.46E-63 | 10 | Rap1b | Macrophages (Mac 1.3) |
| 0.389767 | 0.457 | 0.187 | 9.53E-63 | 10 | Tiparp | Macrophages (Mac 1.3) |
| 0.360502 | 0.516 | 0.226 | 5.06E-61 | 10 | Capg | Macrophages (Mac 1.3) |
| 0.357978 | 0.644 | 0.322 | 8.56E-61 | 10 | Msn | Macrophages (Mac 1.3) |
| 0.436393 | 0.569 | 0.275 | 9.05E-61 | 10 | Sat1 | Macrophages (Mac 1.3) |
| 0.367222 | 0.401 | 0.156 | 1.06E-60 | 10 | Rnh1 | Macrophages (Mac 1.3) |
| 0.316443 | 0.343 | 0.119 | 1.69E-60 | 10 | Metrnl | Macrophages (Mac 1.3) |
| 0.409262 | 0.488 | 0.216 | 3.22E-60 | 10 | Esd | Macrophages (Mac 1.3) |
| 0.254751 | 0.347 | 0.117 | 2.12E-59 | 10 | Rnf149 | Macrophages (Mac 1.3) |
| 0.25732 | 0.286 | 0.089 | 7.16E-59 | 10 | Rab5c | Macrophages (Mac 1.3) |
| 0.294412 | 0.379 | 0.139 | 9.24E-59 | 10 | Tgif1 | Macrophages (Mac 1.3) |
| 0.286655 | 0.325 | 0.109 | 9.63E-59 | 10 | Sirpa | Macrophages (Mac 1.3) |
| 0.315449 | 0.425 | 0.17 | 1.80E-58 | 10 | Actr2 | Macrophages (Mac 1.3) |
| 0.28327 | 0.401 | 0.152 | 1.83E-58 | 10 | Emb | Macrophages (Mac 1.3) |
| 0.460646 | 0.912 | 0.651 | 3.78E-58 | 10 | B2m | Macrophages (Mac 1.3) |
| 0.310891 | 0.528 | 0.231 | 1.29E-57 | 10 | Cox17 | Macrophages (Mac 1.3) |
| 0.357414 | 0.118 | 0.019 | 3.21E-57 | 10 | Csf3 | Macrophages (Mac 1.3) |
| 0.582565 | 0.798 | 0.605 | 1.21E-56 | 10 | Gapdh | Macrophages (Mac 1.3) |
| 0.590148 | 0.16 | 0.034 | 1.65E-56 | 10 | Il10 | Macrophages (Mac 1.3) |
| 0.357192 | 0.786 | 0.46 | 2.15E-56 | 10 | Arpc3 | Macrophages (Mac 1.3) |
| 0.470324 | 0.924 | 0.643 | 2.55E-56 | 10 | H2-D1 | Macrophages (Mac 1.3) |
| 0.383934 | 0.581 | 0.276 | 5.95E-56 | 10 | Tnfaip2 | Macrophages (Mac 1.3) |
| 0.580817 | 0.603 | 0.343 | 1.87E-55 | 10 | Ifrd1 | Macrophages (Mac 1.3) |
| 0.438667 | 0.894 | 0.725 | 5.89E-55 | 10 | Calm1 | Macrophages (Mac 1.3) |
| 0.329757 | 0.668 | 0.358 | 3.83E-54 | 10 | Nfkbiz | Macrophages (Mac 1.3) |
| 0.489631 | 0.606 | 0.329 | 7.93E-54 | 10 | Socs3 | Macrophages (Mac 1.3) |
| 0.506304 | 0.307 | 0.109 | 9.24E-54 | 10 | Hilpda | Macrophages (Mac 1.3) |
| 0.31225 | 0.328 | 0.117 | 1.63E-53 | 10 | Dusp5 | Macrophages (Mac 1.3) |
| 0.418071 | 0.867 | 0.526 | 6.42E-53 | 10 | S100a4 | Macrophages (Mac 1.3) |
| 0.353608 | 0.624 | 0.314 | 8.43E-53 | 10 | Kdm6b | Macrophages (Mac 1.3) |
| 0.25924 | 0.377 | 0.144 | 5.20E-52 | 10 | Pde4b | Macrophages (Mac 1.3) |
| 0.342794 | 0.451 | 0.204 | 1.97E-49 | 10 | Rbms1 | Macrophages (Mac 1.3) |
| 0.376297 | 0.497 | 0.247 | 8.93E-49 | 10 | Aprt | Macrophages (Mac 1.3) |
| 0.309302 | 0.554 | 0.278 | 5.42E-48 | 10 | Atp6v0b | Macrophages (Mac 1.3) |
| 0.259519 | 0.305 | 0.11 | 1.98E-46 | 10 | Cxcl16 | Macrophages (Mac 1.3) |
| 0.933995 | 0.439 | 0.203 | 3.91E-46 | 10 | Slpi | Macrophages (Mac 1.3) |
| 0.284844 | 0.362 | 0.147 | 1.46E-45 | 10 | Txnrd1 | Macrophages (Mac 1.3) |
| 0.341312 | 0.784 | 0.524 | 4.39E-45 | 10 | Cdc42 | Macrophages (Mac 1.3) |
| 0.465327 | 0.978 | 0.969 | 1.76E-44 | 10 | Fau | Macrophages (Mac 1.3) |
| 0.317743 | 0.349 | 0.138 | 2.52E-44 | 10 | Pla2g7 | Macrophages (Mac 1.3) |
| 0.268889 | 0.349 | 0.14 | 2.74E-44 | 10 | Gm2a | Macrophages (Mac 1.3) |
| 0.267507 | 0.373 | 0.156 | 1.11E-42 | 10 | Cxcr4 | Macrophages (Mac 1.3) |
| 0.360781 | 0.702 | 0.445 | 7.23E-42 | 10 | Gpx1 | Macrophages (Mac 1.3) |
| 0.288132 | 0.362 | 0.156 | 2.01E-41 | 10 | Ptbp3 | Macrophages (Mac 1.3) |
| 0.325026 | 0.439 | 0.212 | 3.23E-41 | 10 | Prdx6 | Macrophages (Mac 1.3) |
| 0.327358 | 0.919 | 0.861 | 1.04E-40 | 10 | H3f3a | Macrophages (Mac 1.3) |
| 0.275573 | 0.513 | 0.26 | 1.22E-40 | 10 | Actr3 | Macrophages (Mac 1.3) |
| 0.266737 | 0.298 | 0.117 | 2.63E-40 | 10 | Irf2bp2 | Macrophages (Mac 1.3) |
| 0.340494 | 0.707 | 0.464 | 3.09E-39 | 10 | Clic1 | Macrophages (Mac 1.3) |
| 0.314876 | 0.457 | 0.234 | 1.05E-38 | 10 | H2afj | Macrophages (Mac 1.3) |
| 0.324068 | 0.722 | 0.467 | 4.81E-38 | 10 | Sh3bgrl3 | Macrophages (Mac 1.3) |
| 0.252094 | 0.506 | 0.259 | 5.28E-38 | 10 | Ostf1 | Macrophages (Mac 1.3) |
| 0.285591 | 0.53 | 0.282 | 2.60E-37 | 10 | Nfe2l2 | Macrophages (Mac 1.3) |
| 0.395618 | 0.948 | 0.894 | 4.55E-37 | 10 | H3f3b | Macrophages (Mac 1.3) |
| 0.330375 | 0.259 | 0.096 | 1.48E-36 | 10 | Rgs1 | Macrophages (Mac 1.3) |
| 0.282858 | 0.763 | 0.523 | 1.80E-35 | 10 | Ctsb | Macrophages (Mac 1.3) |
| 0.435281 | 0.939 | 0.863 | 3.43E-34 | 10 | Actg1 | Macrophages (Mac 1.3) |
| 0.262655 | 0.394 | 0.191 | 4.40E-34 | 10 | Ppp2ca | Macrophages (Mac 1.3) |
| 0.256083 | 0.256 | 0.1 | 7.24E-34 | 10 | Samhd1 | Macrophages (Mac 1.3) |
| 0.695913 | 0.762 | 0.545 | 9.50E-34 | 10 | Fn1 | Macrophages (Mac 1.3) |
| 0.465802 | 0.278 | 0.12 | 1.52E-33 | 10 | Pdpn | Macrophages (Mac 1.3) |
| 0.311444 | 0.626 | 0.41 | 3.67E-32 | 10 | Ndufb1-ps | Macrophages (Mac 1.3) |
| 0.332806 | 0.735 | 0.528 | 6.46E-31 | 10 | Emp3 | Macrophages (Mac 1.3) |
| 1.272693 | 0.56 | 0.4 | 9.17E-31 | 10 | Cxcl1 | Macrophages (Mac 1.3) |
| 0.687843 | 0.948 | 0.781 | 1.91E-30 | 10 | Gm42418 | Macrophages (Mac 1.3) |
| 0.267792 | 0.491 | 0.274 | 2.08E-30 | 10 | Tpm3 | Macrophages (Mac 1.3) |
| 0.794972 | 0.735 | 0.634 | 1.00E-29 | 10 | Ifitm3 | Macrophages (Mac 1.3) |
| 0.356937 | 0.677 | 0.451 | 2.21E-29 | 10 | Dusp1 | Macrophages (Mac 1.3) |
| 0.266898 | 0.72 | 0.501 | 3.84E-29 | 10 | Rhoa | Macrophages (Mac 1.3) |
| 0.28401 | 0.906 | 0.79 | 2.20E-28 | 10 | Pfn1 | Macrophages (Mac 1.3) |
| 0.405109 | 0.425 | 0.241 | 2.63E-28 | 10 | Sdc4 | Macrophages (Mac 1.3) |
| 0.280679 | 0.847 | 0.686 | 2.69E-28 | 10 | Cfl1 | Macrophages (Mac 1.3) |
| 0.260352 | 0.305 | 0.142 | 2.87E-28 | 10 | BC005537 | Macrophages (Mac 1.3) |
| 0.345399 | 0.557 | 0.356 | 4.47E-28 | 10 | Emp1 | Macrophages (Mac 1.3) |
| 0.299053 | 0.358 | 0.186 | 9.98E-27 | 10 | Vcan | Macrophages (Mac 1.3) |
| 0.301824 | 0.383 | 0.205 | 1.49E-26 | 10 | Map4k4 | Macrophages (Mac 1.3) |
| 0.250226 | 0.446 | 0.244 | 8.08E-26 | 10 | Grn | Macrophages (Mac 1.3) |
| 0.287308 | 0.889 | 0.707 | 8.63E-25 | 10 | Junb | Macrophages (Mac 1.3) |
| 0.350263 | 0.822 | 0.731 | 5.42E-24 | 10 | Ifitm2 | Macrophages (Mac 1.3) |
| 0.383748 | 0.75 | 0.622 | 3.90E-22 | 10 | Anxa2 | Macrophages (Mac 1.3) |
| 0.394341 | 0.287 | 0.147 | 9.18E-21 | 10 | Hmox1 | Macrophages (Mac 1.3) |
| 0.293633 | 0.4 | 0.239 | 8.44E-20 | 10 | Basp1 | Macrophages (Mac 1.3) |
| 0.290989 | 0.319 | 0.172 | 1.21E-19 | 10 | Rgcc | Macrophages (Mac 1.3) |
| 0.259689 | 0.554 | 0.374 | 5.93E-17 | 10 | Klf6 | Macrophages (Mac 1.3) |
| 0.283782 | 0.539 | 0.397 | 5.64E-15 | 10 | Aldoa | Macrophages (Mac 1.3) |
| 0.299661 | 0.371 | 0.235 | 2.61E-14 | 10 | Zfp36l2 | Macrophages (Mac 1.3) |
| 0.278581 | 0.707 | 0.599 | 2.04E-11 | 10 | Txn1 | Macrophages (Mac 1.3) |
| 0.3669 | 0.284 | 0.189 | 5.87E-06 | 10 | Spp1 | Macrophages (Mac 1.3) |
| 1.663529 | 0.73 | 0.059 | 0 | 11 | Cryab | Neural |
| 1.508851 | 0.202 | 0.001 | 0 | 11 | Mpz | Neural |
| 1.217598 | 0.54 | 0.002 | 0 | 11 | Plp1 | Neural |
| 0.936953 | 0.589 | 0.053 | 0 | 11 | Gpm6b | Neural |
| 0.888901 | 0.718 | 0.133 | 0 | 11 | Crip2 | Neural |
| 0.850332 | 0.414 | 0.016 | 0 | 11 | Itih5 | Neural |
| 0.744497 | 0.343 | 0.001 | 0 | 11 | Egfl8 | Neural |
| 0.735675 | 0.465 | 0.041 | 0 | 11 | Ccnd1 | Neural |
| 0.700335 | 0.487 | 0.05 | 0 | 11 | Dag1 | Neural |
| 0.678688 | 0.469 | 0.053 | 0 | 11 | Arpc1a | Neural |
| 0.671816 | 0.365 | 0.026 | 0 | 11 | Gatm | Neural |
| 0.620837 | 0.343 | 0.002 | 0 | 11 | Ank3 | Neural |
| 0.500277 | 0.267 | 0.004 | 0 | 11 | Itgb8 | Neural |
| 0.494035 | 0.362 | 0.031 | 0 | 11 | Tubb2b | Neural |
| 0.456999 | 0.192 | 0.001 | 0 | 11 | Sostdc1 | Neural |
| 0.427501 | 0.272 | 0.001 | 0 | 11 | Cadm4 | Neural |
| 0.388075 | 0.272 | 0 | 0 | 11 | Foxd3 | Neural |
| 0.350698 | 0.194 | 0.002 | 0 | 11 | Gldn | Neural |
| 0.340215 | 0.158 | 0.003 | 0 | 11 | Gap43 | Neural |
| 0.336538 | 0.241 | 0.008 | 0 | 11 | Pcbp4 | Neural |
| 0.328578 | 0.158 | 0 | 0 | 11 | Mal | Neural |
| 0.325159 | 0.166 | 0 | 0 | 11 | Kcna1 | Neural |
| 0.284746 | 0.188 | 0.001 | 0 | 11 | Ngfr | Neural |
| 0.250003 | 0.166 | 0.002 | 0 | 11 | Uchl1 | Neural |
| 0.282969 | 0.195 | 0.008 | 4.37E-286 | 11 | Nes | Neural |
| 0.679153 | 0.472 | 0.067 | 2.97E-276 | 11 | Pdlim4 | Neural |
| 0.334425 | 0.234 | 0.014 | 3.25E-274 | 11 | Itga6 | Neural |
| 0.31911 | 0.107 | 0.001 | 1.96E-269 | 11 | Egfl7 | Neural |
| 0.748156 | 0.469 | 0.072 | 1.31E-254 | 11 | Crlf1 | Neural |
| 1.590871 | 0.796 | 0.301 | 9.04E-252 | 11 | Dbi | Neural |
| 0.406174 | 0.307 | 0.03 | 2.89E-246 | 11 | Emp2 | Neural |
| 0.32189 | 0.197 | 0.011 | 1.07E-243 | 11 | Nkd2 | Neural |
| 0.382403 | 0.304 | 0.029 | 1.88E-243 | 11 | Cd151 | Neural |
| 0.311439 | 0.224 | 0.016 | 9.11E-225 | 11 | Ldhb | Neural |
| 0.88255 | 0.458 | 0.08 | 1.04E-217 | 11 | Stmn1 | Neural |
| 1.038758 | 0.725 | 0.211 | 9.40E-214 | 11 | Col18a1 | Neural |
| 0.405559 | 0.321 | 0.041 | 2.43E-195 | 11 | S100a16 | Neural |
| 0.332128 | 0.195 | 0.014 | 1.10E-193 | 11 | Cnp | Neural |
| 0.378812 | 0.255 | 0.026 | 1.90E-188 | 11 | Hbegf | Neural |
| 0.729038 | 0.681 | 0.215 | 5.84E-182 | 11 | Fkbp1a | Neural |
| 1.586764 | 0.638 | 0.212 | 9.02E-176 | 11 | Tm4sf1 | Neural |
| 0.552892 | 0.435 | 0.084 | 2.85E-170 | 11 | Ckb | Neural |
| 0.577106 | 0.47 | 0.105 | 2.85E-162 | 11 | Qk | Neural |
| 0.37176 | 0.284 | 0.041 | 3.06E-150 | 11 | Map1b | Neural |
| 0.478901 | 0.401 | 0.081 | 1.02E-148 | 11 | Sox4 | Neural |
| 0.378358 | 0.222 | 0.026 | 4.67E-145 | 11 | Cd59a | Neural |
| 0.377906 | 0.338 | 0.06 | 4.54E-143 | 11 | Pfdn1 | Neural |
| 1.047362 | 0.413 | 0.099 | 3.94E-129 | 11 | Pmp22 | Neural |
| 1.026263 | 0.705 | 0.323 | 4.60E-128 | 11 | Tuba1a | Neural |
| 0.620868 | 0.377 | 0.085 | 2.79E-126 | 11 | Prnp | Neural |
| 0.288735 | 0.195 | 0.023 | 4.17E-126 | 11 | Gas7 | Neural |
| 0.561082 | 0.576 | 0.189 | 2.16E-124 | 11 | Rhoc | Neural |
| 0.279697 | 0.211 | 0.029 | 1.31E-116 | 11 | Cfl2 | Neural |
| 0.93477 | 0.879 | 0.516 | 2.75E-116 | 11 | Cd9 | Neural |
| 0.95463 | 0.978 | 0.829 | 8.23E-115 | 11 | Vim | Neural |
| 0.448163 | 0.438 | 0.116 | 1.30E-113 | 11 | Pdgfa | Neural |
| 0.403472 | 0.346 | 0.078 | 7.10E-110 | 11 | Dst | Neural |
| 0.489277 | 0.47 | 0.138 | 6.84E-109 | 11 | Sptbn1 | Neural |
| 0.963281 | 0.791 | 0.515 | 2.66E-108 | 11 | Arpc1b | Neural |
| 0.270107 | 0.233 | 0.038 | 2.20E-106 | 11 | Adk | Neural |
| 0.652773 | 0.793 | 0.469 | 3.90E-102 | 11 | Selenof | Neural |
| 0.578163 | 0.598 | 0.243 | 1.72E-100 | 11 | Ppp1r14b | Neural |
| 0.481293 | 0.428 | 0.127 | 4.29E-98 | 11 | Tnfrsf12a | Neural |
| 0.662239 | 0.749 | 0.399 | 3.43E-94 | 11 | Cd81 | Neural |
| 0.326809 | 0.292 | 0.064 | 5.22E-94 | 11 | Ctnna1 | Neural |
| 0.79315 | 0.958 | 0.84 | 4.14E-93 | 11 | Ptma | Neural |
| 0.321711 | 0.316 | 0.075 | 2.76E-91 | 11 | Prmt1 | Neural |
| 0.393336 | 0.413 | 0.12 | 1.11E-85 | 11 | Timp3 | Neural |
| 0.588096 | 0.45 | 0.155 | 1.84E-83 | 11 | Prss23 | Neural |
| 0.522596 | 0.557 | 0.229 | 3.43E-83 | 11 | Ndfip1 | Neural |
| 0.513108 | 0.664 | 0.322 | 6.28E-83 | 11 | Ywhae | Neural |
| 0.33046 | 0.316 | 0.08 | 1.01E-82 | 11 | Aopep | Neural |
| 0.664945 | 0.9 | 0.664 | 1.98E-82 | 11 | mt-Nd2 | Neural |
| 0.703812 | 0.944 | 0.837 | 4.42E-81 | 11 | Ppia | Neural |
| 0.313758 | 0.307 | 0.078 | 6.36E-81 | 11 | Vdac3 | Neural |
| 0.683992 | 0.99 | 0.924 | 3.15E-78 | 11 | mt-Atp6 | Neural |
| 0.340839 | 0.375 | 0.113 | 5.59E-76 | 11 | Dync1i2 | Neural |
| 0.329688 | 0.177 | 0.03 | 1.45E-75 | 11 | Cxcl10 | Neural |
| 0.256062 | 0.205 | 0.04 | 6.05E-75 | 11 | Mcam | Neural |
| 0.289439 | 0.285 | 0.074 | 7.83E-72 | 11 | Rnf7 | Neural |
| 0.473566 | 0.304 | 0.085 | 1.53E-71 | 11 | Fxyd1 | Neural |
| 0.258282 | 0.246 | 0.057 | 3.10E-71 | 11 | Dctn2 | Neural |
| 0.405054 | 0.514 | 0.214 | 2.35E-68 | 11 | Rtraf | Neural |
| 0.611749 | 0.813 | 0.62 | 3.04E-66 | 11 | Anxa2 | Neural |
| 0.348592 | 0.351 | 0.112 | 4.16E-66 | 11 | Tubb2a | Neural |
| 0.451078 | 0.535 | 0.227 | 6.95E-66 | 11 | Capg | Neural |
| 0.263175 | 0.278 | 0.075 | 1.37E-64 | 11 | Siva1 | Neural |
| 0.549718 | 0.698 | 0.397 | 1.15E-63 | 11 | Tspo | Neural |
| 0.364914 | 0.334 | 0.106 | 6.93E-62 | 11 | Rhob | Neural |
| 0.326025 | 0.209 | 0.049 | 6.87E-60 | 11 | Scd2 | Neural |
| 0.278123 | 0.209 | 0.048 | 7.87E-60 | 11 | Cuedc2 | Neural |
| 0.347774 | 0.419 | 0.16 | 8.06E-59 | 11 | Ywhaq | Neural |
| 0.438116 | 0.672 | 0.382 | 4.66E-58 | 11 | Sumo2 | Neural |
| 0.545614 | 0.896 | 0.774 | 6.86E-58 | 11 | Hsp90ab1 | Neural |
| 0.557221 | 0.74 | 0.519 | 1.55E-56 | 11 | Anxa5 | Neural |
| 0.574747 | 0.752 | 0.522 | 3.52E-56 | 11 | Tagln2 | Neural |
| 0.648888 | 0.654 | 0.398 | 5.99E-56 | 11 | Tubb5 | Neural |
| 0.439655 | 0.654 | 0.372 | 8.82E-56 | 11 | Prdx2 | Neural |
| 0.302451 | 0.224 | 0.058 | 3.21E-55 | 11 | Tppp3 | Neural |
| 0.402079 | 0.525 | 0.242 | 4.84E-54 | 11 | Cavin3 | Neural |
| 0.489864 | 0.104 | 0.015 | 2.09E-53 | 11 | Mbp | Neural |
| 0.31297 | 0.357 | 0.128 | 1.76E-52 | 11 | Actn4 | Neural |
| 0.333503 | 0.45 | 0.187 | 2.90E-52 | 11 | Plec | Neural |
| 0.456547 | 0.689 | 0.405 | 3.38E-52 | 11 | Itgb1 | Neural |
| 0.555502 | 0.973 | 0.867 | 1.53E-51 | 11 | mt-Cytb | Neural |
| 0.299639 | 0.367 | 0.138 | 1.49E-49 | 11 | St13 | Neural |
| 0.432483 | 0.694 | 0.444 | 1.78E-49 | 11 | Hint1 | Neural |
| 0.386805 | 0.343 | 0.13 | 2.94E-49 | 11 | Tuba1b | Neural |
| 0.276745 | 0.278 | 0.088 | 3.83E-49 | 11 | Pam | Neural |
| 0.372675 | 0.418 | 0.177 | 3.89E-49 | 11 | Ranbp1 | Neural |
| 0.25064 | 0.282 | 0.091 | 1.85E-48 | 11 | Vdac1 | Neural |
| 0.304104 | 0.384 | 0.152 | 1.56E-47 | 11 | Ywhah | Neural |
| 0.250275 | 0.19 | 0.048 | 1.88E-47 | 11 | Utrn | Neural |
| 0.335095 | 0.46 | 0.206 | 2.82E-47 | 11 | Capns1 | Neural |
| 0.32491 | 0.358 | 0.138 | 1.00E-46 | 11 | Hspg2 | Neural |
| 0.465914 | 0.548 | 0.296 | 2.21E-46 | 11 | Ran | Neural |
| 0.426297 | 0.757 | 0.489 | 1.49E-45 | 11 | Hmgb1 | Neural |
| 0.482031 | 0.766 | 0.555 | 3.26E-45 | 11 | Dynll1 | Neural |
| 0.298734 | 0.413 | 0.177 | 3.81E-44 | 11 | Psmb2 | Neural |
| 0.36223 | 0.577 | 0.314 | 5.33E-43 | 11 | Tmed10 | Neural |
| 0.34374 | 0.496 | 0.245 | 2.57E-42 | 11 | Set | Neural |
| 0.456629 | 0.847 | 0.633 | 3.98E-42 | 11 | S100a10 | Neural |
| 0.528664 | 0.896 | 0.783 | 1.28E-41 | 11 | mt-Nd1 | Neural |
| 0.526608 | 0.951 | 0.867 | 1.35E-41 | 11 | Rplp0 | Neural |
| 0.37934 | 0.564 | 0.312 | 1.10E-40 | 11 | Psma7 | Neural |
| 0.361798 | 0.638 | 0.377 | 2.27E-39 | 11 | Serbp1 | Neural |
| 0.42914 | 0.91 | 0.802 | 1.28E-38 | 11 | mt-Nd4 | Neural |
| 0.406112 | 0.674 | 0.447 | 1.57E-38 | 11 | Marcks | Neural |
| 0.283073 | 0.411 | 0.186 | 1.85E-38 | 11 | Vdac2 | Neural |
| 0.266888 | 0.346 | 0.144 | 4.48E-38 | 11 | Ifi27 | Neural |
| 0.275388 | 0.396 | 0.176 | 1.87E-37 | 11 | 1810037I17Rik | Neural |
| 0.356375 | 0.577 | 0.326 | 3.66E-37 | 11 | Msn | Neural |
| 0.25185 | 0.345 | 0.145 | 1.77E-35 | 11 | Jpt1 | Neural |
| 0.257691 | 0.328 | 0.137 | 2.91E-34 | 11 | Pdap1 | Neural |
| 0.269055 | 0.43 | 0.209 | 9.62E-34 | 11 | Txndc17 | Neural |
| 0.337307 | 0.508 | 0.284 | 1.59E-33 | 11 | Hmgn1 | Neural |
| 0.297388 | 0.423 | 0.208 | 9.34E-33 | 11 | Cyb5r3 | Neural |
| 0.33344 | 0.666 | 0.445 | 9.40E-33 | 11 | Gpx4 | Neural |
| 0.436561 | 0.829 | 0.632 | 1.15E-32 | 11 | Lmna | Neural |
| 0.381162 | 0.302 | 0.126 | 1.83E-32 | 11 | Aqp1 | Neural |
| 0.348639 | 0.209 | 0.073 | 3.82E-31 | 11 | Ptn | Neural |
| 0.371436 | 0.981 | 0.926 | 4.91E-31 | 11 | mt-Co2 | Neural |
| 0.423094 | 0.211 | 0.076 | 9.50E-30 | 11 | Apod | Neural |
| 0.359613 | 0.878 | 0.763 | 2.07E-29 | 11 | Rpl3 | Neural |
| 0.266626 | 0.399 | 0.198 | 2.10E-29 | 11 | Psmb5 | Neural |
| 0.272852 | 0.413 | 0.208 | 2.34E-29 | 11 | Psmb3 | Neural |
| 0.282451 | 0.435 | 0.226 | 2.92E-29 | 11 | Snrpb | Neural |
| 0.261508 | 0.426 | 0.218 | 1.57E-28 | 11 | Nap1l1 | Neural |
| 0.399211 | 0.983 | 0.926 | 2.84E-28 | 11 | mt-Co3 | Neural |
| 0.317789 | 0.788 | 0.63 | 1.28E-27 | 11 | Chchd2 | Neural |
| 0.294652 | 0.613 | 0.392 | 1.63E-27 | 11 | Atp5b | Neural |
| 0.352776 | 0.66 | 0.467 | 1.82E-27 | 11 | Clic1 | Neural |
| 0.264479 | 0.479 | 0.265 | 4.51E-27 | 11 | Psma3 | Neural |
| 0.251222 | 0.396 | 0.201 | 5.44E-27 | 11 | Psma2 | Neural |
| 0.367203 | 0.778 | 0.69 | 8.11E-27 | 11 | Cfl1 | Neural |
| 0.26196 | 0.413 | 0.216 | 1.12E-26 | 11 | Anp32b | Neural |
| 0.412098 | 0.9 | 0.783 | 4.44E-25 | 11 | Rps2 | Neural |
| 0.295606 | 0.788 | 0.654 | 8.64E-25 | 11 | Btf3 | Neural |
| 0.304629 | 0.652 | 0.44 | 1.11E-24 | 11 | Rps27l | Neural |
| 0.254091 | 0.424 | 0.23 | 1.89E-24 | 11 | Edf1 | Neural |
| 0.35961 | 0.981 | 0.899 | 2.46E-24 | 11 | Tmsb4x | Neural |
| 0.284481 | 0.256 | 0.111 | 2.78E-24 | 11 | Ndrg1 | Neural |
| 0.279822 | 0.421 | 0.236 | 3.18E-24 | 11 | Nme1 | Neural |
| 0.308233 | 0.747 | 0.58 | 5.27E-24 | 11 | Prdx1 | Neural |
| 0.385168 | 0.452 | 0.263 | 5.79E-24 | 11 | Fbln2 | Neural |
| 0.258854 | 0.474 | 0.275 | 1.20E-23 | 11 | Mrfap1 | Neural |
| 0.399478 | 0.64 | 0.442 | 2.45E-23 | 11 | Pmepa1 | Neural |
| 0.353428 | 0.681 | 0.5 | 8.49E-23 | 11 | mt-Nd3 | Neural |
| 0.304291 | 0.61 | 0.418 | 1.79E-22 | 11 | Hsp90aa1 | Neural |
| 0.347982 | 0.988 | 0.98 | 2.29E-22 | 11 | Tpt1 | Neural |
| 0.323555 | 0.713 | 0.542 | 1.44E-21 | 11 | Anxa1 | Neural |
| 0.331014 | 0.676 | 0.533 | 2.20E-21 | 11 | Eif5a | Neural |
| 0.26424 | 0.399 | 0.223 | 9.17E-21 | 11 | Hspe1 | Neural |
| 0.251542 | 0.441 | 0.259 | 1.97E-20 | 11 | Rtn4 | Neural |
| 0.282776 | 0.823 | 0.702 | 2.66E-20 | 11 | Naca | Neural |
| 0.316407 | 0.986 | 0.942 | 7.01E-19 | 11 | mt-Co1 | Neural |
| 0.256863 | 0.91 | 0.838 | 1.51E-18 | 11 | Rps3 | Neural |
| 0.265996 | 0.839 | 0.782 | 3.57E-18 | 11 | Oaz1 | Neural |
| 0.304303 | 0.321 | 0.171 | 3.82E-18 | 11 | Atf3 | Neural |
| 0.348375 | 0.837 | 0.647 | 4.52E-18 | 11 | Cd63 | Neural |
| 0.401979 | 0.244 | 0.125 | 1.03E-14 | 11 | Gng11 | Neural |
| 0.38407 | 0.643 | 0.502 | 1.60E-12 | 11 | Igfbp7 | Neural |
| 0.310193 | 0.903 | 0.866 | 2.15E-12 | 11 | Ubb | Neural |
| 0.278104 | 0.664 | 0.532 | 3.15E-12 | 11 | Emp3 | Neural |
| 0.306914 | 0.827 | 0.738 | 4.03E-09 | 11 | Lgals1 | Neural |
| 0.257852 | 0.467 | 0.334 | 5.03E-08 | 11 | Csrp2 | Neural |
| 2.750973 | 0.878 | 0.081 | 0 | 12 | Mmp13 | Myofibroblast (Myo 1.2) |
| 1.925312 | 0.922 | 0.247 | 0 | 12 | Gpx3 | Myofibroblast (Myo 1.2) |
| 1.320107 | 0.938 | 0.327 | 1.41E-216 | 12 | Col12a1 | Myofibroblast (Myo 1.2) |
| 0.70494 | 0.67 | 0.148 | 7.04E-208 | 12 | Lrrc15 | Myofibroblast (Myo 1.2) |
| 1.387564 | 0.972 | 0.392 | 2.04E-178 | 12 | Lum | Myofibroblast (Myo 1.2) |
| 1.493335 | 1 | 0.545 | 5.15E-174 | 12 | Col1a1 | Myofibroblast (Myo 1.2) |
| 1.480403 | 1 | 0.556 | 2.07E-173 | 12 | Col1a2 | Myofibroblast (Myo 1.2) |
| 1.032876 | 0.948 | 0.387 | 4.93E-173 | 12 | Aebp1 | Myofibroblast (Myo 1.2) |
| 1.463518 | 0.98 | 0.446 | 1.30E-169 | 12 | Postn | Myofibroblast (Myo 1.2) |
| 0.733932 | 0.838 | 0.258 | 1.17E-162 | 12 | Col11a1 | Myofibroblast (Myo 1.2) |
| 1.021749 | 0.998 | 0.485 | 1.04E-160 | 12 | Col5a2 | Myofibroblast (Myo 1.2) |
| 0.895627 | 0.97 | 0.406 | 4.20E-157 | 12 | Col5a1 | Myofibroblast (Myo 1.2) |
| 1.306421 | 1 | 0.547 | 1.99E-155 | 12 | Col3a1 | Myofibroblast (Myo 1.2) |
| 0.992257 | 0.97 | 0.461 | 2.50E-146 | 12 | Col6a1 | Myofibroblast (Myo 1.2) |
| 0.805667 | 0.926 | 0.363 | 4.51E-145 | 12 | Mmp2 | Myofibroblast (Myo 1.2) |
| 1.245473 | 1 | 0.581 | 8.27E-145 | 12 | Sparc | Myofibroblast (Myo 1.2) |
| 0.986003 | 0.944 | 0.431 | 6.70E-144 | 12 | Col6a3 | Myofibroblast (Myo 1.2) |
| 0.803009 | 0.766 | 0.258 | 2.91E-143 | 12 | Thbs2 | Myofibroblast (Myo 1.2) |
| 0.542865 | 0.614 | 0.168 | 9.67E-139 | 12 | Loxl2 | Myofibroblast (Myo 1.2) |
| 0.812179 | 0.76 | 0.266 | 6.15E-138 | 12 | Htra1 | Myofibroblast (Myo 1.2) |
| 0.600122 | 0.75 | 0.244 | 1.57E-137 | 12 | Cdh11 | Myofibroblast (Myo 1.2) |
| 0.771385 | 0.834 | 0.357 | 3.74E-125 | 12 | Mmp14 | Myofibroblast (Myo 1.2) |
| 0.834896 | 0.93 | 0.424 | 6.17E-125 | 12 | Col6a2 | Myofibroblast (Myo 1.2) |
| 0.882577 | 0.976 | 0.492 | 5.89E-124 | 12 | Serpinh1 | Myofibroblast (Myo 1.2) |
| 0.501845 | 0.632 | 0.191 | 1.44E-121 | 12 | Fgfr1 | Myofibroblast (Myo 1.2) |
| 0.616197 | 0.67 | 0.213 | 5.42E-117 | 12 | Cthrc1 | Myofibroblast (Myo 1.2) |
| 0.525732 | 0.694 | 0.238 | 4.16E-114 | 12 | Mxra8 | Myofibroblast (Myo 1.2) |
| 0.690065 | 0.826 | 0.333 | 2.12E-113 | 12 | Aspn | Myofibroblast (Myo 1.2) |
| 0.409557 | 0.406 | 0.091 | 4.55E-111 | 12 | Gja1 | Myofibroblast (Myo 1.2) |
| 0.261685 | 0.194 | 0.023 | 2.58E-109 | 12 | Alpl | Myofibroblast (Myo 1.2) |
| 0.777895 | 0.89 | 0.435 | 1.44E-107 | 12 | Pmepa1 | Myofibroblast (Myo 1.2) |
| 0.734235 | 0.91 | 0.506 | 5.25E-101 | 12 | Timp2 | Myofibroblast (Myo 1.2) |
| 0.619806 | 0.926 | 0.41 | 5.82E-99 | 12 | Serpinf1 | Myofibroblast (Myo 1.2) |
| 0.730186 | 0.972 | 0.644 | 3.87E-97 | 12 | Cd63 | Myofibroblast (Myo 1.2) |
| 0.387985 | 0.412 | 0.105 | 3.11E-95 | 12 | Ccn4 | Myofibroblast (Myo 1.2) |
| 0.752246 | 0.972 | 0.541 | 4.56E-95 | 12 | Fn1 | Myofibroblast (Myo 1.2) |
| 0.549369 | 0.802 | 0.347 | 7.78E-95 | 12 | Pcolce | Myofibroblast (Myo 1.2) |
| 0.431426 | 0.514 | 0.153 | 1.89E-94 | 12 | Sdc1 | Myofibroblast (Myo 1.2) |
| 0.291637 | 0.248 | 0.042 | 1.33E-93 | 12 | Ptprd | Myofibroblast (Myo 1.2) |
| 0.403796 | 0.952 | 0.409 | 1.29E-89 | 12 | Dcn | Myofibroblast (Myo 1.2) |
| 0.545427 | 0.848 | 0.401 | 5.96E-89 | 12 | Ppic | Myofibroblast (Myo 1.2) |
| 0.454187 | 0.6 | 0.212 | 1.33E-88 | 12 | Olfml3 | Myofibroblast (Myo 1.2) |
| 0.368128 | 0.408 | 0.109 | 4.35E-88 | 12 | Scarf2 | Myofibroblast (Myo 1.2) |
| 0.53185 | 0.502 | 0.155 | 2.22E-87 | 12 | Ogn | Myofibroblast (Myo 1.2) |
| 0.856889 | 0.454 | 0.134 | 1.84E-85 | 12 | Cxcl5 | Myofibroblast (Myo 1.2) |
| 0.398644 | 0.514 | 0.166 | 9.14E-83 | 12 | Pdgfra | Myofibroblast (Myo 1.2) |
| 0.469662 | 0.77 | 0.334 | 2.14E-81 | 12 | Rcn3 | Myofibroblast (Myo 1.2) |
| 0.660285 | 0.77 | 0.361 | 3.21E-81 | 12 | Nupr1 | Myofibroblast (Myo 1.2) |
| 0.505842 | 0.896 | 0.415 | 1.32E-80 | 12 | Igfbp4 | Myofibroblast (Myo 1.2) |
| 0.546995 | 0.492 | 0.162 | 1.09E-78 | 12 | Itm2a | Myofibroblast (Myo 1.2) |
| 0.696149 | 0.996 | 0.519 | 1.40E-77 | 12 | Bgn | Myofibroblast (Myo 1.2) |
| 0.33032 | 0.8 | 0.348 | 1.75E-76 | 12 | Ctsk | Myofibroblast (Myo 1.2) |
| 0.341298 | 0.306 | 0.072 | 1.77E-76 | 12 | Rflnb | Myofibroblast (Myo 1.2) |
| 0.426522 | 0.316 | 0.076 | 2.70E-76 | 12 | Il1rl1 | Myofibroblast (Myo 1.2) |
| 0.560017 | 0.492 | 0.162 | 3.41E-76 | 12 | Crabp1 | Myofibroblast (Myo 1.2) |
| 0.394162 | 0.498 | 0.168 | 4.77E-76 | 12 | Wls | Myofibroblast (Myo 1.2) |
| 0.444142 | 0.67 | 0.269 | 6.15E-76 | 12 | Lox | Myofibroblast (Myo 1.2) |
| 0.285999 | 0.278 | 0.061 | 1.07E-75 | 12 | Ncam1 | Myofibroblast (Myo 1.2) |
| 0.401616 | 0.532 | 0.192 | 1.39E-73 | 12 | Olfml2b | Myofibroblast (Myo 1.2) |
| 0.402452 | 0.432 | 0.131 | 1.71E-73 | 12 | Sfrp2 | Myofibroblast (Myo 1.2) |
| 0.507507 | 0.906 | 0.458 | 2.90E-72 | 12 | Cald1 | Myofibroblast (Myo 1.2) |
| 0.512504 | 0.85 | 0.46 | 1.76E-69 | 12 | Rrbp1 | Myofibroblast (Myo 1.2) |
| 0.414252 | 0.472 | 0.157 | 4.82E-69 | 12 | Tnn | Myofibroblast (Myo 1.2) |
| 0.431812 | 0.798 | 0.371 | 6.52E-68 | 12 | Tpm1 | Myofibroblast (Myo 1.2) |
| 0.490984 | 0.58 | 0.236 | 5.09E-67 | 12 | Tsc22d1 | Myofibroblast (Myo 1.2) |
| 0.359343 | 0.456 | 0.155 | 7.36E-66 | 12 | Col16a1 | Myofibroblast (Myo 1.2) |
| 0.284521 | 0.668 | 0.274 | 2.44E-65 | 12 | Tpm2 | Myofibroblast (Myo 1.2) |
| 0.476373 | 0.928 | 0.605 | 3.61E-64 | 12 | Ppib | Myofibroblast (Myo 1.2) |
| 0.435163 | 0.676 | 0.313 | 4.01E-62 | 12 | Kdelr2 | Myofibroblast (Myo 1.2) |
| 0.506782 | 0.73 | 0.349 | 5.39E-61 | 12 | Timp1 | Myofibroblast (Myo 1.2) |
| 0.435121 | 0.754 | 0.375 | 1.72E-60 | 12 | Selenom | Myofibroblast (Myo 1.2) |
| 0.3965 | 0.672 | 0.313 | 2.88E-60 | 12 | Tceal9 | Myofibroblast (Myo 1.2) |
| 0.65864 | 0.984 | 0.727 | 8.91E-59 | 12 | Tmsb10 | Myofibroblast (Myo 1.2) |
| 0.432923 | 0.868 | 0.427 | 4.86E-58 | 12 | Fstl1 | Myofibroblast (Myo 1.2) |
| 0.381121 | 0.444 | 0.161 | 3.11E-57 | 12 | Nrep | Myofibroblast (Myo 1.2) |
| 0.309339 | 0.314 | 0.09 | 4.60E-56 | 12 | Omd | Myofibroblast (Myo 1.2) |
| 0.27379 | 0.248 | 0.062 | 3.64E-55 | 12 | Htra3 | Myofibroblast (Myo 1.2) |
| 0.387064 | 0.774 | 0.395 | 4.07E-55 | 12 | Nedd4 | Myofibroblast (Myo 1.2) |
| 0.331458 | 0.522 | 0.211 | 1.32E-54 | 12 | Cnn3 | Myofibroblast (Myo 1.2) |
| 0.324346 | 0.468 | 0.179 | 1.67E-54 | 12 | Maged1 | Myofibroblast (Myo 1.2) |
| 0.671749 | 0.422 | 0.155 | 2.05E-54 | 12 | Igfbp5 | Myofibroblast (Myo 1.2) |
| 0.303586 | 0.374 | 0.126 | 5.13E-53 | 12 | Antxr1 | Myofibroblast (Myo 1.2) |
| 0.364052 | 0.568 | 0.25 | 3.00E-52 | 12 | Ckap4 | Myofibroblast (Myo 1.2) |
| 0.354524 | 0.476 | 0.183 | 1.50E-51 | 12 | Cpxm1 | Myofibroblast (Myo 1.2) |
| 0.338221 | 0.436 | 0.165 | 3.09E-51 | 12 | Bmp1 | Myofibroblast (Myo 1.2) |
| 0.344757 | 0.568 | 0.252 | 2.41E-50 | 12 | Calu | Myofibroblast (Myo 1.2) |
| 0.358255 | 0.704 | 0.339 | 5.13E-50 | 12 | Fbn1 | Myofibroblast (Myo 1.2) |
| 0.318899 | 0.424 | 0.159 | 5.14E-50 | 12 | C1qtnf6 | Myofibroblast (Myo 1.2) |
| 0.26154 | 0.286 | 0.083 | 1.28E-49 | 12 | Spon1 | Myofibroblast (Myo 1.2) |
| 0.355272 | 0.79 | 0.429 | 3.80E-49 | 12 | Slc25a4 | Myofibroblast (Myo 1.2) |
| 0.39773 | 0.692 | 0.358 | 4.10E-49 | 12 | Lrp1 | Myofibroblast (Myo 1.2) |
| 0.300495 | 0.416 | 0.156 | 1.31E-48 | 12 | Txndc5 | Myofibroblast (Myo 1.2) |
| 0.407743 | 0.38 | 0.135 | 3.10E-48 | 12 | Fmod | Myofibroblast (Myo 1.2) |
| 0.316453 | 0.456 | 0.183 | 1.93E-47 | 12 | Itgb5 | Myofibroblast (Myo 1.2) |
| 0.29019 | 0.416 | 0.156 | 5.14E-47 | 12 | Palld | Myofibroblast (Myo 1.2) |
| 0.325239 | 0.366 | 0.131 | 9.87E-47 | 12 | Maf | Myofibroblast (Myo 1.2) |
| 0.312948 | 0.44 | 0.176 | 2.53E-46 | 12 | Lman1 | Myofibroblast (Myo 1.2) |
| 0.347061 | 0.594 | 0.287 | 4.84E-45 | 12 | Ostc | Myofibroblast (Myo 1.2) |
| 0.311655 | 0.462 | 0.197 | 2.13E-43 | 12 | Arl1 | Myofibroblast (Myo 1.2) |
| 0.401163 | 0.496 | 0.22 | 5.69E-43 | 12 | Col5a3 | Myofibroblast (Myo 1.2) |
| 0.379078 | 0.662 | 0.329 | 3.17E-42 | 12 | Csrp2 | Myofibroblast (Myo 1.2) |
| 0.335013 | 0.714 | 0.388 | 1.97E-41 | 12 | P4hb | Myofibroblast (Myo 1.2) |
| 0.313098 | 0.642 | 0.325 | 7.92E-40 | 12 | Prrx1 | Myofibroblast (Myo 1.2) |
| 0.494821 | 0.972 | 0.733 | 9.99E-40 | 12 | Lgals1 | Myofibroblast (Myo 1.2) |
| 0.349095 | 0.82 | 0.497 | 1.15E-39 | 12 | Laptm4a | Myofibroblast (Myo 1.2) |
| 0.27519 | 0.464 | 0.199 | 6.42E-39 | 12 | Mfge8 | Myofibroblast (Myo 1.2) |
| 0.264595 | 0.332 | 0.124 | 5.33E-38 | 12 | Kdelr3 | Myofibroblast (Myo 1.2) |
| 0.411805 | 0.878 | 0.611 | 6.89E-38 | 12 | Sec61g | Myofibroblast (Myo 1.2) |
| 0.398183 | 0.78 | 0.498 | 1.53E-37 | 12 | Sec61b | Myofibroblast (Myo 1.2) |
| 0.294168 | 0.562 | 0.277 | 1.53E-37 | 12 | Hdlbp | Myofibroblast (Myo 1.2) |
| 0.293933 | 0.534 | 0.257 | 1.71E-37 | 12 | Ssr2 | Myofibroblast (Myo 1.2) |
| 0.283305 | 0.43 | 0.196 | 5.30E-33 | 12 | Cnn2 | Myofibroblast (Myo 1.2) |
| 0.266953 | 0.486 | 0.228 | 7.62E-33 | 12 | Nbl1 | Myofibroblast (Myo 1.2) |
| 0.353569 | 0.46 | 0.216 | 8.46E-33 | 12 | Sgk1 | Myofibroblast (Myo 1.2) |
| 0.25923 | 0.444 | 0.205 | 1.45E-32 | 12 | Tmed3 | Myofibroblast (Myo 1.2) |
| 0.388453 | 0.498 | 0.25 | 3.86E-32 | 12 | Ecm1 | Myofibroblast (Myo 1.2) |
| 0.3685 | 0.928 | 0.708 | 4.76E-32 | 12 | Nme2 | Myofibroblast (Myo 1.2) |
| 0.404562 | 0.338 | 0.142 | 8.62E-32 | 12 | Inhba | Myofibroblast (Myo 1.2) |
| 0.275111 | 0.704 | 0.41 | 1.75E-31 | 12 | Arf4 | Myofibroblast (Myo 1.2) |
| 0.284055 | 0.3 | 0.116 | 3.78E-31 | 12 | Wnt5a | Myofibroblast (Myo 1.2) |
| 0.303386 | 0.914 | 0.628 | 2.09E-30 | 12 | Gnas | Myofibroblast (Myo 1.2) |
| 0.27447 | 0.398 | 0.181 | 3.30E-29 | 12 | Gas1 | Myofibroblast (Myo 1.2) |
| 0.260819 | 0.506 | 0.256 | 5.41E-29 | 12 | Tcf4 | Myofibroblast (Myo 1.2) |
| 0.342389 | 0.486 | 0.243 | 1.31E-28 | 12 | Errfi1 | Myofibroblast (Myo 1.2) |
| 0.276615 | 0.708 | 0.433 | 3.27E-28 | 12 | Bsg | Myofibroblast (Myo 1.2) |
| 0.294725 | 0.68 | 0.407 | 1.32E-27 | 12 | Itgb1 | Myofibroblast (Myo 1.2) |
| 0.329149 | 0.948 | 0.836 | 8.91E-27 | 12 | Serf2 | Myofibroblast (Myo 1.2) |
| 0.26416 | 0.748 | 0.484 | 3.05E-26 | 12 | Arf5 | Myofibroblast (Myo 1.2) |
| 0.314539 | 0.996 | 0.943 | 8.12E-26 | 12 | Rpl41 | Myofibroblast (Myo 1.2) |
| 0.281979 | 0.2 | 0.067 | 9.56E-26 | 12 | Col7a1 | Myofibroblast (Myo 1.2) |
| 0.285665 | 0.76 | 0.52 | 5.76E-22 | 12 | Hsp90b1 | Myofibroblast (Myo 1.2) |
| 0.264822 | 0.408 | 0.204 | 1.48E-21 | 12 | Rbp1 | Myofibroblast (Myo 1.2) |
| 0.256775 | 0.812 | 0.564 | 4.89E-20 | 12 | Rpl22l1 | Myofibroblast (Myo 1.2) |
| 0.258516 | 0.984 | 0.87 | 1.72E-18 | 12 | Rps20 | Myofibroblast (Myo 1.2) |
| 0.269019 | 0.676 | 0.45 | 5.81E-16 | 12 | Fosb | Myofibroblast (Myo 1.2) |
| 0.269958 | 0.696 | 0.492 | 9.30E-16 | 12 | Hmgb1 | Myofibroblast (Myo 1.2) |
| 0.302973 | 0.622 | 0.406 | 2.24E-14 | 12 | Jun | Myofibroblast (Myo 1.2) |
| 0.309189 | 0.786 | 0.631 | 5.04E-13 | 12 | Ybx1 | Myofibroblast (Myo 1.2) |
| 0.293384 | 0.43 | 0.296 | 2.30E-06 | 12 | Fabp5 | Myofibroblast (Myo 1.2) |
| 3.505164 | 1 | 0.159 | 0 | 13 | Cd74 | Dendritic_Cells |
| 3.396167 | 0.994 | 0.123 | 0 | 13 | H2-Aa | Dendritic_Cells |
| 3.345907 | 0.976 | 0.122 | 0 | 13 | H2-Ab1 | Dendritic_Cells |
| 1.978176 | 0.509 | 0.053 | 0 | 13 | H2-Eb1 | Dendritic_Cells |
| 1.795236 | 0.906 | 0.18 | 0 | 13 | Ifi30 | Dendritic_Cells |
| 1.049829 | 0.748 | 0.076 | 0 | 13 | Syngr2 | Dendritic_Cells |
| 1.035967 | 0.782 | 0.145 | 0 | 13 | Cytip | Dendritic_Cells |
| 1.013505 | 0.722 | 0.126 | 0 | 13 | Psmb8 | Dendritic_Cells |
| 0.855175 | 0.603 | 0.033 | 0 | 13 | H2-DMb1 | Dendritic_Cells |
| 0.84753 | 0.62 | 0.065 | 0 | 13 | Napsa | Dendritic_Cells |
| 0.796209 | 0.643 | 0.053 | 0 | 13 | H2-DMa | Dendritic_Cells |
| 0.670702 | 0.519 | 0.05 | 0 | 13 | Plbd1 | Dendritic_Cells |
| 0.584618 | 0.282 | 0.008 | 0 | 13 | Clec10a | Dendritic_Cells |
| 0.582401 | 0.244 | 0.002 | 0 | 13 | Il4i1 | Dendritic_Cells |
| 0.511972 | 0.229 | 0.006 | 0 | 13 | Tbc1d4 | Dendritic_Cells |
| 0.481531 | 0.28 | 0.001 | 0 | 13 | Timd4 | Dendritic_Cells |
| 0.480749 | 0.205 | 0.002 | 0 | 13 | Cd209a | Dendritic_Cells |
| 0.417243 | 0.235 | 0.004 | 0 | 13 | Klrd1 | Dendritic_Cells |
| 0.588148 | 0.31 | 0.021 | 6.17E-281 | 13 | Tnip3 | Dendritic_Cells |
| 0.953235 | 0.474 | 0.055 | 1.64E-279 | 13 | Gpr183 | Dendritic_Cells |
| 0.902561 | 0.66 | 0.11 | 1.61E-273 | 13 | Cd83 | Dendritic_Cells |
| 0.372459 | 0.316 | 0.023 | 4.56E-266 | 13 | H2-DMb2 | Dendritic_Cells |
| 0.509722 | 0.335 | 0.03 | 3.16E-243 | 13 | Ramp3 | Dendritic_Cells |
| 0.551669 | 0.376 | 0.042 | 2.43E-225 | 13 | Avpi1 | Dendritic_Cells |
| 0.865554 | 0.874 | 0.206 | 2.00E-222 | 13 | Ctss | Dendritic_Cells |
| 0.750911 | 0.605 | 0.112 | 4.02E-219 | 13 | Traf1 | Dendritic_Cells |
| 1.896689 | 1 | 0.899 | 2.97E-218 | 13 | Tmsb4x | Dendritic_Cells |
| 0.71321 | 0.618 | 0.124 | 8.35E-214 | 13 | Gng10 | Dendritic_Cells |
| 0.477102 | 0.391 | 0.05 | 6.14E-203 | 13 | Cd86 | Dendritic_Cells |
| 0.623599 | 0.547 | 0.102 | 3.55E-198 | 13 | H2afy | Dendritic_Cells |
| 1.048713 | 0.34 | 0.041 | 7.79E-190 | 13 | Ccr7 | Dendritic_Cells |
| 0.772441 | 0.543 | 0.105 | 3.84E-189 | 13 | Cxcl16 | Dendritic_Cells |
| 0.690089 | 0.618 | 0.135 | 1.37E-187 | 13 | Gm2a | Dendritic_Cells |
| 1.18327 | 0.906 | 0.419 | 1.36E-177 | 13 | Atox1 | Dendritic_Cells |
| 2.365521 | 0.632 | 0.166 | 8.03E-177 | 13 | Ifitm1 | Dendritic_Cells |
| 0.697244 | 0.594 | 0.135 | 4.64E-175 | 13 | Psme1 | Dendritic_Cells |
| 0.28277 | 0.194 | 0.014 | 5.79E-171 | 13 | Gpr171 | Dendritic_Cells |
| 0.849805 | 0.479 | 0.092 | 1.39E-160 | 13 | Rgs1 | Dendritic_Cells |
| 0.754568 | 0.803 | 0.247 | 2.12E-160 | 13 | Coro1a | Dendritic_Cells |
| 0.484292 | 0.412 | 0.069 | 7.34E-159 | 13 | Tnfaip8 | Dendritic_Cells |
| 0.414241 | 0.34 | 0.048 | 3.27E-156 | 13 | Runx3 | Dendritic_Cells |
| 1.145219 | 0.838 | 0.293 | 2.19E-155 | 13 | Cd52 | Dendritic_Cells |
| 1.566146 | 0.919 | 0.51 | 4.57E-155 | 13 | H2afz | Dendritic_Cells |
| 0.418367 | 0.308 | 0.04 | 6.52E-154 | 13 | Etv3 | Dendritic_Cells |
| 0.93143 | 0.761 | 0.256 | 7.61E-154 | 13 | Lsp1 | Dendritic_Cells |
| 0.710336 | 0.581 | 0.142 | 3.49E-152 | 13 | Rel | Dendritic_Cells |
| 0.954238 | 0.844 | 0.351 | 1.91E-148 | 13 | Sub1 | Dendritic_Cells |
| 0.414049 | 0.291 | 0.037 | 1.44E-146 | 13 | Bcl2a1a | Dendritic_Cells |
| 0.740256 | 0.795 | 0.27 | 2.28E-142 | 13 | Ucp2 | Dendritic_Cells |
| 1.284846 | 0.998 | 0.959 | 4.51E-141 | 13 | Actb | Dendritic_Cells |
| 2.262199 | 0.929 | 0.643 | 2.73E-137 | 13 | Cst3 | Dendritic_Cells |
| 0.345909 | 0.271 | 0.035 | 7.92E-135 | 13 | Psmb9 | Dendritic_Cells |
| 0.405551 | 0.182 | 0.016 | 2.27E-133 | 13 | Naaa | Dendritic_Cells |
| 0.452638 | 0.244 | 0.03 | 6.94E-130 | 13 | Irf8 | Dendritic_Cells |
| 0.574631 | 0.498 | 0.115 | 4.20E-129 | 13 | Csf2rb | Dendritic_Cells |
| 1.093356 | 0.994 | 0.645 | 7.20E-127 | 13 | H2-D1 | Dendritic_Cells |
| 0.37124 | 0.28 | 0.04 | 5.59E-126 | 13 | Lactb | Dendritic_Cells |
| 0.538442 | 0.816 | 0.24 | 3.76E-123 | 13 | Il1b | Dendritic_Cells |
| 0.452612 | 0.382 | 0.074 | 5.55E-123 | 13 | Cfp | Dendritic_Cells |
| 0.814572 | 0.878 | 0.39 | 3.31E-119 | 13 | Cyba | Dendritic_Cells |
| 0.477869 | 0.451 | 0.103 | 5.28E-119 | 13 | Ctsh | Dendritic_Cells |
| 0.673101 | 0.917 | 0.314 | 5.13E-117 | 13 | Tyrobp | Dendritic_Cells |
| 0.286726 | 0.216 | 0.026 | 1.74E-114 | 13 | Plscr1 | Dendritic_Cells |
| 0.732614 | 0.998 | 0.969 | 6.60E-113 | 13 | Fau | Dendritic_Cells |
| 0.804064 | 0.823 | 0.395 | 8.56E-113 | 13 | Tspo | Dendritic_Cells |
| 0.979753 | 0.65 | 0.234 | 3.91E-112 | 13 | Basp1 | Dendritic_Cells |
| 0.821991 | 0.976 | 0.789 | 7.15E-112 | 13 | Pfn1 | Dendritic_Cells |
| 0.807 | 0.861 | 0.466 | 7.17E-112 | 13 | Sh3bgrl3 | Dendritic_Cells |
| 0.613803 | 0.735 | 0.261 | 1.78E-110 | 13 | Laptm5 | Dendritic_Cells |
| 0.453776 | 0.485 | 0.125 | 8.95E-107 | 13 | Fam49b | Dendritic_Cells |
| 0.317411 | 0.216 | 0.028 | 6.20E-106 | 13 | Grasp | Dendritic_Cells |
| 0.530581 | 0.524 | 0.138 | 4.41E-105 | 13 | Wfdc17 | Dendritic_Cells |
| 1.073563 | 0.94 | 0.726 | 1.55E-104 | 13 | Calm1 | Dendritic_Cells |
| 0.516225 | 0.44 | 0.111 | 6.64E-104 | 13 | Psme2 | Dendritic_Cells |
| 0.56773 | 1 | 0.973 | 1.68E-98 | 13 | Rps29 | Dendritic_Cells |
| 0.571444 | 0.656 | 0.222 | 1.24E-95 | 13 | Alox5ap | Dendritic_Cells |
| 0.410596 | 0.402 | 0.098 | 4.72E-91 | 13 | Selplg | Dendritic_Cells |
| 0.367927 | 0.316 | 0.065 | 4.45E-90 | 13 | Ass1 | Dendritic_Cells |
| 0.484978 | 0.491 | 0.15 | 4.68E-88 | 13 | Ywhah | Dendritic_Cells |
| 0.428214 | 0.588 | 0.19 | 8.71E-87 | 13 | Spi1 | Dendritic_Cells |
| 0.71715 | 0.983 | 0.859 | 3.20E-86 | 13 | Rps11 | Dendritic_Cells |
| 0.604843 | 0.697 | 0.309 | 9.10E-85 | 13 | Cd47 | Dendritic_Cells |
| 0.588583 | 0.9 | 0.537 | 2.93E-84 | 13 | Arpc2 | Dendritic_Cells |
| 0.802545 | 0.78 | 0.385 | 7.46E-84 | 13 | Fxyd5 | Dendritic_Cells |
| 0.54587 | 0.994 | 0.949 | 1.94E-79 | 13 | Rps9 | Dendritic_Cells |
| 0.482209 | 0.449 | 0.129 | 6.05E-79 | 13 | Ms4a6c | Dendritic_Cells |
| 0.429907 | 0.357 | 0.09 | 9.39E-79 | 13 | Ckb | Dendritic_Cells |
| 0.256858 | 0.216 | 0.036 | 2.77E-78 | 13 | Ikbkb | Dendritic_Cells |
| 0.302529 | 0.218 | 0.037 | 7.95E-77 | 13 | Stap1 | Dendritic_Cells |
| 0.568254 | 0.799 | 0.306 | 4.23E-76 | 13 | Fcer1g | Dendritic_Cells |
| 0.688102 | 0.878 | 0.519 | 5.66E-73 | 13 | H2-K1 | Dendritic_Cells |
| 0.527588 | 0.874 | 0.515 | 6.15E-73 | 13 | Arpc1b | Dendritic_Cells |
| 0.567205 | 0.709 | 0.327 | 4.29E-72 | 13 | Ctsz | Dendritic_Cells |
| 0.334543 | 0.226 | 0.042 | 4.39E-72 | 13 | Ms4a4c | Dendritic_Cells |
| 0.862362 | 0.739 | 0.439 | 7.82E-71 | 13 | Rps27l | Dendritic_Cells |
| 0.60908 | 0.959 | 0.861 | 1.20E-69 | 13 | H3f3a | Dendritic_Cells |
| 0.446039 | 0.609 | 0.238 | 1.73E-69 | 13 | Arhgdib | Dendritic_Cells |
| 0.328099 | 0.338 | 0.087 | 3.81E-69 | 13 | Tspan13 | Dendritic_Cells |
| 0.411707 | 0.506 | 0.182 | 1.03E-65 | 13 | Pitpna | Dendritic_Cells |
| 0.991651 | 0.893 | 0.73 | 5.54E-65 | 13 | Ifitm2 | Dendritic_Cells |
| 0.515931 | 0.917 | 0.686 | 2.01E-64 | 13 | Cfl1 | Dendritic_Cells |
| 0.358393 | 0.406 | 0.126 | 1.42E-63 | 13 | Grb2 | Dendritic_Cells |
| 0.467721 | 0.844 | 0.463 | 3.47E-63 | 13 | Arpc3 | Dendritic_Cells |
| 0.416023 | 0.35 | 0.1 | 6.80E-63 | 13 | Malt1 | Dendritic_Cells |
| 0.335929 | 0.686 | 0.27 | 4.00E-61 | 13 | Lcp1 | Dendritic_Cells |
| 0.410116 | 0.656 | 0.286 | 1.22E-60 | 13 | Arpc5 | Dendritic_Cells |
| 0.514037 | 0.799 | 0.446 | 1.90E-60 | 13 | Gpx1 | Dendritic_Cells |
| 0.396836 | 0.526 | 0.195 | 1.92E-60 | 13 | Efhd2 | Dendritic_Cells |
| 0.342786 | 0.323 | 0.089 | 5.11E-60 | 13 | Aif1 | Dendritic_Cells |
| 0.465344 | 0.252 | 0.059 | 9.75E-60 | 13 | Tnfsf9 | Dendritic_Cells |
| 0.423044 | 0.62 | 0.272 | 3.40E-59 | 13 | Tpm3 | Dendritic_Cells |
| 0.499227 | 0.923 | 0.751 | 1.34E-58 | 13 | Rps25 | Dendritic_Cells |
| 0.540746 | 0.979 | 0.892 | 2.78E-58 | 13 | Rpl35a | Dendritic_Cells |
| 0.409227 | 0.436 | 0.158 | 5.25E-56 | 13 | Rnh1 | Dendritic_Cells |
| 0.586202 | 0.968 | 0.863 | 1.08E-55 | 13 | Actg1 | Dendritic_Cells |
| 0.837256 | 0.806 | 0.533 | 3.39E-55 | 13 | S100a4 | Dendritic_Cells |
| 0.358642 | 0.558 | 0.227 | 3.35E-54 | 13 | Myl12b | Dendritic_Cells |
| 0.607931 | 0.462 | 0.191 | 2.98E-53 | 13 | Tmem176a | Dendritic_Cells |
| 0.523592 | 0.91 | 0.724 | 3.78E-53 | 13 | S100a11 | Dendritic_Cells |
| 0.380495 | 0.425 | 0.153 | 1.28E-52 | 13 | Arl4c | Dendritic_Cells |
| 0.471478 | 0.778 | 0.465 | 1.42E-52 | 13 | Clic1 | Dendritic_Cells |
| 0.415828 | 0.985 | 0.909 | 9.01E-52 | 13 | Rps16 | Dendritic_Cells |
| 0.304026 | 0.357 | 0.11 | 2.90E-50 | 13 | Il1r2 | Dendritic_Cells |
| 0.433577 | 0.953 | 0.837 | 3.73E-50 | 13 | Rpl26 | Dendritic_Cells |
| 0.41911 | 0.746 | 0.384 | 1.21E-49 | 13 | Lgals3 | Dendritic_Cells |
| 0.432446 | 0.489 | 0.203 | 8.90E-49 | 13 | Bhlhe40 | Dendritic_Cells |
| 0.46786 | 0.549 | 0.259 | 3.59E-48 | 13 | Gdi2 | Dendritic_Cells |
| 0.285643 | 0.299 | 0.09 | 3.69E-48 | 13 | Rala | Dendritic_Cells |
| 0.376015 | 0.577 | 0.261 | 5.22E-48 | 13 | Actr3 | Dendritic_Cells |
| 0.427504 | 0.831 | 0.573 | 4.32E-47 | 13 | Gnai2 | Dendritic_Cells |
| 0.42998 | 0.716 | 0.41 | 4.98E-47 | 13 | Ndufb1-ps | Dendritic_Cells |
| 0.357852 | 0.423 | 0.163 | 5.57E-47 | 13 | Mpc1 | Dendritic_Cells |
| 0.319389 | 0.541 | 0.227 | 6.18E-47 | 13 | Taldo1 | Dendritic_Cells |
| 0.267906 | 0.22 | 0.054 | 1.06E-45 | 13 | Il7r | Dendritic_Cells |
| 0.322166 | 0.397 | 0.144 | 1.24E-45 | 13 | Ifngr1 | Dendritic_Cells |
| 0.367221 | 0.97 | 0.849 | 4.11E-45 | 13 | Rps13 | Dendritic_Cells |
| 0.368931 | 0.346 | 0.12 | 2.78E-44 | 13 | Dusp5 | Dendritic_Cells |
| 0.269608 | 0.274 | 0.081 | 5.05E-44 | 13 | Got1 | Dendritic_Cells |
| 0.496025 | 0.474 | 0.198 | 8.66E-44 | 13 | Id2 | Dendritic_Cells |
| 0.418629 | 0.994 | 0.912 | 1.40E-43 | 13 | Rps27a | Dendritic_Cells |
| 0.45106 | 0.964 | 0.837 | 2.15E-43 | 13 | Rps26 | Dendritic_Cells |
| 0.477448 | 0.271 | 0.085 | 2.37E-43 | 13 | Tmem123 | Dendritic_Cells |
| 0.467839 | 0.598 | 0.302 | 1.68E-42 | 13 | Ly6e | Dendritic_Cells |
| 0.300834 | 0.517 | 0.214 | 1.72E-42 | 13 | Cd53 | Dendritic_Cells |
| 0.353571 | 0.481 | 0.211 | 8.83E-42 | 13 | Pomp | Dendritic_Cells |
| 0.312349 | 0.754 | 0.38 | 1.13E-41 | 13 | Pim1 | Dendritic_Cells |
| 0.535304 | 0.487 | 0.238 | 1.44E-41 | 13 | Tmem176b | Dendritic_Cells |
| 0.521362 | 0.923 | 0.654 | 3.35E-41 | 13 | B2m | Dendritic_Cells |
| 0.82454 | 0.925 | 0.769 | 3.79E-41 | 13 | Crip1 | Dendritic_Cells |
| 0.477773 | 0.453 | 0.205 | 7.26E-41 | 13 | Map4k4 | Dendritic_Cells |
| 0.368579 | 0.806 | 0.526 | 3.80E-40 | 13 | Cdc42 | Dendritic_Cells |
| 0.357908 | 0.994 | 0.901 | 5.54E-40 | 13 | Rps24 | Dendritic_Cells |
| 0.269202 | 0.229 | 0.064 | 5.91E-40 | 13 | H2-Q7 | Dendritic_Cells |
| 0.329793 | 0.402 | 0.15 | 6.64E-40 | 13 | Samsn1 | Dendritic_Cells |
| 0.442497 | 0.793 | 0.49 | 8.78E-40 | 13 | Atp6v0c | Dendritic_Cells |
| 0.399588 | 0.346 | 0.128 | 1.14E-39 | 13 | Nr4a3 | Dendritic_Cells |
| 0.364045 | 0.632 | 0.326 | 1.29E-39 | 13 | Msn | Dendritic_Cells |
| 0.35906 | 0.949 | 0.795 | 8.33E-38 | 13 | Rpl27 | Dendritic_Cells |
| 0.385711 | 1 | 0.908 | 4.07E-37 | 13 | Rps28 | Dendritic_Cells |
| 0.30261 | 0.425 | 0.18 | 1.10E-36 | 13 | Sri | Dendritic_Cells |
| 0.400434 | 0.985 | 0.844 | 4.10E-36 | 13 | Rps19 | Dendritic_Cells |
| 0.297149 | 0.464 | 0.216 | 1.67E-33 | 13 | Erp29 | Dendritic_Cells |
| 0.259194 | 0.16 | 0.039 | 3.00E-33 | 13 | AA467197 | Dendritic_Cells |
| 0.326994 | 0.944 | 0.839 | 6.45E-33 | 13 | Rpl17 | Dendritic_Cells |
| 0.482354 | 0.752 | 0.523 | 3.30E-32 | 13 | Tagln2 | Dendritic_Cells |
| 0.262493 | 0.259 | 0.088 | 3.51E-32 | 13 | M6pr | Dendritic_Cells |
| 0.330657 | 0.887 | 0.709 | 4.30E-32 | 13 | Rps6 | Dendritic_Cells |
| 0.340008 | 0.962 | 0.854 | 1.99E-31 | 13 | Rpl18 | Dendritic_Cells |
| 0.354422 | 0.575 | 0.294 | 2.23E-31 | 13 | Cdkn1a | Dendritic_Cells |
| 0.31608 | 0.637 | 0.358 | 2.93E-31 | 13 | Ubl5 | Dendritic_Cells |
| 0.362188 | 0.583 | 0.344 | 5.94E-30 | 13 | Mrpl52 | Dendritic_Cells |
| 0.272561 | 0.391 | 0.172 | 7.00E-30 | 13 | Ndufa6 | Dendritic_Cells |
| 0.439617 | 0.707 | 0.502 | 9.71E-30 | 13 | Uba52 | Dendritic_Cells |
| 0.260442 | 0.991 | 0.943 | 3.24E-29 | 13 | Rps27 | Dendritic_Cells |
| 0.430517 | 0.459 | 0.234 | 1.06E-28 | 13 | Zfp36l2 | Dendritic_Cells |
| 0.281325 | 0.408 | 0.185 | 1.21E-28 | 13 | Cotl1 | Dendritic_Cells |
| 0.311978 | 0.966 | 0.861 | 2.97E-28 | 13 | Rps23 | Dendritic_Cells |
| 0.389334 | 0.355 | 0.158 | 7.85E-26 | 13 | Bcl2a1b | Dendritic_Cells |
| 0.266789 | 0.387 | 0.181 | 8.29E-26 | 13 | Psmd8 | Dendritic_Cells |
| 0.335108 | 0.968 | 0.845 | 1.30E-25 | 13 | Rps4x | Dendritic_Cells |
| 0.285473 | 0.976 | 0.883 | 1.48E-25 | 13 | Rps10 | Dendritic_Cells |
| 0.284927 | 0.953 | 0.851 | 4.03E-25 | 13 | Rps14 | Dendritic_Cells |
| 0.283352 | 0.947 | 0.853 | 1.06E-24 | 13 | Rpl8 | Dendritic_Cells |
| 0.354148 | 0.972 | 0.857 | 1.19E-24 | 13 | Rpsa | Dendritic_Cells |
| 0.349077 | 0.248 | 0.094 | 1.57E-24 | 13 | Bst2 | Dendritic_Cells |
| 0.337107 | 0.97 | 0.871 | 1.96E-24 | 13 | Rpl34 | Dendritic_Cells |
| 0.27924 | 0.517 | 0.283 | 9.69E-24 | 13 | Atp6v0b | Dendritic_Cells |
| 0.463756 | 0.378 | 0.184 | 1.30E-23 | 13 | Plac8 | Dendritic_Cells |
| 0.327963 | 0.983 | 0.848 | 2.07E-23 | 13 | Rpl32 | Dendritic_Cells |
| 0.316963 | 0.808 | 0.619 | 2.17E-23 | 13 | Rbm3 | Dendritic_Cells |
| 0.285974 | 0.504 | 0.272 | 2.30E-23 | 13 | Picalm | Dendritic_Cells |
| 0.25747 | 0.278 | 0.114 | 2.39E-23 | 13 | Rilpl2 | Dendritic_Cells |
| 0.275577 | 0.994 | 0.896 | 2.99E-23 | 13 | Rpl13 | Dendritic_Cells |
| 0.328928 | 0.94 | 0.826 | 3.41E-23 | 13 | Rpl10 | Dendritic_Cells |
| 0.25983 | 0.953 | 0.839 | 5.82E-23 | 13 | Rps7 | Dendritic_Cells |
| 0.276181 | 0.545 | 0.315 | 1.10E-22 | 13 | Psma7 | Dendritic_Cells |
| 0.344091 | 0.959 | 0.861 | 1.85E-22 | 13 | Rpl28 | Dendritic_Cells |
| 0.273339 | 0.419 | 0.217 | 1.89E-22 | 13 | Smdt1 | Dendritic_Cells |
| 0.296798 | 0.964 | 0.841 | 4.53E-22 | 13 | Rplp2 | Dendritic_Cells |
| 0.274111 | 0.972 | 0.883 | 1.38E-21 | 13 | Rpl27a | Dendritic_Cells |
| 0.322269 | 0.771 | 0.565 | 4.02E-21 | 13 | Rpl36al | Dendritic_Cells |
| 0.34264 | 0.985 | 0.905 | 7.16E-21 | 13 | Rpl39 | Dendritic_Cells |
| 0.255321 | 0.451 | 0.244 | 1.35E-20 | 13 | Hnrnpa3 | Dendritic_Cells |
| 0.282299 | 0.957 | 0.842 | 1.82E-20 | 13 | Rpl11 | Dendritic_Cells |
| 0.330904 | 0.985 | 0.907 | 1.04E-19 | 13 | Rpl37 | Dendritic_Cells |
| 0.275484 | 0.927 | 0.832 | 2.19E-19 | 13 | Rpl21 | Dendritic_Cells |
| 0.283368 | 0.904 | 0.75 | 5.20E-19 | 13 | Rpl14 | Dendritic_Cells |
| 0.25342 | 0.981 | 0.901 | 5.50E-17 | 13 | Rpl38 | Dendritic_Cells |
| 0.260854 | 0.959 | 0.837 | 9.63E-17 | 13 | Ppia | Dendritic_Cells |
| 0.258129 | 0.932 | 0.832 | 1.85E-16 | 13 | Rpl30 | Dendritic_Cells |
| 0.277689 | 0.359 | 0.192 | 4.29E-16 | 13 | Wnk1 | Dendritic_Cells |
| 0.26074 | 0.951 | 0.796 | 1.47E-15 | 13 | Rpl35 | Dendritic_Cells |
| 0.53199 | 0.391 | 0.227 | 3.65E-15 | 13 | Ifi27l2a | Dendritic_Cells |
| 0.274712 | 0.226 | 0.114 | 2.38E-10 | 13 | Hilpda | Dendritic_Cells |
| 0.78733 | 0.662 | 0.638 | 3.27E-10 | 13 | Ifitm3 | Dendritic_Cells |
| 0.284128 | 0.615 | 0.475 | 6.68E-09 | 13 | Npc2 | Dendritic_Cells |
| 0.357722 | 0.12 | 0.05 | 2.66E-08 | 13 | Fscn1 | Dendritic_Cells |
| 0.353594 | 0.707 | 0.625 | 1.34E-07 | 13 | Anxa2 | Dendritic_Cells |
| 0.257713 | 0.735 | 0.639 | 5.52E-05 | 13 | S100a10 | Dendritic_Cells |
| 0.359337 | 0.84 | 0.732 | 5.96E-05 | 13 | Tmsb10 | Dendritic_Cells |
| 0.310861 | 0.145 | 0.082 | 0.020737 | 13 | Ccl4 | Dendritic_Cells |
| 2.444726 | 0.988 | 0.222 | 0 | 14 | Lyz2 | Macrophages (Mac 1.4) |
| 1.992043 | 0.755 | 0.062 | 0 | 14 | C1qa | Macrophages (Mac 1.4) |
| 1.89507 | 0.869 | 0.091 | 0 | 14 | Ms4a7 | Macrophages (Mac 1.4) |
| 1.891008 | 0.762 | 0.069 | 0 | 14 | C1qb | Macrophages (Mac 1.4) |
| 1.863636 | 0.621 | 0.067 | 0 | 14 | Pf4 | Macrophages (Mac 1.4) |
| 1.542738 | 0.896 | 0.154 | 0 | 14 | Lgmn | Macrophages (Mac 1.4) |
| 1.428341 | 0.703 | 0.056 | 0 | 14 | C1qc | Macrophages (Mac 1.4) |
| 0.83875 | 0.658 | 0.07 | 0 | 14 | Trem2 | Macrophages (Mac 1.4) |
| 0.762971 | 0.589 | 0.05 | 0 | 14 | C3ar1 | Macrophages (Mac 1.4) |
| 0.583948 | 0.453 | 0.032 | 0 | 14 | Adgre1 | Macrophages (Mac 1.4) |
| 0.913156 | 0.681 | 0.099 | 5.65E-298 | 14 | Dab2 | Macrophages (Mac 1.4) |
| 0.951661 | 0.673 | 0.101 | 4.31E-285 | 14 | Pltp | Macrophages (Mac 1.4) |
| 1.307975 | 0.921 | 0.235 | 9.72E-272 | 14 | Grn | Macrophages (Mac 1.4) |
| 1.337176 | 0.921 | 0.208 | 1.58E-260 | 14 | Ctss | Macrophages (Mac 1.4) |
| 0.890958 | 0.725 | 0.123 | 2.56E-258 | 14 | Ms4a6c | Macrophages (Mac 1.4) |
| 0.598862 | 0.374 | 0.032 | 1.42E-253 | 14 | Stab1 | Macrophages (Mac 1.4) |
| 0.827912 | 0.624 | 0.104 | 8.47E-231 | 14 | Sirpa | Macrophages (Mac 1.4) |
| 0.604577 | 0.371 | 0.035 | 5.39E-230 | 14 | Mrc1 | Macrophages (Mac 1.4) |
| 0.696065 | 0.609 | 0.098 | 3.38E-222 | 14 | Ms4a6d | Macrophages (Mac 1.4) |
| 0.800177 | 0.686 | 0.127 | 8.59E-222 | 14 | Cd68 | Macrophages (Mac 1.4) |
| 0.682732 | 0.554 | 0.084 | 7.28E-219 | 14 | Aif1 | Macrophages (Mac 1.4) |
| 0.950274 | 0.644 | 0.115 | 4.93E-218 | 14 | Mafb | Macrophages (Mac 1.4) |
| 2.465052 | 0.869 | 0.237 | 2.91E-216 | 14 | Apoe | Macrophages (Mac 1.4) |
| 0.725764 | 0.616 | 0.105 | 5.60E-214 | 14 | Cxcl16 | Macrophages (Mac 1.4) |
| 0.6757 | 0.619 | 0.108 | 2.32E-209 | 14 | Csf1r | Macrophages (Mac 1.4) |
| 0.616066 | 0.557 | 0.089 | 9.26E-205 | 14 | Unc93b1 | Macrophages (Mac 1.4) |
| 0.869224 | 0.673 | 0.134 | 1.53E-199 | 14 | Mpeg1 | Macrophages (Mac 1.4) |
| 0.304837 | 0.151 | 0.006 | 1.50E-182 | 14 | Fcrls | Macrophages (Mac 1.4) |
| 0.465153 | 0.406 | 0.057 | 2.34E-166 | 14 | Ms4a6b | Macrophages (Mac 1.4) |
| 1.588924 | 0.96 | 0.522 | 2.63E-163 | 14 | Ctsb | Macrophages (Mac 1.4) |
| 0.493794 | 0.438 | 0.068 | 7.35E-163 | 14 | Pid1 | Macrophages (Mac 1.4) |
| 0.380805 | 0.3 | 0.033 | 3.70E-156 | 14 | Clec12a | Macrophages (Mac 1.4) |
| 0.972355 | 0.812 | 0.273 | 1.98E-154 | 14 | Ctsc | Macrophages (Mac 1.4) |
| 1.340414 | 1 | 0.91 | 1.35E-150 | 14 | Ftl1 | Macrophages (Mac 1.4) |
| 1.019365 | 0.542 | 0.117 | 1.73E-149 | 14 | Selenop | Macrophages (Mac 1.4) |
| 0.809291 | 0.832 | 0.26 | 1.26E-148 | 14 | Laptm5 | Macrophages (Mac 1.4) |
| 0.631806 | 0.507 | 0.099 | 2.88E-145 | 14 | Plin2 | Macrophages (Mac 1.4) |
| 0.510642 | 0.408 | 0.065 | 7.51E-145 | 14 | Msr1 | Macrophages (Mac 1.4) |
| 1.160124 | 0.928 | 0.381 | 1.67E-140 | 14 | Lgals3 | Macrophages (Mac 1.4) |
| 0.264212 | 0.196 | 0.015 | 5.53E-140 | 14 | Tmem106a | Macrophages (Mac 1.4) |
| 0.456991 | 0.428 | 0.075 | 6.66E-137 | 14 | Clec4a2 | Macrophages (Mac 1.4) |
| 1.083343 | 0.822 | 0.307 | 8.21E-137 | 14 | Ctsd | Macrophages (Mac 1.4) |
| 1.067218 | 0.933 | 0.46 | 1.00E-134 | 14 | Psap | Macrophages (Mac 1.4) |
| 0.732748 | 0.698 | 0.188 | 1.30E-133 | 14 | Ifi30 | Macrophages (Mac 1.4) |
| 0.916028 | 0.938 | 0.304 | 1.52E-133 | 14 | Fcer1g | Macrophages (Mac 1.4) |
| 0.340354 | 0.265 | 0.03 | 1.88E-132 | 14 | Clec4a1 | Macrophages (Mac 1.4) |
| 0.973206 | 0.913 | 0.392 | 1.12E-131 | 14 | Cyba | Macrophages (Mac 1.4) |
| 0.664425 | 0.681 | 0.192 | 2.51E-128 | 14 | Efhd2 | Macrophages (Mac 1.4) |
| 1.004163 | 0.889 | 0.445 | 2.28E-126 | 14 | Gpx1 | Macrophages (Mac 1.4) |
| 0.411967 | 0.361 | 0.058 | 7.03E-124 | 14 | Cd93 | Macrophages (Mac 1.4) |
| 0.527039 | 0.453 | 0.09 | 2.29E-123 | 14 | Abca1 | Macrophages (Mac 1.4) |
| 0.821297 | 0.941 | 0.487 | 1.29E-114 | 14 | Atp6v0c | Macrophages (Mac 1.4) |
| 0.869218 | 0.866 | 0.359 | 7.72E-114 | 14 | Cstb | Macrophages (Mac 1.4) |
| 0.479503 | 0.46 | 0.102 | 4.05E-111 | 14 | Rgs10 | Macrophages (Mac 1.4) |
| 0.333671 | 0.3 | 0.045 | 1.15E-109 | 14 | Gusb | Macrophages (Mac 1.4) |
| 0.671865 | 0.77 | 0.272 | 2.38E-109 | 14 | Ucp2 | Macrophages (Mac 1.4) |
| 0.568842 | 0.582 | 0.154 | 2.17E-108 | 14 | Cxcr4 | Macrophages (Mac 1.4) |
| 0.544185 | 0.552 | 0.147 | 7.01E-107 | 14 | Ctsa | Macrophages (Mac 1.4) |
| 0.31532 | 0.24 | 0.031 | 1.44E-106 | 14 | Pld4 | Macrophages (Mac 1.4) |
| 0.45572 | 0.436 | 0.096 | 7.65E-103 | 14 | Atp6v1a | Macrophages (Mac 1.4) |
| 0.525615 | 0.47 | 0.107 | 3.87E-102 | 14 | Ccl9 | Macrophages (Mac 1.4) |
| 0.454616 | 0.49 | 0.12 | 9.22E-100 | 14 | Fyb | Macrophages (Mac 1.4) |
| 0.630285 | 0.918 | 0.316 | 2.06E-97 | 14 | Tyrobp | Macrophages (Mac 1.4) |
| 0.449964 | 0.381 | 0.08 | 3.16E-97 | 14 | Hexb | Macrophages (Mac 1.4) |
| 0.479839 | 0.458 | 0.111 | 3.28E-97 | 14 | Ptpn18 | Macrophages (Mac 1.4) |
| 0.401441 | 0.394 | 0.083 | 3.54E-96 | 14 | Cd300c2 | Macrophages (Mac 1.4) |
| 0.354701 | 0.29 | 0.048 | 5.59E-96 | 14 | Tcirg1 | Macrophages (Mac 1.4) |
| 0.58239 | 0.7 | 0.223 | 9.59E-96 | 14 | Alox5ap | Macrophages (Mac 1.4) |
| 0.56411 | 0.78 | 0.269 | 5.50E-95 | 14 | Lcp1 | Macrophages (Mac 1.4) |
| 0.444588 | 0.119 | 0.008 | 5.00E-93 | 14 | Fxyd2 | Macrophages (Mac 1.4) |
| 0.976932 | 0.998 | 0.899 | 5.88E-93 | 14 | Tmsb4x | Macrophages (Mac 1.4) |
| 0.728831 | 0.795 | 0.372 | 4.11E-92 | 14 | Lamp1 | Macrophages (Mac 1.4) |
| 0.549316 | 0.743 | 0.278 | 7.17E-92 | 14 | Atp6v0b | Macrophages (Mac 1.4) |
| 0.29718 | 0.245 | 0.037 | 6.24E-90 | 14 | Clec4a3 | Macrophages (Mac 1.4) |
| 0.566616 | 0.693 | 0.249 | 2.31E-88 | 14 | Sdcbp | Macrophages (Mac 1.4) |
| 0.348487 | 0.334 | 0.066 | 2.61E-88 | 14 | Cyfip1 | Macrophages (Mac 1.4) |
| 0.281186 | 0.176 | 0.02 | 3.11E-88 | 14 | Cebpa | Macrophages (Mac 1.4) |
| 0.298838 | 0.24 | 0.036 | 4.20E-88 | 14 | Ehd4 | Macrophages (Mac 1.4) |
| 0.906464 | 0.738 | 0.315 | 6.04E-88 | 14 | Tgfbi | Macrophages (Mac 1.4) |
| 0.848017 | 0.973 | 0.654 | 9.17E-86 | 14 | B2m | Macrophages (Mac 1.4) |
| 0.28275 | 0.218 | 0.031 | 1.40E-85 | 14 | Kcnn4 | Macrophages (Mac 1.4) |
| 0.413014 | 0.351 | 0.076 | 2.48E-83 | 14 | Cfp | Macrophages (Mac 1.4) |
| 0.596142 | 0.651 | 0.247 | 4.76E-83 | 14 | Aprt | Macrophages (Mac 1.4) |
| 0.463066 | 0.604 | 0.191 | 4.65E-82 | 14 | Spi1 | Macrophages (Mac 1.4) |
| 0.43189 | 0.406 | 0.097 | 1.26E-81 | 14 | Cybb | Macrophages (Mac 1.4) |
| 0.342742 | 0.21 | 0.03 | 2.49E-81 | 14 | Cd72 | Macrophages (Mac 1.4) |
| 0.595519 | 0.76 | 0.327 | 8.77E-79 | 14 | Ctsz | Macrophages (Mac 1.4) |
| 0.70377 | 0.921 | 0.644 | 3.02E-77 | 14 | Cst3 | Macrophages (Mac 1.4) |
| 0.326636 | 0.3 | 0.06 | 3.86E-77 | 14 | Snx5 | Macrophages (Mac 1.4) |
| 0.487563 | 0.5 | 0.153 | 1.73E-76 | 14 | Cltc | Macrophages (Mac 1.4) |
| 0.383642 | 0.401 | 0.1 | 2.85E-76 | 14 | Fcgr3 | Macrophages (Mac 1.4) |
| 0.560555 | 0.579 | 0.196 | 3.00E-76 | 14 | Ninj1 | Macrophages (Mac 1.4) |
| 0.327625 | 0.302 | 0.062 | 3.16E-75 | 14 | Plekho1 | Macrophages (Mac 1.4) |
| 0.29805 | 0.255 | 0.047 | 1.01E-72 | 14 | Mfsd1 | Macrophages (Mac 1.4) |
| 0.427486 | 0.557 | 0.182 | 3.90E-72 | 14 | Cotl1 | Macrophages (Mac 1.4) |
| 0.586903 | 0.634 | 0.245 | 1.04E-71 | 14 | Atp2b1 | Macrophages (Mac 1.4) |
| 0.297521 | 0.27 | 0.053 | 3.35E-70 | 14 | Pirb | Macrophages (Mac 1.4) |
| 0.548458 | 0.609 | 0.229 | 5.43E-70 | 14 | Capg | Macrophages (Mac 1.4) |
| 0.623448 | 0.834 | 0.469 | 7.02E-70 | 14 | Npc2 | Macrophages (Mac 1.4) |
| 0.453465 | 0.46 | 0.14 | 3.91E-69 | 14 | BC005537 | Macrophages (Mac 1.4) |
| 0.926418 | 0.891 | 0.532 | 4.00E-67 | 14 | S100a4 | Macrophages (Mac 1.4) |
| 0.280094 | 0.24 | 0.045 | 2.03E-66 | 14 | Gpr137b | Macrophages (Mac 1.4) |
| 0.592254 | 0.817 | 0.423 | 9.88E-66 | 14 | Atox1 | Macrophages (Mac 1.4) |
| 0.408986 | 0.512 | 0.162 | 2.15E-65 | 14 | C5ar1 | Macrophages (Mac 1.4) |
| 0.40619 | 0.51 | 0.17 | 4.32E-65 | 14 | Vamp8 | Macrophages (Mac 1.4) |
| 0.291159 | 0.262 | 0.054 | 8.25E-65 | 14 | Glul | Macrophages (Mac 1.4) |
| 0.477656 | 0.671 | 0.25 | 2.66E-63 | 14 | Slfn2 | Macrophages (Mac 1.4) |
| 0.619436 | 0.822 | 0.469 | 5.45E-62 | 14 | Sh3bgrl3 | Macrophages (Mac 1.4) |
| 0.686466 | 0.983 | 0.647 | 2.72E-60 | 14 | H2-D1 | Macrophages (Mac 1.4) |
| 0.681647 | 0.889 | 0.52 | 6.33E-60 | 14 | H2-K1 | Macrophages (Mac 1.4) |
| 0.303144 | 0.24 | 0.05 | 2.70E-58 | 14 | Anxa4 | Macrophages (Mac 1.4) |
| 0.865704 | 0.941 | 0.759 | 7.97E-57 | 14 | Itm2b | Macrophages (Mac 1.4) |
| 0.650344 | 0.819 | 0.48 | 2.61E-56 | 14 | Ahnak | Macrophages (Mac 1.4) |
| 0.510524 | 0.871 | 0.54 | 1.50E-55 | 14 | Arpc2 | Macrophages (Mac 1.4) |
| 0.469661 | 0.683 | 0.333 | 2.16E-55 | 14 | Akr1a1 | Macrophages (Mac 1.4) |
| 0.569511 | 0.938 | 0.791 | 2.82E-55 | 14 | Pfn1 | Macrophages (Mac 1.4) |
| 0.670629 | 0.995 | 0.959 | 5.14E-55 | 14 | Actb | Macrophages (Mac 1.4) |
| 0.381481 | 0.49 | 0.174 | 2.94E-54 | 14 | Slc6a6 | Macrophages (Mac 1.4) |
| 0.319305 | 0.342 | 0.096 | 1.46E-53 | 14 | Creg1 | Macrophages (Mac 1.4) |
| 0.397658 | 0.45 | 0.153 | 1.64E-53 | 14 | Arl4c | Macrophages (Mac 1.4) |
| 0.555784 | 0.827 | 0.53 | 3.46E-52 | 14 | Emp3 | Macrophages (Mac 1.4) |
| 0.27796 | 0.25 | 0.058 | 5.25E-52 | 14 | Sgpl1 | Macrophages (Mac 1.4) |
| 0.388257 | 0.597 | 0.24 | 1.40E-51 | 14 | Arhgdib | Macrophages (Mac 1.4) |
| 0.313469 | 0.309 | 0.084 | 3.48E-51 | 14 | Man2b1 | Macrophages (Mac 1.4) |
| 0.296594 | 0.322 | 0.09 | 1.29E-50 | 14 | Rala | Macrophages (Mac 1.4) |
| 0.384492 | 0.468 | 0.172 | 7.77E-50 | 14 | Hexa | Macrophages (Mac 1.4) |
| 0.3377 | 0.359 | 0.109 | 8.21E-50 | 14 | Itgam | Macrophages (Mac 1.4) |
| 0.452386 | 0.483 | 0.189 | 7.00E-49 | 14 | Plec | Macrophages (Mac 1.4) |
| 0.527476 | 0.512 | 0.198 | 1.45E-48 | 14 | Id2 | Macrophages (Mac 1.4) |
| 0.482625 | 0.582 | 0.254 | 5.21E-48 | 14 | Zeb2 | Macrophages (Mac 1.4) |
| 0.429083 | 0.995 | 0.973 | 8.00E-47 | 14 | Rps29 | Macrophages (Mac 1.4) |
| 0.318647 | 0.478 | 0.175 | 4.95E-46 | 14 | Card19 | Macrophages (Mac 1.4) |
| 0.334358 | 0.359 | 0.113 | 1.70E-45 | 14 | Adam8 | Macrophages (Mac 1.4) |
| 0.416049 | 0.584 | 0.268 | 5.21E-45 | 14 | Bri3 | Macrophages (Mac 1.4) |
| 0.397066 | 0.54 | 0.232 | 1.51E-44 | 14 | Arhgdia | Macrophages (Mac 1.4) |
| 0.265744 | 0.265 | 0.07 | 3.20E-44 | 14 | Gns | Macrophages (Mac 1.4) |
| 0.37162 | 0.629 | 0.288 | 2.61E-43 | 14 | Arpc5 | Macrophages (Mac 1.4) |
| 0.396938 | 0.324 | 0.097 | 3.64E-43 | 14 | Rgs1 | Macrophages (Mac 1.4) |
| 0.442024 | 0.837 | 0.517 | 1.30E-42 | 14 | Arpc1b | Macrophages (Mac 1.4) |
| 0.343565 | 0.599 | 0.255 | 3.94E-41 | 14 | Coro1a | Macrophages (Mac 1.4) |
| 0.309024 | 0.488 | 0.185 | 1.42E-40 | 14 | Lst1 | Macrophages (Mac 1.4) |
| 0.286168 | 0.213 | 0.051 | 2.47E-40 | 14 | F13a1 | Macrophages (Mac 1.4) |
| 0.310355 | 0.3 | 0.091 | 1.58E-39 | 14 | Fcgr2b | Macrophages (Mac 1.4) |
| 0.418923 | 0.809 | 0.527 | 4.77E-39 | 14 | Cdc42 | Macrophages (Mac 1.4) |
| 0.252668 | 0.248 | 0.067 | 6.21E-39 | 14 | Atp6v1b2 | Macrophages (Mac 1.4) |
| 0.422474 | 0.832 | 0.574 | 7.96E-39 | 14 | Gnai2 | Macrophages (Mac 1.4) |
| 0.318452 | 0.166 | 0.034 | 1.25E-38 | 14 | Arg1 | Macrophages (Mac 1.4) |
| 0.366947 | 0.767 | 0.387 | 1.56E-38 | 14 | Fxyd5 | Macrophages (Mac 1.4) |
| 0.350586 | 0.354 | 0.12 | 1.75E-38 | 14 | Cd83 | Macrophages (Mac 1.4) |
| 0.467236 | 0.903 | 0.728 | 4.04E-38 | 14 | Calm1 | Macrophages (Mac 1.4) |
| 0.314166 | 0.342 | 0.119 | 7.35E-37 | 14 | Irf2bp2 | Macrophages (Mac 1.4) |
| 0.281627 | 0.347 | 0.122 | 2.56E-36 | 14 | Ap2s1 | Macrophages (Mac 1.4) |
| 0.430179 | 0.698 | 0.432 | 6.75E-36 | 14 | Clta | Macrophages (Mac 1.4) |
| 0.318159 | 0.344 | 0.123 | 1.79E-35 | 14 | Metrnl | Macrophages (Mac 1.4) |
| 0.404062 | 0.886 | 0.688 | 5.50E-35 | 14 | Cfl1 | Macrophages (Mac 1.4) |
| 0.273456 | 0.28 | 0.088 | 5.56E-35 | 14 | M6pr | Macrophages (Mac 1.4) |
| 0.291848 | 0.364 | 0.135 | 6.82E-35 | 14 | Bola2 | Macrophages (Mac 1.4) |
| 0.273461 | 0.364 | 0.134 | 1.14E-34 | 14 | Atp1a1 | Macrophages (Mac 1.4) |
| 0.310375 | 0.423 | 0.175 | 2.80E-33 | 14 | Actr2 | Macrophages (Mac 1.4) |
| 0.345336 | 0.163 | 0.036 | 3.54E-33 | 14 | Il10 | Macrophages (Mac 1.4) |
| 0.303798 | 0.391 | 0.149 | 4.39E-33 | 14 | Tlr2 | Macrophages (Mac 1.4) |
| 0.329644 | 0.468 | 0.208 | 7.61E-33 | 14 | Capza2 | Macrophages (Mac 1.4) |
| 0.363628 | 0.527 | 0.259 | 1.91E-32 | 14 | Rtn4 | Macrophages (Mac 1.4) |
| 0.328508 | 0.569 | 0.285 | 1.02E-30 | 14 | Rap1b | Macrophages (Mac 1.4) |
| 0.437858 | 0.488 | 0.235 | 2.21E-30 | 14 | Zfp36l2 | Macrophages (Mac 1.4) |
| 0.264153 | 0.275 | 0.093 | 3.77E-30 | 14 | Rab5c | Macrophages (Mac 1.4) |
| 0.295927 | 0.438 | 0.191 | 5.09E-30 | 14 | Wnk1 | Macrophages (Mac 1.4) |
| 0.349102 | 0.614 | 0.335 | 5.59E-30 | 14 | Cox5a | Macrophages (Mac 1.4) |
| 0.409499 | 0.785 | 0.521 | 1.58E-29 | 14 | Anxa5 | Macrophages (Mac 1.4) |
| 0.629699 | 0.478 | 0.226 | 3.43E-29 | 14 | Ifi27l2a | Macrophages (Mac 1.4) |
| 0.260923 | 0.411 | 0.171 | 5.88E-29 | 14 | Ncf4 | Macrophages (Mac 1.4) |
| 0.280978 | 0.364 | 0.146 | 1.00E-28 | 14 | Dazap2 | Macrophages (Mac 1.4) |
| 0.301965 | 0.339 | 0.133 | 1.31E-28 | 14 | Maf | Macrophages (Mac 1.4) |
| 0.387848 | 0.713 | 0.468 | 6.34E-27 | 14 | Clic1 | Macrophages (Mac 1.4) |
| 0.330689 | 0.233 | 0.076 | 8.18E-27 | 14 | Lpl | Macrophages (Mac 1.4) |
| 0.281783 | 0.418 | 0.186 | 2.41E-26 | 14 | Pitpna | Macrophages (Mac 1.4) |
| 0.262644 | 0.215 | 0.068 | 5.80E-26 | 14 | Mdm2 | Macrophages (Mac 1.4) |
| 0.31006 | 0.512 | 0.261 | 1.49E-25 | 14 | Gdi2 | Macrophages (Mac 1.4) |
| 0.34735 | 0.663 | 0.402 | 1.60E-23 | 14 | Tspo | Macrophages (Mac 1.4) |
| 0.312024 | 0.624 | 0.361 | 6.70E-23 | 14 | Iqgap1 | Macrophages (Mac 1.4) |
| 0.252037 | 0.27 | 0.103 | 6.97E-23 | 14 | Nrp2 | Macrophages (Mac 1.4) |
| 0.340564 | 0.762 | 0.582 | 1.15E-22 | 14 | Prdx1 | Macrophages (Mac 1.4) |
| 0.284424 | 0.562 | 0.314 | 1.93E-21 | 14 | Cd47 | Macrophages (Mac 1.4) |
| 0.334215 | 0.337 | 0.148 | 1.35E-20 | 14 | Hmox1 | Macrophages (Mac 1.4) |
| 0.36106 | 0.54 | 0.305 | 2.93E-20 | 14 | Ly6e | Macrophages (Mac 1.4) |
| 0.315173 | 0.621 | 0.37 | 4.99E-20 | 14 | Cd44 | Macrophages (Mac 1.4) |
| 0.286472 | 0.557 | 0.328 | 7.34E-20 | 14 | Sh3glb1 | Macrophages (Mac 1.4) |
| 0.437391 | 0.985 | 0.83 | 9.76E-20 | 14 | Vim | Macrophages (Mac 1.4) |
| 0.272509 | 0.594 | 0.36 | 1.30E-18 | 14 | Atp6v0e | Macrophages (Mac 1.4) |
| 0.377858 | 0.505 | 0.295 | 7.90E-18 | 14 | Fabp5 | Macrophages (Mac 1.4) |
| 0.252486 | 0.468 | 0.261 | 3.91E-17 | 14 | Uqcr11 | Macrophages (Mac 1.4) |
| 0.430804 | 0.807 | 0.637 | 6.14E-17 | 14 | S100a10 | Macrophages (Mac 1.4) |
| 0.430992 | 0.814 | 0.634 | 8.18E-17 | 14 | Lmna | Macrophages (Mac 1.4) |
| 0.310015 | 0.597 | 0.385 | 1.04E-16 | 14 | Pkm | Macrophages (Mac 1.4) |
| 0.307495 | 0.411 | 0.225 | 1.27E-16 | 14 | Serinc3 | Macrophages (Mac 1.4) |
| 0.302467 | 0.579 | 0.376 | 3.26E-16 | 14 | Atpif1 | Macrophages (Mac 1.4) |
| 0.261426 | 0.75 | 0.516 | 1.10E-15 | 14 | H2afz | Macrophages (Mac 1.4) |
| 0.555869 | 0.901 | 0.786 | 3.94E-15 | 14 | Gm42418 | Macrophages (Mac 1.4) |
| 0.262657 | 0.438 | 0.244 | 5.20E-15 | 14 | Sdc4 | Macrophages (Mac 1.4) |
| 0.368724 | 0.911 | 0.774 | 5.36E-15 | 14 | Hspa8 | Macrophages (Mac 1.4) |
| 0.265491 | 0.656 | 0.464 | 1.68E-13 | 14 | Pabpc1 | Macrophages (Mac 1.4) |
| 0.26193 | 0.478 | 0.282 | 1.74E-13 | 14 | Sat1 | Macrophages (Mac 1.4) |
| 0.280813 | 0.599 | 0.398 | 1.76E-13 | 14 | Aldoa | Macrophages (Mac 1.4) |
| 0.301935 | 0.676 | 0.514 | 1.16E-12 | 14 | Eif4a1 | Macrophages (Mac 1.4) |
| 0.257793 | 0.559 | 0.36 | 1.52E-12 | 14 | Emp1 | Macrophages (Mac 1.4) |
| 0.283375 | 0.267 | 0.134 | 9.34E-11 | 14 | Tuba1b | Macrophages (Mac 1.4) |
| 0.255189 | 0.95 | 0.838 | 5.30E-10 | 14 | Ppia | Macrophages (Mac 1.4) |
| 0.250843 | 0.443 | 0.297 | 7.22E-07 | 14 | Sqstm1 | Macrophages (Mac 1.4) |
| 0.364102 | 0.988 | 0.942 | 8.79E-06 | 14 | mt-Co1 | Macrophages (Mac 1.4) |
| 0.390151 | 0.542 | 0.422 | 3.11E-05 | 14 | Ctsl | Macrophages (Mac 1.4) |
| 0.322585 | 0.188 | 0.106 | 0.000566 | 14 | Hspa1a | Macrophages (Mac 1.4) |
| 0.310101 | 0.879 | 0.771 | 0.001548 | 14 | Crip1 | Macrophages (Mac 1.4) |
| 2.001092 | 0.288 | 0.001 | 0 | 15 | Il17a | T_Cells (Tcell 1.1) |
| 1.987297 | 0.882 | 0.015 | 0 | 15 | Cd3g | T_Cells (Tcell 1.1) |
| 1.675919 | 0.481 | 0.003 | 0 | 15 | Trdc | T_Cells (Tcell 1.1) |
| 1.652059 | 0.824 | 0.146 | 0 | 15 | Emb | T_Cells (Tcell 1.1) |
| 1.623703 | 0.731 | 0.004 | 0 | 15 | Cxcr6 | T_Cells (Tcell 1.1) |
| 1.620276 | 0.794 | 0.007 | 0 | 15 | Icos | T_Cells (Tcell 1.1) |
| 1.391301 | 0.706 | 0.063 | 0 | 15 | Ltb | T_Cells (Tcell 1.1) |
| 1.351774 | 0.764 | 0.042 | 0 | 15 | Il7r | T_Cells (Tcell 1.1) |
| 1.210001 | 0.775 | 0.069 | 0 | 15 | Rora | T_Cells (Tcell 1.1) |
| 1.156866 | 0.681 | 0.02 | 0 | 15 | Trbc2 | T_Cells (Tcell 1.1) |
| 1.128463 | 0.486 | 0.011 | 0 | 15 | Trbc1 | T_Cells (Tcell 1.1) |
| 1.117579 | 0.354 | 0.004 | 0 | 15 | Ctla4 | T_Cells (Tcell 1.1) |
| 1.096707 | 0.725 | 0.011 | 0 | 15 | Cd3e | T_Cells (Tcell 1.1) |
| 1.051659 | 0.72 | 0.027 | 0 | 15 | Cd3d | T_Cells (Tcell 1.1) |
| 1.010544 | 0.464 | 0.006 | 0 | 15 | Tnfrsf9 | T_Cells (Tcell 1.1) |
| 0.936747 | 0.39 | 0.015 | 0 | 15 | AW112010 | T_Cells (Tcell 1.1) |
| 0.9191 | 0.258 | 0.005 | 0 | 15 | Tnfrsf4 | T_Cells (Tcell 1.1) |
| 0.908536 | 0.525 | 0.002 | 0 | 15 | Pdcd1 | T_Cells (Tcell 1.1) |
| 0.90683 | 0.69 | 0.074 | 0 | 15 | Ets1 | T_Cells (Tcell 1.1) |
| 0.841731 | 0.549 | 0.035 | 0 | 15 | Ramp1 | T_Cells (Tcell 1.1) |
| 0.797362 | 0.569 | 0.022 | 0 | 15 | Ptprcap | T_Cells (Tcell 1.1) |
| 0.776269 | 0.409 | 0.001 | 0 | 15 | Tcrg-C1 | T_Cells (Tcell 1.1) |
| 0.765857 | 0.324 | 0.005 | 0 | 15 | Ikzf2 | T_Cells (Tcell 1.1) |
| 0.754149 | 0.514 | 0.029 | 0 | 15 | D16Ertd472e | T_Cells (Tcell 1.1) |
| 0.74686 | 0.516 | 0.004 | 0 | 15 | Il2rb | T_Cells (Tcell 1.1) |
| 0.741257 | 0.201 | 0.002 | 0 | 15 | Gzmb | T_Cells (Tcell 1.1) |
| 0.676737 | 0.387 | 0.011 | 0 | 15 | Trac | T_Cells (Tcell 1.1) |
| 0.648118 | 0.346 | 0.001 | 0 | 15 | Cd163l1 | T_Cells (Tcell 1.1) |
| 0.609693 | 0.442 | 0.009 | 0 | 15 | Lat | T_Cells (Tcell 1.1) |
| 0.592245 | 0.357 | 0.002 | 0 | 15 | Il2ra | T_Cells (Tcell 1.1) |
| 0.558148 | 0.426 | 0.017 | 0 | 15 | 01-Sep | T_Cells (Tcell 1.1) |
| 0.534075 | 0.299 | 0.012 | 0 | 15 | Serpinb1a | T_Cells (Tcell 1.1) |
| 0.510674 | 0.357 | 0.006 | 0 | 15 | Ikzf3 | T_Cells (Tcell 1.1) |
| 0.499965 | 0.269 | 0 | 0 | 15 | Trdv4 | T_Cells (Tcell 1.1) |
| 0.460767 | 0.324 | 0.015 | 0 | 15 | Smco4 | T_Cells (Tcell 1.1) |
| 0.452061 | 0.316 | 0.009 | 0 | 15 | Sh2d2a | T_Cells (Tcell 1.1) |
| 0.4284 | 0.321 | 0.015 | 0 | 15 | Itgb7 | T_Cells (Tcell 1.1) |
| 0.414655 | 0.294 | 0.007 | 0 | 15 | Skap1 | T_Cells (Tcell 1.1) |
| 0.389908 | 0.239 | 0.008 | 0 | 15 | Blk | T_Cells (Tcell 1.1) |
| 0.368741 | 0.231 | 0.004 | 0 | 15 | Pkp3 | T_Cells (Tcell 1.1) |
| 0.363567 | 0.245 | 0.003 | 0 | 15 | Cd247 | T_Cells (Tcell 1.1) |
| 0.335101 | 0.19 | 0.003 | 0 | 15 | Cd5 | T_Cells (Tcell 1.1) |
| 0.322515 | 0.231 | 0.002 | 0 | 15 | Zap70 | T_Cells (Tcell 1.1) |
| 0.273783 | 0.165 | 0.002 | 0 | 15 | Il18r1 | T_Cells (Tcell 1.1) |
| 0.264699 | 0.151 | 0.001 | 0 | 15 | Krt83 | T_Cells (Tcell 1.1) |
| 0.295432 | 0.192 | 0.005 | 2.21E-303 | 15 | Bcl11b | T_Cells (Tcell 1.1) |
| 0.279452 | 0.157 | 0.002 | 1.58E-301 | 15 | Acpp | T_Cells (Tcell 1.1) |
| 0.254472 | 0.181 | 0.004 | 9.44E-296 | 15 | Prelid2 | T_Cells (Tcell 1.1) |
| 0.350428 | 0.247 | 0.01 | 7.52E-288 | 15 | AU020206 | T_Cells (Tcell 1.1) |
| 0.81349 | 0.538 | 0.057 | 1.03E-282 | 15 | Gpr183 | T_Cells (Tcell 1.1) |
| 0.308032 | 0.228 | 0.008 | 6.59E-280 | 15 | Cish | T_Cells (Tcell 1.1) |
| 0.480339 | 0.245 | 0.01 | 1.16E-270 | 15 | Tnfrsf18 | T_Cells (Tcell 1.1) |
| 0.302852 | 0.187 | 0.006 | 1.34E-258 | 15 | Klrk1 | T_Cells (Tcell 1.1) |
| 0.569251 | 0.42 | 0.038 | 1.91E-253 | 15 | Aebp2 | T_Cells (Tcell 1.1) |
| 0.515976 | 0.365 | 0.028 | 2.67E-251 | 15 | Dgat1 | T_Cells (Tcell 1.1) |
| 0.5529 | 0.299 | 0.018 | 5.15E-250 | 15 | Cd2 | T_Cells (Tcell 1.1) |
| 0.899351 | 0.596 | 0.083 | 3.27E-247 | 15 | Crem | T_Cells (Tcell 1.1) |
| 0.336307 | 0.181 | 0.006 | 5.67E-236 | 15 | Cd28 | T_Cells (Tcell 1.1) |
| 0.29522 | 0.187 | 0.007 | 7.62E-233 | 15 | Lck | T_Cells (Tcell 1.1) |
| 1.432251 | 0.602 | 0.091 | 6.33E-228 | 15 | Rgs1 | T_Cells (Tcell 1.1) |
| 0.42694 | 0.286 | 0.018 | 8.57E-228 | 15 | Cd69 | T_Cells (Tcell 1.1) |
| 0.932825 | 0.357 | 0.031 | 1.79E-223 | 15 | Ramp3 | T_Cells (Tcell 1.1) |
| 0.517808 | 0.387 | 0.036 | 3.70E-223 | 15 | Vgll4 | T_Cells (Tcell 1.1) |
| 0.748424 | 0.552 | 0.078 | 8.33E-223 | 15 | Ppp1cc | T_Cells (Tcell 1.1) |
| 0.353395 | 0.242 | 0.013 | 1.66E-221 | 15 | Ptpn22 | T_Cells (Tcell 1.1) |
| 0.6715 | 0.426 | 0.045 | 5.21E-221 | 15 | Nrip1 | T_Cells (Tcell 1.1) |
| 0.308644 | 0.217 | 0.01 | 1.67E-220 | 15 | Rhof | T_Cells (Tcell 1.1) |
| 0.822862 | 0.299 | 0.022 | 2.52E-217 | 15 | Hopx | T_Cells (Tcell 1.1) |
| 0.493518 | 0.371 | 0.035 | 5.74E-212 | 15 | Stk24 | T_Cells (Tcell 1.1) |
| 0.257429 | 0.184 | 0.007 | 1.17E-210 | 15 | Itk | T_Cells (Tcell 1.1) |
| 0.548544 | 0.431 | 0.05 | 8.78E-201 | 15 | Il2rg | T_Cells (Tcell 1.1) |
| 0.327232 | 0.255 | 0.017 | 1.04E-193 | 15 | Ppp1r16b | T_Cells (Tcell 1.1) |
| 0.389817 | 0.28 | 0.022 | 1.52E-186 | 15 | Tmem64 | T_Cells (Tcell 1.1) |
| 1.04006 | 0.72 | 0.15 | 3.61E-186 | 15 | Bcl2a1b | T_Cells (Tcell 1.1) |
| 0.261796 | 0.151 | 0.005 | 1.12E-184 | 15 | Gm45716 | T_Cells (Tcell 1.1) |
| 0.324772 | 0.225 | 0.014 | 6.05E-183 | 15 | Kdm2b | T_Cells (Tcell 1.1) |
| 0.494082 | 0.382 | 0.043 | 1.19E-182 | 15 | Orai1 | T_Cells (Tcell 1.1) |
| 0.543176 | 0.434 | 0.056 | 1.15E-179 | 15 | Hcst | T_Cells (Tcell 1.1) |
| 1.004761 | 0.797 | 0.228 | 1.11E-176 | 15 | Ndfip1 | T_Cells (Tcell 1.1) |
| 0.901337 | 0.566 | 0.1 | 1.38E-176 | 15 | Vps37b | T_Cells (Tcell 1.1) |
| 0.747183 | 0.53 | 0.091 | 1.85E-172 | 15 | Ubald2 | T_Cells (Tcell 1.1) |
| 0.539739 | 0.442 | 0.061 | 1.94E-172 | 15 | Nr3c1 | T_Cells (Tcell 1.1) |
| 1.261741 | 0.747 | 0.193 | 5.63E-168 | 15 | Id2 | T_Cells (Tcell 1.1) |
| 0.875792 | 0.626 | 0.141 | 5.64E-156 | 15 | Ifngr1 | T_Cells (Tcell 1.1) |
| 0.532382 | 0.404 | 0.056 | 6.16E-155 | 15 | Ccr2 | T_Cells (Tcell 1.1) |
| 0.378586 | 0.283 | 0.028 | 1.55E-153 | 15 | Gpr65 | T_Cells (Tcell 1.1) |
| 0.625736 | 0.489 | 0.084 | 2.53E-153 | 15 | Sec11c | T_Cells (Tcell 1.1) |
| 0.455108 | 0.338 | 0.04 | 9.83E-153 | 15 | Smap2 | T_Cells (Tcell 1.1) |
| 0.599193 | 0.426 | 0.064 | 1.10E-152 | 15 | Uhrf2 | T_Cells (Tcell 1.1) |
| 0.592556 | 0.467 | 0.077 | 1.18E-152 | 15 | Mbd2 | T_Cells (Tcell 1.1) |
| 0.57307 | 0.467 | 0.077 | 2.14E-150 | 15 | Cmtm7 | T_Cells (Tcell 1.1) |
| 0.466323 | 0.379 | 0.051 | 5.42E-149 | 15 | Cd82 | T_Cells (Tcell 1.1) |
| 0.260917 | 0.187 | 0.012 | 2.76E-148 | 15 | Mmd | T_Cells (Tcell 1.1) |
| 1.032711 | 0.852 | 0.298 | 9.16E-147 | 15 | Ly6e | T_Cells (Tcell 1.1) |
| 1.308087 | 0.956 | 0.52 | 8.43E-140 | 15 | H2-K1 | T_Cells (Tcell 1.1) |
| 0.645795 | 0.53 | 0.107 | 5.48E-138 | 15 | Ncor1 | T_Cells (Tcell 1.1) |
| 0.698791 | 0.637 | 0.15 | 3.51E-136 | 15 | Rac2 | T_Cells (Tcell 1.1) |
| 0.594016 | 0.662 | 0.145 | 2.71E-134 | 15 | Samsn1 | T_Cells (Tcell 1.1) |
| 0.429064 | 0.346 | 0.048 | 2.56E-132 | 15 | Pdcd4 | T_Cells (Tcell 1.1) |
| 0.447701 | 0.363 | 0.053 | 4.45E-131 | 15 | Limd2 | T_Cells (Tcell 1.1) |
| 0.357251 | 0.277 | 0.031 | 1.59E-130 | 15 | Ltb4r1 | T_Cells (Tcell 1.1) |
| 0.615958 | 0.245 | 0.024 | 3.87E-130 | 15 | Ctla2a | T_Cells (Tcell 1.1) |
| 1.349079 | 0.984 | 0.648 | 3.99E-128 | 15 | H2-D1 | T_Cells (Tcell 1.1) |
| 0.401351 | 0.294 | 0.036 | 6.77E-128 | 15 | Fnbp1 | T_Cells (Tcell 1.1) |
| 0.710927 | 0.626 | 0.153 | 6.44E-126 | 15 | Cytip | T_Cells (Tcell 1.1) |
| 0.611808 | 0.47 | 0.089 | 2.34E-125 | 15 | Fgl2 | T_Cells (Tcell 1.1) |
| 0.267887 | 0.179 | 0.013 | 6.48E-123 | 15 | Gimap3 | T_Cells (Tcell 1.1) |
| 0.344607 | 0.272 | 0.032 | 1.58E-122 | 15 | Zdhhc18 | T_Cells (Tcell 1.1) |
| 0.45783 | 0.299 | 0.039 | 7.62E-122 | 15 | Bcl2a1a | T_Cells (Tcell 1.1) |
| 0.326129 | 0.245 | 0.026 | 2.55E-121 | 15 | Il21r | T_Cells (Tcell 1.1) |
| 0.826757 | 0.544 | 0.128 | 2.96E-121 | 15 | Maf | T_Cells (Tcell 1.1) |
| 0.826369 | 0.703 | 0.227 | 7.14E-118 | 15 | Capg | T_Cells (Tcell 1.1) |
| 0.56205 | 0.478 | 0.098 | 6.40E-116 | 15 | Selplg | T_Cells (Tcell 1.1) |
| 1.012658 | 0.495 | 0.108 | 1.61E-115 | 15 | Hilpda | T_Cells (Tcell 1.1) |
| 0.358909 | 0.288 | 0.038 | 2.26E-113 | 15 | Znrf1 | T_Cells (Tcell 1.1) |
| 1.056636 | 0.225 | 0.024 | 4.92E-113 | 15 | Areg | T_Cells (Tcell 1.1) |
| 1.048199 | 0.714 | 0.259 | 3.71E-111 | 15 | Ostf1 | T_Cells (Tcell 1.1) |
| 0.294624 | 0.192 | 0.017 | 5.08E-111 | 15 | Gimap1 | T_Cells (Tcell 1.1) |
| 1.130576 | 0.426 | 0.089 | 4.36E-109 | 15 | Odc1 | T_Cells (Tcell 1.1) |
| 0.302606 | 0.22 | 0.023 | 4.71E-108 | 15 | P2ry10 | T_Cells (Tcell 1.1) |
| 0.623128 | 0.459 | 0.101 | 4.52E-106 | 15 | Cd164 | T_Cells (Tcell 1.1) |
| 1.260386 | 1 | 0.9 | 2.19E-104 | 15 | Tmsb4x | T_Cells (Tcell 1.1) |
| 0.467033 | 0.354 | 0.062 | 3.72E-102 | 15 | H2-Q7 | T_Cells (Tcell 1.1) |
| 0.373516 | 0.97 | 0.377 | 3.44E-101 | 15 | Srgn | T_Cells (Tcell 1.1) |
| 0.53075 | 0.5 | 0.119 | 7.43E-101 | 15 | Gna13 | T_Cells (Tcell 1.1) |
| 0.417051 | 0.352 | 0.061 | 1.53E-100 | 15 | Saraf | T_Cells (Tcell 1.1) |
| 0.617039 | 0.459 | 0.105 | 1.31E-99 | 15 | Furin | T_Cells (Tcell 1.1) |
| 0.259396 | 0.195 | 0.02 | 1.49E-99 | 15 | Madd | T_Cells (Tcell 1.1) |
| 0.259727 | 0.137 | 0.01 | 3.39E-99 | 15 | Cd27 | T_Cells (Tcell 1.1) |
| 0.497097 | 0.473 | 0.11 | 1.33E-96 | 15 | Prr13 | T_Cells (Tcell 1.1) |
| 0.308735 | 0.261 | 0.036 | 2.13E-95 | 15 | Dock2 | T_Cells (Tcell 1.1) |
| 0.556442 | 0.492 | 0.118 | 5.25E-94 | 15 | Traf1 | T_Cells (Tcell 1.1) |
| 0.685897 | 0.67 | 0.226 | 8.07E-94 | 15 | Myl12b | T_Cells (Tcell 1.1) |
| 0.720455 | 0.684 | 0.239 | 1.65E-93 | 15 | Arhgdib | T_Cells (Tcell 1.1) |
| 0.368619 | 0.36 | 0.067 | 2.31E-93 | 15 | Cd37 | T_Cells (Tcell 1.1) |
| 0.276212 | 0.198 | 0.022 | 2.76E-93 | 15 | Fam110a | T_Cells (Tcell 1.1) |
| 0.587396 | 0.563 | 0.162 | 5.01E-93 | 15 | Spcs2 | T_Cells (Tcell 1.1) |
| 0.39895 | 0.316 | 0.054 | 5.41E-92 | 15 | Leprotl1 | T_Cells (Tcell 1.1) |
| 0.652178 | 0.604 | 0.191 | 1.64E-90 | 15 | Akap13 | T_Cells (Tcell 1.1) |
| 0.815021 | 0.824 | 0.297 | 4.85E-90 | 15 | Cd52 | T_Cells (Tcell 1.1) |
| 0.286882 | 0.179 | 0.019 | 3.17E-86 | 15 | Ahr | T_Cells (Tcell 1.1) |
| 0.544201 | 0.657 | 0.207 | 2.45E-85 | 15 | Gmfg | T_Cells (Tcell 1.1) |
| 0.427766 | 0.352 | 0.071 | 4.44E-85 | 15 | Mier1 | T_Cells (Tcell 1.1) |
| 0.775802 | 1 | 0.98 | 7.99E-85 | 15 | Tpt1 | T_Cells (Tcell 1.1) |
| 0.434521 | 0.354 | 0.072 | 1.28E-83 | 15 | Tnfaip8 | T_Cells (Tcell 1.1) |
| 1.078164 | 0.92 | 0.635 | 1.31E-83 | 15 | S100a10 | T_Cells (Tcell 1.1) |
| 0.595102 | 0.582 | 0.182 | 2.34E-83 | 15 | Cotl1 | T_Cells (Tcell 1.1) |
| 0.527027 | 0.426 | 0.104 | 3.58E-83 | 15 | Anp32a | T_Cells (Tcell 1.1) |
| 0.364677 | 0.272 | 0.046 | 1.26E-79 | 15 | Isy1 | T_Cells (Tcell 1.1) |
| 0.488798 | 0.495 | 0.136 | 1.45E-79 | 15 | Psmb8 | T_Cells (Tcell 1.1) |
| 0.293714 | 0.234 | 0.035 | 1.21E-78 | 15 | AC149090.1 | T_Cells (Tcell 1.1) |
| 0.309632 | 0.258 | 0.042 | 4.89E-78 | 15 | Slc38a1 | T_Cells (Tcell 1.1) |
| 0.365221 | 0.272 | 0.047 | 5.82E-78 | 15 | Avpi1 | T_Cells (Tcell 1.1) |
| 0.970411 | 0.747 | 0.352 | 3.81E-75 | 15 | Zfp36l1 | T_Cells (Tcell 1.1) |
| 0.781545 | 0.761 | 0.356 | 5.37E-75 | 15 | Sub1 | T_Cells (Tcell 1.1) |
| 0.267828 | 0.22 | 0.033 | 2.52E-72 | 15 | Sms | T_Cells (Tcell 1.1) |
| 0.25641 | 0.198 | 0.028 | 2.42E-71 | 15 | Pon2 | T_Cells (Tcell 1.1) |
| 0.464664 | 0.365 | 0.087 | 1.17E-70 | 15 | Ankrd12 | T_Cells (Tcell 1.1) |
| 0.504442 | 0.527 | 0.171 | 1.70E-70 | 15 | Tle5 | T_Cells (Tcell 1.1) |
| 0.282885 | 0.203 | 0.031 | 2.06E-66 | 15 | Birc2 | T_Cells (Tcell 1.1) |
| 0.320335 | 0.283 | 0.057 | 2.03E-65 | 15 | Gng2 | T_Cells (Tcell 1.1) |
| 0.4223 | 0.442 | 0.124 | 2.04E-65 | 15 | Rab8b | T_Cells (Tcell 1.1) |
| 0.256205 | 0.22 | 0.036 | 4.21E-65 | 15 | Ttc14 | T_Cells (Tcell 1.1) |
| 0.372077 | 0.36 | 0.089 | 3.84E-64 | 15 | Celf2 | T_Cells (Tcell 1.1) |
| 0.286022 | 0.266 | 0.053 | 3.38E-62 | 15 | Fkbp3 | T_Cells (Tcell 1.1) |
| 0.40944 | 0.407 | 0.113 | 1.16E-61 | 15 | Ptpn18 | T_Cells (Tcell 1.1) |
| 0.30426 | 0.245 | 0.046 | 8.73E-61 | 15 | Gtf2b | T_Cells (Tcell 1.1) |
| 0.338845 | 0.269 | 0.056 | 4.36E-60 | 15 | Tut4 | T_Cells (Tcell 1.1) |
| 0.451923 | 0.525 | 0.173 | 1.23E-59 | 15 | Stk17b | T_Cells (Tcell 1.1) |
| 0.618145 | 0.585 | 0.22 | 1.33E-59 | 15 | Hmgb2 | T_Cells (Tcell 1.1) |
| 0.522478 | 0.651 | 0.255 | 3.00E-59 | 15 | Coro1a | T_Cells (Tcell 1.1) |
| 1.064036 | 0.882 | 0.595 | 3.61E-59 | 15 | Btg1 | T_Cells (Tcell 1.1) |
| 0.451954 | 0.429 | 0.131 | 2.29E-58 | 15 | Tpr | T_Cells (Tcell 1.1) |
| 0.345565 | 0.33 | 0.081 | 3.75E-58 | 15 | Ubl3 | T_Cells (Tcell 1.1) |
| 0.305575 | 0.228 | 0.043 | 1.08E-57 | 15 | Arhgap45 | T_Cells (Tcell 1.1) |
| 0.623502 | 0.885 | 0.782 | 2.11E-57 | 15 | Oaz1 | T_Cells (Tcell 1.1) |
| 0.419522 | 0.365 | 0.1 | 1.84E-56 | 15 | Rbpj | T_Cells (Tcell 1.1) |
| 0.71256 | 0.934 | 0.806 | 2.15E-56 | 15 | Rpl13a | T_Cells (Tcell 1.1) |
| 0.366405 | 0.316 | 0.078 | 9.20E-56 | 15 | Mdh1 | T_Cells (Tcell 1.1) |
| 0.320486 | 0.308 | 0.074 | 1.15E-55 | 15 | Ppp1r11 | T_Cells (Tcell 1.1) |
| 0.789787 | 0.915 | 0.532 | 2.39E-55 | 15 | S100a4 | T_Cells (Tcell 1.1) |
| 0.341769 | 0.28 | 0.064 | 1.29E-54 | 15 | Irs2 | T_Cells (Tcell 1.1) |
| 0.337981 | 0.349 | 0.094 | 6.06E-54 | 15 | Pdcd6 | T_Cells (Tcell 1.1) |
| 0.489675 | 0.651 | 0.266 | 1.07E-53 | 15 | Laptm5 | T_Cells (Tcell 1.1) |
| 0.596482 | 0.97 | 0.85 | 3.61E-53 | 15 | Rps13 | T_Cells (Tcell 1.1) |
| 0.655366 | 0.802 | 0.47 | 4.46E-53 | 15 | Sh3bgrl3 | T_Cells (Tcell 1.1) |
| 0.641482 | 0.495 | 0.192 | 5.53E-53 | 15 | Tmem176a | T_Cells (Tcell 1.1) |
| 0.584997 | 0.986 | 0.882 | 1.54E-52 | 15 | Rpl19 | T_Cells (Tcell 1.1) |
| 0.590996 | 0.967 | 0.839 | 2.62E-52 | 15 | Rpl17 | T_Cells (Tcell 1.1) |
| 0.276908 | 0.234 | 0.048 | 2.91E-52 | 15 | H2-T23 | T_Cells (Tcell 1.1) |
| 0.653951 | 0.931 | 0.656 | 6.49E-52 | 15 | B2m | T_Cells (Tcell 1.1) |
| 0.519664 | 0.541 | 0.214 | 2.09E-51 | 15 | Prdx6 | T_Cells (Tcell 1.1) |
| 0.268425 | 0.245 | 0.052 | 2.39E-51 | 15 | Runx3 | T_Cells (Tcell 1.1) |
| 0.476159 | 0.475 | 0.169 | 8.28E-51 | 15 | Shisa5 | T_Cells (Tcell 1.1) |
| 0.27237 | 0.236 | 0.049 | 1.01E-50 | 15 | Fam129a | T_Cells (Tcell 1.1) |
| 0.506408 | 0.64 | 0.277 | 1.42E-50 | 15 | Ucp2 | T_Cells (Tcell 1.1) |
| 0.84933 | 0.923 | 0.71 | 2.23E-50 | 15 | Junb | T_Cells (Tcell 1.1) |
| 0.516343 | 0.552 | 0.23 | 2.62E-50 | 15 | Prkar1a | T_Cells (Tcell 1.1) |
| 0.46445 | 0.541 | 0.213 | 3.41E-50 | 15 | Cnbp | T_Cells (Tcell 1.1) |
| 0.522835 | 0.648 | 0.311 | 4.38E-50 | 15 | Eif3h | T_Cells (Tcell 1.1) |
| 0.324746 | 0.445 | 0.144 | 1.25E-49 | 15 | Cdk2ap2 | T_Cells (Tcell 1.1) |
| 0.29848 | 0.272 | 0.065 | 3.87E-49 | 15 | Gpr132 | T_Cells (Tcell 1.1) |
| 0.496681 | 0.797 | 0.388 | 5.79E-49 | 15 | Fxyd5 | T_Cells (Tcell 1.1) |
| 0.336016 | 0.288 | 0.073 | 2.36E-48 | 15 | 4930523C07Rik | T_Cells (Tcell 1.1) |
| 0.261201 | 0.239 | 0.053 | 1.24E-47 | 15 | Emg1 | T_Cells (Tcell 1.1) |
| 0.29832 | 0.255 | 0.06 | 6.15E-47 | 15 | Zc3hav1 | T_Cells (Tcell 1.1) |
| 0.324377 | 0.321 | 0.09 | 3.96E-46 | 15 | Syf2 | T_Cells (Tcell 1.1) |
| 0.549531 | 0.975 | 0.851 | 4.53E-46 | 15 | Rps14 | T_Cells (Tcell 1.1) |
| 0.286283 | 0.269 | 0.067 | 3.03E-45 | 15 | Sptssa | T_Cells (Tcell 1.1) |
| 0.402906 | 0.385 | 0.126 | 2.57E-44 | 15 | Ccnd2 | T_Cells (Tcell 1.1) |
| 0.321688 | 0.33 | 0.097 | 4.74E-44 | 15 | Timm23 | T_Cells (Tcell 1.1) |
| 0.326317 | 0.319 | 0.091 | 9.86E-44 | 15 | Fam107b | T_Cells (Tcell 1.1) |
| 0.429805 | 0.467 | 0.175 | 1.47E-43 | 15 | Mbnl1 | T_Cells (Tcell 1.1) |
| 0.397712 | 0.464 | 0.176 | 3.30E-43 | 15 | S100a13 | T_Cells (Tcell 1.1) |
| 0.268496 | 0.223 | 0.05 | 3.66E-43 | 15 | Cldnd1 | T_Cells (Tcell 1.1) |
| 0.557697 | 0.937 | 0.763 | 4.31E-43 | 15 | Rpl12 | T_Cells (Tcell 1.1) |
| 0.31411 | 0.324 | 0.094 | 9.48E-43 | 15 | Arl6ip1 | T_Cells (Tcell 1.1) |
| 0.268999 | 0.261 | 0.066 | 1.83E-42 | 15 | Elf1 | T_Cells (Tcell 1.1) |
| 0.409464 | 0.492 | 0.198 | 2.07E-42 | 15 | Tmem50a | T_Cells (Tcell 1.1) |
| 0.330218 | 0.404 | 0.137 | 2.18E-42 | 15 | Trir | T_Cells (Tcell 1.1) |
| 0.568431 | 0.975 | 0.839 | 2.98E-42 | 15 | Rps7 | T_Cells (Tcell 1.1) |
| 0.295388 | 0.239 | 0.058 | 6.42E-42 | 15 | Cenpa | T_Cells (Tcell 1.1) |
| 0.34726 | 0.39 | 0.131 | 9.00E-42 | 15 | Supt4a | T_Cells (Tcell 1.1) |
| 0.483221 | 0.755 | 0.383 | 9.37E-42 | 15 | Pim1 | T_Cells (Tcell 1.1) |
| 0.303143 | 0.33 | 0.1 | 2.24E-41 | 15 | Ndufv3 | T_Cells (Tcell 1.1) |
| 0.504529 | 0.838 | 0.541 | 6.50E-41 | 15 | Arpc2 | T_Cells (Tcell 1.1) |
| 0.416599 | 0.459 | 0.181 | 9.60E-41 | 15 | Sri | T_Cells (Tcell 1.1) |
| 0.38718 | 0.404 | 0.144 | 1.76E-40 | 15 | Dnajb6 | T_Cells (Tcell 1.1) |
| 0.381457 | 0.321 | 0.099 | 2.43E-40 | 15 | Sdf4 | T_Cells (Tcell 1.1) |
| 0.469774 | 0.431 | 0.159 | 5.03E-40 | 15 | Cxcr4 | T_Cells (Tcell 1.1) |
| 0.512847 | 0.937 | 0.796 | 5.24E-40 | 15 | Rpl27 | T_Cells (Tcell 1.1) |
| 0.42571 | 0.473 | 0.191 | 1.17E-38 | 15 | Wnk1 | T_Cells (Tcell 1.1) |
| 0.325464 | 0.321 | 0.101 | 5.86E-38 | 15 | Smad7 | T_Cells (Tcell 1.1) |
| 0.668363 | 0.904 | 0.761 | 9.55E-38 | 15 | Itm2b | T_Cells (Tcell 1.1) |
| 0.617206 | 0.357 | 0.126 | 1.01E-37 | 15 | Ly6a | T_Cells (Tcell 1.1) |
| 0.445723 | 0.445 | 0.18 | 1.22E-37 | 15 | Tgfb1 | T_Cells (Tcell 1.1) |
| 0.534093 | 0.772 | 0.467 | 1.26E-37 | 15 | Arpc3 | T_Cells (Tcell 1.1) |
| 0.299441 | 0.335 | 0.109 | 2.07E-37 | 15 | Zfp706 | T_Cells (Tcell 1.1) |
| 0.343516 | 0.277 | 0.08 | 2.50E-37 | 15 | Rgs2 | T_Cells (Tcell 1.1) |
| 0.290607 | 0.266 | 0.075 | 2.71E-37 | 15 | Uqcrfs1 | T_Cells (Tcell 1.1) |
| 0.360905 | 0.459 | 0.185 | 2.73E-37 | 15 | Park7 | T_Cells (Tcell 1.1) |
| 0.463784 | 0.997 | 0.969 | 1.51E-36 | 15 | Fau | T_Cells (Tcell 1.1) |
| 0.271465 | 0.294 | 0.089 | 9.73E-36 | 15 | Dnajc8 | T_Cells (Tcell 1.1) |
| 0.27356 | 0.261 | 0.074 | 1.12E-35 | 15 | Clint1 | T_Cells (Tcell 1.1) |
| 0.363352 | 0.434 | 0.175 | 1.59E-35 | 15 | Ube2i | T_Cells (Tcell 1.1) |
| 0.292413 | 0.286 | 0.086 | 1.82E-35 | 15 | Tmem123 | T_Cells (Tcell 1.1) |
| 0.499106 | 0.975 | 0.842 | 2.14E-35 | 15 | Rpl11 | T_Cells (Tcell 1.1) |
| 0.357725 | 0.396 | 0.149 | 2.59E-35 | 15 | Sumo1 | T_Cells (Tcell 1.1) |
| 0.297113 | 0.368 | 0.129 | 4.31E-35 | 15 | Pak2 | T_Cells (Tcell 1.1) |
| 0.494259 | 0.747 | 0.468 | 4.60E-35 | 15 | Clic1 | T_Cells (Tcell 1.1) |
| 0.259643 | 0.264 | 0.076 | 1.59E-34 | 15 | Tprgl | T_Cells (Tcell 1.1) |
| 0.466206 | 0.69 | 0.402 | 1.69E-34 | 15 | Rbm39 | T_Cells (Tcell 1.1) |
| 0.573747 | 0.964 | 0.861 | 3.51E-34 | 15 | Rps11 | T_Cells (Tcell 1.1) |
| 0.477161 | 0.956 | 0.838 | 4.37E-34 | 15 | Rps3 | T_Cells (Tcell 1.1) |
| 0.339017 | 0.456 | 0.189 | 1.81E-33 | 15 | Prrc2c | T_Cells (Tcell 1.1) |
| 0.548447 | 0.915 | 0.792 | 2.85E-33 | 15 | Pfn1 | T_Cells (Tcell 1.1) |
| 0.382289 | 0.585 | 0.285 | 2.96E-33 | 15 | Rap1b | T_Cells (Tcell 1.1) |
| 0.431309 | 0.648 | 0.36 | 4.00E-33 | 15 | Ubl5 | T_Cells (Tcell 1.1) |
| 0.257381 | 0.286 | 0.089 | 4.34E-33 | 15 | Cwc15 | T_Cells (Tcell 1.1) |
| 0.461681 | 0.992 | 0.901 | 8.48E-33 | 15 | Rps24 | T_Cells (Tcell 1.1) |
| 0.405126 | 0.995 | 0.943 | 8.73E-33 | 15 | Rps27 | T_Cells (Tcell 1.1) |
| 0.332307 | 0.354 | 0.129 | 2.08E-32 | 15 | Ankrd11 | T_Cells (Tcell 1.1) |
| 0.254091 | 0.269 | 0.082 | 2.13E-32 | 15 | Tecr | T_Cells (Tcell 1.1) |
| 0.306115 | 0.374 | 0.142 | 2.73E-31 | 15 | Fis1 | T_Cells (Tcell 1.1) |
| 0.450567 | 0.973 | 0.872 | 1.77E-30 | 15 | Rpl18a | T_Cells (Tcell 1.1) |
| 0.459964 | 0.978 | 0.846 | 2.91E-30 | 15 | Rps5 | T_Cells (Tcell 1.1) |
| 0.438399 | 0.857 | 0.667 | 1.61E-29 | 15 | Rpl5 | T_Cells (Tcell 1.1) |
| 0.332664 | 0.308 | 0.108 | 2.97E-29 | 15 | Ddit4 | T_Cells (Tcell 1.1) |
| 0.262894 | 0.272 | 0.088 | 3.49E-29 | 15 | BC031181 | T_Cells (Tcell 1.1) |
| 0.439214 | 0.64 | 0.387 | 6.68E-29 | 15 | Sumo2 | T_Cells (Tcell 1.1) |
| 0.251169 | 0.288 | 0.097 | 7.00E-29 | 15 | Eif3m | T_Cells (Tcell 1.1) |
| 0.260833 | 0.288 | 0.097 | 8.41E-29 | 15 | Vps28 | T_Cells (Tcell 1.1) |
| 0.41872 | 0.978 | 0.884 | 1.65E-28 | 15 | Rps10 | T_Cells (Tcell 1.1) |
| 0.343442 | 0.451 | 0.202 | 1.67E-28 | 15 | Ptp4a2 | T_Cells (Tcell 1.1) |
| 0.262235 | 0.269 | 0.088 | 2.06E-28 | 15 | Pdcd10 | T_Cells (Tcell 1.1) |
| 0.265524 | 0.324 | 0.118 | 4.99E-28 | 15 | Gabarapl2 | T_Cells (Tcell 1.1) |
| 0.281574 | 0.363 | 0.142 | 5.05E-28 | 15 | Taf10 | T_Cells (Tcell 1.1) |
| 0.396739 | 0.484 | 0.237 | 5.56E-28 | 15 | Abracl | T_Cells (Tcell 1.1) |
| 0.342211 | 0.547 | 0.265 | 7.55E-28 | 15 | Lsp1 | T_Cells (Tcell 1.1) |
| 0.365935 | 0.516 | 0.26 | 1.02E-27 | 15 | Tma7 | T_Cells (Tcell 1.1) |
| 0.260847 | 0.354 | 0.136 | 2.15E-27 | 15 | Sf3b6 | T_Cells (Tcell 1.1) |
| 0.58543 | 0.761 | 0.517 | 2.32E-27 | 15 | H2afz | T_Cells (Tcell 1.1) |
| 0.375295 | 0.544 | 0.263 | 8.53E-27 | 15 | Tnfaip3 | T_Cells (Tcell 1.1) |
| 0.31329 | 0.36 | 0.145 | 1.43E-26 | 15 | Kmt2e | T_Cells (Tcell 1.1) |
| 0.468808 | 0.945 | 0.832 | 1.84E-25 | 15 | Rpl30 | T_Cells (Tcell 1.1) |
| 0.256906 | 0.299 | 0.11 | 4.64E-25 | 15 | Diaph1 | T_Cells (Tcell 1.1) |
| 0.441578 | 0.945 | 0.839 | 5.30E-25 | 15 | Ppia | T_Cells (Tcell 1.1) |
| 0.474879 | 0.462 | 0.241 | 8.80E-25 | 15 | Tmem176b | T_Cells (Tcell 1.1) |
| 0.295783 | 0.42 | 0.189 | 1.84E-24 | 15 | Paip2 | T_Cells (Tcell 1.1) |
| 0.370854 | 0.984 | 0.869 | 2.24E-24 | 15 | Rpl9 | T_Cells (Tcell 1.1) |
| 0.258571 | 0.253 | 0.087 | 5.10E-24 | 15 | Nsd3 | T_Cells (Tcell 1.1) |
| 0.383275 | 0.992 | 0.928 | 1.09E-23 | 15 | Eef1a1 | T_Cells (Tcell 1.1) |
| 0.27716 | 0.245 | 0.083 | 1.30E-23 | 15 | Got1 | T_Cells (Tcell 1.1) |
| 0.465 | 0.973 | 0.875 | 2.20E-23 | 15 | Rplp1 | T_Cells (Tcell 1.1) |
| 0.299889 | 0.396 | 0.177 | 6.14E-23 | 15 | Clk1 | T_Cells (Tcell 1.1) |
| 0.316966 | 0.464 | 0.232 | 6.87E-23 | 15 | Edf1 | T_Cells (Tcell 1.1) |
| 0.314391 | 0.453 | 0.22 | 7.13E-23 | 15 | Ube2b | T_Cells (Tcell 1.1) |
| 0.348276 | 0.253 | 0.09 | 9.47E-23 | 15 | Neurl3 | T_Cells (Tcell 1.1) |
| 0.253815 | 0.467 | 0.219 | 1.79E-21 | 15 | Ptprc | T_Cells (Tcell 1.1) |
| 0.253235 | 0.324 | 0.134 | 2.19E-21 | 15 | Ssb | T_Cells (Tcell 1.1) |
| 0.29732 | 0.525 | 0.277 | 2.92E-21 | 15 | Tpm3 | T_Cells (Tcell 1.1) |
| 0.327293 | 0.393 | 0.186 | 2.93E-21 | 15 | Srrm2 | T_Cells (Tcell 1.1) |
| 0.408224 | 0.816 | 0.645 | 5.01E-21 | 15 | Ddx5 | T_Cells (Tcell 1.1) |
| 0.258144 | 0.368 | 0.163 | 9.37E-21 | 15 | Jak1 | T_Cells (Tcell 1.1) |
| 0.347118 | 0.492 | 0.275 | 9.42E-21 | 15 | Ppp1ca | T_Cells (Tcell 1.1) |
| 0.266907 | 0.33 | 0.139 | 9.94E-21 | 15 | Luc7l2 | T_Cells (Tcell 1.1) |
| 0.338183 | 0.571 | 0.336 | 1.51E-20 | 15 | Cox5a | T_Cells (Tcell 1.1) |
| 0.353686 | 0.956 | 0.853 | 1.55E-20 | 15 | Rpl8 | T_Cells (Tcell 1.1) |
| 0.334774 | 0.662 | 0.419 | 3.98E-20 | 15 | Map1lc3b | T_Cells (Tcell 1.1) |
| 0.309873 | 0.374 | 0.173 | 5.53E-20 | 15 | Ndufa1 | T_Cells (Tcell 1.1) |
| 0.375414 | 0.973 | 0.91 | 9.35E-20 | 15 | Rps16 | T_Cells (Tcell 1.1) |
| 0.363529 | 0.973 | 0.902 | 2.07E-19 | 15 | Rpl38 | T_Cells (Tcell 1.1) |
| 0.306981 | 0.473 | 0.253 | 2.30E-19 | 15 | Tomm20 | T_Cells (Tcell 1.1) |
| 0.466142 | 0.701 | 0.526 | 3.99E-19 | 15 | Tagln2 | T_Cells (Tcell 1.1) |
| 0.383472 | 0.975 | 0.856 | 4.59E-19 | 15 | Rps15a | T_Cells (Tcell 1.1) |
| 0.355393 | 0.94 | 0.855 | 4.60E-19 | 15 | Rpl18 | T_Cells (Tcell 1.1) |
| 0.355455 | 0.604 | 0.388 | 6.96E-19 | 15 | Atp5d | T_Cells (Tcell 1.1) |
| 0.384543 | 0.83 | 0.69 | 1.09E-18 | 15 | Cfl1 | T_Cells (Tcell 1.1) |
| 0.354323 | 0.558 | 0.334 | 1.48E-18 | 15 | Ldha | T_Cells (Tcell 1.1) |
| 0.378411 | 0.951 | 0.85 | 1.48E-18 | 15 | Rpl32 | T_Cells (Tcell 1.1) |
| 0.360843 | 0.945 | 0.832 | 1.69E-18 | 15 | Rpl21 | T_Cells (Tcell 1.1) |
| 0.26579 | 0.36 | 0.167 | 1.90E-18 | 15 | Stat3 | T_Cells (Tcell 1.1) |
| 0.377273 | 0.89 | 0.759 | 1.92E-18 | 15 | Rack1 | T_Cells (Tcell 1.1) |
| 0.319196 | 1 | 0.942 | 1.92E-18 | 15 | mt-Co1 | T_Cells (Tcell 1.1) |
| 0.394657 | 0.835 | 0.712 | 2.33E-18 | 15 | Rps6 | T_Cells (Tcell 1.1) |
| 0.251504 | 0.269 | 0.109 | 2.48E-18 | 15 | Zc3h15 | T_Cells (Tcell 1.1) |
| 0.374959 | 0.898 | 0.764 | 5.66E-18 | 15 | Rpl3 | T_Cells (Tcell 1.1) |
| 0.334113 | 0.53 | 0.316 | 7.31E-18 | 15 | Cd47 | T_Cells (Tcell 1.1) |
| 0.355396 | 0.613 | 0.399 | 1.30E-17 | 15 | Snrpg | T_Cells (Tcell 1.1) |
| 0.262287 | 0.327 | 0.149 | 3.13E-17 | 15 | Jpt1 | T_Cells (Tcell 1.1) |
| 0.26653 | 0.288 | 0.123 | 3.21E-17 | 15 | Dusp5 | T_Cells (Tcell 1.1) |
| 0.287889 | 0.992 | 0.973 | 3.28E-17 | 15 | Rps29 | T_Cells (Tcell 1.1) |
| 0.410455 | 0.94 | 0.865 | 3.48E-17 | 15 | Ubb | T_Cells (Tcell 1.1) |
| 0.335763 | 0.959 | 0.842 | 7.09E-17 | 15 | Rplp2 | T_Cells (Tcell 1.1) |
| 0.291705 | 0.632 | 0.403 | 1.68E-16 | 15 | Tspo | T_Cells (Tcell 1.1) |
| 0.377207 | 0.962 | 0.872 | 2.29E-16 | 15 | Rpl34 | T_Cells (Tcell 1.1) |
| 0.530096 | 0.887 | 0.771 | 2.49E-16 | 15 | Crip1 | T_Cells (Tcell 1.1) |
| 0.310023 | 0.604 | 0.397 | 4.78E-16 | 15 | Eif3f | T_Cells (Tcell 1.1) |
| 0.275692 | 0.599 | 0.365 | 7.62E-16 | 15 | Pnrc1 | T_Cells (Tcell 1.1) |
| 0.317743 | 0.937 | 0.815 | 1.01E-15 | 15 | Rpl7 | T_Cells (Tcell 1.1) |
| 0.368615 | 0.981 | 0.858 | 1.03E-15 | 15 | Rpsa | T_Cells (Tcell 1.1) |
| 0.291411 | 0.453 | 0.254 | 1.62E-15 | 15 | Hnrnpf | T_Cells (Tcell 1.1) |
| 0.341986 | 0.997 | 0.924 | 1.71E-15 | 15 | mt-Atp6 | T_Cells (Tcell 1.1) |
| 0.321181 | 0.953 | 0.862 | 4.25E-15 | 15 | Rps23 | T_Cells (Tcell 1.1) |
| 0.28087 | 0.522 | 0.307 | 8.37E-15 | 15 | Ywhaz | T_Cells (Tcell 1.1) |
| 0.329659 | 0.995 | 0.927 | 1.10E-14 | 15 | mt-Co3 | T_Cells (Tcell 1.1) |
| 0.303285 | 0.995 | 0.897 | 2.28E-14 | 15 | Rpl13 | T_Cells (Tcell 1.1) |
| 0.28557 | 0.404 | 0.219 | 2.66E-14 | 15 | Anp32b | T_Cells (Tcell 1.1) |
| 0.322627 | 0.97 | 0.845 | 3.39E-14 | 15 | Rps4x | T_Cells (Tcell 1.1) |
| 0.256194 | 0.25 | 0.108 | 4.50E-14 | 15 | Rgs10 | T_Cells (Tcell 1.1) |
| 0.318748 | 0.764 | 0.656 | 4.56E-14 | 15 | Btf3 | T_Cells (Tcell 1.1) |
| 0.294475 | 0.582 | 0.382 | 4.63E-14 | 15 | Serbp1 | T_Cells (Tcell 1.1) |
| 0.295384 | 0.995 | 0.926 | 7.09E-14 | 15 | mt-Co2 | T_Cells (Tcell 1.1) |
| 0.296681 | 0.97 | 0.884 | 1.05E-13 | 15 | Rpl27a | T_Cells (Tcell 1.1) |
| 0.359306 | 0.937 | 0.731 | 1.17E-13 | 15 | Tmsb10 | T_Cells (Tcell 1.1) |
| 0.265674 | 0.374 | 0.198 | 1.66E-13 | 15 | Slc3a2 | T_Cells (Tcell 1.1) |
| 0.272135 | 0.418 | 0.229 | 1.69E-13 | 15 | Nfkb1 | T_Cells (Tcell 1.1) |
| 0.354717 | 0.777 | 0.621 | 1.80E-13 | 15 | Rbm3 | T_Cells (Tcell 1.1) |
| 0.292635 | 0.448 | 0.25 | 2.18E-13 | 15 | Atp2b1 | T_Cells (Tcell 1.1) |
| 0.255755 | 0.5 | 0.292 | 3.44E-13 | 15 | Arpc5 | T_Cells (Tcell 1.1) |
| 0.254054 | 0.42 | 0.236 | 4.07E-13 | 15 | Srsf3 | T_Cells (Tcell 1.1) |
| 0.35216 | 0.94 | 0.838 | 5.48E-13 | 15 | Rps26 | T_Cells (Tcell 1.1) |
| 0.251231 | 0.629 | 0.416 | 2.75E-12 | 15 | Gadd45b | T_Cells (Tcell 1.1) |
| 0.320404 | 0.81 | 0.704 | 3.06E-10 | 15 | Naca | T_Cells (Tcell 1.1) |
| 0.368727 | 0.505 | 0.352 | 5.47E-10 | 15 | Xist | T_Cells (Tcell 1.1) |
| 0.274966 | 0.519 | 0.351 | 6.74E-10 | 15 | Atp5md | T_Cells (Tcell 1.1) |
| 0.284873 | 0.901 | 0.828 | 1.84E-09 | 15 | Rpl10 | T_Cells (Tcell 1.1) |
| 0.281652 | 0.849 | 0.744 | 2.45E-09 | 15 | Rpl22 | T_Cells (Tcell 1.1) |
| 0.295389 | 0.986 | 0.909 | 4.77E-09 | 15 | Rps21 | T_Cells (Tcell 1.1) |
| 0.253606 | 0.566 | 0.404 | 5.90E-09 | 15 | Ndufa4 | T_Cells (Tcell 1.1) |
| 0.26379 | 0.967 | 0.86 | 1.51E-08 | 15 | Rpl6 | T_Cells (Tcell 1.1) |
| 0.267772 | 0.989 | 0.917 | 1.55E-08 | 15 | Rpl23 | T_Cells (Tcell 1.1) |
| 0.254613 | 0.657 | 0.53 | 3.08E-08 | 15 | Uqcrh | T_Cells (Tcell 1.1) |
| 0.253054 | 0.621 | 0.484 | 7.71E-08 | 15 | Atp5h | T_Cells (Tcell 1.1) |
| 0.261269 | 0.714 | 0.599 | 9.32E-08 | 15 | Rpl4 | T_Cells (Tcell 1.1) |
| 0.264521 | 0.951 | 0.824 | 1.26E-07 | 15 | Rpl15 | T_Cells (Tcell 1.1) |
| 0.2587 | 0.385 | 0.238 | 1.28E-07 | 15 | Zfp36l2 | T_Cells (Tcell 1.1) |
| 0.273157 | 0.915 | 0.896 | 4.97E-07 | 15 | H3f3b | T_Cells (Tcell 1.1) |
| 0.271116 | 0.962 | 0.906 | 2.85E-05 | 15 | Rpl39 | T_Cells (Tcell 1.1) |
| 0.318181 | 0.176 | 0.096 | 0.001811 | 15 | Lmo4 | T_Cells (Tcell 1.1) |
| 0.262419 | 0.758 | 0.675 | 0.002074 | 15 | Jund | T_Cells (Tcell 1.1) |
| 1.322382 | 0.735 | 0.096 | 0 | 16 | Vps37b | T_Cells (Tcell 1.2) |
| 1.28638 | 0.642 | 0.021 | 0 | 16 | Trbc2 | T_Cells (Tcell 1.2) |
| 1.018872 | 0.603 | 0.039 | 0 | 16 | Satb1 | T_Cells (Tcell 1.2) |
| 1.004445 | 0.552 | 0.039 | 0 | 16 | Ccr7 | T_Cells (Tcell 1.2) |
| 0.867079 | 0.358 | 0.014 | 0 | 16 | Trbc1 | T_Cells (Tcell 1.2) |
| 0.785279 | 0.445 | 0.029 | 0 | 16 | Ramp3 | T_Cells (Tcell 1.2) |
| 0.7754 | 0.493 | 0.017 | 0 | 16 | Gimap6 | T_Cells (Tcell 1.2) |
| 0.760013 | 0.53 | 0.032 | 0 | 16 | Cd3d | T_Cells (Tcell 1.2) |
| 0.676587 | 0.47 | 0.025 | 0 | 16 | Cd3g | T_Cells (Tcell 1.2) |
| 0.662589 | 0.428 | 0.007 | 0 | 16 | Gimap3 | T_Cells (Tcell 1.2) |
| 0.599938 | 0.394 | 0.019 | 0 | 16 | Gramd3 | T_Cells (Tcell 1.2) |
| 0.591475 | 0.273 | 0.003 | 0 | 16 | Ms4a4b | T_Cells (Tcell 1.2) |
| 0.55616 | 0.369 | 0.012 | 0 | 16 | Trac | T_Cells (Tcell 1.2) |
| 0.547447 | 0.332 | 0.002 | 0 | 16 | Lef1 | T_Cells (Tcell 1.2) |
| 0.545838 | 0.358 | 0.005 | 0 | 16 | Gimap4 | T_Cells (Tcell 1.2) |
| 0.526952 | 0.372 | 0.019 | 0 | 16 | Cd3e | T_Cells (Tcell 1.2) |
| 0.522786 | 0.346 | 0.017 | 0 | 16 | Cd2 | T_Cells (Tcell 1.2) |
| 0.515571 | 0.349 | 0.014 | 0 | 16 | Gimap1 | T_Cells (Tcell 1.2) |
| 0.489393 | 0.321 | 0.005 | 0 | 16 | Cd27 | T_Cells (Tcell 1.2) |
| 0.433303 | 0.293 | 0.005 | 0 | 16 | Itk | T_Cells (Tcell 1.2) |
| 0.399798 | 0.276 | 0.01 | 0 | 16 | Tnfrsf18 | T_Cells (Tcell 1.2) |
| 0.395474 | 0.256 | 0.004 | 0 | 16 | Txk | T_Cells (Tcell 1.2) |
| 0.35745 | 0.256 | 0.008 | 0 | 16 | Skap1 | T_Cells (Tcell 1.2) |
| 0.347382 | 0.22 | 0.005 | 0 | 16 | Cd28 | T_Cells (Tcell 1.2) |
| 0.335497 | 0.223 | 0.006 | 0 | 16 | Bcl2 | T_Cells (Tcell 1.2) |
| 0.252808 | 0.166 | 0.002 | 0 | 16 | Gimap7 | T_Cells (Tcell 1.2) |
| 0.320553 | 0.206 | 0.006 | 2.60E-298 | 16 | Grap2 | T_Cells (Tcell 1.2) |
| 0.385902 | 0.282 | 0.013 | 6.59E-293 | 16 | Lat | T_Cells (Tcell 1.2) |
| 0.573259 | 0.366 | 0.024 | 2.11E-284 | 16 | S1pr1 | T_Cells (Tcell 1.2) |
| 0.298205 | 0.189 | 0.005 | 9.02E-280 | 16 | Trib2 | T_Cells (Tcell 1.2) |
| 0.295452 | 0.203 | 0.006 | 5.15E-270 | 16 | Lck | T_Cells (Tcell 1.2) |
| 0.387691 | 0.29 | 0.016 | 9.60E-255 | 16 | Prkca | T_Cells (Tcell 1.2) |
| 0.718701 | 0.515 | 0.058 | 1.91E-253 | 16 | Saraf | T_Cells (Tcell 1.2) |
| 0.356051 | 0.239 | 0.011 | 7.28E-249 | 16 | Sh2d2a | T_Cells (Tcell 1.2) |
| 0.40644 | 0.285 | 0.016 | 4.11E-244 | 16 | Dusp10 | T_Cells (Tcell 1.2) |
| 0.436382 | 0.282 | 0.018 | 2.40E-221 | 16 | Ablim1 | T_Cells (Tcell 1.2) |
| 0.345617 | 0.239 | 0.013 | 5.89E-211 | 16 | Ptpn22 | T_Cells (Tcell 1.2) |
| 0.361805 | 0.11 | 0.002 | 2.73E-198 | 16 | Nkg7 | T_Cells (Tcell 1.2) |
| 0.956362 | 0.676 | 0.14 | 1.80E-185 | 16 | Ifngr1 | T_Cells (Tcell 1.2) |
| 0.459196 | 0.355 | 0.038 | 7.79E-174 | 16 | Crlf3 | T_Cells (Tcell 1.2) |
| 1.045127 | 0.676 | 0.15 | 2.38E-173 | 16 | Emb | T_Cells (Tcell 1.2) |
| 0.56871 | 0.411 | 0.051 | 2.55E-173 | 16 | Il7r | T_Cells (Tcell 1.2) |
| 1.454924 | 1 | 0.973 | 3.56E-168 | 16 | Rps29 | T_Cells (Tcell 1.2) |
| 1.482268 | 1 | 0.943 | 2.07E-162 | 16 | Rps27 | T_Cells (Tcell 1.2) |
| 1.360305 | 0.994 | 0.882 | 4.79E-162 | 16 | Rpl19 | T_Cells (Tcell 1.2) |
| 0.638235 | 0.493 | 0.079 | 4.81E-162 | 16 | Ets1 | T_Cells (Tcell 1.2) |
| 1.489321 | 1 | 0.901 | 5.30E-162 | 16 | Rps24 | T_Cells (Tcell 1.2) |
| 1.321022 | 0.994 | 0.91 | 5.12E-160 | 16 | Rps16 | T_Cells (Tcell 1.2) |
| 1.433149 | 0.986 | 0.852 | 3.55E-159 | 16 | Rpl8 | T_Cells (Tcell 1.2) |
| 1.295688 | 0.992 | 0.849 | 7.09E-156 | 16 | Rps13 | T_Cells (Tcell 1.2) |
| 1.479824 | 0.977 | 0.805 | 1.75E-154 | 16 | Rpl13a | T_Cells (Tcell 1.2) |
| 1.414086 | 0.992 | 0.837 | 2.88E-153 | 16 | Rps3 | T_Cells (Tcell 1.2) |
| 1.675135 | 0.963 | 0.763 | 2.76E-151 | 16 | Rpl12 | T_Cells (Tcell 1.2) |
| 1.342289 | 0.994 | 0.869 | 1.89E-150 | 16 | Rpl9 | T_Cells (Tcell 1.2) |
| 1.362306 | 0.992 | 0.846 | 2.29E-149 | 16 | Rps5 | T_Cells (Tcell 1.2) |
| 1.272998 | 0.992 | 0.884 | 2.10E-147 | 16 | Rps10 | T_Cells (Tcell 1.2) |
| 1.528163 | 0.989 | 0.875 | 2.33E-146 | 16 | Rplp1 | T_Cells (Tcell 1.2) |
| 1.288421 | 0.961 | 0.709 | 1.41E-144 | 16 | Rps6 | T_Cells (Tcell 1.2) |
| 1.321845 | 0.989 | 0.845 | 1.66E-143 | 16 | Rps4x | T_Cells (Tcell 1.2) |
| 1.114358 | 0.989 | 0.851 | 5.56E-143 | 16 | Rps14 | T_Cells (Tcell 1.2) |
| 1.155149 | 0.989 | 0.854 | 9.34E-143 | 16 | Rpl18 | T_Cells (Tcell 1.2) |
| 1.462627 | 0.983 | 0.839 | 1.22E-142 | 16 | Rps7 | T_Cells (Tcell 1.2) |
| 1.469962 | 0.935 | 0.666 | 2.54E-142 | 16 | Rpl5 | T_Cells (Tcell 1.2) |
| 0.366268 | 0.265 | 0.025 | 4.00E-142 | 16 | Il21r | T_Cells (Tcell 1.2) |
| 1.209892 | 0.994 | 0.871 | 3.99E-141 | 16 | Rpl18a | T_Cells (Tcell 1.2) |
| 1.287044 | 1 | 0.897 | 6.63E-141 | 16 | Rpl13 | T_Cells (Tcell 1.2) |
| 0.469972 | 0.38 | 0.053 | 7.92E-141 | 16 | Tut4 | T_Cells (Tcell 1.2) |
| 1.087941 | 0.994 | 0.95 | 3.10E-139 | 16 | Rps9 | T_Cells (Tcell 1.2) |
| 1.423474 | 0.994 | 0.98 | 2.86E-134 | 16 | Tpt1 | T_Cells (Tcell 1.2) |
| 1.241106 | 0.986 | 0.839 | 7.21E-134 | 16 | Rpl17 | T_Cells (Tcell 1.2) |
| 1.323573 | 0.997 | 0.857 | 6.68E-132 | 16 | Rpsa | T_Cells (Tcell 1.2) |
| 0.28707 | 0.228 | 0.02 | 1.11E-130 | 16 | Cblb | T_Cells (Tcell 1.2) |
| 1.136843 | 0.997 | 0.909 | 3.87E-130 | 16 | Rps28 | T_Cells (Tcell 1.2) |
| 0.299052 | 0.223 | 0.02 | 2.06E-128 | 16 | Bzw2 | T_Cells (Tcell 1.2) |
| 1.147742 | 0.977 | 0.757 | 1.09E-127 | 16 | Rack1 | T_Cells (Tcell 1.2) |
| 0.447404 | 0.361 | 0.053 | 2.20E-125 | 16 | Limd2 | T_Cells (Tcell 1.2) |
| 1.068679 | 1 | 0.884 | 2.35E-125 | 16 | Rps3a1 | T_Cells (Tcell 1.2) |
| 1.041685 | 0.994 | 0.86 | 1.09E-122 | 16 | Rps11 | T_Cells (Tcell 1.2) |
| 1.130442 | 0.972 | 0.814 | 2.76E-122 | 16 | Rpl7 | T_Cells (Tcell 1.2) |
| 1.097891 | 0.98 | 0.831 | 3.13E-122 | 16 | Rpl21 | T_Cells (Tcell 1.2) |
| 0.93674 | 0.997 | 0.969 | 1.18E-121 | 16 | Fau | T_Cells (Tcell 1.2) |
| 1.051591 | 0.994 | 0.908 | 5.31E-121 | 16 | Rps21 | T_Cells (Tcell 1.2) |
| 1.207712 | 0.963 | 0.762 | 2.06E-120 | 16 | Rpl3 | T_Cells (Tcell 1.2) |
| 1.02159 | 0.997 | 0.916 | 1.99E-119 | 16 | Rpl23 | T_Cells (Tcell 1.2) |
| 1.089377 | 0.994 | 0.856 | 3.42E-119 | 16 | Rps15a | T_Cells (Tcell 1.2) |
| 0.996071 | 0.992 | 0.883 | 9.86E-117 | 16 | Rpl27a | T_Cells (Tcell 1.2) |
| 1.077853 | 0.989 | 0.849 | 1.64E-115 | 16 | Rpl32 | T_Cells (Tcell 1.2) |
| 1.133917 | 0.938 | 0.52 | 2.17E-115 | 16 | H2-K1 | T_Cells (Tcell 1.2) |
| 0.915729 | 0.975 | 0.795 | 2.20E-112 | 16 | Rpl27 | T_Cells (Tcell 1.2) |
| 0.990781 | 0.975 | 0.842 | 1.12E-111 | 16 | Rplp2 | T_Cells (Tcell 1.2) |
| 0.941799 | 0.994 | 0.928 | 2.59E-111 | 16 | Eef1a1 | T_Cells (Tcell 1.2) |
| 0.964492 | 0.986 | 0.842 | 2.36E-110 | 16 | Rpl11 | T_Cells (Tcell 1.2) |
| 0.37883 | 0.293 | 0.041 | 2.98E-107 | 16 | Zeb1 | T_Cells (Tcell 1.2) |
| 0.943149 | 0.986 | 0.862 | 4.64E-107 | 16 | Rps23 | T_Cells (Tcell 1.2) |
| 0.467698 | 0.363 | 0.062 | 2.94E-106 | 16 | H2-Q7 | T_Cells (Tcell 1.2) |
| 0.977731 | 0.986 | 0.832 | 5.66E-106 | 16 | Rpl30 | T_Cells (Tcell 1.2) |
| 0.899588 | 0.989 | 0.912 | 2.45E-105 | 16 | Rps27a | T_Cells (Tcell 1.2) |
| 1.123278 | 0.975 | 0.845 | 2.12E-104 | 16 | Rps19 | T_Cells (Tcell 1.2) |
| 0.964114 | 0.983 | 0.859 | 9.04E-104 | 16 | Rpl6 | T_Cells (Tcell 1.2) |
| 0.267054 | 0.214 | 0.022 | 1.51E-103 | 16 | 01-Sep | T_Cells (Tcell 1.2) |
| 0.557375 | 0.197 | 0.02 | 2.83E-100 | 16 | AW112010 | T_Cells (Tcell 1.2) |
| 0.416993 | 0.344 | 0.058 | 5.08E-100 | 16 | Zc3hav1 | T_Cells (Tcell 1.2) |
| 0.330219 | 0.279 | 0.04 | 4.68E-98 | 16 | Rapgef6 | T_Cells (Tcell 1.2) |
| 0.837729 | 0.994 | 0.902 | 2.69E-96 | 16 | Rpl38 | T_Cells (Tcell 1.2) |
| 0.28045 | 0.211 | 0.024 | 9.32E-96 | 16 | P2ry10 | T_Cells (Tcell 1.2) |
| 1.004498 | 0.992 | 0.871 | 3.01E-95 | 16 | Rps20 | T_Cells (Tcell 1.2) |
| 0.30655 | 0.211 | 0.024 | 3.87E-95 | 16 | Tmem64 | T_Cells (Tcell 1.2) |
| 0.990372 | 0.994 | 0.868 | 4.12E-95 | 16 | Rplp0 | T_Cells (Tcell 1.2) |
| 0.5614 | 0.496 | 0.121 | 4.83E-95 | 16 | Fyb | T_Cells (Tcell 1.2) |
| 1.033992 | 0.972 | 0.648 | 9.93E-95 | 16 | H2-D1 | T_Cells (Tcell 1.2) |
| 0.958375 | 0.963 | 0.788 | 3.41E-94 | 16 | Rpl10a | T_Cells (Tcell 1.2) |
| 0.853794 | 0.98 | 0.838 | 1.26E-91 | 16 | Rpl26 | T_Cells (Tcell 1.2) |
| 0.266836 | 0.194 | 0.021 | 3.26E-90 | 16 | Gtf2i | T_Cells (Tcell 1.2) |
| 0.556821 | 0.439 | 0.099 | 6.14E-90 | 16 | Selplg | T_Cells (Tcell 1.2) |
| 0.33975 | 0.299 | 0.049 | 1.43E-88 | 16 | Pdcd4 | T_Cells (Tcell 1.2) |
| 0.940189 | 0.927 | 0.636 | 9.71E-87 | 16 | Rps18 | T_Cells (Tcell 1.2) |
| 0.858367 | 0.944 | 0.735 | 5.10E-86 | 16 | Rps15 | T_Cells (Tcell 1.2) |
| 0.413745 | 0.324 | 0.059 | 6.10E-85 | 16 | Hcst | T_Cells (Tcell 1.2) |
| 0.83428 | 0.966 | 0.826 | 7.94E-85 | 16 | Rpl10 | T_Cells (Tcell 1.2) |
| 0.415373 | 0.355 | 0.071 | 4.44E-83 | 16 | Smchd1 | T_Cells (Tcell 1.2) |
| 0.779096 | 0.992 | 0.892 | 1.14E-81 | 16 | Rpl35a | T_Cells (Tcell 1.2) |
| 0.792174 | 0.989 | 0.914 | 4.59E-81 | 16 | Rps8 | T_Cells (Tcell 1.2) |
| 0.831004 | 0.887 | 0.595 | 9.25E-81 | 16 | Rpl4 | T_Cells (Tcell 1.2) |
| 0.818878 | 0.963 | 0.824 | 5.25E-80 | 16 | Rpl15 | T_Cells (Tcell 1.2) |
| 0.627696 | 0.62 | 0.223 | 1.07E-78 | 16 | Cox7a2l | T_Cells (Tcell 1.2) |
| 0.317164 | 0.259 | 0.042 | 2.11E-77 | 16 | Arhgap45 | T_Cells (Tcell 1.2) |
| 0.545271 | 0.558 | 0.173 | 9.23E-77 | 16 | Mbnl1 | T_Cells (Tcell 1.2) |
| 0.476021 | 0.439 | 0.112 | 3.18E-76 | 16 | Ptpn18 | T_Cells (Tcell 1.2) |
| 0.330046 | 0.287 | 0.052 | 7.00E-76 | 16 | Emg1 | T_Cells (Tcell 1.2) |
| 0.505728 | 0.485 | 0.136 | 1.80E-74 | 16 | Psmb8 | T_Cells (Tcell 1.2) |
| 0.784773 | 0.977 | 0.855 | 1.52E-73 | 16 | Rpl36 | T_Cells (Tcell 1.2) |
| 0.868062 | 0.901 | 0.688 | 1.61E-73 | 16 | Eef1b2 | T_Cells (Tcell 1.2) |
| 0.310607 | 0.245 | 0.04 | 1.14E-72 | 16 | Vgll4 | T_Cells (Tcell 1.2) |
| 0.817174 | 0.972 | 0.837 | 1.70E-71 | 16 | Rps26 | T_Cells (Tcell 1.2) |
| 0.335227 | 0.282 | 0.052 | 2.99E-71 | 16 | 1810026B05Rik | T_Cells (Tcell 1.2) |
| 0.810377 | 0.918 | 0.751 | 3.44E-71 | 16 | Rpl14 | T_Cells (Tcell 1.2) |
| 0.633954 | 0.586 | 0.213 | 2.94E-70 | 16 | Prdx6 | T_Cells (Tcell 1.2) |
| 0.718293 | 0.994 | 0.905 | 3.77E-70 | 16 | Rpl39 | T_Cells (Tcell 1.2) |
| 0.354484 | 0.299 | 0.06 | 1.69E-68 | 16 | Ssh2 | T_Cells (Tcell 1.2) |
| 0.463309 | 0.366 | 0.087 | 1.51E-67 | 16 | Neurl3 | T_Cells (Tcell 1.2) |
| 0.706791 | 0.941 | 0.752 | 6.54E-67 | 16 | Rps25 | T_Cells (Tcell 1.2) |
| 0.717914 | 0.769 | 0.356 | 3.41E-66 | 16 | Sub1 | T_Cells (Tcell 1.2) |
| 0.382463 | 0.293 | 0.061 | 1.20E-64 | 16 | Ms4a6b | T_Cells (Tcell 1.2) |
| 0.447823 | 0.389 | 0.102 | 1.48E-64 | 16 | Atp1b3 | T_Cells (Tcell 1.2) |
| 0.539927 | 0.51 | 0.168 | 2.23E-64 | 16 | Shisa5 | T_Cells (Tcell 1.2) |
| 0.492152 | 0.392 | 0.104 | 2.32E-64 | 16 | Rgs10 | T_Cells (Tcell 1.2) |
| 0.669007 | 0.997 | 0.927 | 1.30E-63 | 16 | Rpl37a | T_Cells (Tcell 1.2) |
| 0.673606 | 0.997 | 0.871 | 2.24E-62 | 16 | Rpl34 | T_Cells (Tcell 1.2) |
| 0.449138 | 0.49 | 0.154 | 4.67E-60 | 16 | Rac2 | T_Cells (Tcell 1.2) |
| 0.661404 | 0.989 | 0.908 | 5.91E-60 | 16 | Rpl37 | T_Cells (Tcell 1.2) |
| 0.771079 | 0.961 | 0.784 | 5.02E-59 | 16 | Rps2 | T_Cells (Tcell 1.2) |
| 0.337179 | 0.293 | 0.065 | 5.21E-58 | 16 | Elf1 | T_Cells (Tcell 1.2) |
| 1.318162 | 0.101 | 0.009 | 1.20E-57 | 16 | Ccl5 | T_Cells (Tcell 1.2) |
| 0.671095 | 0.761 | 0.393 | 1.80E-57 | 16 | Gm10260 | T_Cells (Tcell 1.2) |
| 0.643284 | 0.873 | 0.683 | 1.67E-56 | 16 | Rpl23a | T_Cells (Tcell 1.2) |
| 0.2524 | 0.194 | 0.032 | 1.56E-55 | 16 | Stk4 | T_Cells (Tcell 1.2) |
| 0.268144 | 0.237 | 0.047 | 6.98E-54 | 16 | Orai1 | T_Cells (Tcell 1.2) |
| 0.330171 | 0.307 | 0.073 | 1.22E-53 | 16 | Ltb | T_Cells (Tcell 1.2) |
| 0.575357 | 0.899 | 0.702 | 2.81E-50 | 16 | Naca | T_Cells (Tcell 1.2) |
| 0.410193 | 0.166 | 0.026 | 6.38E-50 | 16 | Ctla2a | T_Cells (Tcell 1.2) |
| 0.364623 | 0.417 | 0.131 | 1.06E-49 | 16 | Supt4a | T_Cells (Tcell 1.2) |
| 0.616076 | 0.98 | 0.861 | 1.42E-49 | 16 | Rpl28 | T_Cells (Tcell 1.2) |
| 0.429499 | 0.459 | 0.158 | 2.40E-49 | 16 | Srsf7 | T_Cells (Tcell 1.2) |
| 0.619865 | 0.899 | 0.69 | 4.14E-48 | 16 | Rpl36a | T_Cells (Tcell 1.2) |
| 0.330769 | 0.332 | 0.091 | 7.14E-48 | 16 | Fam107b | T_Cells (Tcell 1.2) |
| 0.461859 | 0.456 | 0.157 | 3.69E-47 | 16 | Cytip | T_Cells (Tcell 1.2) |
| 0.354838 | 0.335 | 0.096 | 1.51E-46 | 16 | Eif3m | T_Cells (Tcell 1.2) |
| 0.552744 | 0.961 | 0.848 | 5.52E-46 | 16 | Rpl24 | T_Cells (Tcell 1.2) |
| 0.631565 | 0.986 | 0.888 | 6.63E-46 | 16 | Rps12 | T_Cells (Tcell 1.2) |
| 0.475351 | 0.569 | 0.242 | 1.04E-43 | 16 | Arhgdib | T_Cells (Tcell 1.2) |
| 0.501562 | 0.625 | 0.312 | 1.27E-42 | 16 | Eif3h | T_Cells (Tcell 1.2) |
| 0.344822 | 0.335 | 0.101 | 5.20E-42 | 16 | Smad7 | T_Cells (Tcell 1.2) |
| 0.599521 | 0.887 | 0.744 | 6.21E-42 | 16 | Rpl22 | T_Cells (Tcell 1.2) |
| 0.304044 | 0.313 | 0.09 | 1.49E-41 | 16 | Syf2 | T_Cells (Tcell 1.2) |
| 0.322637 | 0.299 | 0.084 | 2.10E-41 | 16 | Ppp1cc | T_Cells (Tcell 1.2) |
| 0.278954 | 0.259 | 0.065 | 2.42E-41 | 16 | Gpr132 | T_Cells (Tcell 1.2) |
| 0.339639 | 0.414 | 0.147 | 8.11E-40 | 16 | Jpt1 | T_Cells (Tcell 1.2) |
| 0.516416 | 0.986 | 0.925 | 1.15E-38 | 16 | mt-Atp6 | T_Cells (Tcell 1.2) |
| 0.259642 | 0.242 | 0.062 | 1.89E-37 | 16 | Chd2 | T_Cells (Tcell 1.2) |
| 0.614539 | 0.859 | 0.669 | 2.37E-37 | 16 | mt-Nd2 | T_Cells (Tcell 1.2) |
| 0.385069 | 0.527 | 0.216 | 1.03E-36 | 16 | Cd53 | T_Cells (Tcell 1.2) |
| 0.697932 | 0.758 | 0.543 | 1.26E-36 | 16 | Rpl29 | T_Cells (Tcell 1.2) |
| 0.363144 | 0.299 | 0.09 | 1.55E-36 | 16 | Crem | T_Cells (Tcell 1.2) |
| 0.298687 | 0.135 | 0.023 | 2.73E-36 | 16 | Ly6c2 | T_Cells (Tcell 1.2) |
| 0.348991 | 0.361 | 0.132 | 3.23E-33 | 16 | Sec11a | T_Cells (Tcell 1.2) |
| 0.418635 | 0.561 | 0.257 | 6.23E-33 | 16 | Coro1a | T_Cells (Tcell 1.2) |
| 0.453843 | 0.67 | 0.396 | 1.02E-32 | 16 | Eif3f | T_Cells (Tcell 1.2) |
| 0.534643 | 0.941 | 0.731 | 1.04E-32 | 16 | Tmsb10 | T_Cells (Tcell 1.2) |
| 0.317395 | 0.299 | 0.096 | 2.41E-32 | 16 | Ubald2 | T_Cells (Tcell 1.2) |
| 0.411348 | 0.53 | 0.253 | 2.46E-32 | 16 | Hnrnpf | T_Cells (Tcell 1.2) |
| 0.352054 | 0.439 | 0.176 | 7.01E-32 | 16 | Stk17b | T_Cells (Tcell 1.2) |
| 0.354693 | 0.515 | 0.23 | 7.51E-32 | 16 | Myl12b | T_Cells (Tcell 1.2) |
| 0.407925 | 0.586 | 0.303 | 7.73E-32 | 16 | Eef1d | T_Cells (Tcell 1.2) |
| 0.279587 | 0.259 | 0.077 | 7.93E-32 | 16 | Prpf4b | T_Cells (Tcell 1.2) |
| 0.439904 | 0.538 | 0.264 | 1.36E-31 | 16 | Nsa2 | T_Cells (Tcell 1.2) |
| 0.3706 | 0.403 | 0.162 | 3.74E-31 | 16 | Jak1 | T_Cells (Tcell 1.2) |
| 0.443751 | 0.924 | 0.656 | 4.49E-31 | 16 | B2m | T_Cells (Tcell 1.2) |
| 0.341229 | 0.423 | 0.174 | 5.27E-31 | 16 | Tle5 | T_Cells (Tcell 1.2) |
| 0.390172 | 0.476 | 0.215 | 5.47E-31 | 16 | Cnbp | T_Cells (Tcell 1.2) |
| 0.267345 | 0.276 | 0.086 | 8.17E-31 | 16 | Nsd3 | T_Cells (Tcell 1.2) |
| 0.442364 | 0.763 | 0.467 | 2.97E-30 | 16 | Arpc3 | T_Cells (Tcell 1.2) |
| 0.450891 | 0.907 | 0.75 | 1.33E-29 | 16 | Rpl7a | T_Cells (Tcell 1.2) |
| 0.329994 | 0.372 | 0.145 | 2.72E-29 | 16 | Dnajb6 | T_Cells (Tcell 1.2) |
| 0.314006 | 0.372 | 0.144 | 3.31E-29 | 16 | Psme1 | T_Cells (Tcell 1.2) |
| 0.3491 | 0.448 | 0.197 | 3.77E-29 | 16 | Eif3e | T_Cells (Tcell 1.2) |
| 0.49265 | 0.786 | 0.516 | 1.30E-28 | 16 | H2afz | T_Cells (Tcell 1.2) |
| 0.305692 | 0.341 | 0.129 | 2.12E-27 | 16 | Ankrd11 | T_Cells (Tcell 1.2) |
| 0.517926 | 0.797 | 0.59 | 2.15E-27 | 16 | Npm1 | T_Cells (Tcell 1.2) |
| 0.269887 | 0.304 | 0.111 | 9.90E-26 | 16 | H2afy | T_Cells (Tcell 1.2) |
| 0.340963 | 0.423 | 0.19 | 3.76E-25 | 16 | Prrc2c | T_Cells (Tcell 1.2) |
| 0.447955 | 0.941 | 0.797 | 9.63E-25 | 16 | Rpl35 | T_Cells (Tcell 1.2) |
| 0.306655 | 0.406 | 0.186 | 1.17E-22 | 16 | Srrm2 | T_Cells (Tcell 1.2) |
| 0.311417 | 0.27 | 0.098 | 1.24E-22 | 16 | Gdpd3 | T_Cells (Tcell 1.2) |
| 0.335746 | 0.541 | 0.276 | 4.01E-22 | 16 | Klf2 | T_Cells (Tcell 1.2) |
| 0.28672 | 0.392 | 0.177 | 2.72E-21 | 16 | Clk1 | T_Cells (Tcell 1.2) |
| 0.366073 | 0.792 | 0.656 | 2.08E-20 | 16 | Btf3 | T_Cells (Tcell 1.2) |
| 0.396025 | 0.963 | 0.838 | 2.31E-20 | 16 | Ppia | T_Cells (Tcell 1.2) |
| 0.3576 | 0.606 | 0.368 | 2.40E-20 | 16 | Eef1g | T_Cells (Tcell 1.2) |
| 0.326512 | 0.989 | 0.927 | 5.96E-20 | 16 | mt-Co3 | T_Cells (Tcell 1.2) |
| 0.288182 | 0.456 | 0.236 | 1.43E-18 | 16 | Hnrnpa1 | T_Cells (Tcell 1.2) |
| 0.310679 | 0.389 | 0.19 | 3.35E-18 | 16 | Paip2 | T_Cells (Tcell 1.2) |
| 0.301799 | 0.989 | 0.927 | 5.41E-18 | 16 | mt-Co2 | T_Cells (Tcell 1.2) |
| 0.369636 | 0.662 | 0.465 | 3.28E-17 | 16 | Pabpc1 | T_Cells (Tcell 1.2) |
| 0.368324 | 0.882 | 0.782 | 5.31E-17 | 16 | Oaz1 | T_Cells (Tcell 1.2) |
| 0.270128 | 0.304 | 0.134 | 8.16E-17 | 16 | Tpr | T_Cells (Tcell 1.2) |
| 0.288907 | 0.459 | 0.253 | 1.66E-16 | 16 | Tomm20 | T_Cells (Tcell 1.2) |
| 0.32255 | 0.434 | 0.224 | 7.98E-16 | 16 | Hmgb2 | T_Cells (Tcell 1.2) |
| 0.282916 | 0.437 | 0.235 | 1.12E-15 | 16 | Srsf3 | T_Cells (Tcell 1.2) |
| 0.313965 | 0.408 | 0.219 | 1.35E-15 | 16 | Hnrnpa0 | T_Cells (Tcell 1.2) |
| 0.290314 | 0.417 | 0.221 | 1.41E-15 | 16 | Ube2b | T_Cells (Tcell 1.2) |
| 0.303977 | 0.586 | 0.366 | 3.94E-15 | 16 | Pnrc1 | T_Cells (Tcell 1.2) |
| 0.322029 | 0.792 | 0.653 | 1.73E-14 | 16 | Rps17 | T_Cells (Tcell 1.2) |
| 0.275133 | 0.49 | 0.293 | 7.29E-14 | 16 | Psmb1 | T_Cells (Tcell 1.2) |
| 0.271726 | 0.932 | 0.896 | 2.52E-12 | 16 | H3f3b | T_Cells (Tcell 1.2) |
| 0.295141 | 0.732 | 0.568 | 5.80E-12 | 16 | Rpl36al | T_Cells (Tcell 1.2) |
| 0.310387 | 0.761 | 0.609 | 1.20E-11 | 16 | Eef2 | T_Cells (Tcell 1.2) |
| 0.269231 | 0.482 | 0.295 | 2.21E-11 | 16 | mt-Nd5 | T_Cells (Tcell 1.2) |
| 0.35229 | 0.71 | 0.578 | 1.02E-10 | 16 | Rpl31 | T_Cells (Tcell 1.2) |
| 0.288904 | 0.532 | 0.346 | 1.02E-10 | 16 | Ncl | T_Cells (Tcell 1.2) |
| 0.300008 | 0.127 | 0.042 | 4.88E-10 | 16 | Gm26917 | T_Cells (Tcell 1.2) |
| 0.262592 | 0.194 | 0.082 | 6.14E-10 | 16 | Rgs2 | T_Cells (Tcell 1.2) |
| 0.271087 | 0.513 | 0.335 | 9.32E-10 | 16 | Ldha | T_Cells (Tcell 1.2) |
| 0.310531 | 0.78 | 0.646 | 1.06E-09 | 16 | Ddx5 | T_Cells (Tcell 1.2) |
| 0.286935 | 0.561 | 0.401 | 2.25E-09 | 16 | Snrpg | T_Cells (Tcell 1.2) |
| 0.260939 | 0.575 | 0.405 | 3.01E-09 | 16 | Rbm39 | T_Cells (Tcell 1.2) |
| 0.285054 | 0.541 | 0.383 | 4.44E-09 | 16 | Serbp1 | T_Cells (Tcell 1.2) |
| 0.301923 | 0.654 | 0.53 | 1.80E-08 | 16 | Uqcrh | T_Cells (Tcell 1.2) |
| 0.269117 | 0.724 | 0.568 | 4.85E-08 | 16 | Rpl22l1 | T_Cells (Tcell 1.2) |
| 0.279751 | 0.634 | 0.485 | 3.30E-07 | 16 | Gas5 | T_Cells (Tcell 1.2) |
| 0.251554 | 0.93 | 0.842 | 3.85E-06 | 16 | Ptma | T_Cells (Tcell 1.2) |
| 2.193324 | 0.73 | 0.181 | 4.82E-177 | 17 | Spp1 | Myofibroblast (Myo 1.3) |
| 2.060077 | 0.938 | 0.348 | 1.28E-189 | 17 | Timp1 | Myofibroblast (Myo 1.3) |
| 1.248765 | 0.994 | 0.735 | 1.51E-122 | 17 | Lgals1 | Myofibroblast (Myo 1.3) |
| 1.106007 | 0.992 | 0.73 | 1.86E-94 | 17 | Tmsb10 | Myofibroblast (Myo 1.3) |
| 0.996213 | 0.42 | 0.138 | 1.79E-49 | 17 | Cxcl5 | Myofibroblast (Myo 1.3) |
| 0.994807 | 0.992 | 0.708 | 4.03E-131 | 17 | Nme2 | Myofibroblast (Myo 1.3) |
| 0.983008 | 0.969 | 0.496 | 1.92E-105 | 17 | Serpinh1 | Myofibroblast (Myo 1.3) |
| 0.978268 | 0.6 | 0.161 | 1.41E-109 | 17 | Tnc | Myofibroblast (Myo 1.3) |
| 0.944966 | 1 | 0.943 | 1.73E-125 | 17 | Rpl41 | Myofibroblast (Myo 1.3) |
| 0.934136 | 0.975 | 0.794 | 1.92E-66 | 17 | Gm10076 | Myofibroblast (Myo 1.3) |
| 0.90082 | 0.961 | 0.611 | 4.13E-115 | 17 | Sec61g | Myofibroblast (Myo 1.3) |
| 0.818586 | 0.952 | 0.496 | 2.54E-120 | 17 | Sec61b | Myofibroblast (Myo 1.3) |
| 0.813833 | 0.997 | 0.783 | 2.96E-95 | 17 | Rps2 | Myofibroblast (Myo 1.3) |
| 0.810825 | 0.659 | 0.2 | 7.68E-100 | 17 | Rbp1 | Myofibroblast (Myo 1.3) |
| 0.710611 | 0.983 | 0.647 | 2.37E-70 | 17 | Cd63 | Myofibroblast (Myo 1.3) |
| 0.710403 | 1 | 0.857 | 3.58E-83 | 17 | Rpsa | Myofibroblast (Myo 1.3) |
| 0.709178 | 0.882 | 0.463 | 7.41E-85 | 17 | Rrbp1 | Myofibroblast (Myo 1.3) |
| 0.702527 | 0.901 | 0.519 | 1.00E-76 | 17 | Hsp90b1 | Myofibroblast (Myo 1.3) |
| 0.683235 | 0.994 | 0.85 | 4.12E-43 | 17 | S100a6 | Myofibroblast (Myo 1.3) |
| 0.670763 | 0.732 | 0.216 | 3.23E-106 | 17 | Cthrc1 | Myofibroblast (Myo 1.3) |
| 0.665041 | 0.989 | 0.796 | 4.85E-63 | 17 | Rpl35 | Myofibroblast (Myo 1.3) |
| 0.657269 | 0.958 | 0.649 | 7.27E-81 | 17 | Rps17 | Myofibroblast (Myo 1.3) |
| 0.624816 | 0.786 | 0.329 | 1.28E-69 | 17 | Csrp2 | Myofibroblast (Myo 1.3) |
| 0.618858 | 0.665 | 0.219 | 1.22E-83 | 17 | Col5a3 | Myofibroblast (Myo 1.3) |
| 0.617504 | 0.451 | 0.166 | 4.47E-42 | 17 | Crabp1 | Myofibroblast (Myo 1.3) |
| 0.613395 | 0.823 | 0.313 | 3.50E-92 | 17 | Kdelr2 | Myofibroblast (Myo 1.3) |
| 0.610144 | 0.949 | 0.586 | 9.69E-73 | 17 | Npm1 | Myofibroblast (Myo 1.3) |
| 0.604677 | 0.825 | 0.285 | 1.63E-108 | 17 | Ostc | Myofibroblast (Myo 1.3) |
| 0.601974 | 0.927 | 0.629 | 3.14E-67 | 17 | Ybx1 | Myofibroblast (Myo 1.3) |
| 0.600181 | 0.93 | 0.563 | 8.65E-67 | 17 | Rpl22l1 | Myofibroblast (Myo 1.3) |
| 0.59756 | 0.811 | 0.301 | 5.05E-83 | 17 | Acta2 | Myofibroblast (Myo 1.3) |
| 0.592807 | 0.997 | 0.888 | 2.34E-66 | 17 | Rps12 | Myofibroblast (Myo 1.3) |
| 0.588299 | 0.977 | 0.75 | 7.29E-72 | 17 | Rpl14 | Myofibroblast (Myo 1.3) |
| 0.580094 | 0.794 | 0.327 | 3.52E-76 | 17 | Tuba1a | Myofibroblast (Myo 1.3) |
| 0.577638 | 1 | 0.914 | 2.30E-63 | 17 | Rps8 | Myofibroblast (Myo 1.3) |
| 0.577273 | 0.899 | 0.437 | 2.57E-75 | 17 | Rps27l | Myofibroblast (Myo 1.3) |
| 0.57676 | 0.915 | 0.53 | 7.18E-71 | 17 | Eif5a | Myofibroblast (Myo 1.3) |
| 0.575079 | 0.997 | 0.908 | 1.82E-56 | 17 | Rpl37 | Myofibroblast (Myo 1.3) |
| 0.565248 | 0.935 | 0.563 | 5.48E-66 | 17 | Rpl36al | Myofibroblast (Myo 1.3) |
| 0.561401 | 0.997 | 0.842 | 1.21E-67 | 17 | Rpl11 | Myofibroblast (Myo 1.3) |
| 0.560072 | 0.986 | 0.838 | 1.56E-58 | 17 | Ppia | Myofibroblast (Myo 1.3) |
| 0.556617 | 0.789 | 0.393 | 4.88E-59 | 17 | Gm10260 | Myofibroblast (Myo 1.3) |
| 0.556139 | 0.989 | 0.787 | 4.13E-66 | 17 | Rpl10a | Myofibroblast (Myo 1.3) |
| 0.554842 | 0.997 | 0.861 | 1.22E-58 | 17 | Rpl28 | Myofibroblast (Myo 1.3) |
| 0.552245 | 0.718 | 0.287 | 1.18E-71 | 17 | Manf | Myofibroblast (Myo 1.3) |
| 0.550064 | 0.997 | 0.871 | 5.71E-54 | 17 | Rps20 | Myofibroblast (Myo 1.3) |
| 0.548541 | 1 | 0.855 | 7.18E-55 | 17 | Rps15a | Myofibroblast (Myo 1.3) |
| 0.545677 | 0.735 | 0.255 | 3.77E-92 | 17 | Ssr2 | Myofibroblast (Myo 1.3) |
| 0.545479 | 0.997 | 0.854 | 7.61E-55 | 17 | Rpl36 | Myofibroblast (Myo 1.3) |
| 0.54523 | 0.727 | 0.245 | 4.43E-92 | 17 | Ppp1r14b | Myofibroblast (Myo 1.3) |
| 0.543902 | 0.994 | 0.849 | 7.31E-57 | 17 | Rpl32 | Myofibroblast (Myo 1.3) |
| 0.536874 | 0.859 | 0.49 | 1.05E-53 | 17 | Hmgb1 | Myofibroblast (Myo 1.3) |
| 0.53679 | 0.859 | 0.423 | 5.75E-68 | 17 | Ssr4 | Myofibroblast (Myo 1.3) |
| 0.533676 | 0.961 | 0.608 | 3.64E-58 | 17 | Ppib | Myofibroblast (Myo 1.3) |
| 0.533646 | 0.997 | 0.875 | 1.34E-62 | 17 | Rplp1 | Myofibroblast (Myo 1.3) |
| 0.533158 | 0.744 | 0.275 | 1.15E-82 | 17 | Tmem258 | Myofibroblast (Myo 1.3) |
| 0.529469 | 0.727 | 0.281 | 3.64E-76 | 17 | Pdia6 | Myofibroblast (Myo 1.3) |
| 0.528514 | 0.831 | 0.398 | 6.89E-62 | 17 | Tubb5 | Myofibroblast (Myo 1.3) |
| 0.525447 | 0.738 | 0.295 | 1.20E-71 | 17 | Ran | Myofibroblast (Myo 1.3) |
| 0.524324 | 0.992 | 0.861 | 3.64E-58 | 17 | Rps23 | Myofibroblast (Myo 1.3) |
| 0.521229 | 0.851 | 0.388 | 3.34E-70 | 17 | P4hb | Myofibroblast (Myo 1.3) |
| 0.513752 | 0.814 | 0.337 | 4.44E-70 | 17 | Rcn3 | Myofibroblast (Myo 1.3) |
| 0.513512 | 0.983 | 0.823 | 1.03E-54 | 17 | Rpl15 | Myofibroblast (Myo 1.3) |
| 0.511995 | 1 | 0.912 | 6.83E-57 | 17 | Rps27a | Myofibroblast (Myo 1.3) |
| 0.510403 | 0.893 | 0.414 | 2.79E-53 | 17 | Col5a1 | Myofibroblast (Myo 1.3) |
| 0.50898 | 0.825 | 0.377 | 1.58E-61 | 17 | Selenom | Myofibroblast (Myo 1.3) |
| 0.508564 | 0.918 | 0.573 | 1.45E-62 | 17 | Rpl31 | Myofibroblast (Myo 1.3) |
| 0.506509 | 0.887 | 0.437 | 4.63E-47 | 17 | Col6a3 | Myofibroblast (Myo 1.3) |
| 0.50037 | 0.915 | 0.58 | 4.63E-54 | 17 | Cox7c | Myofibroblast (Myo 1.3) |
| 0.500174 | 0.983 | 0.842 | 3.01E-53 | 17 | Rplp2 | Myofibroblast (Myo 1.3) |
| 0.498229 | 0.873 | 0.483 | 3.13E-49 | 17 | Calr | Myofibroblast (Myo 1.3) |
| 0.497533 | 0.983 | 0.837 | 2.21E-38 | 17 | Rps26 | Myofibroblast (Myo 1.3) |
| 0.495895 | 0.71 | 0.272 | 8.62E-64 | 17 | Lox | Myofibroblast (Myo 1.3) |
| 0.494303 | 0.932 | 0.617 | 3.12E-57 | 17 | Rbm3 | Myofibroblast (Myo 1.3) |
| 0.49351 | 0.946 | 0.689 | 6.27E-48 | 17 | Rpl36a | Myofibroblast (Myo 1.3) |
| 0.490749 | 0.685 | 0.29 | 1.66E-56 | 17 | Mif | Myofibroblast (Myo 1.3) |
| 0.490587 | 0.758 | 0.287 | 9.05E-79 | 17 | Krtcap2 | Myofibroblast (Myo 1.3) |
| 0.490446 | 0.699 | 0.232 | 2.40E-85 | 17 | Nme1 | Myofibroblast (Myo 1.3) |
| 0.486956 | 0.997 | 0.871 | 6.84E-44 | 17 | Rpl34 | Myofibroblast (Myo 1.3) |
| 0.483245 | 0.814 | 0.364 | 1.21E-67 | 17 | Eef1g | Myofibroblast (Myo 1.3) |
| 0.482582 | 1 | 0.884 | 4.60E-50 | 17 | Rps3a1 | Myofibroblast (Myo 1.3) |
| 0.482126 | 0.82 | 0.411 | 1.35E-56 | 17 | Pdia3 | Myofibroblast (Myo 1.3) |
| 0.481936 | 0.972 | 0.742 | 1.10E-46 | 17 | Rpl22 | Myofibroblast (Myo 1.3) |
| 0.470422 | 0.997 | 0.883 | 7.52E-50 | 17 | Rpl27a | Myofibroblast (Myo 1.3) |
| 0.470082 | 1 | 0.901 | 2.46E-46 | 17 | Rpl38 | Myofibroblast (Myo 1.3) |
| 0.467705 | 0.997 | 0.831 | 5.71E-48 | 17 | Rpl30 | Myofibroblast (Myo 1.3) |
| 0.465766 | 0.6 | 0.175 | 2.94E-87 | 17 | Lman1 | Myofibroblast (Myo 1.3) |
| 0.465632 | 0.89 | 0.431 | 1.45E-46 | 17 | Fstl1 | Myofibroblast (Myo 1.3) |
| 0.463503 | 0.946 | 0.605 | 1.64E-50 | 17 | Gapdh | Myofibroblast (Myo 1.3) |
| 0.463237 | 0.961 | 0.631 | 2.47E-42 | 17 | Ifitm3 | Myofibroblast (Myo 1.3) |
| 0.462216 | 0.997 | 0.867 | 1.86E-39 | 17 | Rplp0 | Myofibroblast (Myo 1.3) |
| 0.461392 | 1 | 0.905 | 4.15E-38 | 17 | Rpl39 | Myofibroblast (Myo 1.3) |
| 0.45873 | 0.946 | 0.687 | 1.18E-43 | 17 | Eef1b2 | Myofibroblast (Myo 1.3) |
| 0.452955 | 0.879 | 0.52 | 3.52E-44 | 17 | Anxa5 | Myofibroblast (Myo 1.3) |
| 0.450814 | 0.977 | 0.774 | 2.92E-40 | 17 | Hsp90ab1 | Myofibroblast (Myo 1.3) |
| 0.449955 | 0.676 | 0.251 | 2.79E-66 | 17 | Ckap4 | Myofibroblast (Myo 1.3) |
| 0.449176 | 0.992 | 0.845 | 7.97E-49 | 17 | Rps19 | Myofibroblast (Myo 1.3) |
| 0.44849 | 1 | 0.897 | 5.61E-48 | 17 | Rpl13 | Myofibroblast (Myo 1.3) |
| 0.44837 | 0.994 | 0.854 | 7.99E-49 | 17 | Rpl18 | Myofibroblast (Myo 1.3) |
| 0.447798 | 0.994 | 0.83 | 1.27E-25 | 17 | Vim | Myofibroblast (Myo 1.3) |
| 0.446635 | 0.913 | 0.462 | 4.80E-49 | 17 | Cald1 | Myofibroblast (Myo 1.3) |
| 0.44385 | 0.994 | 0.847 | 1.83E-48 | 17 | Rpl24 | Myofibroblast (Myo 1.3) |
| 0.443511 | 0.913 | 0.62 | 1.39E-36 | 17 | Anxa2 | Myofibroblast (Myo 1.3) |
| 0.442759 | 0.994 | 0.859 | 5.58E-47 | 17 | Rpl6 | Myofibroblast (Myo 1.3) |
| 0.441135 | 0.834 | 0.444 | 2.33E-49 | 17 | Hint1 | Myofibroblast (Myo 1.3) |
| 0.440915 | 0.772 | 0.334 | 4.50E-61 | 17 | Atp5g1 | Myofibroblast (Myo 1.3) |
| 0.437424 | 0.456 | 0.092 | 4.13E-108 | 17 | Fkbp11 | Myofibroblast (Myo 1.3) |
| 0.437202 | 0.876 | 0.522 | 9.71E-42 | 17 | Tagln2 | Myofibroblast (Myo 1.3) |
| 0.434754 | 0.969 | 0.864 | 1.04E-33 | 17 | Actg1 | Myofibroblast (Myo 1.3) |
| 0.431863 | 0.994 | 0.846 | 1.07E-46 | 17 | Rps5 | Myofibroblast (Myo 1.3) |
| 0.431646 | 0.997 | 0.908 | 6.57E-38 | 17 | Rps21 | Myofibroblast (Myo 1.3) |
| 0.429167 | 0.727 | 0.299 | 2.43E-61 | 17 | Eef1d | Myofibroblast (Myo 1.3) |
| 0.429031 | 0.887 | 0.565 | 2.03E-44 | 17 | Elob | Myofibroblast (Myo 1.3) |
| 0.425515 | 0.651 | 0.261 | 5.78E-51 | 17 | Fbln2 | Myofibroblast (Myo 1.3) |
| 0.424624 | 0.91 | 0.467 | 7.29E-35 | 17 | Col6a1 | Myofibroblast (Myo 1.3) |
| 0.422719 | 0.859 | 0.431 | 2.70E-52 | 17 | Slc25a4 | Myofibroblast (Myo 1.3) |
| 0.419749 | 0.986 | 0.524 | 3.67E-26 | 17 | Bgn | Myofibroblast (Myo 1.3) |
| 0.419408 | 0.772 | 0.276 | 9.39E-77 | 17 | Tpm2 | Myofibroblast (Myo 1.3) |
| 0.418964 | 0.986 | 0.769 | 1.31E-30 | 17 | Crip1 | Myofibroblast (Myo 1.3) |
| 0.417687 | 0.321 | 0.079 | 2.32E-55 | 17 | Il1rl1 | Myofibroblast (Myo 1.3) |
| 0.41743 | 0.997 | 0.86 | 2.51E-44 | 17 | Rps11 | Myofibroblast (Myo 1.3) |
| 0.416995 | 0.823 | 0.398 | 5.30E-53 | 17 | Ndufa4 | Myofibroblast (Myo 1.3) |
| 0.413034 | 0.972 | 0.762 | 3.62E-39 | 17 | Rpl3 | Myofibroblast (Myo 1.3) |
| 0.410943 | 0.485 | 0.122 | 1.78E-84 | 17 | Kdelr3 | Myofibroblast (Myo 1.3) |
| 0.409016 | 0.992 | 0.884 | 1.12E-43 | 17 | Rps10 | Myofibroblast (Myo 1.3) |
| 0.405503 | 0.966 | 0.749 | 6.72E-41 | 17 | Rpl7a | Myofibroblast (Myo 1.3) |
| 0.400824 | 0.806 | 0.408 | 6.92E-44 | 17 | Tpm4 | Myofibroblast (Myo 1.3) |
| 0.400585 | 0.837 | 0.438 | 2.05E-45 | 17 | Dad1 | Myofibroblast (Myo 1.3) |
| 0.399534 | 0.986 | 0.836 | 4.78E-35 | 17 | Serf2 | Myofibroblast (Myo 1.3) |
| 0.399332 | 0.67 | 0.277 | 2.82E-52 | 17 | Hdlbp | Myofibroblast (Myo 1.3) |
| 0.397666 | 0.918 | 0.637 | 3.50E-26 | 17 | Rps18 | Myofibroblast (Myo 1.3) |
| 0.396267 | 0.527 | 0.174 | 5.24E-58 | 17 | Loxl2 | Myofibroblast (Myo 1.3) |
| 0.393812 | 0.972 | 0.82 | 3.45E-43 | 17 | Myl6 | Myofibroblast (Myo 1.3) |
| 0.391723 | 0.797 | 0.434 | 1.42E-39 | 17 | Reep5 | Myofibroblast (Myo 1.3) |
| 0.391562 | 0.428 | 0.131 | 4.67E-54 | 17 | Sod3 | Myofibroblast (Myo 1.3) |
| 0.389006 | 0.594 | 0.214 | 6.87E-59 | 17 | Tmem167 | Myofibroblast (Myo 1.3) |
| 0.388265 | 1 | 0.927 | 4.29E-28 | 17 | Rpl37a | Myofibroblast (Myo 1.3) |
| 0.387114 | 0.989 | 0.795 | 3.27E-39 | 17 | Rpl27 | Myofibroblast (Myo 1.3) |
| 0.386852 | 0.769 | 0.373 | 6.60E-46 | 17 | Atpif1 | Myofibroblast (Myo 1.3) |
| 0.384586 | 0.701 | 0.335 | 3.14E-42 | 17 | Ost4 | Myofibroblast (Myo 1.3) |
| 0.384198 | 0.983 | 0.837 | 1.50E-39 | 17 | Rps3 | Myofibroblast (Myo 1.3) |
| 0.383788 | 0.527 | 0.221 | 7.19E-35 | 17 | Tm4sf1 | Myofibroblast (Myo 1.3) |
| 0.383146 | 0.87 | 0.543 | 5.60E-33 | 17 | Myl12a | Myofibroblast (Myo 1.3) |
| 0.379931 | 0.749 | 0.502 | 1.10E-23 | 17 | Uba52 | Myofibroblast (Myo 1.3) |
| 0.374839 | 0.572 | 0.216 | 1.03E-51 | 17 | Swi5 | Myofibroblast (Myo 1.3) |
| 0.372889 | 0.941 | 0.735 | 4.00E-31 | 17 | Rps15 | Myofibroblast (Myo 1.3) |
| 0.37209 | 0.994 | 0.882 | 3.49E-39 | 17 | Rpl19 | Myofibroblast (Myo 1.3) |
| 0.369886 | 0.952 | 0.681 | 5.79E-33 | 17 | Rpl23a | Myofibroblast (Myo 1.3) |
| 0.368192 | 1 | 0.585 | 5.00E-19 | 17 | Sparc | Myofibroblast (Myo 1.3) |
| 0.367346 | 0.949 | 0.63 | 3.67E-31 | 17 | Gnas | Myofibroblast (Myo 1.3) |
| 0.361876 | 0.546 | 0.21 | 3.97E-47 | 17 | Selenos | Myofibroblast (Myo 1.3) |
| 0.361843 | 0.485 | 0.157 | 1.30E-55 | 17 | Ift20 | Myofibroblast (Myo 1.3) |
| 0.361481 | 0.986 | 0.762 | 1.59E-40 | 17 | Rpl12 | Myofibroblast (Myo 1.3) |
| 0.36137 | 0.98 | 0.831 | 2.53E-31 | 17 | Rpl21 | Myofibroblast (Myo 1.3) |
| 0.359467 | 0.411 | 0.176 | 1.30E-23 | 17 | Mfap5 | Myofibroblast (Myo 1.3) |
| 0.358439 | 0.561 | 0.225 | 8.27E-44 | 17 | Serpinb6a | Myofibroblast (Myo 1.3) |
| 0.357956 | 1 | 0.901 | 6.15E-32 | 17 | Rps24 | Myofibroblast (Myo 1.3) |
| 0.3541 | 0.676 | 0.321 | 2.57E-38 | 17 | Uqcrq | Myofibroblast (Myo 1.3) |
| 0.347642 | 0.989 | 0.91 | 7.08E-35 | 17 | Rps16 | Myofibroblast (Myo 1.3) |
| 0.343923 | 0.594 | 0.278 | 6.75E-35 | 17 | Tmed9 | Myofibroblast (Myo 1.3) |
| 0.343869 | 0.873 | 0.556 | 2.16E-30 | 17 | Dynll1 | Myofibroblast (Myo 1.3) |
| 0.339263 | 0.955 | 0.701 | 1.11E-28 | 17 | Naca | Myofibroblast (Myo 1.3) |
| 0.338436 | 0.561 | 0.225 | 2.44E-42 | 17 | Fkbp1a | Myofibroblast (Myo 1.3) |
| 0.338425 | 0.972 | 0.757 | 7.72E-29 | 17 | Rack1 | Myofibroblast (Myo 1.3) |
| 0.337695 | 0.845 | 0.431 | 4.41E-30 | 17 | Col6a2 | Myofibroblast (Myo 1.3) |
| 0.337351 | 0.631 | 0.314 | 2.53E-31 | 17 | Psma7 | Myofibroblast (Myo 1.3) |
| 0.337196 | 0.718 | 0.397 | 1.67E-30 | 17 | Snrpg | Myofibroblast (Myo 1.3) |
| 0.335388 | 0.346 | 0.144 | 8.95E-22 | 17 | Inhba | Myofibroblast (Myo 1.3) |
| 0.334419 | 0.721 | 0.354 | 9.55E-33 | 17 | Pcolce | Myofibroblast (Myo 1.3) |
| 0.333719 | 0.721 | 0.342 | 1.07E-34 | 17 | Fbn1 | Myofibroblast (Myo 1.3) |
| 0.329598 | 0.879 | 0.417 | 9.15E-36 | 17 | Serpinf1 | Myofibroblast (Myo 1.3) |
| 0.329467 | 0.594 | 0.269 | 1.04E-35 | 17 | Pebp1 | Myofibroblast (Myo 1.3) |
| 0.328926 | 0.806 | 0.556 | 8.37E-21 | 17 | Hspa5 | Myofibroblast (Myo 1.3) |
| 0.326333 | 0.487 | 0.199 | 8.18E-36 | 17 | Arl1 | Myofibroblast (Myo 1.3) |
| 0.323982 | 0.744 | 0.393 | 4.19E-32 | 17 | Atp5b | Myofibroblast (Myo 1.3) |
| 0.323851 | 0.98 | 0.853 | 2.06E-34 | 17 | Rpl8 | Myofibroblast (Myo 1.3) |
| 0.323341 | 0.992 | 0.845 | 9.25E-26 | 17 | Rps4x | Myofibroblast (Myo 1.3) |
| 0.317754 | 0.454 | 0.179 | 7.23E-35 | 17 | Snrpf | Myofibroblast (Myo 1.3) |
| 0.316829 | 0.563 | 0.256 | 9.59E-33 | 17 | Calu | Myofibroblast (Myo 1.3) |
| 0.3166 | 0.352 | 0.089 | 5.25E-58 | 17 | Lrrc59 | Myofibroblast (Myo 1.3) |
| 0.316196 | 1 | 0.909 | 1.98E-22 | 17 | Rps28 | Myofibroblast (Myo 1.3) |
| 0.315357 | 0.994 | 0.917 | 1.44E-25 | 17 | Rpl23 | Myofibroblast (Myo 1.3) |
| 0.312057 | 0.518 | 0.213 | 1.88E-35 | 17 | Loxl1 | Myofibroblast (Myo 1.3) |
| 0.311449 | 0.383 | 0.117 | 9.30E-47 | 17 | Fkbp10 | Myofibroblast (Myo 1.3) |
| 0.310607 | 0.521 | 0.223 | 6.81E-34 | 17 | Hspe1 | Myofibroblast (Myo 1.3) |
| 0.309083 | 0.997 | 0.869 | 2.13E-31 | 17 | Rpl9 | Myofibroblast (Myo 1.3) |
| 0.308995 | 0.411 | 0.151 | 3.32E-36 | 17 | Uqcc2 | Myofibroblast (Myo 1.3) |
| 0.306291 | 0.485 | 0.199 | 1.42E-34 | 17 | Timm13 | Myofibroblast (Myo 1.3) |
| 0.305951 | 0.992 | 0.837 | 2.73E-25 | 17 | Rpl26 | Myofibroblast (Myo 1.3) |
| 0.305375 | 0.654 | 0.332 | 6.66E-29 | 17 | Eif2s2 | Myofibroblast (Myo 1.3) |
| 0.303999 | 0.676 | 0.376 | 1.94E-25 | 17 | Prdx2 | Myofibroblast (Myo 1.3) |
| 0.303454 | 0.276 | 0.076 | 8.66E-39 | 17 | Fibin | Myofibroblast (Myo 1.3) |
| 0.303273 | 0.972 | 0.841 | 6.89E-20 | 17 | Ptma | Myofibroblast (Myo 1.3) |
| 0.302579 | 0.82 | 0.481 | 5.27E-27 | 17 | Atp5g2 | Myofibroblast (Myo 1.3) |
| 0.302535 | 0.761 | 0.376 | 7.73E-33 | 17 | Tpm1 | Myofibroblast (Myo 1.3) |
| 0.300646 | 0.989 | 0.849 | 1.15E-30 | 17 | Rps13 | Myofibroblast (Myo 1.3) |
| 0.300534 | 0.577 | 0.278 | 3.54E-29 | 17 | Atp5o | Myofibroblast (Myo 1.3) |
| 0.300222 | 0.513 | 0.225 | 2.34E-31 | 17 | Ssr3 | Myofibroblast (Myo 1.3) |
| 0.297206 | 0.507 | 0.237 | 8.20E-27 | 17 | Abracl | Myofibroblast (Myo 1.3) |
| 0.296858 | 0.476 | 0.204 | 2.39E-30 | 17 | Snrpd2 | Myofibroblast (Myo 1.3) |
| 0.296077 | 0.485 | 0.208 | 3.62E-30 | 17 | Serp1 | Myofibroblast (Myo 1.3) |
| 0.295619 | 0.589 | 0.285 | 1.29E-28 | 17 | Hmgn1 | Myofibroblast (Myo 1.3) |
| 0.295159 | 0.992 | 0.892 | 3.55E-19 | 17 | Rpl35a | Myofibroblast (Myo 1.3) |
| 0.295043 | 0.865 | 0.585 | 6.46E-23 | 17 | Cox6c | Myofibroblast (Myo 1.3) |
| 0.295011 | 0.82 | 0.473 | 6.26E-26 | 17 | Selenof | Myofibroblast (Myo 1.3) |
| 0.293019 | 0.428 | 0.16 | 3.06E-35 | 17 | Timm10b | Myofibroblast (Myo 1.3) |
| 0.289362 | 0.448 | 0.18 | 5.75E-32 | 17 | Ranbp1 | Myofibroblast (Myo 1.3) |
| 0.287798 | 0.949 | 0.752 | 1.36E-20 | 17 | Rps25 | Myofibroblast (Myo 1.3) |
| 0.287434 | 0.715 | 0.42 | 1.23E-23 | 17 | Atp5j2 | Myofibroblast (Myo 1.3) |
| 0.28664 | 0.682 | 0.359 | 1.64E-27 | 17 | Cox7a2 | Myofibroblast (Myo 1.3) |
| 0.284527 | 0.592 | 0.306 | 5.95E-25 | 17 | Tmed2 | Myofibroblast (Myo 1.3) |
| 0.283199 | 0.896 | 0.594 | 5.84E-25 | 17 | Rpl4 | Myofibroblast (Myo 1.3) |
| 0.281108 | 0.983 | 0.55 | 2.20E-23 | 17 | Col1a1 | Myofibroblast (Myo 1.3) |
| 0.279751 | 0.792 | 0.481 | 3.77E-20 | 17 | Gas5 | Myofibroblast (Myo 1.3) |
| 0.2792 | 0.355 | 0.119 | 8.67E-36 | 17 | Ssr1 | Myofibroblast (Myo 1.3) |
| 0.276658 | 0.504 | 0.244 | 5.08E-24 | 17 | Cox6a1 | Myofibroblast (Myo 1.3) |
| 0.273537 | 0.637 | 0.332 | 4.07E-24 | 17 | Ldha | Myofibroblast (Myo 1.3) |
| 0.273302 | 0.304 | 0.077 | 1.47E-48 | 17 | Selenoh | Myofibroblast (Myo 1.3) |
| 0.27323 | 0.676 | 0.38 | 6.00E-22 | 17 | Serbp1 | Myofibroblast (Myo 1.3) |
| 0.272333 | 0.431 | 0.182 | 2.54E-27 | 17 | Maged1 | Myofibroblast (Myo 1.3) |
| 0.271331 | 0.721 | 0.443 | 4.08E-15 | 17 | Pmepa1 | Myofibroblast (Myo 1.3) |
| 0.271128 | 0.532 | 0.267 | 2.28E-23 | 17 | Psma3 | Myofibroblast (Myo 1.3) |
| 0.270623 | 0.4 | 0.164 | 6.42E-27 | 17 | Surf4 | Myofibroblast (Myo 1.3) |
| 0.270461 | 0.462 | 0.218 | 1.64E-22 | 17 | Anp32b | Myofibroblast (Myo 1.3) |
| 0.269784 | 0.431 | 0.185 | 4.56E-26 | 17 | Prdx4 | Myofibroblast (Myo 1.3) |
| 0.26904 | 0.617 | 0.331 | 2.40E-22 | 17 | Atp5k | Myofibroblast (Myo 1.3) |
| 0.268964 | 0.741 | 0.435 | 1.46E-21 | 17 | Bsg | Myofibroblast (Myo 1.3) |
| 0.268695 | 0.53 | 0.268 | 5.23E-22 | 17 | Cox7b | Myofibroblast (Myo 1.3) |
| 0.268083 | 0.4 | 0.164 | 8.19E-27 | 17 | Dap | Myofibroblast (Myo 1.3) |
| 0.267784 | 0.994 | 0.839 | 4.92E-26 | 17 | Rps7 | Myofibroblast (Myo 1.3) |
| 0.267163 | 0.392 | 0.158 | 4.29E-27 | 17 | Eif3i | Myofibroblast (Myo 1.3) |
| 0.266684 | 0.915 | 0.653 | 1.19E-20 | 17 | Btf3 | Myofibroblast (Myo 1.3) |
| 0.26528 | 0.355 | 0.126 | 1.09E-31 | 17 | Rcn1 | Myofibroblast (Myo 1.3) |
| 0.263836 | 0.994 | 0.871 | 3.07E-23 | 17 | Rpl18a | Myofibroblast (Myo 1.3) |
| 0.261928 | 0.715 | 0.413 | 1.12E-21 | 17 | Arf4 | Myofibroblast (Myo 1.3) |
| 0.26121 | 0.608 | 0.369 | 1.22E-12 | 17 | Nupr1 | Myofibroblast (Myo 1.3) |
| 0.260386 | 0.406 | 0.173 | 6.85E-25 | 17 | Kdelr1 | Myofibroblast (Myo 1.3) |
| 0.259976 | 0.363 | 0.139 | 1.93E-27 | 17 | Pdap1 | Myofibroblast (Myo 1.3) |
| 0.259086 | 0.259 | 0.051 | 1.57E-59 | 17 | Ppa1 | Myofibroblast (Myo 1.3) |
| 0.25855 | 0.4 | 0.183 | 1.11E-20 | 17 | Ybx3 | Myofibroblast (Myo 1.3) |
| 0.258311 | 0.594 | 0.318 | 3.47E-21 | 17 | Tceal9 | Myofibroblast (Myo 1.3) |
| 0.257417 | 0.549 | 0.281 | 8.37E-22 | 17 | Micos10 | Myofibroblast (Myo 1.3) |
| 0.255874 | 0.992 | 0.552 | 1.61E-19 | 17 | Col3a1 | Myofibroblast (Myo 1.3) |
| 0.253351 | 0.431 | 0.2 | 2.19E-21 | 17 | Morf4l2 | Myofibroblast (Myo 1.3) |
| 0.252107 | 0.927 | 0.71 | 2.90E-21 | 17 | Rps6 | Myofibroblast (Myo 1.3) |
| 0.25037 | 0.445 | 0.221 | 2.23E-18 | 17 | Nap1l1 | Myofibroblast (Myo 1.3) |
| 0.25008 | 1 | 0.928 | 4.95E-14 | 17 | Eef1a1 | Myofibroblast (Myo 1.3) |
| 3.079083 | 0.751 | 0.071 | 0 | 18 | Acp5 | Macrophages (Mac 1.5) |
| 0.806608 | 0.469 | 0.003 | 0 | 18 | Atp6v0d2 | Macrophages (Mac 1.5) |
| 0.651424 | 0.444 | 0.015 | 0 | 18 | Chchd10 | Macrophages (Mac 1.5) |
| 0.601362 | 0.419 | 0.021 | 0 | 18 | Slc37a2 | Macrophages (Mac 1.5) |
| 0.414695 | 0.285 | 0.002 | 0 | 18 | Slc9b2 | Macrophages (Mac 1.5) |
| 0.564828 | 0.451 | 0.032 | 8.39E-269 | 18 | Clec12a | Macrophages (Mac 1.5) |
| 0.831958 | 0.599 | 0.062 | 1.16E-260 | 18 | Atp6v1b2 | Macrophages (Mac 1.5) |
| 0.596538 | 0.491 | 0.042 | 2.97E-250 | 18 | Gpr137b | Macrophages (Mac 1.5) |
| 0.704992 | 0.534 | 0.055 | 6.14E-232 | 18 | Nfatc1 | Macrophages (Mac 1.5) |
| 0.786632 | 0.628 | 0.082 | 5.17E-215 | 18 | Jdp2 | Macrophages (Mac 1.5) |
| 0.581072 | 0.527 | 0.059 | 6.68E-206 | 18 | Cyc1 | Macrophages (Mac 1.5) |
| 0.48862 | 0.455 | 0.047 | 1.32E-190 | 18 | Mrpl12 | Macrophages (Mac 1.5) |
| 1.195668 | 0.866 | 0.19 | 2.94E-187 | 18 | Ifi30 | Macrophages (Mac 1.5) |
| 0.690989 | 0.643 | 0.097 | 4.80E-185 | 18 | Nrp2 | Macrophages (Mac 1.5) |
| 0.430901 | 0.336 | 0.026 | 6.79E-184 | 18 | Pstpip1 | Macrophages (Mac 1.5) |
| 0.649198 | 0.498 | 0.065 | 8.09E-169 | 18 | Atp6v1d | Macrophages (Mac 1.5) |
| 0.833187 | 0.596 | 0.096 | 5.20E-165 | 18 | Atp6v1a | Macrophages (Mac 1.5) |
| 0.457211 | 0.354 | 0.033 | 8.09E-164 | 18 | 1110008P14Rik | Macrophages (Mac 1.5) |
| 0.535161 | 0.126 | 0.004 | 9.02E-152 | 18 | Mt3 | Macrophages (Mac 1.5) |
| 0.470294 | 0.408 | 0.047 | 5.23E-148 | 18 | Tcirg1 | Macrophages (Mac 1.5) |
| 0.392161 | 0.314 | 0.028 | 1.82E-147 | 18 | Xpr1 | Macrophages (Mac 1.5) |
| 0.281173 | 0.177 | 0.009 | 1.55E-141 | 18 | Tfrc | Macrophages (Mac 1.5) |
| 0.730044 | 0.61 | 0.108 | 1.31E-139 | 18 | Ccl9 | Macrophages (Mac 1.5) |
| 0.321347 | 0.17 | 0.008 | 9.86E-138 | 18 | Fxyd2 | Macrophages (Mac 1.5) |
| 0.545906 | 0.516 | 0.081 | 3.84E-137 | 18 | Sdhb | Macrophages (Mac 1.5) |
| 0.436695 | 0.433 | 0.058 | 1.35E-135 | 18 | C1qbp | Macrophages (Mac 1.5) |
| 1.18932 | 0.87 | 0.332 | 2.86E-134 | 18 | Cox5a | Macrophages (Mac 1.5) |
| 0.47169 | 0.43 | 0.058 | 3.49E-133 | 18 | Naa50 | Macrophages (Mac 1.5) |
| 0.643646 | 0.596 | 0.113 | 2.79E-132 | 18 | Cycs | Macrophages (Mac 1.5) |
| 1.794774 | 0.982 | 0.49 | 8.69E-130 | 18 | Atp6v0c | Macrophages (Mac 1.5) |
| 0.385081 | 0.321 | 0.033 | 1.15E-129 | 18 | Aco2 | Macrophages (Mac 1.5) |
| 1.02138 | 0.823 | 0.256 | 4.57E-129 | 18 | Uqcr11 | Macrophages (Mac 1.5) |
| 0.253131 | 0.206 | 0.014 | 7.02E-126 | 18 | Hagh | Macrophages (Mac 1.5) |
| 1.167872 | 0.949 | 0.447 | 1.07E-125 | 18 | Gpx1 | Macrophages (Mac 1.5) |
| 0.675743 | 0.643 | 0.133 | 4.35E-124 | 18 | Cd68 | Macrophages (Mac 1.5) |
| 1.096454 | 0.848 | 0.28 | 6.12E-124 | 18 | Atp6v0b | Macrophages (Mac 1.5) |
| 0.848148 | 0.751 | 0.206 | 1.82E-122 | 18 | Atp5g3 | Macrophages (Mac 1.5) |
| 1.231998 | 0.949 | 0.469 | 1.47E-121 | 18 | Sh3bgrl3 | Macrophages (Mac 1.5) |
| 1.117626 | 0.946 | 0.362 | 2.71E-119 | 18 | Cstb | Macrophages (Mac 1.5) |
| 1.644183 | 0.964 | 0.533 | 1.75E-118 | 18 | S100a4 | Macrophages (Mac 1.5) |
| 0.74205 | 0.812 | 0.228 | 6.43E-117 | 18 | Capg | Macrophages (Mac 1.5) |
| 0.407538 | 0.35 | 0.044 | 1.25E-116 | 18 | Ak2 | Macrophages (Mac 1.5) |
| 0.575815 | 0.57 | 0.115 | 9.78E-116 | 18 | Ndufab1 | Macrophages (Mac 1.5) |
| 0.936567 | 0.859 | 0.335 | 1.96E-114 | 18 | Atp5g1 | Macrophages (Mac 1.5) |
| 0.579649 | 0.574 | 0.113 | 3.05E-114 | 18 | Csf1r | Macrophages (Mac 1.5) |
| 0.747436 | 0.715 | 0.2 | 1.84E-110 | 18 | Ndufb8 | Macrophages (Mac 1.5) |
| 0.446188 | 0.451 | 0.077 | 2.59E-104 | 18 | Clec4a2 | Macrophages (Mac 1.5) |
| 0.619009 | 0.7 | 0.186 | 1.03E-102 | 18 | Vdac2 | Macrophages (Mac 1.5) |
| 1.137841 | 0.964 | 0.597 | 1.28E-101 | 18 | Txn1 | Macrophages (Mac 1.5) |
| 0.530614 | 0.509 | 0.101 | 1.17E-100 | 18 | Atp6ap2 | Macrophages (Mac 1.5) |
| 0.621097 | 0.722 | 0.192 | 3.67E-99 | 18 | Spi1 | Macrophages (Mac 1.5) |
| 0.84264 | 0.823 | 0.326 | 6.27E-99 | 18 | Slc25a5 | Macrophages (Mac 1.5) |
| 1.021026 | 0.845 | 0.358 | 4.62E-97 | 18 | Atp6v0e | Macrophages (Mac 1.5) |
| 0.459361 | 0.462 | 0.087 | 2.16E-96 | 18 | Ndufs7 | Macrophages (Mac 1.5) |
| 0.489462 | 0.473 | 0.091 | 1.71E-95 | 18 | G3bp1 | Macrophages (Mac 1.5) |
| 0.92854 | 0.671 | 0.195 | 1.36E-94 | 18 | Atp6v1e1 | Macrophages (Mac 1.5) |
| 0.434474 | 0.415 | 0.073 | 1.86E-92 | 18 | Uqcrfs1 | Macrophages (Mac 1.5) |
| 0.425749 | 0.412 | 0.073 | 5.92E-92 | 18 | Nhp2 | Macrophages (Mac 1.5) |
| 0.807669 | 0.859 | 0.392 | 2.57E-90 | 18 | Atp5b | Macrophages (Mac 1.5) |
| 0.710572 | 0.755 | 0.248 | 4.93E-89 | 18 | Aprt | Macrophages (Mac 1.5) |
| 0.41151 | 0.422 | 0.078 | 2.59E-88 | 18 | Mdh1 | Macrophages (Mac 1.5) |
| 0.919079 | 0.74 | 0.271 | 8.42E-87 | 18 | Uqcr10 | Macrophages (Mac 1.5) |
| 0.293674 | 0.267 | 0.033 | 1.77E-86 | 18 | Sms | Macrophages (Mac 1.5) |
| 0.475547 | 0.495 | 0.107 | 4.99E-86 | 18 | Hspd1 | Macrophages (Mac 1.5) |
| 0.71715 | 0.747 | 0.265 | 1.57E-84 | 18 | Cox7b | Macrophages (Mac 1.5) |
| 0.444529 | 0.419 | 0.079 | 1.42E-83 | 18 | Trem2 | Macrophages (Mac 1.5) |
| 0.791525 | 0.91 | 0.454 | 1.21E-82 | 18 | Cox6b1 | Macrophages (Mac 1.5) |
| 0.440266 | 0.484 | 0.104 | 2.00E-82 | 18 | Rgs10 | Macrophages (Mac 1.5) |
| 0.413198 | 0.43 | 0.085 | 6.16E-82 | 18 | Phb2 | Macrophages (Mac 1.5) |
| 0.589994 | 0.69 | 0.215 | 8.87E-82 | 18 | Erp29 | Macrophages (Mac 1.5) |
| 0.59379 | 0.83 | 0.276 | 9.31E-82 | 18 | Ucp2 | Macrophages (Mac 1.5) |
| 0.951294 | 0.91 | 0.471 | 1.09E-81 | 18 | Npc2 | Macrophages (Mac 1.5) |
| 0.277996 | 0.242 | 0.03 | 4.71E-80 | 18 | Atp6v1c1 | Macrophages (Mac 1.5) |
| 0.770381 | 0.83 | 0.358 | 1.13E-79 | 18 | Cox7a2 | Macrophages (Mac 1.5) |
| 0.764186 | 0.866 | 0.394 | 6.71E-79 | 18 | Aldoa | Macrophages (Mac 1.5) |
| 0.779401 | 0.924 | 0.386 | 2.09E-77 | 18 | Lgals3 | Macrophages (Mac 1.5) |
| 0.732175 | 0.841 | 0.373 | 3.97E-77 | 18 | Atpif1 | Macrophages (Mac 1.5) |
| 0.563755 | 0.599 | 0.173 | 7.69E-77 | 18 | Ndufb5 | Macrophages (Mac 1.5) |
| 1.684086 | 0.329 | 0.058 | 1.34E-76 | 18 | Mmp9 | Macrophages (Mac 1.5) |
| 0.392026 | 0.412 | 0.083 | 4.91E-76 | 18 | Ndufa12 | Macrophages (Mac 1.5) |
| 0.454125 | 0.513 | 0.125 | 1.28E-75 | 18 | Rab5if | Macrophages (Mac 1.5) |
| 0.842891 | 0.856 | 0.4 | 5.38E-75 | 18 | Ndufa4 | Macrophages (Mac 1.5) |
| 0.78494 | 0.805 | 0.358 | 3.05E-74 | 18 | Cox5b | Macrophages (Mac 1.5) |
| 0.323376 | 0.289 | 0.045 | 2.07E-73 | 18 | Hsd17b10 | Macrophages (Mac 1.5) |
| 0.729027 | 0.776 | 0.321 | 9.69E-73 | 18 | Uqcrq | Macrophages (Mac 1.5) |
| 0.837216 | 0.993 | 0.838 | 1.13E-72 | 18 | Ppia | Macrophages (Mac 1.5) |
| 0.55748 | 0.552 | 0.153 | 1.37E-72 | 18 | Mdh2 | Macrophages (Mac 1.5) |
| 0.590476 | 0.581 | 0.173 | 1.26E-71 | 18 | Ndufc1 | Macrophages (Mac 1.5) |
| 0.780378 | 0.841 | 0.419 | 6.44E-71 | 18 | Atp5j2 | Macrophages (Mac 1.5) |
| 0.380125 | 0.415 | 0.088 | 1.01E-70 | 18 | Sec11c | Macrophages (Mac 1.5) |
| 0.355736 | 0.365 | 0.071 | 2.70E-70 | 18 | Ndufb6 | Macrophages (Mac 1.5) |
| 0.939678 | 0.96 | 0.636 | 9.84E-70 | 18 | S100a10 | Macrophages (Mac 1.5) |
| 0.488829 | 0.538 | 0.149 | 4.92E-68 | 18 | Ndufb10 | Macrophages (Mac 1.5) |
| 0.658772 | 0.74 | 0.313 | 1.32E-67 | 18 | Atp5f1 | Macrophages (Mac 1.5) |
| 0.63521 | 0.751 | 0.311 | 2.79E-67 | 18 | Atp5a1 | Macrophages (Mac 1.5) |
| 0.498196 | 0.498 | 0.132 | 4.44E-67 | 18 | Sin3b | Macrophages (Mac 1.5) |
| 0.817276 | 0.949 | 0.668 | 8.42E-67 | 18 | Cox8a | Macrophages (Mac 1.5) |
| 0.438578 | 0.289 | 0.049 | 1.33E-66 | 18 | Snx10 | Macrophages (Mac 1.5) |
| 0.316399 | 0.307 | 0.055 | 1.09E-64 | 18 | Ciao2a | Macrophages (Mac 1.5) |
| 0.848078 | 0.953 | 0.753 | 1.14E-64 | 18 | Cox4i1 | Macrophages (Mac 1.5) |
| 0.302442 | 0.292 | 0.051 | 8.49E-64 | 18 | Mrpl51 | Macrophages (Mac 1.5) |
| 1.063687 | 0.874 | 0.516 | 1.31E-63 | 18 | H2afz | Macrophages (Mac 1.5) |
| 0.526313 | 0.653 | 0.222 | 1.92E-63 | 18 | Hspe1 | Macrophages (Mac 1.5) |
| 0.624301 | 0.881 | 0.481 | 1.92E-63 | 18 | Atp5g2 | Macrophages (Mac 1.5) |
| 0.469005 | 0.621 | 0.198 | 4.72E-63 | 18 | Timm13 | Macrophages (Mac 1.5) |
| 0.461226 | 0.592 | 0.184 | 2.37E-62 | 18 | Park7 | Macrophages (Mac 1.5) |
| 0.753608 | 0.888 | 0.552 | 4.80E-62 | 18 | Atp5l | Macrophages (Mac 1.5) |
| 0.422332 | 0.375 | 0.082 | 6.34E-62 | 18 | Hexb | Macrophages (Mac 1.5) |
| 0.611843 | 0.809 | 0.385 | 3.31E-61 | 18 | Atp5d | Macrophages (Mac 1.5) |
| 0.458312 | 0.606 | 0.196 | 1.81E-59 | 18 | Pgls | Macrophages (Mac 1.5) |
| 0.813071 | 0.758 | 0.35 | 3.10E-59 | 18 | Atp6v1g1 | Macrophages (Mac 1.5) |
| 0.45542 | 0.935 | 0.321 | 3.11E-59 | 18 | Tyrobp | Macrophages (Mac 1.5) |
| 0.616705 | 0.751 | 0.33 | 4.40E-59 | 18 | Atp5k | Macrophages (Mac 1.5) |
| 0.69937 | 0.996 | 0.846 | 5.21E-59 | 18 | Rps19 | Macrophages (Mac 1.5) |
| 0.479088 | 0.693 | 0.25 | 1.38E-58 | 18 | Tomm20 | Macrophages (Mac 1.5) |
| 0.577446 | 0.693 | 0.278 | 1.79E-58 | 18 | Atp5o | Macrophages (Mac 1.5) |
| 0.430757 | 0.433 | 0.11 | 2.24E-56 | 18 | Pltp | Macrophages (Mac 1.5) |
| 0.532179 | 0.653 | 0.242 | 2.25E-56 | 18 | Cox6a1 | Macrophages (Mac 1.5) |
| 0.346359 | 0.401 | 0.097 | 8.40E-56 | 18 | Timm23 | Macrophages (Mac 1.5) |
| 0.593195 | 0.661 | 0.262 | 1.72E-55 | 18 | Uqcrb | Macrophages (Mac 1.5) |
| 0.542242 | 0.697 | 0.289 | 2.11E-55 | 18 | Atp5c1 | Macrophages (Mac 1.5) |
| 0.417911 | 0.527 | 0.159 | 2.59E-55 | 18 | Ndufb7 | Macrophages (Mac 1.5) |
| 0.695847 | 0.993 | 0.911 | 6.20E-55 | 18 | Ftl1 | Macrophages (Mac 1.5) |
| 0.652821 | 0.96 | 0.751 | 7.43E-55 | 18 | Rpl14 | Macrophages (Mac 1.5) |
| 0.732041 | 0.903 | 0.582 | 9.43E-55 | 18 | Cox7c | Macrophages (Mac 1.5) |
| 0.724463 | 0.982 | 0.85 | 1.32E-54 | 18 | Rpl32 | Macrophages (Mac 1.5) |
| 0.630439 | 0.982 | 0.792 | 1.96E-53 | 18 | Pfn1 | Macrophages (Mac 1.5) |
| 0.389139 | 0.433 | 0.117 | 3.48E-53 | 18 | Ndufb4 | Macrophages (Mac 1.5) |
| 0.845952 | 0.982 | 0.832 | 5.39E-53 | 18 | Vim | Macrophages (Mac 1.5) |
| 0.531967 | 1 | 0.973 | 6.03E-53 | 18 | Rps29 | Macrophages (Mac 1.5) |
| 0.536541 | 0.534 | 0.18 | 9.90E-53 | 18 | Ranbp1 | Macrophages (Mac 1.5) |
| 0.428745 | 0.588 | 0.2 | 2.16E-52 | 18 | Polr1d | Macrophages (Mac 1.5) |
| 0.387815 | 0.498 | 0.149 | 4.28E-52 | 18 | Ndufc2 | Macrophages (Mac 1.5) |
| 0.684766 | 0.993 | 0.855 | 4.56E-51 | 18 | Rpl36 | Macrophages (Mac 1.5) |
| 0.543825 | 0.881 | 0.426 | 6.11E-51 | 18 | Atox1 | Macrophages (Mac 1.5) |
| 0.623079 | 0.924 | 0.652 | 1.06E-49 | 18 | Rps17 | Macrophages (Mac 1.5) |
| 0.343045 | 0.372 | 0.093 | 1.43E-49 | 18 | Vdac1 | Macrophages (Mac 1.5) |
| 0.307203 | 0.329 | 0.075 | 2.79E-49 | 18 | Cct6a | Macrophages (Mac 1.5) |
| 0.577196 | 0.993 | 0.842 | 3.57E-49 | 18 | Rplp2 | Macrophages (Mac 1.5) |
| 1.01822 | 0.354 | 0.093 | 1.27E-48 | 18 | Ckb | Macrophages (Mac 1.5) |
| 0.25399 | 0.249 | 0.046 | 1.60E-48 | 18 | Pa2g4 | Macrophages (Mac 1.5) |
| 0.513053 | 0.863 | 0.389 | 7.82E-48 | 18 | Fxyd5 | Macrophages (Mac 1.5) |
| 0.54042 | 0.841 | 0.505 | 1.67E-47 | 18 | Slc25a3 | Macrophages (Mac 1.5) |
| 0.316124 | 0.35 | 0.086 | 3.22E-47 | 18 | Ndufv2 | Macrophages (Mac 1.5) |
| 0.643811 | 1 | 0.912 | 3.69E-47 | 18 | Rps27a | Macrophages (Mac 1.5) |
| 0.39972 | 0.451 | 0.135 | 4.28E-47 | 18 | Bola2 | Macrophages (Mac 1.5) |
| 0.320654 | 0.336 | 0.08 | 5.31E-47 | 18 | Itgav | Macrophages (Mac 1.5) |
| 0.547676 | 0.809 | 0.412 | 1.22E-46 | 18 | Ndufb1-ps | Macrophages (Mac 1.5) |
| 0.323148 | 0.321 | 0.075 | 1.83E-46 | 18 | Cited2 | Macrophages (Mac 1.5) |
| 0.57299 | 0.942 | 0.711 | 2.64E-46 | 18 | Rps6 | Macrophages (Mac 1.5) |
| 0.268355 | 0.253 | 0.049 | 2.92E-46 | 18 | Glrx5 | Macrophages (Mac 1.5) |
| 0.326719 | 0.365 | 0.094 | 3.40E-46 | 18 | Ndufs6 | Macrophages (Mac 1.5) |
| 0.386904 | 0.495 | 0.156 | 5.14E-46 | 18 | Pgk1 | Macrophages (Mac 1.5) |
| 0.601869 | 0.935 | 0.681 | 8.12E-46 | 18 | Atp5e | Macrophages (Mac 1.5) |
| 0.557747 | 0.986 | 0.838 | 9.74E-45 | 18 | Rpl26 | Macrophages (Mac 1.5) |
| 0.602361 | 1 | 0.889 | 1.24E-44 | 18 | Rps12 | Macrophages (Mac 1.5) |
| 0.581068 | 0.834 | 0.397 | 2.21E-44 | 18 | Cyba | Macrophages (Mac 1.5) |
| 0.539786 | 0.845 | 0.462 | 4.77E-44 | 18 | Pabpc1 | Macrophages (Mac 1.5) |
| 0.508979 | 0.906 | 0.631 | 1.17E-43 | 18 | Chchd2 | Macrophages (Mac 1.5) |
| 0.477725 | 0.639 | 0.264 | 1.38E-43 | 18 | Atp5mpl | Macrophages (Mac 1.5) |
| 0.263155 | 0.245 | 0.049 | 1.39E-43 | 18 | Sdhd | Macrophages (Mac 1.5) |
| 0.574442 | 0.653 | 0.299 | 2.18E-43 | 18 | Ran | Macrophages (Mac 1.5) |
| 0.366587 | 0.516 | 0.173 | 2.26E-43 | 18 | Vamp8 | Macrophages (Mac 1.5) |
| 0.332694 | 0.379 | 0.105 | 6.25E-43 | 18 | Ndufa5 | Macrophages (Mac 1.5) |
| 0.480798 | 0.599 | 0.248 | 1.38E-42 | 18 | Ndufb9 | Macrophages (Mac 1.5) |
| 2.7185 | 0.617 | 0.358 | 1.47E-42 | 18 | Ctsk | Macrophages (Mac 1.5) |
| 0.481722 | 0.718 | 0.332 | 1.56E-42 | 18 | Ldha | Macrophages (Mac 1.5) |
| 0.32525 | 0.339 | 0.089 | 1.54E-41 | 18 | M6pr | Macrophages (Mac 1.5) |
| 0.596436 | 0.69 | 0.348 | 2.68E-41 | 18 | Atp5md | Macrophages (Mac 1.5) |
| 0.513374 | 0.996 | 0.91 | 4.14E-41 | 18 | Rps16 | Macrophages (Mac 1.5) |
| 0.594678 | 0.996 | 0.914 | 4.87E-41 | 18 | Rps8 | Macrophages (Mac 1.5) |
| 0.327977 | 0.368 | 0.103 | 6.60E-41 | 18 | Micos13 | Macrophages (Mac 1.5) |
| 0.458106 | 0.917 | 0.518 | 6.70E-41 | 18 | Arpc1b | Macrophages (Mac 1.5) |
| 0.390013 | 0.545 | 0.199 | 6.76E-41 | 18 | Psmb5 | Macrophages (Mac 1.5) |
| 0.340565 | 0.415 | 0.126 | 1.17E-40 | 18 | Gpi1 | Macrophages (Mac 1.5) |
| 0.465618 | 0.931 | 0.646 | 2.46E-40 | 18 | Cst3 | Macrophages (Mac 1.5) |
| 0.285416 | 0.282 | 0.064 | 3.63E-40 | 18 | Gpr183 | Macrophages (Mac 1.5) |
| 0.639682 | 0.881 | 0.586 | 4.14E-40 | 18 | Cox6c | Macrophages (Mac 1.5) |
| 0.407049 | 0.578 | 0.221 | 8.16E-40 | 18 | Esd | Macrophages (Mac 1.5) |
| 0.47523 | 0.986 | 0.85 | 1.46E-39 | 18 | Rps13 | Macrophages (Mac 1.5) |
| 0.498398 | 0.906 | 0.608 | 3.28E-39 | 18 | Gapdh | Macrophages (Mac 1.5) |
| 0.559409 | 0.646 | 0.293 | 4.24E-39 | 18 | Mif | Macrophages (Mac 1.5) |
| 0.557433 | 0.989 | 0.862 | 6.55E-39 | 18 | Rps23 | Macrophages (Mac 1.5) |
| 0.563057 | 0.957 | 0.743 | 1.26E-38 | 18 | Rpl22 | Macrophages (Mac 1.5) |
| 0.418326 | 0.682 | 0.305 | 1.54E-38 | 18 | Tomm7 | Macrophages (Mac 1.5) |
| 0.45219 | 0.964 | 0.781 | 1.59E-38 | 18 | Oaz1 | Macrophages (Mac 1.5) |
| 0.693205 | 0.989 | 0.797 | 2.00E-38 | 18 | Rpl35 | Macrophages (Mac 1.5) |
| 0.541072 | 0.545 | 0.228 | 3.76E-38 | 18 | Atp6v1f | Macrophages (Mac 1.5) |
| 0.367393 | 0.516 | 0.186 | 2.26E-37 | 18 | Pitpna | Macrophages (Mac 1.5) |
| 0.541848 | 0.935 | 0.753 | 2.78E-37 | 18 | Rps25 | Macrophages (Mac 1.5) |
| 0.599002 | 0.986 | 0.838 | 2.81E-37 | 18 | Rps26 | Macrophages (Mac 1.5) |
| 0.472036 | 0.91 | 0.631 | 1.11E-36 | 18 | Ybx1 | Macrophages (Mac 1.5) |
| 0.591455 | 0.668 | 0.344 | 1.66E-36 | 18 | Ncl | Macrophages (Mac 1.5) |
| 0.300843 | 0.365 | 0.108 | 1.81E-36 | 18 | Ndufa8 | Macrophages (Mac 1.5) |
| 0.483793 | 0.69 | 0.345 | 2.02E-36 | 18 | Mrpl52 | Macrophages (Mac 1.5) |
| 0.485068 | 1 | 0.917 | 2.88E-36 | 18 | Rpl23 | Macrophages (Mac 1.5) |
| 0.325913 | 0.39 | 0.123 | 1.46E-35 | 18 | Ndufs5 | Macrophages (Mac 1.5) |
| 0.345692 | 0.44 | 0.149 | 2.29E-35 | 18 | Tpi1 | Macrophages (Mac 1.5) |
| 0.25609 | 0.26 | 0.062 | 2.54E-35 | 18 | Ndufa10 | Macrophages (Mac 1.5) |
| 0.501809 | 0.957 | 0.736 | 4.11E-35 | 18 | Rps15 | Macrophages (Mac 1.5) |
| 0.250823 | 0.217 | 0.046 | 6.24E-35 | 18 | Ccdc88a | Macrophages (Mac 1.5) |
| 0.330178 | 0.372 | 0.115 | 3.45E-34 | 18 | Rnf19b | Macrophages (Mac 1.5) |
| 0.447607 | 0.809 | 0.481 | 4.75E-34 | 18 | Atp5h | Macrophages (Mac 1.5) |
| 0.450415 | 0.899 | 0.565 | 5.21E-34 | 18 | Rpl36al | Macrophages (Mac 1.5) |
| 0.294226 | 0.332 | 0.097 | 2.70E-33 | 18 | Nop10 | Macrophages (Mac 1.5) |
| 0.573702 | 0.996 | 0.908 | 3.65E-33 | 18 | Rpl37 | Macrophages (Mac 1.5) |
| 0.481459 | 0.996 | 0.884 | 5.10E-33 | 18 | Rpl27a | Macrophages (Mac 1.5) |
| 0.598997 | 0.924 | 0.634 | 6.09E-33 | 18 | Lmna | Macrophages (Mac 1.5) |
| 0.449303 | 0.975 | 0.852 | 1.12E-32 | 18 | Rps14 | Macrophages (Mac 1.5) |
| 0.448876 | 1 | 0.875 | 2.19E-32 | 18 | Rplp1 | Macrophages (Mac 1.5) |
| 0.333837 | 0.451 | 0.162 | 2.22E-32 | 18 | Eno1 | Macrophages (Mac 1.5) |
| 0.478047 | 0.549 | 0.249 | 4.03E-32 | 18 | Set | Macrophages (Mac 1.5) |
| 0.480313 | 0.996 | 0.902 | 4.11E-32 | 18 | Rpl38 | Macrophages (Mac 1.5) |
| 0.528083 | 0.946 | 0.69 | 5.32E-32 | 18 | Rpl36a | Macrophages (Mac 1.5) |
| 0.336388 | 0.43 | 0.153 | 2.60E-31 | 18 | Ctsa | Macrophages (Mac 1.5) |
| 0.280387 | 0.3 | 0.086 | 6.16E-31 | 18 | Ndufs2 | Macrophages (Mac 1.5) |
| 0.48094 | 0.83 | 0.533 | 6.49E-31 | 18 | Eif5a | Macrophages (Mac 1.5) |
| 0.49732 | 0.996 | 0.862 | 6.59E-31 | 18 | Rpl28 | Macrophages (Mac 1.5) |
| 0.505217 | 0.96 | 0.775 | 7.28E-31 | 18 | Hsp90ab1 | Macrophages (Mac 1.5) |
| 0.427403 | 0.996 | 0.882 | 7.84E-31 | 18 | Rpl19 | Macrophages (Mac 1.5) |
| 0.476737 | 0.805 | 0.528 | 8.07E-31 | 18 | Uqcrh | Macrophages (Mac 1.5) |
| 0.557906 | 0.946 | 0.785 | 9.35E-31 | 18 | Rps2 | Macrophages (Mac 1.5) |
| 0.436887 | 0.715 | 0.395 | 1.03E-30 | 18 | Atp5j | Macrophages (Mac 1.5) |
| 0.450157 | 0.975 | 0.853 | 1.31E-30 | 18 | Rpl8 | Macrophages (Mac 1.5) |
| 0.433733 | 0.773 | 0.447 | 1.47E-30 | 18 | Hint1 | Macrophages (Mac 1.5) |
| 0.451949 | 0.711 | 0.381 | 2.02E-30 | 18 | Serbp1 | Macrophages (Mac 1.5) |
| 0.304924 | 0.408 | 0.143 | 2.46E-30 | 18 | Taf10 | Macrophages (Mac 1.5) |
| 0.421711 | 0.946 | 0.703 | 3.87E-30 | 18 | Naca | Macrophages (Mac 1.5) |
| 0.463681 | 0.989 | 0.898 | 6.83E-30 | 18 | Rpl13 | Macrophages (Mac 1.5) |
| 0.441039 | 0.982 | 0.855 | 6.58E-29 | 18 | Rpl18 | Macrophages (Mac 1.5) |
| 0.381883 | 0.56 | 0.251 | 7.44E-29 | 18 | Ppp1r14b | Macrophages (Mac 1.5) |
| 0.396256 | 0.827 | 0.512 | 1.66E-28 | 18 | Eif4a1 | Macrophages (Mac 1.5) |
| 0.268499 | 0.296 | 0.087 | 1.67E-28 | 18 | Ndufb3 | Macrophages (Mac 1.5) |
| 0.531761 | 0.859 | 0.59 | 2.65E-28 | 18 | Npm1 | Macrophages (Mac 1.5) |
| 0.439012 | 0.978 | 0.763 | 1.35E-27 | 18 | Rpl3 | Macrophages (Mac 1.5) |
| 0.39594 | 0.585 | 0.282 | 1.51E-27 | 18 | Micos10 | Macrophages (Mac 1.5) |
| 0.427259 | 0.928 | 0.683 | 2.24E-27 | 18 | Rpl23a | Macrophages (Mac 1.5) |
| 0.468139 | 0.96 | 0.65 | 5.70E-27 | 18 | H2-D1 | Macrophages (Mac 1.5) |
| 0.393045 | 0.971 | 0.764 | 8.00E-27 | 18 | Rpl12 | Macrophages (Mac 1.5) |
| 0.398993 | 0.437 | 0.175 | 1.12E-26 | 18 | Hexa | Macrophages (Mac 1.5) |
| 0.386967 | 0.996 | 0.969 | 4.54E-26 | 18 | Fau | Macrophages (Mac 1.5) |
| 0.352628 | 0.43 | 0.174 | 8.60E-26 | 18 | Ndufa6 | Macrophages (Mac 1.5) |
| 0.415346 | 0.957 | 0.758 | 1.36E-25 | 18 | Rack1 | Macrophages (Mac 1.5) |
| 0.388758 | 0.971 | 0.796 | 2.61E-25 | 18 | Rpl27 | Macrophages (Mac 1.5) |
| 0.290731 | 0.415 | 0.157 | 1.23E-24 | 18 | Rac2 | Macrophages (Mac 1.5) |
| 0.484208 | 1 | 0.927 | 1.45E-24 | 18 | Rpl37a | Macrophages (Mac 1.5) |
| 0.411076 | 0.993 | 0.856 | 3.97E-24 | 18 | Rps15a | Macrophages (Mac 1.5) |
| 0.348646 | 0.884 | 0.596 | 6.79E-24 | 18 | Rpl4 | Macrophages (Mac 1.5) |
| 0.295163 | 0.466 | 0.195 | 7.65E-24 | 18 | Pgam1 | Macrophages (Mac 1.5) |
| 0.294837 | 0.444 | 0.183 | 1.07E-23 | 18 | Psmd8 | Macrophages (Mac 1.5) |
| 0.320609 | 0.523 | 0.238 | 1.74E-23 | 18 | Nme1 | Macrophages (Mac 1.5) |
| 0.359704 | 0.989 | 0.861 | 1.80E-23 | 18 | Rps11 | Macrophages (Mac 1.5) |
| 0.433351 | 0.978 | 0.843 | 1.99E-23 | 18 | Rpl11 | Macrophages (Mac 1.5) |
| 0.25512 | 0.379 | 0.14 | 2.91E-23 | 18 | Psmb8 | Macrophages (Mac 1.5) |
| 0.352624 | 0.675 | 0.366 | 3.12E-23 | 18 | Arf1 | Macrophages (Mac 1.5) |
| 0.337935 | 0.993 | 0.839 | 5.88E-23 | 18 | Rps7 | Macrophages (Mac 1.5) |
| 0.359637 | 0.971 | 0.847 | 7.85E-23 | 18 | Rps5 | Macrophages (Mac 1.5) |
| 0.375294 | 0.859 | 0.543 | 9.48E-23 | 18 | Anxa1 | Macrophages (Mac 1.5) |
| 0.448548 | 0.899 | 0.638 | 1.90E-22 | 18 | Rps18 | Macrophages (Mac 1.5) |
| 0.363602 | 0.755 | 0.442 | 2.43E-22 | 18 | Rps27l | Macrophages (Mac 1.5) |
| 0.355927 | 0.971 | 0.728 | 5.64E-22 | 18 | Calm1 | Macrophages (Mac 1.5) |
| 0.262013 | 0.477 | 0.2 | 7.13E-22 | 18 | Efhd2 | Macrophages (Mac 1.5) |
| 0.372471 | 0.852 | 0.524 | 8.54E-22 | 18 | H2-K1 | Macrophages (Mac 1.5) |
| 0.311084 | 0.542 | 0.254 | 9.09E-22 | 18 | Hnrnpf | Macrophages (Mac 1.5) |
| 0.626125 | 0.971 | 0.842 | 1.53E-21 | 18 | Ptma | Macrophages (Mac 1.5) |
| 0.41691 | 0.993 | 0.892 | 2.19E-21 | 18 | Rpl35a | Macrophages (Mac 1.5) |
| 0.327747 | 0.643 | 0.341 | 3.40E-21 | 18 | Ndufa2 | Macrophages (Mac 1.5) |
| 0.416591 | 0.986 | 0.909 | 9.09E-21 | 18 | Rps21 | Macrophages (Mac 1.5) |
| 0.337173 | 0.52 | 0.248 | 1.65E-20 | 18 | Zfand5 | Macrophages (Mac 1.5) |
| 0.381235 | 0.993 | 0.858 | 1.81E-20 | 18 | Rpsa | Macrophages (Mac 1.5) |
| 0.261908 | 0.274 | 0.093 | 2.42E-20 | 18 | Rala | Macrophages (Mac 1.5) |
| 0.36588 | 1 | 0.909 | 2.45E-20 | 18 | Rps28 | Macrophages (Mac 1.5) |
| 0.313203 | 0.635 | 0.337 | 3.02E-20 | 18 | Akr1a1 | Macrophages (Mac 1.5) |
| 0.364721 | 0.643 | 0.369 | 4.61E-20 | 18 | Eef1g | Macrophages (Mac 1.5) |
| 0.317405 | 0.888 | 0.655 | 1.53E-19 | 18 | Btf3 | Macrophages (Mac 1.5) |
| 0.267669 | 0.365 | 0.147 | 1.61E-19 | 18 | Ndufa3 | Macrophages (Mac 1.5) |
| 0.328275 | 0.986 | 0.884 | 1.86E-19 | 18 | Rps10 | Macrophages (Mac 1.5) |
| 0.331901 | 0.56 | 0.291 | 1.99E-19 | 18 | Rnasek | Macrophages (Mac 1.5) |
| 0.447168 | 0.661 | 0.397 | 5.35E-19 | 18 | Gm10260 | Macrophages (Mac 1.5) |
| 0.30136 | 0.971 | 0.84 | 7.36E-19 | 18 | Rpl17 | Macrophages (Mac 1.5) |
| 0.374569 | 1 | 0.944 | 8.06E-19 | 18 | Rpl41 | Macrophages (Mac 1.5) |
| 0.353345 | 0.989 | 0.86 | 1.08E-18 | 18 | Rpl6 | Macrophages (Mac 1.5) |
| 0.317231 | 0.238 | 0.077 | 1.68E-18 | 18 | Lpl | Macrophages (Mac 1.5) |
| 0.291555 | 0.903 | 0.668 | 1.79E-18 | 18 | Rpl5 | Macrophages (Mac 1.5) |
| 0.272433 | 0.978 | 0.806 | 2.78E-18 | 18 | Rpl13a | Macrophages (Mac 1.5) |
| 0.288548 | 0.639 | 0.333 | 2.84E-18 | 18 | Ctsz | Macrophages (Mac 1.5) |
| 0.297555 | 0.426 | 0.193 | 6.27E-18 | 18 | Plec | Macrophages (Mac 1.5) |
| 0.348537 | 0.26 | 0.091 | 6.47E-18 | 18 | Stmn1 | Macrophages (Mac 1.5) |
| 0.297388 | 0.968 | 0.838 | 1.58E-17 | 18 | Rps3 | Macrophages (Mac 1.5) |
| 0.263712 | 0.534 | 0.268 | 1.69E-17 | 18 | Psma3 | Macrophages (Mac 1.5) |
| 0.277886 | 0.578 | 0.304 | 2.53E-17 | 18 | Eef1d | Macrophages (Mac 1.5) |
| 0.264839 | 0.368 | 0.157 | 4.25E-17 | 18 | Ywhah | Macrophages (Mac 1.5) |
| 0.344061 | 0.957 | 0.789 | 5.34E-17 | 18 | Rpl10a | Macrophages (Mac 1.5) |
| 0.338771 | 0.657 | 0.4 | 9.26E-17 | 18 | Snrpg | Macrophages (Mac 1.5) |
| 0.301635 | 0.996 | 0.902 | 1.34E-16 | 18 | Rps24 | Macrophages (Mac 1.5) |
| 0.301567 | 0.845 | 0.543 | 1.35E-16 | 18 | Arpc2 | Macrophages (Mac 1.5) |
| 0.282462 | 0.57 | 0.307 | 4.62E-16 | 18 | Nedd8 | Macrophages (Mac 1.5) |
| 0.260172 | 0.469 | 0.228 | 5.70E-16 | 18 | Cox7a2l | Macrophages (Mac 1.5) |
| 0.265896 | 0.993 | 0.872 | 7.73E-15 | 18 | Rpl18a | Macrophages (Mac 1.5) |
| 0.294854 | 0.823 | 0.577 | 1.09E-14 | 18 | Rpl31 | Macrophages (Mac 1.5) |
| 0.294525 | 0.928 | 0.751 | 8.25E-14 | 18 | Rpl7a | Macrophages (Mac 1.5) |
| 0.32753 | 0.989 | 0.868 | 8.52E-14 | 18 | Rplp0 | Macrophages (Mac 1.5) |
| 0.26929 | 0.986 | 0.87 | 8.62E-14 | 18 | Rpl9 | Macrophages (Mac 1.5) |
| 0.280015 | 0.368 | 0.175 | 1.26E-13 | 18 | Ndufa1 | Macrophages (Mac 1.5) |
| 0.266739 | 0.975 | 0.832 | 1.52E-13 | 18 | Rpl21 | Macrophages (Mac 1.5) |
| 0.296756 | 0.968 | 0.833 | 5.51E-13 | 18 | Rpl30 | Macrophages (Mac 1.5) |
| 0.254152 | 0.697 | 0.435 | 6.10E-13 | 18 | Clta | Macrophages (Mac 1.5) |
| 0.418713 | 0.13 | 0.034 | 8.12E-13 | 18 | Hist1h2ap | Macrophages (Mac 1.5) |
| 0.270348 | 0.935 | 0.688 | 8.71E-13 | 18 | Eef1b2 | Macrophages (Mac 1.5) |
| 0.411479 | 0.693 | 0.505 | 2.50E-12 | 18 | Uba52 | Macrophages (Mac 1.5) |
| 0.288917 | 0.993 | 0.885 | 3.71E-12 | 18 | Rps3a1 | Macrophages (Mac 1.5) |
| 0.257982 | 0.408 | 0.21 | 4.41E-12 | 18 | Hnrnpab | Macrophages (Mac 1.5) |
| 0.258963 | 0.895 | 0.689 | 7.57E-12 | 18 | Cfl1 | Macrophages (Mac 1.5) |
| 0.270139 | 0.986 | 0.848 | 9.41E-12 | 18 | Rpl24 | Macrophages (Mac 1.5) |
| 0.319484 | 0.744 | 0.534 | 1.75E-11 | 18 | Emp3 | Macrophages (Mac 1.5) |
| 0.307002 | 0.996 | 0.905 | 6.77E-11 | 18 | Rpl39 | Macrophages (Mac 1.5) |
| 0.275086 | 0.289 | 0.135 | 1.81E-10 | 18 | Tuba1b | Macrophages (Mac 1.5) |
| 0.256149 | 0.466 | 0.261 | 2.35E-10 | 18 | Tomm6 | Macrophages (Mac 1.5) |
| 0.346227 | 0.924 | 0.785 | 2.85E-10 | 18 | mt-Nd1 | Macrophages (Mac 1.5) |
| 0.302864 | 0.993 | 0.872 | 9.15E-10 | 18 | Rpl34 | Macrophages (Mac 1.5) |
| 0.270517 | 0.632 | 0.422 | 2.60E-09 | 18 | Hsp90aa1 | Macrophages (Mac 1.5) |
| 0.254837 | 0.971 | 0.824 | 1.66E-08 | 18 | Rpl15 | Macrophages (Mac 1.5) |
| 0.27005 | 0.931 | 0.796 | 1.60E-05 | 18 | Gm10076 | Macrophages (Mac 1.5) |
| 0.355288 | 0.357 | 0.23 | 0.02266 | 18 | Ifi27l2a | Macrophages (Mac 1.5) |
| 2.760876 | 0.209 | 0.004 | 0 | 19 | Krt14 | Keratinocyte |
| 1.228694 | 0.182 | 0.002 | 0 | 19 | Krt5 | Keratinocyte |
| 1.177186 | 0.198 | 0.002 | 0 | 19 | Lgals7 | Keratinocyte |
| 0.291613 | 0.134 | 0.001 | 0 | 19 | Dsp | Keratinocyte |
| 0.259136 | 0.123 | 0.001 | 0 | 19 | S100a14 | Keratinocyte |
| 0.463857 | 0.17 | 0.003 | 3.10E-289 | 19 | Perp | Keratinocyte |
| 0.751654 | 0.174 | 0.003 | 1.55E-271 | 19 | Sfn | Keratinocyte |
| 0.786203 | 0.146 | 0.002 | 1.40E-267 | 19 | Krt6a | Keratinocyte |
| 0.284423 | 0.146 | 0.003 | 5.20E-208 | 19 | Fxyd3 | Keratinocyte |
| 0.409336 | 0.162 | 0.012 | 2.41E-84 | 19 | Dmkn | Keratinocyte |
| 0.335351 | 0.111 | 0.007 | 8.28E-63 | 19 | Gsta4 | Keratinocyte |
| 0.784952 | 0.976 | 0.862 | 3.58E-52 | 19 | Rpl28 | Keratinocyte |
| 0.855414 | 0.972 | 0.889 | 1.97E-49 | 19 | Rps12 | Keratinocyte |
| 0.776449 | 0.976 | 0.915 | 3.64E-49 | 19 | Rps8 | Keratinocyte |
| 0.742215 | 0.874 | 0.653 | 7.96E-46 | 19 | Rps17 | Keratinocyte |
| 0.744922 | 0.964 | 0.825 | 1.64E-45 | 19 | Rpl15 | Keratinocyte |
| 0.70334 | 0.972 | 0.86 | 1.79E-45 | 19 | Rpl6 | Keratinocyte |
| 0.778558 | 0.984 | 0.944 | 3.44E-45 | 19 | Rpl41 | Keratinocyte |
| 0.734794 | 0.968 | 0.789 | 2.88E-44 | 19 | Rpl10a | Keratinocyte |
| 0.889409 | 0.953 | 0.839 | 4.24E-43 | 19 | Rps26 | Keratinocyte |
| 0.691378 | 0.984 | 0.884 | 7.01E-43 | 19 | Rpl27a | Keratinocyte |
| 0.728054 | 0.96 | 0.851 | 7.21E-43 | 19 | Rpl32 | Keratinocyte |
| 0.701113 | 0.949 | 0.844 | 3.06E-42 | 19 | Rpl11 | Keratinocyte |
| 0.702204 | 0.901 | 0.752 | 6.02E-42 | 19 | Rpl14 | Keratinocyte |
| 0.653099 | 0.984 | 0.913 | 1.43E-40 | 19 | Rps27a | Keratinocyte |
| 0.673075 | 0.968 | 0.857 | 1.90E-39 | 19 | Rps15a | Keratinocyte |
| 0.650614 | 0.953 | 0.863 | 2.92E-39 | 19 | Rps23 | Keratinocyte |
| 0.753927 | 0.941 | 0.798 | 4.86E-39 | 19 | Rpl35 | Keratinocyte |
| 0.639722 | 0.988 | 0.917 | 1.64E-38 | 19 | Rpl23 | Keratinocyte |
| 0.745118 | 0.968 | 0.909 | 8.65E-38 | 19 | Rpl37 | Keratinocyte |
| 0.680845 | 0.889 | 0.744 | 7.00E-37 | 19 | Rpl22 | Keratinocyte |
| 0.640381 | 0.897 | 0.689 | 8.71E-36 | 19 | Eef1b2 | Keratinocyte |
| 0.868499 | 0.921 | 0.796 | 8.87E-35 | 19 | Gm10076 | Keratinocyte |
| 0.570884 | 0.964 | 0.911 | 1.83E-34 | 19 | Rps16 | Keratinocyte |
| 0.588157 | 0.98 | 0.876 | 2.15E-34 | 19 | Rplp1 | Keratinocyte |
| 0.600611 | 0.968 | 0.885 | 4.45E-34 | 19 | Rps3a1 | Keratinocyte |
| 0.583937 | 0.988 | 0.898 | 9.29E-34 | 19 | Rpl13 | Keratinocyte |
| 0.570582 | 0.921 | 0.765 | 1.72E-33 | 19 | Rpl12 | Keratinocyte |
| 0.610399 | 0.941 | 0.833 | 1.72E-33 | 19 | Rpl21 | Keratinocyte |
| 0.68151 | 0.984 | 0.869 | 3.42E-33 | 19 | Rplp0 | Keratinocyte |
| 0.659556 | 0.945 | 0.856 | 4.35E-33 | 19 | Rpl36 | Keratinocyte |
| 0.580801 | 0.893 | 0.737 | 1.16E-32 | 19 | Rps15 | Keratinocyte |
| 0.583791 | 1 | 0.902 | 1.23E-32 | 19 | Rps24 | Keratinocyte |
| 0.545266 | 0.885 | 0.712 | 1.83E-32 | 19 | Rps6 | Keratinocyte |
| 0.563828 | 0.933 | 0.847 | 1.94E-32 | 19 | Rps19 | Keratinocyte |
| 0.565121 | 0.976 | 0.861 | 3.48E-32 | 19 | Rps11 | Keratinocyte |
| 0.798158 | 0.553 | 0.235 | 4.58E-32 | 19 | Mt2 | Keratinocyte |
| 0.565736 | 0.921 | 0.841 | 5.17E-32 | 19 | Rpl17 | Keratinocyte |
| 0.555657 | 0.976 | 0.885 | 1.04E-31 | 19 | Rps10 | Keratinocyte |
| 0.644802 | 0.96 | 0.873 | 1.85E-31 | 19 | Rpl34 | Keratinocyte |
| 0.663045 | 0.976 | 0.872 | 2.69E-31 | 19 | Rps20 | Keratinocyte |
| 0.586103 | 0.98 | 0.909 | 3.56E-31 | 19 | Rps21 | Keratinocyte |
| 0.554055 | 0.941 | 0.839 | 3.88E-31 | 19 | Rpl26 | Keratinocyte |
| 0.631747 | 0.976 | 0.906 | 5.75E-31 | 19 | Rpl39 | Keratinocyte |
| 0.570774 | 0.953 | 0.855 | 7.80E-31 | 19 | Rpl18 | Keratinocyte |
| 0.518671 | 0.921 | 0.797 | 8.70E-31 | 19 | Rpl27 | Keratinocyte |
| 0.557514 | 0.917 | 0.844 | 1.27E-30 | 19 | Rplp2 | Keratinocyte |
| 0.524442 | 0.897 | 0.752 | 2.07E-30 | 19 | Rpl7a | Keratinocyte |
| 0.660087 | 0.945 | 0.839 | 4.68E-30 | 19 | Ppia | Keratinocyte |
| 0.57895 | 0.941 | 0.84 | 7.26E-30 | 19 | Rps7 | Keratinocyte |
| 0.542539 | 0.957 | 0.838 | 7.41E-30 | 19 | Rps3 | Keratinocyte |
| 0.661112 | 0.842 | 0.692 | 1.03E-29 | 19 | Rpl36a | Keratinocyte |
| 0.546677 | 0.953 | 0.847 | 1.06E-29 | 19 | Rps5 | Keratinocyte |
| 0.45646 | 0.534 | 0.225 | 1.62E-29 | 19 | Hspe1 | Keratinocyte |
| 0.523603 | 0.945 | 0.849 | 2.82E-29 | 19 | Rpl24 | Keratinocyte |
| 0.528268 | 0.957 | 0.851 | 2.89E-29 | 19 | Rps13 | Keratinocyte |
| 0.638933 | 0.945 | 0.785 | 4.30E-29 | 19 | Rps2 | Keratinocyte |
| 0.557195 | 0.597 | 0.294 | 1.70E-28 | 19 | Mif | Keratinocyte |
| 0.521592 | 0.976 | 0.91 | 1.82E-28 | 19 | Rps28 | Keratinocyte |
| 0.563455 | 0.976 | 0.859 | 2.06E-28 | 19 | Rpsa | Keratinocyte |
| 0.500731 | 0.968 | 0.883 | 3.81E-28 | 19 | Rpl19 | Keratinocyte |
| 0.63247 | 0.767 | 0.504 | 9.34E-28 | 19 | Uba52 | Keratinocyte |
| 0.50328 | 0.929 | 0.853 | 1.03E-27 | 19 | Rps14 | Keratinocyte |
| 0.591942 | 0.917 | 0.834 | 1.34E-27 | 19 | Rpl30 | Keratinocyte |
| 0.573232 | 0.862 | 0.755 | 1.73E-27 | 19 | Rps25 | Keratinocyte |
| 0.496978 | 0.964 | 0.873 | 3.33E-27 | 19 | Rpl18a | Keratinocyte |
| 0.556192 | 0.937 | 0.893 | 1.23E-26 | 19 | Rpl35a | Keratinocyte |
| 0.544439 | 0.921 | 0.765 | 1.69E-26 | 19 | Rpl3 | Keratinocyte |
| 0.533851 | 0.538 | 0.251 | 2.38E-26 | 19 | Ppp1r14b | Keratinocyte |
| 0.544952 | 0.85 | 0.684 | 3.90E-26 | 19 | Rpl23a | Keratinocyte |
| 0.717617 | 0.838 | 0.64 | 6.78E-26 | 19 | Rps18 | Keratinocyte |
| 0.496539 | 0.976 | 0.903 | 6.87E-26 | 19 | Rpl38 | Keratinocyte |
| 0.535039 | 0.763 | 0.578 | 1.09E-25 | 19 | Rpl31 | Keratinocyte |
| 0.544025 | 0.964 | 0.846 | 1.74E-25 | 19 | Rps4x | Keratinocyte |
| 0.515436 | 0.988 | 0.927 | 3.88E-25 | 19 | Rpl37a | Keratinocyte |
| 0.440431 | 0.921 | 0.807 | 6.43E-25 | 19 | Rpl13a | Keratinocyte |
| 0.611819 | 0.806 | 0.591 | 2.20E-24 | 19 | Npm1 | Keratinocyte |
| 0.366279 | 0.439 | 0.181 | 4.60E-23 | 19 | Snrpf | Keratinocyte |
| 0.892094 | 0.747 | 0.469 | 1.03E-22 | 19 | Mt1 | Keratinocyte |
| 0.498845 | 0.854 | 0.705 | 3.51E-22 | 19 | Naca | Keratinocyte |
| 0.49418 | 0.925 | 0.816 | 9.24E-22 | 19 | Rpl7 | Keratinocyte |
| 0.443796 | 0.945 | 0.854 | 1.55E-21 | 19 | Rpl8 | Keratinocyte |
| 0.961991 | 0.312 | 0.113 | 4.51E-21 | 19 | Hspb1 | Keratinocyte |
| 0.37159 | 0.482 | 0.219 | 5.25E-21 | 19 | Snrpe | Keratinocyte |
| 0.474934 | 0.826 | 0.669 | 9.95E-21 | 19 | Rpl5 | Keratinocyte |
| 0.383679 | 0.542 | 0.269 | 2.85E-20 | 19 | Cox7b | Keratinocyte |
| 0.441818 | 0.984 | 0.929 | 6.57E-20 | 19 | Eef1a1 | Keratinocyte |
| 0.434981 | 0.941 | 0.871 | 4.23E-19 | 19 | Rpl9 | Keratinocyte |
| 0.415991 | 0.909 | 0.759 | 1.49E-18 | 19 | Rack1 | Keratinocyte |
| 0.554664 | 0.625 | 0.398 | 1.62E-18 | 19 | Gm10260 | Keratinocyte |
| 0.473051 | 0.68 | 0.444 | 1.99E-18 | 19 | Rps27l | Keratinocyte |
| 0.435556 | 0.625 | 0.378 | 9.07E-18 | 19 | Atpif1 | Keratinocyte |
| 0.381184 | 0.834 | 0.656 | 1.20E-17 | 19 | Btf3 | Keratinocyte |
| 0.513813 | 0.929 | 0.843 | 3.71E-17 | 19 | Ptma | Keratinocyte |
| 0.257316 | 0.277 | 0.098 | 5.55E-17 | 19 | Snrpd1 | Keratinocyte |
| 0.481667 | 0.739 | 0.569 | 9.29E-17 | 19 | Rpl36al | Keratinocyte |
| 0.543334 | 0.704 | 0.485 | 9.35E-17 | 19 | Gas5 | Keratinocyte |
| 0.485345 | 0.771 | 0.601 | 3.49E-15 | 19 | Txn1 | Keratinocyte |
| 0.753653 | 0.138 | 0.034 | 1.49E-14 | 19 | Hist1h2ap | Keratinocyte |
| 0.341281 | 0.581 | 0.34 | 2.02E-14 | 19 | Atp5g1 | Keratinocyte |
| 0.302263 | 0.142 | 0.036 | 2.59E-14 | 19 | Igfbp2 | Keratinocyte |
| 0.459882 | 0.755 | 0.569 | 4.62E-14 | 19 | Rpl22l1 | Keratinocyte |
| 0.339443 | 0.19 | 0.06 | 5.84E-14 | 19 | Fam162a | Keratinocyte |
| 0.308782 | 0.542 | 0.305 | 3.73E-13 | 19 | Eef1d | Keratinocyte |
| 0.371469 | 0.617 | 0.401 | 4.65E-13 | 19 | Snrpg | Keratinocyte |
| 0.325051 | 0.431 | 0.22 | 6.48E-13 | 19 | Anp32b | Keratinocyte |
| 0.300226 | 0.49 | 0.263 | 7.55E-13 | 19 | Uqcr11 | Keratinocyte |
| 0.377724 | 0.652 | 0.45 | 1.01E-12 | 19 | Hint1 | Keratinocyte |
| 0.341715 | 0.605 | 0.37 | 1.17E-12 | 19 | Eef1g | Keratinocyte |
| 0.398085 | 0.783 | 0.588 | 1.21E-12 | 19 | Cox6c | Keratinocyte |
| 0.467607 | 0.87 | 0.712 | 1.41E-12 | 19 | Nme2 | Keratinocyte |
| 0.305522 | 0.96 | 0.95 | 1.54E-12 | 19 | Rps9 | Keratinocyte |
| 0.379151 | 0.581 | 0.363 | 2.46E-12 | 19 | Cox5b | Keratinocyte |
| 0.386389 | 0.617 | 0.404 | 2.70E-12 | 19 | Ndufa4 | Keratinocyte |
| 0.362644 | 0.696 | 0.485 | 5.73E-12 | 19 | Atp5g2 | Keratinocyte |
| 0.432659 | 0.85 | 0.777 | 5.97E-12 | 19 | Hsp90ab1 | Keratinocyte |
| 0.358026 | 0.518 | 0.302 | 7.23E-12 | 19 | Ran | Keratinocyte |
| 0.406799 | 0.735 | 0.585 | 1.07E-11 | 19 | Cox7c | Keratinocyte |
| 0.25318 | 0.237 | 0.092 | 1.66E-11 | 19 | Erh | Keratinocyte |
| 0.329189 | 0.787 | 0.544 | 1.66E-11 | 19 | Rpl29 | Keratinocyte |
| 0.328846 | 0.538 | 0.316 | 2.90E-11 | 19 | Dbi | Keratinocyte |
| 0.35922 | 0.798 | 0.671 | 3.01E-11 | 19 | Cox8a | Keratinocyte |
| 0.316105 | 0.692 | 0.484 | 4.93E-11 | 19 | Atp5h | Keratinocyte |
| 0.430617 | 0.676 | 0.496 | 7.09E-11 | 19 | Hmgb1 | Keratinocyte |
| 0.375345 | 0.751 | 0.584 | 9.41E-11 | 19 | Prdx1 | Keratinocyte |
| 0.305913 | 0.506 | 0.289 | 1.02E-10 | 19 | Hmgn1 | Keratinocyte |
| 0.278471 | 0.399 | 0.207 | 1.51E-10 | 19 | Snrpd2 | Keratinocyte |
| 0.26152 | 0.265 | 0.113 | 2.01E-10 | 19 | Snhg1 | Keratinocyte |
| 0.31376 | 0.755 | 0.599 | 2.10E-10 | 19 | Rpl4 | Keratinocyte |
| 0.295916 | 0.455 | 0.251 | 2.93E-10 | 19 | Set | Keratinocyte |
| 0.286261 | 0.601 | 0.383 | 4.81E-10 | 19 | Serbp1 | Keratinocyte |
| 0.30729 | 0.96 | 0.944 | 6.62E-10 | 19 | Rps27 | Keratinocyte |
| 0.325647 | 0.715 | 0.536 | 5.46E-09 | 19 | Eif5a | Keratinocyte |
| 0.306861 | 0.553 | 0.363 | 2.72E-08 | 19 | Cox7a2 | Keratinocyte |
| 0.281781 | 0.617 | 0.423 | 3.36E-08 | 19 | Atp5j2 | Keratinocyte |
| 0.251664 | 0.312 | 0.152 | 4.23E-08 | 19 | Crip2 | Keratinocyte |
| 0.274334 | 0.597 | 0.397 | 5.01E-08 | 19 | Atp5b | Keratinocyte |
| 0.253994 | 0.462 | 0.274 | 5.46E-08 | 19 | Pebp1 | Keratinocyte |
| 0.279236 | 0.466 | 0.282 | 5.81E-08 | 19 | Atp5o | Keratinocyte |
| 0.303488 | 0.901 | 0.828 | 2.01E-07 | 19 | Rpl10 | Keratinocyte |
| 0.33067 | 0.751 | 0.622 | 2.29E-07 | 19 | Rbm3 | Keratinocyte |
| 0.280119 | 0.443 | 0.266 | 3.83E-07 | 19 | Uqcrb | Keratinocyte |
| 0.290549 | 0.526 | 0.347 | 8.00E-07 | 19 | Ncl | Keratinocyte |
| 0.34414 | 0.364 | 0.215 | 1.75E-06 | 19 | Txndc17 | Keratinocyte |
| 0.407022 | 0.715 | 0.618 | 1.99E-06 | 19 | Sec61g | Keratinocyte |
| 0.255499 | 0.379 | 0.218 | 2.33E-06 | 19 | Cnbp | Keratinocyte |
| 0.29095 | 0.455 | 0.283 | 3.47E-06 | 19 | 2410006H16Rik | Keratinocyte |
| 0.335302 | 0.7 | 0.571 | 4.04E-06 | 19 | Elob | Keratinocyte |
| 0.294391 | 0.66 | 0.531 | 5.61E-06 | 19 | Uqcrh | Keratinocyte |
| 0.484688 | 0.889 | 0.853 | 1.23E-05 | 19 | S100a6 | Keratinocyte |
| 0.256739 | 0.474 | 0.312 | 3.08E-05 | 19 | Sfr1 | Keratinocyte |
| 0.302859 | 0.727 | 0.634 | 3.18E-05 | 19 | Ybx1 | Keratinocyte |
| 0.297225 | 0.688 | 0.556 | 0.000108 | 19 | Atp5l | Keratinocyte |
| 0.269649 | 0.692 | 0.561 | 0.001488 | 19 | Dynll1 | Keratinocyte |
| 0.60003 | 0.759 | 0.735 | 0.004346 | 19 | Tmsb10 | Keratinocyte |
| 0.445081 | 0.253 | 0.151 | 0.004346 | 19 | AY036118 | Keratinocyte |
| 0.258355 | 0.462 | 0.336 | 0.007221 | 19 | Atp5k | Keratinocyte |
| 0.407945 | 0.569 | 0.506 | 0.010548 | 19 | Sec61b | Keratinocyte |
| 0.312507 | 0.34 | 0.214 | 0.015508 | 19 | Ccl2 | Keratinocyte |
| 0.303673 | 0.289 | 0.173 | 0.020904 | 19 | Ccl7 | Keratinocyte |
| 0.408825 | 0.316 | 0.209 | 0.022958 | 19 | Rbp1 | Keratinocyte |
| 2.677116 | 0.984 | 0.131 | 0 | 20 | Sparcl1 | Smooth Muscle Cells |
| 2.640653 | 0.899 | 0.071 | 0 | 20 | Mustn1 | Smooth Muscle Cells |
| 2.316317 | 0.846 | 0.08 | 0 | 20 | Gm13889 | Smooth Muscle Cells |
| 2.315834 | 0.899 | 0.02 | 0 | 20 | Myh11 | Smooth Muscle Cells |
| 1.721244 | 0.822 | 0.024 | 0 | 20 | Sncg | Smooth Muscle Cells |
| 1.666667 | 0.895 | 0.082 | 0 | 20 | Mylk | Smooth Muscle Cells |
| 1.258324 | 0.773 | 0.07 | 0 | 20 | Filip1l | Smooth Muscle Cells |
| 1.23939 | 0.7 | 0.006 | 0 | 20 | Sorbs2 | Smooth Muscle Cells |
| 1.233634 | 0.721 | 0.007 | 0 | 20 | Pcp4l1 | Smooth Muscle Cells |
| 1.088928 | 0.753 | 0.08 | 0 | 20 | Ppp1r12a | Smooth Muscle Cells |
| 1.085531 | 0.583 | 0.003 | 0 | 20 | Pln | Smooth Muscle Cells |
| 0.955387 | 0.623 | 0.016 | 0 | 20 | Ppp1r14a | Smooth Muscle Cells |
| 0.936142 | 0.652 | 0.038 | 0 | 20 | Ptp4a3 | Smooth Muscle Cells |
| 0.870917 | 0.664 | 0.054 | 0 | 20 | Notch3 | Smooth Muscle Cells |
| 0.799226 | 0.591 | 0.02 | 0 | 20 | Gucy1a1 | Smooth Muscle Cells |
| 0.797395 | 0.623 | 0.026 | 0 | 20 | Tinagl1 | Smooth Muscle Cells |
| 0.793633 | 0.628 | 0.042 | 0 | 20 | Rbpms | Smooth Muscle Cells |
| 0.789051 | 0.636 | 0.031 | 0 | 20 | Epas1 | Smooth Muscle Cells |
| 0.788177 | 0.575 | 0.026 | 0 | 20 | Map3k20 | Smooth Muscle Cells |
| 0.769726 | 0.401 | 0.008 | 0 | 20 | Rasd1 | Smooth Muscle Cells |
| 0.750665 | 0.551 | 0.005 | 0 | 20 | Bcam | Smooth Muscle Cells |
| 0.743368 | 0.538 | 0.006 | 0 | 20 | Lmod1 | Smooth Muscle Cells |
| 0.722817 | 0.498 | 0.021 | 0 | 20 | Crim1 | Smooth Muscle Cells |
| 0.715019 | 0.547 | 0.02 | 0 | 20 | Pde3a | Smooth Muscle Cells |
| 0.676727 | 0.51 | 0.022 | 0 | 20 | Wtip | Smooth Muscle Cells |
| 0.615431 | 0.482 | 0.018 | 0 | 20 | Aoc3 | Smooth Muscle Cells |
| 0.606969 | 0.393 | 0.014 | 0 | 20 | Rgs4 | Smooth Muscle Cells |
| 0.602394 | 0.445 | 0.009 | 0 | 20 | Tesc | Smooth Muscle Cells |
| 0.592543 | 0.417 | 0.006 | 0 | 20 | Cnn1 | Smooth Muscle Cells |
| 0.590211 | 0.417 | 0.003 | 0 | 20 | Rcan2 | Smooth Muscle Cells |
| 0.577096 | 0.437 | 0.009 | 0 | 20 | Ppp1r12b | Smooth Muscle Cells |
| 0.46333 | 0.312 | 0.004 | 0 | 20 | Fbxl22 | Smooth Muscle Cells |
| 0.462354 | 0.352 | 0.006 | 0 | 20 | Synm | Smooth Muscle Cells |
| 0.456166 | 0.336 | 0.008 | 0 | 20 | Nexn | Smooth Muscle Cells |
| 0.429691 | 0.32 | 0.009 | 0 | 20 | Rgs7bp | Smooth Muscle Cells |
| 0.399223 | 0.308 | 0.009 | 0 | 20 | Rbpms2 | Smooth Muscle Cells |
| 0.381908 | 0.287 | 0.003 | 0 | 20 | Synpo2 | Smooth Muscle Cells |
| 0.373373 | 0.271 | 0.006 | 0 | 20 | Olfr558 | Smooth Muscle Cells |
| 0.373136 | 0.263 | 0.003 | 0 | 20 | Nrip2 | Smooth Muscle Cells |
| 0.369391 | 0.182 | 0.002 | 0 | 20 | Avpr1a | Smooth Muscle Cells |
| 0.364213 | 0.271 | 0.003 | 0 | 20 | Pdlim3 | Smooth Muscle Cells |
| 0.335307 | 0.255 | 0.004 | 0 | 20 | Ttll7 | Smooth Muscle Cells |
| 0.321104 | 0.231 | 0.001 | 0 | 20 | Npy1r | Smooth Muscle Cells |
| 0.273643 | 0.198 | 0.001 | 0 | 20 | Gm13861 | Smooth Muscle Cells |
| 0.255066 | 0.182 | 0.001 | 0 | 20 | Susd5 | Smooth Muscle Cells |
| 1.219135 | 0.765 | 0.087 | 6.30E-296 | 20 | Ckb | Smooth Muscle Cells |
| 0.610194 | 0.486 | 0.033 | 1.90E-279 | 20 | Lamb2 | Smooth Muscle Cells |
| 0.988462 | 0.721 | 0.083 | 1.54E-266 | 20 | Fxyd1 | Smooth Muscle Cells |
| 0.715379 | 0.474 | 0.033 | 5.73E-265 | 20 | Des | Smooth Muscle Cells |
| 0.30474 | 0.223 | 0.006 | 7.18E-265 | 20 | Ntn4 | Smooth Muscle Cells |
| 0.693701 | 0.575 | 0.05 | 3.37E-263 | 20 | Zfhx3 | Smooth Muscle Cells |
| 2.615365 | 0.923 | 0.185 | 4.74E-259 | 20 | Myl9 | Smooth Muscle Cells |
| 0.410778 | 0.328 | 0.015 | 6.95E-257 | 20 | Cystm1 | Smooth Muscle Cells |
| 0.33583 | 0.259 | 0.009 | 5.09E-256 | 20 | Pde5a | Smooth Muscle Cells |
| 0.413185 | 0.263 | 0.009 | 5.58E-254 | 20 | Gja4 | Smooth Muscle Cells |
| 0.558413 | 0.449 | 0.033 | 1.96E-242 | 20 | Tns1 | Smooth Muscle Cells |
| 0.447926 | 0.32 | 0.016 | 2.87E-242 | 20 | Kitl | Smooth Muscle Cells |
| 1.22307 | 0.381 | 0.024 | 2.47E-238 | 20 | Rasl11a | Smooth Muscle Cells |
| 3.526502 | 0.927 | 0.21 | 8.20E-236 | 20 | Tagln | Smooth Muscle Cells |
| 1.183567 | 0.757 | 0.106 | 3.96E-229 | 20 | Hspb1 | Smooth Muscle Cells |
| 1.649178 | 0.644 | 0.08 | 1.47E-228 | 20 | Crispld2 | Smooth Muscle Cells |
| 0.716071 | 0.607 | 0.067 | 3.55E-223 | 20 | Rras | Smooth Muscle Cells |
| 0.371929 | 0.287 | 0.014 | 6.82E-214 | 20 | Gucy1b1 | Smooth Muscle Cells |
| 1.245903 | 0.83 | 0.149 | 3.42E-211 | 20 | Csrp1 | Smooth Muscle Cells |
| 0.35197 | 0.235 | 0.009 | 5.10E-210 | 20 | Pi15 | Smooth Muscle Cells |
| 2.752928 | 0.947 | 0.276 | 1.13E-201 | 20 | Tpm2 | Smooth Muscle Cells |
| 0.288912 | 0.223 | 0.009 | 4.59E-195 | 20 | Sorbs1 | Smooth Muscle Cells |
| 0.606713 | 0.482 | 0.048 | 2.55E-191 | 20 | Nr2f2 | Smooth Muscle Cells |
| 0.319427 | 0.263 | 0.013 | 2.59E-191 | 20 | Ccdc3 | Smooth Muscle Cells |
| 0.288499 | 0.186 | 0.006 | 2.18E-188 | 20 | Kcne4 | Smooth Muscle Cells |
| 0.598072 | 0.47 | 0.047 | 2.10E-185 | 20 | Utrn | Smooth Muscle Cells |
| 0.875675 | 0.66 | 0.096 | 6.29E-183 | 20 | Mef2c | Smooth Muscle Cells |
| 1.560494 | 0.664 | 0.107 | 9.04E-182 | 20 | Adamts1 | Smooth Muscle Cells |
| 0.279702 | 0.219 | 0.009 | 2.84E-181 | 20 | Atp1b2 | Smooth Muscle Cells |
| 3.147721 | 0.931 | 0.303 | 4.01E-175 | 20 | Acta2 | Smooth Muscle Cells |
| 0.251061 | 0.178 | 0.006 | 2.99E-174 | 20 | Tbx2 | Smooth Muscle Cells |
| 0.53707 | 0.425 | 0.041 | 2.47E-172 | 20 | Lbh | Smooth Muscle Cells |
| 0.883394 | 0.737 | 0.131 | 1.49E-171 | 20 | Cavin1 | Smooth Muscle Cells |
| 1.378598 | 0.879 | 0.235 | 7.39E-171 | 20 | Flna | Smooth Muscle Cells |
| 0.306935 | 0.243 | 0.013 | 7.66E-170 | 20 | Arhgap29 | Smooth Muscle Cells |
| 1.717296 | 0.907 | 0.276 | 1.43E-168 | 20 | Dstn | Smooth Muscle Cells |
| 0.922756 | 0.713 | 0.12 | 1.87E-168 | 20 | Gng11 | Smooth Muscle Cells |
| 0.439226 | 0.316 | 0.023 | 5.29E-165 | 20 | S1pr3 | Smooth Muscle Cells |
| 0.808923 | 0.526 | 0.066 | 2.84E-164 | 20 | Ndufa4l2 | Smooth Muscle Cells |
| 0.52014 | 0.429 | 0.044 | 1.74E-162 | 20 | Map1b | Smooth Muscle Cells |
| 0.299001 | 0.235 | 0.013 | 2.69E-162 | 20 | Hspb2 | Smooth Muscle Cells |
| 0.352237 | 0.3 | 0.021 | 1.34E-160 | 20 | Parm1 | Smooth Muscle Cells |
| 1.914505 | 0.972 | 0.376 | 2.09E-159 | 20 | Tpm1 | Smooth Muscle Cells |
| 0.896451 | 0.676 | 0.119 | 5.94E-159 | 20 | Atp2a2 | Smooth Muscle Cells |
| 0.302274 | 0.239 | 0.013 | 1.62E-157 | 20 | Pde1a | Smooth Muscle Cells |
| 0.564828 | 0.478 | 0.058 | 1.27E-151 | 20 | Mgst3 | Smooth Muscle Cells |
| 0.953634 | 0.684 | 0.123 | 3.59E-151 | 20 | Timp3 | Smooth Muscle Cells |
| 0.668613 | 0.555 | 0.08 | 6.09E-151 | 20 | Ppp1cb | Smooth Muscle Cells |
| 0.832742 | 0.704 | 0.138 | 8.34E-147 | 20 | Rock1 | Smooth Muscle Cells |
| 0.358922 | 0.287 | 0.022 | 8.51E-147 | 20 | Lgalsl | Smooth Muscle Cells |
| 0.451393 | 0.397 | 0.041 | 3.73E-146 | 20 | Ramp1 | Smooth Muscle Cells |
| 1.358045 | 0.522 | 0.078 | 1.52E-143 | 20 | Rrad | Smooth Muscle Cells |
| 0.478341 | 0.385 | 0.04 | 8.11E-142 | 20 | Pls3 | Smooth Muscle Cells |
| 0.848689 | 0.709 | 0.146 | 2.84E-136 | 20 | Crip2 | Smooth Muscle Cells |
| 0.30449 | 0.251 | 0.018 | 3.48E-134 | 20 | Fam241a | Smooth Muscle Cells |
| 0.444848 | 0.328 | 0.031 | 7.57E-134 | 20 | Cox4i2 | Smooth Muscle Cells |
| 0.481303 | 0.401 | 0.047 | 4.48E-131 | 20 | Gjc1 | Smooth Muscle Cells |
| 0.402405 | 0.251 | 0.018 | 1.85E-130 | 20 | Apold1 | Smooth Muscle Cells |
| 0.453832 | 0.372 | 0.041 | 3.51E-130 | 20 | Mcam | Smooth Muscle Cells |
| 0.708056 | 0.603 | 0.108 | 3.22E-128 | 20 | Cpe | Smooth Muscle Cells |
| 0.481841 | 0.417 | 0.053 | 1.80E-124 | 20 | Pbxip1 | Smooth Muscle Cells |
| 0.975264 | 0.741 | 0.184 | 2.14E-123 | 20 | Map1lc3a | Smooth Muscle Cells |
| 0.615358 | 0.478 | 0.071 | 5.93E-121 | 20 | 4930523C07Rik | Smooth Muscle Cells |
| 0.310518 | 0.231 | 0.017 | 6.66E-119 | 20 | Itga8 | Smooth Muscle Cells |
| 1.972691 | 0.98 | 0.821 | 7.79E-119 | 20 | Myl6 | Smooth Muscle Cells |
| 0.771704 | 0.64 | 0.136 | 8.63E-118 | 20 | Lpp | Smooth Muscle Cells |
| 1.524425 | 0.996 | 0.869 | 1.02E-117 | 20 | mt-Cytb | Smooth Muscle Cells |
| 0.270619 | 0.194 | 0.012 | 5.30E-117 | 20 | Usp2 | Smooth Muscle Cells |
| 0.456289 | 0.3 | 0.03 | 3.74E-112 | 20 | Higd1b | Smooth Muscle Cells |
| 0.454703 | 0.409 | 0.057 | 2.76E-110 | 20 | Esyt2 | Smooth Muscle Cells |
| 1.574709 | 0.988 | 0.499 | 3.77E-110 | 20 | Igfbp7 | Smooth Muscle Cells |
| 0.865594 | 0.417 | 0.061 | 1.04E-109 | 20 | Adamts4 | Smooth Muscle Cells |
| 2.219145 | 0.992 | 0.77 | 6.56E-109 | 20 | Crip1 | Smooth Muscle Cells |
| 0.339968 | 0.3 | 0.031 | 1.41E-108 | 20 | Uba2 | Smooth Muscle Cells |
| 0.808501 | 0.725 | 0.199 | 3.59E-102 | 20 | Mfge8 | Smooth Muscle Cells |
| 0.255612 | 0.211 | 0.016 | 1.75E-101 | 20 | Ccdc107 | Smooth Muscle Cells |
| 1.200965 | 0.976 | 0.465 | 4.46E-101 | 20 | Cald1 | Smooth Muscle Cells |
| 0.275919 | 0.219 | 0.018 | 1.04E-100 | 20 | Stk38l | Smooth Muscle Cells |
| 0.342042 | 0.287 | 0.031 | 1.48E-99 | 20 | Mapre2 | Smooth Muscle Cells |
| 1.78581 | 0.486 | 0.085 | 8.73E-98 | 20 | Rgs5 | Smooth Muscle Cells |
| 1.226805 | 0.98 | 0.668 | 1.06E-97 | 20 | mt-Nd2 | Smooth Muscle Cells |
| 0.532968 | 0.559 | 0.111 | 1.91E-97 | 20 | Rarres2 | Smooth Muscle Cells |
| 0.481634 | 0.462 | 0.08 | 4.65E-97 | 20 | Hcfc1r1 | Smooth Muscle Cells |
| 1.240443 | 0.996 | 0.925 | 5.32E-96 | 20 | mt-Atp6 | Smooth Muscle Cells |
| 1.034456 | 0.652 | 0.156 | 9.00E-96 | 20 | Igfbp5 | Smooth Muscle Cells |
| 0.276646 | 0.227 | 0.02 | 4.16E-95 | 20 | Marveld1 | Smooth Muscle Cells |
| 0.288273 | 0.219 | 0.019 | 8.56E-95 | 20 | Cspg4 | Smooth Muscle Cells |
| 1.151457 | 0.984 | 0.803 | 3.74E-94 | 20 | mt-Nd4 | Smooth Muscle Cells |
| 1.092257 | 0.996 | 0.927 | 3.48E-92 | 20 | mt-Co2 | Smooth Muscle Cells |
| 0.796145 | 0.623 | 0.157 | 4.40E-91 | 20 | Palld | Smooth Muscle Cells |
| 0.551041 | 0.599 | 0.136 | 4.92E-91 | 20 | Fermt2 | Smooth Muscle Cells |
| 0.538757 | 0.453 | 0.082 | 6.61E-90 | 20 | Dst | Smooth Muscle Cells |
| 0.300961 | 0.198 | 0.017 | 4.10E-89 | 20 | Txndc11 | Smooth Muscle Cells |
| 0.331542 | 0.291 | 0.035 | 1.26E-88 | 20 | Cd151 | Smooth Muscle Cells |
| 0.494972 | 0.453 | 0.083 | 1.67E-88 | 20 | Aopep | Smooth Muscle Cells |
| 0.279153 | 0.215 | 0.02 | 3.84E-88 | 20 | Foxs1 | Smooth Muscle Cells |
| 0.286972 | 0.215 | 0.02 | 2.55E-87 | 20 | Smtn | Smooth Muscle Cells |
| 1.521586 | 0.826 | 0.338 | 3.19E-86 | 20 | Id3 | Smooth Muscle Cells |
| 0.329171 | 0.283 | 0.035 | 4.49E-86 | 20 | Limd1 | Smooth Muscle Cells |
| 0.298727 | 0.211 | 0.02 | 1.27E-85 | 20 | Barx1 | Smooth Muscle Cells |
| 0.251142 | 0.202 | 0.018 | 2.28E-85 | 20 | Esam | Smooth Muscle Cells |
| 0.472894 | 0.271 | 0.033 | 1.41E-84 | 20 | Actg2 | Smooth Muscle Cells |
| 0.823929 | 0.741 | 0.245 | 1.90E-84 | 20 | Cavin3 | Smooth Muscle Cells |
| 0.611142 | 0.599 | 0.15 | 4.24E-84 | 20 | Rap1a | Smooth Muscle Cells |
| 1.063668 | 0.996 | 0.927 | 2.44E-80 | 20 | mt-Co3 | Smooth Muscle Cells |
| 0.318779 | 0.263 | 0.032 | 3.10E-80 | 20 | Lrrc32 | Smooth Muscle Cells |
| 0.339047 | 0.291 | 0.04 | 2.39E-78 | 20 | Enah | Smooth Muscle Cells |
| 0.619576 | 0.615 | 0.159 | 2.80E-78 | 20 | Prss23 | Smooth Muscle Cells |
| 0.697612 | 0.737 | 0.22 | 6.92E-78 | 20 | Tm4sf1 | Smooth Muscle Cells |
| 0.438916 | 0.413 | 0.077 | 4.23E-77 | 20 | Mprip | Smooth Muscle Cells |
| 0.532643 | 0.534 | 0.125 | 1.77E-76 | 20 | Ccnd2 | Smooth Muscle Cells |
| 0.33208 | 0.275 | 0.037 | 1.43E-74 | 20 | Ednra | Smooth Muscle Cells |
| 0.529721 | 0.522 | 0.122 | 1.35E-73 | 20 | Pdgfa | Smooth Muscle Cells |
| 0.895412 | 0.696 | 0.24 | 2.55E-71 | 20 | Tsc22d1 | Smooth Muscle Cells |
| 0.637332 | 0.725 | 0.233 | 1.95E-70 | 20 | Klf9 | Smooth Muscle Cells |
| 0.289312 | 0.267 | 0.037 | 6.43E-70 | 20 | Crtc3 | Smooth Muscle Cells |
| 0.905101 | 0.996 | 0.943 | 8.17E-70 | 20 | mt-Co1 | Smooth Muscle Cells |
| 0.516103 | 0.356 | 0.067 | 1.05E-67 | 20 | Impdh2 | Smooth Muscle Cells |
| 0.710121 | 0.453 | 0.105 | 5.88E-67 | 20 | Gadd45g | Smooth Muscle Cells |
| 0.254603 | 0.198 | 0.022 | 8.60E-66 | 20 | Elovl5 | Smooth Muscle Cells |
| 0.372692 | 0.36 | 0.067 | 2.32E-65 | 20 | Rhoj | Smooth Muscle Cells |
| 1.015169 | 0.984 | 0.785 | 2.70E-65 | 20 | mt-Nd1 | Smooth Muscle Cells |
| 0.329817 | 0.215 | 0.026 | 3.36E-65 | 20 | Bcr | Smooth Muscle Cells |
| 0.566916 | 0.668 | 0.197 | 4.03E-65 | 20 | Ebf1 | Smooth Muscle Cells |
| 0.351067 | 0.397 | 0.08 | 3.99E-63 | 20 | Cryab | Smooth Muscle Cells |
| 0.329635 | 0.202 | 0.024 | 7.77E-63 | 20 | Adamts5 | Smooth Muscle Cells |
| 0.450642 | 0.445 | 0.105 | 9.00E-62 | 20 | Ndufa5 | Smooth Muscle Cells |
| 0.824888 | 0.891 | 0.586 | 5.63E-61 | 20 | Cox6c | Smooth Muscle Cells |
| 0.667909 | 0.854 | 0.402 | 1.89E-60 | 20 | Ptms | Smooth Muscle Cells |
| 0.35577 | 0.478 | 0.112 | 2.13E-60 | 20 | Plpp3 | Smooth Muscle Cells |
| 0.642616 | 0.745 | 0.286 | 4.85E-59 | 20 | mt-Nd4l | Smooth Muscle Cells |
| 0.588039 | 0.66 | 0.221 | 6.26E-59 | 20 | Nudt4 | Smooth Muscle Cells |
| 0.330588 | 0.328 | 0.061 | 1.10E-58 | 20 | Ehd2 | Smooth Muscle Cells |
| 0.777421 | 0.866 | 0.501 | 1.23E-58 | 20 | mt-Nd3 | Smooth Muscle Cells |
| 0.274642 | 0.251 | 0.038 | 2.99E-58 | 20 | Dbndd2 | Smooth Muscle Cells |
| 0.46958 | 0.385 | 0.085 | 3.15E-56 | 20 | Hes1 | Smooth Muscle Cells |
| 0.357414 | 0.356 | 0.074 | 2.46E-55 | 20 | Spop | Smooth Muscle Cells |
| 0.468276 | 0.547 | 0.164 | 4.90E-54 | 20 | Dynlrb1 | Smooth Muscle Cells |
| 1.091755 | 0.53 | 0.171 | 9.32E-54 | 20 | Atf3 | Smooth Muscle Cells |
| 0.390959 | 0.324 | 0.065 | 1.24E-53 | 20 | Irs2 | Smooth Muscle Cells |
| 0.267991 | 0.247 | 0.039 | 2.60E-53 | 20 | Thra | Smooth Muscle Cells |
| 0.779372 | 0.883 | 0.579 | 3.00E-53 | 20 | Calm2 | Smooth Muscle Cells |
| 0.456905 | 0.518 | 0.149 | 1.12E-52 | 20 | Cav1 | Smooth Muscle Cells |
| 0.338423 | 0.348 | 0.074 | 2.03E-52 | 20 | Vcl | Smooth Muscle Cells |
| 0.265151 | 0.231 | 0.035 | 4.89E-52 | 20 | Rassf3 | Smooth Muscle Cells |
| 1.075038 | 0.352 | 0.081 | 1.52E-51 | 20 | Il6 | Smooth Muscle Cells |
| 0.69115 | 0.794 | 0.41 | 5.33E-51 | 20 | Itgb1 | Smooth Muscle Cells |
| 0.283046 | 0.267 | 0.048 | 1.41E-49 | 20 | Ccdc85b | Smooth Muscle Cells |
| 0.264969 | 0.231 | 0.037 | 2.64E-49 | 20 | Prkacb | Smooth Muscle Cells |
| 0.568953 | 0.381 | 0.097 | 5.08E-49 | 20 | Smarca5 | Smooth Muscle Cells |
| 0.514197 | 0.599 | 0.21 | 8.40E-49 | 20 | Cyb5r3 | Smooth Muscle Cells |
| 0.444317 | 0.474 | 0.136 | 1.43E-48 | 20 | Sh3bgrl | Smooth Muscle Cells |
| 0.336475 | 0.324 | 0.07 | 6.07E-48 | 20 | Oxct1 | Smooth Muscle Cells |
| 1.150065 | 0.688 | 0.347 | 7.04E-47 | 20 | Nr4a1 | Smooth Muscle Cells |
| 0.254416 | 0.231 | 0.038 | 1.11E-46 | 20 | B3gnt2 | Smooth Muscle Cells |
| 0.28139 | 0.267 | 0.05 | 4.43E-46 | 20 | B230219D22Rik | Smooth Muscle Cells |
| 0.717552 | 0.895 | 0.67 | 8.57E-45 | 20 | Cox8a | Smooth Muscle Cells |
| 0.462369 | 0.449 | 0.132 | 1.07E-44 | 20 | Actn4 | Smooth Muscle Cells |
| 0.261541 | 0.235 | 0.041 | 1.60E-44 | 20 | Gstm1 | Smooth Muscle Cells |
| 0.433907 | 0.49 | 0.152 | 5.53E-44 | 20 | Actn1 | Smooth Muscle Cells |
| 0.42396 | 0.466 | 0.139 | 1.76E-43 | 20 | Clic4 | Smooth Muscle Cells |
| 0.287777 | 0.296 | 0.063 | 4.59E-43 | 20 | Dag1 | Smooth Muscle Cells |
| 0.490414 | 0.397 | 0.108 | 5.66E-43 | 20 | Ddit4 | Smooth Muscle Cells |
| 0.379158 | 0.421 | 0.121 | 9.43E-42 | 20 | Ndufb2 | Smooth Muscle Cells |
| 0.426618 | 0.385 | 0.109 | 1.01E-39 | 20 | Tob2 | Smooth Muscle Cells |
| 0.515884 | 0.628 | 0.261 | 2.65E-39 | 20 | Uqcr11 | Smooth Muscle Cells |
| 0.578699 | 0.846 | 0.558 | 1.55E-37 | 20 | Dynll1 | Smooth Muscle Cells |
| 0.706643 | 0.717 | 0.355 | 3.04E-37 | 20 | Zfp36l1 | Smooth Muscle Cells |
| 0.298172 | 0.312 | 0.076 | 3.19E-37 | 20 | Lims1 | Smooth Muscle Cells |
| 0.34314 | 0.64 | 0.237 | 3.63E-36 | 20 | Serpine2 | Smooth Muscle Cells |
| 0.523844 | 0.781 | 0.435 | 4.59E-36 | 20 | Slc25a4 | Smooth Muscle Cells |
| 1.758846 | 0.741 | 0.47 | 1.06E-35 | 20 | Mt1 | Smooth Muscle Cells |
| 0.653654 | 0.591 | 0.245 | 8.11E-35 | 20 | Errfi1 | Smooth Muscle Cells |
| 0.25893 | 0.263 | 0.06 | 1.28E-34 | 20 | Ltbp1 | Smooth Muscle Cells |
| 0.805633 | 0.506 | 0.204 | 2.74E-34 | 20 | Nr4a2 | Smooth Muscle Cells |
| 0.325863 | 0.381 | 0.113 | 6.89E-34 | 20 | Rock2 | Smooth Muscle Cells |
| 0.250434 | 0.239 | 0.052 | 1.43E-33 | 20 | Ech1 | Smooth Muscle Cells |
| 0.501963 | 0.911 | 0.523 | 5.85E-33 | 20 | Cd9 | Smooth Muscle Cells |
| 0.262079 | 0.259 | 0.06 | 6.06E-33 | 20 | Atp2b4 | Smooth Muscle Cells |
| 0.313597 | 0.316 | 0.085 | 1.44E-32 | 20 | Ndufa12 | Smooth Muscle Cells |
| 0.525026 | 0.781 | 0.461 | 1.48E-31 | 20 | Selenow | Smooth Muscle Cells |
| 0.535646 | 0.518 | 0.211 | 1.67E-31 | 20 | Ppp1r15a | Smooth Muscle Cells |
| 0.407284 | 0.587 | 0.249 | 2.45E-31 | 20 | Ndufb9 | Smooth Muscle Cells |
| 0.426382 | 0.316 | 0.09 | 3.48E-31 | 20 | Emd | Smooth Muscle Cells |
| 0.374313 | 0.457 | 0.165 | 1.31E-30 | 20 | Ywhaq | Smooth Muscle Cells |
| 0.266415 | 0.267 | 0.067 | 1.47E-29 | 20 | Nfia | Smooth Muscle Cells |
| 0.559813 | 0.551 | 0.246 | 1.72E-29 | 20 | Ier2 | Smooth Muscle Cells |
| 0.330866 | 0.413 | 0.14 | 2.03E-29 | 20 | Lhfp | Smooth Muscle Cells |
| 0.297906 | 0.381 | 0.124 | 1.72E-28 | 20 | Ndufs5 | Smooth Muscle Cells |
| 0.357109 | 0.417 | 0.145 | 1.89E-28 | 20 | Pdgfrb | Smooth Muscle Cells |
| 0.27362 | 0.291 | 0.081 | 5.17E-28 | 20 | Mdh1 | Smooth Muscle Cells |
| 0.260732 | 0.283 | 0.077 | 1.17E-27 | 20 | Tprgl | Smooth Muscle Cells |
| 0.97047 | 0.939 | 0.786 | 2.28E-27 | 20 | Gm42418 | Smooth Muscle Cells |
| 0.381443 | 0.457 | 0.176 | 5.50E-27 | 20 | Rheb | Smooth Muscle Cells |
| 0.382877 | 0.595 | 0.274 | 2.42E-26 | 20 | Uqcr10 | Smooth Muscle Cells |
| 0.431959 | 0.721 | 0.396 | 7.00E-26 | 20 | Atp5b | Smooth Muscle Cells |
| 0.460057 | 0.899 | 0.647 | 1.52E-25 | 20 | Cst3 | Smooth Muscle Cells |
| 0.250075 | 0.239 | 0.062 | 2.50E-25 | 20 | Sik1 | Smooth Muscle Cells |
| 0.515934 | 0.664 | 0.366 | 1.18E-24 | 20 | Pnrc1 | Smooth Muscle Cells |
| 0.3097 | 0.405 | 0.15 | 3.37E-24 | 20 | Ndufa11 | Smooth Muscle Cells |
| 0.461803 | 0.676 | 0.377 | 3.54E-24 | 20 | Atpif1 | Smooth Muscle Cells |
| 0.413731 | 0.632 | 0.321 | 5.78E-24 | 20 | Son | Smooth Muscle Cells |
| 0.260536 | 0.287 | 0.086 | 8.83E-24 | 20 | Ndufs2 | Smooth Muscle Cells |
| 0.791112 | 0.668 | 0.417 | 1.06E-23 | 20 | Gadd45b | Smooth Muscle Cells |
| 0.321645 | 0.632 | 0.277 | 1.21E-23 | 20 | Serping1 | Smooth Muscle Cells |
| 0.383503 | 0.47 | 0.195 | 1.64E-23 | 20 | Arid5b | Smooth Muscle Cells |
| 0.453667 | 0.911 | 0.729 | 2.37E-23 | 20 | Calm1 | Smooth Muscle Cells |
| 0.456435 | 0.478 | 0.205 | 2.39E-23 | 20 | Gem | Smooth Muscle Cells |
| 0.296454 | 0.328 | 0.108 | 2.52E-23 | 20 | Nfib | Smooth Muscle Cells |
| 0.318604 | 0.417 | 0.161 | 1.03E-22 | 20 | Ndufb7 | Smooth Muscle Cells |
| 0.307165 | 0.401 | 0.152 | 1.12E-22 | 20 | Ndufb10 | Smooth Muscle Cells |
| 0.271008 | 0.3 | 0.096 | 2.18E-22 | 20 | Aldh2 | Smooth Muscle Cells |
| 0.341367 | 0.522 | 0.233 | 3.37E-22 | 20 | Prkar1a | Smooth Muscle Cells |
| 0.29752 | 0.433 | 0.174 | 1.53E-21 | 20 | Ndufa1 | Smooth Muscle Cells |
| 0.44084 | 0.482 | 0.22 | 4.94E-21 | 20 | Fosl2 | Smooth Muscle Cells |
| 0.331911 | 0.32 | 0.112 | 5.31E-21 | 20 | Rhob | Smooth Muscle Cells |
| 0.689253 | 0.538 | 0.288 | 2.69E-20 | 20 | Cebpd | Smooth Muscle Cells |
| 0.361044 | 0.348 | 0.132 | 1.50E-19 | 20 | Nr4a3 | Smooth Muscle Cells |
| 0.39652 | 0.603 | 0.329 | 1.56E-19 | 20 | Ndufa13 | Smooth Muscle Cells |
| 0.411636 | 0.49 | 0.236 | 1.68E-19 | 20 | Ccnl1 | Smooth Muscle Cells |
| 0.384467 | 0.64 | 0.362 | 2.12E-19 | 20 | Cox7a2 | Smooth Muscle Cells |
| 0.322969 | 0.279 | 0.092 | 2.34E-19 | 20 | Uap1 | Smooth Muscle Cells |
| 0.337396 | 0.538 | 0.265 | 4.83E-19 | 20 | Uqcrb | Smooth Muscle Cells |
| 0.774709 | 0.559 | 0.316 | 6.98E-19 | 20 | Egr1 | Smooth Muscle Cells |
| 0.358923 | 0.571 | 0.295 | 9.57E-19 | 20 | mt-Nd5 | Smooth Muscle Cells |
| 0.298316 | 0.417 | 0.175 | 9.90E-19 | 20 | Ndufa6 | Smooth Muscle Cells |
| 0.278165 | 0.312 | 0.111 | 1.46E-18 | 20 | Aplp2 | Smooth Muscle Cells |
| 0.323766 | 0.255 | 0.082 | 2.21E-18 | 20 | Ythdc1 | Smooth Muscle Cells |
| 0.478844 | 0.644 | 0.362 | 4.07E-18 | 20 | Btg2 | Smooth Muscle Cells |
| 0.609958 | 0.543 | 0.298 | 4.57E-18 | 20 | Klf4 | Smooth Muscle Cells |
| 0.325324 | 0.279 | 0.095 | 8.39E-18 | 20 | Fgl2 | Smooth Muscle Cells |
| 0.379074 | 0.595 | 0.334 | 9.65E-18 | 20 | Atp5k | Smooth Muscle Cells |
| 0.430269 | 0.575 | 0.298 | 1.33E-17 | 20 | Cdkn1a | Smooth Muscle Cells |
| 0.471934 | 0.976 | 0.865 | 1.55E-17 | 20 | Ubb | Smooth Muscle Cells |
| 0.263652 | 0.348 | 0.139 | 1.00E-16 | 20 | Kif5b | Smooth Muscle Cells |
| 0.251341 | 0.47 | 0.211 | 2.61E-16 | 20 | Zbtb20 | Smooth Muscle Cells |
| 0.411002 | 0.377 | 0.161 | 3.92E-16 | 20 | Lars2 | Smooth Muscle Cells |
| 0.286797 | 0.466 | 0.217 | 4.61E-16 | 20 | Cnbp | Smooth Muscle Cells |
| 0.819389 | 0.741 | 0.579 | 6.44E-16 | 20 | Cebpb | Smooth Muscle Cells |
| 0.582403 | 0.789 | 0.675 | 2.24E-15 | 20 | Jund | Smooth Muscle Cells |
| 0.258616 | 0.275 | 0.103 | 1.84E-14 | 20 | Irf1 | Smooth Muscle Cells |
| 0.360887 | 0.741 | 0.53 | 6.97E-14 | 20 | Uqcrh | Smooth Muscle Cells |
| 0.300987 | 0.579 | 0.328 | 1.79E-13 | 20 | Eif4g2 | Smooth Muscle Cells |
| 0.311752 | 0.866 | 0.646 | 2.73E-13 | 20 | Ddx5 | Smooth Muscle Cells |
| 0.309348 | 0.344 | 0.15 | 5.50E-13 | 20 | AY036118 | Smooth Muscle Cells |
| 0.328347 | 0.903 | 0.762 | 5.66E-13 | 20 | Itm2b | Smooth Muscle Cells |
| 0.309988 | 0.883 | 0.754 | 1.85E-12 | 20 | Cox4i1 | Smooth Muscle Cells |
| 0.36333 | 0.802 | 0.633 | 3.64E-12 | 20 | Chchd2 | Smooth Muscle Cells |
| 0.263768 | 0.372 | 0.177 | 1.77E-11 | 20 | Ndufc1 | Smooth Muscle Cells |
| 0.253284 | 0.389 | 0.188 | 3.17E-11 | 20 | Oat | Smooth Muscle Cells |
| 0.279038 | 0.603 | 0.363 | 3.88E-11 | 20 | Cox5b | Smooth Muscle Cells |
| 0.298518 | 0.757 | 0.555 | 4.23E-11 | 20 | Atp5l | Smooth Muscle Cells |
| 0.277004 | 0.543 | 0.316 | 5.52E-11 | 20 | Atp5a1 | Smooth Muscle Cells |
| 0.260403 | 0.364 | 0.177 | 7.45E-11 | 20 | Tle5 | Smooth Muscle Cells |
| 0.31764 | 0.988 | 0.96 | 1.67E-10 | 20 | Actb | Smooth Muscle Cells |
| 0.259519 | 0.668 | 0.408 | 1.92E-10 | 20 | Cd81 | Smooth Muscle Cells |
| 0.255056 | 0.972 | 0.928 | 5.19E-10 | 20 | Eif1 | Smooth Muscle Cells |
| 0.290884 | 0.599 | 0.389 | 1.10E-09 | 20 | Atp5d | Smooth Muscle Cells |
| 0.258717 | 0.656 | 0.421 | 1.17E-09 | 20 | Map1lc3b | Smooth Muscle Cells |
| 0.612903 | 0.753 | 0.714 | 1.19E-09 | 20 | Junb | Smooth Muscle Cells |
| 0.282198 | 0.607 | 0.398 | 1.32E-09 | 20 | Atp5j | Smooth Muscle Cells |
| 0.394568 | 0.372 | 0.196 | 1.69E-09 | 20 | Tiparp | Smooth Muscle Cells |
| 0.295701 | 0.526 | 0.317 | 2.58E-09 | 20 | Slc38a2 | Smooth Muscle Cells |
| 0.28774 | 0.955 | 0.839 | 3.79E-09 | 20 | Ppia | Smooth Muscle Cells |
| 0.326374 | 0.984 | 0.842 | 4.50E-09 | 20 | Ptma | Smooth Muscle Cells |
| 0.297672 | 0.215 | 0.088 | 1.49E-08 | 20 | Sap18 | Smooth Muscle Cells |
| 0.268579 | 0.676 | 0.484 | 1.86E-08 | 20 | Atp5h | Smooth Muscle Cells |
| 0.274366 | 0.215 | 0.087 | 2.22E-08 | 20 | Maff | Smooth Muscle Cells |
| 0.256181 | 0.583 | 0.365 | 2.74E-08 | 20 | Iqgap1 | Smooth Muscle Cells |
| 0.755885 | 0.947 | 0.973 | 1.14E-07 | 20 | Malat1 | Smooth Muscle Cells |
| 0.2516 | 0.652 | 0.45 | 1.69E-07 | 20 | Gpx4 | Smooth Muscle Cells |
| 0.338437 | 0.648 | 0.469 | 6.43E-07 | 20 | Zfp36 | Smooth Muscle Cells |
| 0.487019 | 0.636 | 0.454 | 1.09E-06 | 20 | Fosb | Smooth Muscle Cells |
| 0.29201 | 0.409 | 0.238 | 3.60E-06 | 20 | Mt2 | Smooth Muscle Cells |
| 0.457858 | 0.445 | 0.279 | 3.76E-06 | 20 | Klf2 | Smooth Muscle Cells |
| 0.272865 | 0.555 | 0.396 | 6.01E-05 | 20 | Hnrnpa2b1 | Smooth Muscle Cells |
| 0.299338 | 0.85 | 0.776 | 0.001278 | 20 | Hspa8 | Smooth Muscle Cells |
| 2.728921 | 0.917 | 0.081 | 0 | 21 | Rgs5 | Pericytes (P 1.2) |
| 1.988549 | 0.829 | 0.083 | 0 | 21 | Gm13889 | Pericytes (P 1.2) |
| 1.986341 | 0.834 | 0.075 | 0 | 21 | Il6 | Pericytes (P 1.2) |
| 0.771771 | 0.488 | 0.018 | 0 | 21 | Ngf | Pericytes (P 1.2) |
| 1.132399 | 0.654 | 0.059 | 2.34E-255 | 21 | Adamts4 | Pericytes (P 1.2) |
| 3.340957 | 0.976 | 0.205 | 8.01E-224 | 21 | Ccl2 | Pericytes (P 1.2) |
| 0.798006 | 0.605 | 0.056 | 2.04E-219 | 21 | Notch3 | Pericytes (P 1.2) |
| 0.512709 | 0.361 | 0.02 | 1.03E-209 | 21 | Adra2a | Pericytes (P 1.2) |
| 0.665671 | 0.507 | 0.042 | 7.65E-208 | 21 | Pakap.1 | Pericytes (P 1.2) |
| 1.298098 | 0.834 | 0.136 | 5.22E-177 | 21 | Sparcl1 | Pericytes (P 1.2) |
| 0.278094 | 0.171 | 0.005 | 9.63E-166 | 21 | Gm42679 | Pericytes (P 1.2) |
| 0.815661 | 0.449 | 0.043 | 5.00E-157 | 21 | Rgs16 | Pericytes (P 1.2) |
| 0.523415 | 0.429 | 0.044 | 1.96E-137 | 21 | Il1r1 | Pericytes (P 1.2) |
| 1.211765 | 0.898 | 0.218 | 5.08E-136 | 21 | Col4a2 | Pericytes (P 1.2) |
| 0.653624 | 0.541 | 0.07 | 1.23E-135 | 21 | Itga1 | Pericytes (P 1.2) |
| 1.569949 | 0.956 | 0.275 | 2.09E-133 | 21 | Col4a1 | Pericytes (P 1.2) |
| 2.482457 | 1 | 0.399 | 1.75E-132 | 21 | Cxcl1 | Pericytes (P 1.2) |
| 1.23348 | 0.395 | 0.041 | 2.22E-128 | 21 | Ccl11 | Pericytes (P 1.2) |
| 0.623422 | 0.517 | 0.067 | 4.36E-126 | 21 | Ndufa4l2 | Pericytes (P 1.2) |
| 1.633744 | 0.761 | 0.167 | 7.74E-122 | 21 | Ccl7 | Pericytes (P 1.2) |
| 0.62113 | 0.288 | 0.023 | 2.33E-121 | 21 | Procr | Pericytes (P 1.2) |
| 1.228162 | 0.873 | 0.235 | 2.94E-113 | 21 | Serpine2 | Pericytes (P 1.2) |
| 1.430587 | 0.888 | 0.273 | 1.00E-112 | 21 | Phlda1 | Pericytes (P 1.2) |
| 1.902939 | 0.941 | 0.285 | 1.12E-109 | 21 | Mgp | Pericytes (P 1.2) |
| 0.908413 | 0.815 | 0.196 | 4.62E-107 | 21 | Ebf1 | Pericytes (P 1.2) |
| 0.373292 | 0.249 | 0.019 | 1.70E-105 | 21 | Apold1 | Pericytes (P 1.2) |
| 0.455105 | 0.317 | 0.031 | 1.85E-103 | 21 | Tinagl1 | Pericytes (P 1.2) |
| 0.557381 | 0.463 | 0.067 | 5.60E-103 | 21 | Rhoj | Pericytes (P 1.2) |
| 0.88186 | 0.849 | 0.222 | 4.86E-100 | 21 | Col18a1 | Pericytes (P 1.2) |
| 0.395135 | 0.205 | 0.014 | 4.34E-96 | 21 | Stc1 | Pericytes (P 1.2) |
| 0.642574 | 0.498 | 0.079 | 5.15E-95 | 21 | Mustn1 | Pericytes (P 1.2) |
| 0.669054 | 0.659 | 0.143 | 6.41E-92 | 21 | Pdgfrb | Pericytes (P 1.2) |
| 0.347531 | 0.268 | 0.026 | 2.98E-90 | 21 | Bcr | Pericytes (P 1.2) |
| 0.744963 | 0.688 | 0.159 | 1.38E-88 | 21 | Prss23 | Pericytes (P 1.2) |
| 0.802686 | 0.541 | 0.11 | 6.61E-81 | 21 | Adamts1 | Pericytes (P 1.2) |
| 0.554821 | 0.493 | 0.089 | 1.15E-79 | 21 | Col15a1 | Pericytes (P 1.2) |
| 1.587687 | 0.961 | 0.501 | 1.90E-79 | 21 | Igfbp7 | Pericytes (P 1.2) |
| 1.012471 | 0.854 | 0.275 | 3.54E-79 | 21 | Serping1 | Pericytes (P 1.2) |
| 1.042273 | 0.961 | 0.466 | 1.57E-74 | 21 | Cald1 | Pericytes (P 1.2) |
| 0.588374 | 0.527 | 0.111 | 2.37E-74 | 21 | Bach1 | Pericytes (P 1.2) |
| 0.69435 | 0.463 | 0.09 | 5.78E-73 | 21 | Uap1 | Pericytes (P 1.2) |
| 0.466061 | 0.405 | 0.068 | 1.82E-72 | 21 | Sash1 | Pericytes (P 1.2) |
| 0.343898 | 0.273 | 0.032 | 7.52E-72 | 21 | Cox4i2 | Pericytes (P 1.2) |
| 0.670806 | 0.551 | 0.123 | 1.91E-71 | 21 | Gng11 | Pericytes (P 1.2) |
| 0.521577 | 0.429 | 0.077 | 6.43E-71 | 21 | Filip1l | Pericytes (P 1.2) |
| 0.600237 | 0.478 | 0.096 | 1.07E-70 | 21 | Tcim | Pericytes (P 1.2) |
| 0.603159 | 0.546 | 0.122 | 7.96E-70 | 21 | Pdgfa | Pericytes (P 1.2) |
| 1.062729 | 0.985 | 0.634 | 3.93E-69 | 21 | Ifitm3 | Pericytes (P 1.2) |
| 0.449078 | 0.463 | 0.09 | 6.63E-67 | 21 | Mylk | Pericytes (P 1.2) |
| 0.907642 | 0.741 | 0.244 | 1.38E-64 | 21 | Errfi1 | Pericytes (P 1.2) |
| 0.306258 | 0.215 | 0.023 | 2.04E-62 | 21 | Parm1 | Pericytes (P 1.2) |
| 0.589798 | 0.659 | 0.174 | 3.83E-62 | 21 | Ifitm1 | Pericytes (P 1.2) |
| 1.425595 | 0.922 | 0.468 | 4.82E-60 | 21 | Mt1 | Pericytes (P 1.2) |
| 0.385595 | 0.229 | 0.028 | 1.22E-57 | 21 | Thbd | Pericytes (P 1.2) |
| 0.343287 | 0.317 | 0.052 | 2.25E-56 | 21 | Pdlim1 | Pericytes (P 1.2) |
| 0.663719 | 0.629 | 0.193 | 3.14E-56 | 21 | Arid5b | Pericytes (P 1.2) |
| 0.523725 | 0.537 | 0.139 | 8.90E-56 | 21 | Lhfp | Pericytes (P 1.2) |
| 0.648436 | 0.702 | 0.24 | 5.58E-54 | 21 | Tmem176b | Pericytes (P 1.2) |
| 0.296877 | 0.224 | 0.029 | 2.08E-53 | 21 | Adamts9 | Pericytes (P 1.2) |
| 0.335 | 0.278 | 0.043 | 5.89E-53 | 21 | Mcam | Pericytes (P 1.2) |
| 0.35988 | 0.127 | 0.01 | 3.39E-52 | 21 | Ccl19 | Pericytes (P 1.2) |
| 0.616561 | 0.556 | 0.159 | 1.53E-51 | 21 | Sdc1 | Pericytes (P 1.2) |
| 0.293152 | 0.215 | 0.028 | 1.07E-50 | 21 | Spry2 | Pericytes (P 1.2) |
| 0.310304 | 0.254 | 0.038 | 2.70E-50 | 21 | Des | Pericytes (P 1.2) |
| 0.568121 | 0.444 | 0.108 | 7.77E-50 | 21 | Ddit4 | Pericytes (P 1.2) |
| 0.285097 | 0.22 | 0.029 | 1.03E-49 | 21 | Abcc9 | Pericytes (P 1.2) |
| 0.345609 | 0.249 | 0.037 | 2.99E-49 | 21 | Arhgap31 | Pericytes (P 1.2) |
| 0.597434 | 0.61 | 0.193 | 4.60E-49 | 21 | Tmem176a | Pericytes (P 1.2) |
| 0.366227 | 0.376 | 0.078 | 5.29E-49 | 21 | Mprip | Pericytes (P 1.2) |
| 0.304421 | 0.249 | 0.038 | 1.00E-47 | 21 | Epas1 | Pericytes (P 1.2) |
| 1.212469 | 0.746 | 0.34 | 6.08E-46 | 21 | Id3 | Pericytes (P 1.2) |
| 0.51381 | 0.561 | 0.169 | 8.02E-46 | 21 | Pten | Pericytes (P 1.2) |
| 0.322059 | 0.278 | 0.049 | 1.84E-44 | 21 | Rbpms | Pericytes (P 1.2) |
| 0.629053 | 0.263 | 0.046 | 8.36E-44 | 21 | Ereg | Pericytes (P 1.2) |
| 0.421846 | 0.405 | 0.1 | 2.63E-42 | 21 | Zfp703 | Pericytes (P 1.2) |
| 0.32695 | 0.273 | 0.049 | 4.20E-42 | 21 | Gjc1 | Pericytes (P 1.2) |
| 0.281649 | 0.234 | 0.038 | 4.38E-41 | 21 | Ednra | Pericytes (P 1.2) |
| 0.569984 | 0.595 | 0.204 | 1.28E-39 | 21 | Gem | Pericytes (P 1.2) |
| 0.622233 | 0.898 | 0.503 | 2.49E-39 | 21 | Laptm4a | Pericytes (P 1.2) |
| 0.496216 | 0.605 | 0.223 | 1.49E-35 | 21 | Nudt4 | Pericytes (P 1.2) |
| 0.26092 | 0.215 | 0.037 | 8.69E-34 | 21 | Gfpt2 | Pericytes (P 1.2) |
| 0.686337 | 0.751 | 0.356 | 1.05E-33 | 21 | Zfp36l1 | Pericytes (P 1.2) |
| 0.731103 | 0.985 | 0.832 | 1.82E-31 | 21 | Vim | Pericytes (P 1.2) |
| 0.807935 | 0.839 | 0.545 | 2.14E-31 | 21 | Anxa1 | Pericytes (P 1.2) |
| 0.878543 | 0.644 | 0.287 | 3.26E-31 | 21 | Cebpd | Pericytes (P 1.2) |
| 0.297172 | 0.302 | 0.073 | 1.08E-30 | 21 | Rras | Pericytes (P 1.2) |
| 0.599498 | 0.941 | 0.762 | 1.31E-29 | 21 | Itm2b | Pericytes (P 1.2) |
| 1.274892 | 0.415 | 0.14 | 4.09E-29 | 21 | Cxcl5 | Pericytes (P 1.2) |
| 0.340687 | 0.463 | 0.151 | 3.18E-28 | 21 | Crip2 | Pericytes (P 1.2) |
| 0.459431 | 0.332 | 0.094 | 1.45E-27 | 21 | Odc1 | Pericytes (P 1.2) |
| 0.377289 | 0.502 | 0.188 | 8.09E-26 | 21 | Map1lc3a | Pericytes (P 1.2) |
| 0.281786 | 0.307 | 0.081 | 9.15E-26 | 21 | Cygb | Pericytes (P 1.2) |
| 0.688652 | 0.683 | 0.308 | 2.12E-25 | 21 | Acta2 | Pericytes (P 1.2) |
| 0.360023 | 0.434 | 0.15 | 6.27E-25 | 21 | Adamts2 | Pericytes (P 1.2) |
| 0.419588 | 0.561 | 0.224 | 3.50E-24 | 21 | Col5a3 | Pericytes (P 1.2) |
| 0.338997 | 0.371 | 0.119 | 8.34E-24 | 21 | Il6st | Pericytes (P 1.2) |
| 0.291683 | 0.249 | 0.062 | 1.75E-23 | 21 | Ugdh | Pericytes (P 1.2) |
| 0.4199 | 0.507 | 0.204 | 4.37E-23 | 21 | Mfge8 | Pericytes (P 1.2) |
| 0.538108 | 0.527 | 0.217 | 1.52E-22 | 21 | Tagln | Pericytes (P 1.2) |
| 0.307446 | 0.322 | 0.097 | 1.94E-22 | 21 | Lamc1 | Pericytes (P 1.2) |
| 0.262734 | 0.171 | 0.034 | 3.38E-22 | 21 | Lrrc32 | Pericytes (P 1.2) |
| 0.30109 | 0.615 | 0.267 | 6.63E-22 | 21 | Thbs1 | Pericytes (P 1.2) |
| 0.466099 | 0.741 | 0.38 | 1.36E-21 | 21 | Tpm1 | Pericytes (P 1.2) |
| 0.303543 | 0.356 | 0.115 | 1.55E-21 | 21 | Rarres2 | Pericytes (P 1.2) |
| 0.347178 | 0.366 | 0.126 | 2.34E-20 | 21 | Tent5a | Pericytes (P 1.2) |
| 0.747404 | 0.99 | 0.973 | 7.16E-20 | 21 | Malat1 | Pericytes (P 1.2) |
| 0.384691 | 0.556 | 0.251 | 7.82E-20 | 21 | Csnk1a1 | Pericytes (P 1.2) |
| 0.298166 | 0.293 | 0.088 | 1.63E-19 | 21 | Ppp1r12a | Pericytes (P 1.2) |
| 0.403302 | 0.537 | 0.237 | 2.14E-19 | 21 | Klf9 | Pericytes (P 1.2) |
| 0.274317 | 0.185 | 0.042 | 2.26E-19 | 21 | Lif | Pericytes (P 1.2) |
| 0.444975 | 0.893 | 0.577 | 4.76E-19 | 21 | Cebpb | Pericytes (P 1.2) |
| 0.254872 | 0.195 | 0.046 | 4.94E-19 | 21 | Cyth3 | Pericytes (P 1.2) |
| 0.357062 | 0.6 | 0.283 | 1.20E-18 | 21 | Tpm2 | Pericytes (P 1.2) |
| 0.270228 | 0.283 | 0.087 | 1.69E-18 | 21 | Ndufs2 | Pericytes (P 1.2) |
| 0.27626 | 0.341 | 0.114 | 2.20E-18 | 21 | Hilpda | Pericytes (P 1.2) |
| 0.349542 | 0.512 | 0.225 | 6.66E-18 | 21 | Tm4sf1 | Pericytes (P 1.2) |
| 0.275098 | 0.229 | 0.062 | 7.04E-18 | 21 | Tppp3 | Pericytes (P 1.2) |
| 0.416875 | 0.629 | 0.331 | 1.35E-17 | 21 | Prrx1 | Pericytes (P 1.2) |
| 0.557923 | 0.537 | 0.257 | 2.26E-17 | 21 | Meg3 | Pericytes (P 1.2) |
| 0.601574 | 0.815 | 0.524 | 2.77E-17 | 21 | Ier3 | Pericytes (P 1.2) |
| 0.301848 | 0.254 | 0.075 | 3.95E-17 | 21 | Cfh | Pericytes (P 1.2) |
| 0.262023 | 0.259 | 0.078 | 5.56E-17 | 21 | Lpl | Pericytes (P 1.2) |
| 0.32382 | 0.468 | 0.201 | 6.52E-17 | 21 | Rhoc | Pericytes (P 1.2) |
| 0.284084 | 0.327 | 0.113 | 9.08E-17 | 21 | Cpe | Pericytes (P 1.2) |
| 0.301097 | 0.351 | 0.129 | 1.18E-16 | 21 | Ccnd2 | Pericytes (P 1.2) |
| 0.362939 | 0.741 | 0.408 | 1.35E-16 | 21 | Cd81 | Pericytes (P 1.2) |
| 0.379883 | 0.722 | 0.403 | 1.75E-16 | 21 | Nedd4 | Pericytes (P 1.2) |
| 0.329964 | 0.454 | 0.193 | 3.07E-16 | 21 | Myl9 | Pericytes (P 1.2) |
| 0.311344 | 0.317 | 0.115 | 1.91E-15 | 21 | Rock2 | Pericytes (P 1.2) |
| 0.348309 | 0.498 | 0.239 | 9.23E-15 | 21 | 07-Sep | Pericytes (P 1.2) |
| 0.403194 | 0.932 | 0.826 | 3.54E-14 | 21 | Rpl15 | Pericytes (P 1.2) |
| 0.379632 | 0.883 | 0.705 | 9.82E-14 | 21 | Naca | Pericytes (P 1.2) |
| 0.415036 | 0.356 | 0.145 | 1.17E-13 | 21 | Inhba | Pericytes (P 1.2) |
| 0.481402 | 0.868 | 0.636 | 1.99E-13 | 21 | Lmna | Pericytes (P 1.2) |
| 0.282102 | 0.341 | 0.137 | 2.03E-13 | 21 | Eva1b | Pericytes (P 1.2) |
| 0.254044 | 0.327 | 0.125 | 2.14E-13 | 21 | Nid1 | Pericytes (P 1.2) |
| 0.42202 | 0.424 | 0.195 | 2.62E-13 | 21 | Tiparp | Pericytes (P 1.2) |
| 0.341976 | 0.2 | 0.059 | 9.24E-13 | 21 | Ptx3 | Pericytes (P 1.2) |
| 0.270692 | 0.576 | 0.289 | 1.77E-12 | 21 | Nfe2l2 | Pericytes (P 1.2) |
| 0.27133 | 0.478 | 0.229 | 2.79E-12 | 21 | Cox7a2l | Pericytes (P 1.2) |
| 0.286875 | 0.337 | 0.138 | 3.73E-12 | 21 | Ptpn1 | Pericytes (P 1.2) |
| 0.351665 | 0.834 | 0.633 | 5.89E-12 | 21 | Chchd2 | Pericytes (P 1.2) |
| 0.292208 | 0.341 | 0.142 | 7.53E-12 | 21 | Ppp1r2 | Pericytes (P 1.2) |
| 0.293026 | 0.385 | 0.172 | 2.91E-11 | 21 | Hk2 | Pericytes (P 1.2) |
| 0.253279 | 0.312 | 0.126 | 3.13E-11 | 21 | Metrnl | Pericytes (P 1.2) |
| 0.352645 | 0.478 | 0.237 | 5.48E-11 | 21 | Mt2 | Pericytes (P 1.2) |
| 0.397736 | 0.868 | 0.674 | 6.66E-11 | 21 | Jund | Pericytes (P 1.2) |
| 0.368318 | 0.937 | 0.772 | 2.05E-10 | 21 | Crip1 | Pericytes (P 1.2) |
| 0.257189 | 0.332 | 0.141 | 2.09E-10 | 21 | Fermt2 | Pericytes (P 1.2) |
| 0.321335 | 0.966 | 0.849 | 2.99E-10 | 21 | Rpl24 | Pericytes (P 1.2) |
| 0.265776 | 0.356 | 0.168 | 7.48E-09 | 21 | Cd302 | Pericytes (P 1.2) |
| 0.264983 | 0.688 | 0.412 | 1.03E-08 | 21 | Ppic | Pericytes (P 1.2) |
| 0.260732 | 0.395 | 0.197 | 1.46E-08 | 21 | Runx1 | Pericytes (P 1.2) |
| 0.275133 | 0.585 | 0.338 | 3.85E-08 | 21 | Socs3 | Pericytes (P 1.2) |
| 0.275833 | 0.995 | 0.925 | 6.03E-08 | 21 | mt-Atp6 | Pericytes (P 1.2) |
| 0.255861 | 0.966 | 0.833 | 6.19E-08 | 21 | Rpl21 | Pericytes (P 1.2) |
| 0.359935 | 0.946 | 0.843 | 7.48E-08 | 21 | Ptma | Pericytes (P 1.2) |
| 0.339572 | 0.863 | 0.776 | 8.06E-08 | 21 | Hspa8 | Pericytes (P 1.2) |
| 0.287888 | 0.6 | 0.39 | 8.14E-08 | 21 | Atp5d | Pericytes (P 1.2) |
| 0.321295 | 0.785 | 0.611 | 8.95E-08 | 21 | Eef2 | Pericytes (P 1.2) |
| 0.307686 | 0.493 | 0.283 | 1.60E-07 | 21 | 2410006H16Rik | Pericytes (P 1.2) |
| 0.277514 | 0.829 | 0.591 | 2.05E-06 | 21 | Npm1 | Pericytes (P 1.2) |
| 0.299317 | 0.99 | 0.929 | 5.09E-06 | 21 | Eef1a1 | Pericytes (P 1.2) |
| 0.252362 | 0.283 | 0.133 | 7.20E-06 | 21 | Nr4a3 | Pericytes (P 1.2) |
| 0.26194 | 0.541 | 0.337 | 1.37E-05 | 21 | Eif2s2 | Pericytes (P 1.2) |
| 0.34161 | 0.932 | 0.866 | 1.96E-05 | 21 | Ubb | Pericytes (P 1.2) |
| 0.25519 | 0.922 | 0.786 | 0.000471 | 21 | Rps2 | Pericytes (P 1.2) |
| 1.654649 | 0.366 | 0.04 | 1.33E-110 | 22 | Gm26917 | Fibroblast (Fib 1.5) |
| 1.425474 | 0.609 | 0.159 | 6.48E-75 | 22 | Lars2 | Fibroblast (Fib 1.5) |
| 0.87296 | 0.396 | 0.067 | 1.77E-71 | 22 | Col7a1 | Fibroblast (Fib 1.5) |
| 1.285512 | 0.995 | 0.925 | 9.88E-51 | 22 | mt-Atp6 | Fibroblast (Fib 1.5) |
| 1.313589 | 0.955 | 0.87 | 1.43E-49 | 22 | mt-Cytb | Fibroblast (Fib 1.5) |
| 1.188421 | 0.98 | 0.943 | 3.23E-49 | 22 | mt-Co1 | Fibroblast (Fib 1.5) |
| 1.41508 | 0.936 | 0.555 | 1.44E-48 | 22 | Col1a1 | Fibroblast (Fib 1.5) |
| 1.188647 | 0.97 | 0.927 | 4.70E-47 | 22 | mt-Co2 | Fibroblast (Fib 1.5) |
| 1.30225 | 0.96 | 0.928 | 7.67E-47 | 22 | mt-Co3 | Fibroblast (Fib 1.5) |
| 0.742893 | 0.535 | 0.166 | 1.66E-45 | 22 | Nrep | Fibroblast (Fib 1.5) |
| 1.004964 | 0.822 | 0.419 | 8.16E-44 | 22 | Col5a1 | Fibroblast (Fib 1.5) |
| 0.923848 | 0.851 | 0.435 | 4.10E-43 | 22 | Col6a2 | Fibroblast (Fib 1.5) |
| 0.9571 | 0.827 | 0.399 | 4.84E-42 | 22 | Aebp1 | Fibroblast (Fib 1.5) |
| 1.058344 | 0.916 | 0.804 | 3.61E-41 | 22 | mt-Nd4 | Fibroblast (Fib 1.5) |
| 2.620769 | 0.946 | 0.787 | 4.91E-40 | 22 | Gm42418 | Fibroblast (Fib 1.5) |
| 0.7074 | 0.505 | 0.16 | 8.01E-40 | 22 | Lrrc15 | Fibroblast (Fib 1.5) |
| 0.990699 | 0.881 | 0.497 | 1.02E-39 | 22 | Col5a2 | Fibroblast (Fib 1.5) |
| 0.46389 | 0.485 | 0.142 | 1.03E-39 | 22 | Hspg2 | Fibroblast (Fib 1.5) |
| 0.376053 | 0.297 | 0.06 | 2.00E-39 | 22 | Col27a1 | Fibroblast (Fib 1.5) |
| 0.333267 | 0.297 | 0.061 | 9.77E-39 | 22 | Mrc2 | Fibroblast (Fib 1.5) |
| 1.161142 | 0.901 | 0.458 | 1.82E-38 | 22 | Postn | Fibroblast (Fib 1.5) |
| 0.938168 | 0.748 | 0.367 | 5.72E-38 | 22 | Mmp14 | Fibroblast (Fib 1.5) |
| 0.829385 | 0.663 | 0.27 | 2.15E-37 | 22 | Thbs2 | Fibroblast (Fib 1.5) |
| 0.47442 | 0.505 | 0.16 | 6.24E-37 | 22 | Col16a1 | Fibroblast (Fib 1.5) |
| 0.968771 | 0.866 | 0.472 | 7.22E-37 | 22 | Col6a1 | Fibroblast (Fib 1.5) |
| 1.026601 | 0.896 | 0.786 | 3.18E-35 | 22 | mt-Nd1 | Fibroblast (Fib 1.5) |
| 0.540602 | 0.505 | 0.17 | 1.12E-34 | 22 | Bmp1 | Fibroblast (Fib 1.5) |
| 0.663559 | 0.658 | 0.294 | 2.30E-33 | 22 | mt-Nd5 | Fibroblast (Fib 1.5) |
| 1.592015 | 0.975 | 0.973 | 1.20E-32 | 22 | Malat1 | Fibroblast (Fib 1.5) |
| 0.957371 | 0.812 | 0.443 | 7.60E-32 | 22 | Col6a3 | Fibroblast (Fib 1.5) |
| 0.814926 | 0.728 | 0.342 | 5.57E-31 | 22 | Col12a1 | Fibroblast (Fib 1.5) |
| 1.049716 | 0.946 | 0.557 | 6.15E-31 | 22 | Col3a1 | Fibroblast (Fib 1.5) |
| 0.973734 | 0.96 | 0.565 | 6.91E-31 | 22 | Col1a2 | Fibroblast (Fib 1.5) |
| 0.641828 | 0.634 | 0.289 | 7.99E-31 | 22 | mt-Nd4l | Fibroblast (Fib 1.5) |
| 0.76656 | 0.802 | 0.437 | 3.96E-29 | 22 | Fstl1 | Fibroblast (Fib 1.5) |
| 0.849543 | 0.767 | 0.468 | 5.20E-29 | 22 | Rrbp1 | Fibroblast (Fib 1.5) |
| 0.779063 | 0.866 | 0.67 | 5.79E-28 | 22 | mt-Nd2 | Fibroblast (Fib 1.5) |
| 0.313937 | 0.287 | 0.072 | 1.13E-26 | 22 | Chd3 | Fibroblast (Fib 1.5) |
| 0.316623 | 0.297 | 0.077 | 1.16E-26 | 22 | P3h3 | Fibroblast (Fib 1.5) |
| 0.63599 | 0.629 | 0.272 | 6.40E-26 | 22 | Col11a1 | Fibroblast (Fib 1.5) |
| 0.940866 | 0.406 | 0.15 | 4.62E-24 | 22 | AY036118 | Fibroblast (Fib 1.5) |
| 0.276505 | 0.267 | 0.068 | 1.26E-23 | 22 | Lama4 | Fibroblast (Fib 1.5) |
| 0.357038 | 0.282 | 0.077 | 1.89E-23 | 22 | Lrrc17 | Fibroblast (Fib 1.5) |
| 0.557213 | 0.48 | 0.189 | 2.12E-23 | 22 | Cpxm1 | Fibroblast (Fib 1.5) |
| 0.466202 | 0.49 | 0.201 | 4.89E-23 | 22 | Fgfr1 | Fibroblast (Fib 1.5) |
| 0.28409 | 0.287 | 0.08 | 2.55E-22 | 22 | Ptprs | Fibroblast (Fib 1.5) |
| 0.2547 | 0.243 | 0.061 | 8.89E-22 | 22 | Adam12 | Fibroblast (Fib 1.5) |
| 0.824028 | 0.579 | 0.265 | 9.72E-22 | 22 | Gpx3 | Fibroblast (Fib 1.5) |
| 0.329281 | 0.347 | 0.112 | 1.49E-21 | 22 | Lamb1 | Fibroblast (Fib 1.5) |
| 0.546915 | 0.738 | 0.376 | 6.99E-21 | 22 | Mmp2 | Fibroblast (Fib 1.5) |
| 0.651565 | 0.743 | 0.503 | 8.87E-21 | 22 | mt-Nd3 | Fibroblast (Fib 1.5) |
| 0.558274 | 0.431 | 0.179 | 2.12E-20 | 22 | Loxl2 | Fibroblast (Fib 1.5) |
| 0.289467 | 0.302 | 0.095 | 7.32E-19 | 22 | Nisch | Fibroblast (Fib 1.5) |
| 0.428501 | 0.495 | 0.221 | 1.41E-18 | 22 | Ccdc80 | Fibroblast (Fib 1.5) |
| 0.673572 | 0.485 | 0.226 | 2.44E-18 | 22 | Col5a3 | Fibroblast (Fib 1.5) |
| 0.372338 | 0.411 | 0.164 | 8.71E-18 | 22 | C1qtnf6 | Fibroblast (Fib 1.5) |
| 0.869515 | 0.411 | 0.168 | 1.28E-17 | 22 | Tnc | Fibroblast (Fib 1.5) |
| 0.506174 | 0.564 | 0.278 | 2.80E-17 | 22 | Lox | Fibroblast (Fib 1.5) |
| 0.250702 | 0.252 | 0.074 | 3.75E-17 | 22 | Mlec | Fibroblast (Fib 1.5) |
| 0.440227 | 0.545 | 0.266 | 6.04E-17 | 22 | Fbln2 | Fibroblast (Fib 1.5) |
| 0.440356 | 0.535 | 0.261 | 7.46E-17 | 22 | Tcf4 | Fibroblast (Fib 1.5) |
| 0.307975 | 0.292 | 0.097 | 9.87E-17 | 22 | Creb3l1 | Fibroblast (Fib 1.5) |
| 0.296461 | 0.277 | 0.089 | 1.09E-16 | 22 | Plod2 | Fibroblast (Fib 1.5) |
| 0.39218 | 0.411 | 0.175 | 2.67E-16 | 22 | Wls | Fibroblast (Fib 1.5) |
| 0.310205 | 0.282 | 0.093 | 3.23E-16 | 22 | Atrx | Fibroblast (Fib 1.5) |
| 0.340619 | 0.337 | 0.124 | 3.37E-16 | 22 | Rpn1 | Fibroblast (Fib 1.5) |
| 0.671217 | 0.847 | 0.551 | 4.02E-16 | 22 | Fn1 | Fibroblast (Fib 1.5) |
| 0.526193 | 0.614 | 0.347 | 7.43E-16 | 22 | Fbn1 | Fibroblast (Fib 1.5) |
| 0.539583 | 0.782 | 0.469 | 7.51E-16 | 22 | Cald1 | Fibroblast (Fib 1.5) |
| 0.365778 | 0.416 | 0.174 | 8.11E-16 | 22 | Pdgfra | Fibroblast (Fib 1.5) |
| 0.664252 | 0.688 | 0.446 | 1.04E-15 | 22 | Pmepa1 | Fibroblast (Fib 1.5) |
| 0.285371 | 0.287 | 0.097 | 1.24E-15 | 22 | Lima1 | Fibroblast (Fib 1.5) |
| 0.345602 | 0.347 | 0.132 | 1.48E-15 | 22 | Antxr1 | Fibroblast (Fib 1.5) |
| 0.365118 | 0.386 | 0.162 | 5.21E-15 | 22 | Palld | Fibroblast (Fib 1.5) |
| 0.625744 | 0.851 | 0.53 | 8.60E-15 | 22 | Bgn | Fibroblast (Fib 1.5) |
| 1.051472 | 0.292 | 0.104 | 9.26E-15 | 22 | Mmp13 | Fibroblast (Fib 1.5) |
| 0.287434 | 0.317 | 0.116 | 1.21E-14 | 22 | Scarf2 | Fibroblast (Fib 1.5) |
| 0.259112 | 0.257 | 0.086 | 2.46E-13 | 22 | Dst | Fibroblast (Fib 1.5) |
| 0.413774 | 0.752 | 0.426 | 3.75E-13 | 22 | Igfbp4 | Fibroblast (Fib 1.5) |
| 0.452807 | 0.639 | 0.396 | 5.00E-13 | 22 | P4hb | Fibroblast (Fib 1.5) |
| 0.294041 | 0.312 | 0.121 | 8.10E-13 | 22 | Fkbp10 | Fibroblast (Fib 1.5) |
| 0.505313 | 0.96 | 0.589 | 1.67E-12 | 22 | Sparc | Fibroblast (Fib 1.5) |
| 0.25769 | 0.228 | 0.074 | 2.01E-12 | 22 | Epha3 | Fibroblast (Fib 1.5) |
| 0.266014 | 0.267 | 0.095 | 3.14E-12 | 22 | Tgfb2 | Fibroblast (Fib 1.5) |
| 0.289097 | 0.233 | 0.078 | 5.59E-12 | 22 | Rflnb | Fibroblast (Fib 1.5) |
| 0.403304 | 0.5 | 0.257 | 7.57E-12 | 22 | Cdh11 | Fibroblast (Fib 1.5) |
| 0.295753 | 0.347 | 0.147 | 3.07E-11 | 22 | Pdgfrb | Fibroblast (Fib 1.5) |
| 0.378809 | 0.401 | 0.187 | 3.80E-11 | 22 | Col8a1 | Fibroblast (Fib 1.5) |
| 0.301684 | 0.396 | 0.185 | 4.62E-11 | 22 | Maged1 | Fibroblast (Fib 1.5) |
| 0.30137 | 0.406 | 0.191 | 4.96E-11 | 22 | Vcan | Fibroblast (Fib 1.5) |
| 0.385082 | 0.658 | 0.382 | 5.64E-11 | 22 | Tpm1 | Fibroblast (Fib 1.5) |
| 0.841305 | 0.46 | 0.258 | 6.99E-11 | 22 | Meg3 | Fibroblast (Fib 1.5) |
| 0.442088 | 0.337 | 0.144 | 1.20E-10 | 22 | Igfbp3 | Fibroblast (Fib 1.5) |
| 0.279613 | 0.292 | 0.116 | 1.47E-10 | 22 | mt-Atp8 | Fibroblast (Fib 1.5) |
| 0.326681 | 0.431 | 0.218 | 8.14E-10 | 22 | Loxl1 | Fibroblast (Fib 1.5) |
| 0.281594 | 0.361 | 0.164 | 3.92E-09 | 22 | Tnn | Fibroblast (Fib 1.5) |
| 0.482666 | 0.728 | 0.407 | 5.69E-09 | 22 | Lum | Fibroblast (Fib 1.5) |
| 0.261413 | 0.287 | 0.12 | 7.52E-09 | 22 | Hnrnph1 | Fibroblast (Fib 1.5) |
| 0.344223 | 0.302 | 0.136 | 1.61E-08 | 22 | Gm47283 | Fibroblast (Fib 1.5) |
| 0.258146 | 0.218 | 0.081 | 2.05E-08 | 22 | Rian | Fibroblast (Fib 1.5) |
| 0.353835 | 0.792 | 0.504 | 2.13E-07 | 22 | Serpinh1 | Fibroblast (Fib 1.5) |
| 0.393753 | 0.465 | 0.279 | 5.01E-06 | 22 | Htra1 | Fibroblast (Fib 1.5) |
| 0.375175 | 0.584 | 0.372 | 1.07E-05 | 22 | Nupr1 | Fibroblast (Fib 1.5) |
| 0.288945 | 0.431 | 0.26 | 1.58E-05 | 22 | Calu | Fibroblast (Fib 1.5) |
| 0.315215 | 0.594 | 0.405 | 4.89E-05 | 22 | Nedd4 | Fibroblast (Fib 1.5) |
| 0.309091 | 0.267 | 0.131 | 5.35E-05 | 22 | Aqp1 | Fibroblast (Fib 1.5) |
| 0.270909 | 0.307 | 0.155 | 5.76E-05 | 22 | Sfrp1 | Fibroblast (Fib 1.5) |
| 0.270967 | 0.545 | 0.367 | 0.000112 | 22 | Lrp1 | Fibroblast (Fib 1.5) |
| 0.259653 | 0.426 | 0.258 | 0.00026 | 22 | Ckap4 | Fibroblast (Fib 1.5) |
| 0.275041 | 0.441 | 0.284 | 0.000485 | 22 | Hdlbp | Fibroblast (Fib 1.5) |
| 0.285693 | 0.391 | 0.234 | 0.000627 | 22 | Nbl1 | Fibroblast (Fib 1.5) |
| 0.367104 | 0.515 | 0.347 | 0.002113 | 22 | Aspn | Fibroblast (Fib 1.5) |
| 0.250025 | 0.515 | 0.346 | 0.002373 | 22 | Rcn3 | Fibroblast (Fib 1.5) |
| 0.413401 | 0.599 | 0.441 | 0.003552 | 22 | Neat1 | Fibroblast (Fib 1.5) |
| 0.290462 | 0.262 | 0.142 | 0.00921 | 22 | Fmod | Fibroblast (Fib 1.5) |
| 0.286536 | 0.584 | 0.414 | 0.010719 | 22 | Ppic | Fibroblast (Fib 1.5) |
| 0.258659 | 0.243 | 0.129 | 0.012978 | 22 | Serpine1 | Fibroblast (Fib 1.5) |
| 1.591646 | 0.966 | 0.019 | 1.28E-276 | 23 | Ube2c | Mixed |
| 0.903679 | 0.655 | 0.012 | 2.79E-200 | 23 | Cenpf | Mixed |
| 0.844713 | 0.759 | 0.016 | 7.78E-200 | 23 | Cdk1 | Mixed |
| 1.092994 | 0.966 | 0.031 | 1.60E-171 | 23 | Birc5 | Mixed |
| 0.420421 | 0.345 | 0.004 | 4.95E-153 | 23 | Ccnb1 | Mixed |
| 1.024726 | 0.828 | 0.027 | 2.51E-145 | 23 | Top2a | Mixed |
| 0.645974 | 0.552 | 0.012 | 2.22E-140 | 23 | Prc1 | Mixed |
| 0.415773 | 0.345 | 0.008 | 1.91E-85 | 23 | Cdca3 | Mixed |
| 0.379041 | 0.31 | 0.007 | 4.34E-76 | 23 | Hmmr | Mixed |
| 0.414197 | 0.345 | 0.009 | 3.26E-75 | 23 | Cdca8 | Mixed |
| 0.949062 | 0.897 | 0.061 | 3.22E-73 | 23 | Cenpa | Mixed |
| 0.450074 | 0.345 | 0.009 | 4.50E-72 | 23 | Cdc20 | Mixed |
| 0.60195 | 0.517 | 0.021 | 1.61E-71 | 23 | Smc2 | Mixed |
| 0.479226 | 0.414 | 0.013 | 7.37E-71 | 23 | Spc24 | Mixed |
| 1.749205 | 1 | 0.093 | 2.12E-63 | 23 | Stmn1 | Mixed |
| 0.304013 | 0.241 | 0.005 | 4.06E-61 | 23 | Cenpe | Mixed |
| 0.509386 | 0.414 | 0.016 | 1.26E-59 | 23 | Ccnb2 | Mixed |
| 0.726398 | 0.655 | 0.039 | 1.56E-59 | 23 | Cks1b | Mixed |
| 0.828466 | 0.897 | 0.081 | 8.78E-53 | 23 | Selenoh | Mixed |
| 0.302525 | 0.241 | 0.006 | 2.00E-52 | 23 | Nusap1 | Mixed |
| 0.623445 | 0.517 | 0.028 | 2.72E-52 | 23 | H2afx | Mixed |
| 0.920522 | 0.931 | 0.091 | 1.06E-49 | 23 | Cks2 | Mixed |
| 1.150211 | 0.931 | 0.136 | 6.48E-35 | 23 | Tuba1b | Mixed |
| 1.2368 | 0.966 | 0.156 | 2.37E-34 | 23 | Tubb4b | Mixed |
| 0.515105 | 0.483 | 0.035 | 8.51E-34 | 23 | Ccdc34 | Mixed |
| 0.534005 | 0.517 | 0.04 | 1.89E-33 | 23 | Pclaf | Mixed |
| 0.26049 | 0.207 | 0.007 | 3.06E-33 | 23 | Tpx2 | Mixed |
| 0.296774 | 0.241 | 0.009 | 4.65E-32 | 23 | Cdkn2c | Mixed |
| 0.961106 | 0.759 | 0.099 | 4.71E-30 | 23 | Arl6ip1 | Mixed |
| 1.398778 | 1 | 0.25 | 4.16E-27 | 23 | Ube2s | Mixed |
| 0.880092 | 0.862 | 0.144 | 6.69E-25 | 23 | H2afv | Mixed |
| 0.736053 | 0.793 | 0.125 | 2.66E-23 | 23 | Nucks1 | Mixed |
| 0.599978 | 0.655 | 0.085 | 3.62E-23 | 23 | Tubb6 | Mixed |
| 1.111675 | 0.966 | 0.228 | 6.04E-20 | 23 | Hmgb2 | Mixed |
| 1.744757 | 1 | 0.407 | 7.95E-20 | 23 | Tubb5 | Mixed |
| 0.447294 | 0.379 | 0.034 | 8.79E-20 | 23 | Tmpo | Mixed |
| 0.356195 | 0.276 | 0.02 | 3.17E-18 | 23 | Mki67 | Mixed |
| 1.31852 | 0.966 | 0.336 | 5.64E-17 | 23 | Tuba1a | Mixed |
| 0.286442 | 0.241 | 0.017 | 2.18E-16 | 23 | H1fx | Mixed |
| 1.754006 | 1 | 0.522 | 2.54E-16 | 23 | H2afz | Mixed |
| 0.613707 | 0.586 | 0.091 | 3.59E-16 | 23 | Hmgn2 | Mixed |
| 1.18619 | 1 | 0.498 | 1.39E-15 | 23 | Hmgb1 | Mixed |
| 0.705651 | 0.828 | 0.189 | 7.89E-15 | 23 | Prdx4 | Mixed |
| 0.700049 | 0.345 | 0.036 | 1.81E-14 | 23 | Hist1h2ap | Mixed |
| 0.518752 | 0.586 | 0.1 | 1.23E-13 | 23 | Snrpd1 | Mixed |
| 0.925861 | 0.931 | 0.304 | 2.29E-13 | 23 | Ran | Mixed |
| 0.939334 | 1 | 0.583 | 1.13E-11 | 23 | Calm2 | Mixed |
| 0.578275 | 0.862 | 0.222 | 2.33E-11 | 23 | Anp32b | Mixed |
| 0.751394 | 0.655 | 0.152 | 6.98E-11 | 23 | Jpt1 | Mixed |
| 0.541465 | 0.759 | 0.185 | 2.36E-10 | 23 | Ranbp1 | Mixed |
| 0.352228 | 0.345 | 0.047 | 1.72E-09 | 23 | Smc4 | Mixed |
| 0.583265 | 0.586 | 0.135 | 8.65E-09 | 23 | Dek | Mixed |
| 0.795772 | 1 | 0.624 | 1.15E-08 | 23 | Rbm3 | Mixed |
| 0.876327 | 1 | 0.841 | 6.71E-08 | 23 | Ppia | Mixed |
| 0.959542 | 1 | 0.844 | 2.01E-07 | 23 | Ptma | Mixed |
| 0.493907 | 0.655 | 0.166 | 3.39E-07 | 23 | Tnn | Mixed |
| 0.294061 | 0.276 | 0.038 | 8.01E-07 | 23 | Bub3 | Mixed |
| 0.694073 | 0.931 | 0.409 | 8.46E-07 | 23 | Ptms | Mixed |
| 0.813276 | 0.966 | 0.53 | 2.64E-06 | 23 | Tagln2 | Mixed |
| 0.600621 | 0.897 | 0.392 | 1.75E-05 | 23 | Sumo2 | Mixed |
| 0.406667 | 0.517 | 0.134 | 4.43E-05 | 23 | Cbx3 | Mixed |
| 0.539695 | 0.724 | 0.252 | 4.62E-05 | 23 | Cavin3 | Mixed |
| 0.539854 | 0.828 | 0.323 | 4.75E-05 | 23 | Tceal9 | Mixed |
| 0.561387 | 0.931 | 0.441 | 4.89E-05 | 23 | Reep5 | Mixed |
| 0.510167 | 0.793 | 0.29 | 6.11E-05 | 23 | Pdia6 | Mixed |
| 0.385484 | 0.483 | 0.12 | 7.67E-05 | 23 | Tubb2a | Mixed |
| 0.838996 | 1 | 0.866 | 0.000104 | 23 | Actg1 | Mixed |
| 0.853479 | 1 | 0.74 | 0.000115 | 23 | Lgals1 | Mixed |
| 0.514388 | 0.759 | 0.292 | 0.000234 | 23 | Hmgn1 | Mixed |
| 0.487597 | 0.759 | 0.269 | 0.000269 | 23 | Fbln2 | Mixed |
| 0.653963 | 0.966 | 0.527 | 0.000271 | 23 | Hsp90b1 | Mixed |
| 0.429496 | 0.69 | 0.233 | 0.00033 | 23 | Snrpb | Mixed |
| 0.734511 | 1 | 0.654 | 0.000363 | 23 | Cd63 | Mixed |
| 0.493928 | 0.966 | 0.452 | 0.000425 | 23 | Hint1 | Mixed |
| 0.27699 | 0.276 | 0.049 | 0.000437 | 23 | Dut | Mixed |
| 0.715438 | 0.552 | 0.17 | 0.000449 | 23 | Tnc | Mixed |
| 0.686867 | 0.552 | 0.172 | 0.000733 | 23 | Crabp1 | Mixed |
| 0.429542 | 0.759 | 0.282 | 0.000769 | 23 | Mrfap1 | Mixed |
| 0.456824 | 0.552 | 0.164 | 0.00082 | 23 | Lrrc15 | Mixed |
| 0.583809 | 0.931 | 0.562 | 0.00091 | 23 | Dynll1 | Mixed |
| 0.531823 | 1 | 0.635 | 0.000954 | 23 | Ybx1 | Mixed |
| 0.729212 | 0.655 | 0.228 | 0.000992 | 23 | Tm4sf1 | Mixed |
| 0.53648 | 1 | 0.615 | 0.002484 | 23 | Ppib | Mixed |
| 0.463012 | 0.69 | 0.26 | 0.002691 | 23 | Ckap4 | Mixed |
| 0.575502 | 0.931 | 0.449 | 0.003072 | 23 | Pmepa1 | Mixed |
| 0.523669 | 0.931 | 0.473 | 0.003397 | 23 | Clic1 | Mixed |
| 0.423509 | 0.621 | 0.218 | 0.003428 | 23 | Psmb6 | Mixed |
| 0.470335 | 0.724 | 0.286 | 0.004663 | 23 | Tpm2 | Mixed |
| 0.577967 | 0.724 | 0.275 | 0.004982 | 23 | Col11a1 | Mixed |
| 0.374563 | 0.448 | 0.134 | 0.013409 | 23 | Hdgf | Mixed |
| 0.430275 | 0.966 | 0.48 | 0.014107 | 23 | Selenof | Mixed |
| 0.43325 | 0.931 | 0.488 | 0.020349 | 23 | Atp5g2 | Mixed |
| 0.755108 | 0.897 | 0.507 | 0.02111 | 23 | Serpinh1 | Mixed |
| 0.430273 | 0.724 | 0.296 | 0.02365 | 23 | Manf | Mixed |
| 0.362086 | 0.621 | 0.224 | 0.023808 | 23 | Olfml3 | Mixed |
| 0.396984 | 0.828 | 0.371 | 0.02475 | 23 | Arf1 | Mixed |
| 0.604007 | 0.966 | 0.463 | 0.027119 | 23 | Postn | Mixed |
| 0.336613 | 0.448 | 0.137 | 0.030499 | 23 | Sf3b5 | Mixed |
| 0.475358 | 0.69 | 0.297 | 0.035406 | 23 | Ostc | Mixed |
| 0.454273 | 0.862 | 0.415 | 0.037171 | 23 | Ppic | Mixed |
| 0.488923 | 0.966 | 0.637 | 0.038489 | 23 | Gnas | Mixed |
| 0.296995 | 0.345 | 0.091 | 0.040479 | 23 | Ndufv2 | Mixed |
| 0.845039 | 0.75 | 0.036 | 5.24E-46 | 24 | Sncg | Mixed |
| 1.773721 | 1 | 0.083 | 1.55E-38 | 24 | Mustn1 | Mixed |
| 1.659953 | 1 | 0.092 | 1.07E-32 | 24 | Gm13889 | Mixed |
| 0.437721 | 0.375 | 0.014 | 3.23E-29 | 24 | Casp6 | Mixed |
| 0.416333 | 0.375 | 0.018 | 1.03E-21 | 24 | Pcp4l1 | Mixed |
| 0.5091 | 0.5 | 0.034 | 2.00E-19 | 24 | Myh11 | Mixed |
| 0.989364 | 0.75 | 0.082 | 1.30E-18 | 24 | Acp5 | Mixed |
| 0.670998 | 0.562 | 0.047 | 6.75E-18 | 24 | Ptp4a3 | Mixed |
| 0.798443 | 0.812 | 0.097 | 3.01E-17 | 24 | Ckb | Mixed |
| 1.492668 | 1 | 0.196 | 9.97E-15 | 24 | Myl9 | Mixed |
| 0.392934 | 0.375 | 0.025 | 2.79E-14 | 24 | Ly6c2 | Mixed |
| 1.737358 | 1 | 0.221 | 8.67E-14 | 24 | Tagln | Mixed |
| 0.709389 | 0.812 | 0.116 | 4.08E-13 | 24 | Hspb1 | Mixed |
| 0.298285 | 0.25 | 0.013 | 9.81E-12 | 24 | Bcam | Mixed |
| 1.865771 | 1 | 0.241 | 2.21E-10 | 24 | Lyz2 | Mixed |
| 0.873833 | 0.812 | 0.144 | 2.78E-09 | 24 | Sparcl1 | Mixed |
| 0.75819 | 0.812 | 0.149 | 6.28E-09 | 24 | Wfdc17 | Mixed |
| 0.483888 | 0.5 | 0.062 | 1.29E-08 | 24 | Fam162a | Mixed |
| 1.594914 | 1 | 0.312 | 2.00E-08 | 24 | Acta2 | Mixed |
| 0.400721 | 0.375 | 0.039 | 1.28E-07 | 24 | Cib1 | Mixed |
| 0.638822 | 0.938 | 0.184 | 1.90E-07 | 24 | Cd74 | Mixed |
| 0.799193 | 0.938 | 0.243 | 2.34E-07 | 24 | H2afj | Mixed |
| 1.110363 | 0.938 | 0.286 | 3.58E-07 | 24 | Tpm2 | Mixed |
| 0.39633 | 0.375 | 0.042 | 8.20E-07 | 24 | Cenpx | Mixed |
| 1.086225 | 1 | 0.293 | 2.02E-06 | 24 | Mgp | Mixed |
| 0.393469 | 0.375 | 0.044 | 2.58E-06 | 24 | Naa38 | Mixed |
| 0.470356 | 0.5 | 0.075 | 3.58E-06 | 24 | Rras | Mixed |
| 1.234375 | 1 | 0.823 | 6.27E-06 | 24 | Myl6 | Mixed |
| 1.732912 | 1 | 0.774 | 6.98E-06 | 24 | Crip1 | Mixed |
| 0.545952 | 0.875 | 0.192 | 7.41E-06 | 24 | Lst1 | Mixed |
| 0.772017 | 0.938 | 0.235 | 8.43E-06 | 24 | Alox5ap | Mixed |
| 0.365697 | 0.375 | 0.047 | 2.52E-05 | 24 | Ms4a4c | Mixed |
| 1.160655 | 1 | 0.32 | 2.81E-05 | 24 | Fcer1g | Mixed |
| 0.490489 | 0.562 | 0.101 | 2.95E-05 | 24 | Vps28 | Mixed |
| 0.646259 | 0.938 | 0.226 | 3.08E-05 | 24 | Ctss | Mixed |
| 0.318163 | 0.312 | 0.035 | 5.20E-05 | 24 | Cox4i2 | Mixed |
| 1.124523 | 1 | 0.499 | 7.33E-05 | 24 | Atp6v0c | Mixed |
| 0.320295 | 0.312 | 0.036 | 7.83E-05 | 24 | Irf8 | Mixed |
| 0.663106 | 0.5 | 0.089 | 0.000142 | 24 | Crispld2 | Mixed |
| 1.207218 | 1 | 0.96 | 0.000239 | 24 | Actb | Mixed |
| 0.33857 | 0.5 | 0.085 | 0.000246 | 24 | Il6 | Mixed |
| 0.331992 | 0.312 | 0.038 | 0.000249 | 24 | 1110008P14Rik | Mixed |
| 0.481356 | 0.562 | 0.111 | 0.000258 | 24 | Mrps21 | Mixed |
| 0.695706 | 0.75 | 0.189 | 0.00026 | 24 | Plac8 | Mixed |
| 1.140757 | 1 | 0.638 | 0.000272 | 24 | Ifitm3 | Mixed |
| 0.937193 | 1 | 0.456 | 0.000346 | 24 | Gpx1 | Mixed |
| 0.782609 | 1 | 0.309 | 0.000364 | 24 | Cd52 | Mixed |
| 1.173629 | 1 | 0.507 | 0.000383 | 24 | Igfbp7 | Mixed |
| 0.712865 | 0.938 | 0.351 | 0.000546 | 24 | Mrpl52 | Mixed |
| 0.427105 | 0.75 | 0.193 | 0.000609 | 24 | Spp1 | Mixed |
| 1.041537 | 1 | 0.331 | 0.000701 | 24 | Tyrobp | Mixed |
| 0.412673 | 0.438 | 0.075 | 0.000985 | 24 | Ndufb6 | Mixed |
| 0.548736 | 0.625 | 0.141 | 0.001015 | 24 | Cd68 | Mixed |
| 0.521826 | 0.562 | 0.122 | 0.001169 | 24 | Pdcd5 | Mixed |
| 0.504529 | 1 | 0.295 | 0.002085 | 24 | Cd14 | Mixed |
| 0.919425 | 0.938 | 0.613 | 0.003074 | 24 | Gapdh | Mixed |
| 0.717334 | 0.875 | 0.333 | 0.003214 | 24 | Ndufa13 | Mixed |
| 0.784424 | 1 | 0.894 | 0.003932 | 24 | Rpl35a | Mixed |
| 0.275295 | 0.25 | 0.029 | 0.003965 | 24 | Mrps6 | Mixed |
| 0.399624 | 0.625 | 0.138 | 0.004166 | 24 | Ms4a6c | Mixed |
| 0.873123 | 1 | 0.473 | 0.004271 | 24 | Mt1 | Mixed |
| 0.508855 | 0.75 | 0.201 | 0.005365 | 24 | Ifi30 | Mixed |
| 0.630064 | 1 | 0.392 | 0.006681 | 24 | Atp5d | Mixed |
| 0.658163 | 0.875 | 0.346 | 0.008044 | 24 | Ndufa2 | Mixed |
| 0.570998 | 1 | 0.395 | 0.010604 | 24 | Lgals3 | Mixed |
| 0.398031 | 0.438 | 0.085 | 0.010645 | 24 | Hcfc1r1 | Mixed |
| 0.802433 | 1 | 0.97 | 0.011271 | 24 | Fau | Mixed |
| 0.508076 | 0.812 | 0.253 | 0.011466 | 24 | Rbx1 | Mixed |
| 0.308251 | 0.312 | 0.046 | 0.011613 | 24 | Ramp1 | Mixed |
| 0.813146 | 1 | 0.841 | 0.011689 | 24 | Ppia | Mixed |
| 0.273396 | 0.25 | 0.032 | 0.013311 | 24 | S100a1 | Mixed |
| 0.429151 | 0.75 | 0.206 | 0.016944 | 24 | Plaur | Mixed |
| 0.273396 | 0.25 | 0.033 | 0.019771 | 24 | Commd4 | Mixed |
| 0.271679 | 0.25 | 0.033 | 0.021623 | 24 | Ndufaf8 | Mixed |
| 0.74707 | 1 | 0.673 | 0.023154 | 24 | Cox8a | Mixed |
| 0.562868 | 0.688 | 0.209 | 0.024253 | 24 | Ndufb8 | Mixed |
| 0.497567 | 0.438 | 0.094 | 0.028402 | 24 | Sec11c | Mixed |
| 0.434355 | 0.5 | 0.111 | 0.028741 | 24 | Ms4a6d | Mixed |
| 0.423161 | 0.562 | 0.137 | 0.029717 | 24 | Sod3 | Mixed |
| 0.644431 | 1 | 0.756 | 0.038429 | 24 | Cox4i1 | Mixed |
| 0.31196 | 0.312 | 0.051 | 0.042893 | 24 | Orai1 | Mixed |
| 0.374484 | 0.312 | 0.052 | 0.043179 | 24 | Rbpms | Mixed |
| 0.583341 | 0.875 | 0.338 | 0.044905 | 24 | Ldha | Mixed |
