# Supplementary material for: Single Cell Sequencing Identifies Distinct Cellular Alterations in Impaired Aged and Diabetic Wounds
Source: Aging Cell. 2025 Nov 4;24(12):e70217. doi: 10.1111/acel.70217 (PMC12686554; doi:10.1111/acel.70217)
Supplement: Supplementary file 8 — Table S6: Differential gene expression of all stromal cell in wounded gingiva from diabetic wounded gingiva and NG‐Aged mice in 7 day wounds. Differential expression gene in stromal cells from diabetic versus NG‐Aged mice. Each comparison run is noted in column “comparison.” DEG was analyzed using MAST method. Values are expressed as average of logarithmic fold‐change (avg_log2FC). The genes which have p‐adjusted value (p_val_adj) < 0.05 are considered as differentially expressed. Percent of cells expressing the gene in the cluster is represented by “pct.1” and in all other cluster by “pct.2.” [file ACEL-24-e70217-s002.docx]

Table S6 Differential gene expression of all stromal cell in wounded gingiva from diabetic wounded gingiva and NG-Aged mice at Day 7 post wounding. DEGs in stromal cells from diabetic versus NG-Aged mice. Each comparison run is noted in column “comparison.” DEG was analyzed using MAST method. Values are expressed as average of logarithmic fold-change (avg_log2FC). The genes which have *p*-adjusted value (*p*_val_adj) < 0.05 are considered as differentially expressed. Percentage of cells expressing the gene in the cluster is represented by “pct.1” and in all other cluster by “pct.2.”

| p_val | avg_log2FC | pct.1 | pct.2 | p_val_adj | gene | comparison | cell_type |
| --- | --- | --- | --- | --- | --- | --- | --- |
| 1.09E-242 | 3.287053766 | 0.411 | 0.052 | 1.99E-238 | Hk2 | Diabetic_WT/Old_WT | Stromal |
| 1.95E-53 | 3.267543953 | 0.103 | 0.011 | 3.56E-49 | Arl4c | Diabetic_WT/Old_WT | Stromal |
| 1.38E-100 | 2.661205118 | 0.217 | 0.035 | 2.52E-96 | Gm47283 | Diabetic_WT/Old_WT | Stromal |
| 1.29E-44 | 2.367147357 | 0.107 | 0.019 | 2.36E-40 | Map2k3 | Diabetic_WT/Old_WT | Stromal |
| 1.46E-133 | 2.291611713 | 0.318 | 0.07 | 2.66E-129 | Basp1 | Diabetic_WT/Old_WT | Stromal |
| 7.56E-47 | 2.279079908 | 0.123 | 0.026 | 1.38E-42 | Spsb1 | Diabetic_WT/Old_WT | Stromal |
| 9.15E-59 | 2.272783669 | 0.149 | 0.031 | 1.67E-54 | Bcl10 | Diabetic_WT/Old_WT | Stromal |
| 4.37E-48 | 2.230965389 | 0.129 | 0.029 | 7.97E-44 | Eif4e | Diabetic_WT/Old_WT | Stromal |
| 6.67E-129 | 2.133706414 | 0.345 | 0.093 | 1.22E-124 | Nfkb1 | Diabetic_WT/Old_WT | Stromal |
| 2.65E-233 | 2.05348721 | 0.58 | 0.189 | 4.83E-229 | Btg1 | Diabetic_WT/Old_WT | Stromal |
| 5.24E-39 | 2.023991987 | 0.11 | 0.026 | 9.55E-35 | Btg3 | Diabetic_WT/Old_WT | Stromal |
| 1.44E-39 | 1.976201155 | 0.12 | 0.031 | 2.62E-35 | Aamp | Diabetic_WT/Old_WT | Stromal |
| 3.57E-75 | 1.929038519 | 0.218 | 0.057 | 6.50E-71 | Hmox1 | Diabetic_WT/Old_WT | Stromal |
| 3.54E-41 | 1.915753058 | 0.131 | 0.036 | 6.45E-37 | Rnf19b | Diabetic_WT/Old_WT | Stromal |
| 2.92E-188 | 1.907051162 | 0.514 | 0.172 | 5.33E-184 | Ier5 | Diabetic_WT/Old_WT | Stromal |
| 6.77E-57 | 1.900879781 | 0.175 | 0.047 | 1.23E-52 | Chic2 | Diabetic_WT/Old_WT | Stromal |
| 2.47E-39 | 1.89255011 | 0.132 | 0.038 | 4.51E-35 | Ptpn1 | Diabetic_WT/Old_WT | Stromal |
| 1.57E-32 | 1.879608494 | 0.11 | 0.032 | 2.87E-28 | Mast4 | Diabetic_WT/Old_WT | Stromal |
| 1.95E-52 | 1.876967675 | 0.192 | 0.063 | 3.55E-48 | Col7a1 | Diabetic_WT/Old_WT | Stromal |
| 3.72E-38 | 1.872684336 | 0.121 | 0.033 | 6.77E-34 | Dnajb9 | Diabetic_WT/Old_WT | Stromal |
| 5.09E-41 | 1.851055177 | 0.13 | 0.035 | 9.28E-37 | Plaur | Diabetic_WT/Old_WT | Stromal |
| 5.45E-77 | 1.80957513 | 0.28 | 0.098 | 9.93E-73 | Tcim | Diabetic_WT/Old_WT | Stromal |
| 4.24E-65 | 1.802448478 | 0.211 | 0.061 | 7.74E-61 | Slc3a2 | Diabetic_WT/Old_WT | Stromal |
| 7.80E-32 | 1.798939393 | 0.101 | 0.027 | 1.42E-27 | Ehd1 | Diabetic_WT/Old_WT | Stromal |
| 2.76E-36 | 1.785151186 | 0.139 | 0.046 | 5.03E-32 | Odc1 | Diabetic_WT/Old_WT | Stromal |
| 1.59E-133 | 1.784888731 | 0.433 | 0.156 | 2.90E-129 | Ctsc | Diabetic_WT/Old_WT | Stromal |
| 1.64E-130 | 1.740666351 | 0.421 | 0.147 | 2.98E-126 | Zfand5 | Diabetic_WT/Old_WT | Stromal |
| 1.23E-119 | 1.716449598 | 0.39 | 0.132 | 2.25E-115 | Igfbp3 | Diabetic_WT/Old_WT | Stromal |
| 4.36E-55 | 1.706581824 | 0.213 | 0.074 | 7.94E-51 | Bpgm | Diabetic_WT/Old_WT | Stromal |
| 1.36E-39 | 1.699403719 | 0.155 | 0.053 | 2.48E-35 | Bach1 | Diabetic_WT/Old_WT | Stromal |
| 2.22E-47 | 1.659871682 | 0.174 | 0.056 | 4.05E-43 | Raly | Diabetic_WT/Old_WT | Stromal |
| 1.88E-47 | 1.643661167 | 0.182 | 0.061 | 3.42E-43 | Ndrg1 | Diabetic_WT/Old_WT | Stromal |
| 2.14E-77 | 1.635983811 | 0.28 | 0.095 | 3.90E-73 | Kdm6b | Diabetic_WT/Old_WT | Stromal |
| 4.17E-123 | 1.633547787 | 0.394 | 0.13 | 7.60E-119 | Tomm20 | Diabetic_WT/Old_WT | Stromal |
| 1.38E-45 | 1.609043264 | 0.209 | 0.084 | 2.52E-41 | Uap1 | Diabetic_WT/Old_WT | Stromal |
| 1.34E-31 | 1.599956726 | 0.148 | 0.057 | 2.44E-27 | Matn4 | Diabetic_WT/Old_WT | Stromal |
| 2.25E-25 | 1.587063871 | 0.1 | 0.033 | 4.10E-21 | P3h4 | Diabetic_WT/Old_WT | Stromal |
| 3.11E-56 | 1.573187287 | 0.214 | 0.072 | 5.68E-52 | Sumo1 | Diabetic_WT/Old_WT | Stromal |
| 1.44E-32 | 1.56395919 | 0.131 | 0.045 | 2.63E-28 | Atp6v1e1 | Diabetic_WT/Old_WT | Stromal |
| 2.45E-55 | 1.563362338 | 0.216 | 0.075 | 4.47E-51 | Arl6ip5 | Diabetic_WT/Old_WT | Stromal |
| 1.15E-26 | 1.54693453 | 0.109 | 0.038 | 2.11E-22 | Socs1 | Diabetic_WT/Old_WT | Stromal |
| 5.69E-88 | 1.532277786 | 0.414 | 0.198 | 1.04E-83 | Sgk1 | Diabetic_WT/Old_WT | Stromal |
| 3.98E-30 | 1.524601522 | 0.123 | 0.043 | 7.26E-26 | Herpud1 | Diabetic_WT/Old_WT | Stromal |
| 7.34E-64 | 1.513164353 | 0.248 | 0.087 | 1.34E-59 | Pim1 | Diabetic_WT/Old_WT | Stromal |
| 2.70E-68 | 1.506611012 | 0.274 | 0.101 | 4.92E-64 | Nfe2l2 | Diabetic_WT/Old_WT | Stromal |
| 7.54E-39 | 1.495307032 | 0.167 | 0.061 | 1.38E-34 | Fbn2 | Diabetic_WT/Old_WT | Stromal |
| 5.02E-34 | 1.484381065 | 0.186 | 0.082 | 9.15E-30 | Tsc22d3 | Diabetic_WT/Old_WT | Stromal |
| 1.06E-39 | 1.446999302 | 0.164 | 0.058 | 1.94E-35 | Srsf9 | Diabetic_WT/Old_WT | Stromal |
| 2.29E-58 | 1.44460563 | 0.356 | 0.184 | 4.17E-54 | Ptgs2 | Diabetic_WT/Old_WT | Stromal |
| 1.06E-78 | 1.431006848 | 0.314 | 0.117 | 1.94E-74 | Cd44 | Diabetic_WT/Old_WT | Stromal |
| 3.56E-22 | 1.425858814 | 0.101 | 0.038 | 6.50E-18 | Jtb | Diabetic_WT/Old_WT | Stromal |
| 5.41E-24 | 1.423899961 | 0.121 | 0.049 | 9.87E-20 | Metrnl | Diabetic_WT/Old_WT | Stromal |
| 9.02E-33 | 1.422581107 | 0.147 | 0.055 | 1.64E-28 | Klf13 | Diabetic_WT/Old_WT | Stromal |
| 1.40E-37 | 1.414995855 | 0.168 | 0.063 | 2.55E-33 | Trir | Diabetic_WT/Old_WT | Stromal |
| 2.84E-32 | 1.389820023 | 0.147 | 0.055 | 5.17E-28 | Slc39a7 | Diabetic_WT/Old_WT | Stromal |
| 1.40E-27 | 1.38922251 | 0.128 | 0.049 | 2.55E-23 | Sdf2 | Diabetic_WT/Old_WT | Stromal |
| 2.21E-62 | 1.384557813 | 0.339 | 0.162 | 4.04E-58 | Inhba | Diabetic_WT/Old_WT | Stromal |
| 9.84E-59 | 1.371068568 | 0.261 | 0.102 | 1.79E-54 | Ppp2ca | Diabetic_WT/Old_WT | Stromal |
| 1.64E-105 | 1.357144993 | 0.485 | 0.236 | 2.98E-101 | Errfi1 | Diabetic_WT/Old_WT | Stromal |
| 1.01E-102 | 1.351266563 | 0.433 | 0.184 | 1.84E-98 | Atf4 | Diabetic_WT/Old_WT | Stromal |
| 6.67E-52 | 1.347454894 | 0.243 | 0.097 | 1.22E-47 | Akap13 | Diabetic_WT/Old_WT | Stromal |
| 1.84E-50 | 1.340804346 | 0.225 | 0.086 | 3.36E-46 | Tiparp | Diabetic_WT/Old_WT | Stromal |
| 5.37E-25 | 1.321336641 | 0.121 | 0.047 | 9.80E-21 | Luzp1 | Diabetic_WT/Old_WT | Stromal |
| 4.81E-25 | 1.314123151 | 0.119 | 0.046 | 8.77E-21 | Dync1i2 | Diabetic_WT/Old_WT | Stromal |
| 1.23E-84 | 1.303712921 | 0.408 | 0.187 | 2.23E-80 | Mcl1 | Diabetic_WT/Old_WT | Stromal |
| 2.86E-36 | 1.30250881 | 0.196 | 0.085 | 5.21E-32 | Jak1 | Diabetic_WT/Old_WT | Stromal |
| 1.23E-30 | 1.302044738 | 0.153 | 0.062 | 2.25E-26 | Col27a1 | Diabetic_WT/Old_WT | Stromal |
| 8.53E-24 | 1.284694142 | 0.122 | 0.05 | 1.55E-19 | Ankrd11 | Diabetic_WT/Old_WT | Stromal |
| 4.29E-58 | 1.283925701 | 0.288 | 0.123 | 7.82E-54 | Plat | Diabetic_WT/Old_WT | Stromal |
| 1.55E-26 | 1.26849855 | 0.142 | 0.06 | 2.83E-22 | Dnajb6 | Diabetic_WT/Old_WT | Stromal |
| 3.58E-20 | 1.266963275 | 0.142 | 0.07 | 6.53E-16 | Ugdh | Diabetic_WT/Old_WT | Stromal |
| 9.89E-36 | 1.266412313 | 0.256 | 0.135 | 1.80E-31 | Rgcc | Diabetic_WT/Old_WT | Stromal |
| 1.23E-65 | 1.260431794 | 0.337 | 0.153 | 2.25E-61 | Tnfrsf12a | Diabetic_WT/Old_WT | Stromal |
| 1.56E-18 | 1.241144211 | 0.103 | 0.045 | 2.85E-14 | Bag3 | Diabetic_WT/Old_WT | Stromal |
| 7.60E-20 | 1.238331497 | 0.108 | 0.046 | 1.38E-15 | Tm2d2 | Diabetic_WT/Old_WT | Stromal |
| 5.68E-63 | 1.236547494 | 0.32 | 0.141 | 1.04E-58 | Hexa | Diabetic_WT/Old_WT | Stromal |
| 5.03E-26 | 1.232709653 | 0.137 | 0.057 | 9.18E-22 | Pdcd6 | Diabetic_WT/Old_WT | Stromal |
| 2.05E-76 | 1.232210205 | 0.413 | 0.205 | 3.73E-72 | Top1 | Diabetic_WT/Old_WT | Stromal |
| 2.31E-22 | 1.224926708 | 0.135 | 0.061 | 4.21E-18 | Dnajb1 | Diabetic_WT/Old_WT | Stromal |
| 1.24E-22 | 1.210312903 | 0.127 | 0.055 | 2.26E-18 | Pxdn | Diabetic_WT/Old_WT | Stromal |
| 5.50E-29 | 1.209280193 | 0.159 | 0.068 | 1.00E-24 | Ndufs2 | Diabetic_WT/Old_WT | Stromal |
| 7.96E-34 | 1.208788486 | 0.185 | 0.08 | 1.45E-29 | Eif6 | Diabetic_WT/Old_WT | Stromal |
| 6.04E-35 | 1.206390757 | 0.19 | 0.082 | 1.10E-30 | Pgrmc1 | Diabetic_WT/Old_WT | Stromal |
| 1.23E-48 | 1.192232995 | 0.261 | 0.114 | 2.25E-44 | Rheb | Diabetic_WT/Old_WT | Stromal |
| 1.11E-17 | 1.181555414 | 0.101 | 0.044 | 2.02E-13 | Dpm1 | Diabetic_WT/Old_WT | Stromal |
| 3.35E-23 | 1.172838197 | 0.133 | 0.058 | 6.10E-19 | Bex3 | Diabetic_WT/Old_WT | Stromal |
| 1.76E-24 | 1.15980436 | 0.138 | 0.06 | 3.20E-20 | Timm23 | Diabetic_WT/Old_WT | Stromal |
| 8.51E-134 | 1.153527815 | 0.692 | 0.422 | 1.55E-129 | Cebpb | Diabetic_WT/Old_WT | Stromal |
| 2.15E-20 | 1.146365986 | 0.125 | 0.057 | 3.93E-16 | Srpx | Diabetic_WT/Old_WT | Stromal |
| 4.31E-77 | 1.142418801 | 0.491 | 0.284 | 7.86E-73 | Cpxm1 | Diabetic_WT/Old_WT | Stromal |
| 1.05E-65 | 1.140737048 | 0.362 | 0.171 | 1.92E-61 | Cox7a2l | Diabetic_WT/Old_WT | Stromal |
| 1.49E-31 | 1.133490641 | 0.193 | 0.09 | 2.72E-27 | Wwtr1 | Diabetic_WT/Old_WT | Stromal |
| 4.28E-23 | 1.126290209 | 0.142 | 0.065 | 7.80E-19 | Fam104a | Diabetic_WT/Old_WT | Stromal |
| 5.31E-19 | 1.121969332 | 0.117 | 0.053 | 9.67E-15 | Rab10 | Diabetic_WT/Old_WT | Stromal |
| 1.88E-43 | 1.111176557 | 0.259 | 0.12 | 3.43E-39 | Tgfb2 | Diabetic_WT/Old_WT | Stromal |
| 2.62E-20 | 1.111137498 | 0.13 | 0.061 | 4.78E-16 | Litaf | Diabetic_WT/Old_WT | Stromal |
| 4.31E-16 | 1.107079281 | 0.102 | 0.048 | 7.86E-12 | Myc | Diabetic_WT/Old_WT | Stromal |
| 1.71E-16 | 1.105724001 | 0.103 | 0.048 | 3.11E-12 | Lrpap1 | Diabetic_WT/Old_WT | Stromal |
| 1.50E-24 | 1.102022409 | 0.155 | 0.072 | 2.74E-20 | Brk1 | Diabetic_WT/Old_WT | Stromal |
| 3.55E-40 | 1.097822134 | 0.238 | 0.109 | 6.47E-36 | Snx3 | Diabetic_WT/Old_WT | Stromal |
| 5.34E-38 | 1.096325303 | 0.235 | 0.11 | 9.73E-34 | Brd2 | Diabetic_WT/Old_WT | Stromal |
| 6.50E-38 | 1.093999475 | 0.238 | 0.113 | 1.19E-33 | Serinc3 | Diabetic_WT/Old_WT | Stromal |
| 9.48E-40 | 1.090439273 | 0.251 | 0.121 | 1.73E-35 | Atraid | Diabetic_WT/Old_WT | Stromal |
| 9.07E-21 | 1.086572901 | 0.135 | 0.064 | 1.65E-16 | Ube2d2a | Diabetic_WT/Old_WT | Stromal |
| 5.17E-23 | 1.086358677 | 0.153 | 0.073 | 9.42E-19 | Tnfrsf1a | Diabetic_WT/Old_WT | Stromal |
| 1.68E-24 | 1.080223629 | 0.132 | 0.055 | 3.06E-20 | Marcksl1 | Diabetic_WT/Old_WT | Stromal |
| 1.05E-22 | 1.079500681 | 0.155 | 0.076 | 1.91E-18 | Eif1b | Diabetic_WT/Old_WT | Stromal |
| 6.53E-32 | 1.077468741 | 0.203 | 0.096 | 1.19E-27 | Rap1b | Diabetic_WT/Old_WT | Stromal |
| 1.82E-36 | 1.074379873 | 0.232 | 0.111 | 3.31E-32 | Mpc1 | Diabetic_WT/Old_WT | Stromal |
| 2.58E-26 | 1.063739986 | 0.177 | 0.086 | 4.71E-22 | Txnl1 | Diabetic_WT/Old_WT | Stromal |
| 2.05E-22 | 1.059829235 | 0.142 | 0.066 | 3.74E-18 | Adamts4 | Diabetic_WT/Old_WT | Stromal |
| 6.23E-15 | 1.059654353 | 0.102 | 0.05 | 1.14E-10 | Yipf3 | Diabetic_WT/Old_WT | Stromal |
| 2.58E-18 | 1.04900961 | 0.127 | 0.062 | 4.71E-14 | Zfp706 | Diabetic_WT/Old_WT | Stromal |
| 9.31E-19 | 1.044795003 | 0.128 | 0.062 | 1.70E-14 | Phb2 | Diabetic_WT/Old_WT | Stromal |
| 2.99E-19 | 1.03570838 | 0.131 | 0.063 | 5.46E-15 | Cfdp1 | Diabetic_WT/Old_WT | Stromal |
| 3.40E-57 | 1.035427389 | 0.38 | 0.199 | 6.19E-53 | Mdk | Diabetic_WT/Old_WT | Stromal |
| 6.20E-20 | 1.033357024 | 0.142 | 0.07 | 1.13E-15 | Sdcbp | Diabetic_WT/Old_WT | Stromal |
| 2.74E-18 | 1.03205209 | 0.13 | 0.064 | 4.99E-14 | Cmtm3 | Diabetic_WT/Old_WT | Stromal |
| 2.05E-52 | 1.029511131 | 0.367 | 0.197 | 3.74E-48 | Sdc1 | Diabetic_WT/Old_WT | Stromal |
| 6.16E-17 | 1.026289534 | 0.125 | 0.063 | 1.12E-12 | Ppa1 | Diabetic_WT/Old_WT | Stromal |
| 5.24E-14 | 1.020835104 | 0.1 | 0.05 | 9.55E-10 | Dcun1d5 | Diabetic_WT/Old_WT | Stromal |
| 1.72E-16 | 1.01930925 | 0.115 | 0.056 | 3.14E-12 | Rab7 | Diabetic_WT/Old_WT | Stromal |
| 9.18E-13 | 1.018312725 | 0.196 | 0.134 | 1.67E-08 | Gm13889 | Diabetic_WT/Old_WT | Stromal |
| 3.40E-19 | 1.017709003 | 0.138 | 0.068 | 6.19E-15 | Nktr | Diabetic_WT/Old_WT | Stromal |
| 5.56E-16 | 1.015638872 | 0.116 | 0.058 | 1.01E-11 | Ifnar2 | Diabetic_WT/Old_WT | Stromal |
| 2.09E-18 | 1.003001811 | 0.119 | 0.056 | 3.82E-14 | Atp2b1 | Diabetic_WT/Old_WT | Stromal |
| 1.06E-14 | 1.000249332 | 0.102 | 0.05 | 1.93E-10 | Taldo1 | Diabetic_WT/Old_WT | Stromal |
| 7.29E-16 | 0.999976499 | 0.122 | 0.063 | 1.33E-11 | Sntb2 | Diabetic_WT/Old_WT | Stromal |
| 6.71E-22 | 0.997113503 | 0.167 | 0.086 | 1.22E-17 | Stat3 | Diabetic_WT/Old_WT | Stromal |
| 2.62E-30 | 0.995482314 | 0.217 | 0.109 | 4.77E-26 | Vapa | Diabetic_WT/Old_WT | Stromal |
| 7.07E-50 | 0.992227148 | 0.368 | 0.201 | 1.29E-45 | Arid5b | Diabetic_WT/Old_WT | Stromal |
| 1.37E-16 | 0.98766824 | 0.129 | 0.067 | 2.49E-12 | Eif1a | Diabetic_WT/Old_WT | Stromal |
| 7.92E-18 | 0.986544083 | 0.133 | 0.067 | 1.44E-13 | Eif4a2 | Diabetic_WT/Old_WT | Stromal |
| 1.50E-21 | 0.984417705 | 0.143 | 0.068 | 2.74E-17 | Cpxm2 | Diabetic_WT/Old_WT | Stromal |
| 2.22E-26 | 0.976882919 | 0.196 | 0.099 | 4.04E-22 | Psma5 | Diabetic_WT/Old_WT | Stromal |
| 1.92E-16 | 0.970794421 | 0.114 | 0.055 | 3.51E-12 | Aopep | Diabetic_WT/Old_WT | Stromal |
| 1.96E-36 | 0.96921223 | 0.301 | 0.168 | 3.57E-32 | Lsp1 | Diabetic_WT/Old_WT | Stromal |
| 5.01E-37 | 0.965825395 | 0.259 | 0.133 | 9.14E-33 | Eln | Diabetic_WT/Old_WT | Stromal |
| 3.80E-14 | 0.964383383 | 0.116 | 0.061 | 6.93E-10 | Zfp703 | Diabetic_WT/Old_WT | Stromal |
| 9.58E-27 | 0.964092592 | 0.196 | 0.099 | 1.75E-22 | Epha3 | Diabetic_WT/Old_WT | Stromal |
| 1.07E-23 | 0.959597474 | 0.208 | 0.115 | 1.96E-19 | Vasn | Diabetic_WT/Old_WT | Stromal |
| 7.81E-14 | 0.959280433 | 0.115 | 0.061 | 1.42E-09 | Gabarapl2 | Diabetic_WT/Old_WT | Stromal |
| 1.77E-22 | 0.95591958 | 0.176 | 0.091 | 3.22E-18 | Tmco1 | Diabetic_WT/Old_WT | Stromal |
| 7.47E-32 | 0.95280462 | 0.232 | 0.118 | 1.36E-27 | Atp6v0b | Diabetic_WT/Old_WT | Stromal |
| 9.53E-15 | 0.952226661 | 0.117 | 0.061 | 1.74E-10 | U2af1 | Diabetic_WT/Old_WT | Stromal |
| 4.62E-37 | 0.951294171 | 0.268 | 0.137 | 8.42E-33 | Psmd8 | Diabetic_WT/Old_WT | Stromal |
| 7.02E-16 | 0.941047078 | 0.123 | 0.063 | 1.28E-11 | Ube2n | Diabetic_WT/Old_WT | Stromal |
| 2.10E-30 | 0.939838676 | 0.228 | 0.117 | 3.83E-26 | P3h3 | Diabetic_WT/Old_WT | Stromal |
| 2.24E-14 | 0.939365615 | 0.122 | 0.065 | 4.09E-10 | Socs2 | Diabetic_WT/Old_WT | Stromal |
| 7.83E-19 | 0.939109037 | 0.152 | 0.08 | 1.43E-14 | Eif4e2 | Diabetic_WT/Old_WT | Stromal |
| 3.66E-19 | 0.935378328 | 0.152 | 0.079 | 6.68E-15 | Tra2a | Diabetic_WT/Old_WT | Stromal |
| 7.87E-33 | 0.933472224 | 0.255 | 0.135 | 1.43E-28 | Bzw1 | Diabetic_WT/Old_WT | Stromal |
| 2.98E-39 | 0.926982587 | 0.338 | 0.194 | 5.44E-35 | Nr4a2 | Diabetic_WT/Old_WT | Stromal |
| 7.27E-18 | 0.926584778 | 0.144 | 0.075 | 1.32E-13 | Eif3m | Diabetic_WT/Old_WT | Stromal |
| 2.06E-31 | 0.922954352 | 0.252 | 0.135 | 3.76E-27 | Ube2b | Diabetic_WT/Old_WT | Stromal |
| 7.55E-18 | 0.919551768 | 0.145 | 0.076 | 1.38E-13 | Hnrnpc | Diabetic_WT/Old_WT | Stromal |
| 5.05E-54 | 0.917328913 | 0.398 | 0.218 | 9.21E-50 | Scand1 | Diabetic_WT/Old_WT | Stromal |
| 2.52E-36 | 0.915538594 | 0.262 | 0.133 | 4.59E-32 | Cdkn1a | Diabetic_WT/Old_WT | Stromal |
| 9.17E-18 | 0.915490248 | 0.15 | 0.08 | 1.67E-13 | Pdlim4 | Diabetic_WT/Old_WT | Stromal |
| 1.48E-12 | 0.91504044 | 0.14 | 0.084 | 2.70E-08 | Ltbp2 | Diabetic_WT/Old_WT | Stromal |
| 2.74E-38 | 0.913493551 | 0.265 | 0.132 | 5.00E-34 | Omd | Diabetic_WT/Old_WT | Stromal |
| 1.55E-38 | 0.91200397 | 0.318 | 0.178 | 2.83E-34 | Scarf2 | Diabetic_WT/Old_WT | Stromal |
| 2.16E-19 | 0.909803791 | 0.165 | 0.089 | 3.95E-15 | Xbp1 | Diabetic_WT/Old_WT | Stromal |
| 2.34E-13 | 0.908610989 | 0.116 | 0.063 | 4.27E-09 | mt-Atp8 | Diabetic_WT/Old_WT | Stromal |
| 3.06E-61 | 0.907993634 | 0.444 | 0.245 | 5.59E-57 | Slc38a2 | Diabetic_WT/Old_WT | Stromal |
| 2.27E-06 | 0.90726624 | 0.103 | 0.069 | 0.041403385 | Has1 | Diabetic_WT/Old_WT | Stromal |
| 1.95E-50 | 0.906871749 | 0.469 | 0.301 | 3.55E-46 | Dnaja1 | Diabetic_WT/Old_WT | Stromal |
| 1.49E-18 | 0.902922308 | 0.151 | 0.08 | 2.73E-14 | Rora | Diabetic_WT/Old_WT | Stromal |
| 4.66E-31 | 0.901334815 | 0.259 | 0.141 | 8.50E-27 | Hnrnpf | Diabetic_WT/Old_WT | Stromal |
| 2.87E-73 | 0.900421948 | 0.523 | 0.306 | 5.24E-69 | Fgfr1 | Diabetic_WT/Old_WT | Stromal |
| 3.70E-24 | 0.899940498 | 0.246 | 0.146 | 6.75E-20 | Pnp | Diabetic_WT/Old_WT | Stromal |
| 1.09E-13 | 0.898844579 | 0.114 | 0.06 | 1.99E-09 | Ubxn4 | Diabetic_WT/Old_WT | Stromal |
| 2.97E-33 | 0.893781731 | 0.296 | 0.168 | 5.42E-29 | Il11ra1 | Diabetic_WT/Old_WT | Stromal |
| 2.57E-19 | 0.892264705 | 0.177 | 0.098 | 4.69E-15 | Ppp1r2 | Diabetic_WT/Old_WT | Stromal |
| 6.45E-21 | 0.888314988 | 0.171 | 0.09 | 1.18E-16 | Clk1 | Diabetic_WT/Old_WT | Stromal |
| 9.63E-32 | 0.888286462 | 0.26 | 0.14 | 1.76E-27 | Ergic3 | Diabetic_WT/Old_WT | Stromal |
| 2.10E-12 | 0.887618938 | 0.101 | 0.053 | 3.84E-08 | Atp1a1 | Diabetic_WT/Old_WT | Stromal |
| 2.30E-20 | 0.885694717 | 0.181 | 0.099 | 4.20E-16 | Ostf1 | Diabetic_WT/Old_WT | Stromal |
| 7.08E-22 | 0.88333158 | 0.192 | 0.105 | 1.29E-17 | Ube2i | Diabetic_WT/Old_WT | Stromal |
| 2.77E-15 | 0.88333158 | 0.12 | 0.062 | 5.05E-11 | Rap1a | Diabetic_WT/Old_WT | Stromal |
| 9.88E-80 | 0.878620735 | 0.551 | 0.31 | 1.80E-75 | Klf9 | Diabetic_WT/Old_WT | Stromal |
| 4.66E-15 | 0.876962055 | 0.136 | 0.075 | 8.50E-11 | Tecr | Diabetic_WT/Old_WT | Stromal |
| 3.40E-13 | 0.875203699 | 0.107 | 0.056 | 6.19E-09 | Rsrc2 | Diabetic_WT/Old_WT | Stromal |
| 1.60E-45 | 0.870758995 | 0.411 | 0.246 | 2.91E-41 | Ifrd1 | Diabetic_WT/Old_WT | Stromal |
| 3.17E-14 | 0.870392524 | 0.132 | 0.073 | 5.79E-10 | Rab34 | Diabetic_WT/Old_WT | Stromal |
| 3.26E-52 | 0.860180616 | 0.393 | 0.213 | 5.94E-48 | Arpc3 | Diabetic_WT/Old_WT | Stromal |
| 2.02E-14 | 0.85721874 | 0.129 | 0.07 | 3.68E-10 | Skil | Diabetic_WT/Old_WT | Stromal |
| 2.38E-21 | 0.856364532 | 0.193 | 0.107 | 4.33E-17 | Chmp4b | Diabetic_WT/Old_WT | Stromal |
| 4.49E-234 | 0.853502226 | 0.937 | 0.855 | 8.19E-230 | H3f3a | Diabetic_WT/Old_WT | Stromal |
| 3.97E-66 | 0.847203415 | 0.502 | 0.293 | 7.25E-62 | Atp6v0c | Diabetic_WT/Old_WT | Stromal |
| 2.83E-26 | 0.845768167 | 0.247 | 0.141 | 5.17E-22 | Vdac2 | Diabetic_WT/Old_WT | Stromal |
| 3.68E-48 | 0.845494759 | 0.4 | 0.23 | 6.70E-44 | Oaf | Diabetic_WT/Old_WT | Stromal |
| 6.70E-14 | 0.842879536 | 0.128 | 0.071 | 1.22E-09 | Cct4 | Diabetic_WT/Old_WT | Stromal |
| 1.35E-46 | 0.841931464 | 0.391 | 0.224 | 2.46E-42 | Ctsz | Diabetic_WT/Old_WT | Stromal |
| 1.43E-43 | 0.835181293 | 0.366 | 0.206 | 2.61E-39 | Itm2c | Diabetic_WT/Old_WT | Stromal |
| 6.12E-50 | 0.830103592 | 0.427 | 0.252 | 1.11E-45 | Rtn4 | Diabetic_WT/Old_WT | Stromal |
| 2.42E-16 | 0.828260198 | 0.155 | 0.087 | 4.41E-12 | Glrx3 | Diabetic_WT/Old_WT | Stromal |
| 1.13E-53 | 0.82479861 | 0.534 | 0.363 | 2.06E-49 | Tsc22d1 | Diabetic_WT/Old_WT | Stromal |
| 9.99E-16 | 0.82238223 | 0.159 | 0.091 | 1.82E-11 | Bcl7c | Diabetic_WT/Old_WT | Stromal |
| 7.55E-30 | 0.82166856 | 0.277 | 0.158 | 1.38E-25 | Sec11a | Diabetic_WT/Old_WT | Stromal |
| 1.22E-50 | 0.821635128 | 0.517 | 0.339 | 2.22E-46 | Klf4 | Diabetic_WT/Old_WT | Stromal |
| 1.83E-197 | 0.820368078 | 0.994 | 0.937 | 3.33E-193 | Malat1 | Diabetic_WT/Old_WT | Stromal |
| 6.72E-12 | 0.818561325 | 0.118 | 0.067 | 1.22E-07 | Sin3b | Diabetic_WT/Old_WT | Stromal |
| 9.43E-13 | 0.815903653 | 0.183 | 0.119 | 1.72E-08 | Tnfaip2 | Diabetic_WT/Old_WT | Stromal |
| 6.86E-14 | 0.813253144 | 0.134 | 0.076 | 1.25E-09 | Ryk | Diabetic_WT/Old_WT | Stromal |
| 7.42E-22 | 0.813040258 | 0.21 | 0.119 | 1.35E-17 | Tpm3 | Diabetic_WT/Old_WT | Stromal |
| 6.70E-34 | 0.811971759 | 0.34 | 0.206 | 1.22E-29 | Sub1 | Diabetic_WT/Old_WT | Stromal |
| 1.35E-11 | 0.810980736 | 0.102 | 0.056 | 2.46E-07 | Kcnq1ot1 | Diabetic_WT/Old_WT | Stromal |
| 2.89E-56 | 0.809426387 | 0.507 | 0.316 | 5.26E-52 | Col4a2 | Diabetic_WT/Old_WT | Stromal |
| 7.31E-17 | 0.80086942 | 0.177 | 0.103 | 1.33E-12 | Srsf7 | Diabetic_WT/Old_WT | Stromal |
| 1.09E-16 | 0.798994224 | 0.178 | 0.104 | 1.98E-12 | Tspan3 | Diabetic_WT/Old_WT | Stromal |
| 6.61E-15 | 0.798683754 | 0.154 | 0.089 | 1.21E-10 | Zc3h15 | Diabetic_WT/Old_WT | Stromal |
| 5.26E-20 | 0.793267882 | 0.199 | 0.114 | 9.58E-16 | Cct2 | Diabetic_WT/Old_WT | Stromal |
| 1.65E-25 | 0.792819287 | 0.432 | 0.322 | 3.01E-21 | Gem | Diabetic_WT/Old_WT | Stromal |
| 2.48E-10 | 0.789209403 | 0.107 | 0.062 | 4.51E-06 | Qk | Diabetic_WT/Old_WT | Stromal |
| 4.74E-50 | 0.77853539 | 0.453 | 0.27 | 8.64E-46 | Ube2s | Diabetic_WT/Old_WT | Stromal |
| 3.05E-14 | 0.77532439 | 0.162 | 0.097 | 5.56E-10 | Hnrnph1 | Diabetic_WT/Old_WT | Stromal |
| 2.37E-14 | 0.772874932 | 0.138 | 0.078 | 4.32E-10 | Tgfb1 | Diabetic_WT/Old_WT | Stromal |
| 1.38E-11 | 0.769990107 | 0.125 | 0.073 | 2.52E-07 | Bcap31 | Diabetic_WT/Old_WT | Stromal |
| 6.77E-30 | 0.76881514 | 0.337 | 0.212 | 1.24E-25 | Sqstm1 | Diabetic_WT/Old_WT | Stromal |
| 8.32E-25 | 0.76349181 | 0.262 | 0.156 | 1.52E-20 | Ywhaq | Diabetic_WT/Old_WT | Stromal |
| 2.84E-13 | 0.762025284 | 0.156 | 0.095 | 5.17E-09 | Rgs3 | Diabetic_WT/Old_WT | Stromal |
| 1.69E-18 | 0.761562617 | 0.194 | 0.113 | 3.07E-14 | Ppp1r15a | Diabetic_WT/Old_WT | Stromal |
| 2.99E-16 | 0.757800698 | 0.176 | 0.104 | 5.45E-12 | Rnh1 | Diabetic_WT/Old_WT | Stromal |
| 1.99E-119 | 0.756468864 | 0.89 | 0.802 | 3.62E-115 | Ubc | Diabetic_WT/Old_WT | Stromal |
| 2.34E-56 | 0.753331181 | 0.6 | 0.426 | 4.26E-52 | Col11a1 | Diabetic_WT/Old_WT | Stromal |
| 1.62E-09 | 0.747979727 | 0.11 | 0.066 | 2.95E-05 | Anapc5 | Diabetic_WT/Old_WT | Stromal |
| 1.42E-32 | 0.747676481 | 0.391 | 0.254 | 2.60E-28 | Itm2a | Diabetic_WT/Old_WT | Stromal |
| 3.76E-23 | 0.743581451 | 0.26 | 0.158 | 6.86E-19 | Ptov1 | Diabetic_WT/Old_WT | Stromal |
| 2.79E-18 | 0.741184682 | 0.212 | 0.129 | 5.08E-14 | Dnajc3 | Diabetic_WT/Old_WT | Stromal |
| 1.82E-16 | 0.739787454 | 0.193 | 0.117 | 3.32E-12 | St13 | Diabetic_WT/Old_WT | Stromal |
| 1.05E-10 | 0.737199075 | 0.119 | 0.071 | 1.92E-06 | Cct8 | Diabetic_WT/Old_WT | Stromal |
| 4.96E-09 | 0.730480092 | 0.103 | 0.062 | 9.03E-05 | Map2k2 | Diabetic_WT/Old_WT | Stromal |
| 4.65E-22 | 0.73026416 | 0.245 | 0.147 | 8.47E-18 | Pgam1 | Diabetic_WT/Old_WT | Stromal |
| 3.48E-09 | 0.726362234 | 0.105 | 0.063 | 6.35E-05 | Ctsa | Diabetic_WT/Old_WT | Stromal |
| 4.20E-31 | 0.725748393 | 0.344 | 0.212 | 7.66E-27 | Eif5 | Diabetic_WT/Old_WT | Stromal |
| 2.61E-30 | 0.725169694 | 0.335 | 0.207 | 4.75E-26 | Erp29 | Diabetic_WT/Old_WT | Stromal |
| 2.11E-26 | 0.720639724 | 0.303 | 0.187 | 3.85E-22 | Polr1d | Diabetic_WT/Old_WT | Stromal |
| 1.36E-27 | 0.718762745 | 0.384 | 0.261 | 2.47E-23 | Lrrc15 | Diabetic_WT/Old_WT | Stromal |
| 8.28E-09 | 0.718046966 | 0.104 | 0.063 | 0.000151041 | Mapre1 | Diabetic_WT/Old_WT | Stromal |
| 1.64E-10 | 0.716594856 | 0.143 | 0.091 | 2.99E-06 | Ddit4 | Diabetic_WT/Old_WT | Stromal |
| 6.19E-10 | 0.710817457 | 0.125 | 0.078 | 1.13E-05 | Csrnp1 | Diabetic_WT/Old_WT | Stromal |
| 2.76E-16 | 0.710358713 | 0.204 | 0.127 | 5.03E-12 | Rdx | Diabetic_WT/Old_WT | Stromal |
| 2.65E-47 | 0.707278583 | 0.572 | 0.406 | 4.84E-43 | Cebpd | Diabetic_WT/Old_WT | Stromal |
| 2.70E-11 | 0.706203892 | 0.134 | 0.081 | 4.93E-07 | Emc10 | Diabetic_WT/Old_WT | Stromal |
| 5.52E-69 | 0.703271273 | 0.721 | 0.711 | 1.01E-64 | Rpl29 | Diabetic_WT/Old_WT | Stromal |
| 5.02E-11 | 0.698777914 | 0.136 | 0.083 | 9.15E-07 | Smim14 | Diabetic_WT/Old_WT | Stromal |
| 5.96E-10 | 0.694886491 | 0.129 | 0.081 | 1.09E-05 | 9530068E07Rik | Diabetic_WT/Old_WT | Stromal |
| 1.89E-09 | 0.69400686 | 0.129 | 0.082 | 3.44E-05 | Prmt1 | Diabetic_WT/Old_WT | Stromal |
| 2.05E-21 | 0.686263315 | 0.281 | 0.18 | 3.75E-17 | Lamb1 | Diabetic_WT/Old_WT | Stromal |
| 0 | 0.685469901 | 0.999 | 0.993 | 0 | Tpt1 | Diabetic_WT/Old_WT | Stromal |
| 6.05E-09 | 0.685002864 | 0.115 | 0.071 | 0.000110241 | Ext1 | Diabetic_WT/Old_WT | Stromal |
| 1.97E-181 | 0.684776714 | 0.974 | 0.936 | 3.58E-177 | Ftl1 | Diabetic_WT/Old_WT | Stromal |
| 2.41E-10 | 0.682462871 | 0.176 | 0.121 | 4.40E-06 | Tuba1b | Diabetic_WT/Old_WT | Stromal |
| 1.79E-38 | 0.678249399 | 0.432 | 0.272 | 3.26E-34 | mt-Nd5 | Diabetic_WT/Old_WT | Stromal |
| 1.18E-11 | 0.6776179 | 0.157 | 0.099 | 2.16E-07 | Mrc2 | Diabetic_WT/Old_WT | Stromal |
| 3.25E-16 | 0.676212014 | 0.215 | 0.136 | 5.93E-12 | Mpc2 | Diabetic_WT/Old_WT | Stromal |
| 2.20E-45 | 0.673612989 | 0.4 | 0.236 | 4.00E-41 | Mfap4 | Diabetic_WT/Old_WT | Stromal |
| 4.52E-11 | 0.672564484 | 0.155 | 0.099 | 8.25E-07 | Sh3pxd2a | Diabetic_WT/Old_WT | Stromal |
| 7.73E-20 | 0.670139125 | 0.243 | 0.15 | 1.41E-15 | Fbln5 | Diabetic_WT/Old_WT | Stromal |
| 1.88E-09 | 0.669533386 | 0.134 | 0.086 | 3.43E-05 | Myl12b | Diabetic_WT/Old_WT | Stromal |
| 4.23E-09 | 0.669076208 | 0.116 | 0.072 | 7.71E-05 | Wipi1 | Diabetic_WT/Old_WT | Stromal |
| 4.68E-08 | 0.668737529 | 0.121 | 0.08 | 0.000853663 | Lmo4 | Diabetic_WT/Old_WT | Stromal |
| 3.80E-12 | 0.667758528 | 0.154 | 0.096 | 6.94E-08 | Sf3b1 | Diabetic_WT/Old_WT | Stromal |
| 1.19E-22 | 0.665740145 | 0.295 | 0.189 | 2.16E-18 | Paip2 | Diabetic_WT/Old_WT | Stromal |
| 5.93E-09 | 0.663550372 | 0.116 | 0.072 | 0.000108194 | Wnk1 | Diabetic_WT/Old_WT | Stromal |
| 1.04E-08 | 0.659825538 | 0.113 | 0.07 | 0.000189997 | Cdc37 | Diabetic_WT/Old_WT | Stromal |
| 1.22E-51 | 0.659502641 | 0.578 | 0.409 | 2.23E-47 | Tmed9 | Diabetic_WT/Old_WT | Stromal |
| 1.01E-10 | 0.658686003 | 0.138 | 0.086 | 1.85E-06 | Nisch | Diabetic_WT/Old_WT | Stromal |
| 4.05E-43 | 0.657450173 | 0.513 | 0.352 | 7.38E-39 | Arpc2 | Diabetic_WT/Old_WT | Stromal |
| 1.00E-13 | 0.655921084 | 0.198 | 0.129 | 1.82E-09 | Lrrc17 | Diabetic_WT/Old_WT | Stromal |
| 1.82E-18 | 0.655505902 | 0.279 | 0.191 | 3.32E-14 | Ecrg4 | Diabetic_WT/Old_WT | Stromal |
| 1.34E-10 | 0.655062592 | 0.148 | 0.094 | 2.44E-06 | Rab5if | Diabetic_WT/Old_WT | Stromal |
| 1.07E-12 | 0.652994212 | 0.176 | 0.113 | 1.96E-08 | Bax | Diabetic_WT/Old_WT | Stromal |
| 1.48E-13 | 0.652416485 | 0.182 | 0.115 | 2.70E-09 | Crlf1 | Diabetic_WT/Old_WT | Stromal |
| 6.56E-09 | 0.651458106 | 0.179 | 0.127 | 0.000119603 | Hes1 | Diabetic_WT/Old_WT | Stromal |
| 4.17E-32 | 0.650446785 | 0.4 | 0.26 | 7.60E-28 | Ube2d3 | Diabetic_WT/Old_WT | Stromal |
| 4.02E-42 | 0.650156463 | 0.502 | 0.34 | 7.33E-38 | Eif3h | Diabetic_WT/Old_WT | Stromal |
| 1.96E-49 | 0.645426719 | 0.544 | 0.362 | 3.57E-45 | Tmem59 | Diabetic_WT/Old_WT | Stromal |
| 5.70E-27 | 0.644115794 | 0.387 | 0.265 | 1.04E-22 | Vmp1 | Diabetic_WT/Old_WT | Stromal |
| 1.65E-282 | 0.643796861 | 0.998 | 0.985 | 3.01E-278 | mt-Atp6 | Diabetic_WT/Old_WT | Stromal |
| 3.01E-20 | 0.642912023 | 0.279 | 0.181 | 5.49E-16 | Mtdh | Diabetic_WT/Old_WT | Stromal |
| 9.21E-45 | 0.642553074 | 0.586 | 0.427 | 1.68E-40 | Hsp90aa1 | Diabetic_WT/Old_WT | Stromal |
| 2.26E-09 | 0.640761277 | 0.129 | 0.082 | 4.13E-05 | Slc50a1 | Diabetic_WT/Old_WT | Stromal |
| 4.15E-07 | 0.639226903 | 0.106 | 0.069 | 0.007559187 | Ncor1 | Diabetic_WT/Old_WT | Stromal |
| 2.35E-27 | 0.638365467 | 0.363 | 0.238 | 4.28E-23 | Ndfip1 | Diabetic_WT/Old_WT | Stromal |
| 1.22E-68 | 0.636483005 | 0.777 | 0.656 | 2.22E-64 | Pmepa1 | Diabetic_WT/Old_WT | Stromal |
| 1.84E-10 | 0.634796744 | 0.149 | 0.095 | 3.36E-06 | F2r | Diabetic_WT/Old_WT | Stromal |
| 2.04E-08 | 0.63385528 | 0.116 | 0.074 | 0.000372603 | Rbm25 | Diabetic_WT/Old_WT | Stromal |
| 1.19E-15 | 0.633502713 | 0.246 | 0.165 | 2.16E-11 | Tubb2a | Diabetic_WT/Old_WT | Stromal |
| 2.82E-10 | 0.633207283 | 0.137 | 0.086 | 5.15E-06 | Capza2 | Diabetic_WT/Old_WT | Stromal |
| 4.09E-83 | 0.632990567 | 0.87 | 0.824 | 7.46E-79 | Lmna | Diabetic_WT/Old_WT | Stromal |
| 4.07E-47 | 0.630134629 | 0.547 | 0.373 | 7.42E-43 | Mrfap1 | Diabetic_WT/Old_WT | Stromal |
| 1.01E-92 | 0.627950747 | 0.783 | 0.639 | 1.85E-88 | Arf5 | Diabetic_WT/Old_WT | Stromal |
| 5.57E-50 | 0.627716492 | 0.708 | 0.569 | 1.02E-45 | Mmp14 | Diabetic_WT/Old_WT | Stromal |
| 1.65E-09 | 0.624695638 | 0.132 | 0.085 | 3.00E-05 | Zfp91 | Diabetic_WT/Old_WT | Stromal |
| 7.77E-26 | 0.621419457 | 0.366 | 0.245 | 1.42E-21 | Eva1b | Diabetic_WT/Old_WT | Stromal |
| 5.29E-09 | 0.621284983 | 0.142 | 0.094 | 9.64E-05 | Smad7 | Diabetic_WT/Old_WT | Stromal |
| 7.35E-19 | 0.619264465 | 0.279 | 0.184 | 1.34E-14 | Tmbim6 | Diabetic_WT/Old_WT | Stromal |
| 2.65E-07 | 0.618875099 | 0.102 | 0.066 | 0.004825382 | Tmem234 | Diabetic_WT/Old_WT | Stromal |
| 4.95E-32 | 0.617486272 | 0.442 | 0.299 | 9.03E-28 | H2-K1 | Diabetic_WT/Old_WT | Stromal |
| 1.29E-09 | 0.616284112 | 0.109 | 0.065 | 2.35E-05 | Snrnp70 | Diabetic_WT/Old_WT | Stromal |
| 4.06E-08 | 0.613004682 | 0.149 | 0.104 | 0.000739604 | Tnfaip3 | Diabetic_WT/Old_WT | Stromal |
| 7.67E-07 | 0.610872161 | 0.106 | 0.07 | 0.013979996 | Pura | Diabetic_WT/Old_WT | Stromal |
| 2.18E-10 | 0.610406425 | 0.118 | 0.07 | 3.97E-06 | Mest | Diabetic_WT/Old_WT | Stromal |
| 2.80E-17 | 0.609606896 | 0.266 | 0.178 | 5.10E-13 | Tent5a | Diabetic_WT/Old_WT | Stromal |
| 1.79E-22 | 0.608847154 | 0.356 | 0.247 | 3.26E-18 | Hnrnpa1 | Diabetic_WT/Old_WT | Stromal |
| 1.58E-15 | 0.608806354 | 0.236 | 0.156 | 2.88E-11 | Eif3a | Diabetic_WT/Old_WT | Stromal |
| 2.00E-20 | 0.606754616 | 0.326 | 0.223 | 3.65E-16 | Nsa2 | Diabetic_WT/Old_WT | Stromal |
| 1.92E-07 | 0.606622237 | 0.106 | 0.069 | 0.003495456 | Dnajc1 | Diabetic_WT/Old_WT | Stromal |
| 1.69E-09 | 0.604615552 | 0.146 | 0.096 | 3.08E-05 | Eif5b | Diabetic_WT/Old_WT | Stromal |
| 3.46E-66 | 0.599527812 | 0.875 | 0.811 | 6.31E-62 | Col6a3 | Diabetic_WT/Old_WT | Stromal |
| 4.59E-07 | 0.597770605 | 0.131 | 0.091 | 0.008360318 | Frmd6 | Diabetic_WT/Old_WT | Stromal |
| 7.28E-113 | 0.596592366 | 0.913 | 0.709 | 1.33E-108 | mt-Nd2 | Diabetic_WT/Old_WT | Stromal |
| 3.11E-10 | 0.595391832 | 0.181 | 0.124 | 5.68E-06 | Sox4 | Diabetic_WT/Old_WT | Stromal |
| 2.26E-07 | 0.592282798 | 0.124 | 0.084 | 0.004111868 | Kmt2e | Diabetic_WT/Old_WT | Stromal |
| 2.25E-34 | 0.590031624 | 0.547 | 0.388 | 4.11E-30 | H2-D1 | Diabetic_WT/Old_WT | Stromal |
| 4.36E-17 | 0.589118863 | 0.28 | 0.191 | 7.95E-13 | Tceal8 | Diabetic_WT/Old_WT | Stromal |
| 4.23E-22 | 0.587676482 | 0.344 | 0.232 | 7.72E-18 | Twist1 | Diabetic_WT/Old_WT | Stromal |
| 7.68E-23 | 0.587231154 | 0.381 | 0.269 | 1.40E-18 | Col16a1 | Diabetic_WT/Old_WT | Stromal |
| 7.74E-07 | 0.586257418 | 0.109 | 0.072 | 0.014111489 | Vps29 | Diabetic_WT/Old_WT | Stromal |
| 5.01E-36 | 0.586019071 | 0.484 | 0.324 | 9.13E-32 | mt-Nd4l | Diabetic_WT/Old_WT | Stromal |
| 3.34E-87 | 0.583275581 | 0.812 | 0.719 | 6.09E-83 | Chchd2 | Diabetic_WT/Old_WT | Stromal |
| 1.47E-07 | 0.582788871 | 0.106 | 0.068 | 0.002679644 | Ptprd | Diabetic_WT/Old_WT | Stromal |
| 2.61E-22 | 0.580198312 | 0.47 | 0.356 | 4.77E-18 | Phlda1 | Diabetic_WT/Old_WT | Stromal |
| 9.00E-64 | 0.57920678 | 0.717 | 0.562 | 1.64E-59 | Selenok | Diabetic_WT/Old_WT | Stromal |
| 1.85E-09 | 0.576039255 | 0.159 | 0.107 | 3.37E-05 | Ddah2 | Diabetic_WT/Old_WT | Stromal |
| 2.77E-07 | 0.573812272 | 0.121 | 0.082 | 0.005052294 | Tbcb | Diabetic_WT/Old_WT | Stromal |
| 9.21E-09 | 0.572991459 | 0.15 | 0.102 | 0.000167904 | Ube2r2 | Diabetic_WT/Old_WT | Stromal |
| 1.29E-14 | 0.572834066 | 0.245 | 0.166 | 2.35E-10 | Ccnl1 | Diabetic_WT/Old_WT | Stromal |
| 3.53E-35 | 0.569985134 | 0.581 | 0.44 | 6.44E-31 | Col4a1 | Diabetic_WT/Old_WT | Stromal |
| 1.57E-143 | 0.565390539 | 0.953 | 0.906 | 2.86E-139 | Rack1 | Diabetic_WT/Old_WT | Stromal |
| 1.82E-57 | 0.565111534 | 0.696 | 0.536 | 3.31E-53 | Tceal9 | Diabetic_WT/Old_WT | Stromal |
| 4.08E-08 | 0.560176182 | 0.141 | 0.096 | 0.000744672 | Pold4 | Diabetic_WT/Old_WT | Stromal |
| 1.08E-40 | 0.559887253 | 0.494 | 0.331 | 1.97E-36 | Dstn | Diabetic_WT/Old_WT | Stromal |
| 5.74E-190 | 0.559481339 | 0.978 | 0.97 | 1.05E-185 | Rpl7 | Diabetic_WT/Old_WT | Stromal |
| 1.32E-40 | 0.555860235 | 0.588 | 0.435 | 2.40E-36 | Clta | Diabetic_WT/Old_WT | Stromal |
| 1.78E-13 | 0.548699655 | 0.167 | 0.102 | 3.25E-09 | Grem1 | Diabetic_WT/Old_WT | Stromal |
| 2.06E-08 | 0.548004646 | 0.147 | 0.1 | 0.000376228 | Ap2s1 | Diabetic_WT/Old_WT | Stromal |
| 8.54E-51 | 0.547270242 | 0.699 | 0.544 | 1.56E-46 | Ddx5 | Diabetic_WT/Old_WT | Stromal |
| 9.68E-28 | 0.547169103 | 0.398 | 0.263 | 1.77E-23 | Nrep | Diabetic_WT/Old_WT | Stromal |
| 9.61E-24 | 0.545153943 | 0.527 | 0.423 | 1.75E-19 | Mfap2 | Diabetic_WT/Old_WT | Stromal |
| 1.22E-06 | 0.544783223 | 0.12 | 0.083 | 0.0221959 | Cct7 | Diabetic_WT/Old_WT | Stromal |
| 3.08E-48 | 0.544055906 | 0.8 | 0.724 | 5.61E-44 | Lum | Diabetic_WT/Old_WT | Stromal |
| 1.44E-07 | 0.543077672 | 0.142 | 0.098 | 0.00263226 | Fuca1 | Diabetic_WT/Old_WT | Stromal |
| 1.66E-14 | 0.537715482 | 0.269 | 0.188 | 3.03E-10 | Rer1 | Diabetic_WT/Old_WT | Stromal |
| 3.32E-23 | 0.537505217 | 0.399 | 0.282 | 6.06E-19 | Atp6v0e | Diabetic_WT/Old_WT | Stromal |
| 8.97E-22 | 0.536166194 | 0.388 | 0.276 | 1.63E-17 | Ybx3 | Diabetic_WT/Old_WT | Stromal |
| 2.02E-15 | 0.534869556 | 0.224 | 0.144 | 3.69E-11 | Cyba | Diabetic_WT/Old_WT | Stromal |
| 6.30E-09 | 0.534344473 | 0.171 | 0.119 | 0.000114909 | Eif1ax | Diabetic_WT/Old_WT | Stromal |
| 4.71E-60 | 0.533487177 | 0.668 | 0.463 | 8.58E-56 | Htra1 | Diabetic_WT/Old_WT | Stromal |
| 3.50E-07 | 0.52858486 | 0.137 | 0.095 | 0.006381657 | Stub1 | Diabetic_WT/Old_WT | Stromal |
| 1.04E-16 | 0.528561111 | 0.313 | 0.221 | 1.89E-12 | Rac1 | Diabetic_WT/Old_WT | Stromal |
| 1.47E-95 | 0.528442441 | 0.947 | 0.916 | 2.69E-91 | H3f3b | Diabetic_WT/Old_WT | Stromal |
| 3.29E-14 | 0.527978071 | 0.272 | 0.192 | 6.01E-10 | Tmem14c | Diabetic_WT/Old_WT | Stromal |
| 3.44E-32 | 0.527066581 | 0.601 | 0.462 | 6.28E-28 | Neat1 | Diabetic_WT/Old_WT | Stromal |
| 9.00E-08 | 0.525568886 | 0.15 | 0.105 | 0.001640748 | Ubxn1 | Diabetic_WT/Old_WT | Stromal |
| 3.35E-10 | 0.525375176 | 0.206 | 0.146 | 6.11E-06 | Hdgf | Diabetic_WT/Old_WT | Stromal |
| 1.39E-06 | 0.521090695 | 0.131 | 0.092 | 0.025392909 | Mat2a | Diabetic_WT/Old_WT | Stromal |
| 3.43E-13 | 0.52094683 | 0.249 | 0.173 | 6.26E-09 | Grn | Diabetic_WT/Old_WT | Stromal |
| 3.94E-11 | 0.520083383 | 0.211 | 0.146 | 7.19E-07 | Snu13 | Diabetic_WT/Old_WT | Stromal |
| 3.01E-07 | 0.519409397 | 0.14 | 0.098 | 0.00548305 | Glud1 | Diabetic_WT/Old_WT | Stromal |
| 9.01E-07 | 0.5176354 | 0.139 | 0.099 | 0.016432798 | Mlec | Diabetic_WT/Old_WT | Stromal |
| 1.05E-82 | 0.51600543 | 0.88 | 0.793 | 1.91E-78 | Ppic | Diabetic_WT/Old_WT | Stromal |
| 2.08E-204 | 0.515897548 | 0.998 | 0.985 | 3.79E-200 | mt-Co3 | Diabetic_WT/Old_WT | Stromal |
| 5.16E-10 | 0.514168823 | 0.199 | 0.14 | 9.41E-06 | Ikbip | Diabetic_WT/Old_WT | Stromal |
| 6.53E-12 | 0.511362803 | 0.284 | 0.211 | 1.19E-07 | Sdc2 | Diabetic_WT/Old_WT | Stromal |
| 3.07E-22 | 0.511115363 | 0.448 | 0.336 | 5.59E-18 | Atp5f1 | Diabetic_WT/Old_WT | Stromal |
| 1.40E-17 | 0.510043063 | 0.353 | 0.256 | 2.55E-13 | Rcn1 | Diabetic_WT/Old_WT | Stromal |
| 1.39E-22 | 0.507063651 | 0.448 | 0.331 | 2.53E-18 | Col18a1 | Diabetic_WT/Old_WT | Stromal |
| 1.53E-32 | 0.505690021 | 0.581 | 0.45 | 2.80E-28 | Sumo2 | Diabetic_WT/Old_WT | Stromal |
| 7.07E-20 | 0.504422246 | 0.398 | 0.292 | 1.29E-15 | Csnk1a1 | Diabetic_WT/Old_WT | Stromal |
| 5.93E-22 | 0.504369513 | 0.428 | 0.315 | 1.08E-17 | Rtraf | Diabetic_WT/Old_WT | Stromal |
| 1.13E-29 | 0.503909771 | 0.562 | 0.436 | 2.06E-25 | Map1lc3b | Diabetic_WT/Old_WT | Stromal |
| 9.26E-24 | 0.503802183 | 0.52 | 0.408 | 1.69E-19 | Manf | Diabetic_WT/Old_WT | Stromal |
| 1.46E-11 | 0.50033655 | 0.248 | 0.178 | 2.67E-07 | Map4k4 | Diabetic_WT/Old_WT | Stromal |
| 1.73E-25 | 0.498633433 | 0.669 | 0.594 | 3.16E-21 | Tuba1a | Diabetic_WT/Old_WT | Stromal |
| 7.15E-14 | 0.498450925 | 0.287 | 0.205 | 1.30E-09 | Nid1 | Diabetic_WT/Old_WT | Stromal |
| 6.16E-07 | 0.497491721 | 0.167 | 0.122 | 0.011223974 | Ptgfrn | Diabetic_WT/Old_WT | Stromal |
| 2.21E-137 | 0.496561806 | 0.965 | 0.959 | 4.04E-133 | Rpl10 | Diabetic_WT/Old_WT | Stromal |
| 1.90E-09 | 0.495922176 | 0.2 | 0.143 | 3.46E-05 | Mydgf | Diabetic_WT/Old_WT | Stromal |
| 2.19E-08 | 0.495117869 | 0.172 | 0.122 | 0.000398405 | Snhg1 | Diabetic_WT/Old_WT | Stromal |
| 8.53E-08 | 0.49502598 | 0.138 | 0.094 | 0.001554498 | Slit2 | Diabetic_WT/Old_WT | Stromal |
| 3.05E-40 | 0.493355002 | 0.653 | 0.511 | 5.56E-36 | Eif4a1 | Diabetic_WT/Old_WT | Stromal |
| 5.82E-26 | 0.492891445 | 0.587 | 0.476 | 1.06E-21 | Meg3 | Diabetic_WT/Old_WT | Stromal |
| 6.98E-83 | 0.490835364 | 0.944 | 0.878 | 1.27E-78 | Itm2b | Diabetic_WT/Old_WT | Stromal |
| 2.05E-13 | 0.488534873 | 0.29 | 0.21 | 3.73E-09 | Ndufb5 | Diabetic_WT/Old_WT | Stromal |
| 2.46E-75 | 0.487629153 | 0.938 | 0.894 | 4.49E-71 | Col5a2 | Diabetic_WT/Old_WT | Stromal |
| 1.05E-12 | 0.487144611 | 0.298 | 0.22 | 1.92E-08 | Ddx3x | Diabetic_WT/Old_WT | Stromal |
| 5.98E-16 | 0.486824364 | 0.328 | 0.236 | 1.09E-11 | Hnrnpa0 | Diabetic_WT/Old_WT | Stromal |
| 3.98E-10 | 0.484081696 | 0.233 | 0.17 | 7.26E-06 | Sri | Diabetic_WT/Old_WT | Stromal |
| 3.62E-11 | 0.483642045 | 0.163 | 0.104 | 6.59E-07 | Fosl2 | Diabetic_WT/Old_WT | Stromal |
| 4.02E-14 | 0.483106275 | 0.288 | 0.204 | 7.33E-10 | Bri3 | Diabetic_WT/Old_WT | Stromal |
| 2.88E-25 | 0.481788438 | 0.512 | 0.393 | 5.25E-21 | Arf1 | Diabetic_WT/Old_WT | Stromal |
| 1.02E-10 | 0.475877252 | 0.231 | 0.165 | 1.85E-06 | Drap1 | Diabetic_WT/Old_WT | Stromal |
| 3.01E-32 | 0.474496805 | 0.591 | 0.447 | 5.48E-28 | Cdh11 | Diabetic_WT/Old_WT | Stromal |
| 3.11E-07 | 0.47397399 | 0.155 | 0.111 | 0.005675756 | Cdk4 | Diabetic_WT/Old_WT | Stromal |
| 9.94E-13 | 0.473302202 | 0.298 | 0.22 | 1.81E-08 | Snrpb | Diabetic_WT/Old_WT | Stromal |
| 4.09E-21 | 0.472122032 | 0.542 | 0.441 | 7.46E-17 | Emp1 | Diabetic_WT/Old_WT | Stromal |
| 1.00E-09 | 0.471812851 | 0.237 | 0.175 | 1.83E-05 | Colec12 | Diabetic_WT/Old_WT | Stromal |
| 1.05E-12 | 0.469379222 | 0.333 | 0.255 | 1.91E-08 | Prrx2 | Diabetic_WT/Old_WT | Stromal |
| 3.56E-17 | 0.464241564 | 0.419 | 0.32 | 6.49E-13 | Socs3 | Diabetic_WT/Old_WT | Stromal |
| 2.32E-21 | 0.461937762 | 0.505 | 0.399 | 4.22E-17 | Clic1 | Diabetic_WT/Old_WT | Stromal |
| 2.11E-35 | 0.46012632 | 0.737 | 0.648 | 3.85E-31 | Ctsl | Diabetic_WT/Old_WT | Stromal |
| 1.58E-10 | 0.453434322 | 0.246 | 0.18 | 2.88E-06 | Pomp | Diabetic_WT/Old_WT | Stromal |
| 7.67E-07 | 0.452604707 | 0.151 | 0.109 | 0.013985272 | Kif5b | Diabetic_WT/Old_WT | Stromal |
| 1.02E-58 | 0.450343086 | 0.921 | 0.889 | 1.85E-54 | Col6a1 | Diabetic_WT/Old_WT | Stromal |
| 4.56E-10 | 0.447151947 | 0.217 | 0.154 | 8.32E-06 | Bhlhe40 | Diabetic_WT/Old_WT | Stromal |
| 5.90E-07 | 0.443027322 | 0.167 | 0.122 | 0.010760976 | Tra2b | Diabetic_WT/Old_WT | Stromal |
| 5.99E-26 | 0.442801291 | 0.471 | 0.334 | 1.09E-21 | Mfge8 | Diabetic_WT/Old_WT | Stromal |
| 3.83E-07 | 0.442699249 | 0.272 | 0.217 | 0.006976004 | Thbs4 | Diabetic_WT/Old_WT | Stromal |
| 1.33E-33 | 0.442591009 | 0.733 | 0.645 | 2.42E-29 | Calr | Diabetic_WT/Old_WT | Stromal |
| 2.78E-07 | 0.44068185 | 0.187 | 0.139 | 0.005077442 | Gng10 | Diabetic_WT/Old_WT | Stromal |
| 9.28E-19 | 0.439724929 | 0.404 | 0.295 | 1.69E-14 | Cd302 | Diabetic_WT/Old_WT | Stromal |
| 1.47E-60 | 0.439169397 | 0.99 | 0.964 | 2.68E-56 | Col1a2 | Diabetic_WT/Old_WT | Stromal |
| 3.32E-08 | 0.435151796 | 0.197 | 0.144 | 0.000605586 | Chd4 | Diabetic_WT/Old_WT | Stromal |
| 1.42E-06 | 0.430245473 | 0.172 | 0.128 | 0.025832759 | Atp6v1f | Diabetic_WT/Old_WT | Stromal |
| 3.80E-16 | 0.430146267 | 0.357 | 0.263 | 6.93E-12 | Ogn | Diabetic_WT/Old_WT | Stromal |
| 6.37E-20 | 0.428926374 | 0.489 | 0.384 | 1.16E-15 | Psmb1 | Diabetic_WT/Old_WT | Stromal |
| 8.73E-11 | 0.426352318 | 0.297 | 0.226 | 1.59E-06 | Fcgrt | Diabetic_WT/Old_WT | Stromal |
| 4.55E-61 | 0.425465433 | 0.928 | 0.887 | 8.30E-57 | Hspa8 | Diabetic_WT/Old_WT | Stromal |
| 5.43E-18 | 0.422490391 | 0.457 | 0.356 | 9.90E-14 | Arl1 | Diabetic_WT/Old_WT | Stromal |
| 1.97E-17 | 0.419537858 | 0.406 | 0.302 | 3.58E-13 | Itgb5 | Diabetic_WT/Old_WT | Stromal |
| 7.95E-56 | 0.417722118 | 0.771 | 0.626 | 1.45E-51 | Ctsk | Diabetic_WT/Old_WT | Stromal |
| 4.25E-10 | 0.417246475 | 0.271 | 0.204 | 7.76E-06 | Ifi27 | Diabetic_WT/Old_WT | Stromal |
| 4.08E-10 | 0.413669572 | 0.268 | 0.201 | 7.43E-06 | Prkar1a | Diabetic_WT/Old_WT | Stromal |
| 9.06E-76 | 0.404914409 | 0.923 | 0.901 | 1.65E-71 | Oaz1 | Diabetic_WT/Old_WT | Stromal |
| 1.08E-09 | 0.402400301 | 0.315 | 0.248 | 1.97E-05 | Abracl | Diabetic_WT/Old_WT | Stromal |
| 5.09E-09 | 0.398302683 | 0.282 | 0.219 | 9.29E-05 | Cav1 | Diabetic_WT/Old_WT | Stromal |
| 1.33E-18 | 0.398202185 | 0.547 | 0.455 | 2.43E-14 | Mtch1 | Diabetic_WT/Old_WT | Stromal |
| 3.16E-20 | 0.397149217 | 0.531 | 0.422 | 5.75E-16 | Lamp1 | Diabetic_WT/Old_WT | Stromal |
| 3.91E-27 | 0.396241943 | 0.421 | 0.281 | 7.13E-23 | Gadd45b | Diabetic_WT/Old_WT | Stromal |
| 4.30E-12 | 0.394494968 | 0.636 | 0.58 | 7.84E-08 | Ier3 | Diabetic_WT/Old_WT | Stromal |
| 5.85E-17 | 0.392151226 | 0.378 | 0.275 | 1.07E-12 | Nfkbiz | Diabetic_WT/Old_WT | Stromal |
| 1.29E-44 | 0.39177814 | 0.763 | 0.577 | 2.35E-40 | mt-Nd3 | Diabetic_WT/Old_WT | Stromal |
| 6.25E-11 | 0.390291569 | 0.343 | 0.268 | 1.14E-06 | Ddost | Diabetic_WT/Old_WT | Stromal |
| 1.81E-39 | 0.387995732 | 0.833 | 0.742 | 3.31E-35 | Jund | Diabetic_WT/Old_WT | Stromal |
| 5.90E-111 | 0.387153111 | 0.976 | 0.969 | 1.08E-106 | Eif1 | Diabetic_WT/Old_WT | Stromal |
| 5.31E-21 | 0.385787278 | 0.221 | 0.129 | 9.68E-17 | Mmp13 | Diabetic_WT/Old_WT | Stromal |
| 4.25E-09 | 0.385712891 | 0.297 | 0.233 | 7.74E-05 | Capns1 | Diabetic_WT/Old_WT | Stromal |
| 1.18E-11 | 0.385443651 | 0.426 | 0.351 | 2.15E-07 | Prdx4 | Diabetic_WT/Old_WT | Stromal |
| 1.96E-31 | 0.385109928 | 0.698 | 0.563 | 3.58E-27 | Ctsb | Diabetic_WT/Old_WT | Stromal |
| 1.15E-09 | 0.38405356 | 0.297 | 0.229 | 2.09E-05 | Ctsd | Diabetic_WT/Old_WT | Stromal |
| 6.32E-11 | 0.378453823 | 0.376 | 0.302 | 1.15E-06 | Txndc5 | Diabetic_WT/Old_WT | Stromal |
| 5.97E-12 | 0.371862799 | 0.514 | 0.453 | 1.09E-07 | Pdia6 | Diabetic_WT/Old_WT | Stromal |
| 1.51E-64 | 0.371399024 | 0.908 | 0.88 | 2.75E-60 | Laptm4a | Diabetic_WT/Old_WT | Stromal |
| 5.50E-11 | 0.369502256 | 0.34 | 0.265 | 1.00E-06 | Atp6v1g1 | Diabetic_WT/Old_WT | Stromal |
| 1.70E-08 | 0.369207682 | 0.376 | 0.314 | 0.000310725 | Col8a1 | Diabetic_WT/Old_WT | Stromal |
| 2.13E-07 | 0.369087077 | 0.259 | 0.205 | 0.003877085 | Psma2 | Diabetic_WT/Old_WT | Stromal |
| 3.33E-41 | 0.368758407 | 0.815 | 0.787 | 6.07E-37 | Selenof | Diabetic_WT/Old_WT | Stromal |
| 3.77E-53 | 0.367154731 | 0.834 | 0.672 | 6.87E-49 | Cst3 | Diabetic_WT/Old_WT | Stromal |
| 1.34E-65 | 0.362871063 | 0.952 | 0.937 | 2.44E-61 | Rpl3 | Diabetic_WT/Old_WT | Stromal |
| 1.41E-14 | 0.362530937 | 0.392 | 0.297 | 2.57E-10 | Map1lc3a | Diabetic_WT/Old_WT | Stromal |
| 2.68E-32 | 0.361397837 | 0.812 | 0.72 | 4.89E-28 | S100a10 | Diabetic_WT/Old_WT | Stromal |
| 3.54E-07 | 0.356213154 | 0.265 | 0.211 | 0.006455718 | Ccn4 | Diabetic_WT/Old_WT | Stromal |
| 2.79E-65 | 0.354228502 | 0.967 | 0.958 | 5.08E-61 | Cd63 | Diabetic_WT/Old_WT | Stromal |
| 1.66E-12 | 0.353292653 | 0.454 | 0.372 | 3.03E-08 | Olfml2b | Diabetic_WT/Old_WT | Stromal |
| 8.06E-07 | 0.352538055 | 0.208 | 0.158 | 0.014695344 | Sat1 | Diabetic_WT/Old_WT | Stromal |
| 6.09E-82 | 0.352524079 | 0.989 | 0.983 | 1.11E-77 | Rplp0 | Diabetic_WT/Old_WT | Stromal |
| 8.08E-12 | 0.352399719 | 0.411 | 0.33 | 1.47E-07 | Pnrc1 | Diabetic_WT/Old_WT | Stromal |
| 1.50E-26 | 0.349434097 | 0.856 | 0.801 | 2.73E-22 | Postn | Diabetic_WT/Old_WT | Stromal |
| 5.71E-19 | 0.349327877 | 0.567 | 0.462 | 1.04E-14 | Pebp1 | Diabetic_WT/Old_WT | Stromal |
| 6.95E-10 | 0.344449575 | 0.35 | 0.278 | 1.27E-05 | Eif3k | Diabetic_WT/Old_WT | Stromal |
| 4.15E-26 | 0.34420207 | 0.72 | 0.592 | 7.56E-22 | Cd9 | Diabetic_WT/Old_WT | Stromal |
| 9.19E-09 | 0.343831579 | 0.347 | 0.282 | 0.000167474 | Psmb4 | Diabetic_WT/Old_WT | Stromal |
| 2.08E-06 | 0.343825644 | 0.247 | 0.197 | 0.037980636 | Tmem50a | Diabetic_WT/Old_WT | Stromal |
| 1.35E-22 | 0.342153051 | 0.75 | 0.667 | 2.46E-18 | Nupr1 | Diabetic_WT/Old_WT | Stromal |
| 2.64E-14 | 0.336357334 | 0.616 | 0.539 | 4.81E-10 | Id3 | Diabetic_WT/Old_WT | Stromal |
| 1.03E-105 | 0.331538862 | 0.996 | 0.997 | 1.87E-101 | Eef1a1 | Diabetic_WT/Old_WT | Stromal |
| 4.18E-29 | 0.330822853 | 0.823 | 0.799 | 7.62E-25 | Eef2 | Diabetic_WT/Old_WT | Stromal |
| 3.88E-08 | 0.330014706 | 0.377 | 0.316 | 0.000707121 | Wls | Diabetic_WT/Old_WT | Stromal |
| 3.88E-07 | 0.329010707 | 0.303 | 0.247 | 0.007078477 | Eif3e | Diabetic_WT/Old_WT | Stromal |
| 1.29E-17 | 0.327110211 | 0.621 | 0.542 | 2.34E-13 | Slc25a3 | Diabetic_WT/Old_WT | Stromal |
| 4.93E-18 | 0.323819266 | 0.729 | 0.669 | 8.99E-14 | Hspa5 | Diabetic_WT/Old_WT | Stromal |
| 1.09E-12 | 0.317846993 | 0.828 | 0.806 | 1.99E-08 | Ybx1 | Diabetic_WT/Old_WT | Stromal |
| 5.19E-08 | 0.317524032 | 0.322 | 0.259 | 0.000945487 | Anp32b | Diabetic_WT/Old_WT | Stromal |
| 9.15E-14 | 0.312523174 | 0.536 | 0.456 | 1.67E-09 | Cdc42 | Diabetic_WT/Old_WT | Stromal |
| 6.09E-16 | 0.308177482 | 0.776 | 0.754 | 1.11E-11 | Anxa1 | Diabetic_WT/Old_WT | Stromal |
| 1.74E-06 | 0.307200646 | 0.318 | 0.264 | 0.031801094 | Lhfp | Diabetic_WT/Old_WT | Stromal |
| 3.28E-10 | 0.306613073 | 0.381 | 0.302 | 5.98E-06 | Cyb5a | Diabetic_WT/Old_WT | Stromal |
| 1.80E-29 | 0.306450665 | 0.87 | 0.835 | 3.28E-25 | Col6a2 | Diabetic_WT/Old_WT | Stromal |
| 7.48E-08 | 0.304541554 | 0.289 | 0.228 | 0.00136439 | Iqgap1 | Diabetic_WT/Old_WT | Stromal |
| 6.72E-30 | 0.30451636 | 0.849 | 0.821 | 1.22E-25 | Btf3 | Diabetic_WT/Old_WT | Stromal |
| 4.37E-09 | 0.303663379 | 0.357 | 0.286 | 7.97E-05 | Adamts2 | Diabetic_WT/Old_WT | Stromal |
| 1.45E-07 | 0.301585792 | 0.389 | 0.331 | 0.002636772 | Sh3glb1 | Diabetic_WT/Old_WT | Stromal |
| 9.78E-07 | 0.301396772 | 0.327 | 0.272 | 0.017831914 | Fkbp9 | Diabetic_WT/Old_WT | Stromal |
| 8.18E-26 | 0.299824436 | 0.777 | 0.737 | 1.49E-21 | Bsg | Diabetic_WT/Old_WT | Stromal |
| 4.88E-93 | 0.298894375 | 0.998 | 0.985 | 8.90E-89 | mt-Co1 | Diabetic_WT/Old_WT | Stromal |
| 2.00E-34 | 0.29675591 | 0.882 | 0.851 | 3.65E-30 | Gabarap | Diabetic_WT/Old_WT | Stromal |
| 1.09E-11 | 0.295654695 | 0.527 | 0.452 | 1.99E-07 | Pabpc1 | Diabetic_WT/Old_WT | Stromal |
| 1.59E-22 | 0.292975821 | 0.73 | 0.59 | 2.89E-18 | Nfkbia | Diabetic_WT/Old_WT | Stromal |
| 2.26E-11 | 0.292456913 | 0.528 | 0.456 | 4.12E-07 | Tmed2 | Diabetic_WT/Old_WT | Stromal |
| 9.28E-18 | 0.290152318 | 0.714 | 0.677 | 1.69E-13 | Arf4 | Diabetic_WT/Old_WT | Stromal |
| 1.70E-54 | 0.289981294 | 0.972 | 0.958 | 3.09E-50 | Rps3 | Diabetic_WT/Old_WT | Stromal |
| 1.52E-09 | 0.2898504 | 0.458 | 0.39 | 2.77E-05 | Akr1a1 | Diabetic_WT/Old_WT | Stromal |
| 1.07E-16 | 0.286904287 | 0.709 | 0.649 | 1.95E-12 | Cfl1 | Diabetic_WT/Old_WT | Stromal |
| 2.67E-89 | 0.286426841 | 0.998 | 0.986 | 4.86E-85 | mt-Co2 | Diabetic_WT/Old_WT | Stromal |
| 5.91E-34 | 0.283293196 | 0.935 | 0.913 | 1.08E-29 | Ppib | Diabetic_WT/Old_WT | Stromal |
| 7.27E-48 | 0.28092647 | 0.984 | 0.878 | 1.33E-43 | mt-Nd1 | Diabetic_WT/Old_WT | Stromal |
| 2.64E-13 | 0.276277105 | 0.616 | 0.542 | 4.81E-09 | Npc2 | Diabetic_WT/Old_WT | Stromal |
| 7.60E-30 | 0.273660239 | 0.976 | 0.966 | 1.39E-25 | Ptma | Diabetic_WT/Old_WT | Stromal |
| 3.09E-16 | 0.273349489 | 0.927 | 0.937 | 5.64E-12 | Igfbp7 | Diabetic_WT/Old_WT | Stromal |
| 2.25E-10 | 0.270334331 | 0.858 | 0.861 | 4.09E-06 | Tmsb4x | Diabetic_WT/Old_WT | Stromal |
| 8.91E-08 | 0.269345261 | 0.301 | 0.238 | 0.001623823 | Csrp1 | Diabetic_WT/Old_WT | Stromal |
| 8.59E-30 | 0.268587017 | 0.974 | 0.949 | 1.57E-25 | Col1a1 | Diabetic_WT/Old_WT | Stromal |
| 1.75E-10 | 0.267326657 | 0.149 | 0.094 | 3.18E-06 | Slc6a6 | Diabetic_WT/Old_WT | Stromal |
| 6.44E-07 | 0.262409299 | 0.489 | 0.433 | 0.011749652 | Col5a3 | Diabetic_WT/Old_WT | Stromal |
| 5.26E-48 | 0.254781931 | 0.984 | 0.946 | 9.59E-44 | mt-Nd4 | Diabetic_WT/Old_WT | Stromal |
| 1.82E-08 | 0.252498039 | 0.502 | 0.439 | 0.000332186 | Serbp1 | Diabetic_WT/Old_WT | Stromal |
| 1.68E-23 | 0.252136619 | 0.992 | 0.992 | 3.06E-19 | Vim | Diabetic_WT/Old_WT | Stromal |
| 1.78E-07 | 0.250573568 | 0.52 | 0.467 | 0.003241861 | Hmgn1 | Diabetic_WT/Old_WT | Stromal |
| 1.32E-16 | 0.246063188 | 0.803 | 0.759 | 2.40E-12 | Cd81 | Diabetic_WT/Old_WT | Stromal |
| 7.68E-07 | 0.242102592 | 0.449 | 0.392 | 0.014000373 | Rab2a | Diabetic_WT/Old_WT | Stromal |
| 8.53E-44 | 0.240936559 | 0.981 | 0.975 | 1.56E-39 | Rpl8 | Diabetic_WT/Old_WT | Stromal |
| 2.36E-06 | 0.236752222 | 0.432 | 0.378 | 0.043038305 | Atp5a1 | Diabetic_WT/Old_WT | Stromal |
| 1.05E-09 | 0.235010926 | 0.501 | 0.422 | 1.92E-05 | Psap | Diabetic_WT/Old_WT | Stromal |
| 1.88E-12 | 0.233377643 | 0.56 | 0.442 | 3.42E-08 | H2afz | Diabetic_WT/Old_WT | Stromal |
| 1.32E-09 | 0.231432851 | 0.595 | 0.53 | 2.41E-05 | Eef1g | Diabetic_WT/Old_WT | Stromal |
| 5.74E-09 | 0.229880997 | 0.619 | 0.572 | 0.000104671 | Gnai2 | Diabetic_WT/Old_WT | Stromal |
| 1.61E-28 | 0.22854572 | 0.96 | 0.945 | 2.94E-24 | Hsp90ab1 | Diabetic_WT/Old_WT | Stromal |
| 1.88E-06 | 0.225750201 | 0.492 | 0.439 | 0.034341513 | Ccdc80 | Diabetic_WT/Old_WT | Stromal |
| 8.70E-32 | 0.223630316 | 0.954 | 0.965 | 1.59E-27 | Gnas | Diabetic_WT/Old_WT | Stromal |
| 6.43E-23 | 0.221274222 | 0.925 | 0.899 | 1.17E-18 | Eef1b2 | Diabetic_WT/Old_WT | Stromal |
| 1.68E-07 | 0.221178667 | 0.498 | 0.436 | 0.003071608 | Rabac1 | Diabetic_WT/Old_WT | Stromal |
| 2.65E-20 | 0.217185189 | 0.881 | 0.854 | 4.84E-16 | Rpl5 | Diabetic_WT/Old_WT | Stromal |
| 2.21E-17 | 0.216033012 | 0.846 | 0.85 | 4.03E-13 | Serpinf1 | Diabetic_WT/Old_WT | Stromal |
| 4.77E-15 | 0.215761596 | 0.277 | 0.184 | 8.69E-11 | Wnt5a | Diabetic_WT/Old_WT | Stromal |
| 1.05E-10 | 0.215494897 | 0.702 | 0.627 | 1.91E-06 | Eif5a | Diabetic_WT/Old_WT | Stromal |
| 2.14E-18 | 0.215315857 | 0.957 | 0.951 | 3.91E-14 | Rps2 | Diabetic_WT/Old_WT | Stromal |
| 1.06E-06 | 0.214968067 | 0.587 | 0.548 | 0.01929808 | Eif3f | Diabetic_WT/Old_WT | Stromal |
| 8.58E-18 | 0.20901363 | 0.9 | 0.905 | 1.56E-13 | Naca | Diabetic_WT/Old_WT | Stromal |
| 2.14E-14 | 0.205658706 | 0.859 | 0.838 | 3.90E-10 | Timp2 | Diabetic_WT/Old_WT | Stromal |
| 2.37E-07 | 0.202955191 | 0.559 | 0.502 | 0.004315253 | App | Diabetic_WT/Old_WT | Stromal |
| 1.10E-12 | 0.198566413 | 0.804 | 0.793 | 2.00E-08 | Rpl4 | Diabetic_WT/Old_WT | Stromal |
| 1.65E-07 | 0.195212375 | 0.164 | 0.117 | 0.003015212 | Il1rl1 | Diabetic_WT/Old_WT | Stromal |
| 2.51E-08 | 0.194841441 | 0.519 | 0.454 | 0.00045786 | Nbl1 | Diabetic_WT/Old_WT | Stromal |
| 2.51E-20 | 0.188670719 | 0.997 | 0.987 | 4.58E-16 | Sparc | Diabetic_WT/Old_WT | Stromal |
| 6.73E-15 | 0.186207001 | 0.993 | 0.969 | 1.23E-10 | Col3a1 | Diabetic_WT/Old_WT | Stromal |
| 1.20E-10 | 0.174462891 | 0.876 | 0.872 | 2.18E-06 | Rps6 | Diabetic_WT/Old_WT | Stromal |
| 6.42E-26 | 0.17025577 | 0.831 | 0.731 | 1.17E-21 | Igfbp4 | Diabetic_WT/Old_WT | Stromal |
| 2.35E-16 | 0.159067595 | 0.974 | 0.966 | 4.29E-12 | Rpl15 | Diabetic_WT/Old_WT | Stromal |
| 2.10E-06 | 0.156989888 | 0.747 | 0.732 | 0.038291684 | Myl12a | Diabetic_WT/Old_WT | Stromal |
| 1.97E-08 | 0.152638583 | 0.306 | 0.243 | 0.000360062 | Fmod | Diabetic_WT/Old_WT | Stromal |
| 7.83E-10 | 0.149461644 | 0.819 | 0.824 | 1.43E-05 | Slc25a4 | Diabetic_WT/Old_WT | Stromal |
| 1.61E-06 | 0.145256319 | 0.756 | 0.75 | 0.02942516 | Calm2 | Diabetic_WT/Old_WT | Stromal |
| 8.31E-09 | 0.141736564 | 0.601 | 0.517 | 0.000151522 | Ahnak | Diabetic_WT/Old_WT | Stromal |
| 1.16E-09 | 0.127089739 | 0.176 | 0.118 | 2.11E-05 | Apoe | Diabetic_WT/Old_WT | Stromal |
| 7.16E-11 | -0.10281765 | 0.976 | 0.981 | 1.30E-06 | Rpl18a | Diabetic_WT/Old_WT | Stromal |
| 2.77E-08 | -0.121897336 | 0.944 | 0.947 | 0.000505375 | Rpl12 | Diabetic_WT/Old_WT | Stromal |
| 1.38E-12 | -0.125985841 | 0.957 | 0.968 | 2.51E-08 | Rpl10a | Diabetic_WT/Old_WT | Stromal |
| 8.97E-27 | -0.13380679 | 0.981 | 0.986 | 1.64E-22 | Rps3a1 | Diabetic_WT/Old_WT | Stromal |
| 9.05E-10 | -0.140698489 | 0.757 | 0.816 | 1.65E-05 | Nedd4 | Diabetic_WT/Old_WT | Stromal |
| 6.93E-19 | -0.140722686 | 0.99 | 0.99 | 1.26E-14 | Rplp1 | Diabetic_WT/Old_WT | Stromal |
| 8.58E-12 | -0.143404326 | 0.872 | 0.926 | 1.56E-07 | Cox4i1 | Diabetic_WT/Old_WT | Stromal |
| 1.90E-10 | -0.164875362 | 0.73 | 0.808 | 3.46E-06 | Prdx1 | Diabetic_WT/Old_WT | Stromal |
| 1.02E-25 | -0.174024749 | 0.965 | 0.977 | 1.86E-21 | Rpl24 | Diabetic_WT/Old_WT | Stromal |
| 4.25E-07 | -0.175057822 | 0.272 | 0.339 | 0.007754687 | Prss23 | Diabetic_WT/Old_WT | Stromal |
| 3.57E-08 | -0.177328215 | 0.653 | 0.716 | 0.000651152 | Emp3 | Diabetic_WT/Old_WT | Stromal |
| 6.56E-09 | -0.177474148 | 0.719 | 0.779 | 0.000119664 | Calm1 | Diabetic_WT/Old_WT | Stromal |
| 7.05E-08 | -0.179785437 | 0.765 | 0.802 | 0.001286045 | Anxa2 | Diabetic_WT/Old_WT | Stromal |
| 1.35E-06 | -0.180798757 | 0.364 | 0.424 | 0.024614584 | Mt2 | Diabetic_WT/Old_WT | Stromal |
| 2.93E-27 | -0.181867371 | 0.965 | 0.979 | 5.33E-23 | Rpl18 | Diabetic_WT/Old_WT | Stromal |
| 3.60E-10 | -0.187927103 | 0.594 | 0.674 | 6.56E-06 | Reep5 | Diabetic_WT/Old_WT | Stromal |
| 9.25E-12 | -0.205519644 | 0.643 | 0.719 | 1.69E-07 | Pdia3 | Diabetic_WT/Old_WT | Stromal |
| 3.37E-59 | -0.213860651 | 0.995 | 0.995 | 6.15E-55 | Rps29 | Diabetic_WT/Old_WT | Stromal |
| 1.56E-10 | -0.213982854 | 0.513 | 0.598 | 2.84E-06 | Nenf | Diabetic_WT/Old_WT | Stromal |
| 1.72E-06 | -0.218317313 | 0.318 | 0.378 | 0.031275599 | Ldha | Diabetic_WT/Old_WT | Stromal |
| 1.58E-07 | -0.226641262 | 0.348 | 0.415 | 0.002881268 | Vkorc1 | Diabetic_WT/Old_WT | Stromal |
| 2.80E-50 | -0.234209398 | 0.955 | 0.98 | 5.11E-46 | Rps14 | Diabetic_WT/Old_WT | Stromal |
| 4.50E-07 | -0.24808066 | 0.332 | 0.394 | 0.008197799 | Nedd8 | Diabetic_WT/Old_WT | Stromal |
| 3.04E-18 | -0.251932414 | 0.643 | 0.74 | 5.53E-14 | Ssr4 | Diabetic_WT/Old_WT | Stromal |
| 1.48E-07 | -0.252972827 | 0.39 | 0.459 | 0.00270042 | Tm4sf1 | Diabetic_WT/Old_WT | Stromal |
| 3.67E-27 | -0.254157501 | 0.932 | 0.933 | 6.68E-23 | Nme2 | Diabetic_WT/Old_WT | Stromal |
| 8.32E-17 | -0.255823133 | 0.567 | 0.677 | 1.52E-12 | Atp5h | Diabetic_WT/Old_WT | Stromal |
| 1.04E-07 | -0.264859899 | 0.439 | 0.492 | 0.001899929 | Dusp1 | Diabetic_WT/Old_WT | Stromal |
| 1.87E-61 | -0.267258578 | 0.966 | 0.978 | 3.42E-57 | Rps11 | Diabetic_WT/Old_WT | Stromal |
| 2.12E-12 | -0.276165222 | 0.472 | 0.562 | 3.86E-08 | Cavin3 | Diabetic_WT/Old_WT | Stromal |
| 2.07E-41 | -0.279030527 | 0.943 | 0.965 | 3.78E-37 | Ppia | Diabetic_WT/Old_WT | Stromal |
| 2.68E-46 | -0.279660174 | 0.92 | 0.937 | 4.89E-42 | Rpl14 | Diabetic_WT/Old_WT | Stromal |
| 2.56E-09 | -0.281632561 | 0.357 | 0.431 | 4.66E-05 | Lman1 | Diabetic_WT/Old_WT | Stromal |
| 1.38E-11 | -0.282841286 | 0.313 | 0.4 | 2.52E-07 | Vcan | Diabetic_WT/Old_WT | Stromal |
| 1.71E-08 | -0.283111325 | 0.3 | 0.368 | 0.000311516 | Ndufb9 | Diabetic_WT/Old_WT | Stromal |
| 7.43E-13 | -0.285882909 | 0.661 | 0.72 | 1.35E-08 | Prrx1 | Diabetic_WT/Old_WT | Stromal |
| 2.15E-06 | -0.286593421 | 0.236 | 0.29 | 0.039132195 | Psmb3 | Diabetic_WT/Old_WT | Stromal |
| 2.00E-23 | -0.296183077 | 0.908 | 0.951 | 3.64E-19 | Myl6 | Diabetic_WT/Old_WT | Stromal |
| 1.07E-10 | -0.299069466 | 0.328 | 0.409 | 1.96E-06 | Copz2 | Diabetic_WT/Old_WT | Stromal |
| 3.60E-86 | -0.307275653 | 0.979 | 0.984 | 6.56E-82 | Rps10 | Diabetic_WT/Old_WT | Stromal |
| 3.05E-35 | -0.311906376 | 0.902 | 0.916 | 5.57E-31 | Ifitm2 | Diabetic_WT/Old_WT | Stromal |
| 8.89E-50 | -0.312404005 | 0.992 | 0.988 | 1.62E-45 | mt-Cytb | Diabetic_WT/Old_WT | Stromal |
| 4.40E-08 | -0.319575341 | 0.236 | 0.298 | 0.000803154 | Vcp | Diabetic_WT/Old_WT | Stromal |
| 5.82E-08 | -0.323002852 | 0.248 | 0.31 | 0.001061945 | Timm13 | Diabetic_WT/Old_WT | Stromal |
| 1.21E-08 | -0.325123547 | 0.301 | 0.367 | 0.000220562 | Cnn2 | Diabetic_WT/Old_WT | Stromal |
| 3.13E-17 | -0.329426782 | 0.619 | 0.696 | 5.70E-13 | Itgb1 | Diabetic_WT/Old_WT | Stromal |
| 7.86E-80 | -0.330860587 | 0.949 | 0.974 | 1.43E-75 | Rpl17 | Diabetic_WT/Old_WT | Stromal |
| 1.26E-10 | -0.332068254 | 0.289 | 0.367 | 2.30E-06 | Ndufb11 | Diabetic_WT/Old_WT | Stromal |
| 8.13E-09 | -0.335753365 | 0.236 | 0.302 | 0.000148276 | Nap1l1 | Diabetic_WT/Old_WT | Stromal |
| 5.24E-10 | -0.339290077 | 0.253 | 0.325 | 9.55E-06 | Antxr1 | Diabetic_WT/Old_WT | Stromal |
| 6.58E-24 | -0.341926175 | 0.662 | 0.751 | 1.20E-19 | Hmgb1 | Diabetic_WT/Old_WT | Stromal |
| 3.47E-35 | -0.355937923 | 0.691 | 0.812 | 6.33E-31 | Gas5 | Diabetic_WT/Old_WT | Stromal |
| 8.86E-10 | -0.357424564 | 0.256 | 0.327 | 1.61E-05 | Nme1 | Diabetic_WT/Old_WT | Stromal |
| 2.16E-121 | -0.359029257 | 0.98 | 0.988 | 3.93E-117 | Rps16 | Diabetic_WT/Old_WT | Stromal |
| 5.47E-47 | -0.362358973 | 0.761 | 0.865 | 9.97E-43 | Selenom | Diabetic_WT/Old_WT | Stromal |
| 5.20E-22 | -0.366084015 | 0.529 | 0.628 | 9.49E-18 | Lox | Diabetic_WT/Old_WT | Stromal |
| 1.37E-13 | -0.370963026 | 0.614 | 0.696 | 2.51E-09 | Mt1 | Diabetic_WT/Old_WT | Stromal |
| 6.42E-29 | -0.372515597 | 0.584 | 0.701 | 1.17E-24 | Hint1 | Diabetic_WT/Old_WT | Stromal |
| 1.29E-17 | -0.377814658 | 0.741 | 0.805 | 2.36E-13 | Aebp1 | Diabetic_WT/Old_WT | Stromal |
| 1.15E-103 | -0.384568762 | 0.968 | 0.983 | 2.09E-99 | Rps19 | Diabetic_WT/Old_WT | Stromal |
| 1.29E-08 | -0.390169825 | 0.183 | 0.243 | 0.000235215 | Ndufb8 | Diabetic_WT/Old_WT | Stromal |
| 8.40E-08 | -0.390341346 | 0.184 | 0.239 | 0.00153193 | Ndufc2 | Diabetic_WT/Old_WT | Stromal |
| 1.61E-46 | -0.391218537 | 0.671 | 0.82 | 2.94E-42 | Selenow | Diabetic_WT/Old_WT | Stromal |
| 2.13E-06 | -0.396209205 | 0.132 | 0.176 | 0.038884279 | Pdcd5 | Diabetic_WT/Old_WT | Stromal |
| 2.37E-34 | -0.397149485 | 0.582 | 0.71 | 4.32E-30 | P4hb | Diabetic_WT/Old_WT | Stromal |
| 2.88E-13 | -0.402312397 | 0.269 | 0.355 | 5.25E-09 | Rex1bd | Diabetic_WT/Old_WT | Stromal |
| 1.49E-10 | -0.402639637 | 0.231 | 0.302 | 2.71E-06 | Smdt1 | Diabetic_WT/Old_WT | Stromal |
| 1.26E-16 | -0.403014696 | 0.363 | 0.467 | 2.30E-12 | 2410006H16Rik | Diabetic_WT/Old_WT | Stromal |
| 6.09E-09 | -0.410114139 | 0.162 | 0.22 | 0.000111093 | Fibin | Diabetic_WT/Old_WT | Stromal |
| 1.67E-96 | -0.410228563 | 0.898 | 0.951 | 3.05E-92 | Rpl27 | Diabetic_WT/Old_WT | Stromal |
| 1.69E-141 | -0.414933394 | 0.986 | 0.988 | 3.08E-137 | Rps27a | Diabetic_WT/Old_WT | Stromal |
| 1.14E-33 | -0.415705846 | 0.627 | 0.733 | 2.08E-29 | Jun | Diabetic_WT/Old_WT | Stromal |
| 3.77E-134 | -0.418059584 | 0.943 | 0.979 | 6.87E-130 | Rpl26 | Diabetic_WT/Old_WT | Stromal |
| 4.78E-09 | -0.421135545 | 0.156 | 0.214 | 8.71E-05 | Slc39a1 | Diabetic_WT/Old_WT | Stromal |
| 9.08E-16 | -0.424928397 | 0.329 | 0.423 | 1.66E-11 | Pdgfra | Diabetic_WT/Old_WT | Stromal |
| 1.90E-54 | -0.428543715 | 0.626 | 0.801 | 3.47E-50 | Uqcrh | Diabetic_WT/Old_WT | Stromal |
| 7.14E-156 | -0.43915053 | 0.977 | 0.989 | 1.30E-151 | Rpl27a | Diabetic_WT/Old_WT | Stromal |
| 9.49E-139 | -0.440398757 | 0.961 | 0.983 | 1.73E-134 | Rpl11 | Diabetic_WT/Old_WT | Stromal |
| 1.11E-129 | -0.443409149 | 0.984 | 0.986 | 2.03E-125 | Rps12 | Diabetic_WT/Old_WT | Stromal |
| 2.52E-07 | -0.447313732 | 0.15 | 0.199 | 0.004593562 | Pam | Diabetic_WT/Old_WT | Stromal |
| 2.39E-45 | -0.451692172 | 0.531 | 0.704 | 4.36E-41 | Gng5 | Diabetic_WT/Old_WT | Stromal |
| 3.73E-09 | -0.454035023 | 0.206 | 0.268 | 6.80E-05 | Cavin1 | Diabetic_WT/Old_WT | Stromal |
| 1.21E-167 | -0.457117236 | 0.987 | 0.993 | 2.21E-163 | Rpl23 | Diabetic_WT/Old_WT | Stromal |
| 2.83E-24 | -0.459900028 | 0.385 | 0.509 | 5.15E-20 | Sh3bgrl3 | Diabetic_WT/Old_WT | Stromal |
| 5.22E-154 | -0.465716092 | 0.947 | 0.976 | 9.52E-150 | Rps13 | Diabetic_WT/Old_WT | Stromal |
| 2.45E-13 | -0.469044489 | 0.193 | 0.273 | 4.47E-09 | Cope | Diabetic_WT/Old_WT | Stromal |
| 7.36E-13 | -0.470451241 | 0.243 | 0.322 | 1.34E-08 | Rnase4 | Diabetic_WT/Old_WT | Stromal |
| 1.27E-16 | -0.4741917 | 0.389 | 0.483 | 2.32E-12 | Zfp36 | Diabetic_WT/Old_WT | Stromal |
| 2.29E-11 | -0.48642504 | 0.158 | 0.225 | 4.18E-07 | Pdap1 | Diabetic_WT/Old_WT | Stromal |
| 9.17E-87 | -0.495239611 | 0.814 | 0.888 | 1.67E-82 | Rpl23a | Diabetic_WT/Old_WT | Stromal |
| 2.50E-27 | -0.497975062 | 0.372 | 0.502 | 4.55E-23 | Hnrnpa2b1 | Diabetic_WT/Old_WT | Stromal |
| 6.17E-10 | -0.498608231 | 0.148 | 0.207 | 1.13E-05 | Copb2 | Diabetic_WT/Old_WT | Stromal |
| 1.84E-07 | -0.503070829 | 0.149 | 0.197 | 0.003359289 | Prelp | Diabetic_WT/Old_WT | Stromal |
| 3.05E-61 | -0.506014724 | 0.535 | 0.734 | 5.56E-57 | Morf4l1 | Diabetic_WT/Old_WT | Stromal |
| 1.79E-55 | -0.509042949 | 0.758 | 0.855 | 3.26E-51 | Rps18 | Diabetic_WT/Old_WT | Stromal |
| 3.21E-09 | -0.511528037 | 0.106 | 0.157 | 5.85E-05 | Selenoh | Diabetic_WT/Old_WT | Stromal |
| 1.09E-11 | -0.517989926 | 0.151 | 0.218 | 1.99E-07 | Rwdd1 | Diabetic_WT/Old_WT | Stromal |
| 1.46E-12 | -0.520086836 | 0.17 | 0.243 | 2.67E-08 | Eny2 | Diabetic_WT/Old_WT | Stromal |
| 1.32E-06 | -0.522233167 | 0.111 | 0.151 | 0.024106717 | C1ra | Diabetic_WT/Old_WT | Stromal |
| 7.79E-17 | -0.522270339 | 0.229 | 0.322 | 1.42E-12 | Fermt2 | Diabetic_WT/Old_WT | Stromal |
| 6.07E-33 | -0.526849991 | 0.327 | 0.477 | 1.11E-28 | Tmem167 | Diabetic_WT/Old_WT | Stromal |
| 3.79E-58 | -0.533038663 | 0.466 | 0.674 | 6.91E-54 | Atp5j | Diabetic_WT/Old_WT | Stromal |
| 1.99E-195 | -0.535043323 | 0.954 | 0.979 | 3.63E-191 | Rpl21 | Diabetic_WT/Old_WT | Stromal |
| 4.13E-15 | -0.53649893 | 0.18 | 0.263 | 7.54E-11 | Tbca | Diabetic_WT/Old_WT | Stromal |
| 2.03E-231 | -0.538030628 | 0.988 | 0.993 | 3.70E-227 | Rps28 | Diabetic_WT/Old_WT | Stromal |
| 1.93E-06 | -0.540584071 | 0.073 | 0.108 | 0.035179798 | Denr | Diabetic_WT/Old_WT | Stromal |
| 2.00E-06 | -0.541383833 | 0.079 | 0.114 | 0.036398821 | Ndufs6 | Diabetic_WT/Old_WT | Stromal |
| 3.33E-80 | -0.547222255 | 0.807 | 0.864 | 6.08E-76 | S100a11 | Diabetic_WT/Old_WT | Stromal |
| 4.48E-21 | -0.553120628 | 0.348 | 0.452 | 8.17E-17 | Gas1 | Diabetic_WT/Old_WT | Stromal |
| 1.27E-86 | -0.554353272 | 0.946 | 0.985 | 2.32E-82 | Actb | Diabetic_WT/Old_WT | Stromal |
| 1.58E-06 | -0.556412432 | 0.083 | 0.12 | 0.028726593 | Cycs | Diabetic_WT/Old_WT | Stromal |
| 3.87E-221 | -0.556423556 | 0.973 | 0.988 | 7.06E-217 | Rpl28 | Diabetic_WT/Old_WT | Stromal |
| 2.25E-12 | -0.564278853 | 0.179 | 0.249 | 4.11E-08 | Nfib | Diabetic_WT/Old_WT | Stromal |
| 4.01E-237 | -0.567552324 | 0.972 | 0.987 | 7.31E-233 | Rps27 | Diabetic_WT/Old_WT | Stromal |
| 1.13E-40 | -0.577279193 | 0.35 | 0.52 | 2.05E-36 | Atp5k | Diabetic_WT/Old_WT | Stromal |
| 6.95E-08 | -0.578848903 | 0.084 | 0.126 | 0.001267226 | Hivep2 | Diabetic_WT/Old_WT | Stromal |
| 2.43E-197 | -0.581338794 | 0.963 | 0.983 | 4.44E-193 | Rpl32 | Diabetic_WT/Old_WT | Stromal |
| 4.85E-44 | -0.584021116 | 0.386 | 0.559 | 8.84E-40 | Atp5g1 | Diabetic_WT/Old_WT | Stromal |
| 2.51E-14 | -0.589766042 | 0.163 | 0.239 | 4.58E-10 | Rsrp1 | Diabetic_WT/Old_WT | Stromal |
| 2.89E-24 | -0.599877422 | 0.225 | 0.341 | 5.27E-20 | Tma7 | Diabetic_WT/Old_WT | Stromal |
| 8.22E-110 | -0.60004664 | 0.87 | 0.944 | 1.50E-105 | Ifitm3 | Diabetic_WT/Old_WT | Stromal |
| 3.49E-07 | -0.602095247 | 0.065 | 0.101 | 0.006371216 | Mrpl54 | Diabetic_WT/Old_WT | Stromal |
| 5.19E-277 | -0.612553629 | 0.979 | 0.994 | 9.46E-273 | Fau | Diabetic_WT/Old_WT | Stromal |
| 1.08E-52 | -0.619992452 | 0.358 | 0.555 | 1.96E-48 | Micos10 | Diabetic_WT/Old_WT | Stromal |
| 1.33E-19 | -0.622981149 | 0.173 | 0.269 | 2.43E-15 | Tbrg1 | Diabetic_WT/Old_WT | Stromal |
| 3.46E-45 | -0.626580215 | 0.366 | 0.542 | 6.30E-41 | Serpinb6a | Diabetic_WT/Old_WT | Stromal |
| 6.30E-07 | -0.626580215 | 0.066 | 0.1 | 0.011486443 | Ndufb6 | Diabetic_WT/Old_WT | Stromal |
| 3.29E-20 | -0.627752607 | 0.184 | 0.282 | 6.00E-16 | Hnrnpa3 | Diabetic_WT/Old_WT | Stromal |
| 4.85E-100 | -0.631082287 | 0.676 | 0.858 | 8.84E-96 | Cox8a | Diabetic_WT/Old_WT | Stromal |
| 8.78E-84 | -0.631539025 | 0.543 | 0.753 | 1.60E-79 | Sem1 | Diabetic_WT/Old_WT | Stromal |
| 1.35E-14 | -0.631567221 | 0.132 | 0.204 | 2.47E-10 | Ndufa6 | Diabetic_WT/Old_WT | Stromal |
| 6.18E-11 | -0.632089987 | 0.102 | 0.157 | 1.13E-06 | Srp19 | Diabetic_WT/Old_WT | Stromal |
| 2.81E-18 | -0.637422374 | 0.165 | 0.255 | 5.12E-14 | Grcc10 | Diabetic_WT/Old_WT | Stromal |
| 0 | -0.643995679 | 0.989 | 0.995 | 0 | Rps24 | Diabetic_WT/Old_WT | Stromal |
| 4.14E-14 | -0.649076615 | 0.121 | 0.191 | 7.54E-10 | Ndufs5 | Diabetic_WT/Old_WT | Stromal |
| 1.01E-17 | -0.649838812 | 0.149 | 0.235 | 1.84E-13 | Eloc | Diabetic_WT/Old_WT | Stromal |
| 1.87E-20 | -0.654496945 | 0.17 | 0.267 | 3.41E-16 | Ier3ip1 | Diabetic_WT/Old_WT | Stromal |
| 1.91E-08 | -0.657236801 | 0.076 | 0.118 | 0.000347365 | Mgst1 | Diabetic_WT/Old_WT | Stromal |
| 1.62E-09 | -0.657883025 | 0.082 | 0.129 | 2.95E-05 | Fgf7 | Diabetic_WT/Old_WT | Stromal |
| 4.36E-16 | -0.659243611 | 0.135 | 0.213 | 7.95E-12 | Gtf2h5 | Diabetic_WT/Old_WT | Stromal |
| 5.73E-34 | -0.660075771 | 0.318 | 0.459 | 1.04E-29 | Nfix | Diabetic_WT/Old_WT | Stromal |
| 5.32E-07 | -0.66385427 | 0.121 | 0.164 | 0.009699707 | Cfh | Diabetic_WT/Old_WT | Stromal |
| 5.81E-296 | -0.672056736 | 0.965 | 0.985 | 1.06E-291 | Rps23 | Diabetic_WT/Old_WT | Stromal |
| 0 | -0.6763387 | 0.996 | 0.996 | 0 | Rps8 | Diabetic_WT/Old_WT | Stromal |
| 1.03E-06 | -0.683174016 | 0.101 | 0.139 | 0.018844654 | Rrad | Diabetic_WT/Old_WT | Stromal |
| 2.95E-262 | -0.686319393 | 0.988 | 0.992 | 5.38E-258 | Rps20 | Diabetic_WT/Old_WT | Stromal |
| 4.63E-61 | -0.687000648 | 0.418 | 0.612 | 8.44E-57 | Fbln2 | Diabetic_WT/Old_WT | Stromal |
| 3.22E-14 | -0.693456989 | 0.12 | 0.189 | 5.87E-10 | Nucks1 | Diabetic_WT/Old_WT | Stromal |
| 2.16E-94 | -0.696022917 | 0.684 | 0.814 | 3.94E-90 | Rrbp1 | Diabetic_WT/Old_WT | Stromal |
| 1.10E-73 | -0.701630921 | 0.38 | 0.612 | 2.00E-69 | Sec62 | Diabetic_WT/Old_WT | Stromal |
| 3.84E-09 | -0.701630921 | 0.062 | 0.103 | 7.00E-05 | Bmpr2 | Diabetic_WT/Old_WT | Stromal |
| 5.66E-08 | -0.701630921 | 0.077 | 0.118 | 0.001031522 | Dleu2 | Diabetic_WT/Old_WT | Stromal |
| 1.22E-57 | -0.704608445 | 0.461 | 0.658 | 2.22E-53 | S100a4 | Diabetic_WT/Old_WT | Stromal |
| 5.10E-201 | -0.705681974 | 0.841 | 0.953 | 9.31E-197 | Rps15 | Diabetic_WT/Old_WT | Stromal |
| 1.06E-88 | -0.711206923 | 0.636 | 0.773 | 1.94E-84 | Sec61b | Diabetic_WT/Old_WT | Stromal |
| 3.63E-09 | -0.712981845 | 0.064 | 0.106 | 6.62E-05 | Mrps16 | Diabetic_WT/Old_WT | Stromal |
| 4.82E-232 | -0.713468935 | 0.923 | 0.969 | 8.79E-228 | Serf2 | Diabetic_WT/Old_WT | Stromal |
| 1.64E-101 | -0.719267565 | 0.468 | 0.742 | 2.99E-97 | Fos | Diabetic_WT/Old_WT | Stromal |
| 4.12E-25 | -0.722525257 | 0.4 | 0.505 | 7.51E-21 | Rbp1 | Diabetic_WT/Old_WT | Stromal |
| 1.52E-25 | -0.732946461 | 0.19 | 0.302 | 2.78E-21 | Fkbp11 | Diabetic_WT/Old_WT | Stromal |
| 0 | -0.737827016 | 0.972 | 0.992 | 0 | Rpl38 | Diabetic_WT/Old_WT | Stromal |
| 1.15E-295 | -0.740801518 | 0.92 | 0.975 | 2.09E-291 | Rplp2 | Diabetic_WT/Old_WT | Stromal |
| 1.10E-09 | -0.742658189 | 0.061 | 0.103 | 2.01E-05 | Hp1bp3 | Diabetic_WT/Old_WT | Stromal |
| 4.84E-38 | -0.745179701 | 0.21 | 0.358 | 8.82E-34 | Rbx1 | Diabetic_WT/Old_WT | Stromal |
| 1.07E-28 | -0.74652027 | 0.154 | 0.27 | 1.95E-24 | Pdgfrl | Diabetic_WT/Old_WT | Stromal |
| 2.01E-88 | -0.747602629 | 0.412 | 0.657 | 3.67E-84 | Krtcap2 | Diabetic_WT/Old_WT | Stromal |
| 8.06E-14 | -0.755970755 | 0.099 | 0.162 | 1.47E-09 | Snhg8 | Diabetic_WT/Old_WT | Stromal |
| 2.57E-11 | -0.757340353 | 0.078 | 0.129 | 4.68E-07 | Mrpl23 | Diabetic_WT/Old_WT | Stromal |
| 1.21E-204 | -0.75815065 | 0.787 | 0.927 | 2.21E-200 | Rps25 | Diabetic_WT/Old_WT | Stromal |
| 1.20E-08 | -0.761603337 | 0.198 | 0.255 | 0.000219644 | Angptl1 | Diabetic_WT/Old_WT | Stromal |
| 2.18E-176 | -0.765542684 | 0.738 | 0.874 | 3.98E-172 | Rpl22l1 | Diabetic_WT/Old_WT | Stromal |
| 1.20E-31 | -0.770126143 | 0.263 | 0.397 | 2.19E-27 | Ebf1 | Diabetic_WT/Old_WT | Stromal |
| 1.32E-26 | -0.770343671 | 0.167 | 0.279 | 2.41E-22 | Il6st | Diabetic_WT/Old_WT | Stromal |
| 4.42E-11 | -0.774387263 | 0.069 | 0.117 | 8.06E-07 | S100a16 | Diabetic_WT/Old_WT | Stromal |
| 1.34E-29 | -0.78684131 | 0.187 | 0.308 | 2.45E-25 | Hif1a | Diabetic_WT/Old_WT | Stromal |
| 3.79E-13 | -0.79060455 | 0.11 | 0.173 | 6.92E-09 | Mbnl1 | Diabetic_WT/Old_WT | Stromal |
| 3.04E-13 | -0.793147067 | 0.13 | 0.196 | 5.55E-09 | Cd248 | Diabetic_WT/Old_WT | Stromal |
| 2.88E-60 | -0.793398907 | 0.29 | 0.494 | 5.25E-56 | Dbi | Diabetic_WT/Old_WT | Stromal |
| 4.60E-137 | -0.795897891 | 0.487 | 0.784 | 8.39E-133 | Atp5l | Diabetic_WT/Old_WT | Stromal |
| 1.49E-19 | -0.797891462 | 0.175 | 0.265 | 2.71E-15 | Serpine1 | Diabetic_WT/Old_WT | Stromal |
| 3.60E-70 | -0.809604692 | 0.307 | 0.527 | 6.56E-66 | Ndufb1-ps | Diabetic_WT/Old_WT | Stromal |
| 2.62E-13 | -0.821081899 | 0.076 | 0.132 | 4.77E-09 | Ndufa12 | Diabetic_WT/Old_WT | Stromal |
| 2.99E-11 | -0.822485316 | 0.061 | 0.108 | 5.45E-07 | Higd1a | Diabetic_WT/Old_WT | Stromal |
| 1.75E-15 | -0.82670993 | 0.202 | 0.28 | 3.19E-11 | Gng11 | Diabetic_WT/Old_WT | Stromal |
| 3.05E-19 | -0.835962988 | 0.104 | 0.183 | 5.56E-15 | Hcfc1r1 | Diabetic_WT/Old_WT | Stromal |
| 2.50E-218 | -0.836925035 | 0.958 | 0.977 | 4.56E-214 | Lgals1 | Diabetic_WT/Old_WT | Stromal |
| 6.86E-49 | -0.838832909 | 0.218 | 0.389 | 1.25E-44 | Snrpd2 | Diabetic_WT/Old_WT | Stromal |
| 9.83E-13 | -0.844887972 | 0.089 | 0.147 | 1.79E-08 | Filip1l | Diabetic_WT/Old_WT | Stromal |
| 0 | -0.85194977 | 0.93 | 0.982 | 0 | Rpl30 | Diabetic_WT/Old_WT | Stromal |
| 0 | -0.855911199 | 0.96 | 0.985 | 0 | Rps15a | Diabetic_WT/Old_WT | Stromal |
| 1.69E-14 | -0.873575429 | 0.149 | 0.22 | 3.08E-10 | Atf3 | Diabetic_WT/Old_WT | Stromal |
| 0 | -0.875263585 | 0.992 | 0.997 | 0 | Rpl41 | Diabetic_WT/Old_WT | Stromal |
| 4.58E-12 | -0.875980173 | 0.063 | 0.112 | 8.36E-08 | Sap18 | Diabetic_WT/Old_WT | Stromal |
| 0 | -0.884354157 | 0.825 | 0.949 | 0 | Rpl22 | Diabetic_WT/Old_WT | Stromal |
| 9.21E-47 | -0.894922223 | 0.217 | 0.38 | 1.68E-42 | Son | Diabetic_WT/Old_WT | Stromal |
| 9.72E-65 | -0.896141256 | 0.495 | 0.653 | 1.77E-60 | Serping1 | Diabetic_WT/Old_WT | Stromal |
| 1.78E-108 | -0.900598996 | 0.391 | 0.649 | 3.24E-104 | Ndufa4 | Diabetic_WT/Old_WT | Stromal |
| 1.82E-14 | -0.901400433 | 0.11 | 0.176 | 3.32E-10 | Dpt | Diabetic_WT/Old_WT | Stromal |
| 6.48E-15 | -0.910750985 | 0.086 | 0.149 | 1.18E-10 | Irf1 | Diabetic_WT/Old_WT | Stromal |
| 2.94E-17 | -0.91275399 | 0.106 | 0.18 | 5.37E-13 | Col14a1 | Diabetic_WT/Old_WT | Stromal |
| 0 | -0.915444834 | 0.959 | 0.993 | 0 | Rpl36 | Diabetic_WT/Old_WT | Stromal |
| 0 | -0.919378245 | 0.985 | 0.995 | 0 | Rps21 | Diabetic_WT/Old_WT | Stromal |
| 3.13E-08 | -0.920231763 | 0.09 | 0.131 | 0.00057096 | Hmgb2 | Diabetic_WT/Old_WT | Stromal |
| 1.29E-12 | -0.931636526 | 0.078 | 0.131 | 2.35E-08 | Bst2 | Diabetic_WT/Old_WT | Stromal |
| 5.82E-271 | -0.936188622 | 0.743 | 0.922 | 1.06E-266 | Rps17 | Diabetic_WT/Old_WT | Stromal |
| 6.76E-176 | -0.937077732 | 0.561 | 0.819 | 1.23E-171 | Rpl36al | Diabetic_WT/Old_WT | Stromal |
| 1.44E-10 | -0.937220644 | 0.084 | 0.134 | 2.62E-06 | Ltbp4 | Diabetic_WT/Old_WT | Stromal |
| 8.84E-15 | -0.938237201 | 0.057 | 0.111 | 1.61E-10 | Ackr3 | Diabetic_WT/Old_WT | Stromal |
| 0 | -0.940237672 | 0.952 | 0.989 | 0 | Rpl35a | Diabetic_WT/Old_WT | Stromal |
| 7.28E-16 | -0.941255472 | 0.063 | 0.121 | 1.33E-11 | Nt5dc2 | Diabetic_WT/Old_WT | Stromal |
| 0 | -0.942108753 | 0.988 | 0.998 | 0 | Rpl37a | Diabetic_WT/Old_WT | Stromal |
| 1.93E-122 | -0.944286246 | 0.354 | 0.648 | 3.51E-118 | Ndufa13 | Diabetic_WT/Old_WT | Stromal |
| 6.71E-78 | -0.947164553 | 0.438 | 0.641 | 1.22E-73 | Egr1 | Diabetic_WT/Old_WT | Stromal |
| 0 | -0.950538309 | 0.981 | 0.989 | 0 | Rpl39 | Diabetic_WT/Old_WT | Stromal |
| 8.57E-20 | -0.953337922 | 0.222 | 0.313 | 1.56E-15 | Cxcl5 | Diabetic_WT/Old_WT | Stromal |
| 1.46E-14 | -0.962800174 | 0.176 | 0.248 | 2.66E-10 | Timp3 | Diabetic_WT/Old_WT | Stromal |
| 1.55E-73 | -0.967388357 | 0.275 | 0.491 | 2.82E-69 | Atox1 | Diabetic_WT/Old_WT | Stromal |
| 1.06E-80 | -0.969372329 | 0.254 | 0.484 | 1.93E-76 | Snhg18 | Diabetic_WT/Old_WT | Stromal |
| 2.18E-35 | -0.980220141 | 0.123 | 0.244 | 3.97E-31 | Eif3c | Diabetic_WT/Old_WT | Stromal |
| 2.17E-40 | -0.982148755 | 0.147 | 0.285 | 3.95E-36 | Ndufa11 | Diabetic_WT/Old_WT | Stromal |
| 7.59E-27 | -0.988292385 | 0.096 | 0.191 | 1.38E-22 | Ndufb7 | Diabetic_WT/Old_WT | Stromal |
| 4.04E-41 | -0.988512068 | 0.151 | 0.291 | 7.37E-37 | Snrpf | Diabetic_WT/Old_WT | Stromal |
| 4.57E-74 | -0.990248083 | 0.064 | 0.229 | 8.34E-70 | Lars2 | Diabetic_WT/Old_WT | Stromal |
| 1.23E-22 | -0.991137538 | 0.085 | 0.167 | 2.23E-18 | Arpp19 | Diabetic_WT/Old_WT | Stromal |
| 2.68E-64 | -1.008350994 | 0.249 | 0.443 | 4.89E-60 | Mif | Diabetic_WT/Old_WT | Stromal |
| 7.94E-146 | -1.009216133 | 0.362 | 0.68 | 1.45E-141 | Cox6b1 | Diabetic_WT/Old_WT | Stromal |
| 5.78E-103 | -1.012560017 | 0.298 | 0.563 | 1.05E-98 | Cox7a2 | Diabetic_WT/Old_WT | Stromal |
| 0 | -1.012688189 | 0.72 | 0.934 | 0 | Atp5e | Diabetic_WT/Old_WT | Stromal |
| 1.62E-19 | -1.016463438 | 0.069 | 0.139 | 2.96E-15 | Eno1 | Diabetic_WT/Old_WT | Stromal |
| 3.75E-36 | -1.024047155 | 0.118 | 0.239 | 6.84E-32 | Ndufa1 | Diabetic_WT/Old_WT | Stromal |
| 1.37E-259 | -1.026407889 | 0.604 | 0.881 | 2.51E-255 | Elob | Diabetic_WT/Old_WT | Stromal |
| 0 | -1.028441237 | 0.908 | 0.986 | 0 | Rps26 | Diabetic_WT/Old_WT | Stromal |
| 2.91E-79 | -1.034686998 | 0.212 | 0.435 | 5.31E-75 | Ubl5 | Diabetic_WT/Old_WT | Stromal |
| 4.27E-36 | -1.035427441 | 0.213 | 0.348 | 7.79E-32 | Ccl7 | Diabetic_WT/Old_WT | Stromal |
| 4.20E-58 | -1.037472292 | 0.171 | 0.348 | 7.65E-54 | Snrpe | Diabetic_WT/Old_WT | Stromal |
| 0 | -1.042697839 | 0.949 | 0.985 | 0 | Rpl34 | Diabetic_WT/Old_WT | Stromal |
| 0 | -1.043189195 | 0.811 | 0.955 | 0 | Rpl36a | Diabetic_WT/Old_WT | Stromal |
| 2.48E-23 | -1.04415592 | 0.078 | 0.159 | 4.52E-19 | Erh | Diabetic_WT/Old_WT | Stromal |
| 3.35E-18 | -1.047405757 | 0.065 | 0.13 | 6.11E-14 | H1f0 | Diabetic_WT/Old_WT | Stromal |
| 1.06E-149 | -1.049554224 | 0.361 | 0.677 | 1.93E-145 | Atpif1 | Diabetic_WT/Old_WT | Stromal |
| 2.60E-139 | -1.049732192 | 0.428 | 0.697 | 4.74E-135 | Rps27l | Diabetic_WT/Old_WT | Stromal |
| 0 | -1.050104657 | 0.964 | 0.991 | 0 | Rpl37 | Diabetic_WT/Old_WT | Stromal |
| 3.09E-71 | -1.052588064 | 0.197 | 0.404 | 5.64E-67 | Cox6a1 | Diabetic_WT/Old_WT | Stromal |
| 4.31E-94 | -1.05871368 | 0.239 | 0.488 | 7.86E-90 | Uqcrb | Diabetic_WT/Old_WT | Stromal |
| 4.12E-22 | -1.086921077 | 0.071 | 0.147 | 7.52E-18 | Msx1 | Diabetic_WT/Old_WT | Stromal |
| 5.64E-46 | -1.091847248 | 0.136 | 0.282 | 1.03E-41 | H2afj | Diabetic_WT/Old_WT | Stromal |
| 0 | -1.092399405 | 0.624 | 0.896 | 0 | Rpl31 | Diabetic_WT/Old_WT | Stromal |
| 5.42E-193 | -1.10394366 | 0.905 | 0.969 | 9.89E-189 | Crip1 | Diabetic_WT/Old_WT | Stromal |
| 1.09E-273 | -1.111758748 | 0.743 | 0.905 | 2.00E-269 | Sec61g | Diabetic_WT/Old_WT | Stromal |
| 1.21E-99 | -1.138519971 | 0.393 | 0.576 | 2.20E-95 | Gm10260 | Diabetic_WT/Old_WT | Stromal |
| 8.64E-306 | -1.139239101 | 0.585 | 0.895 | 1.57E-301 | Cox6c | Diabetic_WT/Old_WT | Stromal |
| 8.47E-197 | -1.14055368 | 0.362 | 0.728 | 1.54E-192 | Atp5j2 | Diabetic_WT/Old_WT | Stromal |
| 5.90E-21 | -1.150264389 | 0.057 | 0.124 | 1.08E-16 | Slc39a14 | Diabetic_WT/Old_WT | Stromal |
| 5.93E-107 | -1.161223851 | 0.244 | 0.507 | 1.08E-102 | Uqcrq | Diabetic_WT/Old_WT | Stromal |
| 1.20E-15 | -1.177209962 | 0.049 | 0.102 | 2.19E-11 | Cd34 | Diabetic_WT/Old_WT | Stromal |
| 4.54E-84 | -1.185550896 | 0.177 | 0.4 | 8.28E-80 | Atp5mpl | Diabetic_WT/Old_WT | Stromal |
| 8.01E-19 | -1.193289702 | 0.059 | 0.123 | 1.46E-14 | Nr2f2 | Diabetic_WT/Old_WT | Stromal |
| 0 | -1.196131414 | 0.95 | 0.986 | 0 | S100a6 | Diabetic_WT/Old_WT | Stromal |
| 5.88E-29 | -1.197356265 | 0.067 | 0.156 | 1.07E-24 | Ndufv3 | Diabetic_WT/Old_WT | Stromal |
| 5.52E-49 | -1.200216211 | 0.111 | 0.255 | 1.01E-44 | Ndufa3 | Diabetic_WT/Old_WT | Stromal |
| 0 | -1.2070519 | 0.577 | 0.898 | 0 | Cox7c | Diabetic_WT/Old_WT | Stromal |
| 8.54E-46 | -1.208664846 | 0.655 | 0.696 | 1.56E-41 | Timp1 | Diabetic_WT/Old_WT | Stromal |
| 5.14E-16 | -1.210825914 | 0.058 | 0.115 | 9.38E-12 | Cp | Diabetic_WT/Old_WT | Stromal |
| 3.21E-41 | -1.213012289 | 0.102 | 0.227 | 5.86E-37 | Fxyd1 | Diabetic_WT/Old_WT | Stromal |
| 5.19E-178 | -1.234818583 | 0.308 | 0.655 | 9.46E-174 | Ost4 | Diabetic_WT/Old_WT | Stromal |
| 3.01E-116 | -1.238772784 | 0.203 | 0.478 | 5.48E-112 | Cox7b | Diabetic_WT/Old_WT | Stromal |
| 8.98E-204 | -1.245876079 | 0.321 | 0.698 | 1.64E-199 | Snrpg | Diabetic_WT/Old_WT | Stromal |
| 3.39E-84 | -1.24997483 | 0.16 | 0.378 | 6.19E-80 | Tomm6 | Diabetic_WT/Old_WT | Stromal |
| 3.25E-109 | -1.255768644 | 0.271 | 0.531 | 5.92E-105 | Mfap5 | Diabetic_WT/Old_WT | Stromal |
| 2.48E-19 | -1.272149685 | 0.044 | 0.103 | 4.53E-15 | Anapc13 | Diabetic_WT/Old_WT | Stromal |
| 4.30E-34 | -1.277621838 | 0.071 | 0.17 | 7.84E-30 | Bola2 | Diabetic_WT/Old_WT | Stromal |
| 2.86E-167 | -1.295382167 | 0.26 | 0.603 | 5.21E-163 | Cox5b | Diabetic_WT/Old_WT | Stromal |
| 9.04E-20 | -1.302764712 | 0.042 | 0.101 | 1.65E-15 | Ddah1 | Diabetic_WT/Old_WT | Stromal |
| 3.87E-61 | -1.314300351 | 0.109 | 0.272 | 7.05E-57 | Timm10b | Diabetic_WT/Old_WT | Stromal |
| 1.91E-53 | -1.35749809 | 0.093 | 0.237 | 3.48E-49 | Ndufb2 | Diabetic_WT/Old_WT | Stromal |
| 1.23E-147 | -1.367921476 | 0.21 | 0.524 | 2.25E-143 | Uqcr10 | Diabetic_WT/Old_WT | Stromal |
| 2.15E-122 | -1.368967551 | 0.181 | 0.457 | 3.92E-118 | Ndufa7 | Diabetic_WT/Old_WT | Stromal |
| 8.46E-58 | -1.378184998 | 0.095 | 0.247 | 1.54E-53 | Cops9 | Diabetic_WT/Old_WT | Stromal |
| 9.11E-185 | -1.387480176 | 0.272 | 0.621 | 1.66E-180 | Tmem258 | Diabetic_WT/Old_WT | Stromal |
| 1.98E-150 | -1.389552816 | 0.223 | 0.537 | 3.62E-146 | Atp5md | Diabetic_WT/Old_WT | Stromal |
| 5.20E-119 | -1.390843383 | 0.181 | 0.451 | 9.49E-115 | Uqcr11 | Diabetic_WT/Old_WT | Stromal |
| 1.28E-22 | -1.426352919 | 0.043 | 0.108 | 2.34E-18 | Cox17 | Diabetic_WT/Old_WT | Stromal |
| 2.68E-100 | -1.432337524 | 0.138 | 0.372 | 4.88E-96 | Mrpl33 | Diabetic_WT/Old_WT | Stromal |
| 0 | -1.435128342 | 0.861 | 0.98 | 0 | Rpl35 | Diabetic_WT/Old_WT | Stromal |
| 4.75E-215 | -1.440392028 | 0.24 | 0.631 | 8.67E-211 | Ndufa2 | Diabetic_WT/Old_WT | Stromal |
| 2.00E-108 | -1.442503613 | 0.157 | 0.407 | 3.65E-104 | Sod3 | Diabetic_WT/Old_WT | Stromal |
| 6.62E-217 | -1.465429806 | 0.236 | 0.63 | 1.21E-212 | Mrpl52 | Diabetic_WT/Old_WT | Stromal |
| 2.55E-98 | -1.479051658 | 0.129 | 0.357 | 4.65E-94 | Ndufc1 | Diabetic_WT/Old_WT | Stromal |
| 7.15E-85 | -1.492535322 | 0.114 | 0.316 | 1.30E-80 | Tmem256 | Diabetic_WT/Old_WT | Stromal |
| 4.01E-77 | -1.4962217 | 0.23 | 0.428 | 7.32E-73 | Cxcl12 | Diabetic_WT/Old_WT | Stromal |
| 4.07E-107 | -1.501665156 | 0.141 | 0.383 | 7.42E-103 | Dynlrb1 | Diabetic_WT/Old_WT | Stromal |
| 4.16E-103 | -1.545700788 | 0.128 | 0.361 | 7.58E-99 | S100a13 | Diabetic_WT/Old_WT | Stromal |
| 7.77E-59 | -1.546481272 | 0.078 | 0.223 | 1.42E-54 | Ndufb4 | Diabetic_WT/Old_WT | Stromal |
| 5.71E-124 | -1.550408476 | 0.142 | 0.408 | 1.04E-119 | Uqcc2 | Diabetic_WT/Old_WT | Stromal |
| 2.47E-20 | -1.582823491 | 0.117 | 0.196 | 4.50E-16 | Mustn1 | Diabetic_WT/Old_WT | Stromal |
| 1.19E-174 | -1.586707308 | 0.172 | 0.509 | 2.17E-170 | Tomm7 | Diabetic_WT/Old_WT | Stromal |
| 0 | -1.611085485 | 0.844 | 0.938 | 0 | Tmsb10 | Diabetic_WT/Old_WT | Stromal |
| 3.29E-54 | -1.614294184 | 0.152 | 0.308 | 5.99E-50 | Ly6a | Diabetic_WT/Old_WT | Stromal |
| 4.21E-57 | -1.671108976 | 0.063 | 0.197 | 7.67E-53 | Mrps21 | Diabetic_WT/Old_WT | Stromal |
| 1.14E-118 | -1.700327084 | 0.111 | 0.358 | 2.07E-114 | Dpm3 | Diabetic_WT/Old_WT | Stromal |
| 4.34E-35 | -1.701630921 | 0.033 | 0.115 | 7.92E-31 | Snhg6 | Diabetic_WT/Old_WT | Stromal |
| 1.96E-208 | -1.770621102 | 0.156 | 0.525 | 3.57E-204 | Romo1 | Diabetic_WT/Old_WT | Stromal |
| 1.81E-89 | -1.867509089 | 0.072 | 0.26 | 3.30E-85 | Ndufa5 | Diabetic_WT/Old_WT | Stromal |
| 5.43E-57 | -1.896447098 | 0.052 | 0.18 | 9.90E-53 | C3 | Diabetic_WT/Old_WT | Stromal |
| 4.66E-09 | -2.123742045 | 0.123 | 0.171 | 8.50E-05 | Apod | Diabetic_WT/Old_WT | Stromal |
| 0 | -2.216412212 | 0.855 | 0.99 | 0 | Gm10076 | Diabetic_WT/Old_WT | Stromal |
| 3.59E-45 | -2.338650226 | 0.05 | 0.156 | 6.55E-41 | Clec3b | Diabetic_WT/Old_WT | Stromal |
| 4.42E-53 | -2.383244425 | 0.072 | 0.201 | 8.05E-49 | Ifi27l2a | Diabetic_WT/Old_WT | Stromal |
| 3.46E-106 | -2.400083423 | 0.115 | 0.334 | 6.32E-102 | Spp1 | Diabetic_WT/Old_WT | Stromal |
| 7.63E-84 | -2.507736215 | 0.032 | 0.184 | 1.39E-79 | Gstp1 | Diabetic_WT/Old_WT | Stromal |
| 6.54E-58 | -2.582986424 | 0.022 | 0.128 | 1.19E-53 | Emb | Diabetic_WT/Old_WT | Stromal |
| 2.64E-126 | -2.780857612 | 0.061 | 0.284 | 4.81E-122 | AY036118 | Diabetic_WT/Old_WT | Stromal |
| 0 | -2.781726422 | 0.534 | 0.901 | 0 | Gm42418 | Diabetic_WT/Old_WT | Stromal |
| 0 | -3.171496541 | 0.178 | 0.914 | 0 | Uba52 | Diabetic_WT/Old_WT | Stromal |
| 5.03E-91 | -3.44886485 | 0.016 | 0.158 | 9.17E-87 | Gm26532 | Diabetic_WT/Old_WT | Stromal |
| 7.58E-106 | -4.746224652 | 0.008 | 0.158 | 1.38E-101 | Hp | Diabetic_WT/Old_WT | Stromal |
| 9.61E-102 | -5.249084326 | 0.006 | 0.149 | 1.75E-97 | Saa3 | Diabetic_WT/Old_WT | Stromal |
